# Fast skeletal muscle transcriptome of the Gilthead sea bream (*Sparus aurata*) determined by next generation sequencing

**DOI:** 10.1186/1471-2164-13-181

**Published:** 2012-05-11

**Authors:** Daniel Garcia de la serrana, Alicia Estévez, Karl Andree, Ian A Johnston

**Affiliations:** 1 Physiological and Evolutionary Genomics Laboratory, Scottish Oceans Institute, School of Biology, University of St Andrews, Fife, KY16 8LB, , Scotland, UK; 2 Institute for Aquaculture and Food Technology Research (IRTA), Sant Carles de la Ràpita, P.O. Box 200, 43540, Spain

**Keywords:** Teleost, Gene paralogues, Splice variants, Newbler, Roche 454, Myogenesis

## Abstract

**Background:**

The gilthead sea bream (*Sparus aurata* L.) occurs around the Mediterranean and along Eastern Atlantic coasts from Great Britain to Senegal. It is tolerant of a wide range of temperatures and salinities and is often found in brackish coastal lagoons and estuarine areas, particularly early in its life cycle. Gilthead sea bream are extensively cultivated in the Mediterranean with an annual production of 125,000 metric tonnes. Here we present a *de novo* assembly of the fast skeletal muscle transcriptome of gilthead sea bream using 454 reads and identify gene paralogues, splice variants and microsatellite repeats. An annotated transcriptome of the skeletal muscle will facilitate understanding of the genetic and molecular basis of traits linked to production in this economically important species.

**Results:**

Around 2.7 million reads of mRNA sequence data were generated from the fast myotomal of adult fish (~2 kg) and juvenile fish (~0.09 kg) that had been either fed to satiation, fasted for 3-5d or transferred to low (11°C) or high (33°C) temperatures for 3-5d. Newbler v2.5 assembly resulted in 43,461 isotigs >100 bp. The number of sequences annotated by searching protein and gene ontology databases was 10,465. The average coverage of the annotated isotigs was x40 containing 5655 unique gene IDs and 785 full-length cDNAs coding for proteins containing 58–1536 amino acids. The v2.5 assembly was found to be of good quality based on validation using 200 full-length cDNAs from GenBank. Annotated isotigs from the reference transcriptome were attributable to 344 KEGG pathway maps. We identified 26 gene paralogues (20 of them teleost-specific) and 43 splice variants, of which 12 had functional domains missing that were likely to affect their biological function. Many key transcription factors, signaling molecules and structural proteins necessary for myogenesis and muscle growth have been identified. Physiological status affected the number of reads that mapped to isotigs, reflecting changes in gene expression between treatments.

**Conclusions:**

We have produced a comprehensive fast skeletal muscle transcriptome for the gilthead sea bream, which will provide a resource for SNP discovery in genes with a large effect on production traits of commercial interest and for expression studies of growth and adaptation.

## Background

The gilthead sea bream (*Sparus aurata L*.) is widely farmed around the Mediterranean with main centres of production in Greece, Turkey, Spain and Italy. This species which is primarily marketed as fresh fish or fillets is also cultivated in the Red Sea, the Persian Gulf, and the Arabian Sea with global production reaching circa 125,000 metric tonnes in 2008 [1]. Gilthead sea bream is a protandrous hermaphrodite that can reach about 70 cm in length and 5 kg body mass. Males become sexually mature after 0.5 kg and by the second year most individuals have become female (>1.5 kg). The axial musculature or fillet is made up of serially arranged myotomes comprising ~65% of body mass containing slow, intermediate and fast muscle fibre types [2]. Fast muscle fibres comprise the bulk of the myotome. The main expansion of fast muscle fibre with growth occurs by a process called mosaic hyperplasia in which myogenic progenitor cells (MPCs) fuse to form new myotubes on the surface of existing muscle fibres giving rise to a mosaic of fibre diameters as the fish matures [3-6]. MPCs also contribute additional nuclei to the muscle fibre as it expands in length and diameter [5]. In all life history stages, myogenesis involves steps of myoblast proliferation, migration, fusion, terminal differentiation and sarcomere assembly and many of the transcription factors and signaling molecules required for the regulation of these processes have been characterised [7]. In the majority of teleost, mosaic hyperplasia in fast muscle continues until the fish reaches around 40% of its maximum body length [3-6]. Myogenesis is a highly plastic process in which internal and external signals arising from changing environmental conditions; swimming activity and nutritional inputs are integrated to modify growth patterns [8]. Embryonic temperature regime results in persistent changes in growth patterns in later life affecting the final number and size distribution of muscle fibres in adult fish [5,6,9] with potential impacts on flesh quality parameters such as texture [10].

The application of genomic technologies promises to revolutionise our understanding of the genetic and molecular basis of muscle growth and plasticity in farmed fish species; thereby increasing the efficiency and sustainability of aquaculture production. For example, the discovery of genetic polymorphisms associated with commercially important production traits such as growth rate and flesh quality would form the foundation for marker-assisted selection to produce superior strains for farming. Genomic studies could also enable bioactive nutritional components to be identified in commercial feeds and be used to accelerate the development of more sustainable diets with lower environmental impact. The genome of Atlantic cod (*Gadus morhua*) has recently been described [11] and several other farmed fish are in the process of being sequenced to draft level including rainbow trout (*Oncorhynchus mykiss*) [12], Atlantic salmon (*Salmo salar*) [13] and tilapia (*Oreochromis niloticus*) [14]. There are also significant genetic resources available for the European sea bass (*Dicentrarchus labrax*) another important species in Mediterranean aquaculture. For example, Kuhl et al [15,16] developed a complete BAC-end library from the sea bass and gilthead sea bream genomes using the three-spined stickleback (*Gasterosteus aculeatus*) genome as a reference for description and annotation. In contrast, there are only 1414 GenBank sequences and 74877 ESTs for the gilthead sea bream (revised on July 2011). These sequencing efforts have allowed the development of microarray platforms for gene expression studies [17-19] and sets of microsatellites for selection programs [16]. However, the comparative lack of genetic information is a significant handicap for the development of a serious program for genetic improvement of stocks by marker assistance-selection and for a better understanding of the molecular basis of nutrition, growth, flesh quality, reproduction and disease resistance.

Next Generation Sequencing (NGS) technologies have the potential to rapidly and cost effectively expand sequence databases for non-model organisms [20-22]. In the present study we have used Roche 454 GS FLX titanium sequencing to produce a comprehensive transcriptome of fast skeletal muscle using RNA extracted from adult and juvenile gilthead sea bream subject to different nutritional states and temperatures. The resulting transcriptome with 40-times average coverage was annotated and screened for gene paralogues, alternatively spliced transcripts and microsatellite repeat sequences.

## Results

### Transcriptome assembly

Four separate cDNA libraries were created from RNA extracted from the fast skeletal muscle of 5 pooled fish per treatment: juveniles (~0.090 kg) which had been either fed to satiation, fasted for 3–5 d (both at 21°C) or acutely transferred to 11 or 33°C over 48 h and maintained for 3-5d with continued feeding. A cDNA library was also created from RNA extracted from the fast skeletal muscle of one adult (~2 kg) fish fed ~3% body mass d^-1^. 390,000 to 490,000 reads were generated per library giving a total of ~2.7 million sequence reads (Table 1). Reads were deposited in the Sequence Read Archive (SRA) database with the accession number ERP000874 [23]. Sequence reads were assembled using Newbler v2.5 assembler (Roche, 454 Life Sciences). Newbler v2.5 used 42% of the reads to construct 43,461 isotigs over 100 bp ( Additional file 1). The total number of isotigs annotated by Blast2Go was 10,465 (Table 1 and Additional file 2). Details of blast hit and GO distributions for isotigs are provided in Additional file 3 and Additional file 4. Analysis of annotated isotigs revealed the presence of 5655 unique genes transcripts, indicating 46–50% redundancy.

**Table 1:** Number of reads obtained per experimental condition and their respective Newbler assembly results

***Assembler***	***Parameters***	***Adult***	***Fasted***	***Juvenile 21°C***	***Juvenile 30°C***	***Juvenile 11°C***	***Total assembly***
*Newbler v2.5*	*Reads*	351895	486993	439734	459853	433264	2711149
	*Reads assembled*	166460	239792	189314	258331	200313	1157833
	*Singletons*	19809	24231	19140	21125	24896	96351
	*Isotigs*	6502	9152	8407	9922	9524	50515
	*Isotigs over 100 bp*	6254	8595	8242	9554	9267	43461
	*Isotig mean lenght (bp)*	875	589	936	551	550	454
	*N50 (bp)*	1092	732	1269	672	642	679
	*Isotigs Annotated*	2634	2791	3031	3211	3029	10465

Singletons: reads not contained in the final assembly.

Isotig: contigs consistently connected by a set of reads.

N50: The value was computed by sorting all contigs from largest to smallest and by determining the minimum set of contigs whose sizes total 50% of the entire transcriptome.

### Assembly validation

**Figure 1:**
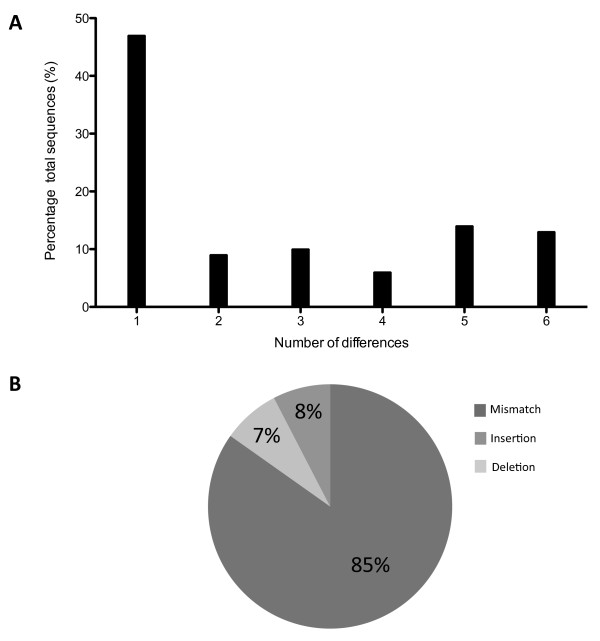
**(A) Barr chart summarizing the percentage of isotigs showing any change in their sequence compared with 80 NCBI sequences (B) Distribution of the differences between NCBI and transcriptome sequences in categories of mismatch, insertion and deletion.** Classification of the discrepancies between sequences was carried out by analysis of the ClustalW alignment.

200 full-length cDNAs (80 to 5475 bp) from gilthead sea bream were retrieved from GenBank and blasted against the assembled transcriptome resulting in 80 positive hits suitable for analysis (e-value lower than e^-140^). Pairwise alignment in ClustalW showed 25% of sequences were identical and 24% differed by only one nucleotide. The proportion of isotigs with more than one nucleotide difference is shown in Figure 1. 85% of differences were mismatches, 7.6% insertions and 7.6% deletions (Figure 1). The coverage of the transcriptome was calculated from a random selection of 200 annotated isotigs and visualized using Tablet software [24]. Coverage for annotated isotigs ranged from 3 to 1,000 times with an average coverage of 40 times.

### Assembly annotation

**Figure 2:**
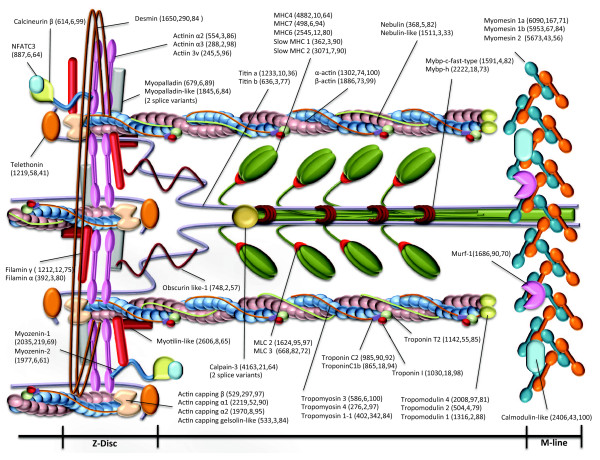
**Myofibrillar genes represented in the transcriptome mapped onto a reconstruction of a half sarcomere based on published models for filaments and M-line**[25]**and z-disc structure**[26]. Numbers on the right side of the gene name represents isotig length (bp), isotig mean coverage and percentage of identity with the zebrafish orthologue.

Annotated isotigs were attributed to 344 different KEGG pathway maps (see Additional files 5, 6 and 7). In addition, the PI3K/Akt/mTOR pathway and sarcomeric proteins maps were manually constructed to determine the actual representation of components in the transcriptome (Additional file 8 and Figure 2 respectively). In the case of sarcomeric proteins, all major components were shown to be present and isoforms of myosin heavy chain (5 isoforms), actinin (3), tropomyosin (3), actin capping protein (4), myomesin (3), filamin (2), myomezin (2), myosin light chain (2), nebulin (2), myosin binding protein (2), actin (2), titin (2), tropomodulin (3) and troponin C (2) were identified together with potential splice variants of calpain-3 and myopalladin-like (Figure 2). Components of the PI3K/Akt/mTOR pathway also occurred as multiple isoforms including AKT (2), PI3K (2), flotillin (2), integrin β-chain (4) and insulin receptor substrate (IRS) (2). The only PI3K/Akt/mTOR pathway component that was not represented in the transcriptome was the companion of mTOR RAPTOR (Additional file 8).

### Identification of full-length coding sequences (CDS) and splice variants

3,000 translated isotigs were manually blasted against the NCBI non-redundant protein (nr) database using blastp. A total of 785 full-length coding sequences were identified. Proteins ranged from 58 to 1536 amino acids (Additional file 9). The 43 genes with splice variants identified among the CDS are summarized in Additional file 10, in all cases one or more exons were predicted to be lost after splicing. Functional domains were identified using InterProScan and 14 splicing events were identified that resulted in some change in domain composition or structure which was predicted to potentially affect their biological function. Because of their biological importance, all 14 genes with a loss of functional domain were verified by PCR, resulting in the experimental confirmation of 12 genes (Table 2).

**Table 2:** Transcripts with functional domains deleted that were experimentally confirmed by PCR

**Isotig annotation**	**CDS fraction (%)**	**Transcripts coverage**	**Orthologs ID**	**Number of exons predicted**	**Exon deleted**	**IPR domain lost**	**Function**
Aspartate beta hydroxylase	37	4	ENSTNIT000000003370	3	2	IPR018939	Authophagic related protein 27 (ATG27)
Coagulation factor x	75	4	ENSGACT00000011445	13	9 and 10	IPR000294	GLA domain
						IPR000742	EGF3 domain
Nucleotide binding	88	4	ENSGACT00000002245	9	1 and 2	IPR000808	Mrp site
						IPR019591	ATPase like ParA
Bridging integrator 1	100	50	ENSGACT00000020571	18	1 to 10	IPR004148	PAR domain
						IPR003005	Amphyphysin
Paraxonase 2	87	5	ENSTNIG00000018456	14	12	No-IPR	Signal Peptide
						IPR013838	autoregulation binding site
Cathepsin H	75	4	ENSTNIG00000022446	7	1	IPR013201	Proteinase inhibitor, cathepsin propeptide
Polyadenylate-binding protein- interacting protein 2	100	4	ENSTNIG00000005741	8	6	IPR009818	Ataxin 2
Transitional endoplasmic reticulum atpase (cdc48)	100	40	ENSGACG00000018832	9	8	IPR003338	AAA + Atpase domain
						IPR009010	Aspartate descarboxylase fold
S-adenosylmethionine decarboxylase	50	5	ENSTNIG00000002751	6	5	IPR018166	adenosylmethionine descarboxylase
O-sialoglycoprotein endopeptidase	62	4	ENSGACG00000019496	6	5	IPR017860	Peptidase M22, Glycopeptidase
Zinc finger x-chromosomal protein	30	4	ENSGACG00000020679	2	1	IPR007087	Zinc finger C2H2
Dead (asp-glu-ala-asp) box polypeptide 1	57	6	ENSGACG00000011162	13	8	IPR000504	RNA recognition motif domain

### Identification of microsatellite sequences

The transcriptome was screened for potential microsatellite repeats excluding adenine repetitions, which most likely correspond to polyA tails. Around 750 potential microsatellites were detected in the total isotigs (data available on request from DG). To provide information linked to known sequences, only microsatellites localized in annotated isotigs were further studied. A total of 177 non-redundant microsatellites were identified in annotated isotigs. Dinucleotide repeated motifs were the most abundant, representing 75% of the total, followed by mononucleotide (13%), trinucleotide (11%), tetranucleotide (2%) and pentanucleotide (1%) repeats (Additional file 11). All 177 microsatellite reported in this study were found in predicted UTR regions with 40% of them linked to full-coding sequence genes.

### Identification of gene paralogues

Translated isotigs from the transcriptome were compared with mouse and teleost proteomes using Inparanoid software producing 3933 positive matches. After removal of false positives and redundant sequences 140 potential paralogues were identified. Phylogenetic analysis confirmed that 26 of these genes were paralogues. 74% of these paralogues (20) were teleost-specific, likely originating from the whole genome duplication event at the base of the teleost radiation (Table 3; paralogues sequences and nwk trees are in Additional file 12 and Additional file 13). Eight Ensembl genes used for the phylogenetical analysis (acethylcholine subunit α-1, carnitine, dysferlin, epithelial factor-3, macroglobulin 2-β, ribosomal protein L5, methylmalonate dehydrogenase and EIF43a) were well annotated and identified as paralogues, but no specific nomenclature was assigned in the database. For two gilthead sea bream paralogues (DUPD1 and FKBP1A) genes from Ensembl were identified as paralogues but not functionally annotated and blastx against the NCBI non-redundant protein database was used to confirm their identity.

**Table 3:** List of paralogues identified in the gilthead sea bream skeletal muscle transcriptome

***Paralogue Gene name***	***Gene Function***	***Fraction of CDS (%)*** ***Paralogue1/ paralogue2***	***Coverage*** ***Paralogue1/ Paralogue2***	***Paralogues identity (%)***	***Nomenclature***
Acethylcoline receptor subunit alpha 1	Ion-conducting channel	50/50	8/12	85	Alpha 1.a/1.b
Adp/atp translocase (Solute carrier family 25, SLC25)	Catalyzes the exchange of ADP and ATP across the mitochondrial inner membrane	100/100	43/87	92	SLC25 member 5 and 6
Calpain small subunit 1	Calcium-regulated thiol-protease involved in cytoskeletal remodeling	100/100	10/11	79	Calpain subunit 1a/b
Carnitine O- acetyltransferase	Carnitine acetylase is specific for short chain fatty acids	100/67	23/7	67	Carnitine O-acetyltransferase a1/a2
Dehydrogenase reductase member 7c	Putative oxidoreductase	100/99	59/32	60	DHR7SC-A/DHR7SC-B
Dysferlin interacting protein 1	Sarcolemma repair mechanism of both skeletal muscle and cardiomyocytes	90/90	39/11	71	Dysferlin1a/b
Epithelial membranse protein 3	Probably involved in cell proliferation and cell-cell interactions	100/100	4/14	68	EMP3a/b
Glioblastoma amplified sequence	Widely expressed. Most abundant in heart and skeletal muscle	100/100	39/10	80	Nipsnap2a/b
High mobility group box 1	DNA binding proteins that associates with chromatin	88/60/87	439/7/9	60	HMG1a/b HMG2
Microglobulin beta-2	Component of the class I major histocompatibility complex (MHC)	100/100	57/90	60	B2ma/b
Myomesin 185 kDa	Major component of the vertebrate myofibrillar M band	98/90	170/54	65	Myomesin1a/b
Serine threonine-protein phosphatase	Essential for cell division, and participates in muscle contractility and protein synthesis	50/50	3/4	89	Subunit alpha/gamma
Solute carrier family 38 member 5	Sodium-dependent, pyrimidine- and purine-selective. Involved in the homeostasis of endogenous nucleosides	100/90	14/5	73	Member 5a/b
Tyrosine 3 monooxigenase	Enzyme function	90/100	5/5	74	Ywhab/ywhag2
Set and mynd domain- containing protein 1	Acts as a transcriptional repressor. Essential for cardiomyocyte differentiation	95/97	154/127	76	Smyd1a/b
Dual specificity phosphatase and pro Isomerase domain containing 1	Catalyse reaction: Protein tyrosine phosphate + H2O = protein tyrosine + phosphate.	95/100	7/7	51	DUPD1a/b
Metalloproteinase inhibitor 2 precursor	Complexes with metalloproteinases and irreversibly inactivates them	80/60	4/4	62	TIMP2b/a
Retinoid x gamma	Rreceptor for retinoic acid	100/100	21/10	73	Gamma/beta
Junctophilin 1	Contributes to the stabilization of the junctional membrane complexes	85/87	7/10	65	Junctophilin 1a/b
60s ribosomal protein l5	Required for rRNA maturation and formation of the 60 S ribosomal subunits	100/100	58/186	91	Rpl5a/b
Trans-2,3-enoyl-CoA reductase	Reduces trans-2,3-stearoyl-CoA to stearoyl-CoA of long and very long chain fatty acids	98/98	11/61	75	Trec.a/b
Methylmalonate- semialdehyde Dehydrogenase	Plays a role in valine and pyrimidine metabolism. Binds fatty acyl-CoA	100/100	26/12	86	Aldha1.a/a1.b
Eukaryotic translation initiation factor 4e type 3	Its translation stimulation activity is repressed by binding to the complex CYFIP1-FMR1	100/100	28/15	73	EIF4E3a/b
Fk506-binding protein 1a	May play a role in modulation of ryanodine receptor isoform-1 (RYR-1)	100/100	52/12	82	FKBP1A.1/A.2
Splicing arginine serine-rich 11	May function in pre-mRNA splicing.	95/95	11/25	82	Srf11a/b
Kelch repeat and btb domain containing 10	Substrate-specific adapter of an E3 ubiquitin-protein ligase complex	95/100	38/15	53	Kbtb5/kbtb10

### Transcription related sequences

Transcription and its regulation is a key component of the cell’s response to its environment and an important target for physiological studies. 320 isotigs were related to transcription, including 218 transcription factors (Table 4). The majority of transcription factors identified were members of the Znf-C2H2 zinc finger sub-family (16.8%) followed by the bZIP (7.8%), beta-scaffold (7.5%), bHLH (6.5%) and general transcription factor (6.2%) families. Homeobox, High Mobility Group, Nuclear Receptors and others families represented less than 6% each of the sequences (Table 4). Over half of the non-transcription factor sequences were identified as co-factors and chromatin-associated proteins (Detailed list in Additional file 14).

**Table 4:** Transcription factor families present in the gilthead sea bream transcriptome

***Transcription Factor Family***	***Example of family member***	***Number of isotigs***	***Percentage of total transcription factors***
ZnF-C2H2	interleukin enhancer-binding factor 3	54	16.8
Chromatin-associated	yy1 transcription represor factor	32	10.0
Cofactor	e1a binding protein p300	25	10.9
Beta-scaffold	signal transducer and activator of transcription 3	24	7.5
bZIP	transcription factor jun-d	25	7.8
bHLH	hypoxia-inducible factor 3 alpha	21	6.5
General transcription factor	transcription factor 20	20	6.2
Protein-protein interaction	zinc finger and btb domain containing 33	16	5.0
Homeobox	six homeobox 1	14	4.4
Others	bromodomain adjacent to zinc finger 2b	19	5.9
Nuclear hormone receptor	Peroxisome proliferator-activated receptor alpha	15	4.7
ZnF-Others	glucocorticoid receptor dna-binding factor 1	16	5.0
High mobility group box	transcription factor sox-6 isoform 2	8	2.5
Trp-clusters	interferon regulatory factor 2	9	2.8
Forkhead	forkhead box o3	4	1.2
TEA	tea domain family member 3	3	0.9
Dwarfin	smad family member 2	3	0.9
E2F	e2f transcription factor 6	2	0.6

Genes were categorized according to the description in Uniprot [27] and TFCONES, Institute of Molecular and Cell Biology [28].

Znf-C2H2: Zinc finger domain with two conserved cysteines and two histidines co-ordinate a zinc ion.

*bZIP* basic leucine zipper.

*bHLH* basic helix loop helix transcription domain.

Trp-clusters: include Interferon regulatory transcription factors and E-twenty six transcription factors.

*TEA* transcriptional enhancer factor.

### Partial assemblies and expression analysis

**Figure 3:**
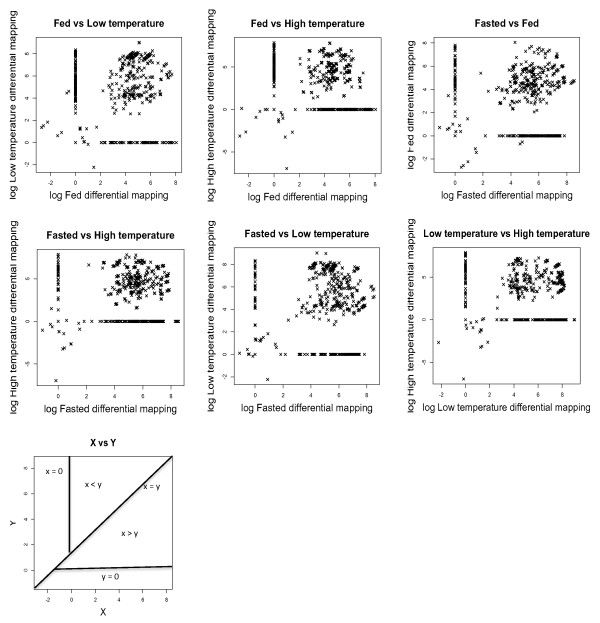
**Dot plot pairwise comparison of reads contribution to the isotigs formation from each experimental group.** Each dot represents a contig with reads from one or both treatments. X vs Y graph illustrate the relation of the number of reads between treatments in function of the region where the dots are placed.

**Figure 4:**
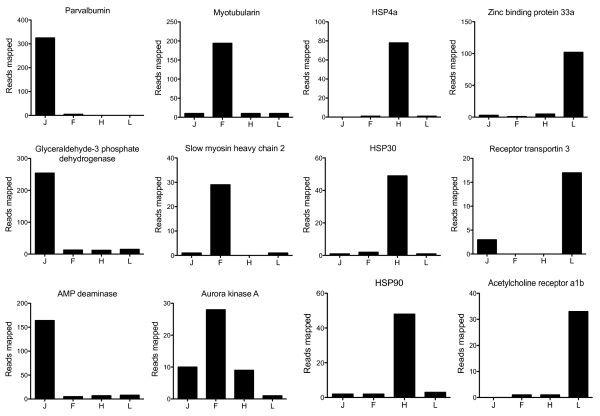
**Barr charts summarizing transcripts with significant differences between groups in the number of reads mapped.** The groups were as follows: 21°C fed (J), fasted 21°C (F), acutely transferred to 33°C fed (H) and acutely transferred to 11°C (L). All genes represented have been selected from Table 5 and have a FDR ≤ 0.01.

Since only one adult individual was sequenced expression analysis was restricted to juveniles (n = 5 per treatment). The individual assemblies for each group are summarized in Table 1. All partial transcriptomes were individually investigated to identify the 20 most expressed genes. Five genes were among the top 20 most abundant transcripts in all groups: phosphoglucose isomerase-2, calsequestrin-1, elongation factor 1-alpha, cyclin g1, parvalbumin and adenosine monophosphate deaminase-1 (Additional file 15). Pairwise comparisons of the number of reads that contributed to each isotig were made to provide information on differential gene expression between treatments (Figure 3). The top 10 genes appearing in the ranked list of significant differences between treatments are shown in Table 5 and three examples of the differences in reads mapped for each experimental group are shown in Figure 4.

**Table 5:** Expression analysis of libraries showing isotigs where reads from each experimental condition significantly contributed to the assembly

**Condition**	**Isotig number**	**Gene description**	**Orthologue accession number**	**e-value**	**p-value**	**FDR p-value**
*Fasted 21°C*	Isotig06049	Slow myosin heavy chain 2	CBN81811.1	0.0	0	0
	Isotig05152	Similar to ankyrin 2	CAM15089.1	2e-24	0	0
	Isotig01065	Calcium binding and coiled coil domain	AAI17592.1	1e-57	0	0
	Isotig30481	myotubularin-related protein 5	NP_001038623	3e-11	0	0
	Contig01939	GTPase, IMAP family member 7	ACO08772.1	2e-53	0	0
	Isotig31931	Myosin, heavy polypeptide 6	CAX12653.1	2e-06	0	0
	Isotig06490	Jeltraxin	ACN11240.1	6e-14	1,00e-05	0.004
	Isotig30481	Adenylate kinase 1-2	ACM41863.1	5e-33	0	0
	Isotig07982	Aurora kinase A-interactinng protein	ACQ58398	4e-92	0	0
	Isotig06835	Slow Troponin T2	AAV80376.1	1e-68	0	0
*Fed 21°C*	Contig02082	Parvalbumin	ACM41857.1	0.72	0	0
	Isotig05339	VHSV-induced protein	AEG78384	1e-15	1,00E-05	0.004
	Isotig17563	Putative nuclease HARBI1	XP_003200346.1	1e-16	0	0
	Isotig18811	Notch 2	BAA20535.1	9e-61	0	0
	Isotig22470	Phosphatidylinositol N-acetylglucosaminyltransferase	NP_955461.1	7e-15	0	0
	Isotig20332	Glyceraldehyde 3-dehydrogenase	XP_9741181.1	5e-14	0	0
	Contig01939	GTPase, IMAP family member 7	ACO08772.1	2e-53	0	0
	Isotig07008	Ribosomal protein L28	ACQ58416.1	5e-59	2,00E-05	0.009
	Isotig38944	AMP deaminase-1-like	XP_003212994.1	3e-04	0	0
	Isotig28974	Myosin light chain 2	AAX34414.1	2e-10	8,00E-05	0.03
*33°C*	Isotig05428	Xin actin-binding repeat containing protein 1	NP_001012377.1	0.0	0	0
	Isotig02673	Clusterin-1	NP_001117890.1	3e-139	0	0
	Isotig02981	Myosin-6-like isoform 1	XP_001923213.1	0.0	0	0
	Isotig08301	Heat shock protein 30	NP_001134440.1	6e-62	0	0
	Isotig05469	Activator of 90kda heat shock protein ATPase homolog 1	NP_997767.1	2e-145	5,00E-05	0.02
	Isotig01745	Selenoprotein L	NP_001180385.1	3e-63	8.00E-05	0.03
	Isotig07184	eEF1A2 binding protein	NP_001133224.1	1e-104	0	0
	Isotig03041	Heat shock protein 4a	NP_999881.1	0.0	5,00E-05	0.02
	Isotig18085	Srfs18	CAG06353.1	1e-45	1,00E-05	0.004
	Isotig02073	Heat shock protein 90	AAQ95586.1	0.0	0	0
*11°C*	Isotig05303	Zinc binding protein 33A	XP_694642.3	5e-61	0	0
	Isotig01520	Interferon stimulated gene 15	BAJ16365.1	4e-46	0	0
	Isotig03692	Receptor transporting protein 3	ACQ57966.1	6e-66	0	0
	Isotig03214	Nicotinic acetylcholine receptor alpha 1b	CAG09972.1	0.0	0	0
	Isotig05152	Similar to ankyrin 2	CAM15089.1	2e-24	0	0
	Isotig06087	G-rich sequence factor 1	NP_001135339.1	1e-107	0	0
	Isotig07416	Presenilin associated	ABG81447.1	6e-45	1,00E-05	0.004
	Isotig05735	Ubtf protein	AAI15119.1	5e-169	0	0
	Isotig19061	rRNA promoter binding protein	NP_671477	7e-10	0	0
	Isotig06639	C6orf64	ACO14504.1	2e-26	0	0

Gene description, orthologue accession number and e-values were obtained by blastx against the NCBI nr database. Both p-values and FDR p-value were calculated by chi-square and FDR statistic using R statistical package [70].

## Discussion

The number of genes that can be obtained from Next Generation Sequencing is higher for normalised than non-normalised libraries of the kind used in the present study; however, unbiased libraries have the advantage of yielding a higher number of full-length cDNA sequences [29]. The number of annotated isotigs in the present study was 10,465 (24% of the total) corresponding to 5,655 unique genes. The total number of annotated sequences was less than reported in the coral (*Millepora arcicornis*) transcriptome (17,000) [30], rainbow trout (*Oncorhynchus mykiss*) (376,238) [31], but similar to that obtained for eel (*Anguilla anguilla*) (5,530) [32]. However, since our transcriptome was for a single tissue type (fast skeletal muscle) a lower number of unique genes would be expected than for transcriptomes based on sequencing dsDNA libraries from multiple tissues. In addition, previous studies [30,31,33] have considered singletons to be a valid source for gene discovery whereas the 96,000 singletons (4805 annotated) obtained in the present study were not included in further analysis.

Our study is the first report of a skeletal muscle transcriptome in teleost fish and it contained 5655 unique transcripts including over 300 annotated transcripts related to transcription control, 750 microsatellite markers (177 associated with annotated istoigs) and 785 full-length cDNAs. The total number of microsatellites obtained was similar than in previous studies [34]. The transcriptome contained all known components of the sarcomere and the majority of proteins were represented by multiple isoforms even though the starting tissue for library construction comprised a pure population of fast twitch muscle fibres (Figure 2). Multiple isoforms of troponins and myosin light chains have previously been reported in single fish muscle fibres [35]. It is likely that isoforms that are expressed at specific developmental stages [36] or temperatures [37] contribute to the overall diversity of sarcomeric proteins (Figure 2). Previously, only 22 genes with splice variants have been reported in gilthead sea bream based on SANGER sequencing [38]. In the present study, 43 genes with potential splice variants were described, including 12 that affected known functional domains. This is a relatively low discovery rate given that 30% of genes in the three-spine stickleback genome were predicted to occur as multiple transcripts [39]. The reads containing the splice variant regions were analysed (data not show). In the majority of cases, the number of reads containing the deletion was lower than for the unspliced sequences, indicating lower levels of expression. In contrast, for *cytochrome c oxidase subunit 4b* and *c4b binding protein* the reads containing the deletion were more abundant and for a few genes, including *bridging integrator 1* and c*athepsin H*, the proportion of splice variants was similar. The physiological effect of the splice variants with altered functional domains was not analysed in the present work and further studies are necessary to evaluate their impact on cell physiology.

A whole genome duplication occurred in basal teleosts around 300–250 million years ago resulting in duplicate copies of many genes relative to the common ancestor with tetrapods [40]. It was estimated that in the green spotted puffer fish *Tetraodon nigriviridis* around 15% of the duplicate genes have been retained [40]. Previous transcriptomic studies in Atlantic cod (*Gadus morhua*) [41], whitefish (*Coregonus clupeamorfis*) [42] and eel (*Anguilla anguilla*) [32] have not attempted to identify paralogues. In the present study with over 10,000 transcripts annotated we expected over 400 paralogues, but only 26 could be identified. Differences between expected and the actual number of paralogues found can be explained by three main factors. The first factor is linked to sequence errors in the transcriptome. We found an error rate of 1:200 bp (99.5% accuracy) similar to previous studies [43] with 15.2% of the transcripts having insertions or deletions in their sequence. In our study, paralogue screening was based on translated isotigs, which are dramatically affected by insertions and deletions. This is because any insertion/deletions that are not multiples of three will change the open reading frame of the isotigs or introduce an in-frame stop codon. The second factor resulting in a low rate of paralogue discovery is the short length of some of the translated peptides. The majority of automatically translated isotigs represented less than 50% of the predicted sequence length (with a large number under 20%). Thus potential paralogues with short translated isotigs failed to pass the quality filters and were not considered further. Finally a very small effect will come from the assembly. Many assemblers are designed to tolerate imperfect sequence alignment to avoid missing true joins. This tolerance for error could result in false positive joins that mask polymorphisms, including paralogues [44]. This effect will be small due the divergence of the paralogues retained after the whole genome duplication, but cannot be completely discarded as a possibility.

Another advantage of using unbiased libraries is that it potentially allows information on gene expression levels to be obtained. The approach used here was to carry out pairwise comparisons between treatments counting the numbers of reads that contributed to isotigs in an assembly derived from the combined treatments (Figure 3; Table 5). The results indicate marked plasticity in gene expression with respect to nutritional status and temperature. In many cases, genes highly ranked for differential abundance between treatments corresponded to the activation of particular pathways. For example, in fed fish, stress chaperones including Hsp90 and Hsp70 and proteins associated with prevention of unfolded protein aggregation, and cytoskeleton structure maintenance was significantly elevated in 33°C compared to 21°C treatments (Figure 3 and 4; Table 5). Heat shock proteins function to increase thermal tolerance following acute exposure to high temperature stress [45]. In contrast, there was no clear pattern of gene expression in the low temperature group that can be specifically associated with treatment. This may result from low temperature inhibiting feeding and inducing a similar depression of protein synthesis and metabolism as observed for fasted fish at higher temperature, thereby masking the specific effects of acute cold stress.

Food deprivation reduces gene expression of enzymes related with glycolysis in fish liver [46] and muscle [47]. We found a decreased contribution of sequences to isotigs for genes associated with carbohydrate metabolism in fasted relative to fed treatments (Figure 3; Table 5). The fed library was also enriched for Notch-2 which is thought to control myoblast activity and be related to the asymmetric self-renewal of the muscle satellite cells through its inhibitor Numb [48,49]. It has been suggested that increased Notch expression inhibits differentiation [50] and stimulates myoblast proliferation [49]. The significant increase of Notch-2 expression and other genes related with metabolism (like GAPDH) could be an indication of higher metabolic rates and myoblast activity in this group compared to treatments exposed to stressful conditions. There was evidence for the upregulation of adenylate kinase-1 (AK) in fasted compared to fed libraries. AK acts as a sensor of the energy status of tissues [51]. An increase of some of the adenylate kinase isoforms was also reported in response to the energy imbalance during fasting in rat tissues [52]. We also found up-regulation of three sarcomeric genes (myosin polypeptide 6, slow myosin light chain 2 and slow troponin 2) consistent with shifts in myofibrillar protein isoform composition towards a slow muscle phenotype in fasted fish. Studies in Atlantic salmon also reported an increase in myosin heavy chain and the myosin light chain 2 transcripts with fasting [47].

## Conclusions

We have produced a detailed fast skeletal muscle transcriptome for the gilthead sea bream, a commercially important aquaculture species in the Mediterranean. The transcriptome contained 5655 unique annotated genes and 785 full-length coding sequences including key transcription factors, signaling molecules and structural proteins involved in myogenesis and growth. Some limitations in the identification of gene paralogues with 454 sequencing were found. In order to facilitate future genomic studies in this species a Blast server has been made available which contains 10, 465 annotated and 35,996 un-annotated isotigs together with ~ 2,700,000 ESTs [53].

## Methods

### Fish

The juvenile gilthead sea bream (*Sparus aurata L.*) used in the present study originated from a fish farm brood stock kept at the Institute de Recerca i Tecnologia Agroalimentàries (IRTA) at St Carles de la Ràpita (IRTA-SCR, Spain) and were reared from the larval to juvenile stages according to the standard production procedures of this research facility. After thirteen months, two hundred juvenile gilthead sea bream, weighing 88.1 ± 7.3 g (mean ± SD, n = 35), were selected and maintained in two 400 litre tanks (22.5 kg m^−3^) in a temperature-controlled seawater re-circulation system (IRTAmar^TM^) at a mean temperature of 21°C (20.7-21.4°C) and natural photoperiod (13 L:11D). Fish were fed a commercial diet (OptiBream^TM^, Skretting; pellet size: 2.6 mm; proximate biochemical composition: 46% protein, 18% fat, 7% ash) at a ration level of 3% (m/m) d^−1^. An adult female of 2 kg body mass that had been held at ambient temperature (annual range: 10-26°C) and natural photoperiod for several years at IRTA-SCR facilities and fed 3% body mass d^-1^ was also sampled.

In order to obtain the widest possible range of expressed transcript sub-sets, fish were exposed to different water temperatures and fasting. Experiments were conducted in 400 litres cylindrical tanks connected to a re-circulation unit in order to maintain constant water temperature and dissolved oxygen over 85% saturation. Fish (n = 5) were transferred from 21°C to 11°C or 33°C over 48 h. During the treatments fish were fed as previously described, however those maintained at 11°C, stopped feeding after their transfer to low temperature. Additionally, another group of fish maintained at 21°C were fasted for 5 days.

Since transcripts concentration will change over time with treatment fish were sampled at day 3 (n = 2) and day 5 (n = 3) following attainment of the new environmental conditions in order to obtain a broader representation of expressed genes. Fish were sacrificed using an overdose of 1:5,000 (m/v) of bicarbonate-buffered tricaine methanesulphonate (MS222, Sigma, Madrid, Spain) in seawater followed by spinal cord transection. Pure samples of fast skeletal muscle were dissected from dorsal epaxial myotomes at ~ 0.5 fork length (FL) on a pre-chilled glass plate maintained at 0–4°C. Muscle samples were flash frozen in liquid nitrogen and stored at −80°C until further analysis. Fish handling and trials were conducted in September 2009 in accordance with EC Directive 86/609/EEC for animal experimentation.

### RNA extraction and dsDNA synthesis

RNA was extracted using QIAzol (QIAGEN, Crawley - West Sussex, UK) following the manufacturer’s recommendations. The integrity of the RNA was confirmed by ethidium bromide gel electrophoresis. RNA concentration, 260/280 and 260/230 ratios were evaluated using a NanoDrop 1000 spectrophotometer (Thermo Fischer Scientific, Waltman, MA). All RNA samples extracted had a 260/280 ratio higher than 1.9 and 260/230 above 2.2. Samples from each experimental condition were pooled in equal concentrations and the RNA integrity, concentration and ratios evaluated again. The pooled RNA samples were used for the following steps.

The dsDNA synthesis was performed using a MINT cDNA synthesis kit (Evrogen, Moscow, Russia) using cDNA synthesis primer described by Meyer et al., 2009 [30] with a broken poly-T to avoid 454 sequencing problems in mono-nucleotide regions (5′-AAGCAGTGGTATCAACGCAGAGTCGCAGTCGGTACTTTTTTCTTTTTTV-3′). For an accurate evaluation of the dsDNA concentration Quati-IT™ PicoGreen® (Invitrogen, Pailey, UK) was used. PicoGreen® fluorescence was detected by a MSPx3000 qPCR machine as previously described [54].

### 454 sequencing

The transcriptome for each physiological condition was determined using Roche 454 GS FLX Titanium pyrosequencing using the service run by Genepool, University of Edinburgh, School of Biological Sciences. Each physiological condition was sequenced using a half 454-plate generating around 390,000–490,000 reads with an average length of 400 bp. Because of a technical problem an initial run of the fasted sampled yielded reads with an average length of only 300 bp and therefore this plate was repeated. Both plates yielded high quality reads and were therefore used in the subsequent global assembly.

### 454 assembly and annotation

Around 2,700,000 reads were used to generate the sea bream transcriptome. For the partial assemblies we used the reads generated from each experimental condition. For the fasted treatment partial assembly reads from the 454 plate that yielded average read lengths (400 bp) were used. Reads were assembled using Newbler 2.5 software (Roche, 454 Life-sciences) which performs well for *de novo* assembly of 454 transcriptome data [55]. Assemblies were run in a Debain Linux system, IBM x3755 8877, with 8 CPU cores (4 x dual-core AMD Opteron), 64-bit, 2.8GHz processor with 128 Gb of RAM maintained by the University of St Andrews.

To avoid assembly problems caused by the reads from highly expressed genes we trimed them using the –vs against a fasta file with the available sequences for these genes in gilthead sea bream (adapters and genes sequences used from trimming are in Additional file 16). Isotigs generated by the Newbler software are contigs that are consistently connected by subsets of reads. Isotigs are longer than contigs and were used for the annotation and transcriptome analysis.

Isotigs were Blasted and annotated using Blast2GO software [56]. Sequences were blasted using Blastx against the NCBI non-redundant protein collection (nr) database with a threshold of 10^-3^. Annotation was done with an E-value Hit Filter of 10^-6^ combined with an Annotation Cutoff of 55 and GO weighting of 5. Blast2GO also annotated sequences for functional domains using InterProScan.

### NGS and Sanger sequencing comparisons

Known sea bream sequences produced by the SANGER sequencing method were downloaded from GenBank [57] and blasted (blastn) against the sea bream transcriptome using a BLAST server [53] generated by the Genepool group. The best hits isotig/GeneBank were aligned using ClustalW [58] to determine the nature and number of differences.

### Pathway annotation

Successfully annotated isotigs were introduced in the KEGG Automatic Annotation Server (KAAS) [59]. The SBH method, optimized for ESTs annotation, was used against human, chimpanzee, orang-utan, rhesus, mouse, rat, dog, giant panda, cow, pig, horse, opossum, platypus, chicken, clawed frog, zebrafish, fruit fly and nematode pathway databases. For a more detailed reconstruction of the pathway components the PPT-Toolkit-Cell-Biology from *motifolio.com* was used.

### Identification of full-length cDNAs

Annotated isotigs were translated to the longest amino acids sequence possible using the ORF translator tool in Blast2GO package (no longer available). Sequences with more than 150 amino acids that started with a methionine or had a methionine in the first 50 amino acids were manually blasted using NCBI Blast server against nr/nt database [60]. Blast results were analysed to confirm that the translated isotig covered, at least 90% of the sequence with best hits and that cover the whole CDS.

### Microsatellite screening

Isotigs successfully annotated were used for microsatellite repeats search using msatcommander-1.0.2-alpha [61]. An isotig was considered to contain a microsatellite if contain any of the following repeated motifs: at least 10 repeated mononucleotides (other than A), 8 repeated di- or trinucleotides, or 6 repeated tetra-, penta- or hexanucleotid motifs. Their position outside coding sequences was confirmed in those microsatellites linked to annotated isotigs by analysing the translated sequences.

### Identification of splice variants

For splice variant identification we screened the list of isogroups generated during Newbler assembly. Each isogroup represents a collection of isotigs containing reads that imply connections between the isotigs. Different isotigs from a given isogroup can be used to infer splice variants. Isogroups with non-annotated isotigs were discarded. The screening was focused on detecting splice variants affecting the coding sequence. The isotigs translated sequences from each isogroup were aligned with ClustalW to detect changes in peptide sequence.

Potential splice variants were filtered a second time by blasting them against the stickleback (*Gasterosteus aculeatus*) genome where possible, or otherwise the green puffer fish (*Tetraodon nigroviridis*) genome using the Ensembl webpage BLAT algorithm [62]. Loci positive alignments were retrieved. Splice variants sequences and loci were aligned using the Spidey mRNA/genome analyser [63] to predict changes in the exon composition. Splice variants with potential changes in exon composition were submitted to InterProScan annotation to detect changes in functional domains. Genes with domain annotation that were altered by splicing were experimentally confirmed using conventional PCR.

### Identification of transcription factors (TF)

For the detection of transcription factors and molecules associated with transcription such as methyl transferases, histone acetyl transferases and others we screened isotigs annotated with GO levels related to transcription: GO:0006355 (regulation of cellular transcription), GO:0003700 (modulate transcription), GO:0003677 (interacts selectively with DNA), GO:0008134 (TF binding), GO:0033276 (protein complex able to transcription regulation), GO:0043425 (basic Helix-Loop-Helix interactive elements), GO:0016563 (any activity required for initiation or upregulation of transcription) and GO:0045941 (any transcription regulator activity). IDs were checked against a Transcription Factor database to confirm a role in transcription regulation and to categorize them into families [64] and against the Uniprot database [28].

### Identification of gene paralogues

Because no formal software has been developed specifically for paralogue screening in assemblies from Next Generation Sequencing we used an indirect approximation using the translated isotigs. A list of protein sequences of known genes from mouse (*Mus musculus*) was downloaded using BioMart tool from ESEMBL [27]. We also downloaded a list of known paralogues from different teleost species: *Takifugu rubripesTetraodon NigroviridisGasterosteus aculeatusOryzias latipes* and *Danio rerio*. Comparisons between proteins groups were performed using Inparanoid 4.0 [65]. Comparisons were performed using the gilthead sea bream translated transcriptome against one of the datasets at time. When at least two different isotigs were identified to represent the same transcript matched with a single mouse gene they were consider as potential paralogues. In addition, if two or more teleost known paralogues matched with two different isotigs they were also considered as potential paralogues. Other relations between transcripts can give similar output from Inparanoid and be included as paralogues: redundant transcripts, splice variants, sequence fragments and wrongly translated isotigs by insertions/deletions. Inparanoid output was explored by aligning translated sequences of paralogues against each other using ClustalW. This exploration allowed us to detect and trim these “False positives” from the list of potential paralogues.

### Phylogenetic analysis

The amino acids sequences of potential paralogues were blasted against the zebrafish (*Danio rerio*), stickleback (*Gasterosteus aculeatus*), takifugu (*Takifugu rubripes*), medaka (*Oryzias latipes*), green pufferfish (*Tetraodon nigroviridis*), chicken (*Gallus gallus*), frog (*Xenopus laevis*) and human (*Homo sapiens*) genomes using Essembl [62]. The sequences from the best hits were downloaded. Alignment of the potential paralogues and their orthologues was performed using the GUIDANCE web tool [66]. Only fragments with an alignment confidence score over 0.93 were used for the phylogenetic analysis. The best evolutionary model was estimated for each alignment using MEGA5 software [67]. Maximum Likelihood phylogenetic analysis was constructed, with the best evolutionary model, using the online pipeline from PhylM [68].

### Expression analysis

Reads from each experimental condition were mapped against the total isotigs from the global assembly using GS Reference Mapper (Roche, 454 Life Sciences). The number of reads per contig from each condition was extracted using the R statistical package [69]. Chi-square statistic was applied to detect significant differences in the number of reads per condition per isotig. Isotigs with less than 10 reads were excluded from the analysis. A FDR correction was applied to all p-values below 0.05. Plot graphs comparing the contribution of reads from each experimental condition to the isotig formation were constructed using R package.

## Competing interests

The authors declare that they have no competing interests.

## Author’s contributions

IAJ conceived the study, DGDLS was responsible for RNA extraction, dsDNA synthesis, sequence assembly and bioinformatics, AE and KA were responsible for fish husbandry and AE assisted with sample preparation, IAJ and DGDLS wrote the manuscript. All authors read and approved the manuscript.

## Supplementary Material

Additional file 1 Isotig nucleotide sequences from the gilthead sea bream fast muscle transcriptome. 454 reads were assembled using Newbler version 2.5 after trimming adaptors used for dsDNA synthesis and *in silico* normalization. Click here for file

Additional file 2 Gilthead sea bream transcriptome sequences annotated. Annotation was performed by Blast2GO software. Sequences were blasted with Blastx algorithms against the NCBI non-redundant protein collection (nr) database with a threshold of 10^−3^. Annotation was done with an E-value Hit Filter of 10^−6^ combined with an Annotation Cutoff of 55 and GO weighting of 5. Click here for file

Additional file 3 Gilthead sea bream fast muscle transcriptome annotation results. All graphs and figures were prepared using Blast2GO software (A) Data distribution of the gilthead sea bream transcriptome annotation results. NoBlastHits represents transcripts with no blastx results. NoMapping category represents blasted sequences with no gene ontology (GO) annotation. NoAnnot sequences are isotigs with a preliminary GO annotation (mapping) which failed to arrive to the minimal annotation threefold. Annot barr represents isotigs that were successfully blasted and annotated (B) Annotated transcripts length (bp) distribution (C) Transcripts BLAST results e-value distribution (e-value threefold 10^−3^). Sequences were blasted by blastx algorithm against the nr protein database from NCBI (D) Species distribution of top hits from whole fast muscle 454 transcriptome with significant homology (<10^−3^) to searches from the NCBI nr database. Only the best/first sequence alignments for a given Blast result for all blast results are show (E) Percentage of sequences annotated as a function of their length (bp) (F) Distribution of transcripts from gilthead sea bream transcriptome with major categories of level 3 molecular function from GO analysis (G) Distribution of transcripts from gilthead sea bream transcriptome with major categories of level 3 biological process from GO analysis (H) Distribution of transcripts from gilthead sea bream transcriptome with major categories of level 6 cellular component from GO analysis. Click here for file

Additional file 4 Transcripts from fast muscle gilthead sea bream (*Sparus aurata* L.) 454 transcriptome summarized by their gene ontology annotation (GO) according to Biological Process, Molecular Function and Cellular Component. Table only shows the most abundant GO terms from each category as a percentage and the number of transcripts associated to this level. Click here for file

Additional file 5 Kyoto Encyclopaedia of Genes and Genomes (KEGG) maps present in the gilthead sea bream fast muscle transcriptome. Transcripts were annotated to KEGG maps using the automatic annotator tool KAAS [60]. Click here for file

Additional file 6 KEGG annotation results. File contains the KEGG maps where isotigs were successfully mapped. Pathway element in green boxes indicates the components represented by at least, one isotig. Gilthead sea bream isotigs were automatically annotated to KEGG maps using KAAS website [60]. The SBH method, optimized for ESTs annotation, was used against human, chimpanzee, orang-utan, rhesus, mouse, rat, dog, giant panda, cow, pig, horse, opossum, platypus, chicken, clawed frog, zebrafish, fruit fly and nematode pathway databases. Click here for file

Additional file 7 Hierarchical information of the isotigs mapped to the KEGG pathways maps using the automatic annotation tool KAAS. File can be opened using KegHier from [60] when maps are displayed the pathway components with an isotig mapped are labeled in red. Click here for file

Additional file 8 PI3K/Akt and mTOR genes represented in the transcriptome mapped onto a reconstruction of the pathway based on KEGG maps (KO:04150 and KO:04910). AMPKα/β/γ, 5′-AMP-activated protein kinase catalytic subunit alpha/beta/gamma; AKT2, Rac-beta serine/threonine protein kinase 2; AKT3, Rac-beta serine/threonine protein kinase 3; ATG1, Serine/threonine-protein kinase ULK1; CBL-B, E3-Ubiquitin-protein kinase CBL; CIP4 (TRIP10), Thyroid hormone receptor interactor 10; eIF4B, Eukaryotic translation initiation factor 4B; eIF4E, Eukaryotic translation initiation factor 4E; Exo-70, Exocyst complex component 7; GYS, Glycogen synthase; GSK3a, Glycogen synthase kinase 3-alpha; GLUT4, Solute carrier family 2, facilitated glucose transporter member 4; HIF1α, Hypoxia inducible factor 1-alpha; HIF3α, Hypoxia inducible factor 3-alpha; IGF, insulin-like growth factor; IGFBP, insulin-like growth factor binding protein; IRS, insulin receptor substrate; mTOR, Mammalian target of rapamycin; p110, Phosphatidylinositol 3-kinase catalytic subunit; p85, Phosphatidylinositol 3-kinase regulatory subunit; PDPK1, 3-phosphoinositide dependent protein kinase 1; PPP1R, Protein phosphatase 1 regulatory subunit; PPP1C, Protein phosphatase 1 catalytic subunit; PHKG, Phosphorylase b kinase gamma; PHK1-B, Phosphorylase kinase alpha-beta subunit; PPAR, Peroxisome proliferation-activated receptor; PYG, Starch phosphorylase; RICTOR, Rapamycin-insensitive companion of mTOR; RAPTOR, Regulatory-associated protein of mTOR; RPS70K, p70 Ribosomal protein S6 kinase; RHEB2, Ras homolog enriched brain; STRADα, STE20 related kinase adaptor alpha; S6, Ribosomal protein S6; TSC, Tuberous sclerosis; TC10 (RHOQ), Ras homolog gene family member Q; VEGF-A1, Vascular endothelial growth factor A1; v-CRK, Proto-oncogen C-crk; 4EBP1, Eukaryotic translator factor 4E binding protein. Numbers associated with the gene name represents isotig length (bp), isotig mean coverage and percentage of identity with the zebrafish orthologue. Click here for file

Additional file 9 Isotig amino acid sequences from gilthead sea bream transcriptome that covers over 90% of the gene coding sequence. Isoigs were translated to peptides using the Blast2GO ORF translation tool. The percentage of CDS coverage was estimated by blasting translated sequences against the NCBI non-redundant protein database. Click here for file

Additional file 10 Predicted amino acids sequences of splice variants. Click here for file

Additional file 11 Gilthead sea bream fast muscle transcriptome microsatellite summary. Microsatellites were identified using msatcommander-1.0.3-alpha and verified their UTR localization by blastx comparison against the NCBI non-redundant protein (nr) database. Click here for file

Additional file 12 Gilthead sea bream paralogues sequences and their alignments. Alignments have been done using ClustalW. Click here for file

Additional file 13 Phylogenetic analysis of paralogues. The amino acids sequences were blasted against the zebrafish (*Danio rerio*), stickleback (*Gasterosteus aculeatus*), Takifugu (*Takifugu rubripes*), medaka (*Oryzias latipes*), green puffer fish (*Tetraodon nigroviridis*), chicken (*Gallus gallus*), frog (*Xenopus laevis*) and human (*Homo sapiens*) genomes using Essembl [63]. Alignment of the sequences was performed using the GUIDANCE web tool [67]. The best evolutionary model was estimated for each alignment using MEGA5 software. Maximum Likelihood phylogenetic analysis was constructed, with the best evolutionary model, using PhylM [69]. Click here for file

Additional file 14 Detailed list of transcription related isotigs found in the gilthead sea bream transcriptome. Click here for file

Additional file 15 The 20 most abundant isotigs from gilthead sea bream partial assemblies. Abundant isotigs were defined as the ones with the highest number of reads. Name in red are genes found in all conditions top 20. Name in blue are genes found in only one condition. Click here for file

Additional file 16 Adapters and abundant genes sequences used for assembly trimming and normalisation respectively. Click here for file
