# Supplementary figures and images for: Fast skeletal muscle transcriptome of the Gilthead sea bream (Sparus aurata) determined by next generation sequencing (part 1 of 3)
Source: BMC Genomics. 2012 May 11;13:181. doi: 10.1186/1471-2164-13-181 (PMC3418159; doi:10.1186/1471-2164-13-181)

**A**


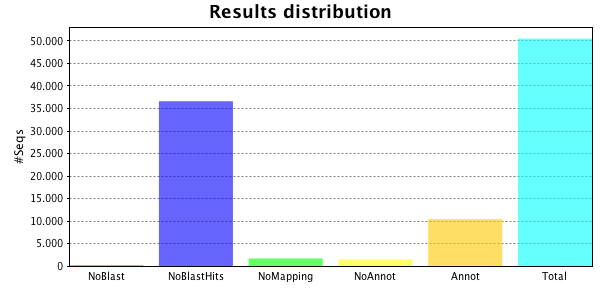


**B
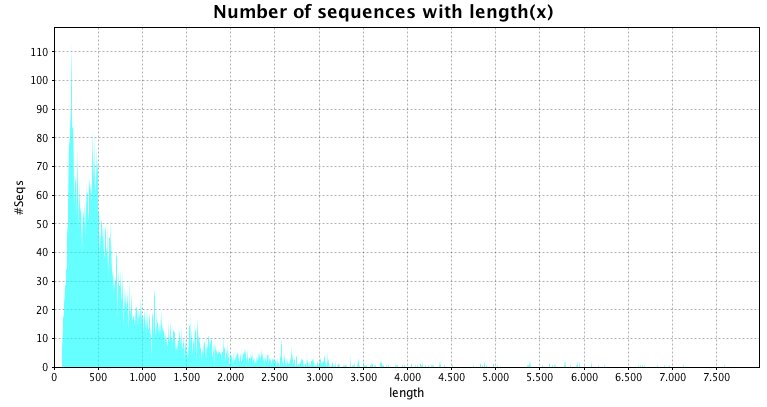
**

**C**

**
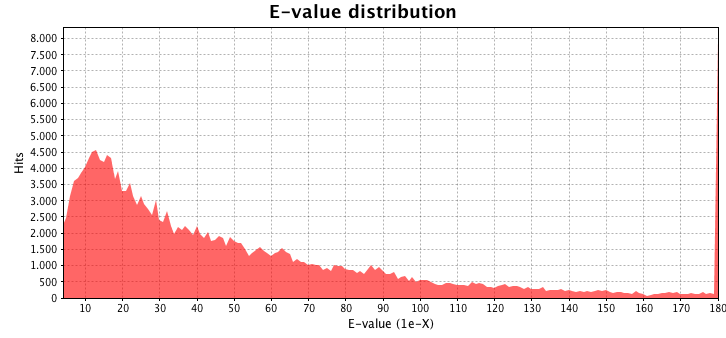
**

**D
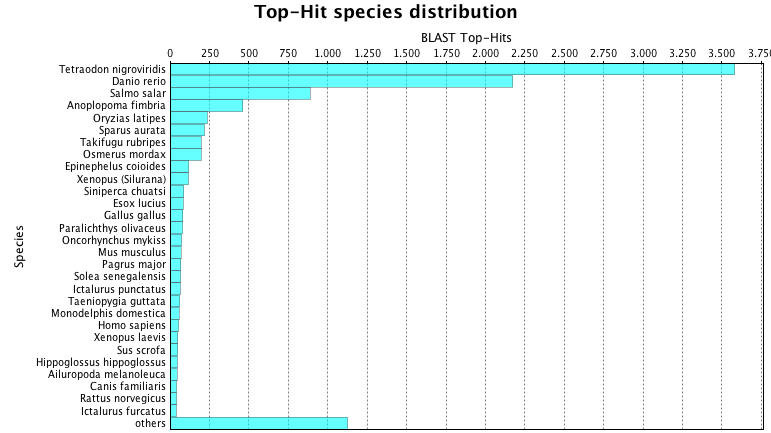
**

**E**

**
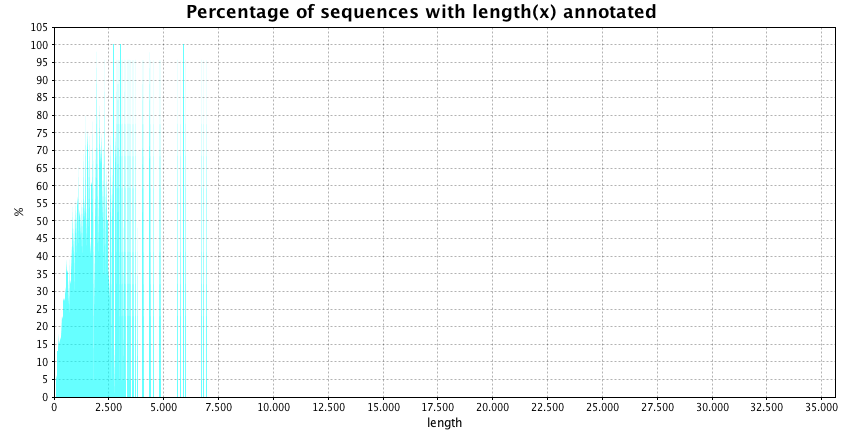
**

**F
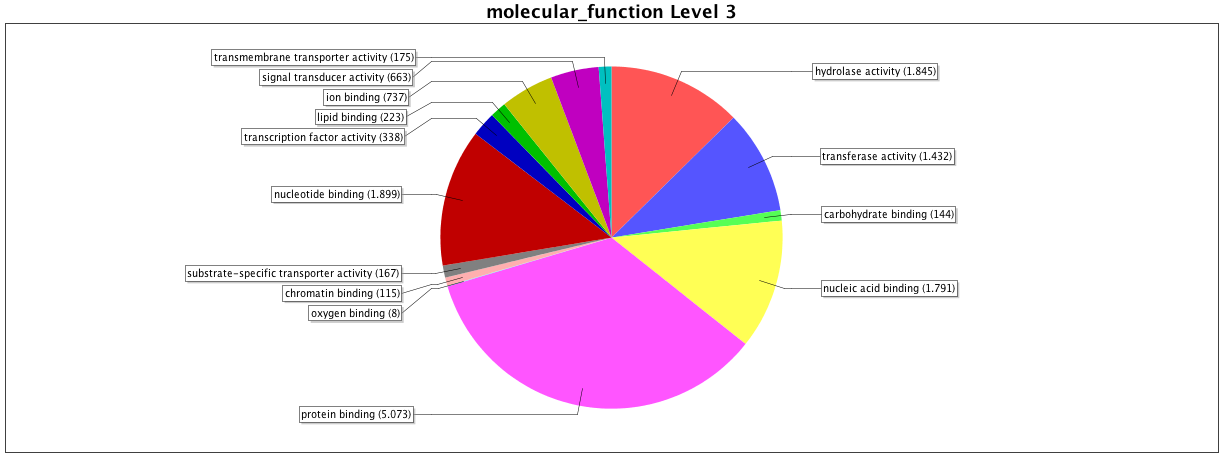
**

**G**

**
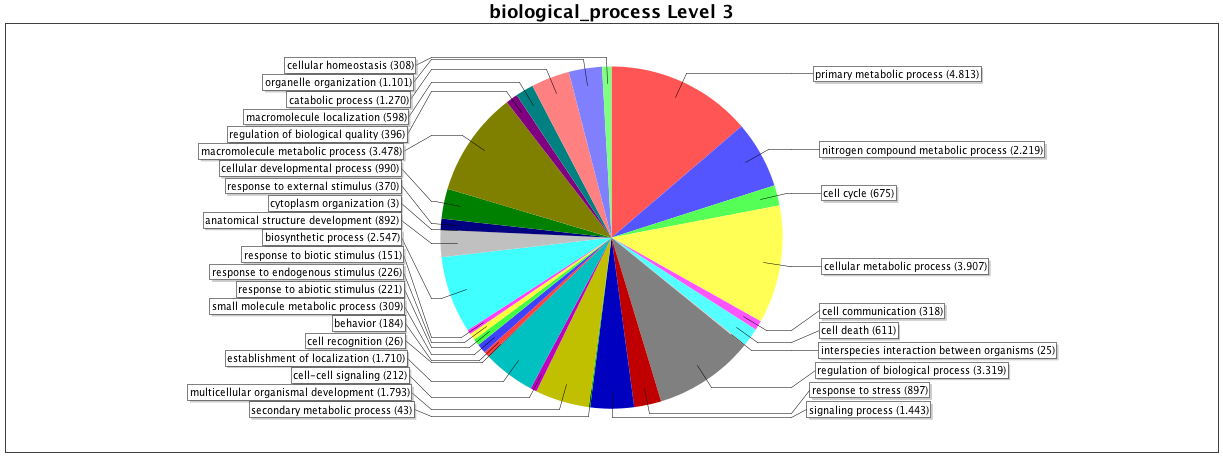
**

**H**

**
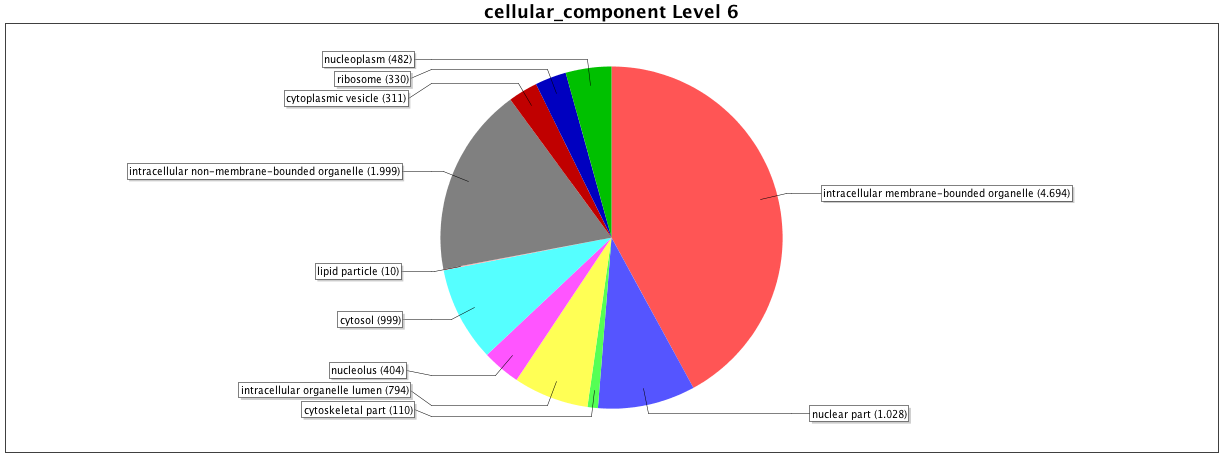
**

Supplement: Additional file 3 — Gilthead sea bream fast muscle transcriptome annotation results. All graphs and figures were prepared using Blast2GO software (A) Data distribution of the gilthead sea bream transcriptome annotation results. NoBlastHits represents transcripts with no blastx results. NoMapping category represents blasted sequences with no gene ontology (GO) annotation. NoAnnot sequences are isotigs with a preliminary GO annotation (mapping) which failed to arrive to the minimal annotation threefold. Annot barr represents isotigs that were successfully blasted and annotated (B) Annotated transcripts length (bp) distribution (C) Transcripts BLAST results e-value distribution (e-value threefold 10−3). Sequences were blasted by blastx algorithm against the nr protein database from NCBI (D) Species distribution of top hits from whole fast muscle 454 transcriptome with significant homology (<10−3) to searches from the NCBI nr database. Only the best/first sequence alignments for a given Blast result for all blast results are show (E) Percentage of sequences annotated as a function of their length (bp) (F) Distribution of transcripts from gilthead sea bream transcriptome with major categories of level 3 molecular function from GO analysis (G) Distribution of transcripts from gilthead sea bream transcriptome with major categories of level 3 biological process from GO analysis (H) Distribution of transcripts from gilthead sea bream transcriptome with major categories of level 6 cellular component from GO analysis. [file 1471-2164-13-181-S3.docx]

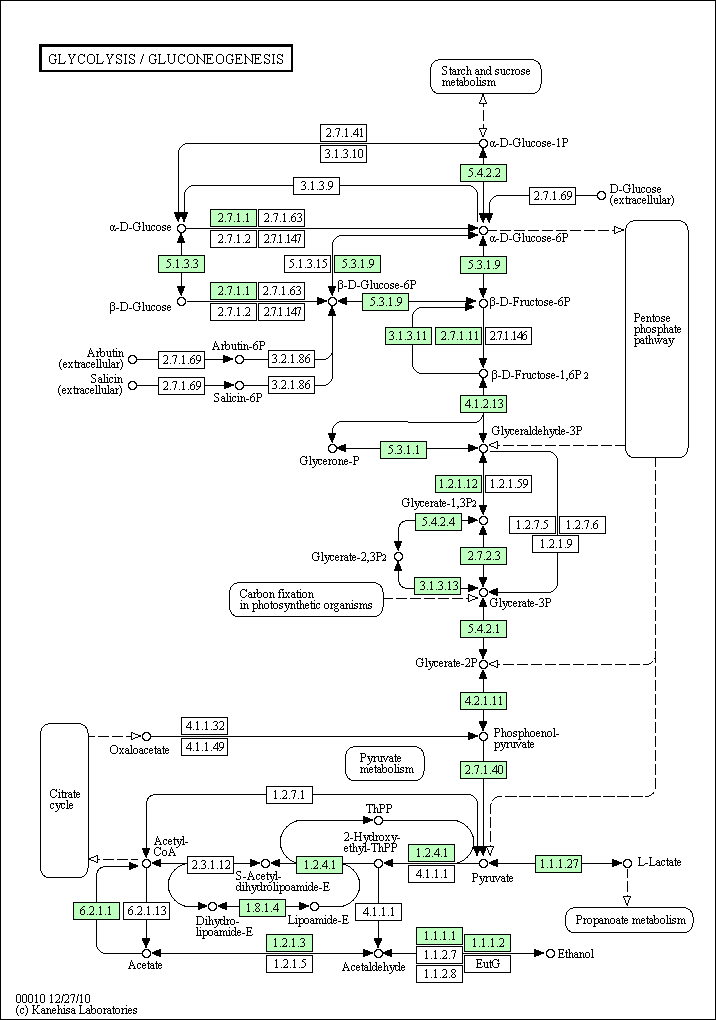

Supplement: Additional file 6 — KEGG annotation results. File contains the KEGG maps where isotigs were successfully mapped. Pathway element in green boxes indicates the components represented by at least, one isotig. Gilthead sea bream isotigs were automatically annotated to KEGG maps using KAAS website [60]. The SBH method, optimized for ESTs annotation, was used against human, chimpanzee, orang-utan, rhesus, mouse, rat, dog, giant panda, cow, pig, horse, opossum, platypus, chicken, clawed frog, zebrafish, fruit fly and nematode pathway databases. [file 1471-2164-13-181-S6.tar › map/map00010.png]

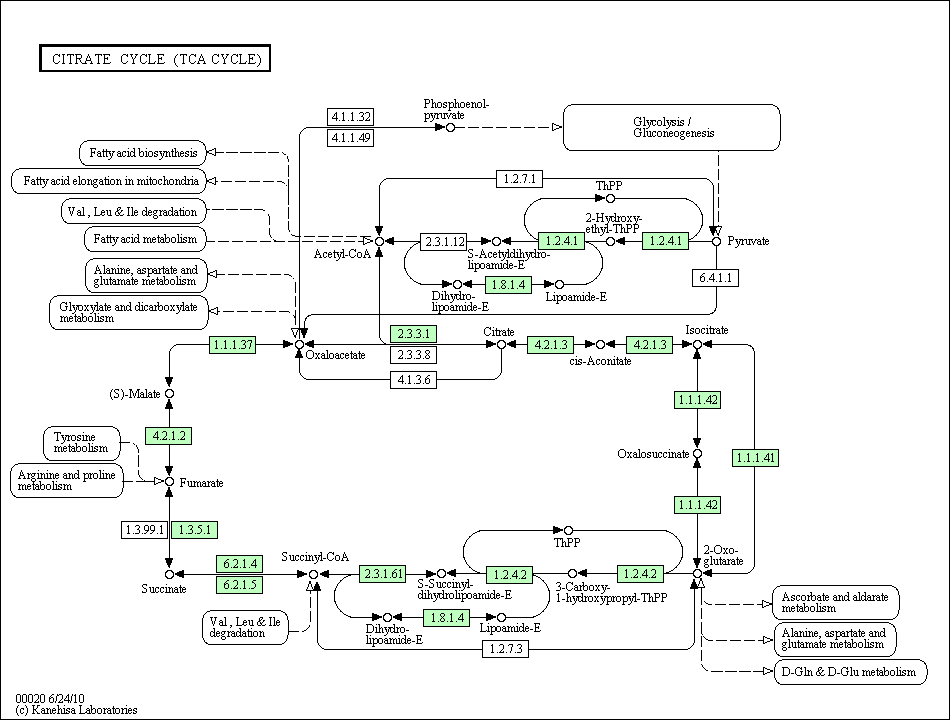

Supplement: Additional file 6 — KEGG annotation results. File contains the KEGG maps where isotigs were successfully mapped. Pathway element in green boxes indicates the components represented by at least, one isotig. Gilthead sea bream isotigs were automatically annotated to KEGG maps using KAAS website [60]. The SBH method, optimized for ESTs annotation, was used against human, chimpanzee, orang-utan, rhesus, mouse, rat, dog, giant panda, cow, pig, horse, opossum, platypus, chicken, clawed frog, zebrafish, fruit fly and nematode pathway databases. [file 1471-2164-13-181-S6.tar › map/map00020.png]

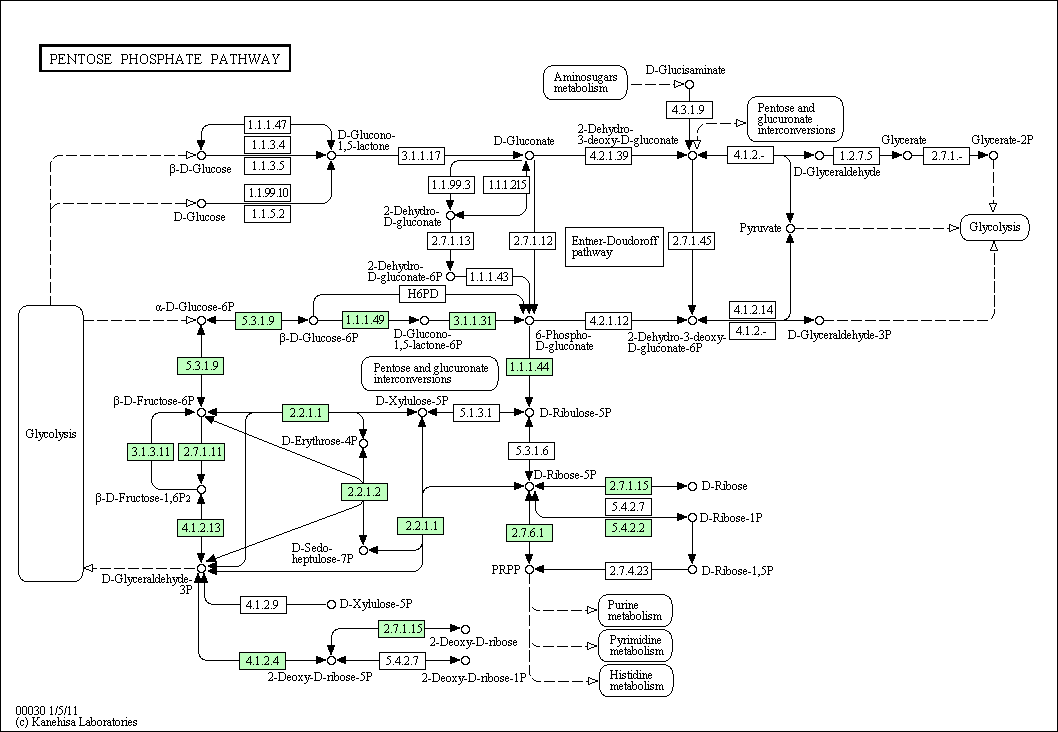

Supplement: Additional file 6 — KEGG annotation results. File contains the KEGG maps where isotigs were successfully mapped. Pathway element in green boxes indicates the components represented by at least, one isotig. Gilthead sea bream isotigs were automatically annotated to KEGG maps using KAAS website [60]. The SBH method, optimized for ESTs annotation, was used against human, chimpanzee, orang-utan, rhesus, mouse, rat, dog, giant panda, cow, pig, horse, opossum, platypus, chicken, clawed frog, zebrafish, fruit fly and nematode pathway databases. [file 1471-2164-13-181-S6.tar › map/map00030.png]

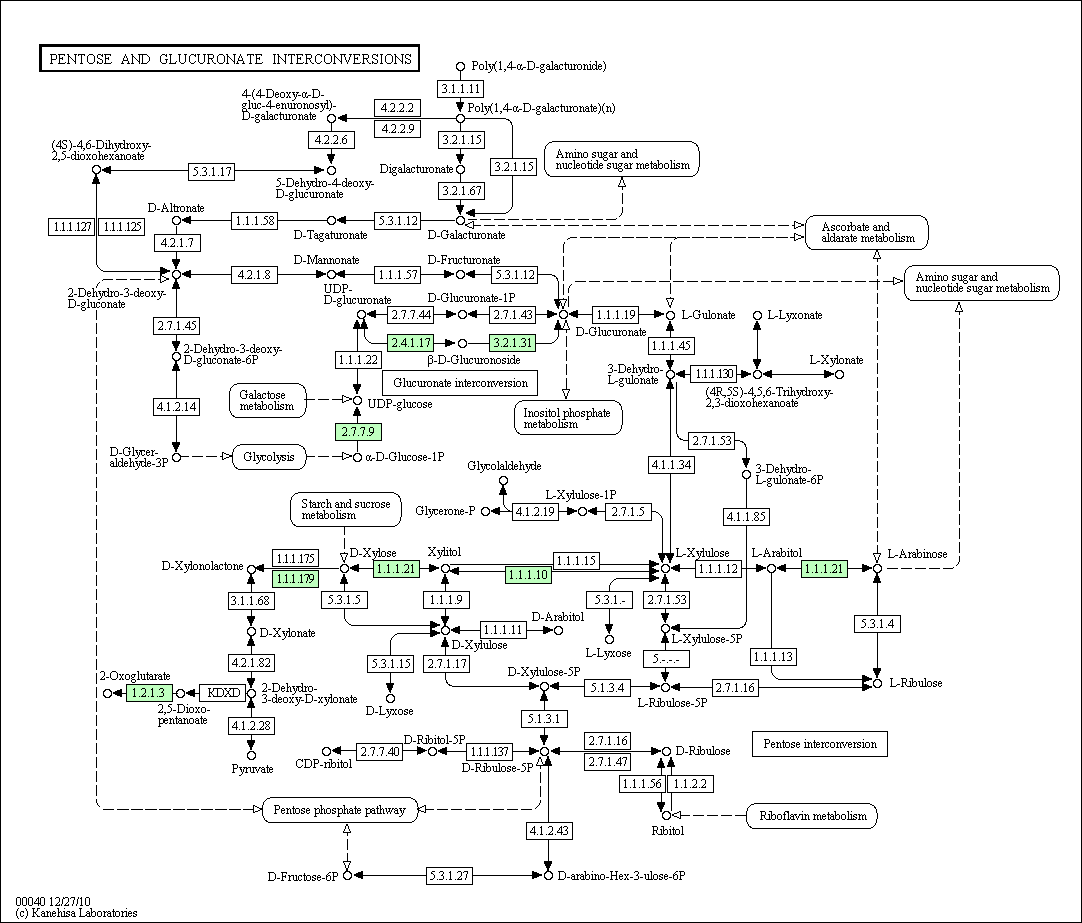

Supplement: Additional file 6 — KEGG annotation results. File contains the KEGG maps where isotigs were successfully mapped. Pathway element in green boxes indicates the components represented by at least, one isotig. Gilthead sea bream isotigs were automatically annotated to KEGG maps using KAAS website [60]. The SBH method, optimized for ESTs annotation, was used against human, chimpanzee, orang-utan, rhesus, mouse, rat, dog, giant panda, cow, pig, horse, opossum, platypus, chicken, clawed frog, zebrafish, fruit fly and nematode pathway databases. [file 1471-2164-13-181-S6.tar › map/map00040.png]

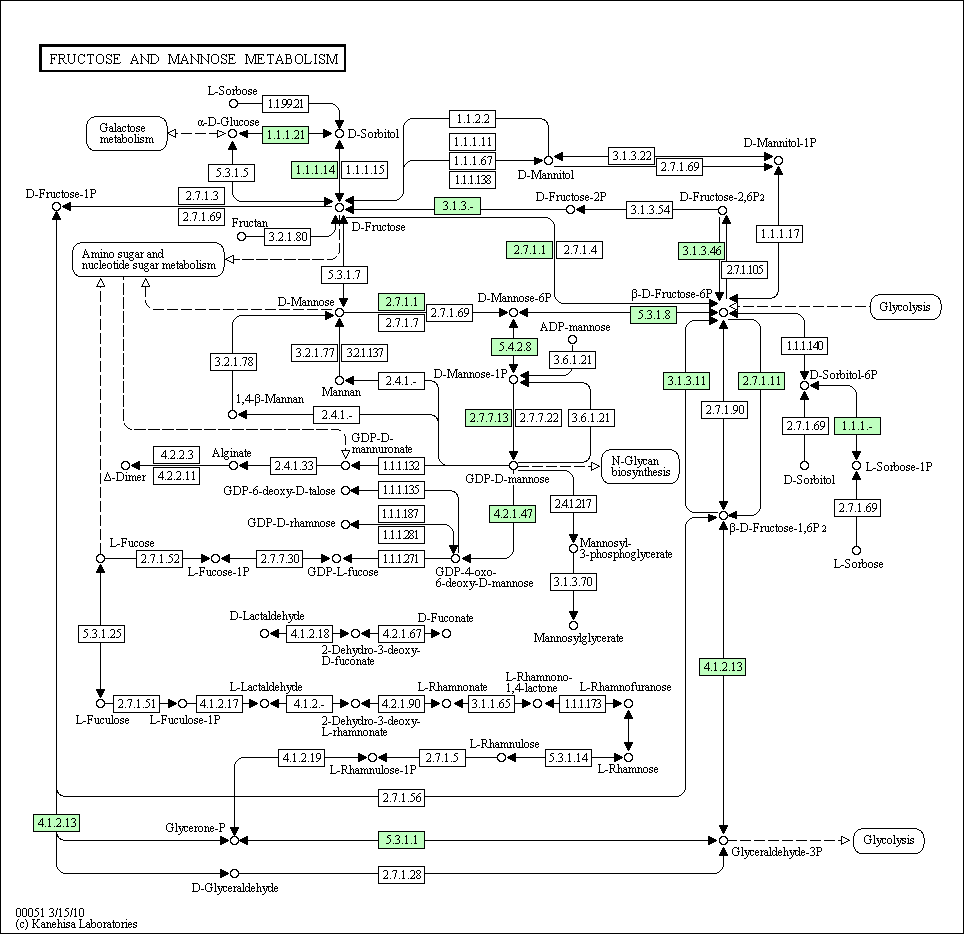

Supplement: Additional file 6 — KEGG annotation results. File contains the KEGG maps where isotigs were successfully mapped. Pathway element in green boxes indicates the components represented by at least, one isotig. Gilthead sea bream isotigs were automatically annotated to KEGG maps using KAAS website [60]. The SBH method, optimized for ESTs annotation, was used against human, chimpanzee, orang-utan, rhesus, mouse, rat, dog, giant panda, cow, pig, horse, opossum, platypus, chicken, clawed frog, zebrafish, fruit fly and nematode pathway databases. [file 1471-2164-13-181-S6.tar › map/map00051.png]

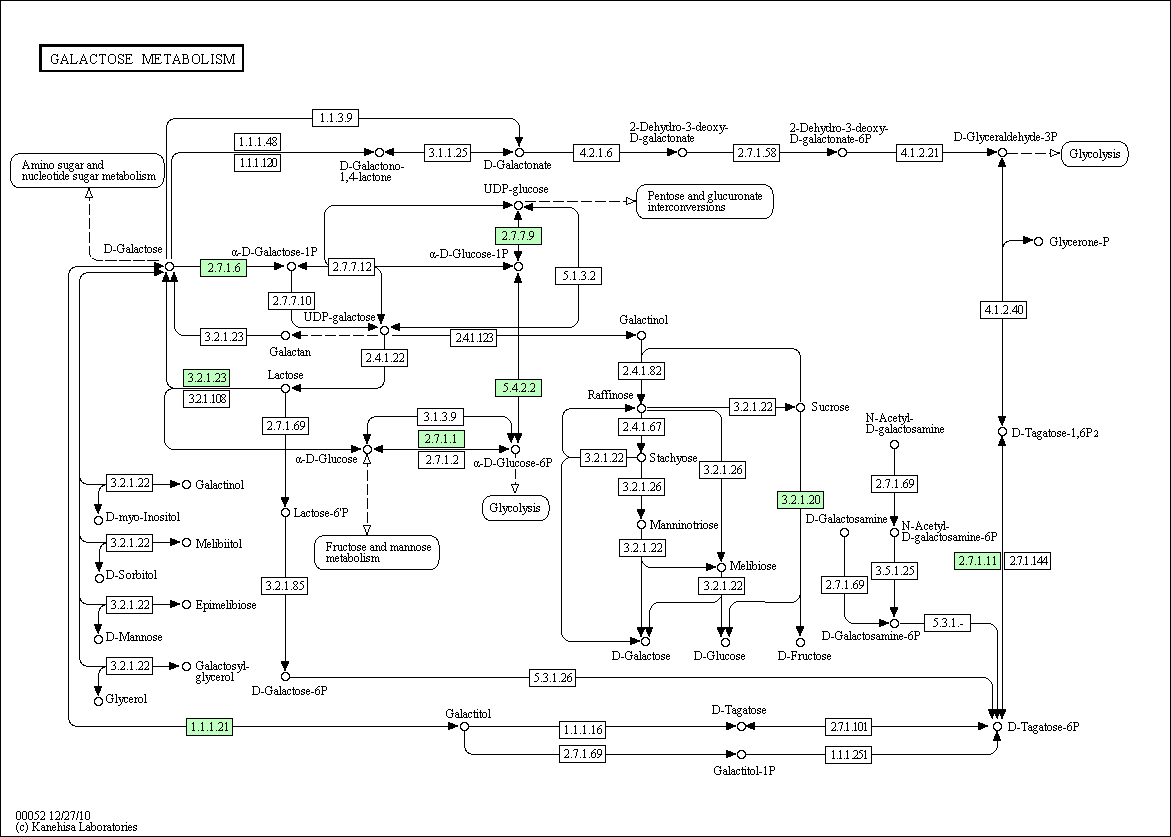

Supplement: Additional file 6 — KEGG annotation results. File contains the KEGG maps where isotigs were successfully mapped. Pathway element in green boxes indicates the components represented by at least, one isotig. Gilthead sea bream isotigs were automatically annotated to KEGG maps using KAAS website [60]. The SBH method, optimized for ESTs annotation, was used against human, chimpanzee, orang-utan, rhesus, mouse, rat, dog, giant panda, cow, pig, horse, opossum, platypus, chicken, clawed frog, zebrafish, fruit fly and nematode pathway databases. [file 1471-2164-13-181-S6.tar › map/map00052.png]

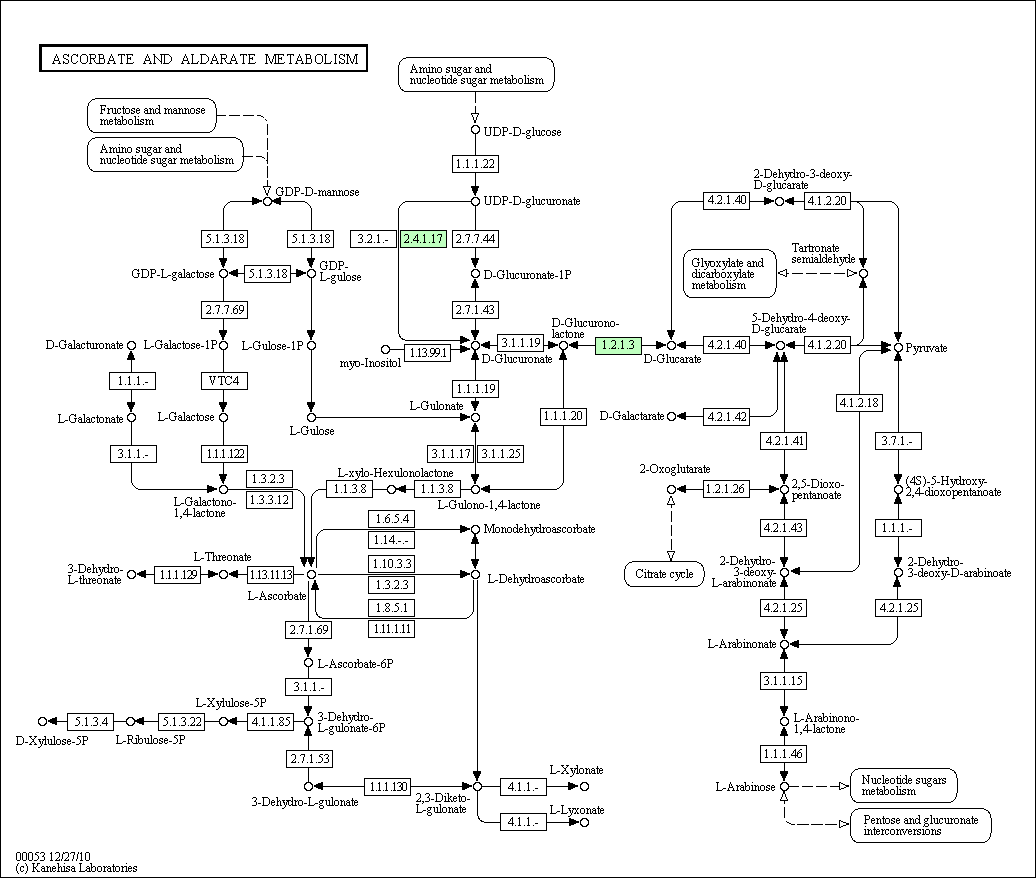

Supplement: Additional file 6 — KEGG annotation results. File contains the KEGG maps where isotigs were successfully mapped. Pathway element in green boxes indicates the components represented by at least, one isotig. Gilthead sea bream isotigs were automatically annotated to KEGG maps using KAAS website [60]. The SBH method, optimized for ESTs annotation, was used against human, chimpanzee, orang-utan, rhesus, mouse, rat, dog, giant panda, cow, pig, horse, opossum, platypus, chicken, clawed frog, zebrafish, fruit fly and nematode pathway databases. [file 1471-2164-13-181-S6.tar › map/map00053.png]

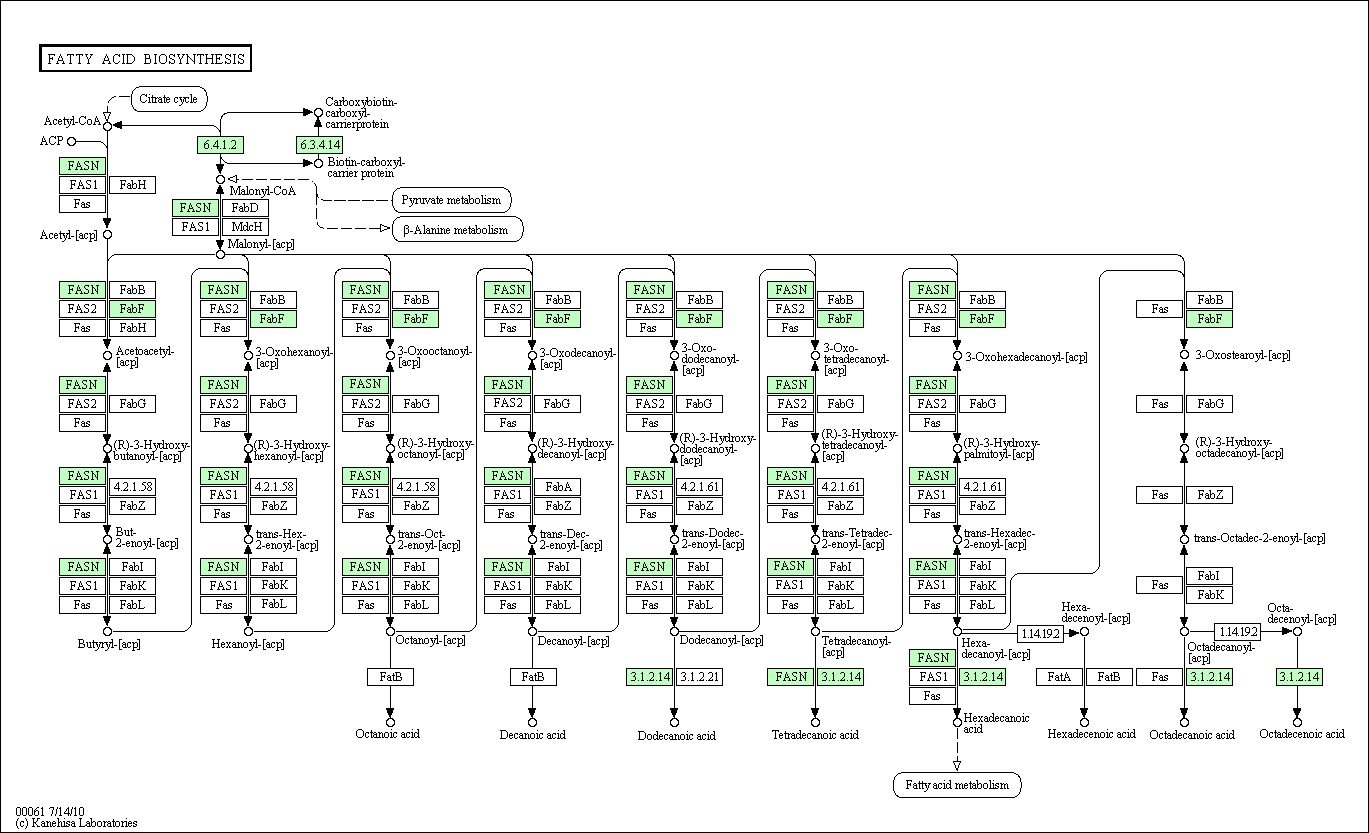

Supplement: Additional file 6 — KEGG annotation results. File contains the KEGG maps where isotigs were successfully mapped. Pathway element in green boxes indicates the components represented by at least, one isotig. Gilthead sea bream isotigs were automatically annotated to KEGG maps using KAAS website [60]. The SBH method, optimized for ESTs annotation, was used against human, chimpanzee, orang-utan, rhesus, mouse, rat, dog, giant panda, cow, pig, horse, opossum, platypus, chicken, clawed frog, zebrafish, fruit fly and nematode pathway databases. [file 1471-2164-13-181-S6.tar › map/map00061.png]

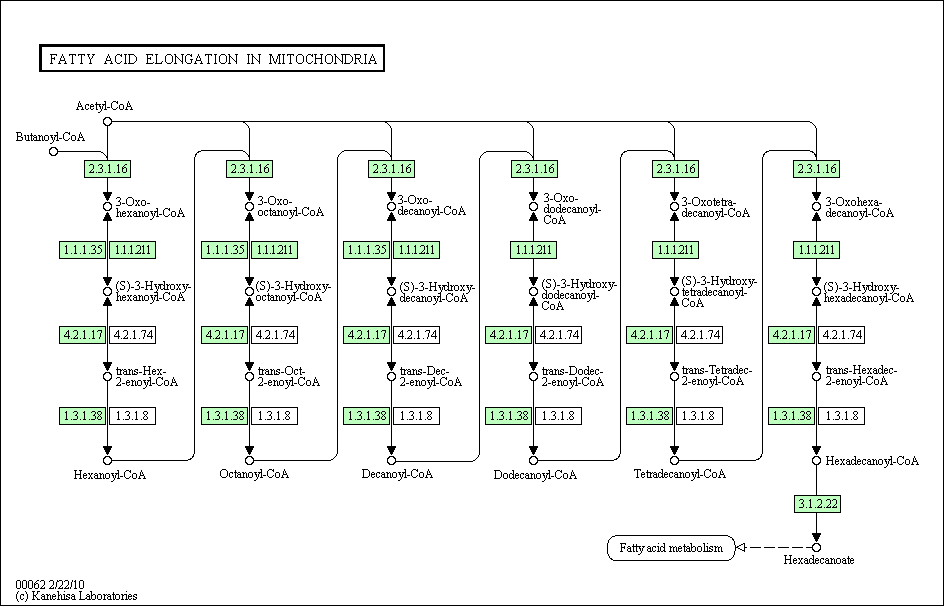

Supplement: Additional file 6 — KEGG annotation results. File contains the KEGG maps where isotigs were successfully mapped. Pathway element in green boxes indicates the components represented by at least, one isotig. Gilthead sea bream isotigs were automatically annotated to KEGG maps using KAAS website [60]. The SBH method, optimized for ESTs annotation, was used against human, chimpanzee, orang-utan, rhesus, mouse, rat, dog, giant panda, cow, pig, horse, opossum, platypus, chicken, clawed frog, zebrafish, fruit fly and nematode pathway databases. [file 1471-2164-13-181-S6.tar › map/map00062.png]

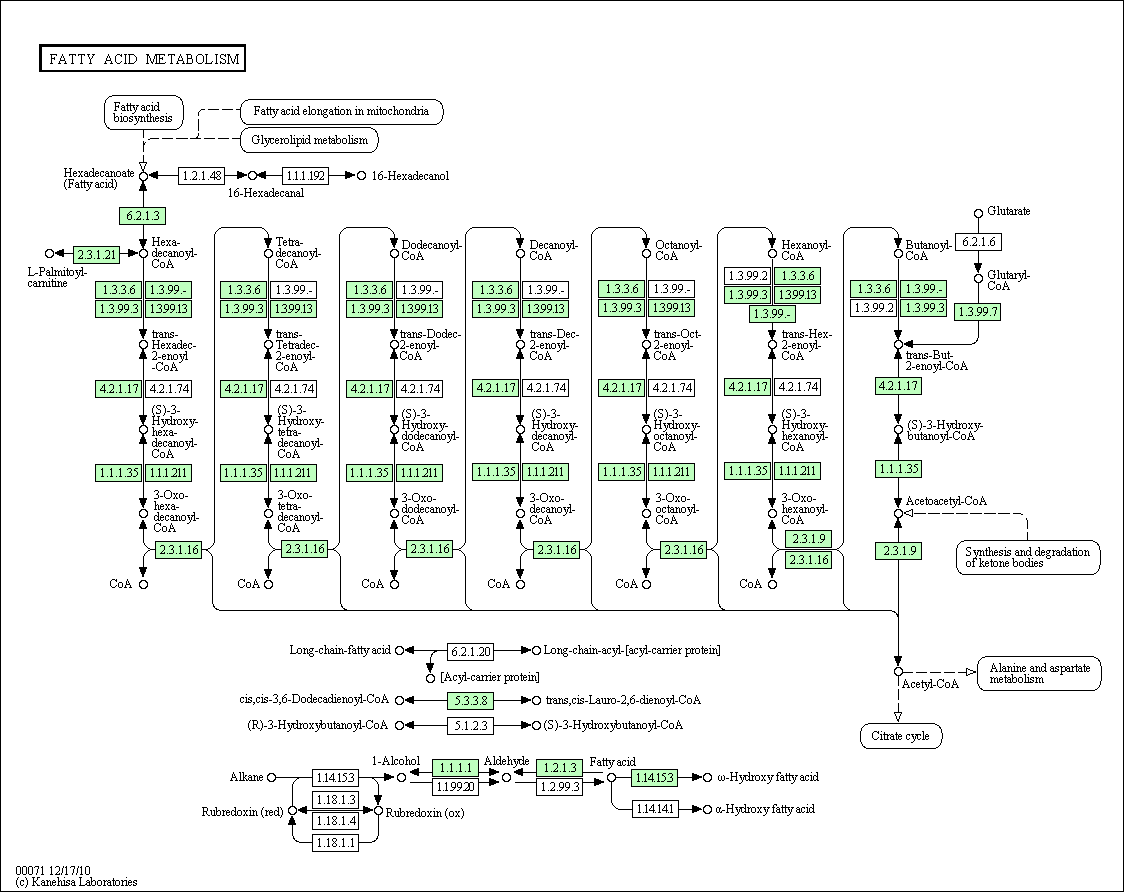

Supplement: Additional file 6 — KEGG annotation results. File contains the KEGG maps where isotigs were successfully mapped. Pathway element in green boxes indicates the components represented by at least, one isotig. Gilthead sea bream isotigs were automatically annotated to KEGG maps using KAAS website [60]. The SBH method, optimized for ESTs annotation, was used against human, chimpanzee, orang-utan, rhesus, mouse, rat, dog, giant panda, cow, pig, horse, opossum, platypus, chicken, clawed frog, zebrafish, fruit fly and nematode pathway databases. [file 1471-2164-13-181-S6.tar › map/map00071.png]

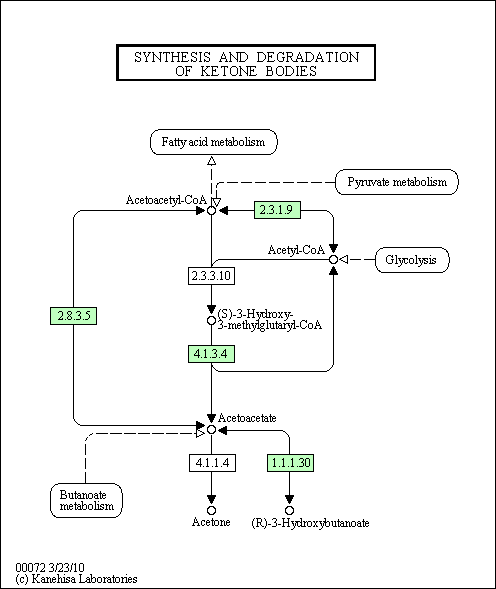

Supplement: Additional file 6 — KEGG annotation results. File contains the KEGG maps where isotigs were successfully mapped. Pathway element in green boxes indicates the components represented by at least, one isotig. Gilthead sea bream isotigs were automatically annotated to KEGG maps using KAAS website [60]. The SBH method, optimized for ESTs annotation, was used against human, chimpanzee, orang-utan, rhesus, mouse, rat, dog, giant panda, cow, pig, horse, opossum, platypus, chicken, clawed frog, zebrafish, fruit fly and nematode pathway databases. [file 1471-2164-13-181-S6.tar › map/map00072.png]

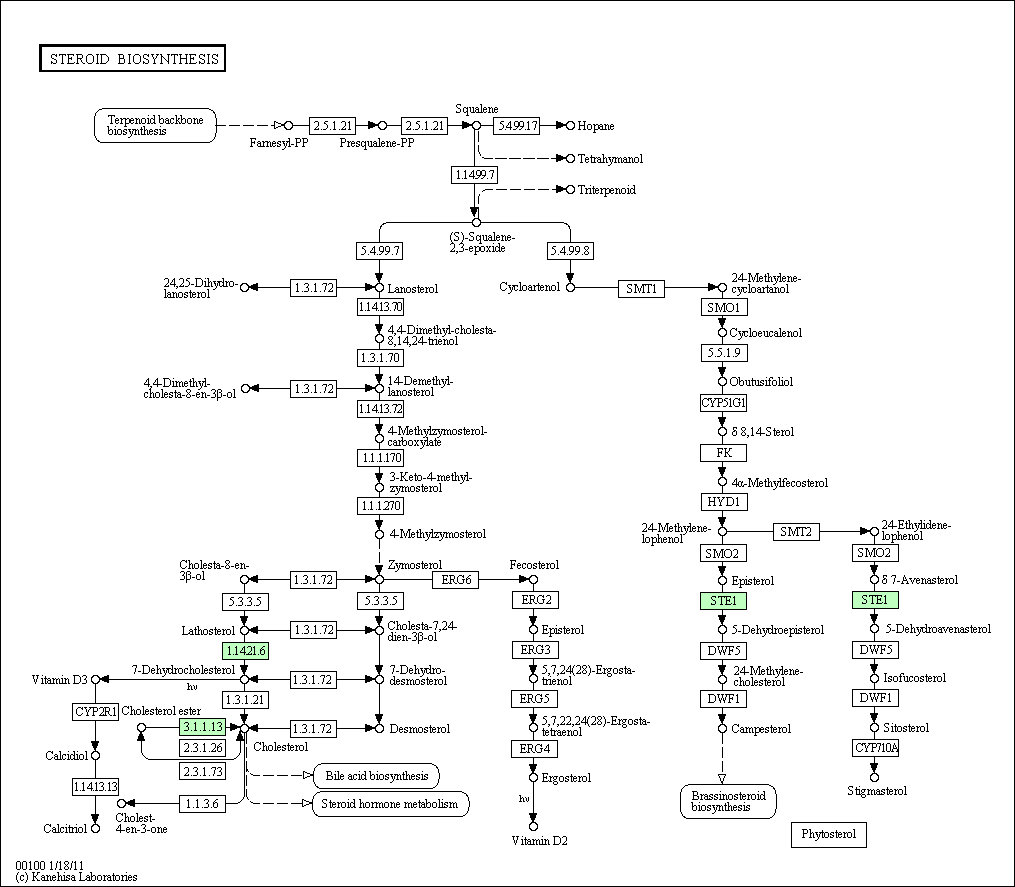

Supplement: Additional file 6 — KEGG annotation results. File contains the KEGG maps where isotigs were successfully mapped. Pathway element in green boxes indicates the components represented by at least, one isotig. Gilthead sea bream isotigs were automatically annotated to KEGG maps using KAAS website [60]. The SBH method, optimized for ESTs annotation, was used against human, chimpanzee, orang-utan, rhesus, mouse, rat, dog, giant panda, cow, pig, horse, opossum, platypus, chicken, clawed frog, zebrafish, fruit fly and nematode pathway databases. [file 1471-2164-13-181-S6.tar › map/map00100.png]

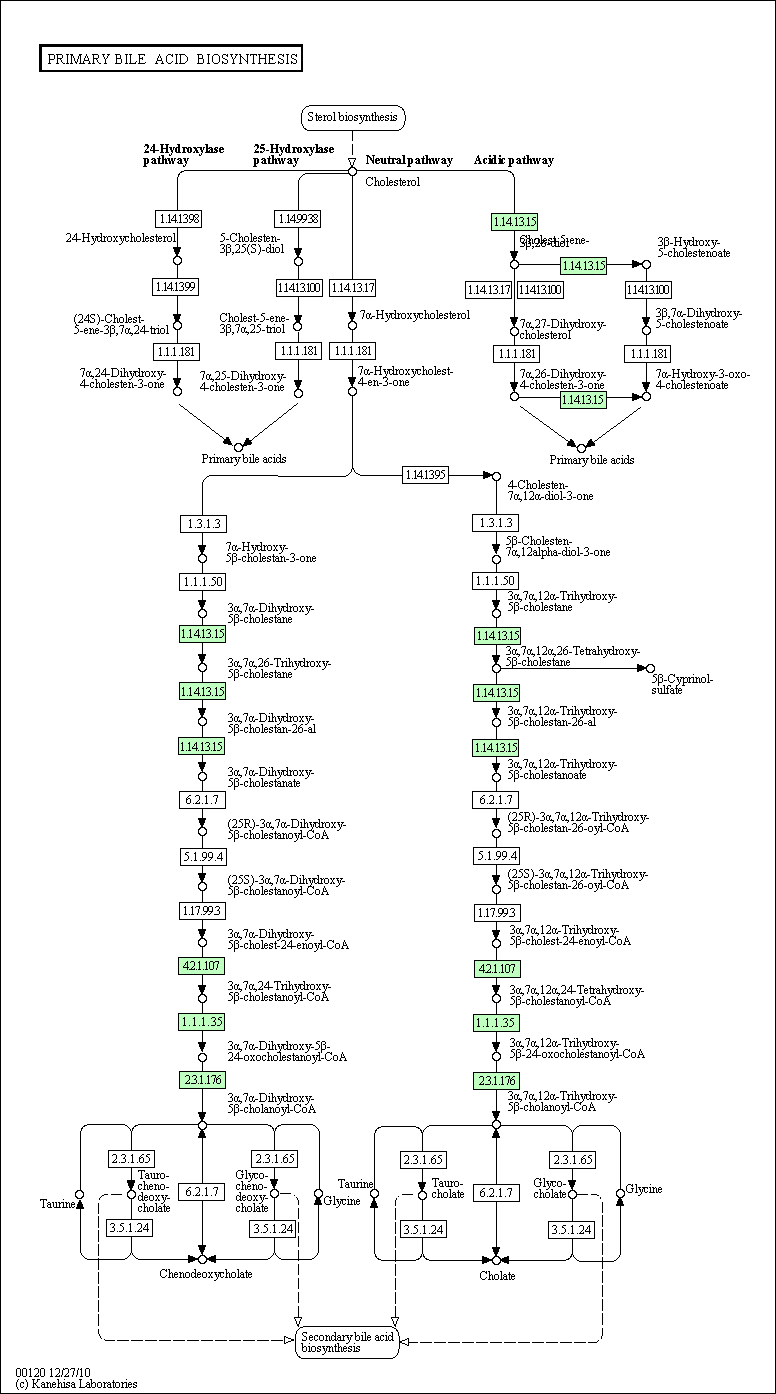

Supplement: Additional file 6 — KEGG annotation results. File contains the KEGG maps where isotigs were successfully mapped. Pathway element in green boxes indicates the components represented by at least, one isotig. Gilthead sea bream isotigs were automatically annotated to KEGG maps using KAAS website [60]. The SBH method, optimized for ESTs annotation, was used against human, chimpanzee, orang-utan, rhesus, mouse, rat, dog, giant panda, cow, pig, horse, opossum, platypus, chicken, clawed frog, zebrafish, fruit fly and nematode pathway databases. [file 1471-2164-13-181-S6.tar › map/map00120.png]

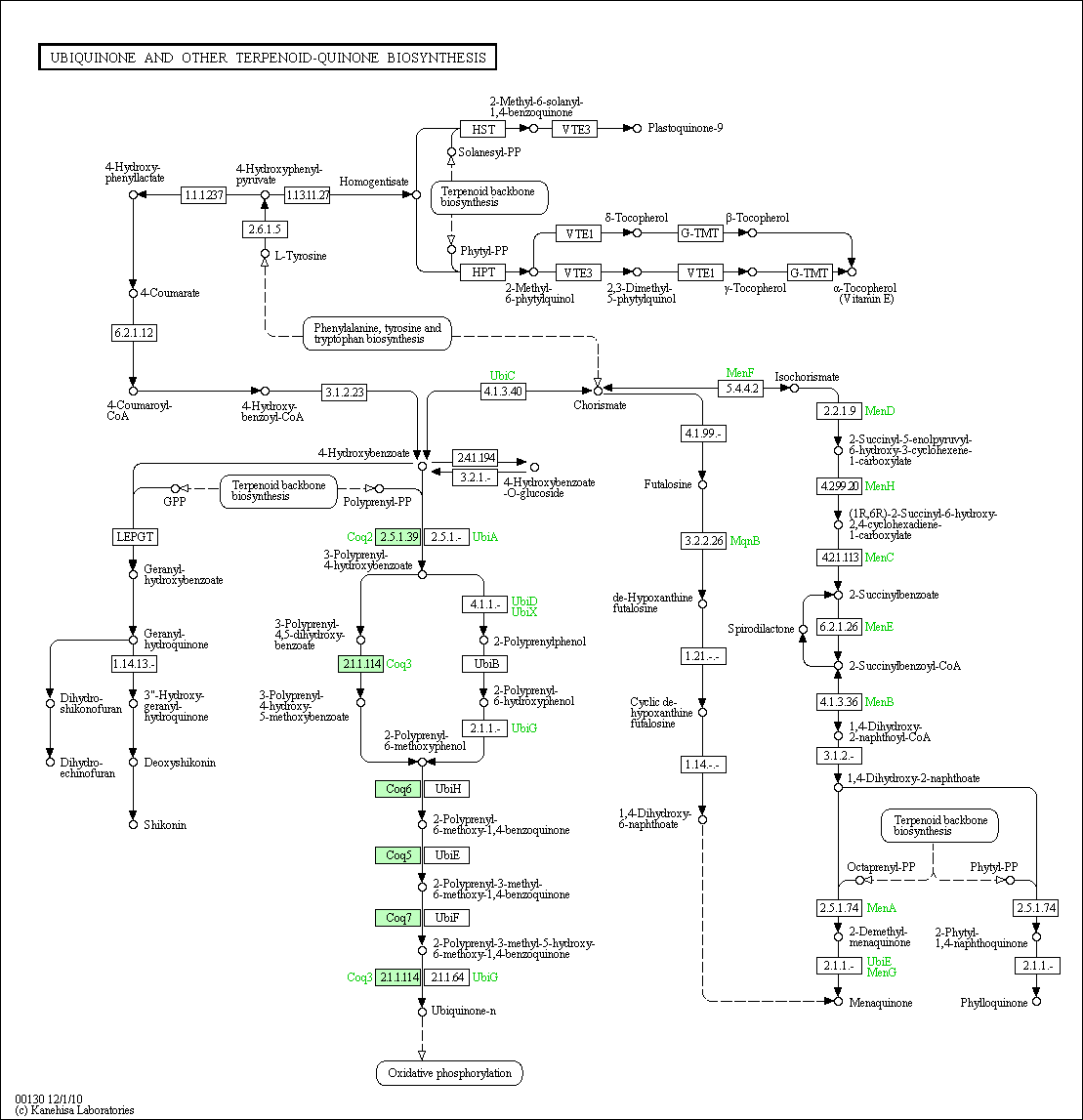

Supplement: Additional file 6 — KEGG annotation results. File contains the KEGG maps where isotigs were successfully mapped. Pathway element in green boxes indicates the components represented by at least, one isotig. Gilthead sea bream isotigs were automatically annotated to KEGG maps using KAAS website [60]. The SBH method, optimized for ESTs annotation, was used against human, chimpanzee, orang-utan, rhesus, mouse, rat, dog, giant panda, cow, pig, horse, opossum, platypus, chicken, clawed frog, zebrafish, fruit fly and nematode pathway databases. [file 1471-2164-13-181-S6.tar › map/map00130.png]

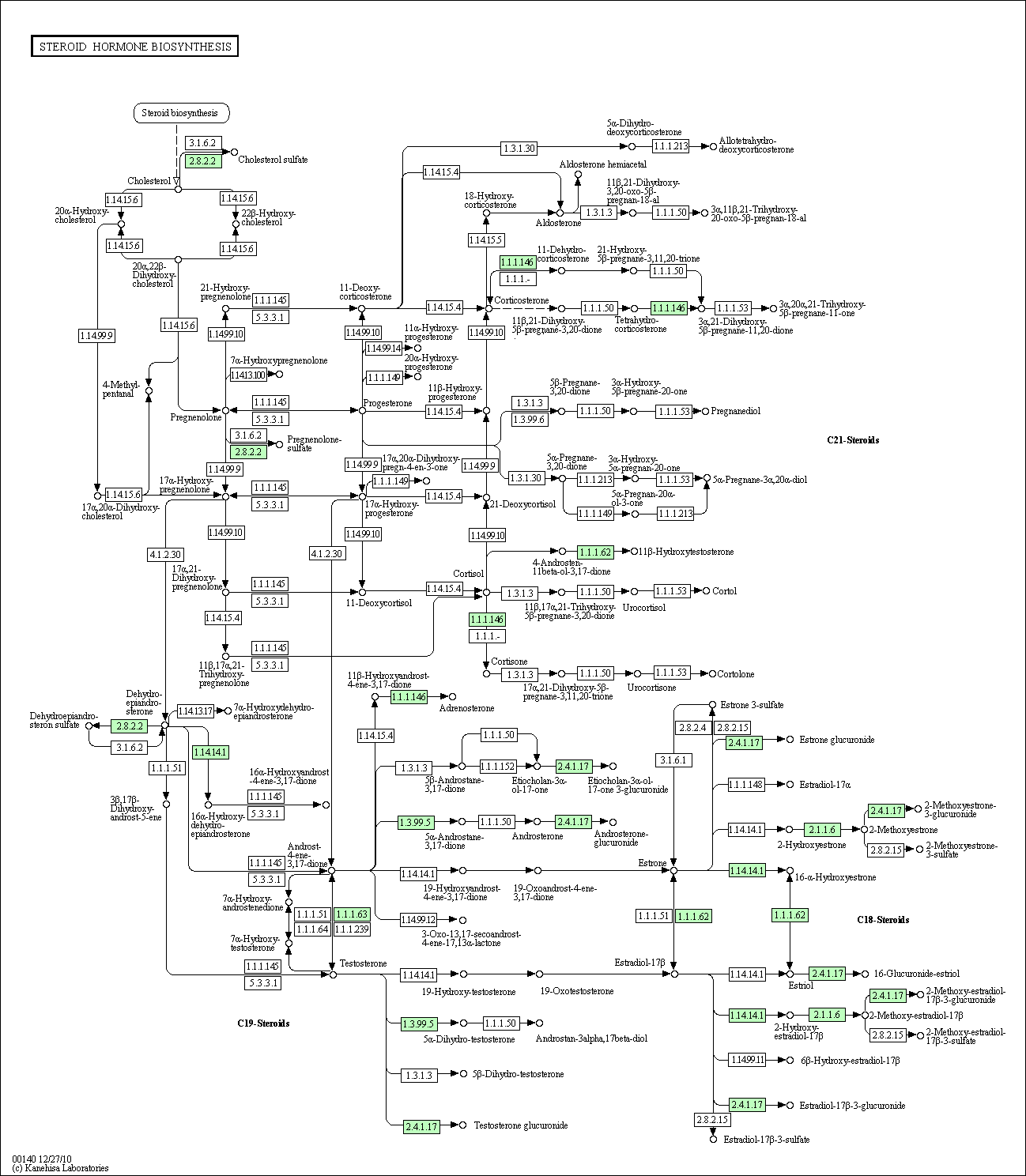

Supplement: Additional file 6 — KEGG annotation results. File contains the KEGG maps where isotigs were successfully mapped. Pathway element in green boxes indicates the components represented by at least, one isotig. Gilthead sea bream isotigs were automatically annotated to KEGG maps using KAAS website [60]. The SBH method, optimized for ESTs annotation, was used against human, chimpanzee, orang-utan, rhesus, mouse, rat, dog, giant panda, cow, pig, horse, opossum, platypus, chicken, clawed frog, zebrafish, fruit fly and nematode pathway databases. [file 1471-2164-13-181-S6.tar › map/map00140.png]

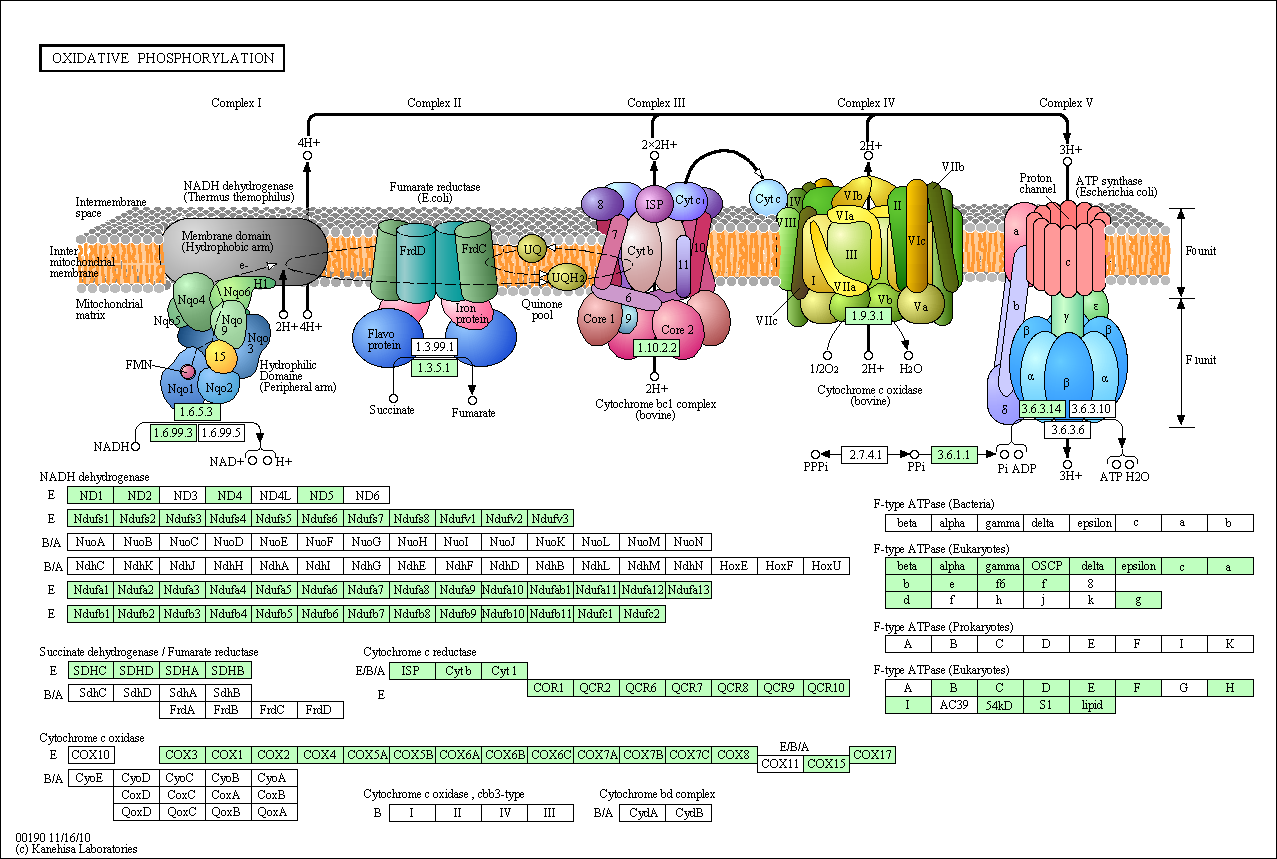

Supplement: Additional file 6 — KEGG annotation results. File contains the KEGG maps where isotigs were successfully mapped. Pathway element in green boxes indicates the components represented by at least, one isotig. Gilthead sea bream isotigs were automatically annotated to KEGG maps using KAAS website [60]. The SBH method, optimized for ESTs annotation, was used against human, chimpanzee, orang-utan, rhesus, mouse, rat, dog, giant panda, cow, pig, horse, opossum, platypus, chicken, clawed frog, zebrafish, fruit fly and nematode pathway databases. [file 1471-2164-13-181-S6.tar › map/map00190.png]

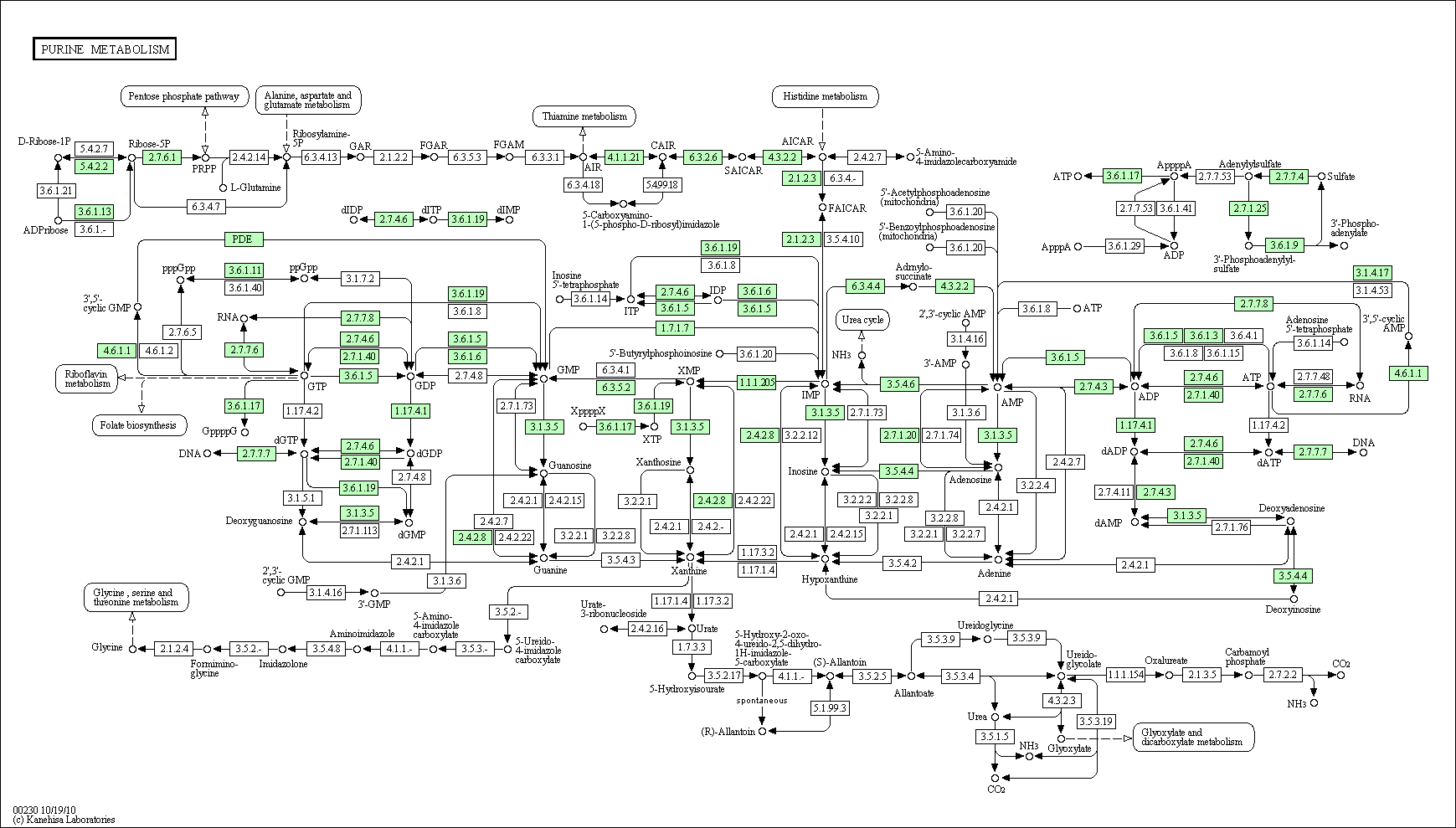

Supplement: Additional file 6 — KEGG annotation results. File contains the KEGG maps where isotigs were successfully mapped. Pathway element in green boxes indicates the components represented by at least, one isotig. Gilthead sea bream isotigs were automatically annotated to KEGG maps using KAAS website [60]. The SBH method, optimized for ESTs annotation, was used against human, chimpanzee, orang-utan, rhesus, mouse, rat, dog, giant panda, cow, pig, horse, opossum, platypus, chicken, clawed frog, zebrafish, fruit fly and nematode pathway databases. [file 1471-2164-13-181-S6.tar › map/map00230.png]

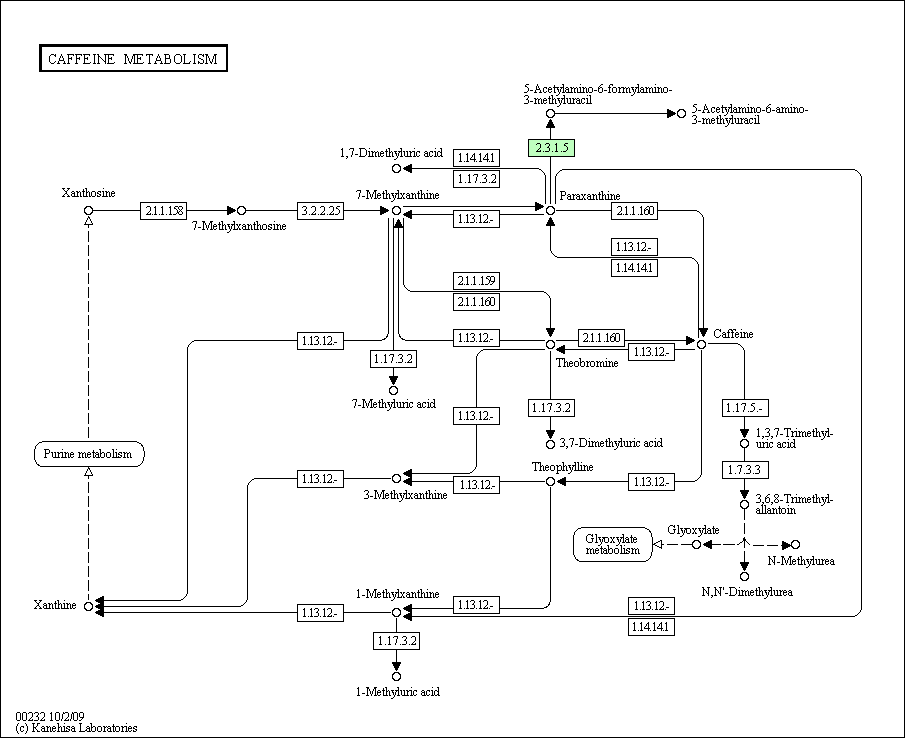

Supplement: Additional file 6 — KEGG annotation results. File contains the KEGG maps where isotigs were successfully mapped. Pathway element in green boxes indicates the components represented by at least, one isotig. Gilthead sea bream isotigs were automatically annotated to KEGG maps using KAAS website [60]. The SBH method, optimized for ESTs annotation, was used against human, chimpanzee, orang-utan, rhesus, mouse, rat, dog, giant panda, cow, pig, horse, opossum, platypus, chicken, clawed frog, zebrafish, fruit fly and nematode pathway databases. [file 1471-2164-13-181-S6.tar › map/map00232.png]

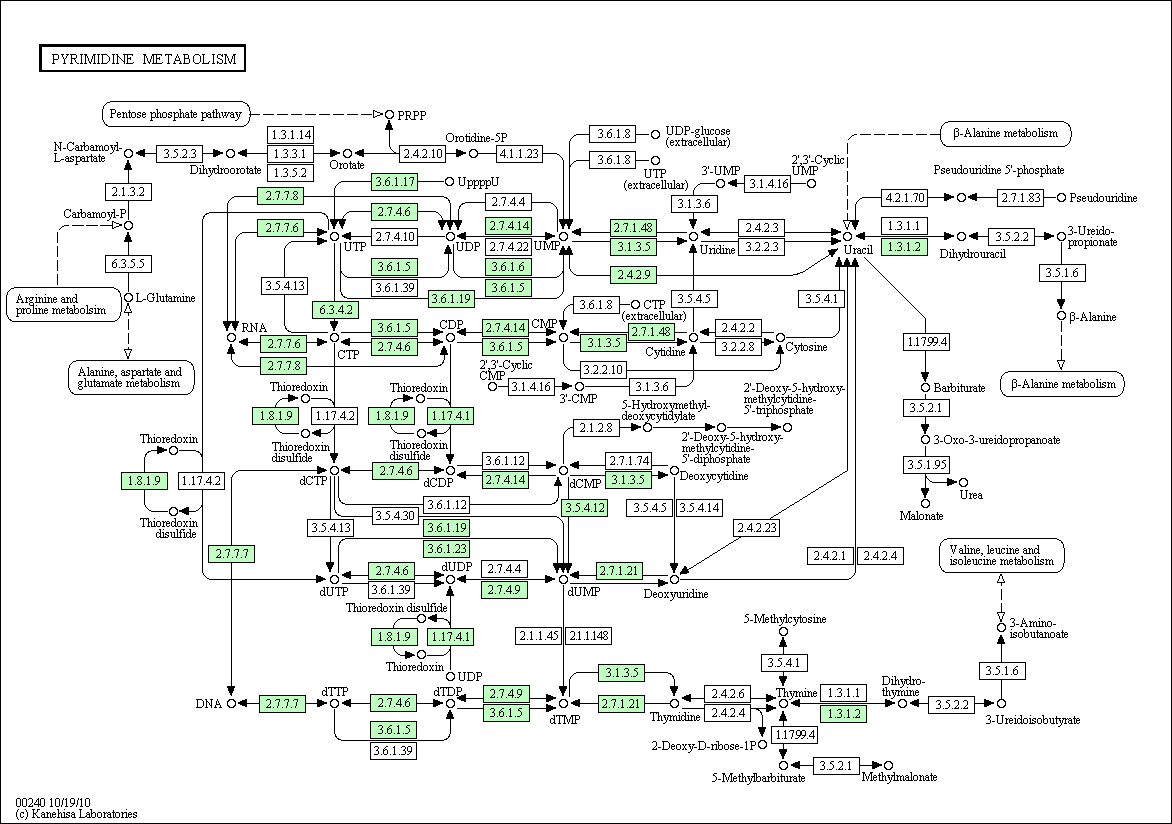

Supplement: Additional file 6 — KEGG annotation results. File contains the KEGG maps where isotigs were successfully mapped. Pathway element in green boxes indicates the components represented by at least, one isotig. Gilthead sea bream isotigs were automatically annotated to KEGG maps using KAAS website [60]. The SBH method, optimized for ESTs annotation, was used against human, chimpanzee, orang-utan, rhesus, mouse, rat, dog, giant panda, cow, pig, horse, opossum, platypus, chicken, clawed frog, zebrafish, fruit fly and nematode pathway databases. [file 1471-2164-13-181-S6.tar › map/map00240.png]

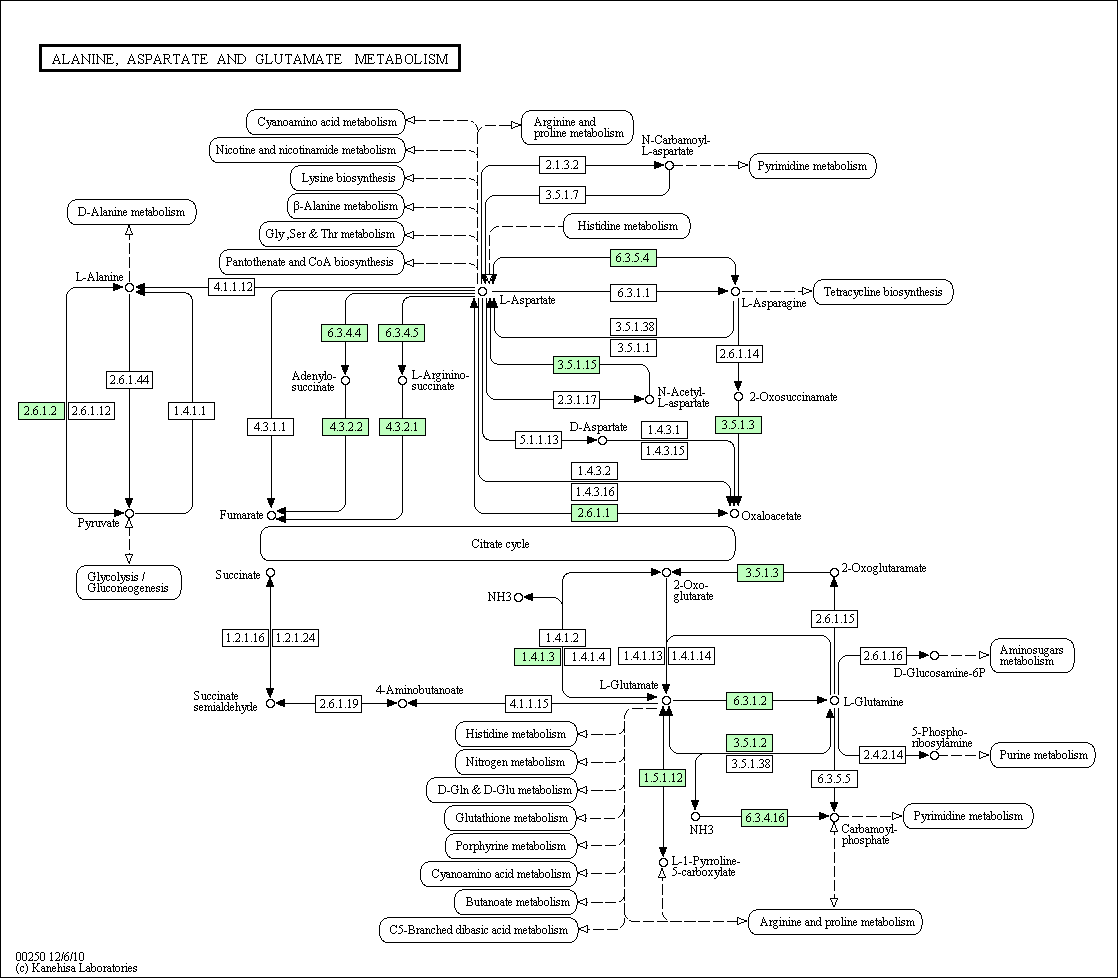

Supplement: Additional file 6 — KEGG annotation results. File contains the KEGG maps where isotigs were successfully mapped. Pathway element in green boxes indicates the components represented by at least, one isotig. Gilthead sea bream isotigs were automatically annotated to KEGG maps using KAAS website [60]. The SBH method, optimized for ESTs annotation, was used against human, chimpanzee, orang-utan, rhesus, mouse, rat, dog, giant panda, cow, pig, horse, opossum, platypus, chicken, clawed frog, zebrafish, fruit fly and nematode pathway databases. [file 1471-2164-13-181-S6.tar › map/map00250.png]

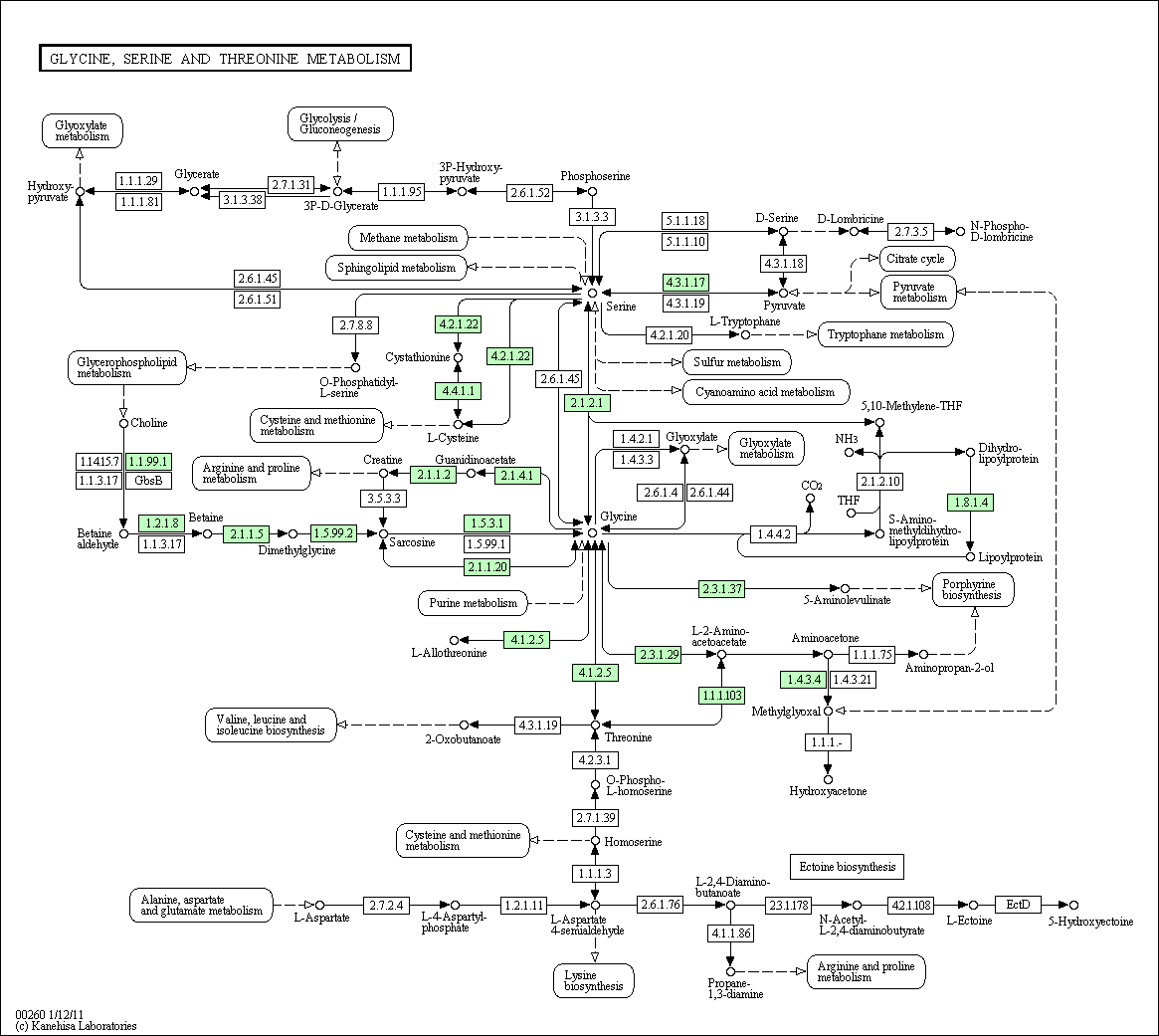

Supplement: Additional file 6 — KEGG annotation results. File contains the KEGG maps where isotigs were successfully mapped. Pathway element in green boxes indicates the components represented by at least, one isotig. Gilthead sea bream isotigs were automatically annotated to KEGG maps using KAAS website [60]. The SBH method, optimized for ESTs annotation, was used against human, chimpanzee, orang-utan, rhesus, mouse, rat, dog, giant panda, cow, pig, horse, opossum, platypus, chicken, clawed frog, zebrafish, fruit fly and nematode pathway databases. [file 1471-2164-13-181-S6.tar › map/map00260.png]

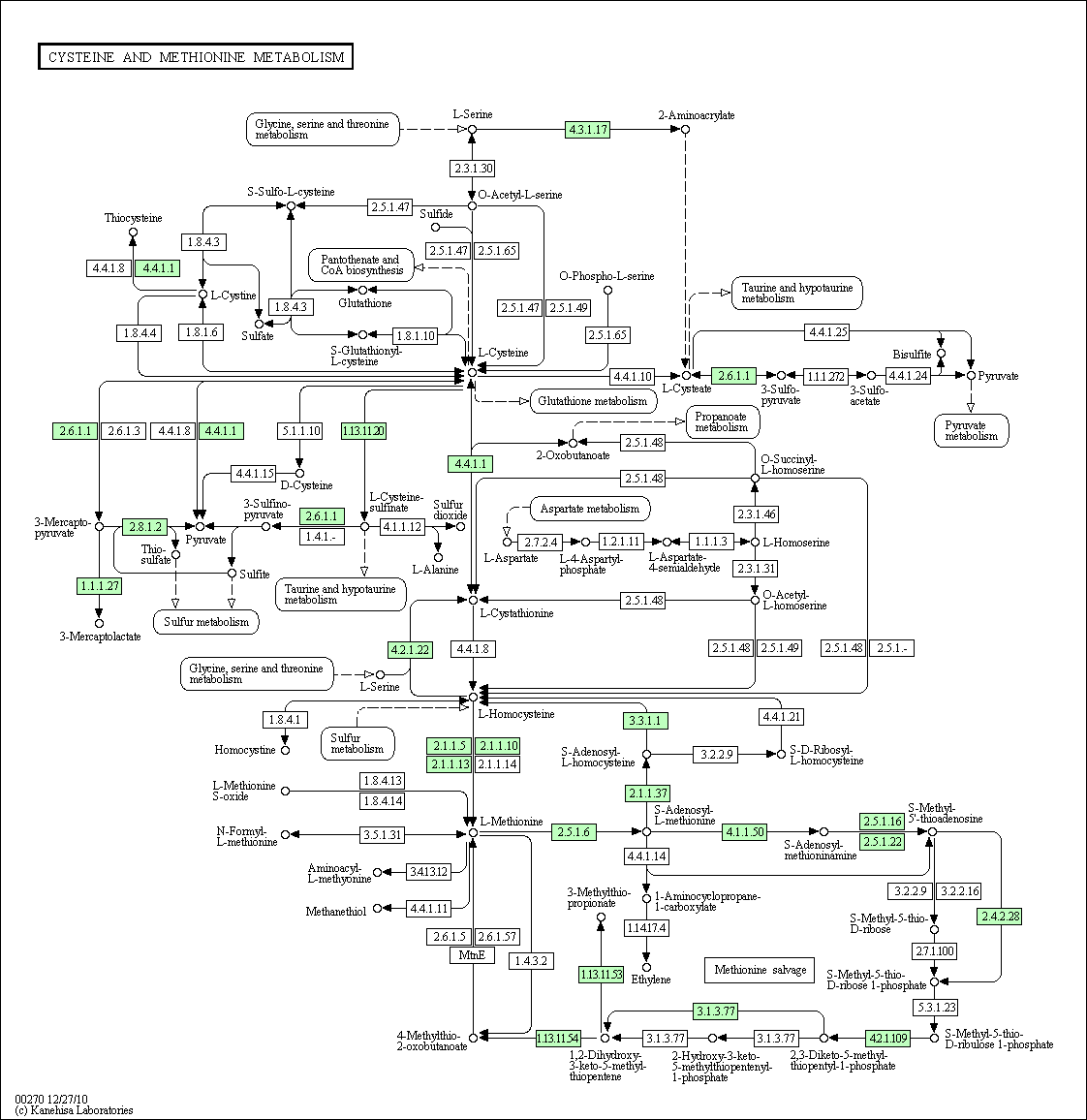

Supplement: Additional file 6 — KEGG annotation results. File contains the KEGG maps where isotigs were successfully mapped. Pathway element in green boxes indicates the components represented by at least, one isotig. Gilthead sea bream isotigs were automatically annotated to KEGG maps using KAAS website [60]. The SBH method, optimized for ESTs annotation, was used against human, chimpanzee, orang-utan, rhesus, mouse, rat, dog, giant panda, cow, pig, horse, opossum, platypus, chicken, clawed frog, zebrafish, fruit fly and nematode pathway databases. [file 1471-2164-13-181-S6.tar › map/map00270.png]

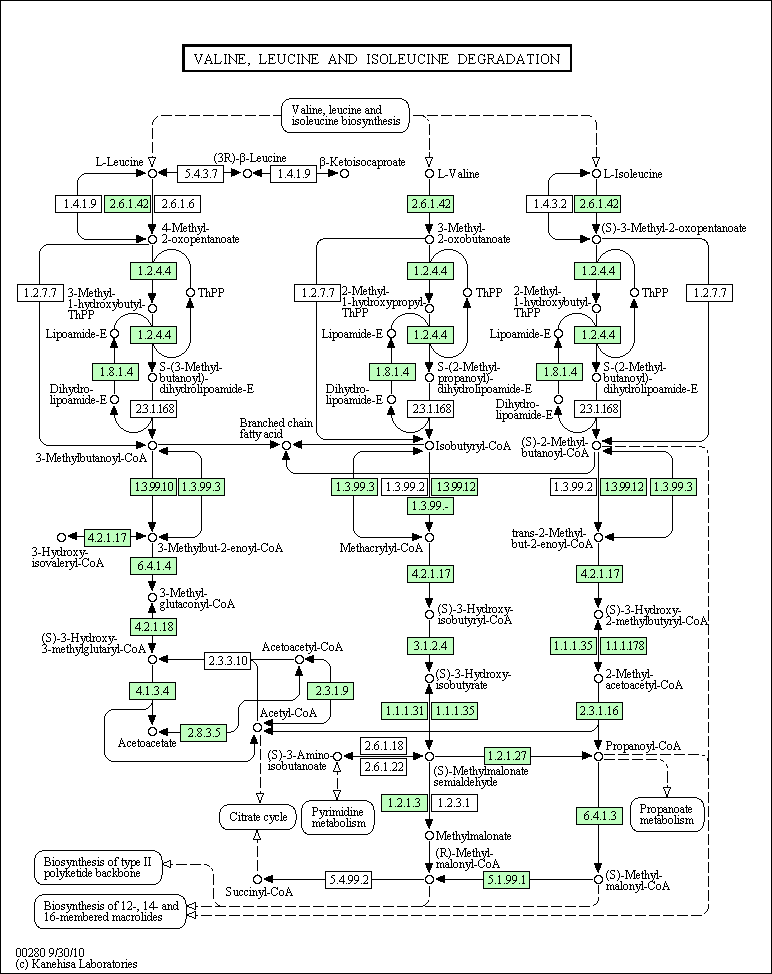

Supplement: Additional file 6 — KEGG annotation results. File contains the KEGG maps where isotigs were successfully mapped. Pathway element in green boxes indicates the components represented by at least, one isotig. Gilthead sea bream isotigs were automatically annotated to KEGG maps using KAAS website [60]. The SBH method, optimized for ESTs annotation, was used against human, chimpanzee, orang-utan, rhesus, mouse, rat, dog, giant panda, cow, pig, horse, opossum, platypus, chicken, clawed frog, zebrafish, fruit fly and nematode pathway databases. [file 1471-2164-13-181-S6.tar › map/map00280.png]

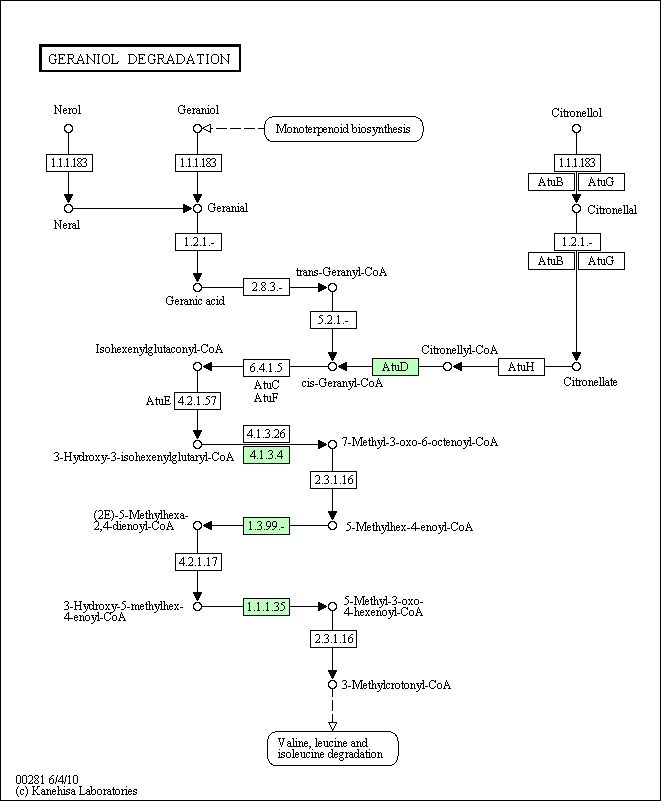

Supplement: Additional file 6 — KEGG annotation results. File contains the KEGG maps where isotigs were successfully mapped. Pathway element in green boxes indicates the components represented by at least, one isotig. Gilthead sea bream isotigs were automatically annotated to KEGG maps using KAAS website [60]. The SBH method, optimized for ESTs annotation, was used against human, chimpanzee, orang-utan, rhesus, mouse, rat, dog, giant panda, cow, pig, horse, opossum, platypus, chicken, clawed frog, zebrafish, fruit fly and nematode pathway databases. [file 1471-2164-13-181-S6.tar › map/map00281.png]

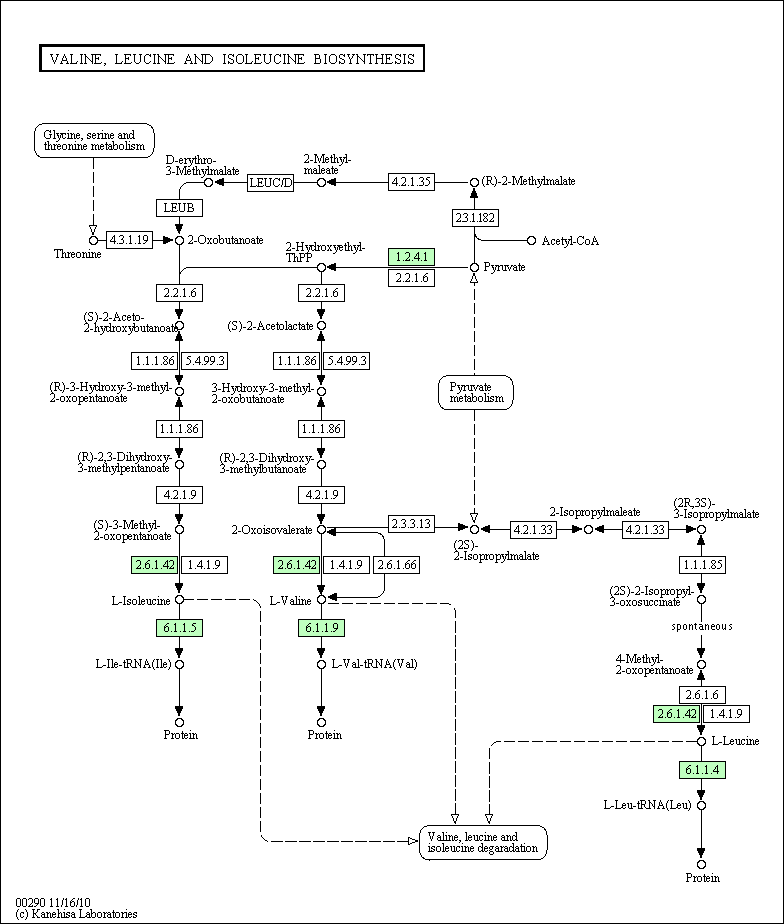

Supplement: Additional file 6 — KEGG annotation results. File contains the KEGG maps where isotigs were successfully mapped. Pathway element in green boxes indicates the components represented by at least, one isotig. Gilthead sea bream isotigs were automatically annotated to KEGG maps using KAAS website [60]. The SBH method, optimized for ESTs annotation, was used against human, chimpanzee, orang-utan, rhesus, mouse, rat, dog, giant panda, cow, pig, horse, opossum, platypus, chicken, clawed frog, zebrafish, fruit fly and nematode pathway databases. [file 1471-2164-13-181-S6.tar › map/map00290.png]

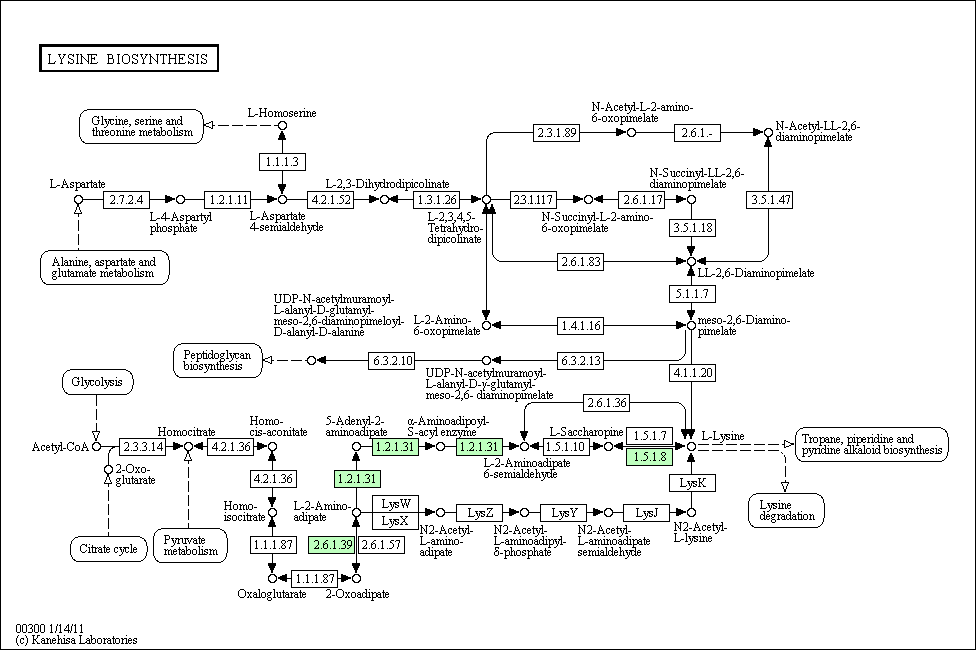

Supplement: Additional file 6 — KEGG annotation results. File contains the KEGG maps where isotigs were successfully mapped. Pathway element in green boxes indicates the components represented by at least, one isotig. Gilthead sea bream isotigs were automatically annotated to KEGG maps using KAAS website [60]. The SBH method, optimized for ESTs annotation, was used against human, chimpanzee, orang-utan, rhesus, mouse, rat, dog, giant panda, cow, pig, horse, opossum, platypus, chicken, clawed frog, zebrafish, fruit fly and nematode pathway databases. [file 1471-2164-13-181-S6.tar › map/map00300.png]

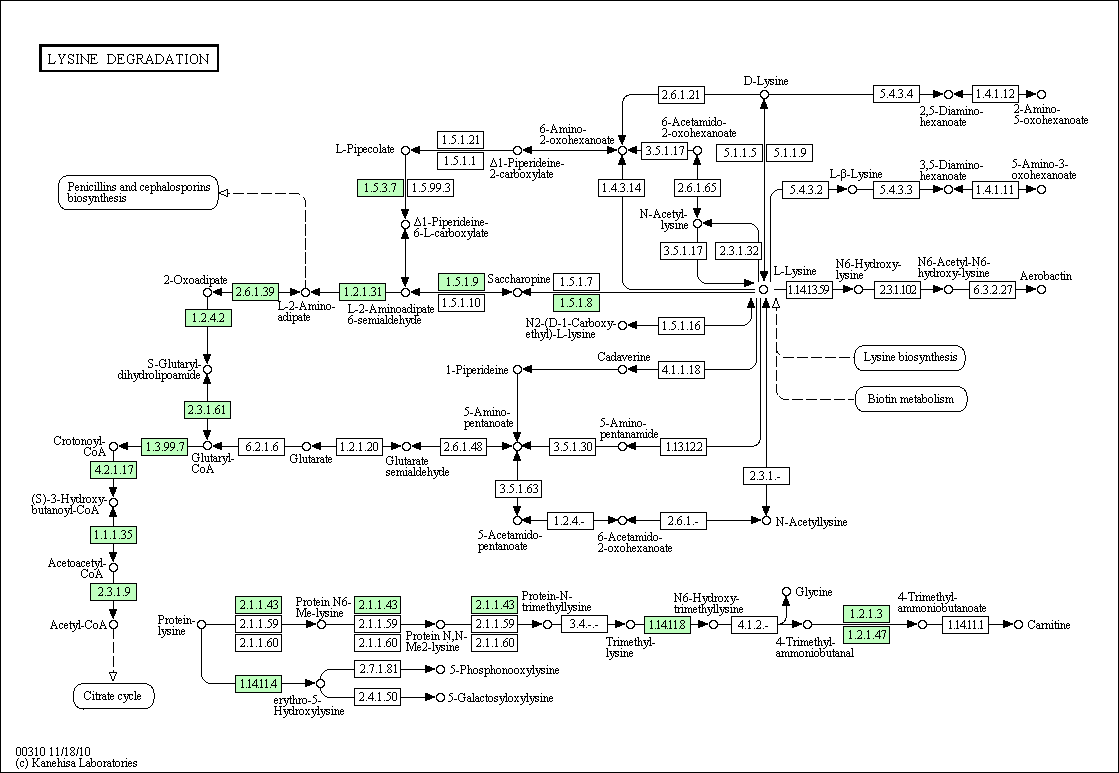

Supplement: Additional file 6 — KEGG annotation results. File contains the KEGG maps where isotigs were successfully mapped. Pathway element in green boxes indicates the components represented by at least, one isotig. Gilthead sea bream isotigs were automatically annotated to KEGG maps using KAAS website [60]. The SBH method, optimized for ESTs annotation, was used against human, chimpanzee, orang-utan, rhesus, mouse, rat, dog, giant panda, cow, pig, horse, opossum, platypus, chicken, clawed frog, zebrafish, fruit fly and nematode pathway databases. [file 1471-2164-13-181-S6.tar › map/map00310.png]

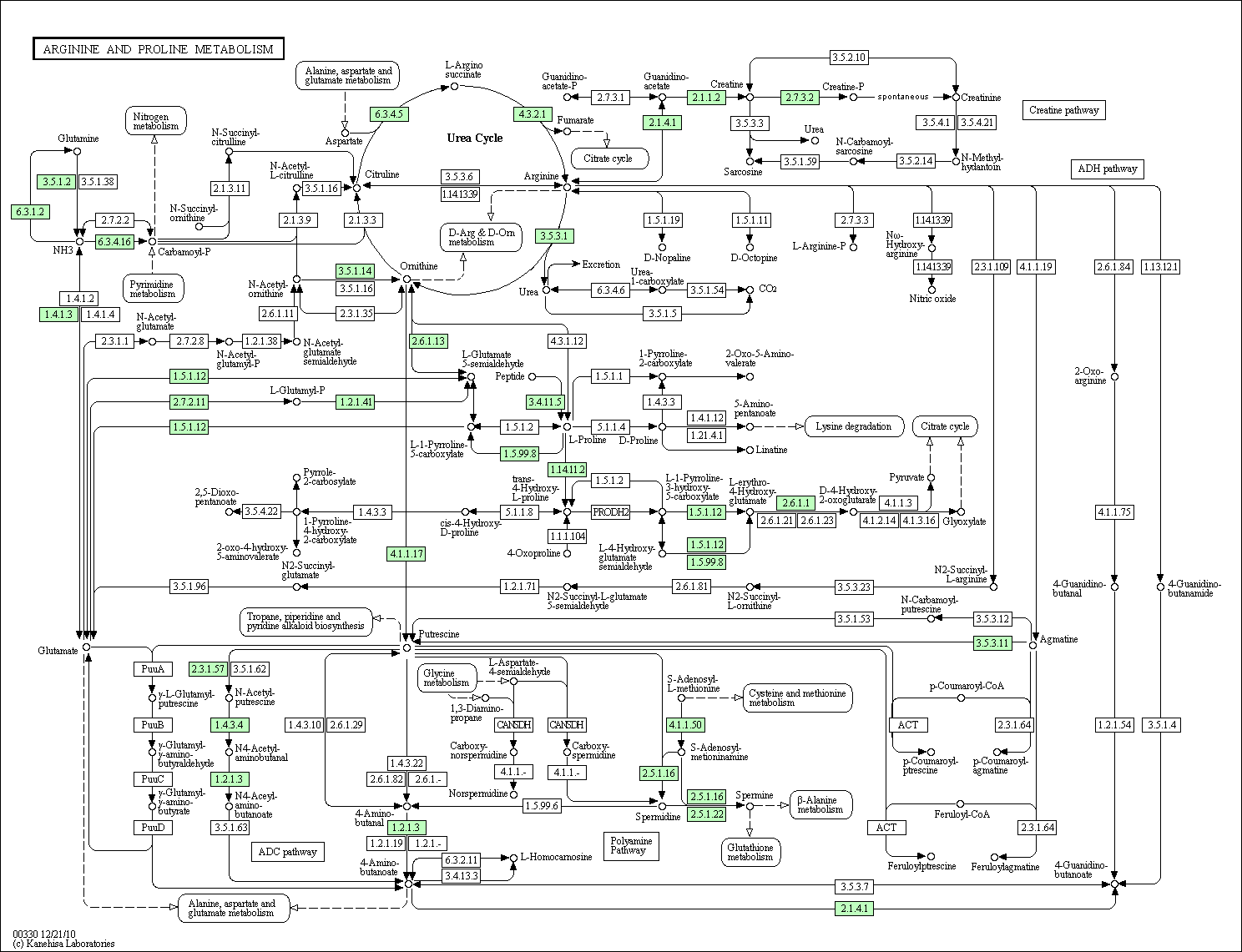

Supplement: Additional file 6 — KEGG annotation results. File contains the KEGG maps where isotigs were successfully mapped. Pathway element in green boxes indicates the components represented by at least, one isotig. Gilthead sea bream isotigs were automatically annotated to KEGG maps using KAAS website [60]. The SBH method, optimized for ESTs annotation, was used against human, chimpanzee, orang-utan, rhesus, mouse, rat, dog, giant panda, cow, pig, horse, opossum, platypus, chicken, clawed frog, zebrafish, fruit fly and nematode pathway databases. [file 1471-2164-13-181-S6.tar › map/map00330.png]

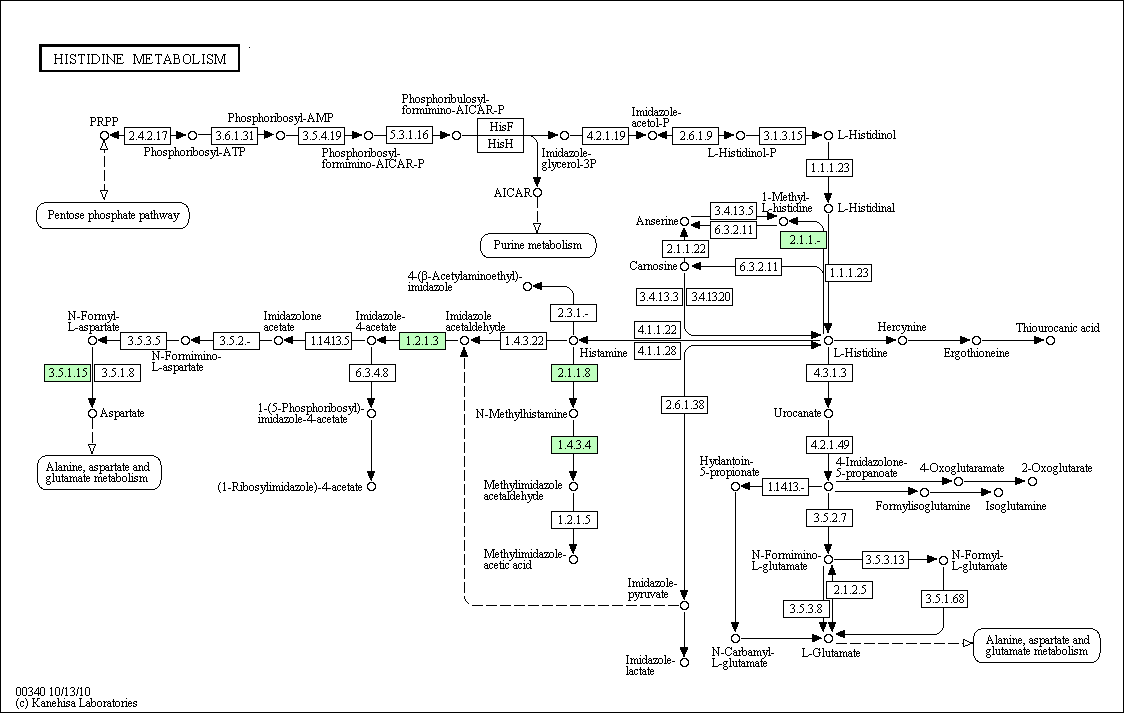

Supplement: Additional file 6 — KEGG annotation results. File contains the KEGG maps where isotigs were successfully mapped. Pathway element in green boxes indicates the components represented by at least, one isotig. Gilthead sea bream isotigs were automatically annotated to KEGG maps using KAAS website [60]. The SBH method, optimized for ESTs annotation, was used against human, chimpanzee, orang-utan, rhesus, mouse, rat, dog, giant panda, cow, pig, horse, opossum, platypus, chicken, clawed frog, zebrafish, fruit fly and nematode pathway databases. [file 1471-2164-13-181-S6.tar › map/map00340.png]

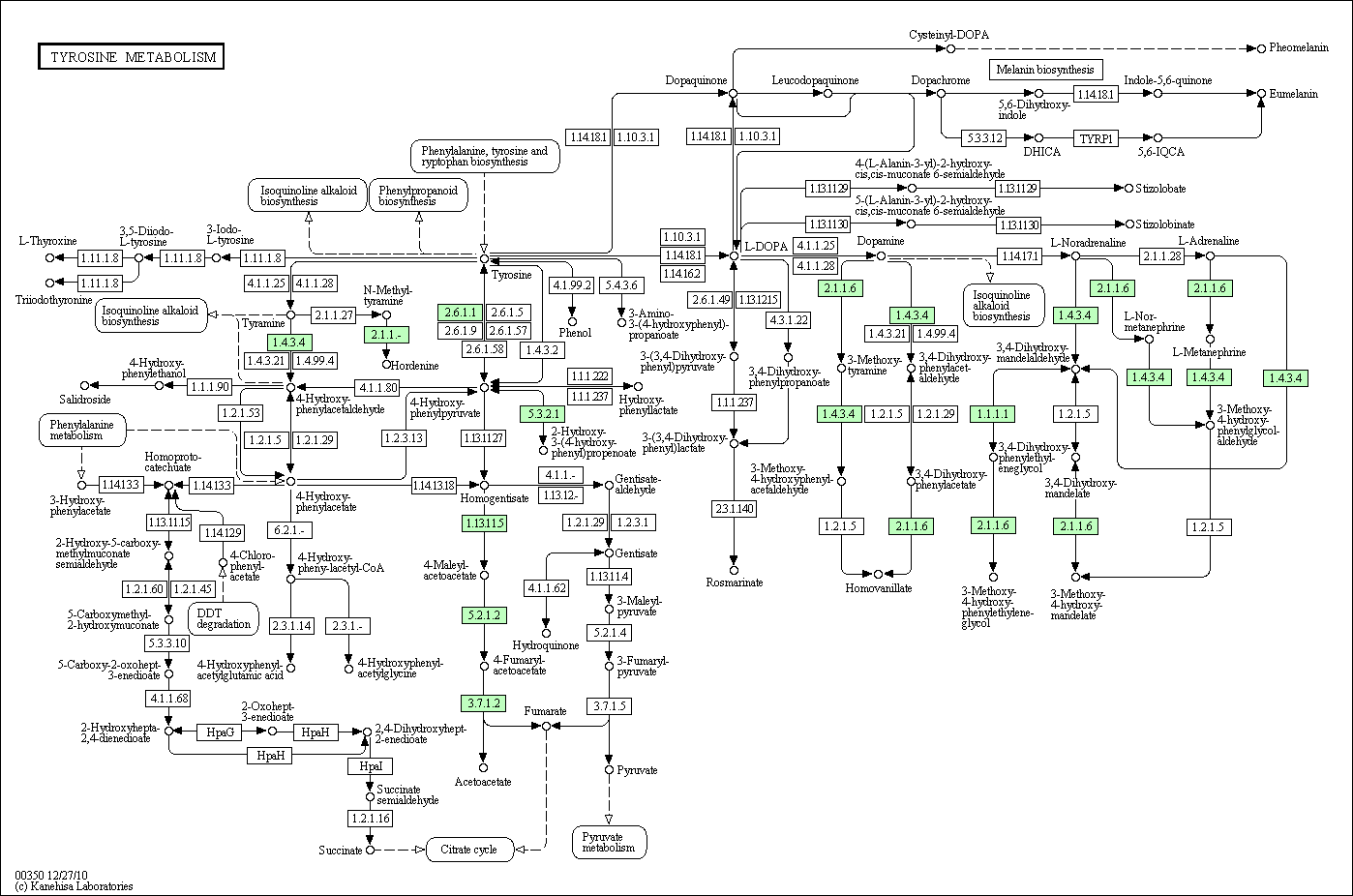

Supplement: Additional file 6 — KEGG annotation results. File contains the KEGG maps where isotigs were successfully mapped. Pathway element in green boxes indicates the components represented by at least, one isotig. Gilthead sea bream isotigs were automatically annotated to KEGG maps using KAAS website [60]. The SBH method, optimized for ESTs annotation, was used against human, chimpanzee, orang-utan, rhesus, mouse, rat, dog, giant panda, cow, pig, horse, opossum, platypus, chicken, clawed frog, zebrafish, fruit fly and nematode pathway databases. [file 1471-2164-13-181-S6.tar › map/map00350.png]

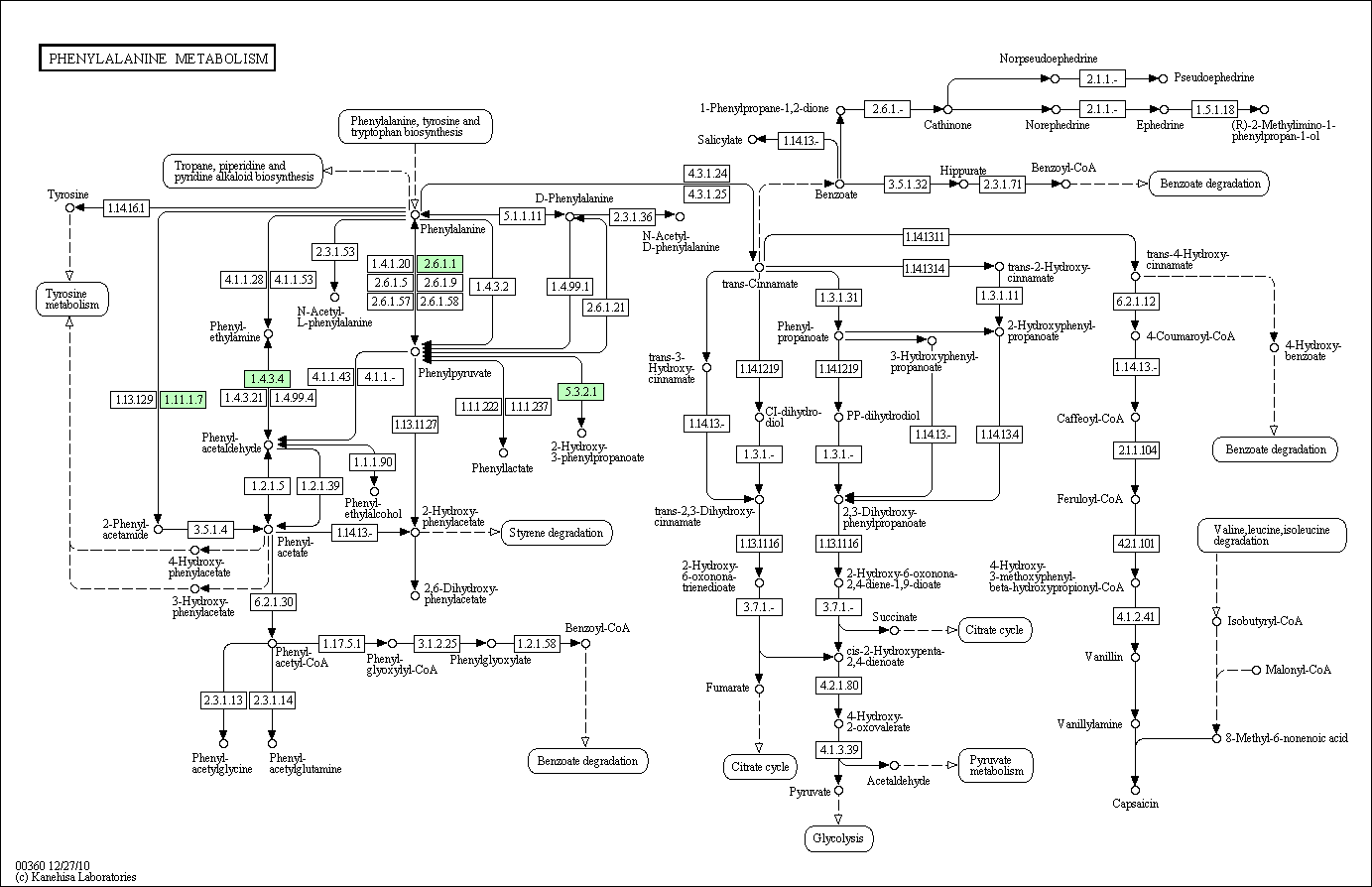

Supplement: Additional file 6 — KEGG annotation results. File contains the KEGG maps where isotigs were successfully mapped. Pathway element in green boxes indicates the components represented by at least, one isotig. Gilthead sea bream isotigs were automatically annotated to KEGG maps using KAAS website [60]. The SBH method, optimized for ESTs annotation, was used against human, chimpanzee, orang-utan, rhesus, mouse, rat, dog, giant panda, cow, pig, horse, opossum, platypus, chicken, clawed frog, zebrafish, fruit fly and nematode pathway databases. [file 1471-2164-13-181-S6.tar › map/map00360.png]

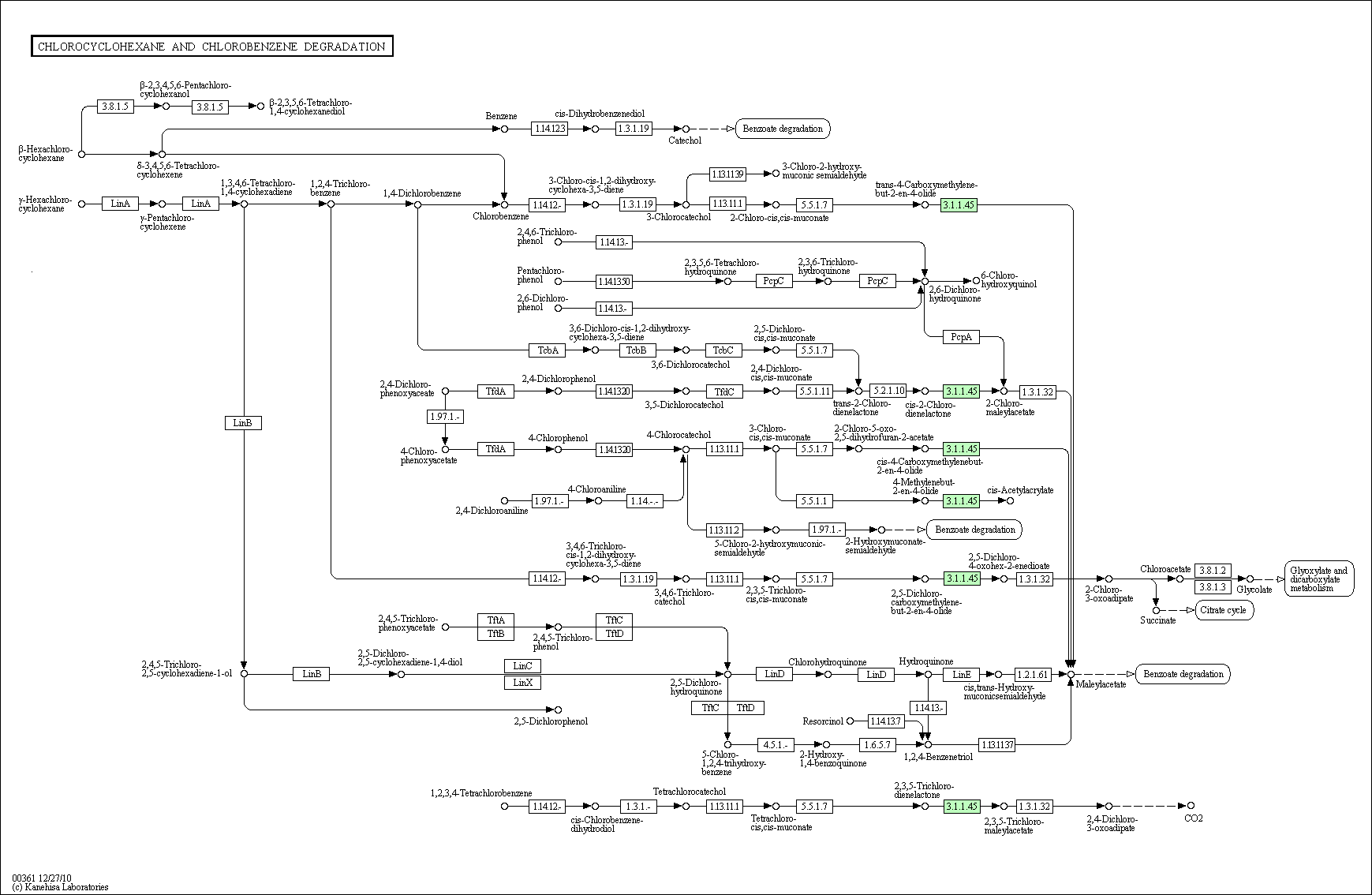

Supplement: Additional file 6 — KEGG annotation results. File contains the KEGG maps where isotigs were successfully mapped. Pathway element in green boxes indicates the components represented by at least, one isotig. Gilthead sea bream isotigs were automatically annotated to KEGG maps using KAAS website [60]. The SBH method, optimized for ESTs annotation, was used against human, chimpanzee, orang-utan, rhesus, mouse, rat, dog, giant panda, cow, pig, horse, opossum, platypus, chicken, clawed frog, zebrafish, fruit fly and nematode pathway databases. [file 1471-2164-13-181-S6.tar › map/map00361.png]

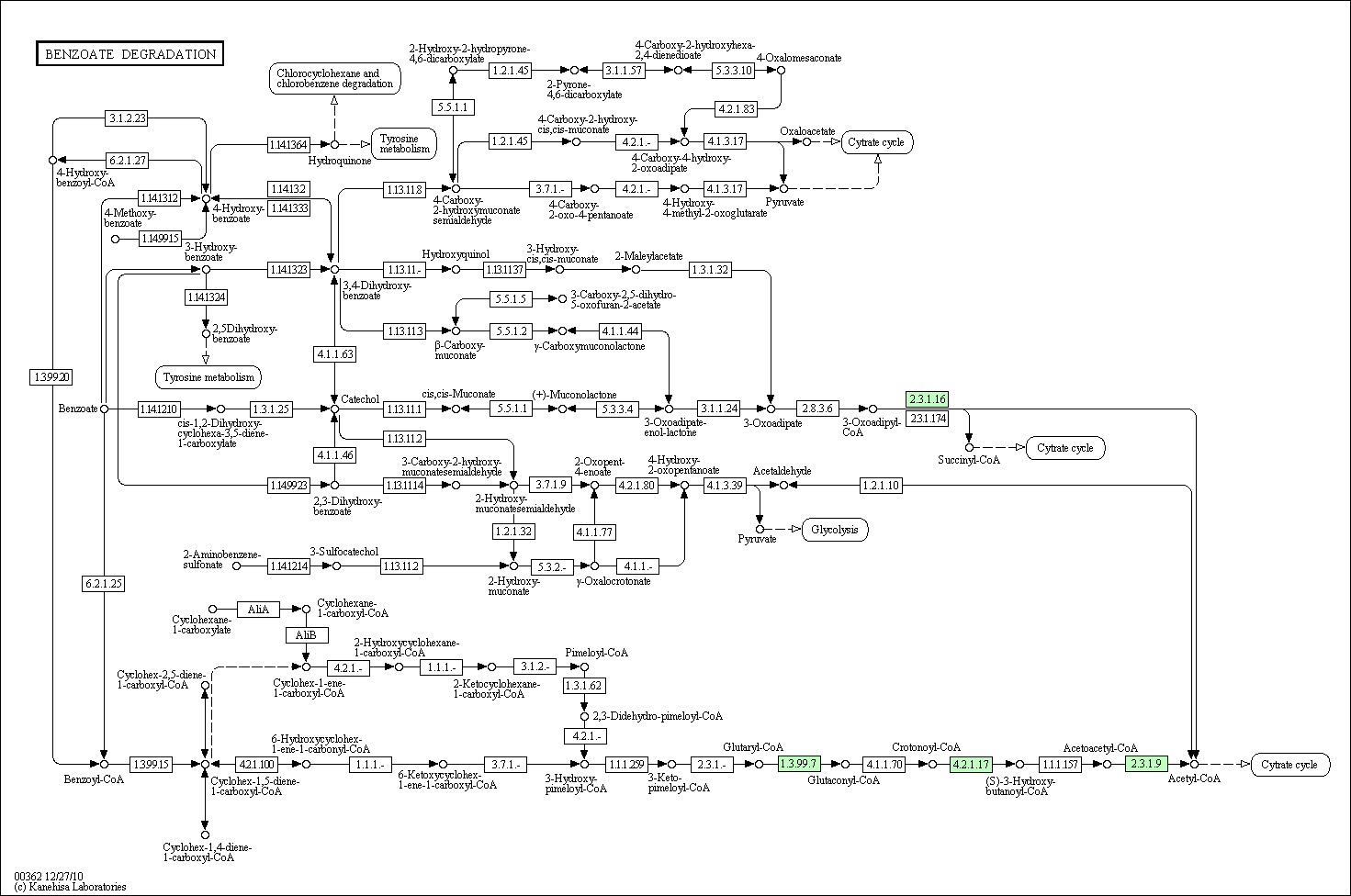

Supplement: Additional file 6 — KEGG annotation results. File contains the KEGG maps where isotigs were successfully mapped. Pathway element in green boxes indicates the components represented by at least, one isotig. Gilthead sea bream isotigs were automatically annotated to KEGG maps using KAAS website [60]. The SBH method, optimized for ESTs annotation, was used against human, chimpanzee, orang-utan, rhesus, mouse, rat, dog, giant panda, cow, pig, horse, opossum, platypus, chicken, clawed frog, zebrafish, fruit fly and nematode pathway databases. [file 1471-2164-13-181-S6.tar › map/map00362.png]

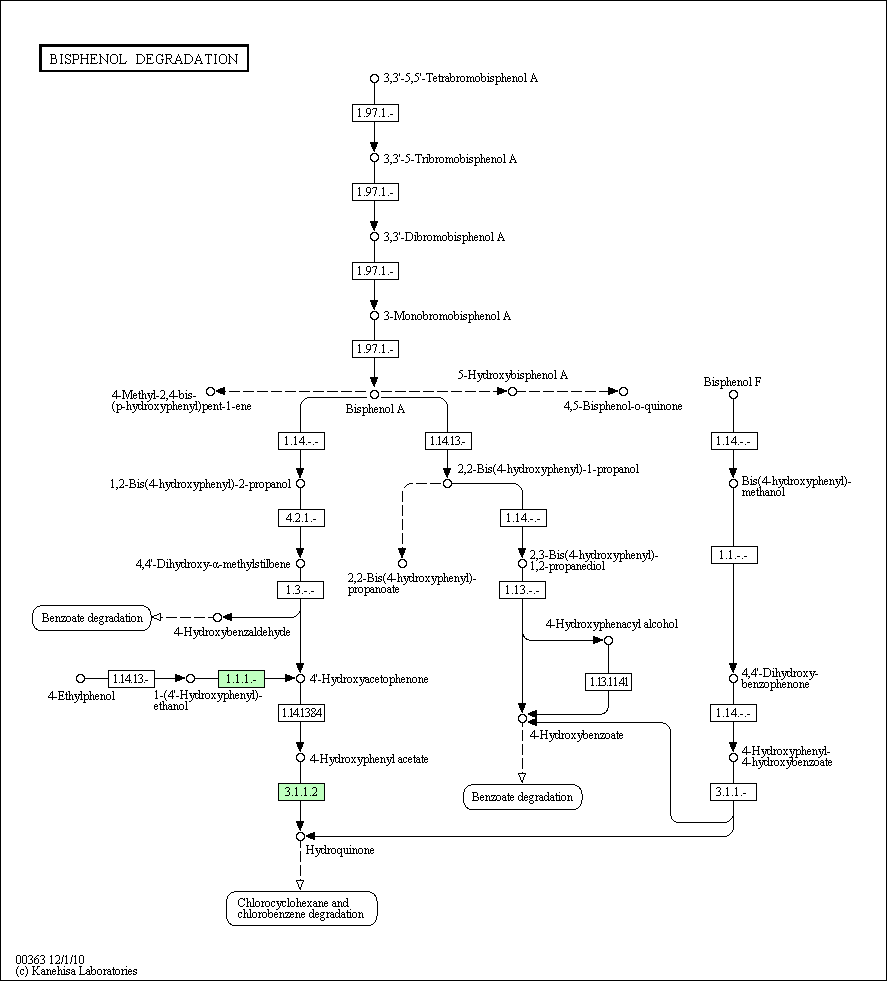

Supplement: Additional file 6 — KEGG annotation results. File contains the KEGG maps where isotigs were successfully mapped. Pathway element in green boxes indicates the components represented by at least, one isotig. Gilthead sea bream isotigs were automatically annotated to KEGG maps using KAAS website [60]. The SBH method, optimized for ESTs annotation, was used against human, chimpanzee, orang-utan, rhesus, mouse, rat, dog, giant panda, cow, pig, horse, opossum, platypus, chicken, clawed frog, zebrafish, fruit fly and nematode pathway databases. [file 1471-2164-13-181-S6.tar › map/map00363.png]

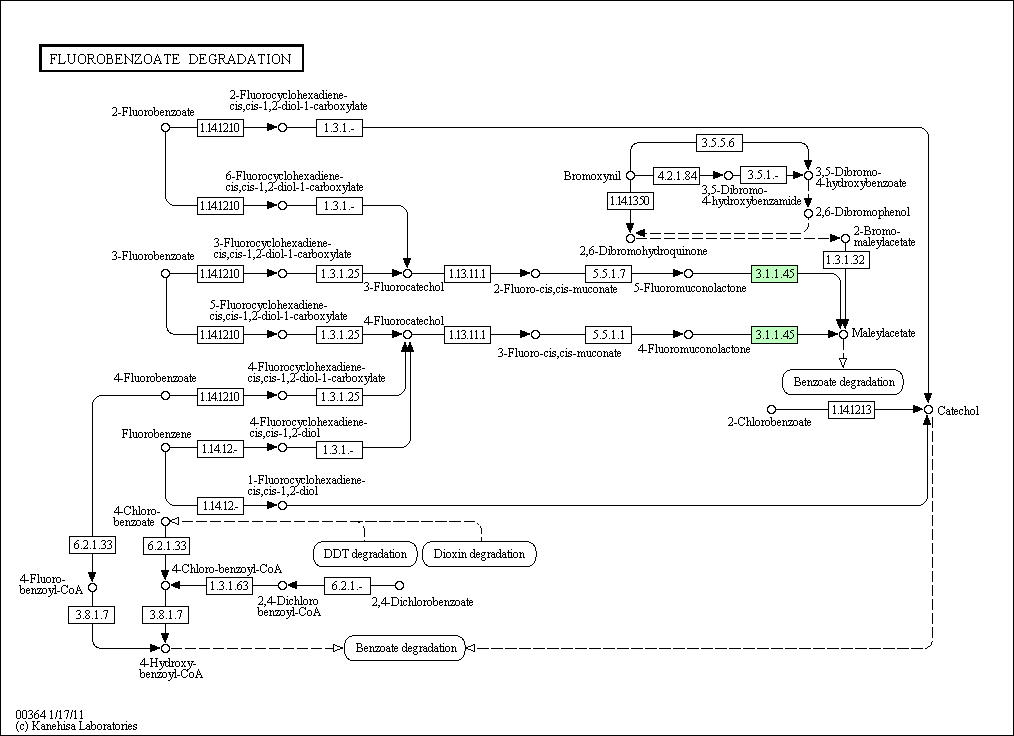

Supplement: Additional file 6 — KEGG annotation results. File contains the KEGG maps where isotigs were successfully mapped. Pathway element in green boxes indicates the components represented by at least, one isotig. Gilthead sea bream isotigs were automatically annotated to KEGG maps using KAAS website [60]. The SBH method, optimized for ESTs annotation, was used against human, chimpanzee, orang-utan, rhesus, mouse, rat, dog, giant panda, cow, pig, horse, opossum, platypus, chicken, clawed frog, zebrafish, fruit fly and nematode pathway databases. [file 1471-2164-13-181-S6.tar › map/map00364.png]

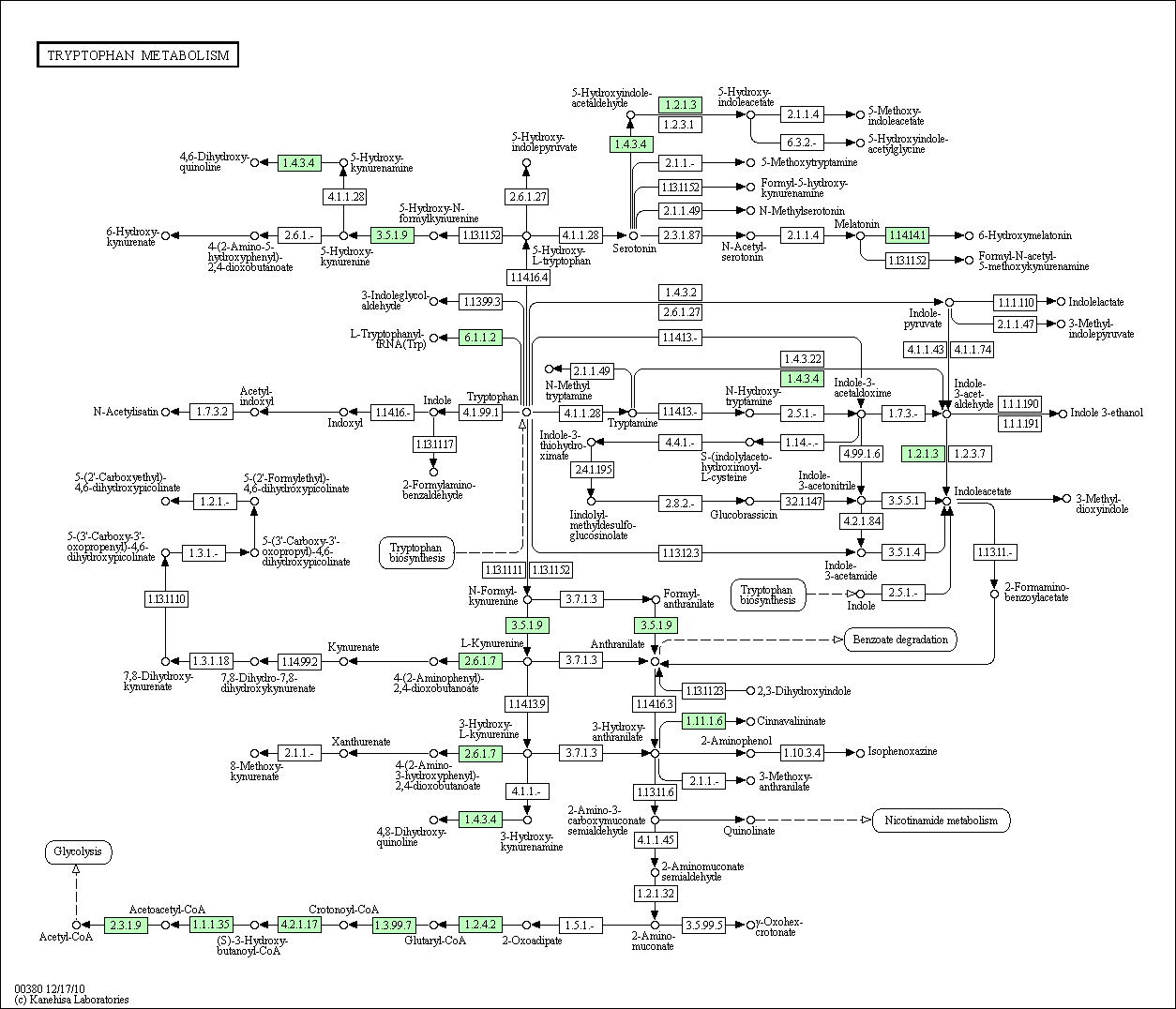

Supplement: Additional file 6 — KEGG annotation results. File contains the KEGG maps where isotigs were successfully mapped. Pathway element in green boxes indicates the components represented by at least, one isotig. Gilthead sea bream isotigs were automatically annotated to KEGG maps using KAAS website [60]. The SBH method, optimized for ESTs annotation, was used against human, chimpanzee, orang-utan, rhesus, mouse, rat, dog, giant panda, cow, pig, horse, opossum, platypus, chicken, clawed frog, zebrafish, fruit fly and nematode pathway databases. [file 1471-2164-13-181-S6.tar › map/map00380.png]

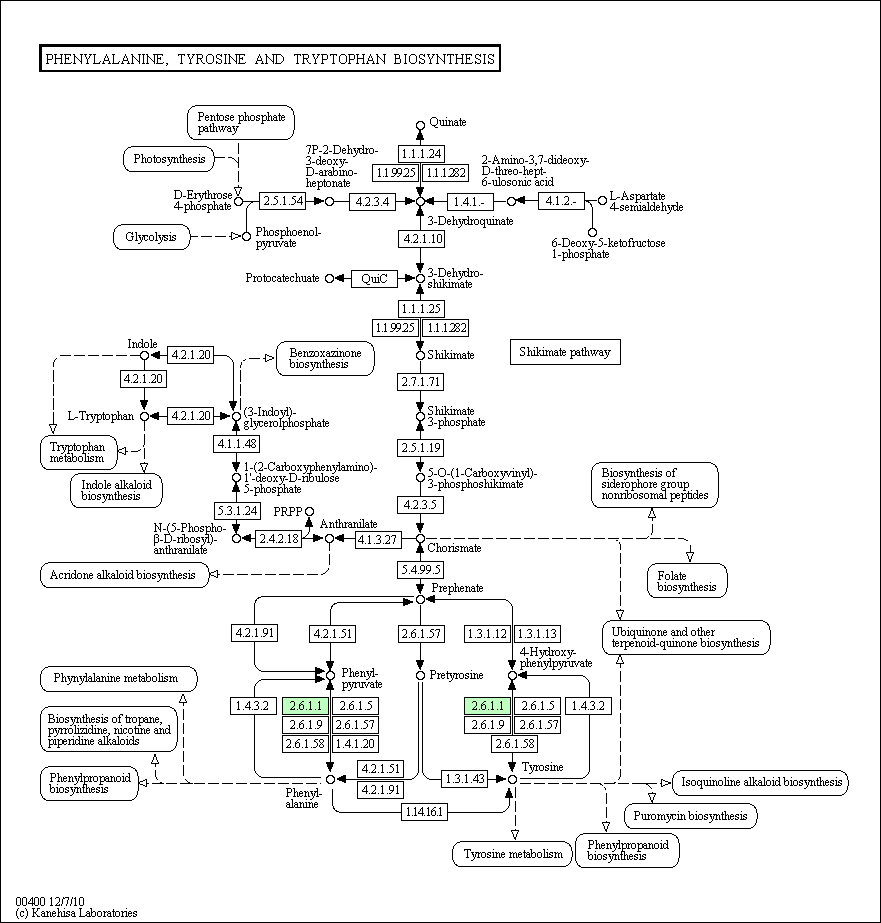

Supplement: Additional file 6 — KEGG annotation results. File contains the KEGG maps where isotigs were successfully mapped. Pathway element in green boxes indicates the components represented by at least, one isotig. Gilthead sea bream isotigs were automatically annotated to KEGG maps using KAAS website [60]. The SBH method, optimized for ESTs annotation, was used against human, chimpanzee, orang-utan, rhesus, mouse, rat, dog, giant panda, cow, pig, horse, opossum, platypus, chicken, clawed frog, zebrafish, fruit fly and nematode pathway databases. [file 1471-2164-13-181-S6.tar › map/map00400.png]

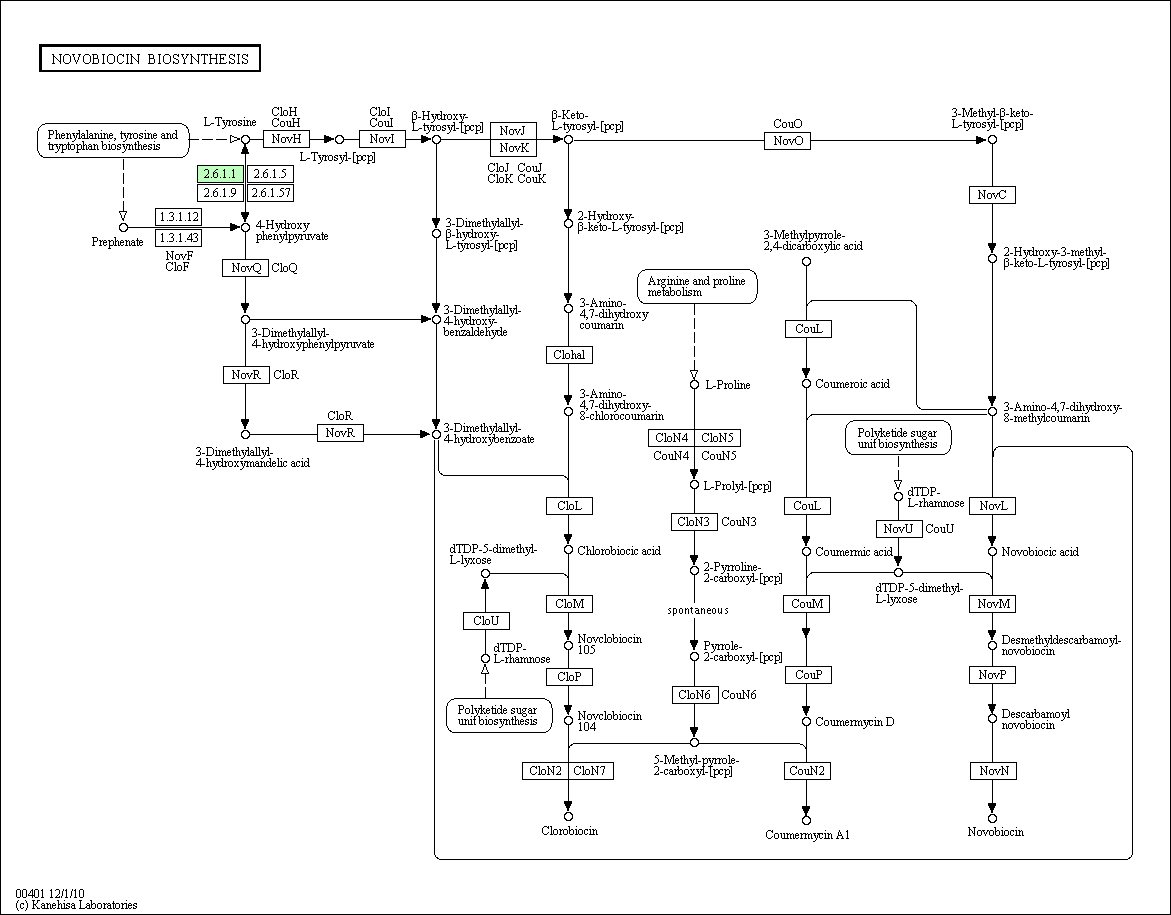

Supplement: Additional file 6 — KEGG annotation results. File contains the KEGG maps where isotigs were successfully mapped. Pathway element in green boxes indicates the components represented by at least, one isotig. Gilthead sea bream isotigs were automatically annotated to KEGG maps using KAAS website [60]. The SBH method, optimized for ESTs annotation, was used against human, chimpanzee, orang-utan, rhesus, mouse, rat, dog, giant panda, cow, pig, horse, opossum, platypus, chicken, clawed frog, zebrafish, fruit fly and nematode pathway databases. [file 1471-2164-13-181-S6.tar › map/map00401.png]

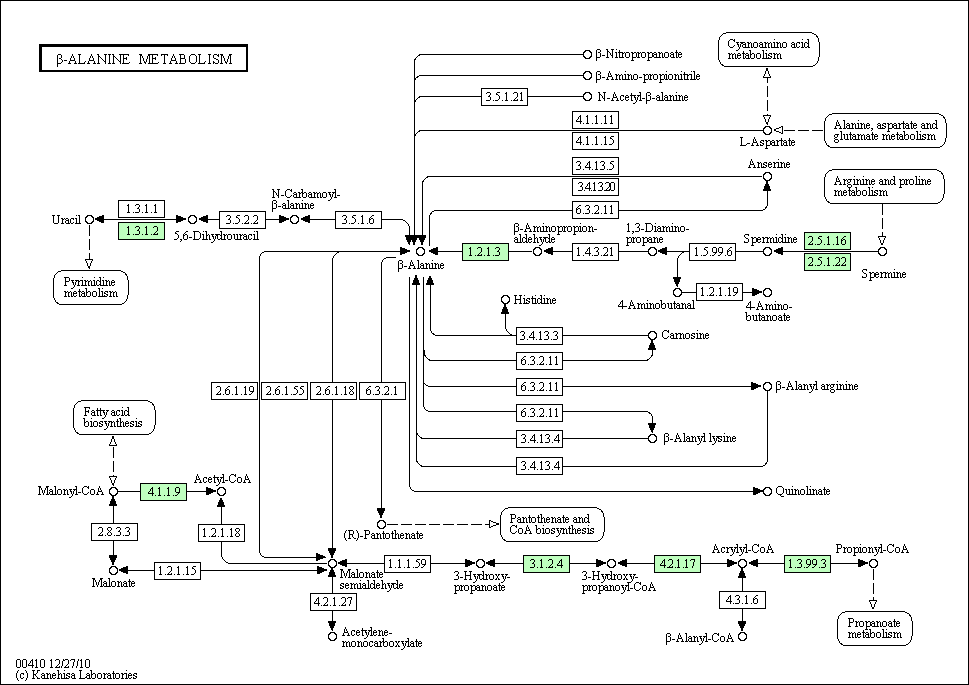

Supplement: Additional file 6 — KEGG annotation results. File contains the KEGG maps where isotigs were successfully mapped. Pathway element in green boxes indicates the components represented by at least, one isotig. Gilthead sea bream isotigs were automatically annotated to KEGG maps using KAAS website [60]. The SBH method, optimized for ESTs annotation, was used against human, chimpanzee, orang-utan, rhesus, mouse, rat, dog, giant panda, cow, pig, horse, opossum, platypus, chicken, clawed frog, zebrafish, fruit fly and nematode pathway databases. [file 1471-2164-13-181-S6.tar › map/map00410.png]

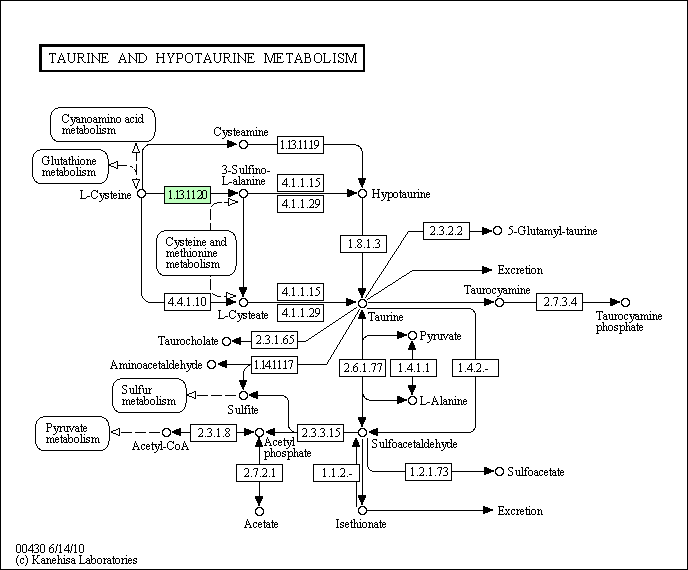

Supplement: Additional file 6 — KEGG annotation results. File contains the KEGG maps where isotigs were successfully mapped. Pathway element in green boxes indicates the components represented by at least, one isotig. Gilthead sea bream isotigs were automatically annotated to KEGG maps using KAAS website [60]. The SBH method, optimized for ESTs annotation, was used against human, chimpanzee, orang-utan, rhesus, mouse, rat, dog, giant panda, cow, pig, horse, opossum, platypus, chicken, clawed frog, zebrafish, fruit fly and nematode pathway databases. [file 1471-2164-13-181-S6.tar › map/map00430.png]

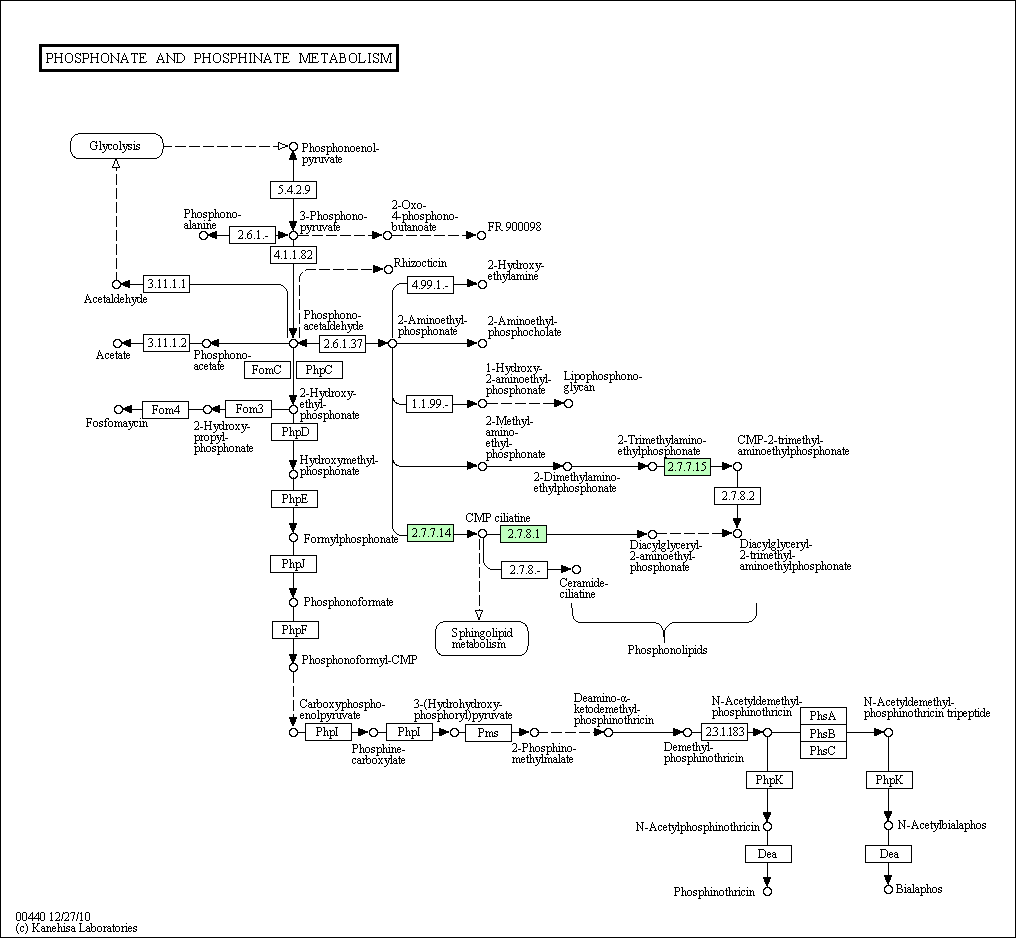

Supplement: Additional file 6 — KEGG annotation results. File contains the KEGG maps where isotigs were successfully mapped. Pathway element in green boxes indicates the components represented by at least, one isotig. Gilthead sea bream isotigs were automatically annotated to KEGG maps using KAAS website [60]. The SBH method, optimized for ESTs annotation, was used against human, chimpanzee, orang-utan, rhesus, mouse, rat, dog, giant panda, cow, pig, horse, opossum, platypus, chicken, clawed frog, zebrafish, fruit fly and nematode pathway databases. [file 1471-2164-13-181-S6.tar › map/map00440.png]

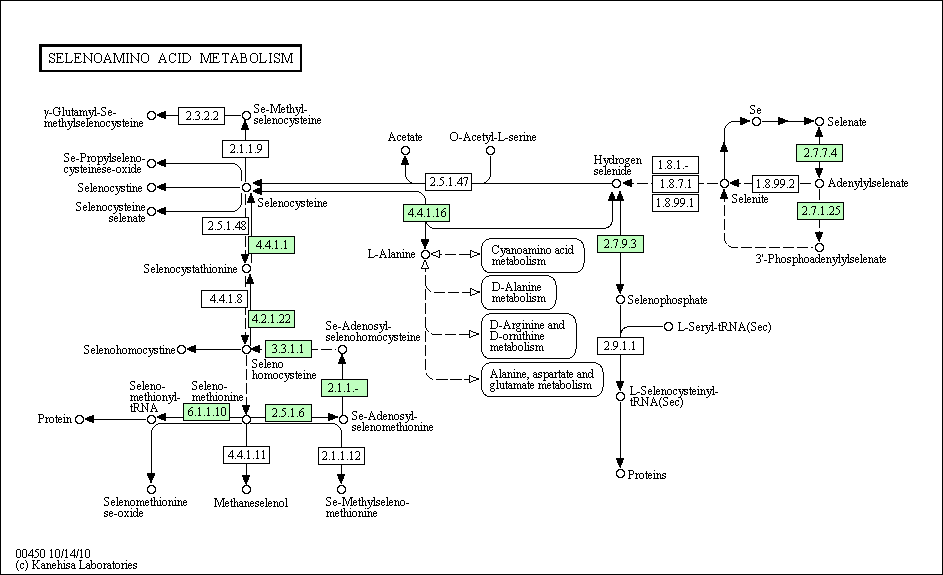

Supplement: Additional file 6 — KEGG annotation results. File contains the KEGG maps where isotigs were successfully mapped. Pathway element in green boxes indicates the components represented by at least, one isotig. Gilthead sea bream isotigs were automatically annotated to KEGG maps using KAAS website [60]. The SBH method, optimized for ESTs annotation, was used against human, chimpanzee, orang-utan, rhesus, mouse, rat, dog, giant panda, cow, pig, horse, opossum, platypus, chicken, clawed frog, zebrafish, fruit fly and nematode pathway databases. [file 1471-2164-13-181-S6.tar › map/map00450.png]

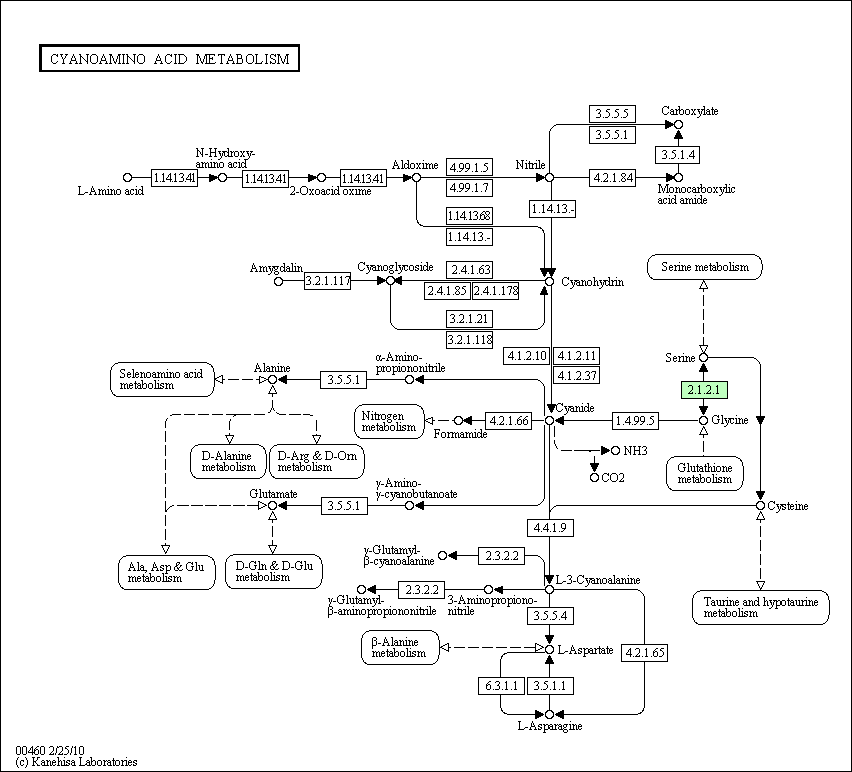

Supplement: Additional file 6 — KEGG annotation results. File contains the KEGG maps where isotigs were successfully mapped. Pathway element in green boxes indicates the components represented by at least, one isotig. Gilthead sea bream isotigs were automatically annotated to KEGG maps using KAAS website [60]. The SBH method, optimized for ESTs annotation, was used against human, chimpanzee, orang-utan, rhesus, mouse, rat, dog, giant panda, cow, pig, horse, opossum, platypus, chicken, clawed frog, zebrafish, fruit fly and nematode pathway databases. [file 1471-2164-13-181-S6.tar › map/map00460.png]

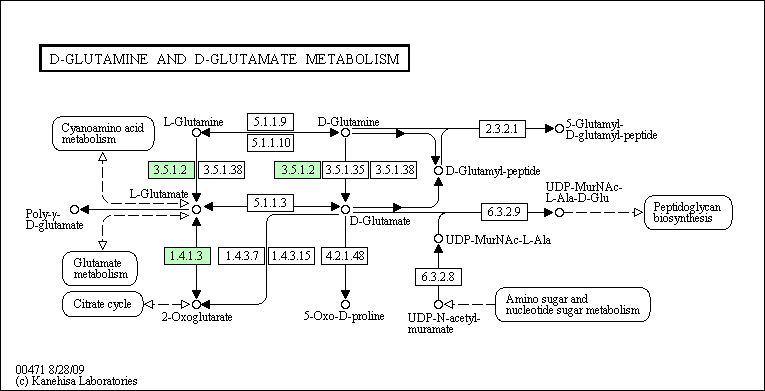

Supplement: Additional file 6 — KEGG annotation results. File contains the KEGG maps where isotigs were successfully mapped. Pathway element in green boxes indicates the components represented by at least, one isotig. Gilthead sea bream isotigs were automatically annotated to KEGG maps using KAAS website [60]. The SBH method, optimized for ESTs annotation, was used against human, chimpanzee, orang-utan, rhesus, mouse, rat, dog, giant panda, cow, pig, horse, opossum, platypus, chicken, clawed frog, zebrafish, fruit fly and nematode pathway databases. [file 1471-2164-13-181-S6.tar › map/map00471.png]

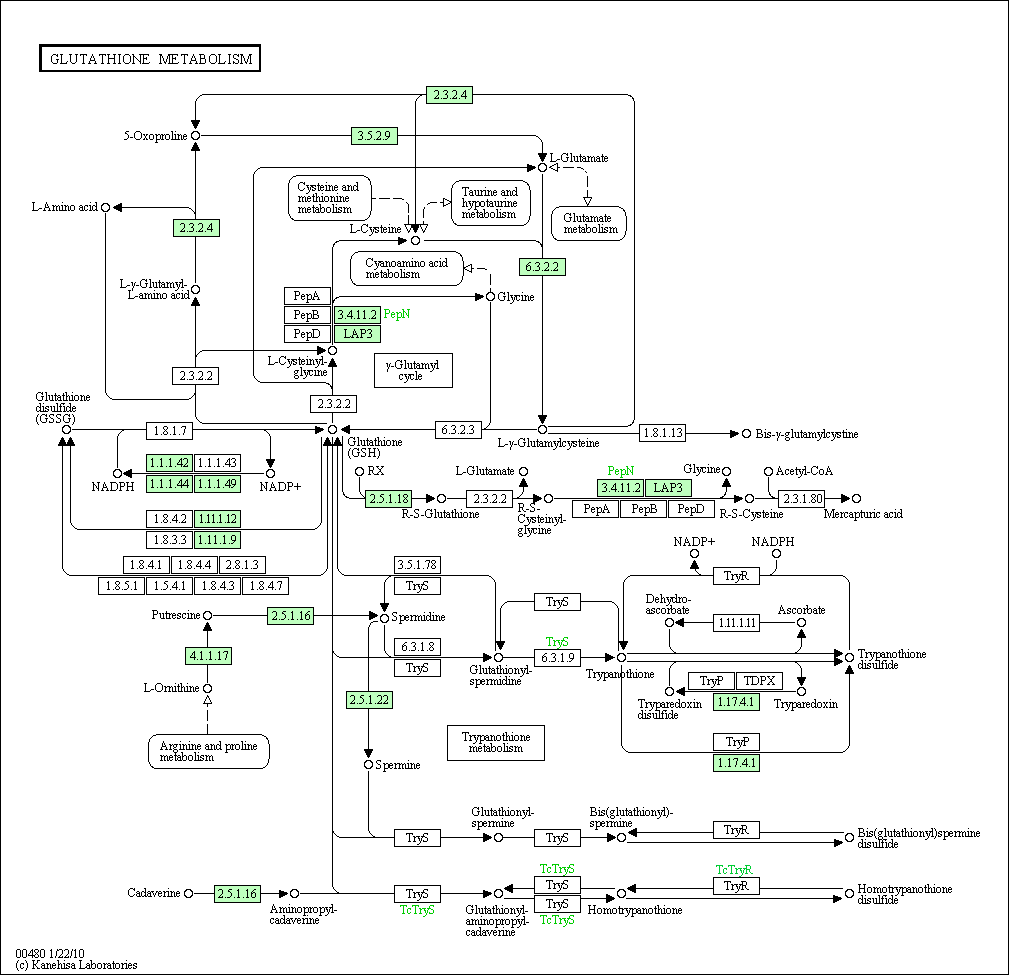

Supplement: Additional file 6 — KEGG annotation results. File contains the KEGG maps where isotigs were successfully mapped. Pathway element in green boxes indicates the components represented by at least, one isotig. Gilthead sea bream isotigs were automatically annotated to KEGG maps using KAAS website [60]. The SBH method, optimized for ESTs annotation, was used against human, chimpanzee, orang-utan, rhesus, mouse, rat, dog, giant panda, cow, pig, horse, opossum, platypus, chicken, clawed frog, zebrafish, fruit fly and nematode pathway databases. [file 1471-2164-13-181-S6.tar › map/map00480.png]

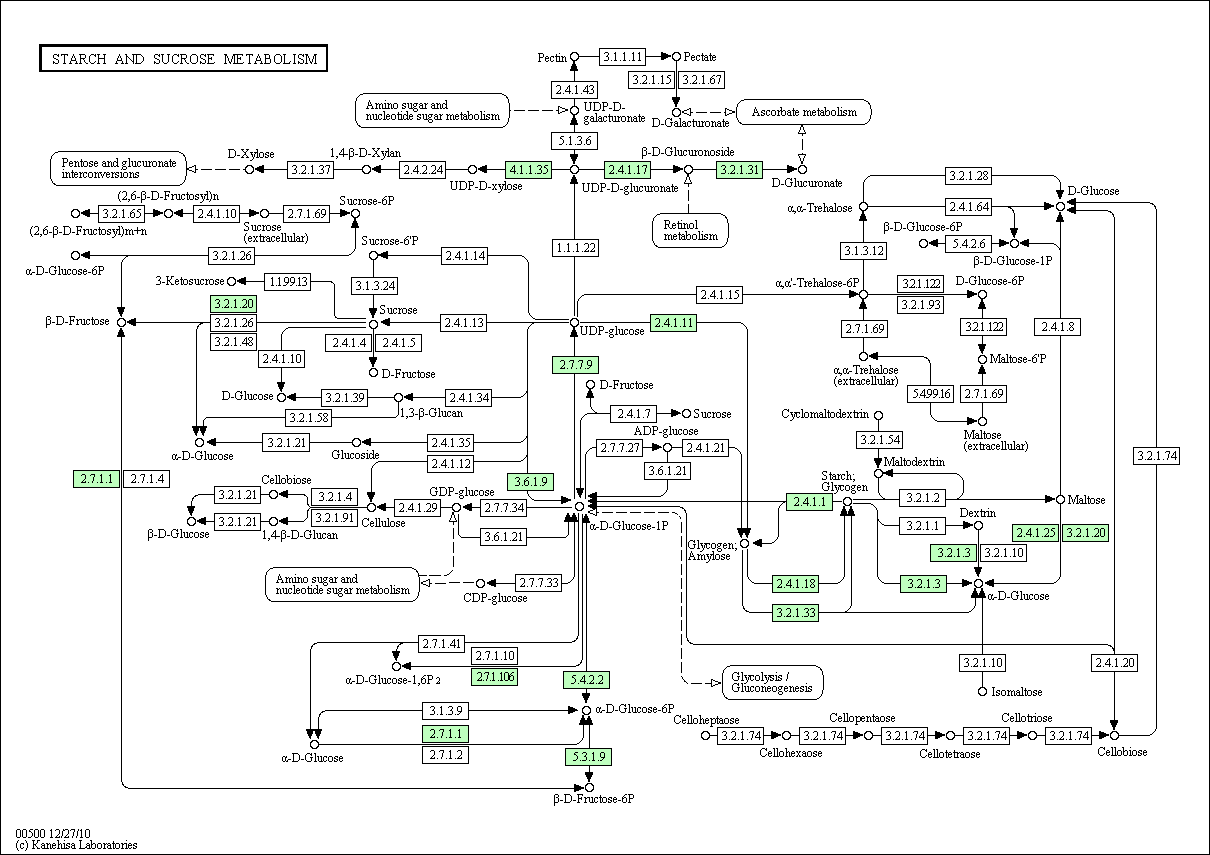

Supplement: Additional file 6 — KEGG annotation results. File contains the KEGG maps where isotigs were successfully mapped. Pathway element in green boxes indicates the components represented by at least, one isotig. Gilthead sea bream isotigs were automatically annotated to KEGG maps using KAAS website [60]. The SBH method, optimized for ESTs annotation, was used against human, chimpanzee, orang-utan, rhesus, mouse, rat, dog, giant panda, cow, pig, horse, opossum, platypus, chicken, clawed frog, zebrafish, fruit fly and nematode pathway databases. [file 1471-2164-13-181-S6.tar › map/map00500.png]

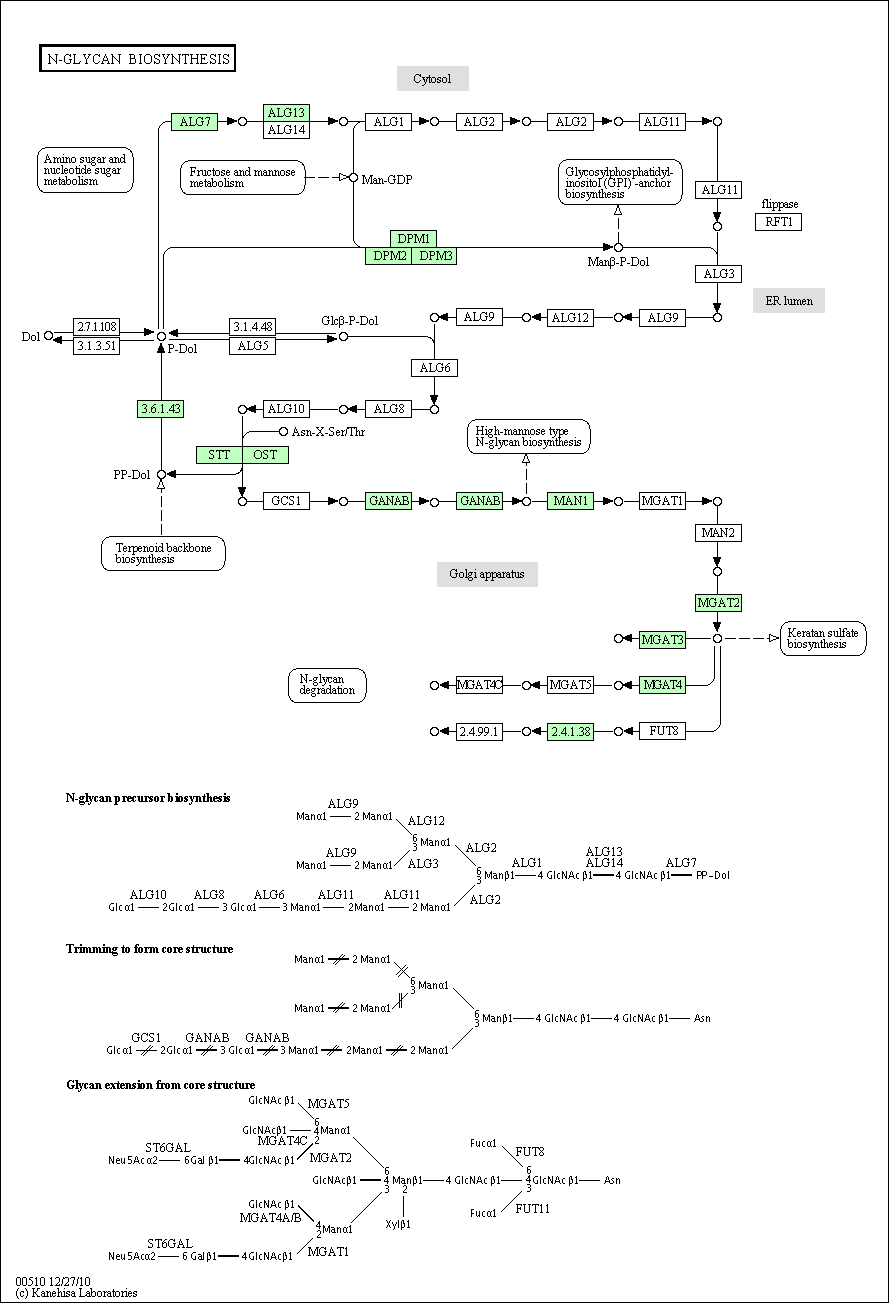

Supplement: Additional file 6 — KEGG annotation results. File contains the KEGG maps where isotigs were successfully mapped. Pathway element in green boxes indicates the components represented by at least, one isotig. Gilthead sea bream isotigs were automatically annotated to KEGG maps using KAAS website [60]. The SBH method, optimized for ESTs annotation, was used against human, chimpanzee, orang-utan, rhesus, mouse, rat, dog, giant panda, cow, pig, horse, opossum, platypus, chicken, clawed frog, zebrafish, fruit fly and nematode pathway databases. [file 1471-2164-13-181-S6.tar › map/map00510.png]

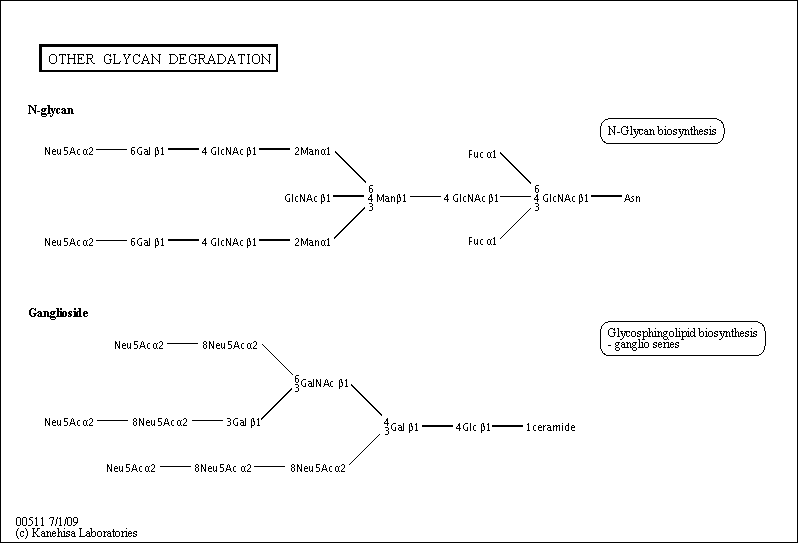

Supplement: Additional file 6 — KEGG annotation results. File contains the KEGG maps where isotigs were successfully mapped. Pathway element in green boxes indicates the components represented by at least, one isotig. Gilthead sea bream isotigs were automatically annotated to KEGG maps using KAAS website [60]. The SBH method, optimized for ESTs annotation, was used against human, chimpanzee, orang-utan, rhesus, mouse, rat, dog, giant panda, cow, pig, horse, opossum, platypus, chicken, clawed frog, zebrafish, fruit fly and nematode pathway databases. [file 1471-2164-13-181-S6.tar › map/map00511.png]

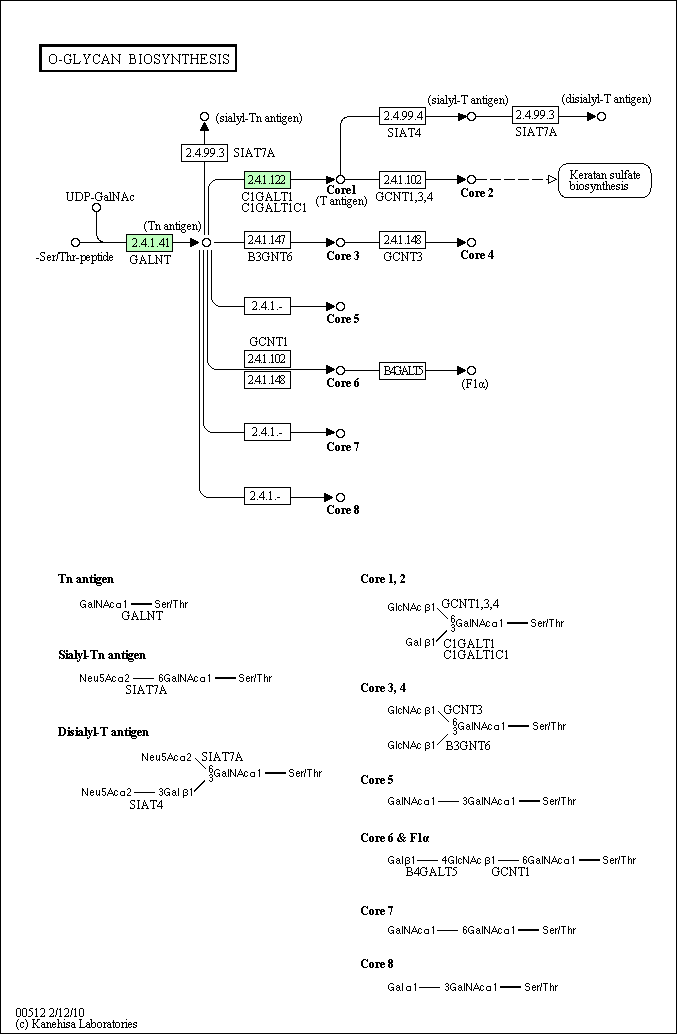

Supplement: Additional file 6 — KEGG annotation results. File contains the KEGG maps where isotigs were successfully mapped. Pathway element in green boxes indicates the components represented by at least, one isotig. Gilthead sea bream isotigs were automatically annotated to KEGG maps using KAAS website [60]. The SBH method, optimized for ESTs annotation, was used against human, chimpanzee, orang-utan, rhesus, mouse, rat, dog, giant panda, cow, pig, horse, opossum, platypus, chicken, clawed frog, zebrafish, fruit fly and nematode pathway databases. [file 1471-2164-13-181-S6.tar › map/map00512.png]

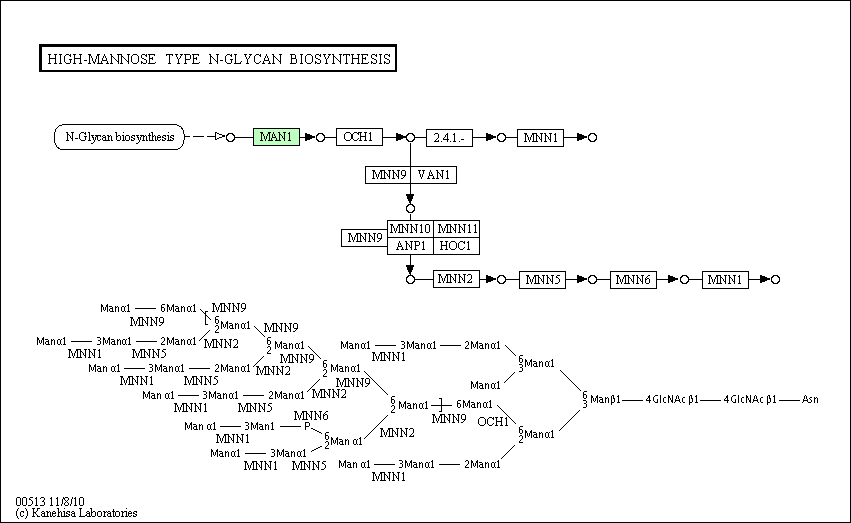

Supplement: Additional file 6 — KEGG annotation results. File contains the KEGG maps where isotigs were successfully mapped. Pathway element in green boxes indicates the components represented by at least, one isotig. Gilthead sea bream isotigs were automatically annotated to KEGG maps using KAAS website [60]. The SBH method, optimized for ESTs annotation, was used against human, chimpanzee, orang-utan, rhesus, mouse, rat, dog, giant panda, cow, pig, horse, opossum, platypus, chicken, clawed frog, zebrafish, fruit fly and nematode pathway databases. [file 1471-2164-13-181-S6.tar › map/map00513.png]

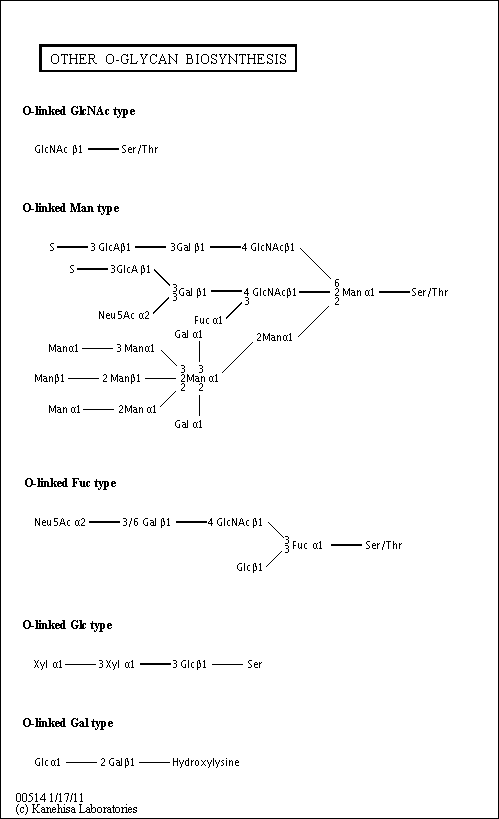

Supplement: Additional file 6 — KEGG annotation results. File contains the KEGG maps where isotigs were successfully mapped. Pathway element in green boxes indicates the components represented by at least, one isotig. Gilthead sea bream isotigs were automatically annotated to KEGG maps using KAAS website [60]. The SBH method, optimized for ESTs annotation, was used against human, chimpanzee, orang-utan, rhesus, mouse, rat, dog, giant panda, cow, pig, horse, opossum, platypus, chicken, clawed frog, zebrafish, fruit fly and nematode pathway databases. [file 1471-2164-13-181-S6.tar › map/map00514.png]

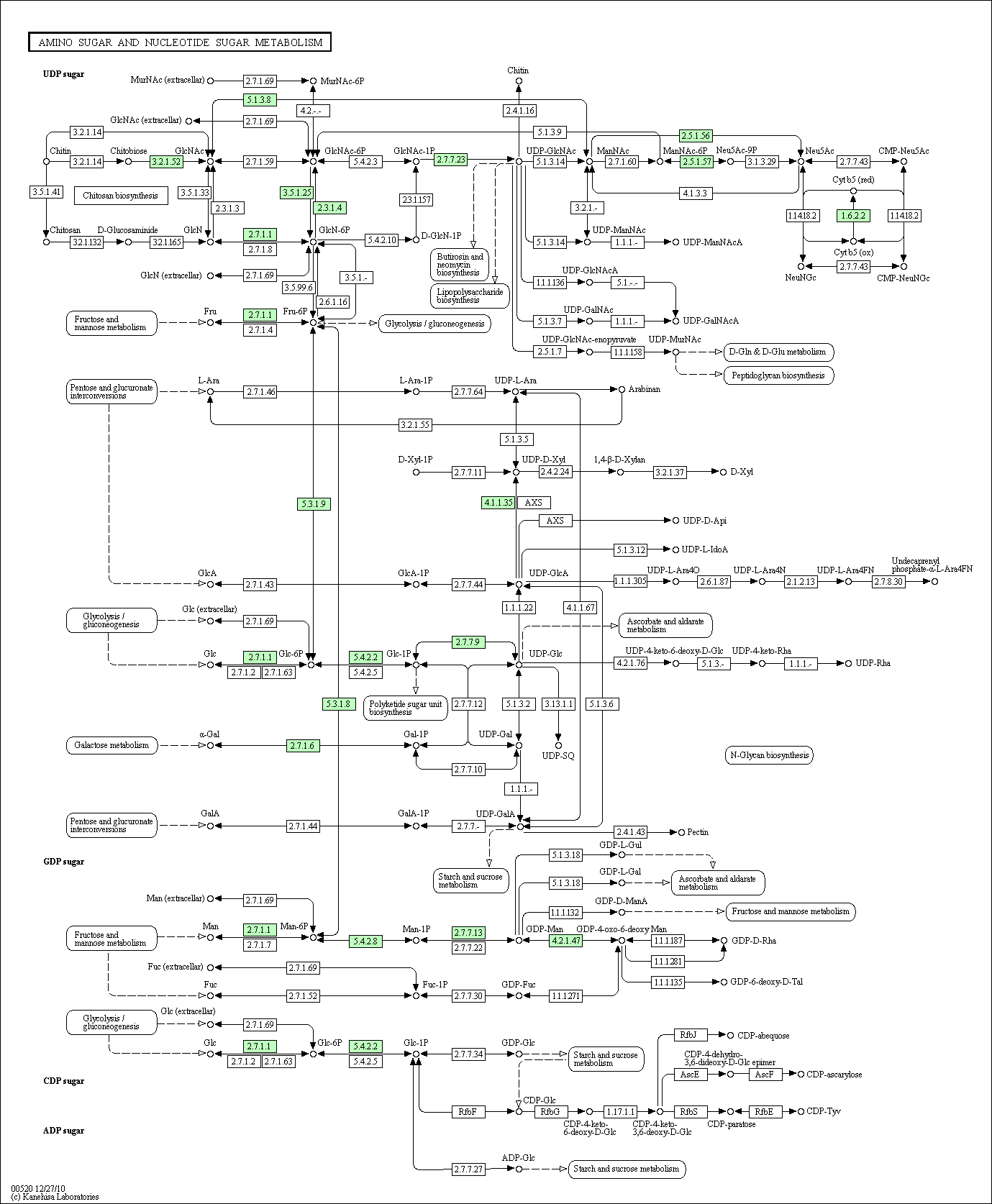

Supplement: Additional file 6 — KEGG annotation results. File contains the KEGG maps where isotigs were successfully mapped. Pathway element in green boxes indicates the components represented by at least, one isotig. Gilthead sea bream isotigs were automatically annotated to KEGG maps using KAAS website [60]. The SBH method, optimized for ESTs annotation, was used against human, chimpanzee, orang-utan, rhesus, mouse, rat, dog, giant panda, cow, pig, horse, opossum, platypus, chicken, clawed frog, zebrafish, fruit fly and nematode pathway databases. [file 1471-2164-13-181-S6.tar › map/map00520.png]

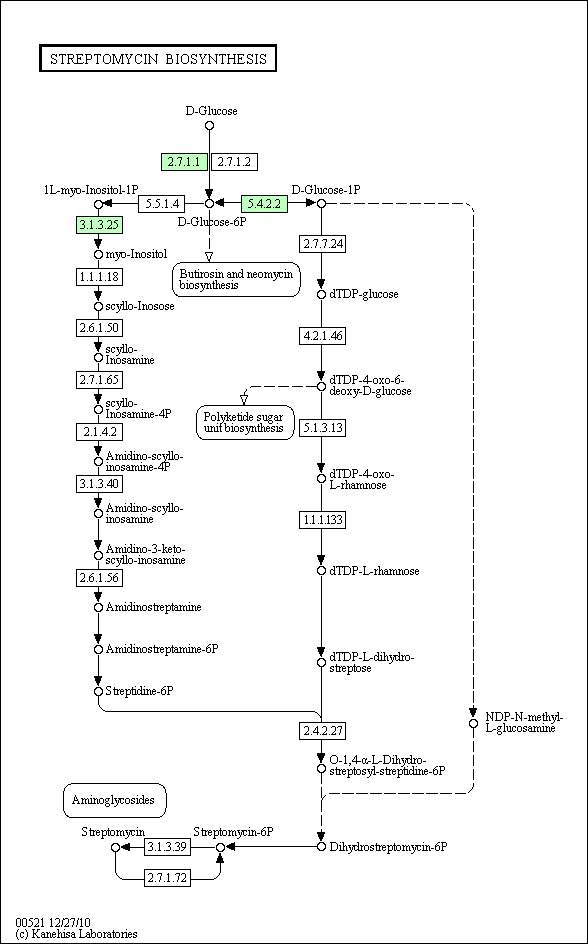

Supplement: Additional file 6 — KEGG annotation results. File contains the KEGG maps where isotigs were successfully mapped. Pathway element in green boxes indicates the components represented by at least, one isotig. Gilthead sea bream isotigs were automatically annotated to KEGG maps using KAAS website [60]. The SBH method, optimized for ESTs annotation, was used against human, chimpanzee, orang-utan, rhesus, mouse, rat, dog, giant panda, cow, pig, horse, opossum, platypus, chicken, clawed frog, zebrafish, fruit fly and nematode pathway databases. [file 1471-2164-13-181-S6.tar › map/map00521.png]

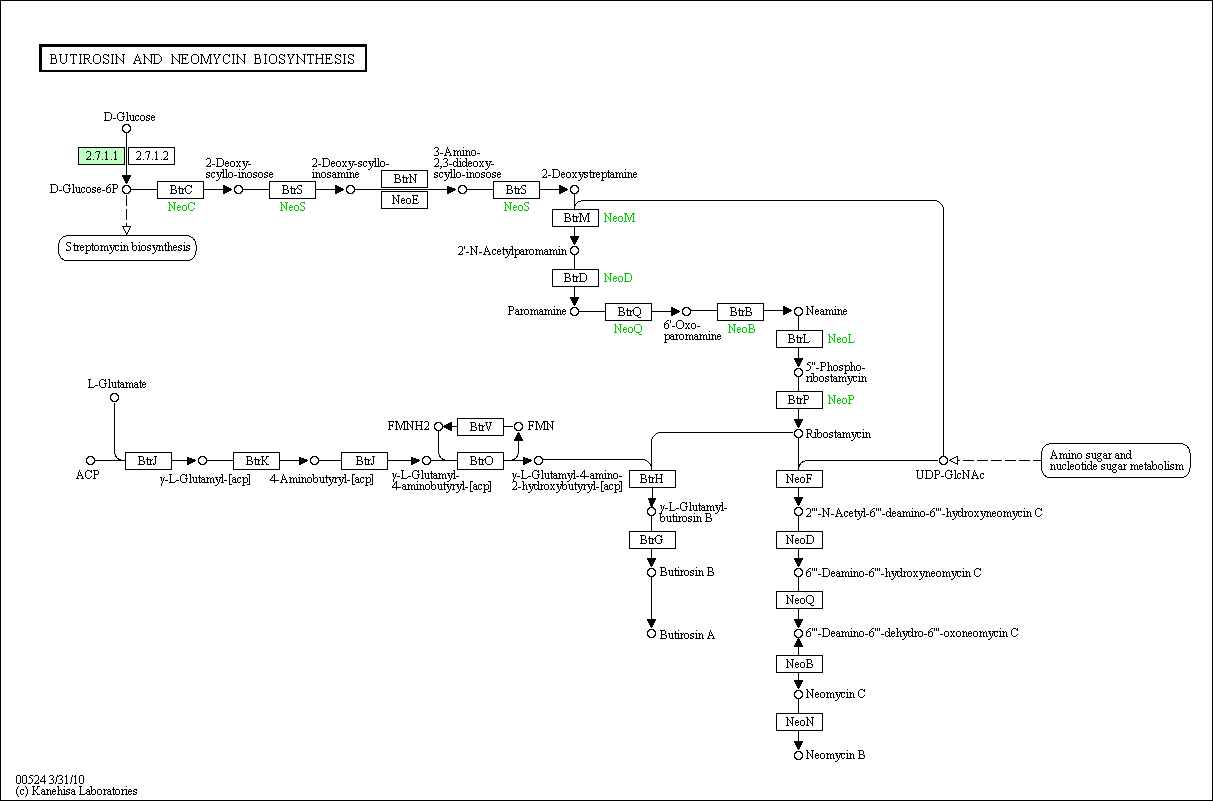

Supplement: Additional file 6 — KEGG annotation results. File contains the KEGG maps where isotigs were successfully mapped. Pathway element in green boxes indicates the components represented by at least, one isotig. Gilthead sea bream isotigs were automatically annotated to KEGG maps using KAAS website [60]. The SBH method, optimized for ESTs annotation, was used against human, chimpanzee, orang-utan, rhesus, mouse, rat, dog, giant panda, cow, pig, horse, opossum, platypus, chicken, clawed frog, zebrafish, fruit fly and nematode pathway databases. [file 1471-2164-13-181-S6.tar › map/map00524.png]

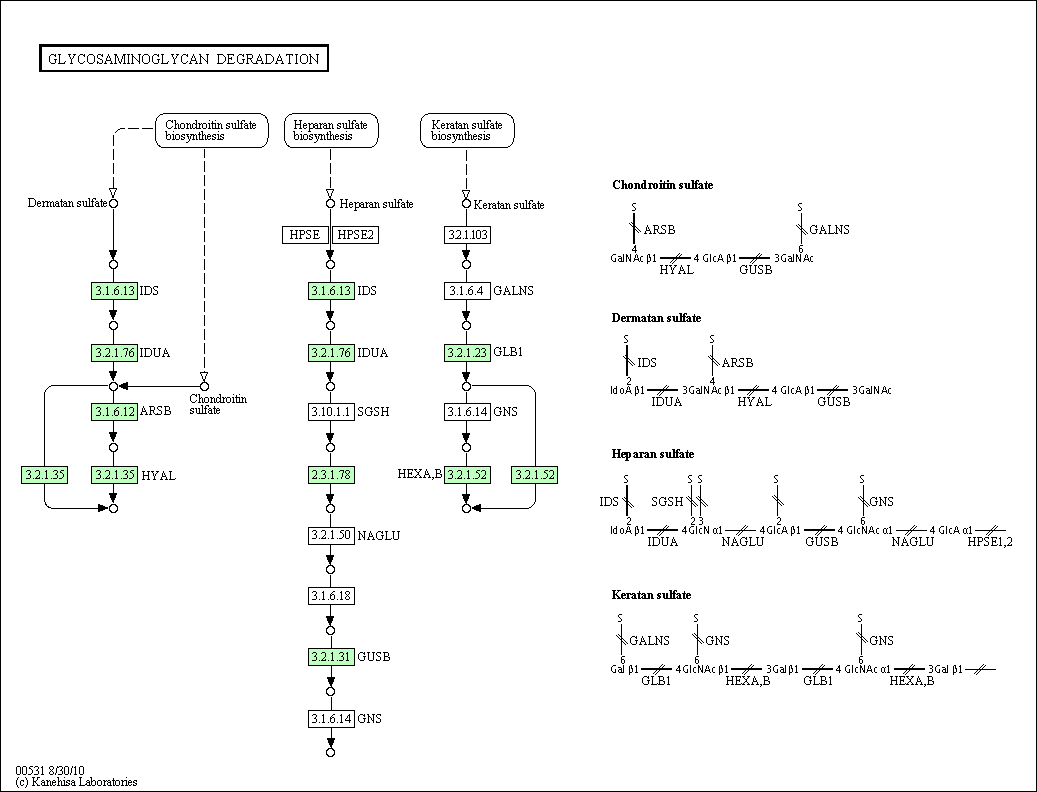

Supplement: Additional file 6 — KEGG annotation results. File contains the KEGG maps where isotigs were successfully mapped. Pathway element in green boxes indicates the components represented by at least, one isotig. Gilthead sea bream isotigs were automatically annotated to KEGG maps using KAAS website [60]. The SBH method, optimized for ESTs annotation, was used against human, chimpanzee, orang-utan, rhesus, mouse, rat, dog, giant panda, cow, pig, horse, opossum, platypus, chicken, clawed frog, zebrafish, fruit fly and nematode pathway databases. [file 1471-2164-13-181-S6.tar › map/map00531.png]

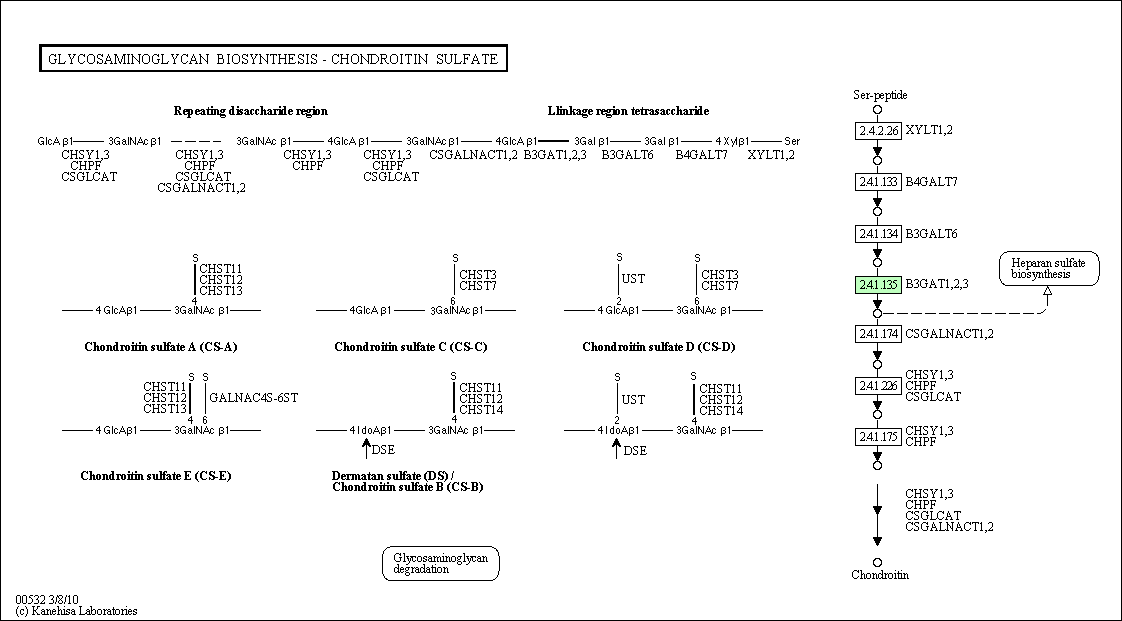

Supplement: Additional file 6 — KEGG annotation results. File contains the KEGG maps where isotigs were successfully mapped. Pathway element in green boxes indicates the components represented by at least, one isotig. Gilthead sea bream isotigs were automatically annotated to KEGG maps using KAAS website [60]. The SBH method, optimized for ESTs annotation, was used against human, chimpanzee, orang-utan, rhesus, mouse, rat, dog, giant panda, cow, pig, horse, opossum, platypus, chicken, clawed frog, zebrafish, fruit fly and nematode pathway databases. [file 1471-2164-13-181-S6.tar › map/map00532.png]

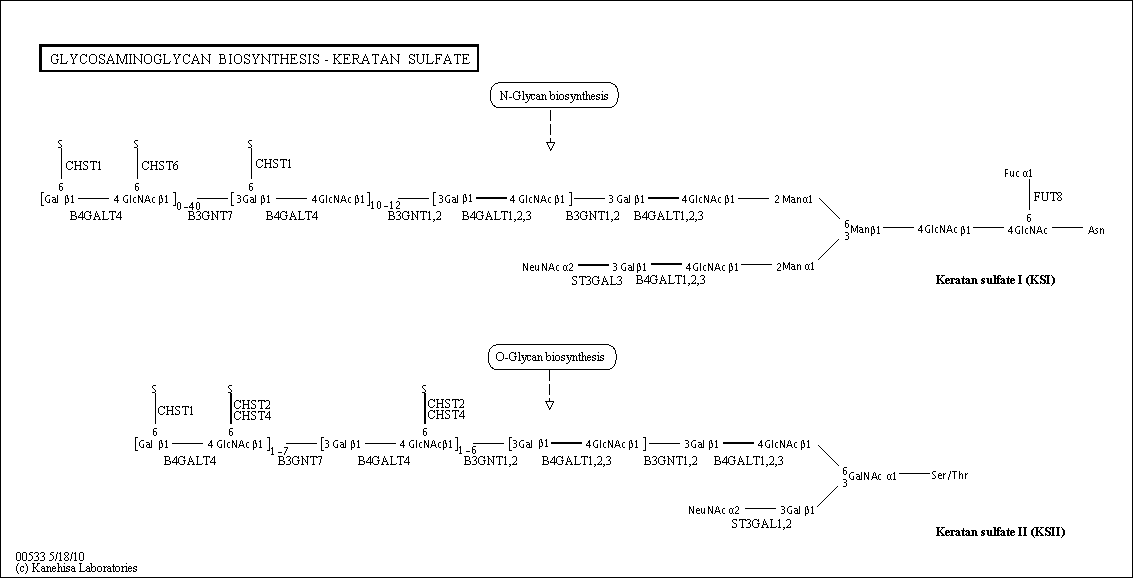

Supplement: Additional file 6 — KEGG annotation results. File contains the KEGG maps where isotigs were successfully mapped. Pathway element in green boxes indicates the components represented by at least, one isotig. Gilthead sea bream isotigs were automatically annotated to KEGG maps using KAAS website [60]. The SBH method, optimized for ESTs annotation, was used against human, chimpanzee, orang-utan, rhesus, mouse, rat, dog, giant panda, cow, pig, horse, opossum, platypus, chicken, clawed frog, zebrafish, fruit fly and nematode pathway databases. [file 1471-2164-13-181-S6.tar › map/map00533.png]

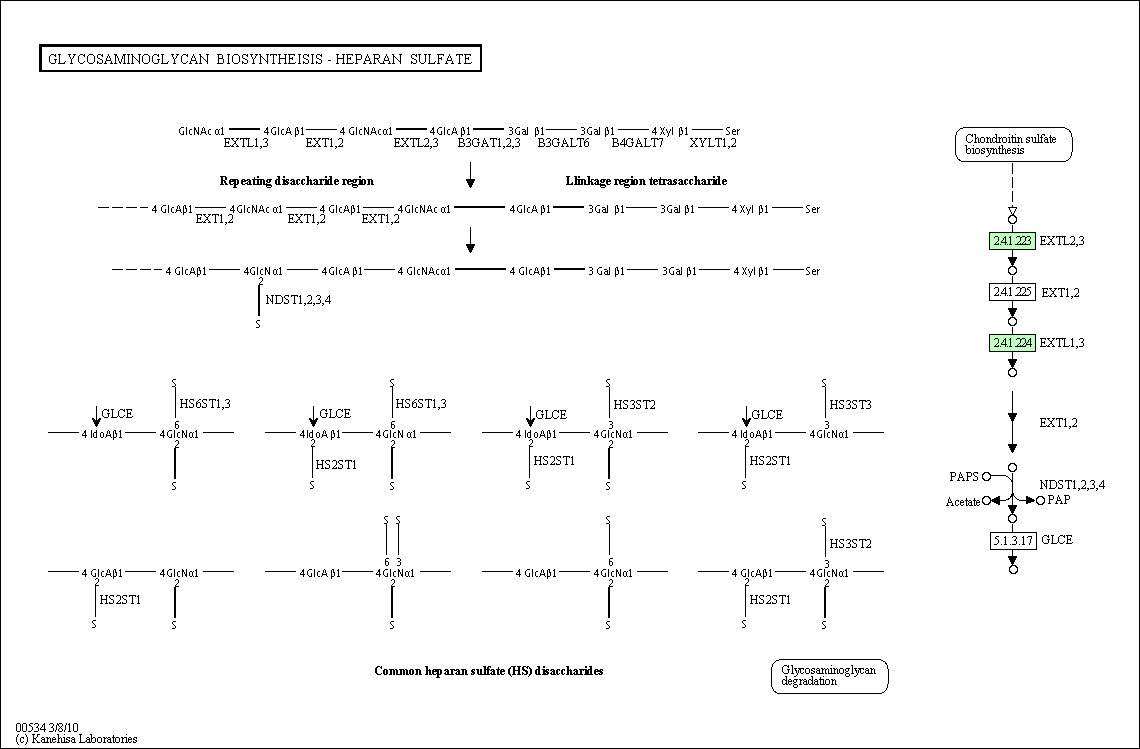

Supplement: Additional file 6 — KEGG annotation results. File contains the KEGG maps where isotigs were successfully mapped. Pathway element in green boxes indicates the components represented by at least, one isotig. Gilthead sea bream isotigs were automatically annotated to KEGG maps using KAAS website [60]. The SBH method, optimized for ESTs annotation, was used against human, chimpanzee, orang-utan, rhesus, mouse, rat, dog, giant panda, cow, pig, horse, opossum, platypus, chicken, clawed frog, zebrafish, fruit fly and nematode pathway databases. [file 1471-2164-13-181-S6.tar › map/map00534.png]

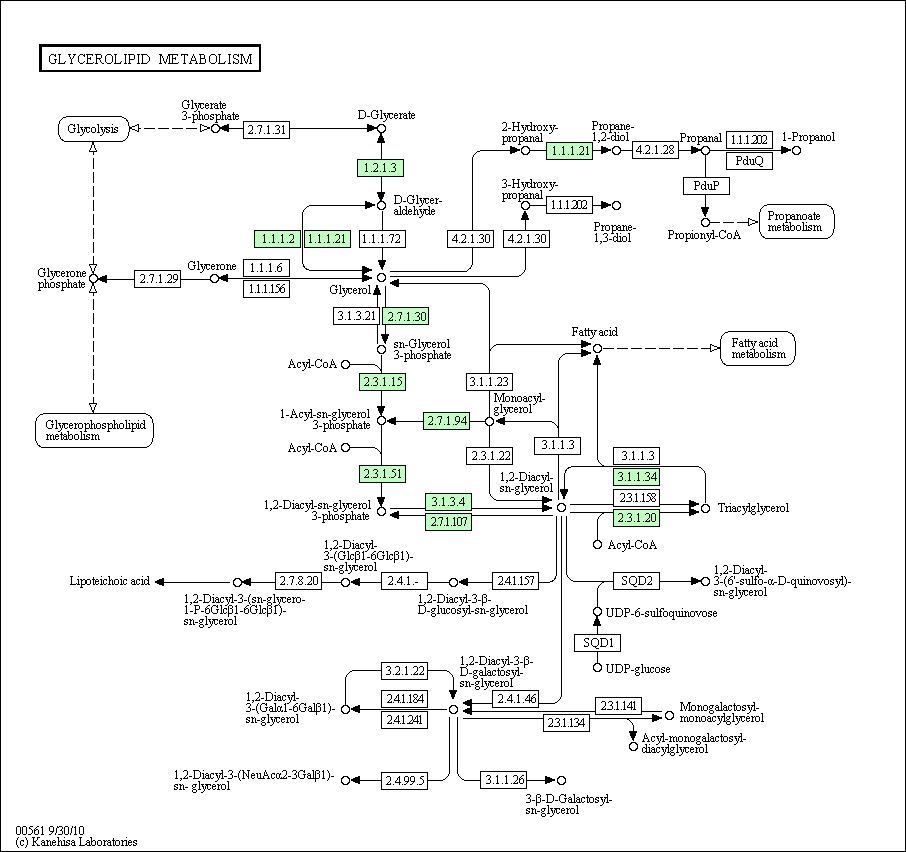

Supplement: Additional file 6 — KEGG annotation results. File contains the KEGG maps where isotigs were successfully mapped. Pathway element in green boxes indicates the components represented by at least, one isotig. Gilthead sea bream isotigs were automatically annotated to KEGG maps using KAAS website [60]. The SBH method, optimized for ESTs annotation, was used against human, chimpanzee, orang-utan, rhesus, mouse, rat, dog, giant panda, cow, pig, horse, opossum, platypus, chicken, clawed frog, zebrafish, fruit fly and nematode pathway databases. [file 1471-2164-13-181-S6.tar › map/map00561.png]

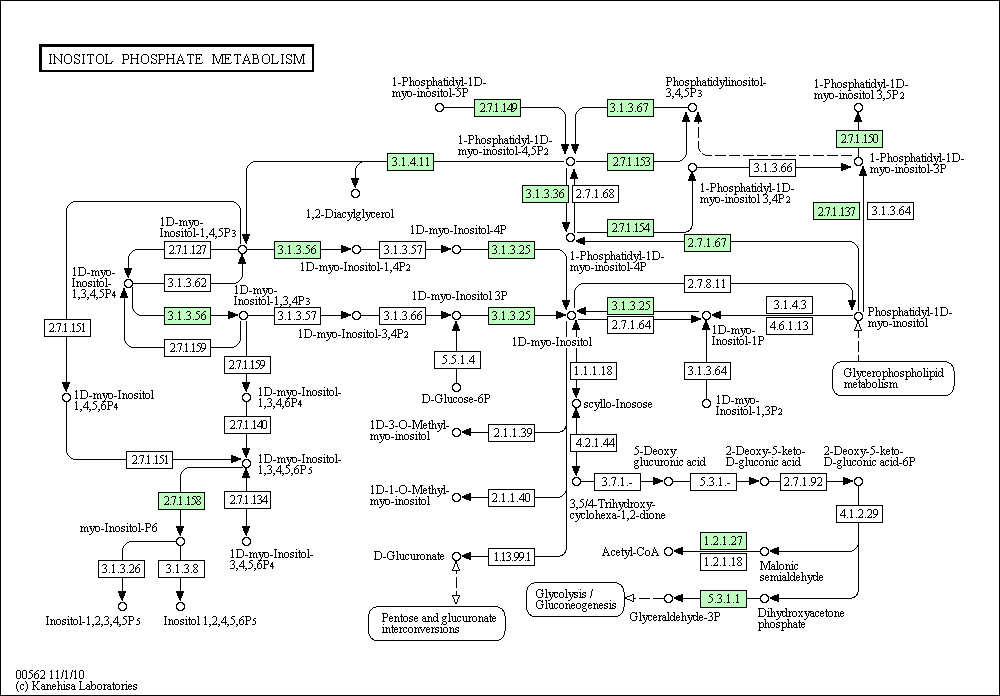

Supplement: Additional file 6 — KEGG annotation results. File contains the KEGG maps where isotigs were successfully mapped. Pathway element in green boxes indicates the components represented by at least, one isotig. Gilthead sea bream isotigs were automatically annotated to KEGG maps using KAAS website [60]. The SBH method, optimized for ESTs annotation, was used against human, chimpanzee, orang-utan, rhesus, mouse, rat, dog, giant panda, cow, pig, horse, opossum, platypus, chicken, clawed frog, zebrafish, fruit fly and nematode pathway databases. [file 1471-2164-13-181-S6.tar › map/map00562.png]

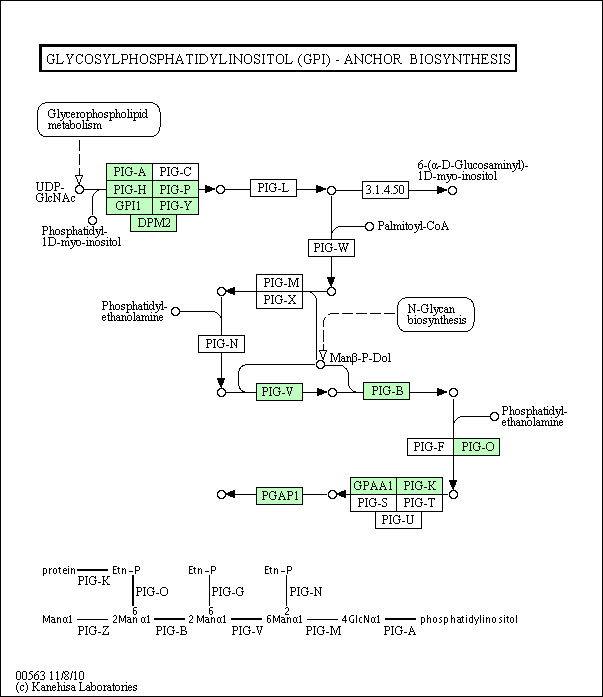

Supplement: Additional file 6 — KEGG annotation results. File contains the KEGG maps where isotigs were successfully mapped. Pathway element in green boxes indicates the components represented by at least, one isotig. Gilthead sea bream isotigs were automatically annotated to KEGG maps using KAAS website [60]. The SBH method, optimized for ESTs annotation, was used against human, chimpanzee, orang-utan, rhesus, mouse, rat, dog, giant panda, cow, pig, horse, opossum, platypus, chicken, clawed frog, zebrafish, fruit fly and nematode pathway databases. [file 1471-2164-13-181-S6.tar › map/map00563.png]

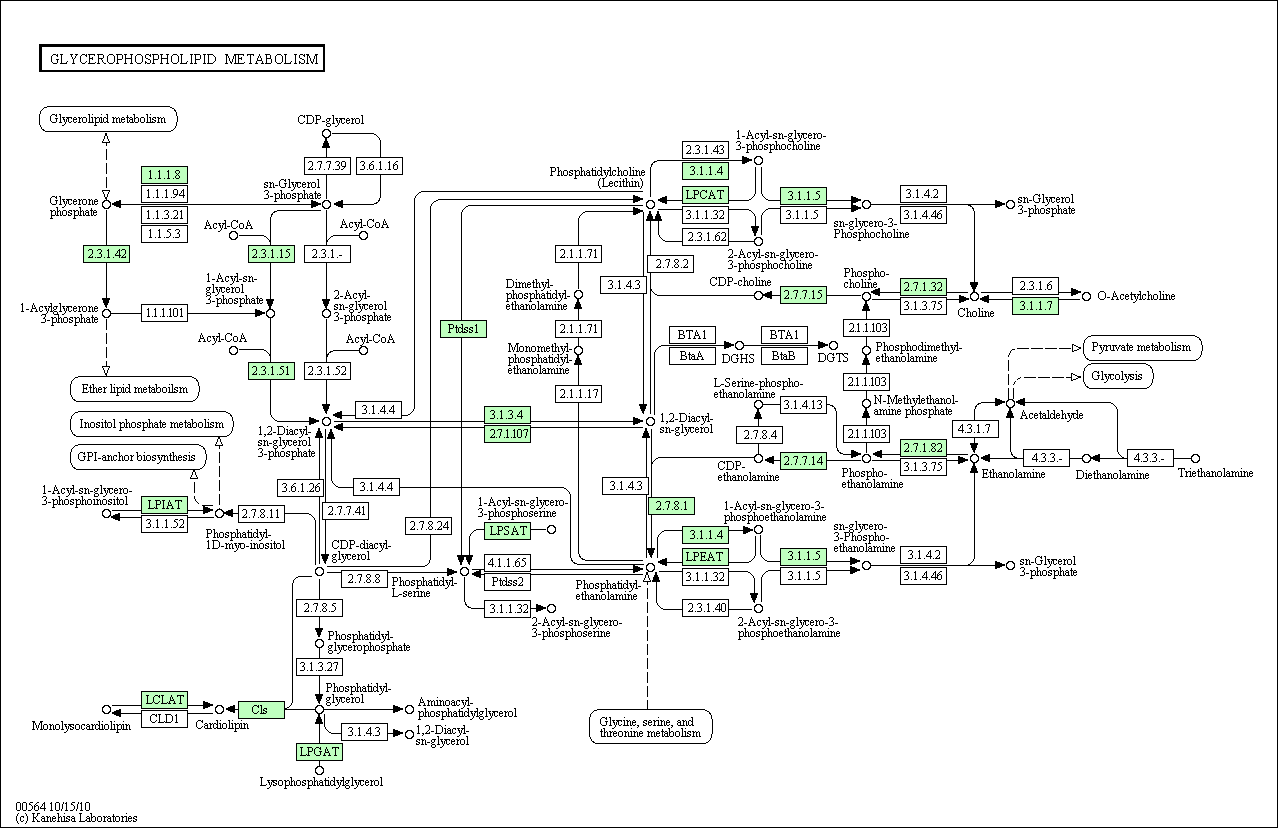

Supplement: Additional file 6 — KEGG annotation results. File contains the KEGG maps where isotigs were successfully mapped. Pathway element in green boxes indicates the components represented by at least, one isotig. Gilthead sea bream isotigs were automatically annotated to KEGG maps using KAAS website [60]. The SBH method, optimized for ESTs annotation, was used against human, chimpanzee, orang-utan, rhesus, mouse, rat, dog, giant panda, cow, pig, horse, opossum, platypus, chicken, clawed frog, zebrafish, fruit fly and nematode pathway databases. [file 1471-2164-13-181-S6.tar › map/map00564.png]

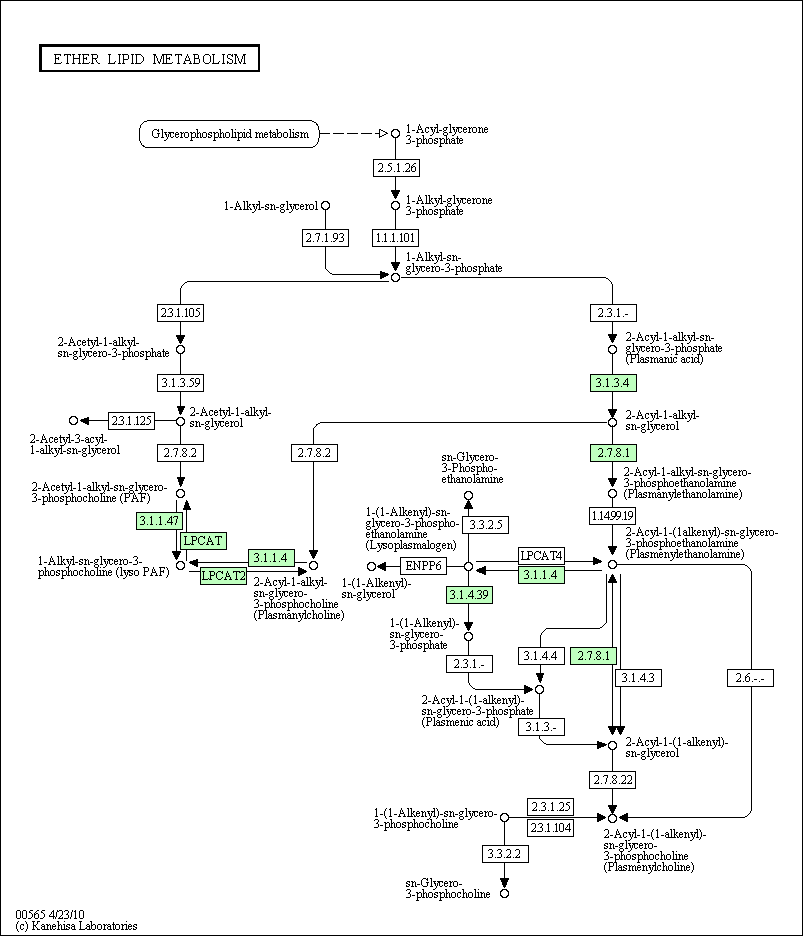

Supplement: Additional file 6 — KEGG annotation results. File contains the KEGG maps where isotigs were successfully mapped. Pathway element in green boxes indicates the components represented by at least, one isotig. Gilthead sea bream isotigs were automatically annotated to KEGG maps using KAAS website [60]. The SBH method, optimized for ESTs annotation, was used against human, chimpanzee, orang-utan, rhesus, mouse, rat, dog, giant panda, cow, pig, horse, opossum, platypus, chicken, clawed frog, zebrafish, fruit fly and nematode pathway databases. [file 1471-2164-13-181-S6.tar › map/map00565.png]

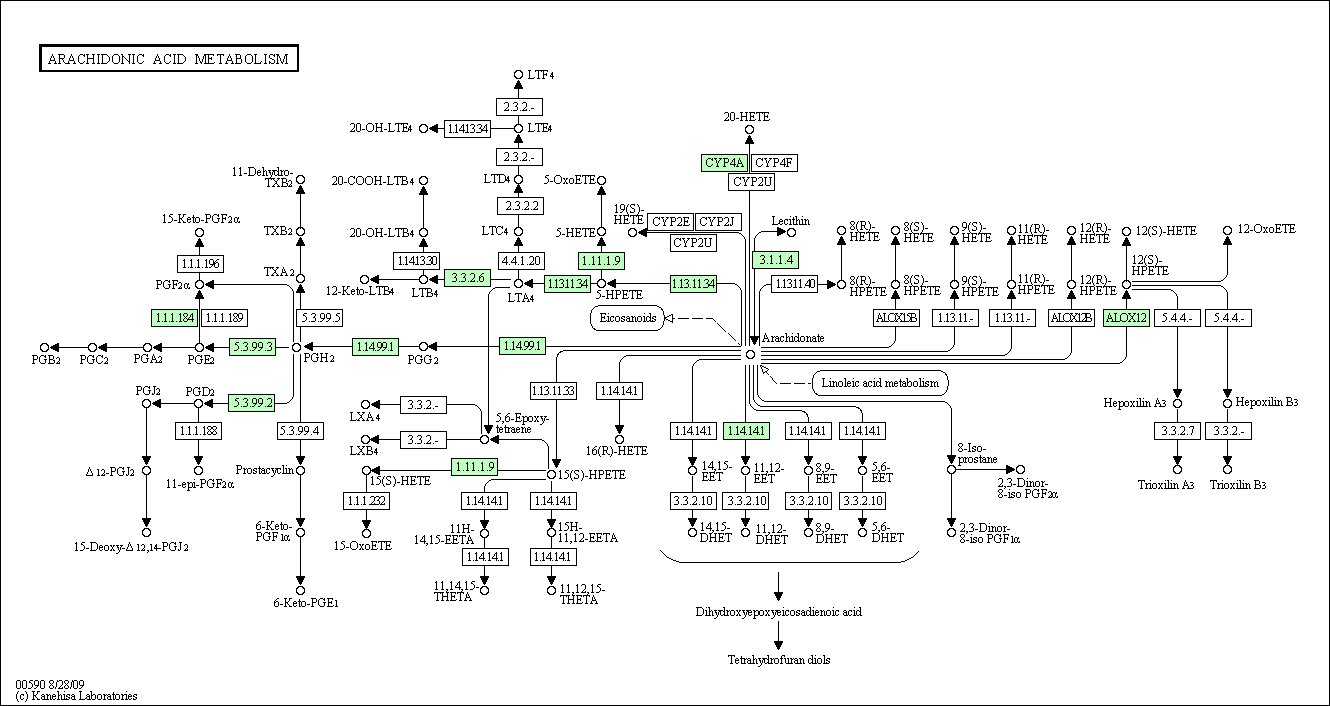

Supplement: Additional file 6 — KEGG annotation results. File contains the KEGG maps where isotigs were successfully mapped. Pathway element in green boxes indicates the components represented by at least, one isotig. Gilthead sea bream isotigs were automatically annotated to KEGG maps using KAAS website [60]. The SBH method, optimized for ESTs annotation, was used against human, chimpanzee, orang-utan, rhesus, mouse, rat, dog, giant panda, cow, pig, horse, opossum, platypus, chicken, clawed frog, zebrafish, fruit fly and nematode pathway databases. [file 1471-2164-13-181-S6.tar › map/map00590.png]

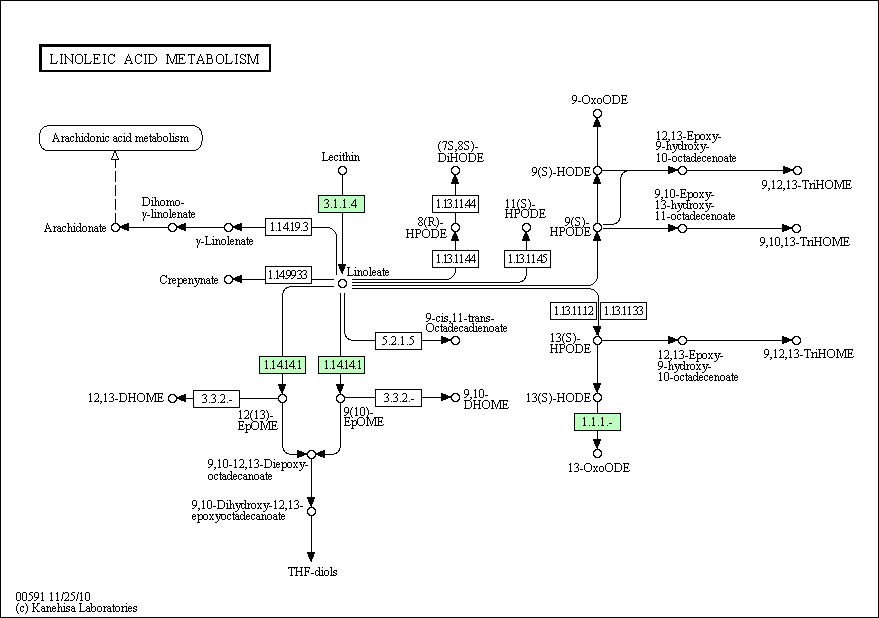

Supplement: Additional file 6 — KEGG annotation results. File contains the KEGG maps where isotigs were successfully mapped. Pathway element in green boxes indicates the components represented by at least, one isotig. Gilthead sea bream isotigs were automatically annotated to KEGG maps using KAAS website [60]. The SBH method, optimized for ESTs annotation, was used against human, chimpanzee, orang-utan, rhesus, mouse, rat, dog, giant panda, cow, pig, horse, opossum, platypus, chicken, clawed frog, zebrafish, fruit fly and nematode pathway databases. [file 1471-2164-13-181-S6.tar › map/map00591.png]

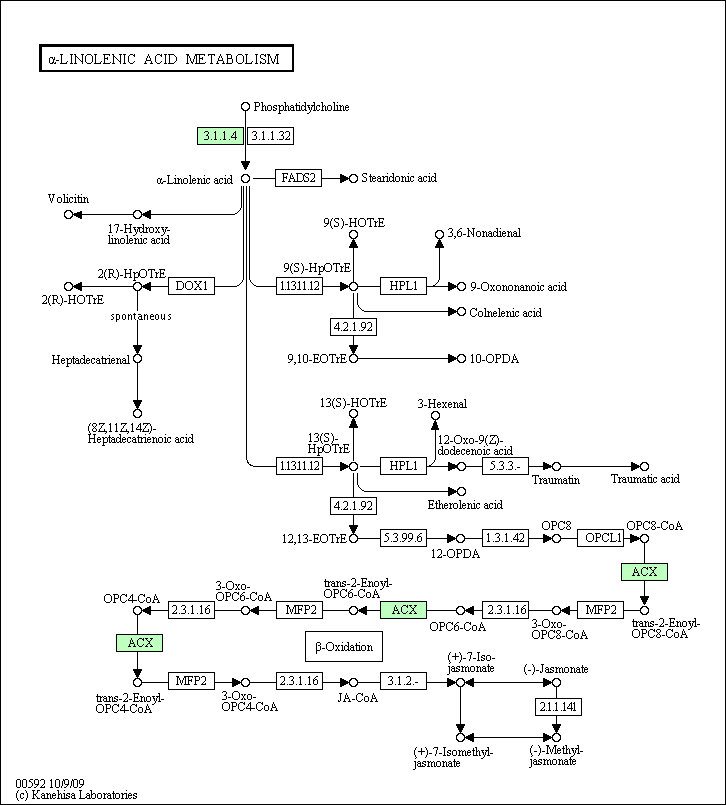

Supplement: Additional file 6 — KEGG annotation results. File contains the KEGG maps where isotigs were successfully mapped. Pathway element in green boxes indicates the components represented by at least, one isotig. Gilthead sea bream isotigs were automatically annotated to KEGG maps using KAAS website [60]. The SBH method, optimized for ESTs annotation, was used against human, chimpanzee, orang-utan, rhesus, mouse, rat, dog, giant panda, cow, pig, horse, opossum, platypus, chicken, clawed frog, zebrafish, fruit fly and nematode pathway databases. [file 1471-2164-13-181-S6.tar › map/map00592.png]

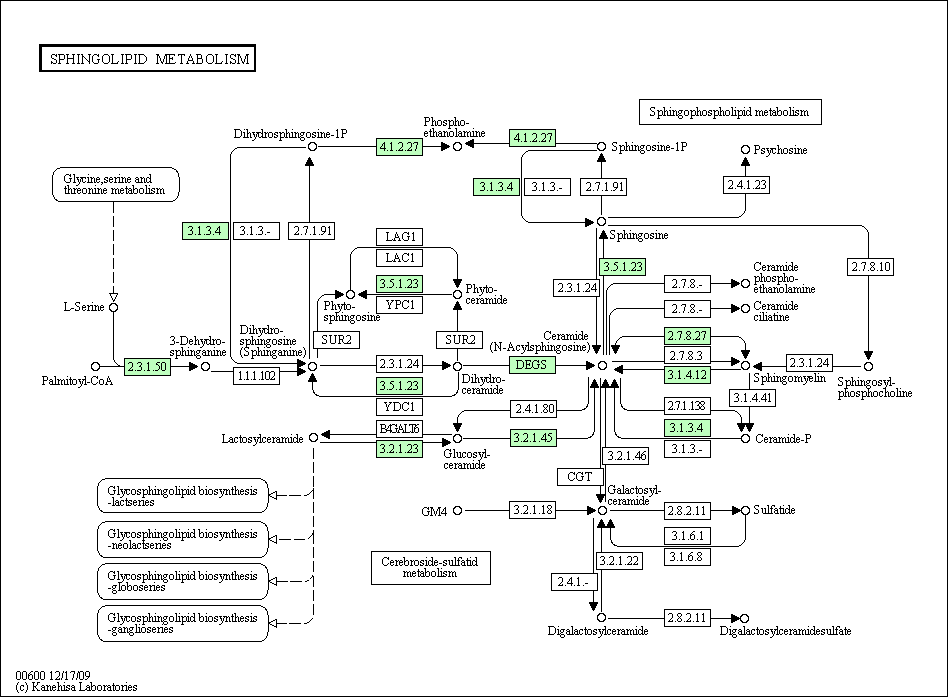

Supplement: Additional file 6 — KEGG annotation results. File contains the KEGG maps where isotigs were successfully mapped. Pathway element in green boxes indicates the components represented by at least, one isotig. Gilthead sea bream isotigs were automatically annotated to KEGG maps using KAAS website [60]. The SBH method, optimized for ESTs annotation, was used against human, chimpanzee, orang-utan, rhesus, mouse, rat, dog, giant panda, cow, pig, horse, opossum, platypus, chicken, clawed frog, zebrafish, fruit fly and nematode pathway databases. [file 1471-2164-13-181-S6.tar › map/map00600.png]

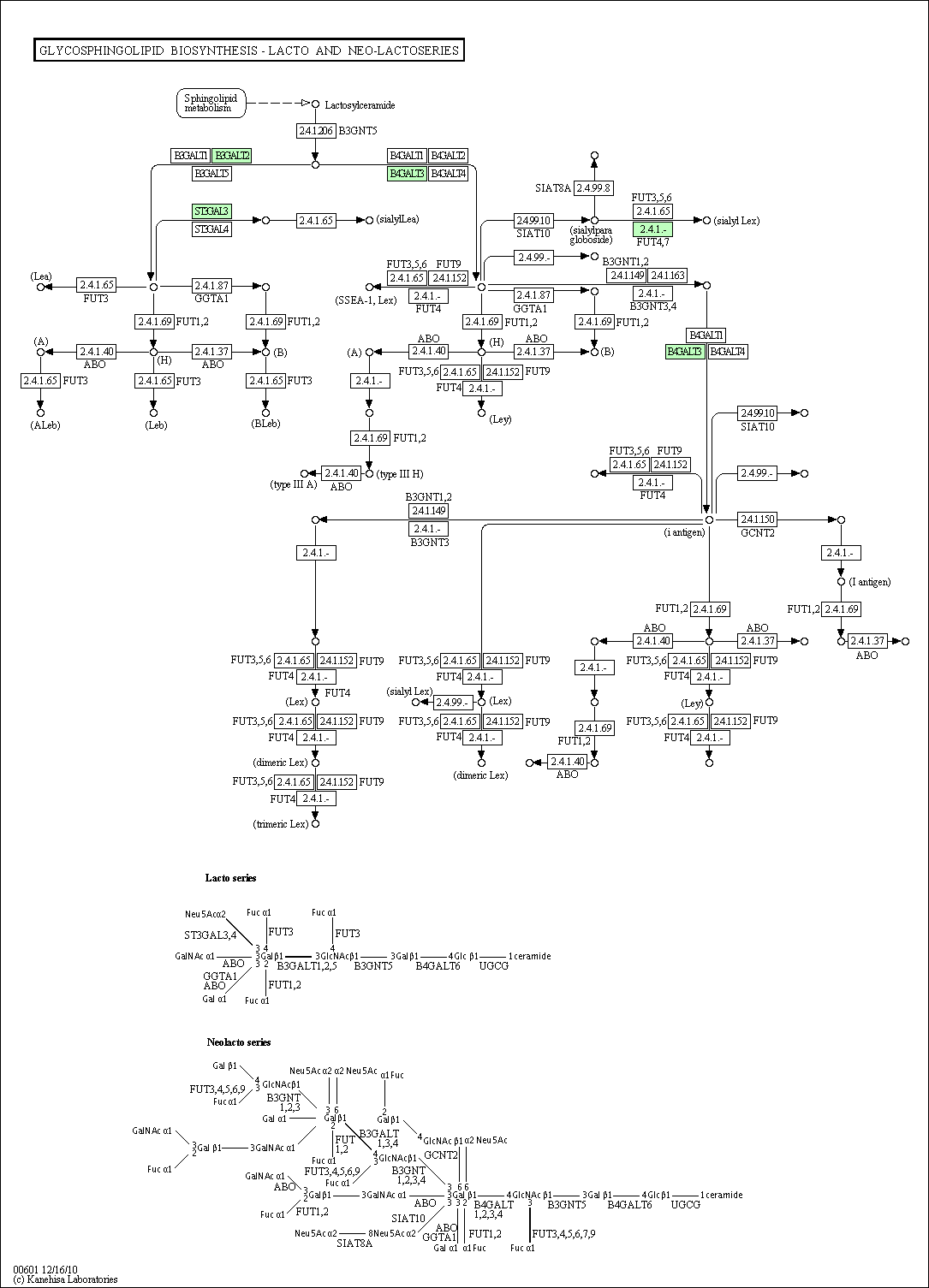

Supplement: Additional file 6 — KEGG annotation results. File contains the KEGG maps where isotigs were successfully mapped. Pathway element in green boxes indicates the components represented by at least, one isotig. Gilthead sea bream isotigs were automatically annotated to KEGG maps using KAAS website [60]. The SBH method, optimized for ESTs annotation, was used against human, chimpanzee, orang-utan, rhesus, mouse, rat, dog, giant panda, cow, pig, horse, opossum, platypus, chicken, clawed frog, zebrafish, fruit fly and nematode pathway databases. [file 1471-2164-13-181-S6.tar › map/map00601.png]

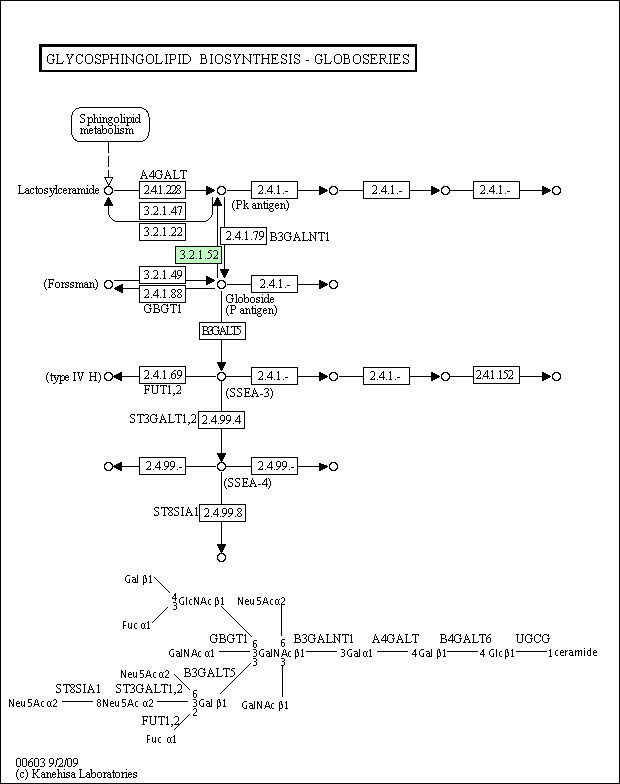

Supplement: Additional file 6 — KEGG annotation results. File contains the KEGG maps where isotigs were successfully mapped. Pathway element in green boxes indicates the components represented by at least, one isotig. Gilthead sea bream isotigs were automatically annotated to KEGG maps using KAAS website [60]. The SBH method, optimized for ESTs annotation, was used against human, chimpanzee, orang-utan, rhesus, mouse, rat, dog, giant panda, cow, pig, horse, opossum, platypus, chicken, clawed frog, zebrafish, fruit fly and nematode pathway databases. [file 1471-2164-13-181-S6.tar › map/map00603.png]

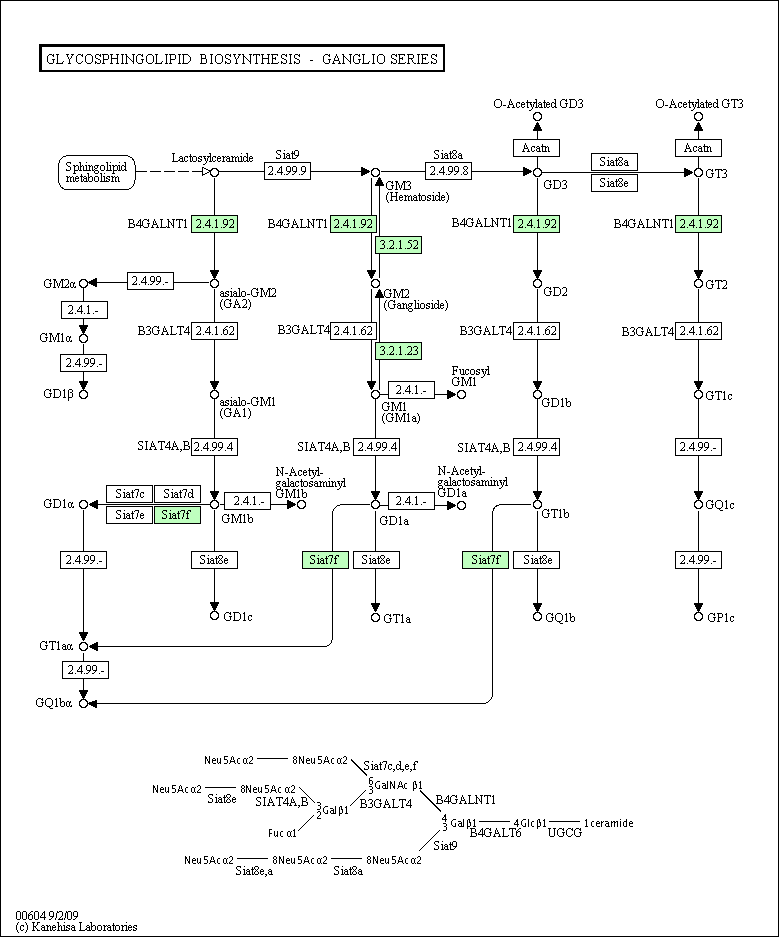

Supplement: Additional file 6 — KEGG annotation results. File contains the KEGG maps where isotigs were successfully mapped. Pathway element in green boxes indicates the components represented by at least, one isotig. Gilthead sea bream isotigs were automatically annotated to KEGG maps using KAAS website [60]. The SBH method, optimized for ESTs annotation, was used against human, chimpanzee, orang-utan, rhesus, mouse, rat, dog, giant panda, cow, pig, horse, opossum, platypus, chicken, clawed frog, zebrafish, fruit fly and nematode pathway databases. [file 1471-2164-13-181-S6.tar › map/map00604.png]

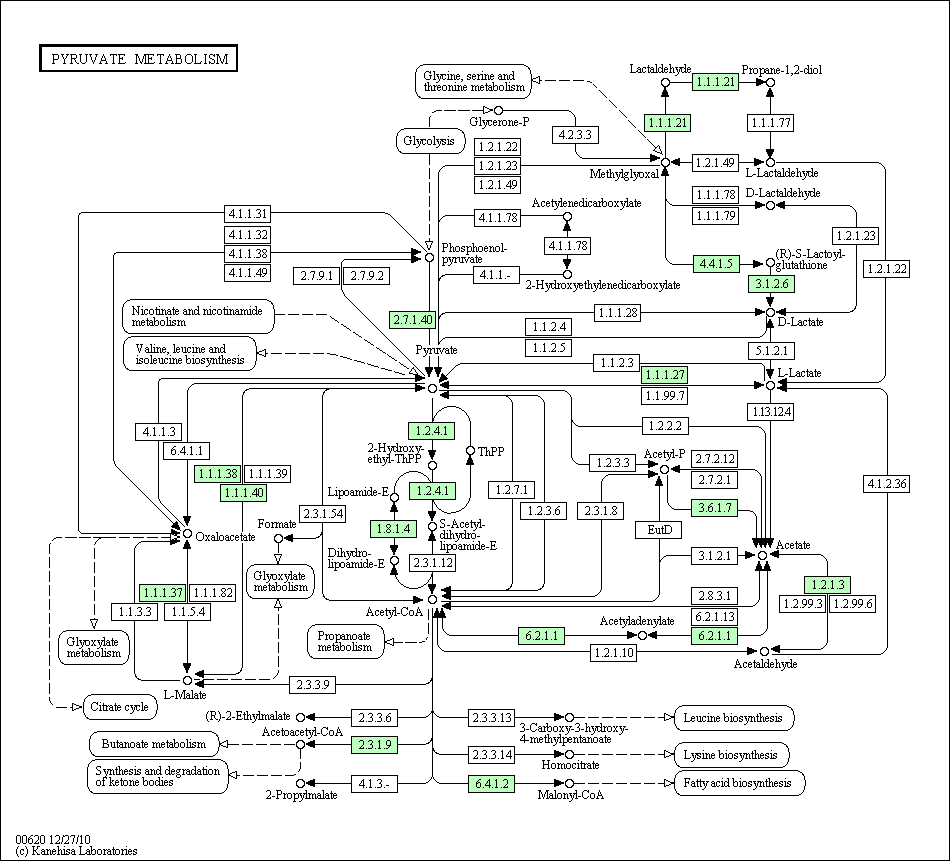

Supplement: Additional file 6 — KEGG annotation results. File contains the KEGG maps where isotigs were successfully mapped. Pathway element in green boxes indicates the components represented by at least, one isotig. Gilthead sea bream isotigs were automatically annotated to KEGG maps using KAAS website [60]. The SBH method, optimized for ESTs annotation, was used against human, chimpanzee, orang-utan, rhesus, mouse, rat, dog, giant panda, cow, pig, horse, opossum, platypus, chicken, clawed frog, zebrafish, fruit fly and nematode pathway databases. [file 1471-2164-13-181-S6.tar › map/map00620.png]

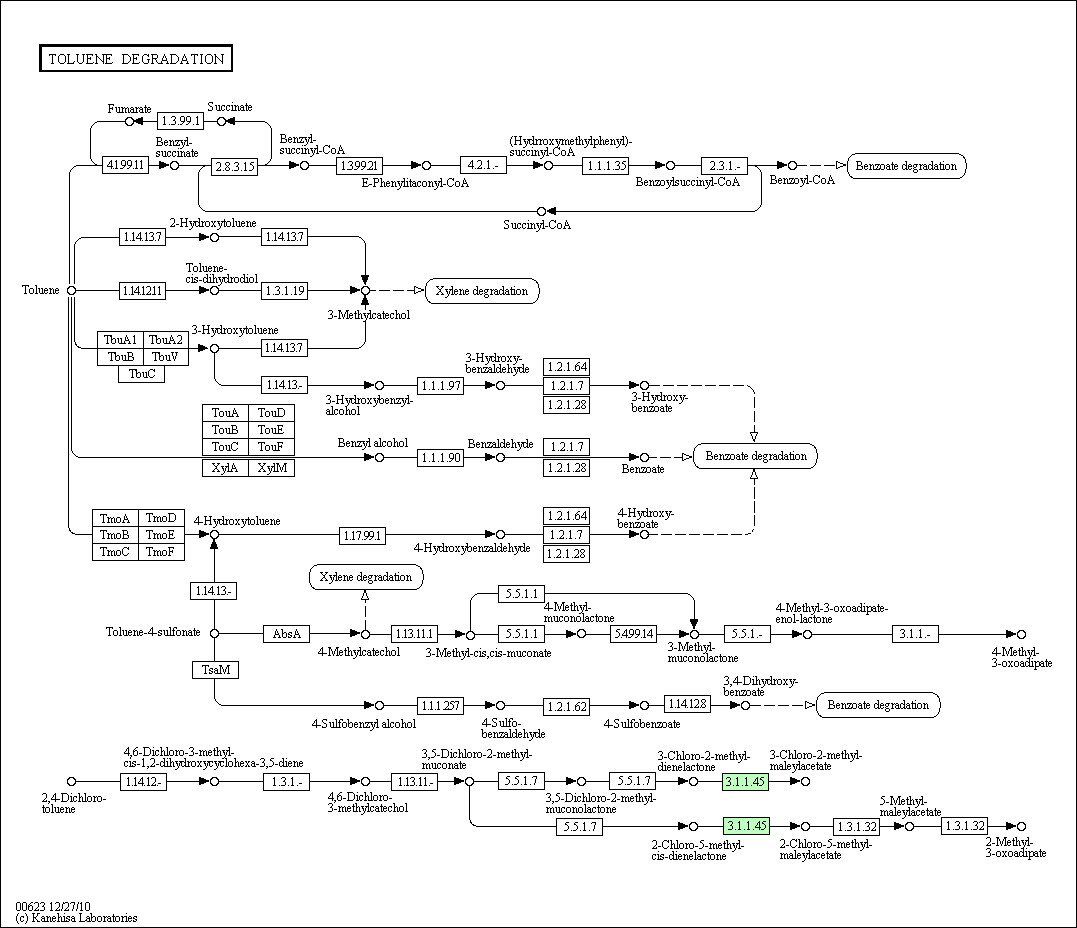

Supplement: Additional file 6 — KEGG annotation results. File contains the KEGG maps where isotigs were successfully mapped. Pathway element in green boxes indicates the components represented by at least, one isotig. Gilthead sea bream isotigs were automatically annotated to KEGG maps using KAAS website [60]. The SBH method, optimized for ESTs annotation, was used against human, chimpanzee, orang-utan, rhesus, mouse, rat, dog, giant panda, cow, pig, horse, opossum, platypus, chicken, clawed frog, zebrafish, fruit fly and nematode pathway databases. [file 1471-2164-13-181-S6.tar › map/map00623.png]

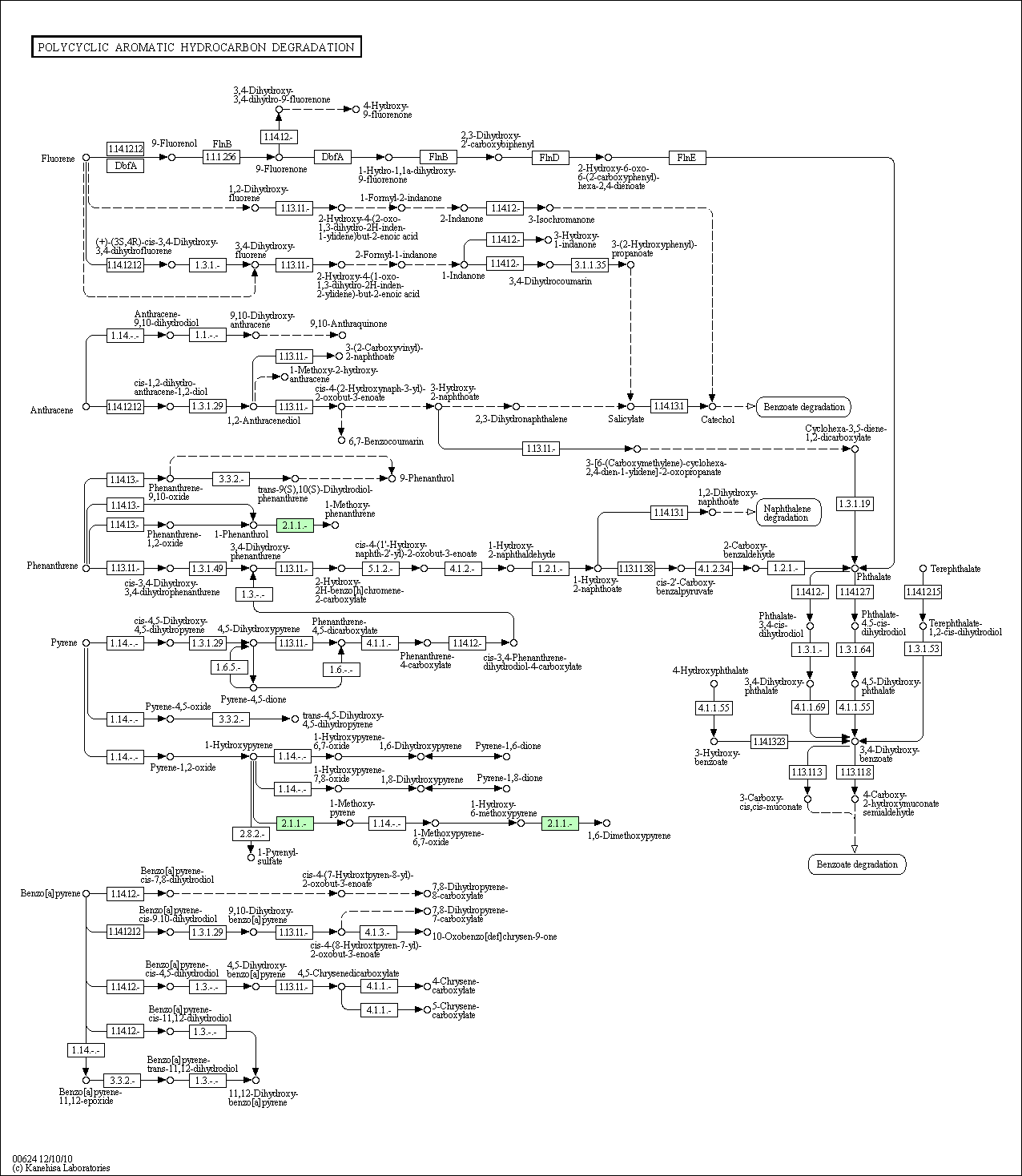

Supplement: Additional file 6 — KEGG annotation results. File contains the KEGG maps where isotigs were successfully mapped. Pathway element in green boxes indicates the components represented by at least, one isotig. Gilthead sea bream isotigs were automatically annotated to KEGG maps using KAAS website [60]. The SBH method, optimized for ESTs annotation, was used against human, chimpanzee, orang-utan, rhesus, mouse, rat, dog, giant panda, cow, pig, horse, opossum, platypus, chicken, clawed frog, zebrafish, fruit fly and nematode pathway databases. [file 1471-2164-13-181-S6.tar › map/map00624.png]

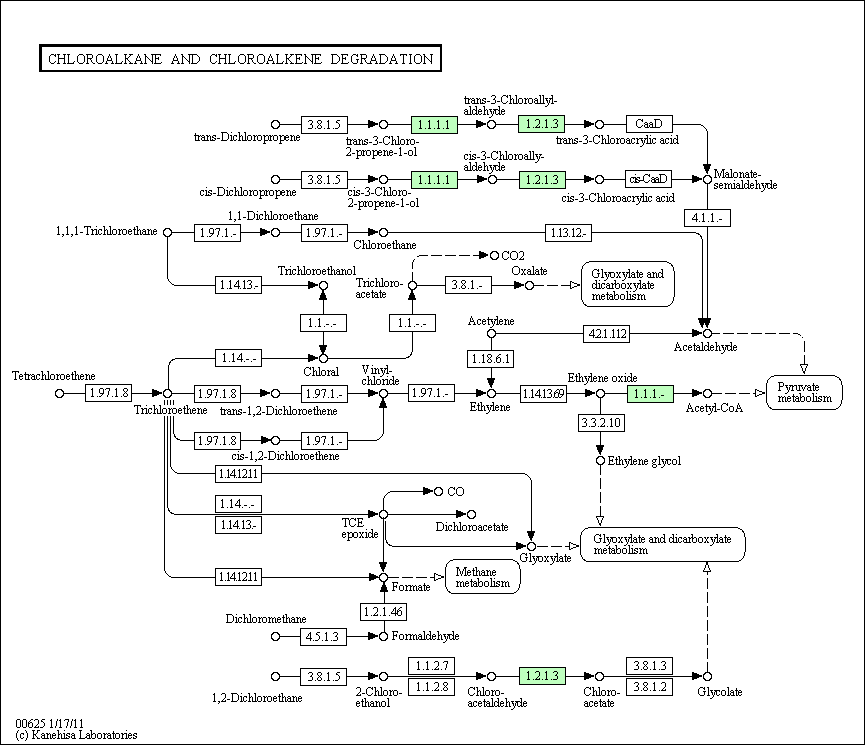

Supplement: Additional file 6 — KEGG annotation results. File contains the KEGG maps where isotigs were successfully mapped. Pathway element in green boxes indicates the components represented by at least, one isotig. Gilthead sea bream isotigs were automatically annotated to KEGG maps using KAAS website [60]. The SBH method, optimized for ESTs annotation, was used against human, chimpanzee, orang-utan, rhesus, mouse, rat, dog, giant panda, cow, pig, horse, opossum, platypus, chicken, clawed frog, zebrafish, fruit fly and nematode pathway databases. [file 1471-2164-13-181-S6.tar › map/map00625.png]

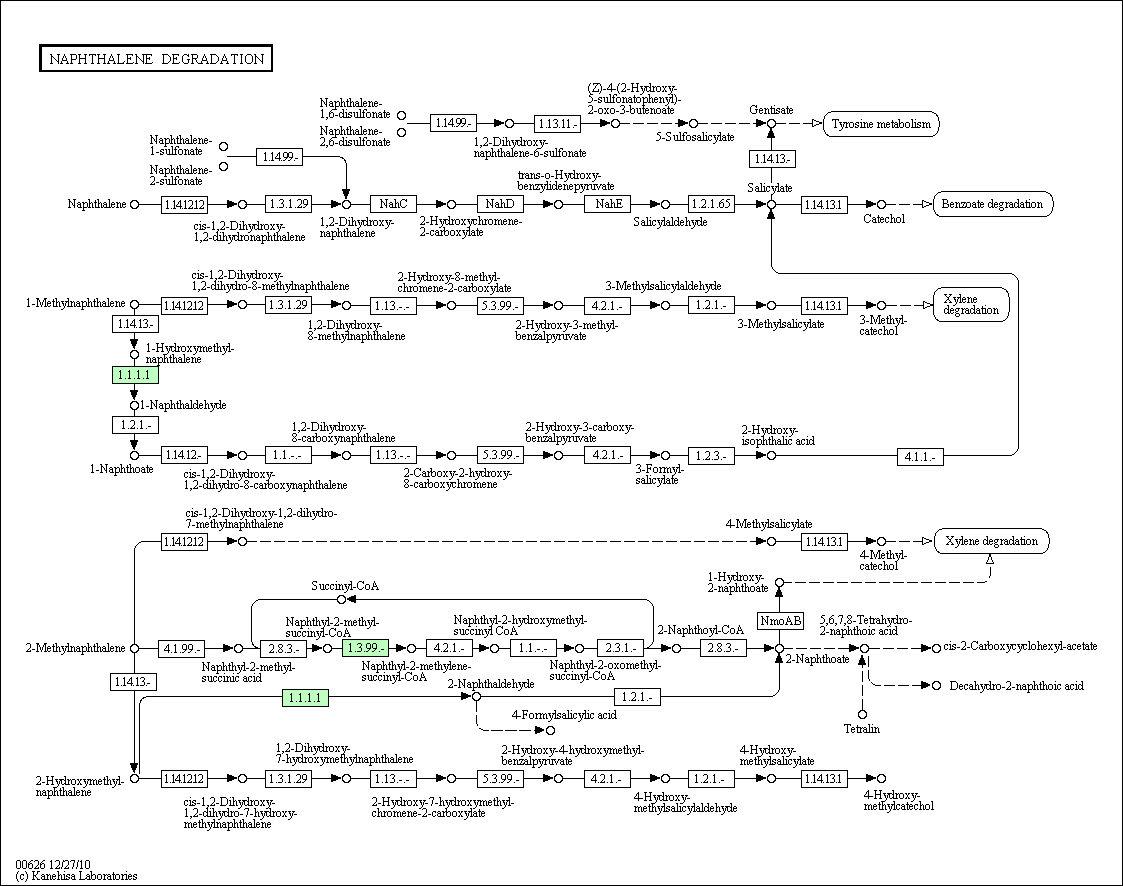

Supplement: Additional file 6 — KEGG annotation results. File contains the KEGG maps where isotigs were successfully mapped. Pathway element in green boxes indicates the components represented by at least, one isotig. Gilthead sea bream isotigs were automatically annotated to KEGG maps using KAAS website [60]. The SBH method, optimized for ESTs annotation, was used against human, chimpanzee, orang-utan, rhesus, mouse, rat, dog, giant panda, cow, pig, horse, opossum, platypus, chicken, clawed frog, zebrafish, fruit fly and nematode pathway databases. [file 1471-2164-13-181-S6.tar › map/map00626.png]

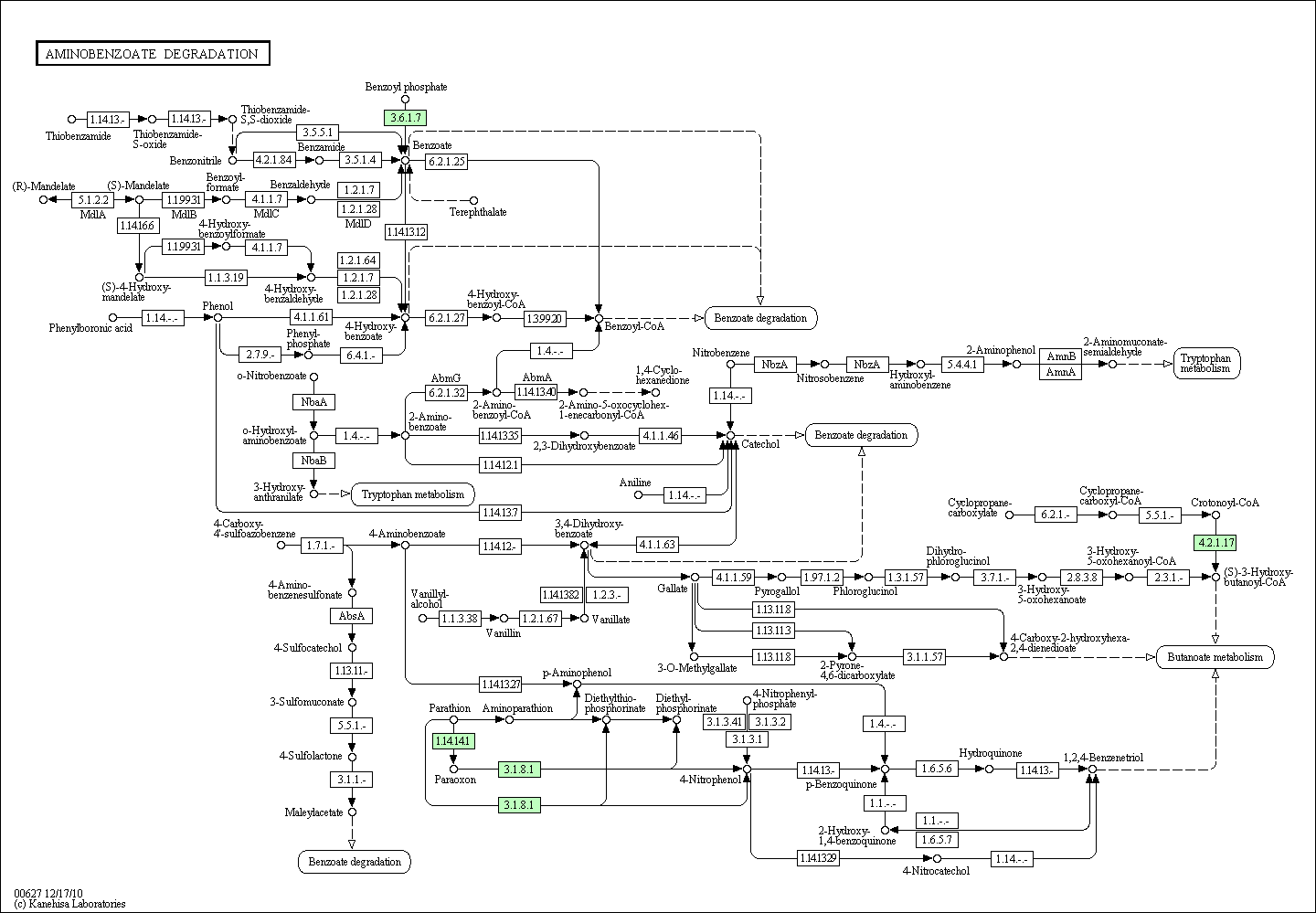

Supplement: Additional file 6 — KEGG annotation results. File contains the KEGG maps where isotigs were successfully mapped. Pathway element in green boxes indicates the components represented by at least, one isotig. Gilthead sea bream isotigs were automatically annotated to KEGG maps using KAAS website [60]. The SBH method, optimized for ESTs annotation, was used against human, chimpanzee, orang-utan, rhesus, mouse, rat, dog, giant panda, cow, pig, horse, opossum, platypus, chicken, clawed frog, zebrafish, fruit fly and nematode pathway databases. [file 1471-2164-13-181-S6.tar › map/map00627.png]

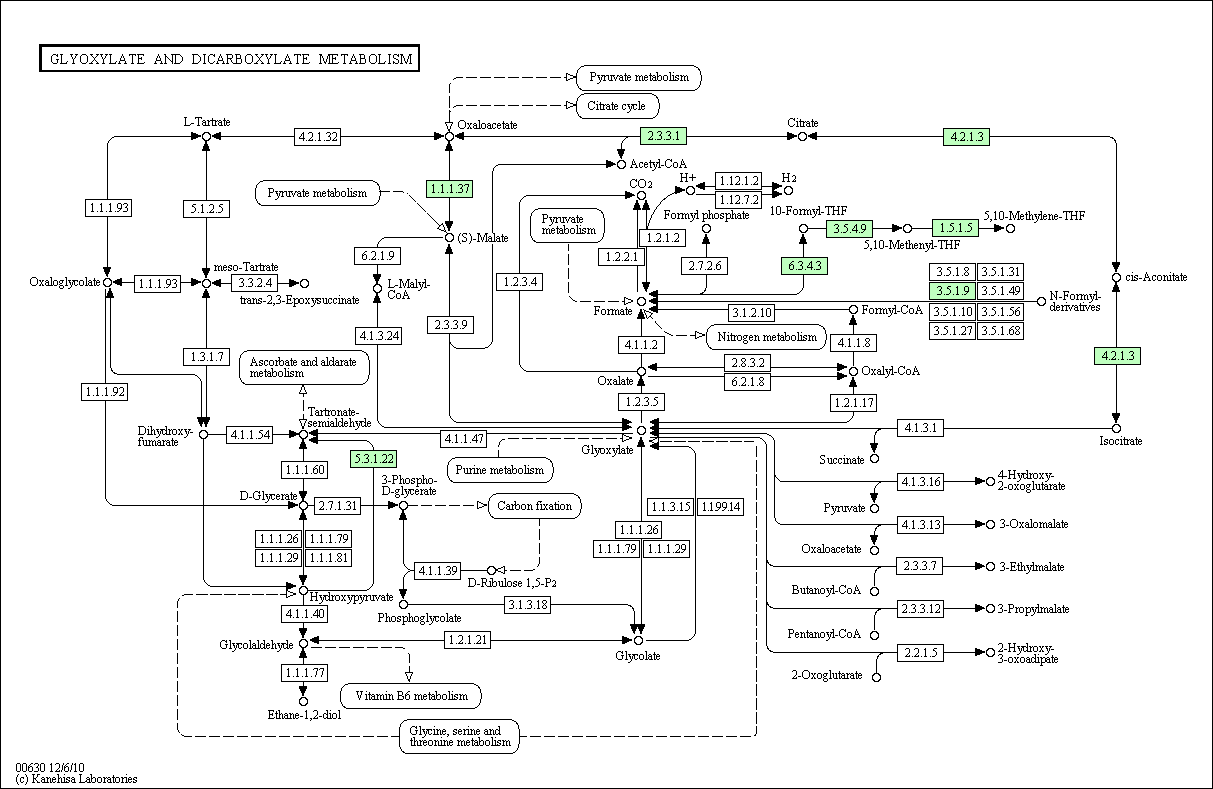

Supplement: Additional file 6 — KEGG annotation results. File contains the KEGG maps where isotigs were successfully mapped. Pathway element in green boxes indicates the components represented by at least, one isotig. Gilthead sea bream isotigs were automatically annotated to KEGG maps using KAAS website [60]. The SBH method, optimized for ESTs annotation, was used against human, chimpanzee, orang-utan, rhesus, mouse, rat, dog, giant panda, cow, pig, horse, opossum, platypus, chicken, clawed frog, zebrafish, fruit fly and nematode pathway databases. [file 1471-2164-13-181-S6.tar › map/map00630.png]

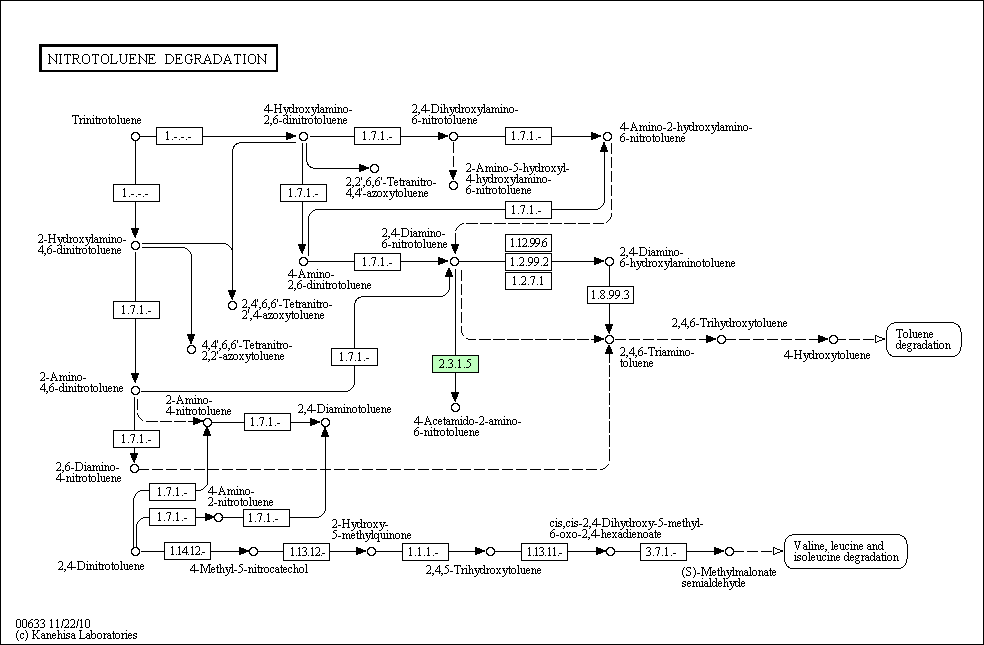

Supplement: Additional file 6 — KEGG annotation results. File contains the KEGG maps where isotigs were successfully mapped. Pathway element in green boxes indicates the components represented by at least, one isotig. Gilthead sea bream isotigs were automatically annotated to KEGG maps using KAAS website [60]. The SBH method, optimized for ESTs annotation, was used against human, chimpanzee, orang-utan, rhesus, mouse, rat, dog, giant panda, cow, pig, horse, opossum, platypus, chicken, clawed frog, zebrafish, fruit fly and nematode pathway databases. [file 1471-2164-13-181-S6.tar › map/map00633.png]

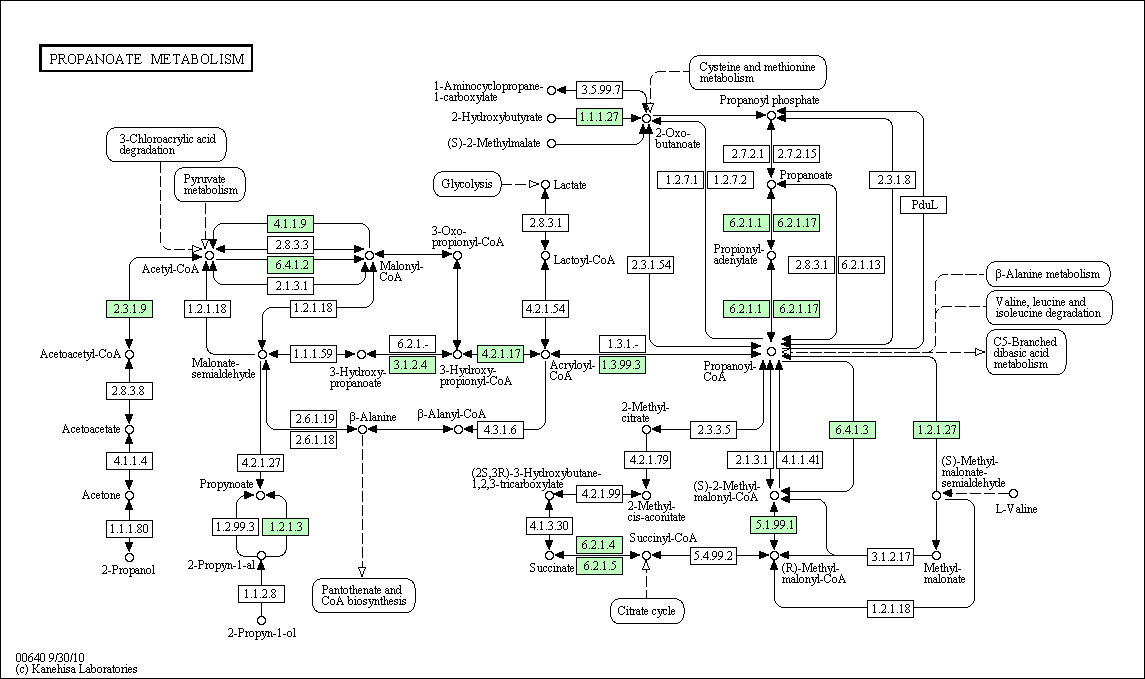

Supplement: Additional file 6 — KEGG annotation results. File contains the KEGG maps where isotigs were successfully mapped. Pathway element in green boxes indicates the components represented by at least, one isotig. Gilthead sea bream isotigs were automatically annotated to KEGG maps using KAAS website [60]. The SBH method, optimized for ESTs annotation, was used against human, chimpanzee, orang-utan, rhesus, mouse, rat, dog, giant panda, cow, pig, horse, opossum, platypus, chicken, clawed frog, zebrafish, fruit fly and nematode pathway databases. [file 1471-2164-13-181-S6.tar › map/map00640.png]

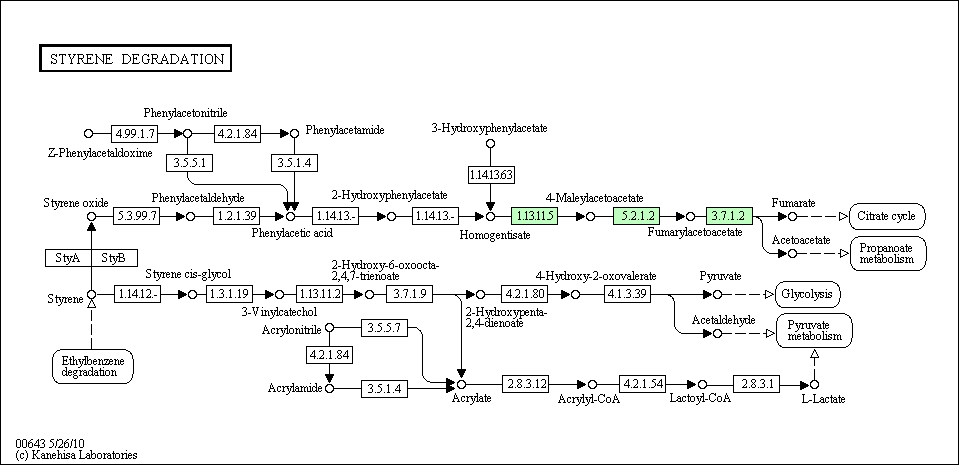

Supplement: Additional file 6 — KEGG annotation results. File contains the KEGG maps where isotigs were successfully mapped. Pathway element in green boxes indicates the components represented by at least, one isotig. Gilthead sea bream isotigs were automatically annotated to KEGG maps using KAAS website [60]. The SBH method, optimized for ESTs annotation, was used against human, chimpanzee, orang-utan, rhesus, mouse, rat, dog, giant panda, cow, pig, horse, opossum, platypus, chicken, clawed frog, zebrafish, fruit fly and nematode pathway databases. [file 1471-2164-13-181-S6.tar › map/map00643.png]

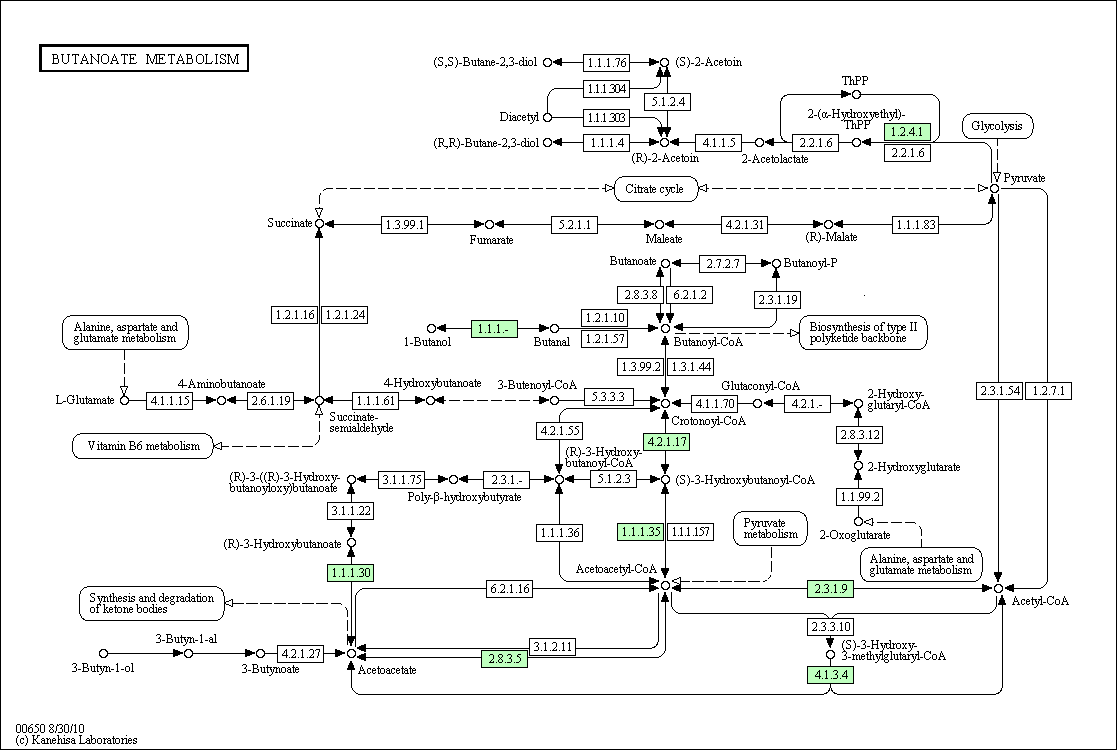

Supplement: Additional file 6 — KEGG annotation results. File contains the KEGG maps where isotigs were successfully mapped. Pathway element in green boxes indicates the components represented by at least, one isotig. Gilthead sea bream isotigs were automatically annotated to KEGG maps using KAAS website [60]. The SBH method, optimized for ESTs annotation, was used against human, chimpanzee, orang-utan, rhesus, mouse, rat, dog, giant panda, cow, pig, horse, opossum, platypus, chicken, clawed frog, zebrafish, fruit fly and nematode pathway databases. [file 1471-2164-13-181-S6.tar › map/map00650.png]

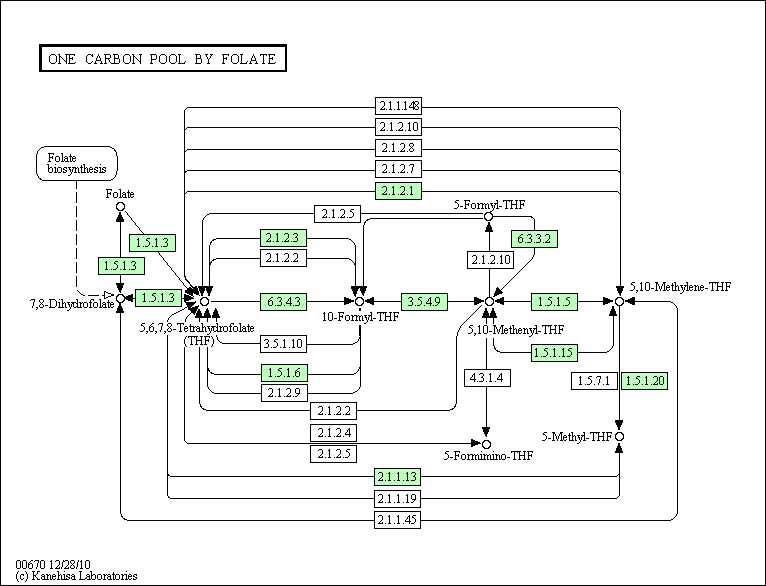

Supplement: Additional file 6 — KEGG annotation results. File contains the KEGG maps where isotigs were successfully mapped. Pathway element in green boxes indicates the components represented by at least, one isotig. Gilthead sea bream isotigs were automatically annotated to KEGG maps using KAAS website [60]. The SBH method, optimized for ESTs annotation, was used against human, chimpanzee, orang-utan, rhesus, mouse, rat, dog, giant panda, cow, pig, horse, opossum, platypus, chicken, clawed frog, zebrafish, fruit fly and nematode pathway databases. [file 1471-2164-13-181-S6.tar › map/map00670.png]

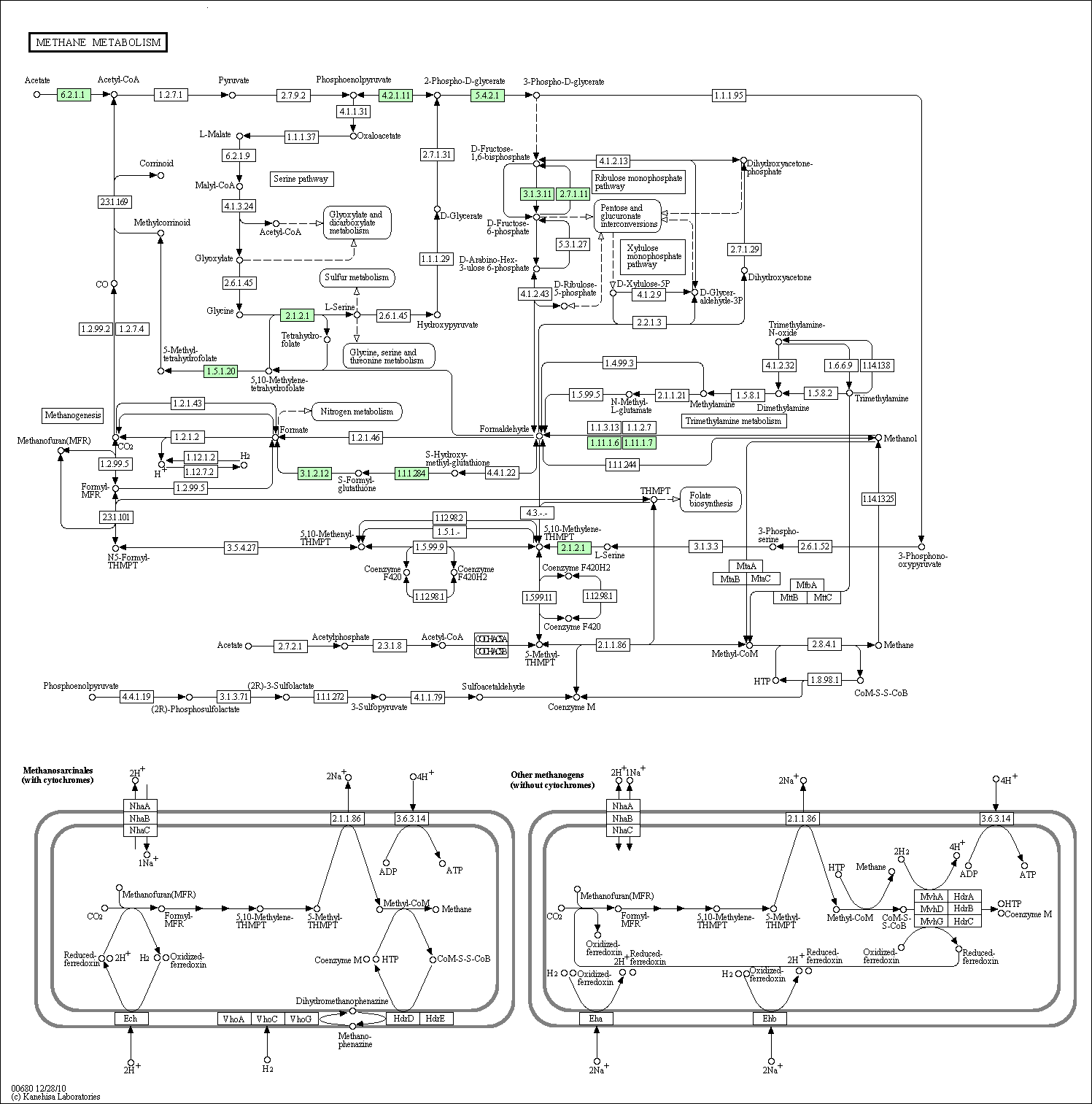

Supplement: Additional file 6 — KEGG annotation results. File contains the KEGG maps where isotigs were successfully mapped. Pathway element in green boxes indicates the components represented by at least, one isotig. Gilthead sea bream isotigs were automatically annotated to KEGG maps using KAAS website [60]. The SBH method, optimized for ESTs annotation, was used against human, chimpanzee, orang-utan, rhesus, mouse, rat, dog, giant panda, cow, pig, horse, opossum, platypus, chicken, clawed frog, zebrafish, fruit fly and nematode pathway databases. [file 1471-2164-13-181-S6.tar › map/map00680.png]

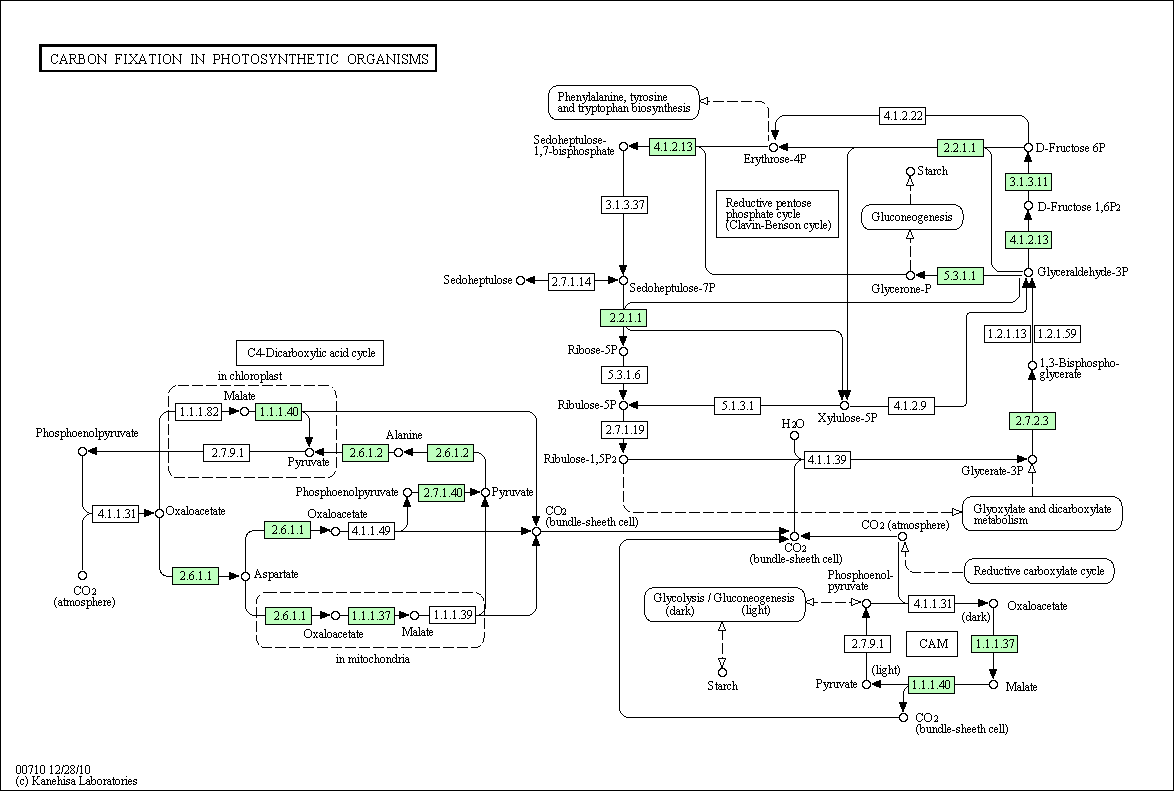

Supplement: Additional file 6 — KEGG annotation results. File contains the KEGG maps where isotigs were successfully mapped. Pathway element in green boxes indicates the components represented by at least, one isotig. Gilthead sea bream isotigs were automatically annotated to KEGG maps using KAAS website [60]. The SBH method, optimized for ESTs annotation, was used against human, chimpanzee, orang-utan, rhesus, mouse, rat, dog, giant panda, cow, pig, horse, opossum, platypus, chicken, clawed frog, zebrafish, fruit fly and nematode pathway databases. [file 1471-2164-13-181-S6.tar › map/map00710.png]

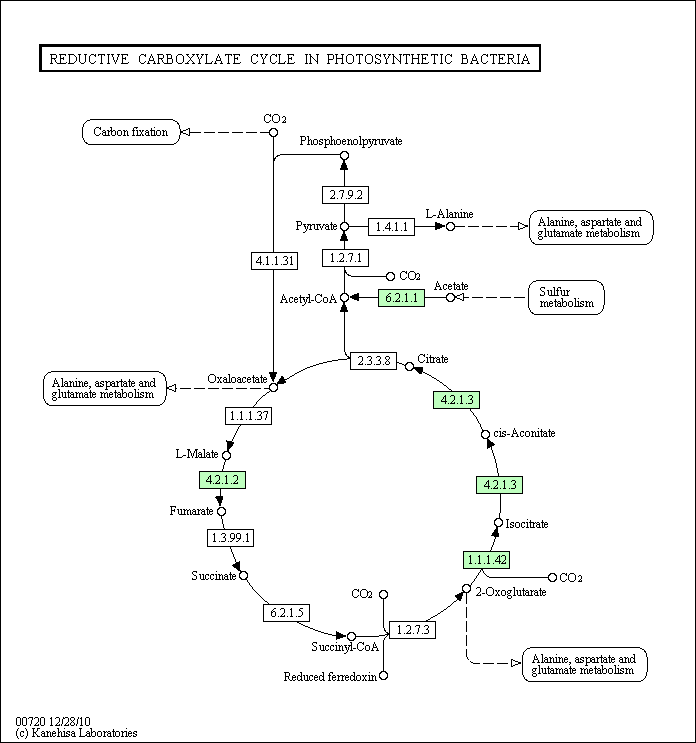

Supplement: Additional file 6 — KEGG annotation results. File contains the KEGG maps where isotigs were successfully mapped. Pathway element in green boxes indicates the components represented by at least, one isotig. Gilthead sea bream isotigs were automatically annotated to KEGG maps using KAAS website [60]. The SBH method, optimized for ESTs annotation, was used against human, chimpanzee, orang-utan, rhesus, mouse, rat, dog, giant panda, cow, pig, horse, opossum, platypus, chicken, clawed frog, zebrafish, fruit fly and nematode pathway databases. [file 1471-2164-13-181-S6.tar › map/map00720.png]

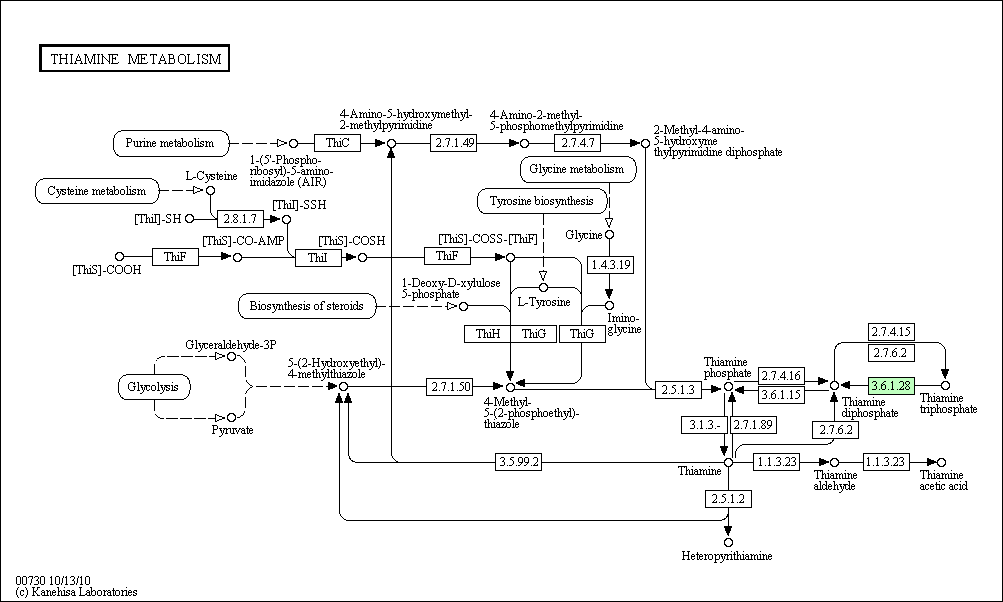

Supplement: Additional file 6 — KEGG annotation results. File contains the KEGG maps where isotigs were successfully mapped. Pathway element in green boxes indicates the components represented by at least, one isotig. Gilthead sea bream isotigs were automatically annotated to KEGG maps using KAAS website [60]. The SBH method, optimized for ESTs annotation, was used against human, chimpanzee, orang-utan, rhesus, mouse, rat, dog, giant panda, cow, pig, horse, opossum, platypus, chicken, clawed frog, zebrafish, fruit fly and nematode pathway databases. [file 1471-2164-13-181-S6.tar › map/map00730.png]

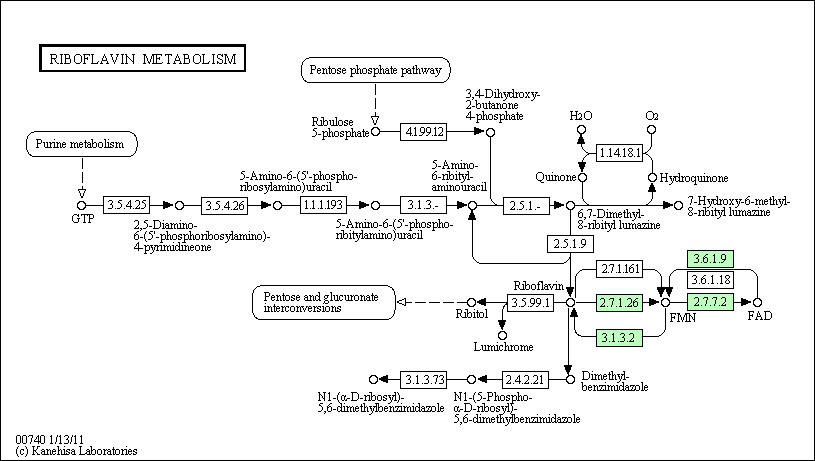

Supplement: Additional file 6 — KEGG annotation results. File contains the KEGG maps where isotigs were successfully mapped. Pathway element in green boxes indicates the components represented by at least, one isotig. Gilthead sea bream isotigs were automatically annotated to KEGG maps using KAAS website [60]. The SBH method, optimized for ESTs annotation, was used against human, chimpanzee, orang-utan, rhesus, mouse, rat, dog, giant panda, cow, pig, horse, opossum, platypus, chicken, clawed frog, zebrafish, fruit fly and nematode pathway databases. [file 1471-2164-13-181-S6.tar › map/map00740.png]

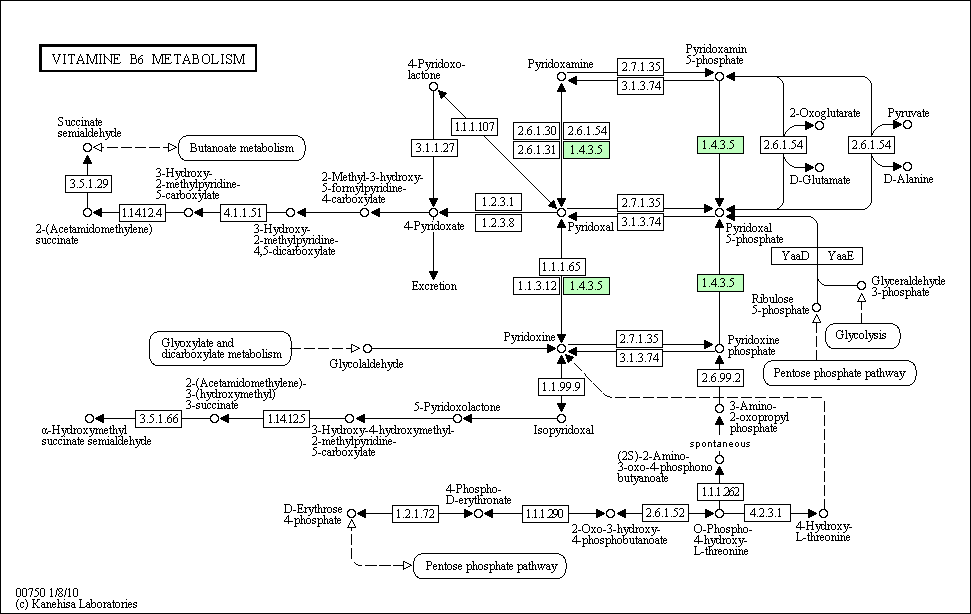

Supplement: Additional file 6 — KEGG annotation results. File contains the KEGG maps where isotigs were successfully mapped. Pathway element in green boxes indicates the components represented by at least, one isotig. Gilthead sea bream isotigs were automatically annotated to KEGG maps using KAAS website [60]. The SBH method, optimized for ESTs annotation, was used against human, chimpanzee, orang-utan, rhesus, mouse, rat, dog, giant panda, cow, pig, horse, opossum, platypus, chicken, clawed frog, zebrafish, fruit fly and nematode pathway databases. [file 1471-2164-13-181-S6.tar › map/map00750.png]

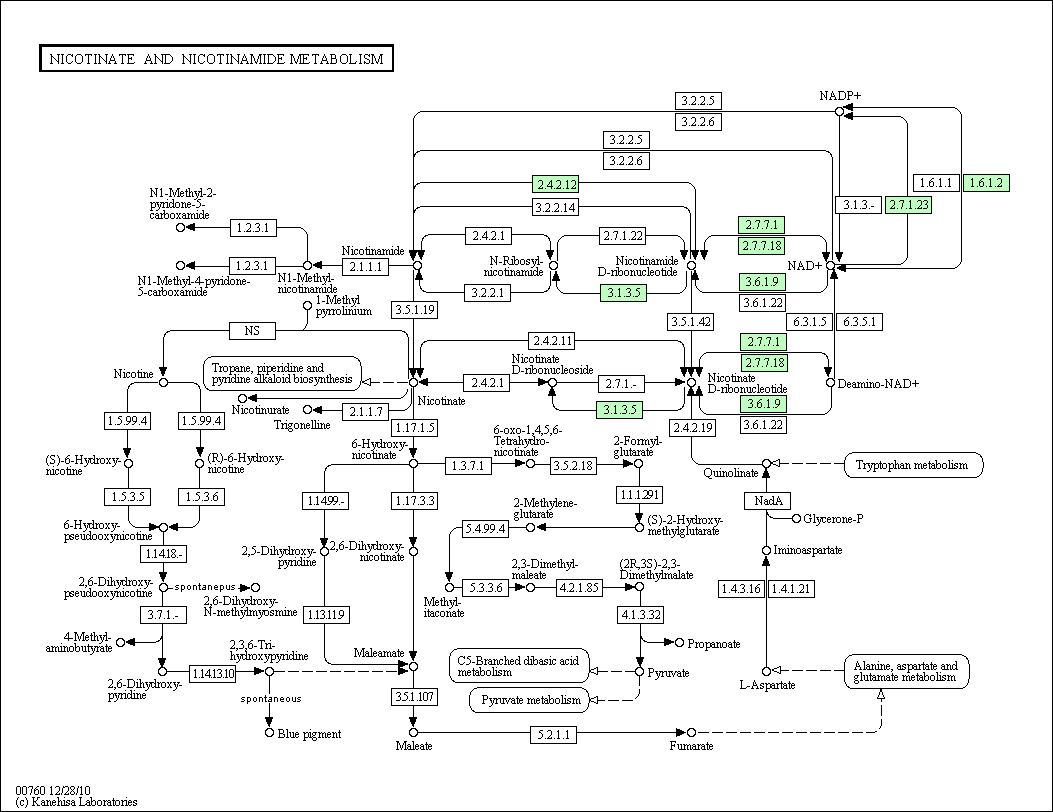

Supplement: Additional file 6 — KEGG annotation results. File contains the KEGG maps where isotigs were successfully mapped. Pathway element in green boxes indicates the components represented by at least, one isotig. Gilthead sea bream isotigs were automatically annotated to KEGG maps using KAAS website [60]. The SBH method, optimized for ESTs annotation, was used against human, chimpanzee, orang-utan, rhesus, mouse, rat, dog, giant panda, cow, pig, horse, opossum, platypus, chicken, clawed frog, zebrafish, fruit fly and nematode pathway databases. [file 1471-2164-13-181-S6.tar › map/map00760.png]

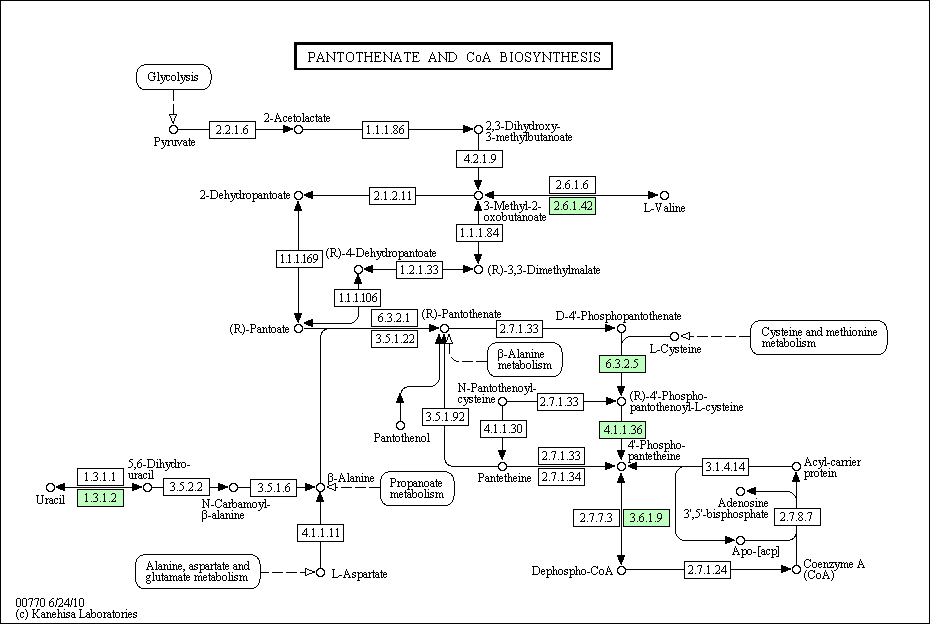

Supplement: Additional file 6 — KEGG annotation results. File contains the KEGG maps where isotigs were successfully mapped. Pathway element in green boxes indicates the components represented by at least, one isotig. Gilthead sea bream isotigs were automatically annotated to KEGG maps using KAAS website [60]. The SBH method, optimized for ESTs annotation, was used against human, chimpanzee, orang-utan, rhesus, mouse, rat, dog, giant panda, cow, pig, horse, opossum, platypus, chicken, clawed frog, zebrafish, fruit fly and nematode pathway databases. [file 1471-2164-13-181-S6.tar › map/map00770.png]

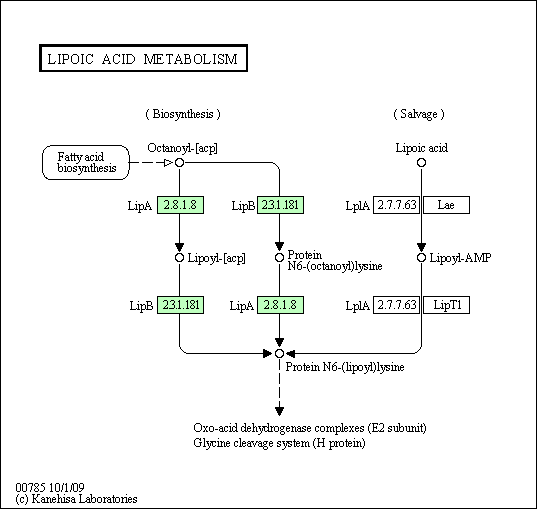

Supplement: Additional file 6 — KEGG annotation results. File contains the KEGG maps where isotigs were successfully mapped. Pathway element in green boxes indicates the components represented by at least, one isotig. Gilthead sea bream isotigs were automatically annotated to KEGG maps using KAAS website [60]. The SBH method, optimized for ESTs annotation, was used against human, chimpanzee, orang-utan, rhesus, mouse, rat, dog, giant panda, cow, pig, horse, opossum, platypus, chicken, clawed frog, zebrafish, fruit fly and nematode pathway databases. [file 1471-2164-13-181-S6.tar › map/map00785.png]

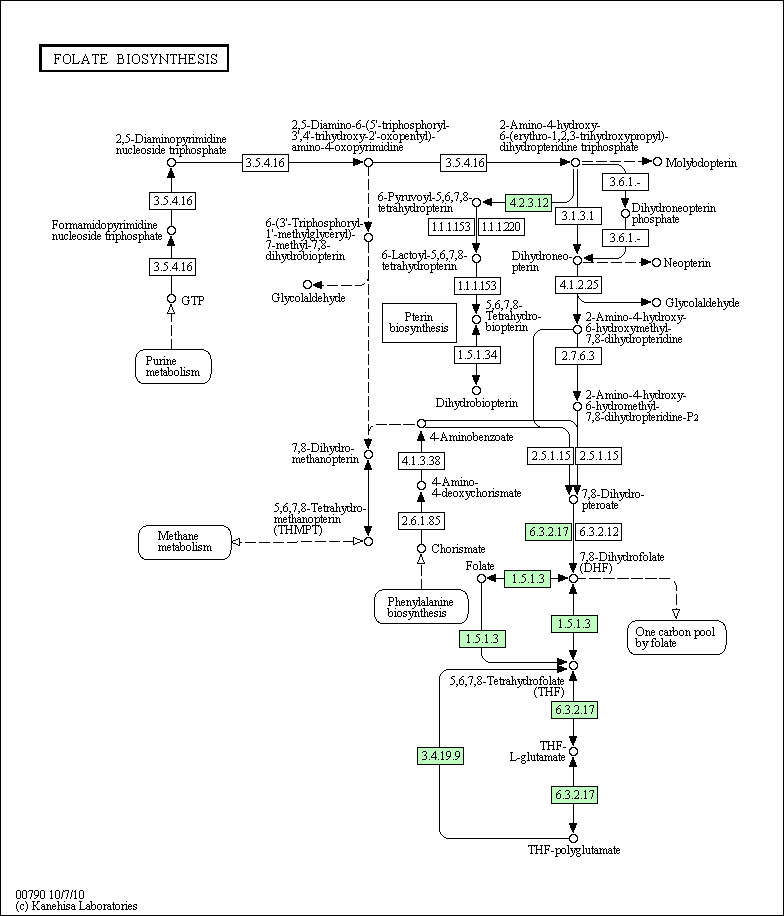

Supplement: Additional file 6 — KEGG annotation results. File contains the KEGG maps where isotigs were successfully mapped. Pathway element in green boxes indicates the components represented by at least, one isotig. Gilthead sea bream isotigs were automatically annotated to KEGG maps using KAAS website [60]. The SBH method, optimized for ESTs annotation, was used against human, chimpanzee, orang-utan, rhesus, mouse, rat, dog, giant panda, cow, pig, horse, opossum, platypus, chicken, clawed frog, zebrafish, fruit fly and nematode pathway databases. [file 1471-2164-13-181-S6.tar › map/map00790.png]

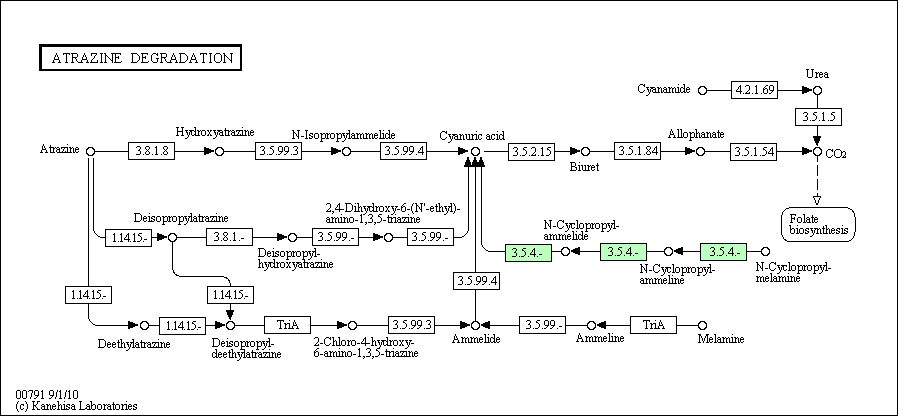

Supplement: Additional file 6 — KEGG annotation results. File contains the KEGG maps where isotigs were successfully mapped. Pathway element in green boxes indicates the components represented by at least, one isotig. Gilthead sea bream isotigs were automatically annotated to KEGG maps using KAAS website [60]. The SBH method, optimized for ESTs annotation, was used against human, chimpanzee, orang-utan, rhesus, mouse, rat, dog, giant panda, cow, pig, horse, opossum, platypus, chicken, clawed frog, zebrafish, fruit fly and nematode pathway databases. [file 1471-2164-13-181-S6.tar › map/map00791.png]

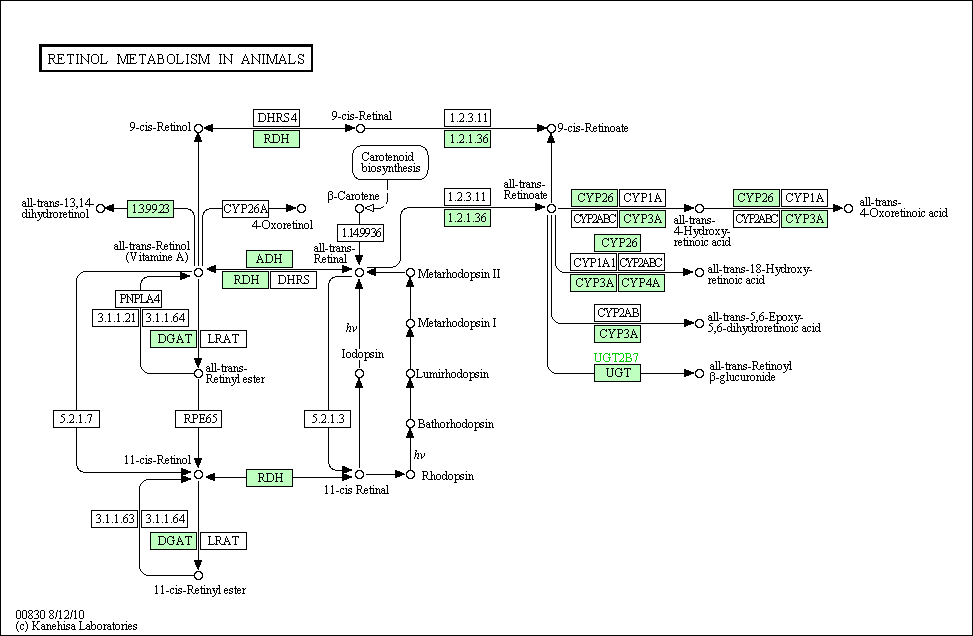

Supplement: Additional file 6 — KEGG annotation results. File contains the KEGG maps where isotigs were successfully mapped. Pathway element in green boxes indicates the components represented by at least, one isotig. Gilthead sea bream isotigs were automatically annotated to KEGG maps using KAAS website [60]. The SBH method, optimized for ESTs annotation, was used against human, chimpanzee, orang-utan, rhesus, mouse, rat, dog, giant panda, cow, pig, horse, opossum, platypus, chicken, clawed frog, zebrafish, fruit fly and nematode pathway databases. [file 1471-2164-13-181-S6.tar › map/map00830.png]

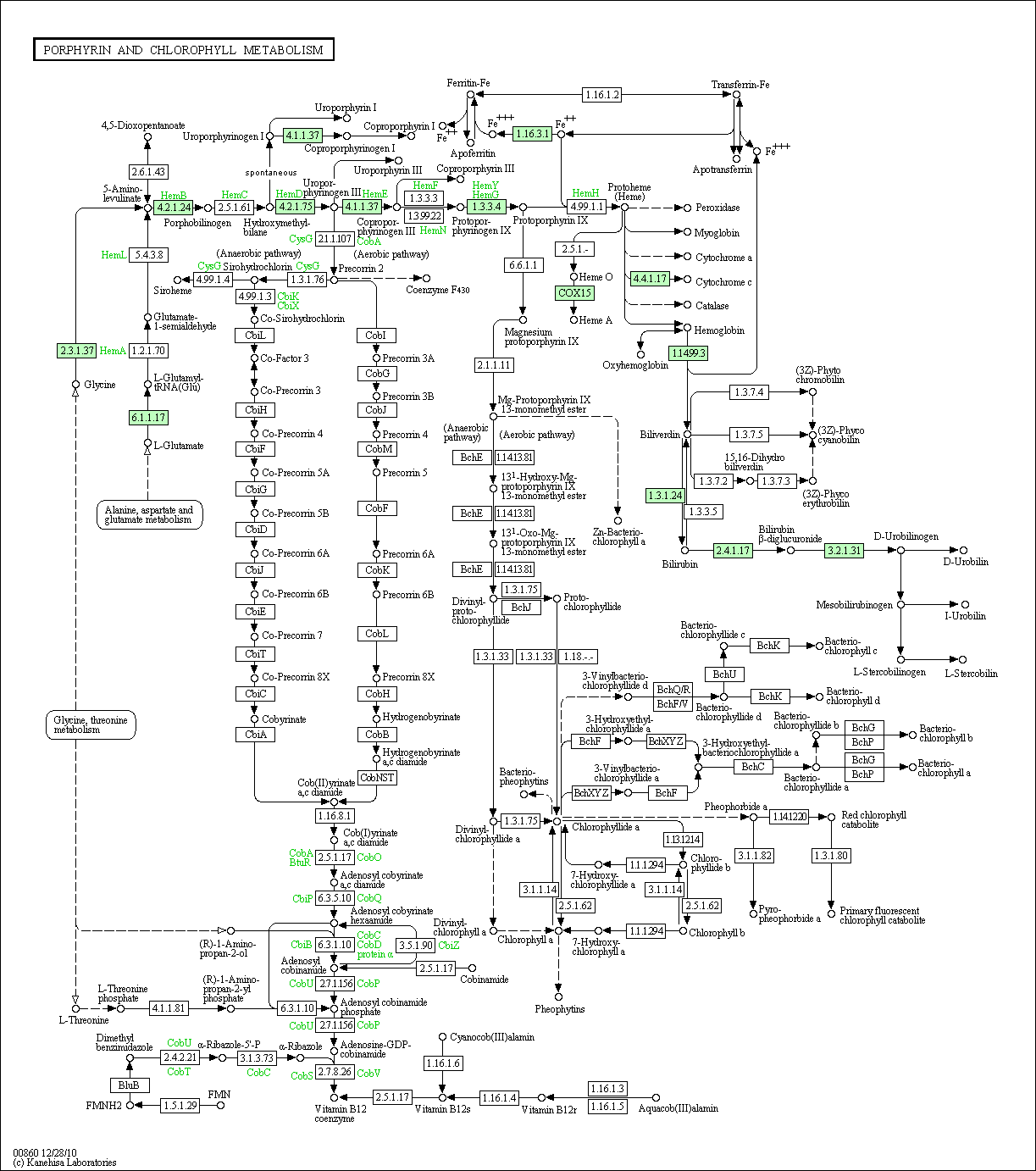

Supplement: Additional file 6 — KEGG annotation results. File contains the KEGG maps where isotigs were successfully mapped. Pathway element in green boxes indicates the components represented by at least, one isotig. Gilthead sea bream isotigs were automatically annotated to KEGG maps using KAAS website [60]. The SBH method, optimized for ESTs annotation, was used against human, chimpanzee, orang-utan, rhesus, mouse, rat, dog, giant panda, cow, pig, horse, opossum, platypus, chicken, clawed frog, zebrafish, fruit fly and nematode pathway databases. [file 1471-2164-13-181-S6.tar › map/map00860.png]

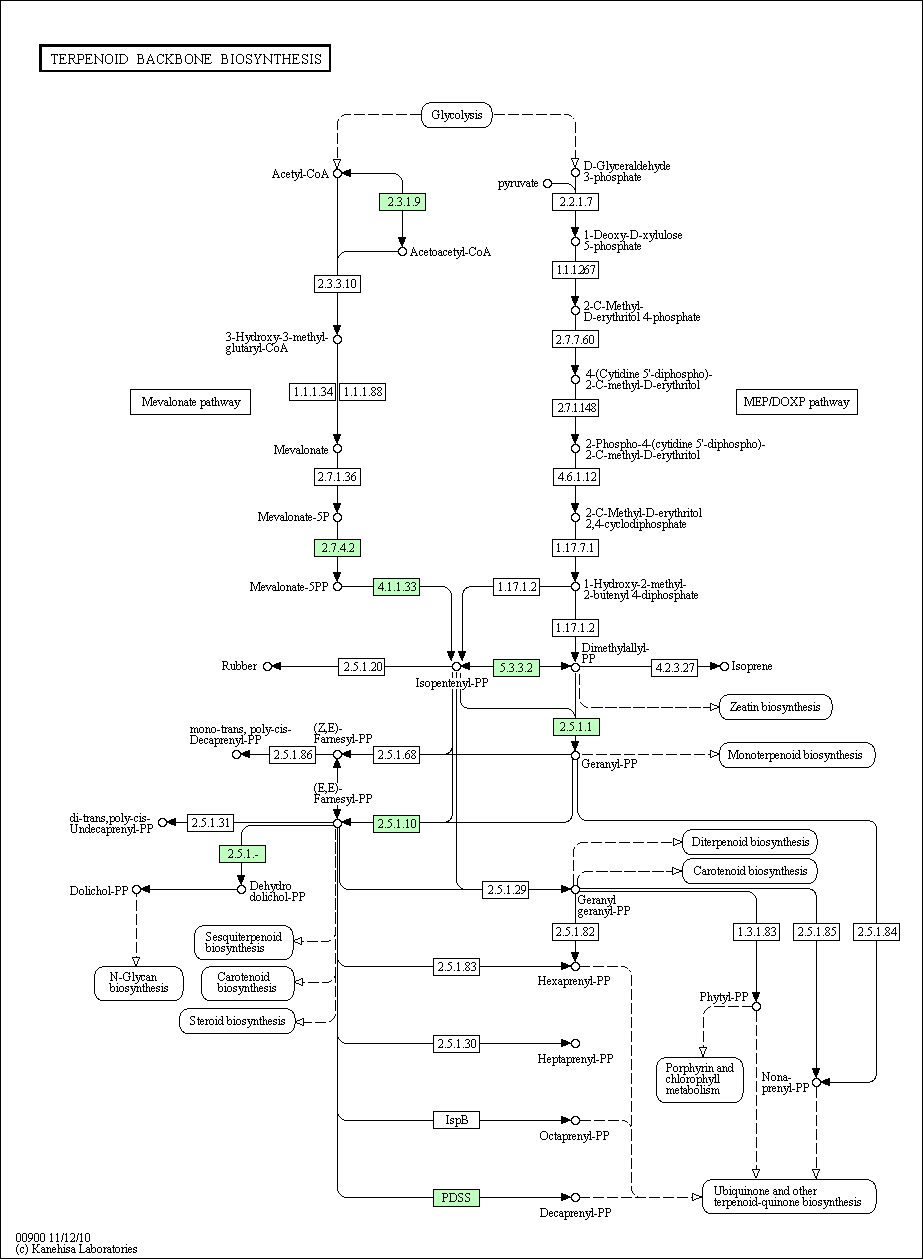

Supplement: Additional file 6 — KEGG annotation results. File contains the KEGG maps where isotigs were successfully mapped. Pathway element in green boxes indicates the components represented by at least, one isotig. Gilthead sea bream isotigs were automatically annotated to KEGG maps using KAAS website [60]. The SBH method, optimized for ESTs annotation, was used against human, chimpanzee, orang-utan, rhesus, mouse, rat, dog, giant panda, cow, pig, horse, opossum, platypus, chicken, clawed frog, zebrafish, fruit fly and nematode pathway databases. [file 1471-2164-13-181-S6.tar › map/map00900.png]

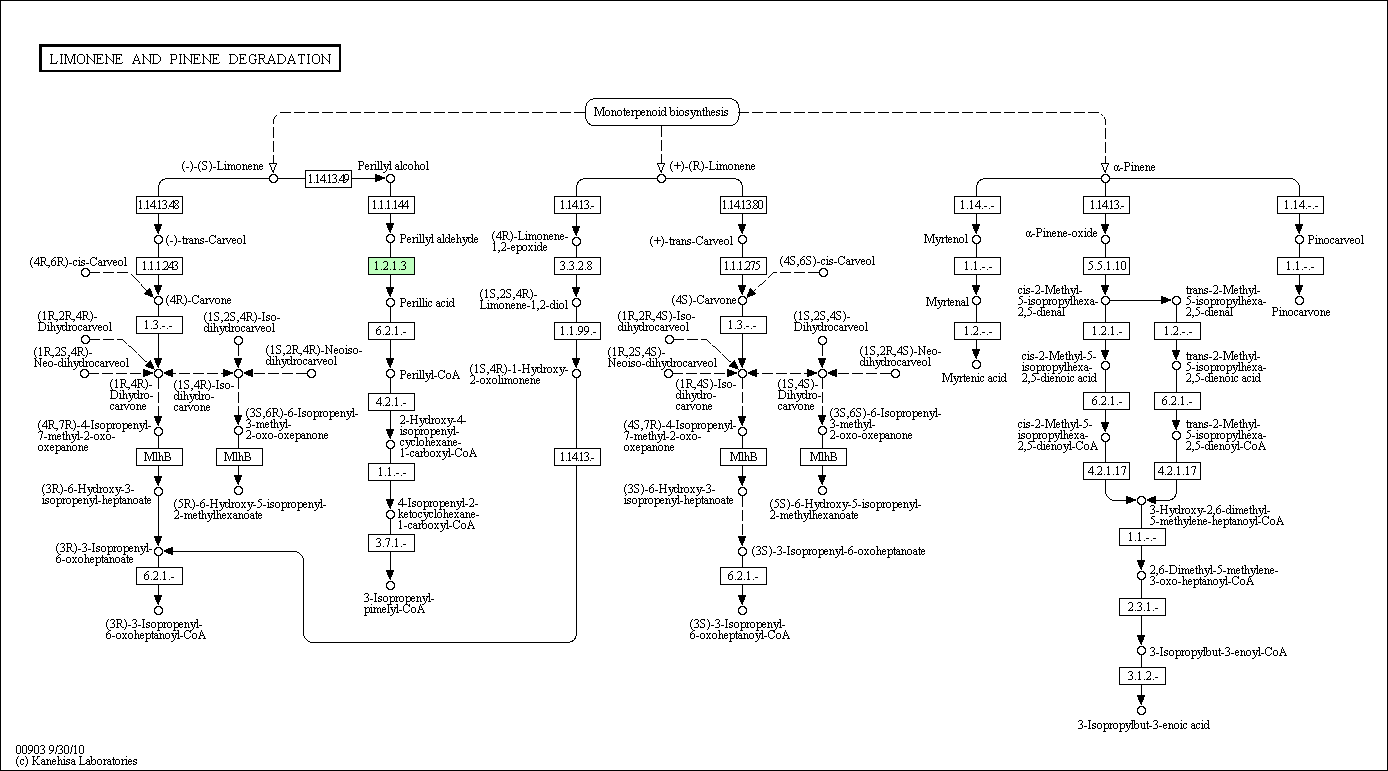

Supplement: Additional file 6 — KEGG annotation results. File contains the KEGG maps where isotigs were successfully mapped. Pathway element in green boxes indicates the components represented by at least, one isotig. Gilthead sea bream isotigs were automatically annotated to KEGG maps using KAAS website [60]. The SBH method, optimized for ESTs annotation, was used against human, chimpanzee, orang-utan, rhesus, mouse, rat, dog, giant panda, cow, pig, horse, opossum, platypus, chicken, clawed frog, zebrafish, fruit fly and nematode pathway databases. [file 1471-2164-13-181-S6.tar › map/map00903.png]

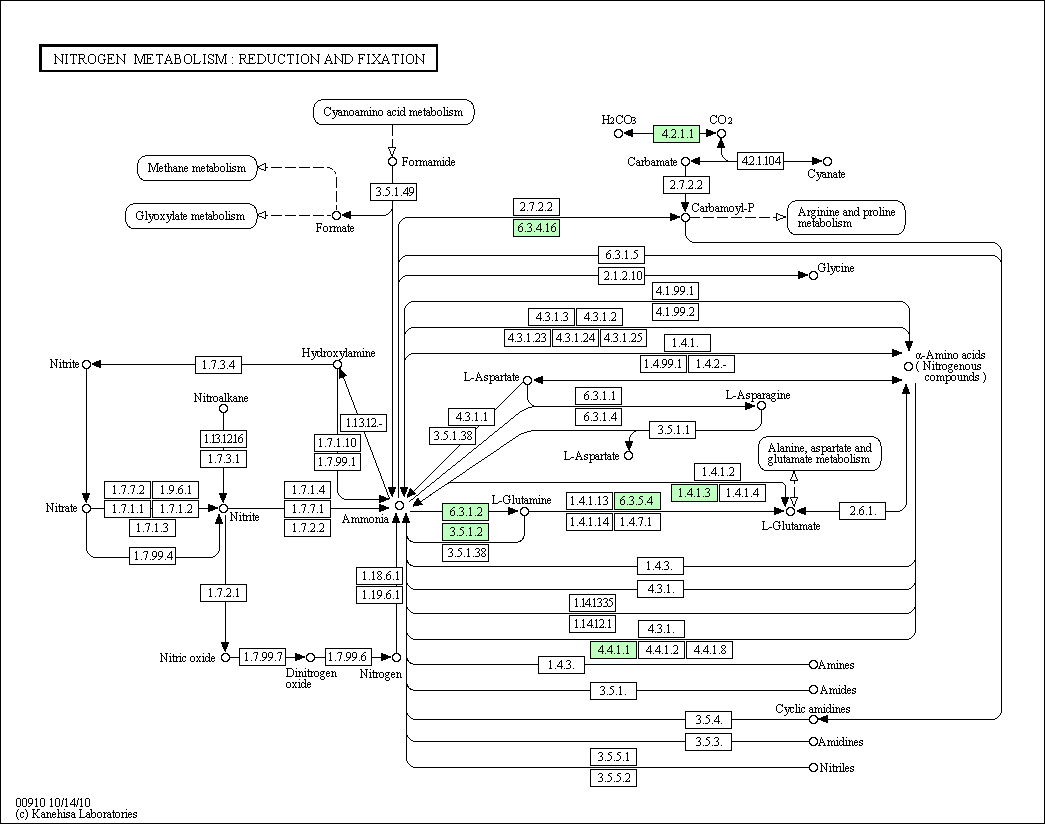

Supplement: Additional file 6 — KEGG annotation results. File contains the KEGG maps where isotigs were successfully mapped. Pathway element in green boxes indicates the components represented by at least, one isotig. Gilthead sea bream isotigs were automatically annotated to KEGG maps using KAAS website [60]. The SBH method, optimized for ESTs annotation, was used against human, chimpanzee, orang-utan, rhesus, mouse, rat, dog, giant panda, cow, pig, horse, opossum, platypus, chicken, clawed frog, zebrafish, fruit fly and nematode pathway databases. [file 1471-2164-13-181-S6.tar › map/map00910.png]

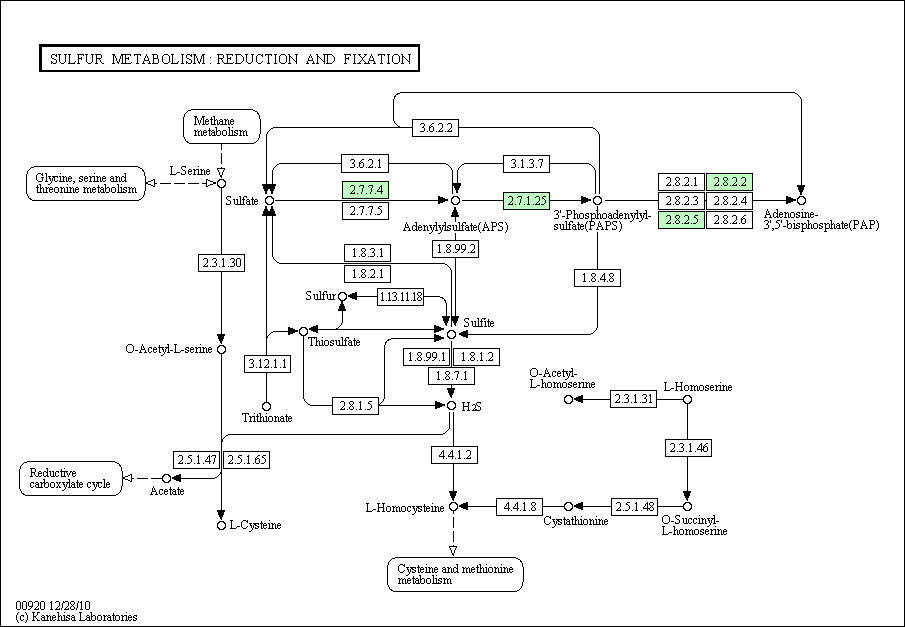

Supplement: Additional file 6 — KEGG annotation results. File contains the KEGG maps where isotigs were successfully mapped. Pathway element in green boxes indicates the components represented by at least, one isotig. Gilthead sea bream isotigs were automatically annotated to KEGG maps using KAAS website [60]. The SBH method, optimized for ESTs annotation, was used against human, chimpanzee, orang-utan, rhesus, mouse, rat, dog, giant panda, cow, pig, horse, opossum, platypus, chicken, clawed frog, zebrafish, fruit fly and nematode pathway databases. [file 1471-2164-13-181-S6.tar › map/map00920.png]
