# Supplementary material for: Fast skeletal muscle transcriptome of the Gilthead sea bream (Sparus aurata) determined by next generation sequencing
Source: BMC Genomics. 2012 May 11;13:181. doi: 10.1186/1471-2164-13-181 (PMC3418159; doi:10.1186/1471-2164-13-181)
Supplement: Additional file 4 — Transcripts from fast muscle gilthead sea bream (Sparus aurata L.) 454 transcriptome summarized by their gene ontology annotation (GO) according to Biological Process, Molecular Function and Cellular Component. Table only shows the most abundant GO terms from each category as a percentage and the number of transcripts associated to this level. [file 1471-2164-13-181-S4.docx]

*Suplementary Table 1*. Transcripts from fast muscle gilthead sea bream (S*parus aurata L.*) 454 transcriptome summarized by their gene ontology annotation (GO) according to Biological Process, Molecular Function and Cellular Component. Table only shows the most abundant GO terms from each category as a percentage and the number of transcripts associated to this level.

| *GO general level* | *GO sublevels (GO ID)* | *Percentage over the total (number of transcripts with this annotation)* |
| --- | --- | --- |
| BP | primary metabolic processes (GO:0044238) | 14% (4813) |
| BP | cellular metabolic process (GO:0044237) | 11% (3907) |
| BP | macromolecule metabolic process (GO:0043170) | 10% (3478) |
| BP | regulation of biological process (GO:0050789) | 9% (3323) |
| BP | biosynthesis process (GO:0009058) | 7% (2547) |
| MF | protein binding (GO:0005515) | 35% (5073) |
| MF | nucleotide binding (GO:0000166) | 13% (1899) |
| MF | hydrolase activity (GO:0016787) | 13% (1845) |
| MF | nucleic acid binding (GO:0003676) | 12% (1791) |
| MF | transferase activity (GO:0016740) | 10% (1432) |
| CC | intracellular membrane-bounded organelle (GO:0043231) | 42% (4694) |
| CC | intracellular non-membrane-bounded organelle (GO:0043232) | 18% (1999) |
| CC | cytosol (GO:0005829) | 9% (999) |
| CC | nuclear part (GO:0044428) | 9% (1028) |
| CC | intracellular organelle lumen (GO:0070013) | 7% (794) |

BP: Biological Process

MF: Molecular Function

CC: Cell Component
