# Supplementary material for: Fast skeletal muscle transcriptome of the Gilthead sea bream (Sparus aurata) determined by next generation sequencing
Source: BMC Genomics. 2012 May 11;13:181. doi: 10.1186/1471-2164-13-181 (PMC3418159; doi:10.1186/1471-2164-13-181)
Supplement: Additional file 5 — Kyoto Encyclopaedia of Genes and Genomes (KEGG) maps present in the gilthead sea bream fast muscle transcriptome. Transcripts were annotated to KEGG maps using the automatic annotator tool KAAS [60]. [file 1471-2164-13-181-S5.docx]

*Supplementary Table 2.* KEEG maps present in the gilthead sea bream (*Sparus aurata*) fast muscle transcriptome. Transcripts were annotated to KEEG maps using the automatic annotator tool KAAS (http://www.genome.jp/tools/kaas/).

| KEEG General Category | Number of Maps | Percentage over the total (%) |
| --- | --- | --- |
|  |  |  |
| Metabolism | 161 | 47 |
| Genetic Information Processing | 32 | 9 |
| Environmental information processing | 36 | 10 |
| Cellular processes | 21 | 6 |
| Organismal system | 48 | 14 |
| Human diseases | 46 | 13 |
