# Supplementary figures and images for: Fast skeletal muscle transcriptome of the Gilthead sea bream (Sparus aurata) determined by next generation sequencing (part 3 of 3)
Source: BMC Genomics. 2012 May 11;13:181. doi: 10.1186/1471-2164-13-181 (PMC3418159; doi:10.1186/1471-2164-13-181)

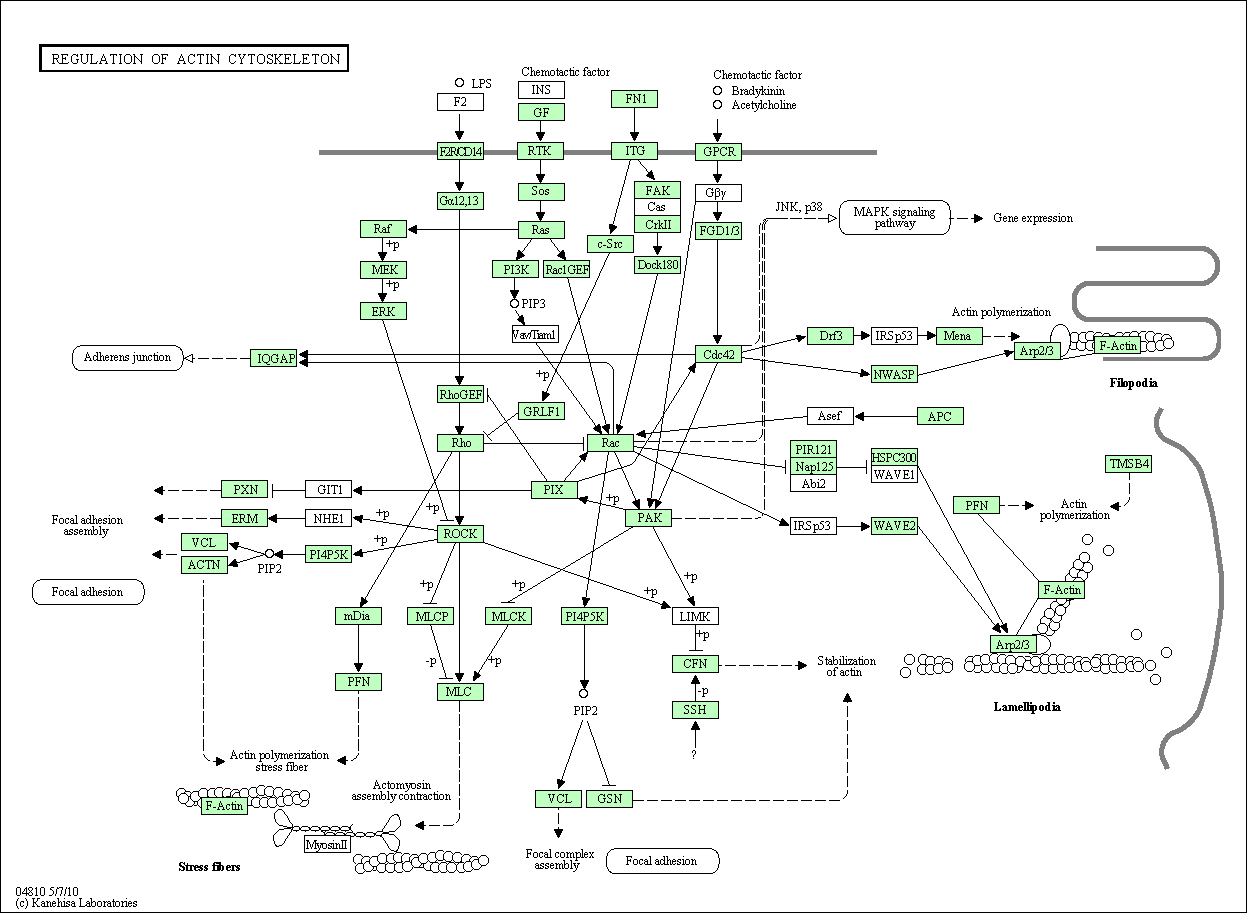

Supplement: Additional file 6 — KEGG annotation results. File contains the KEGG maps where isotigs were successfully mapped. Pathway element in green boxes indicates the components represented by at least, one isotig. Gilthead sea bream isotigs were automatically annotated to KEGG maps using KAAS website [60]. The SBH method, optimized for ESTs annotation, was used against human, chimpanzee, orang-utan, rhesus, mouse, rat, dog, giant panda, cow, pig, horse, opossum, platypus, chicken, clawed frog, zebrafish, fruit fly and nematode pathway databases. [file 1471-2164-13-181-S6.tar › map/map04810.png]

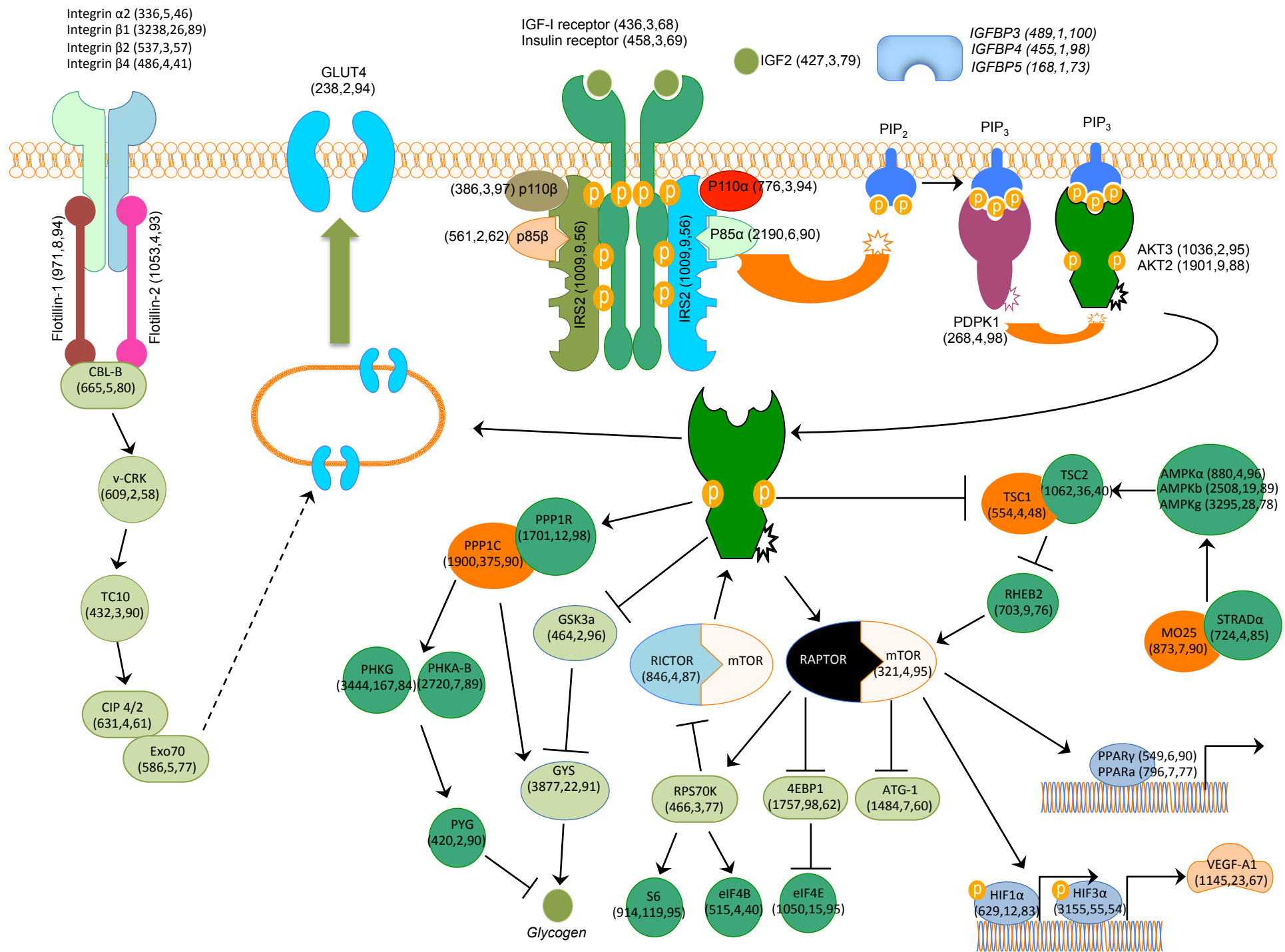

Supplement: Additional file 8 — PI3K/Akt and mTOR genes represented in the transcriptome mapped onto a reconstruction of the pathway based on KEGG maps (KO:04150 and KO:04910). AMPKα/β/γ, 5′-AMP-activated protein kinase catalytic subunit alpha/beta/gamma; AKT2, Rac-beta serine/threonine protein kinase 2; AKT3, Rac-beta serine/threonine protein kinase 3; ATG1, Serine/threonine-protein kinase ULK1; CBL-B, E3-Ubiquitin-protein kinase CBL; CIP4 (TRIP10), Thyroid hormone receptor interactor 10; eIF4B, Eukaryotic translation initiation factor 4B; eIF4E, Eukaryotic translation initiation factor 4E; Exo-70, Exocyst complex component 7; GYS, Glycogen synthase; GSK3a, Glycogen synthase kinase 3-alpha; GLUT4, Solute carrier family 2, facilitated glucose transporter member 4; HIF1α, Hypoxia inducible factor 1-alpha; HIF3α, Hypoxia inducible factor 3-alpha; IGF, insulin-like growth factor; IGFBP, insulin-like growth factor binding protein; IRS, insulin receptor substrate; mTOR, Mammalian target of rapamycin; p110, Phosphatidylinositol 3-kinase catalytic subunit; p85, Phosphatidylinositol 3-kinase regulatory subunit; PDPK1, 3-phosphoinositide dependent protein kinase 1; PPP1R, Protein phosphatase 1 regulatory subunit; PPP1C, Protein phosphatase 1 catalytic subunit; PHKG, Phosphorylase b kinase gamma; PHK1-B, Phosphorylase kinase alpha-beta subunit; PPAR, Peroxisome proliferation-activated receptor; PYG, Starch phosphorylase; RICTOR, Rapamycin-insensitive companion of mTOR; RAPTOR, Regulatory-associated protein of mTOR; RPS70K, p70 Ribosomal protein S6 kinase; RHEB2, Ras homolog enriched brain; STRADα, STE20 related kinase adaptor alpha; S6, Ribosomal protein S6; TSC, Tuberous sclerosis; TC10 (RHOQ), Ras homolog gene family member Q; VEGF-A1, Vascular endothelial growth factor A1; v-CRK, Proto-oncogen C-crk; 4EBP1, Eukaryotic translator factor 4E binding protein. Numbers associated with the gene name represents isotig length (bp), isotig mean coverage and percentage of identity with the zebrafish orthologue. [file 1471-2164-13-181-S8.pdf]
