# Supplementary figures and images for: Fast skeletal muscle transcriptome of the Gilthead sea bream (Sparus aurata) determined by next generation sequencing (part 2 of 3)
Source: BMC Genomics. 2012 May 11;13:181. doi: 10.1186/1471-2164-13-181 (PMC3418159; doi:10.1186/1471-2164-13-181)

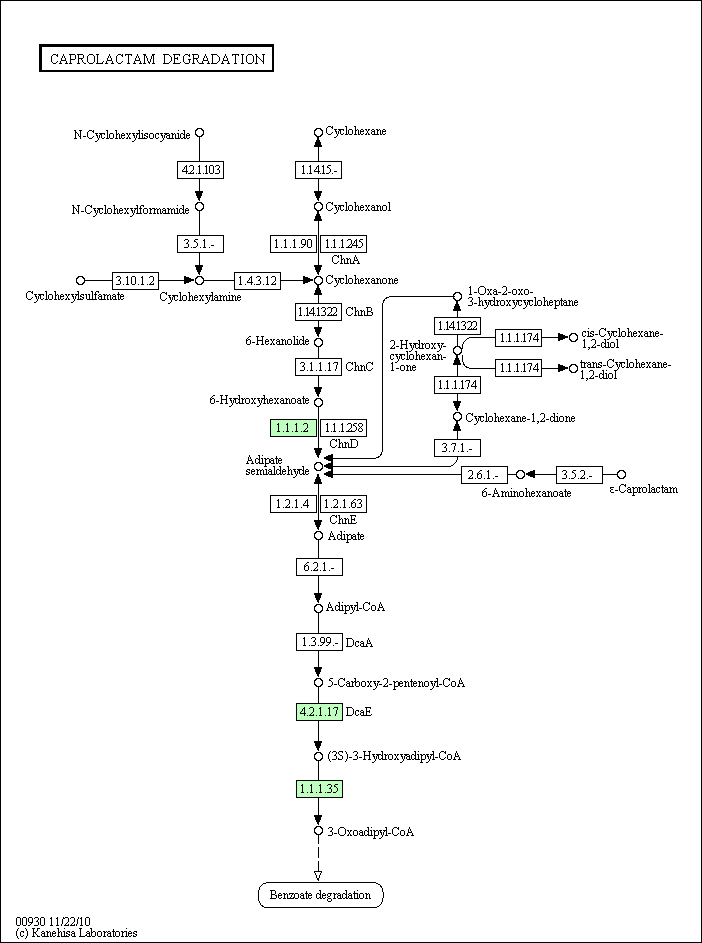

Supplement: Additional file 6 — KEGG annotation results. File contains the KEGG maps where isotigs were successfully mapped. Pathway element in green boxes indicates the components represented by at least, one isotig. Gilthead sea bream isotigs were automatically annotated to KEGG maps using KAAS website [60]. The SBH method, optimized for ESTs annotation, was used against human, chimpanzee, orang-utan, rhesus, mouse, rat, dog, giant panda, cow, pig, horse, opossum, platypus, chicken, clawed frog, zebrafish, fruit fly and nematode pathway databases. [file 1471-2164-13-181-S6.tar › map/map00930.png]

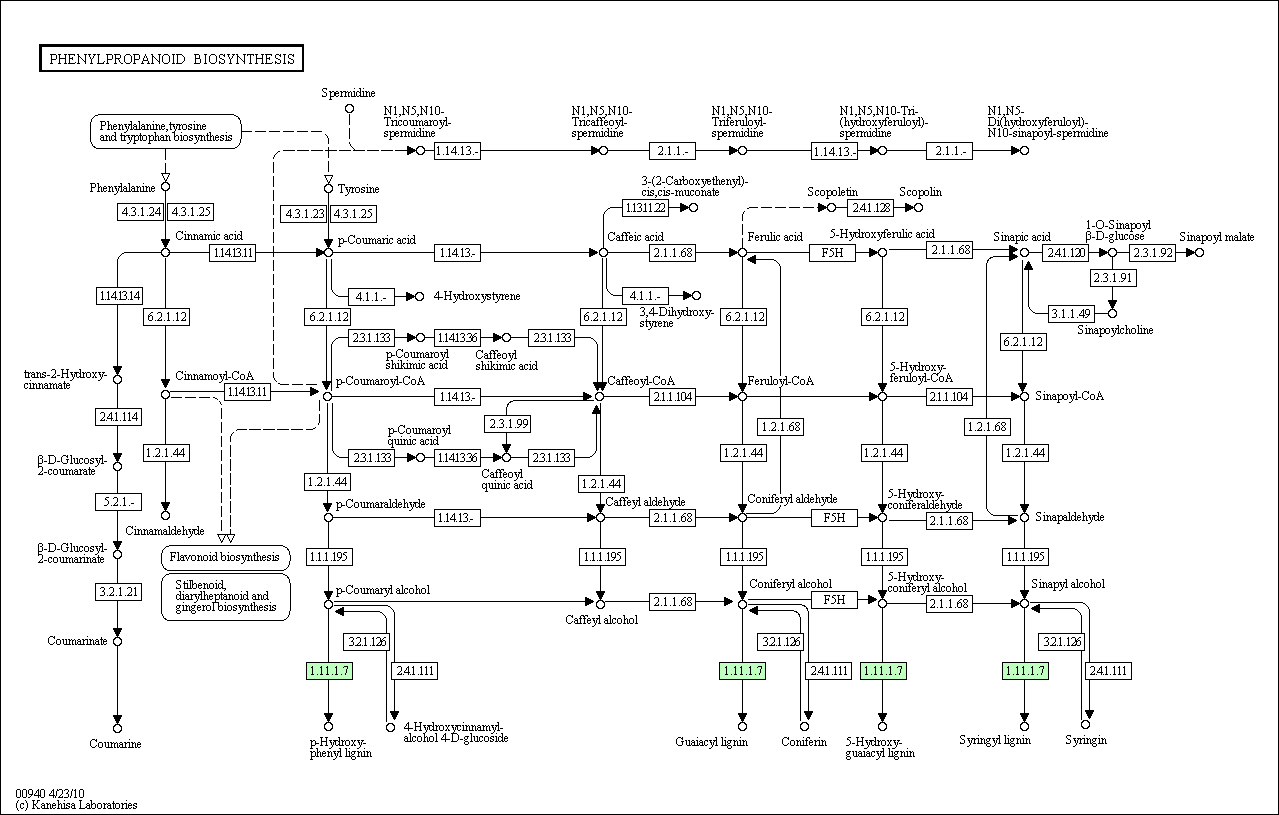

Supplement: Additional file 6 — KEGG annotation results. File contains the KEGG maps where isotigs were successfully mapped. Pathway element in green boxes indicates the components represented by at least, one isotig. Gilthead sea bream isotigs were automatically annotated to KEGG maps using KAAS website [60]. The SBH method, optimized for ESTs annotation, was used against human, chimpanzee, orang-utan, rhesus, mouse, rat, dog, giant panda, cow, pig, horse, opossum, platypus, chicken, clawed frog, zebrafish, fruit fly and nematode pathway databases. [file 1471-2164-13-181-S6.tar › map/map00940.png]

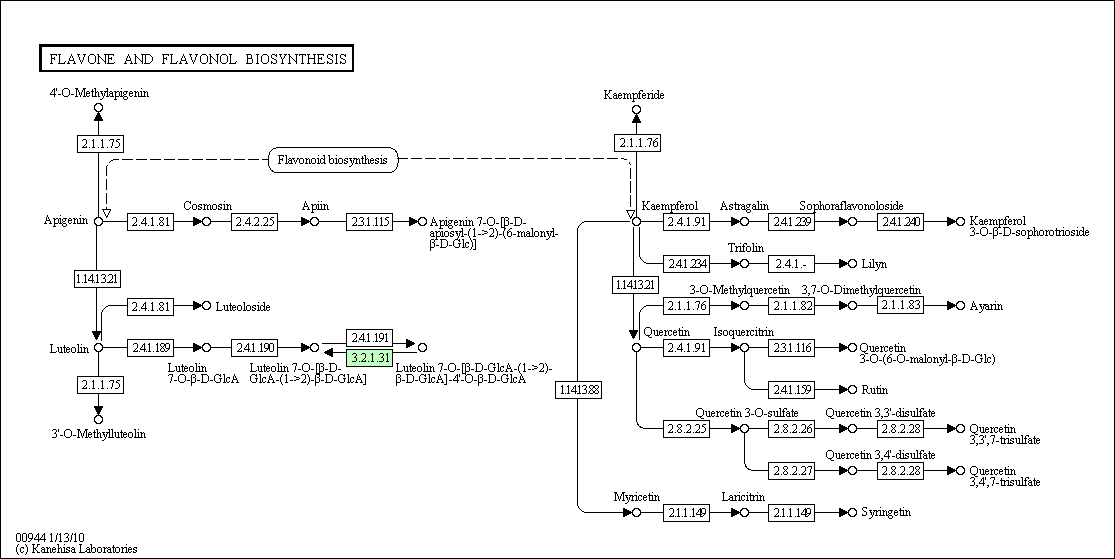

Supplement: Additional file 6 — KEGG annotation results. File contains the KEGG maps where isotigs were successfully mapped. Pathway element in green boxes indicates the components represented by at least, one isotig. Gilthead sea bream isotigs were automatically annotated to KEGG maps using KAAS website [60]. The SBH method, optimized for ESTs annotation, was used against human, chimpanzee, orang-utan, rhesus, mouse, rat, dog, giant panda, cow, pig, horse, opossum, platypus, chicken, clawed frog, zebrafish, fruit fly and nematode pathway databases. [file 1471-2164-13-181-S6.tar › map/map00944.png]

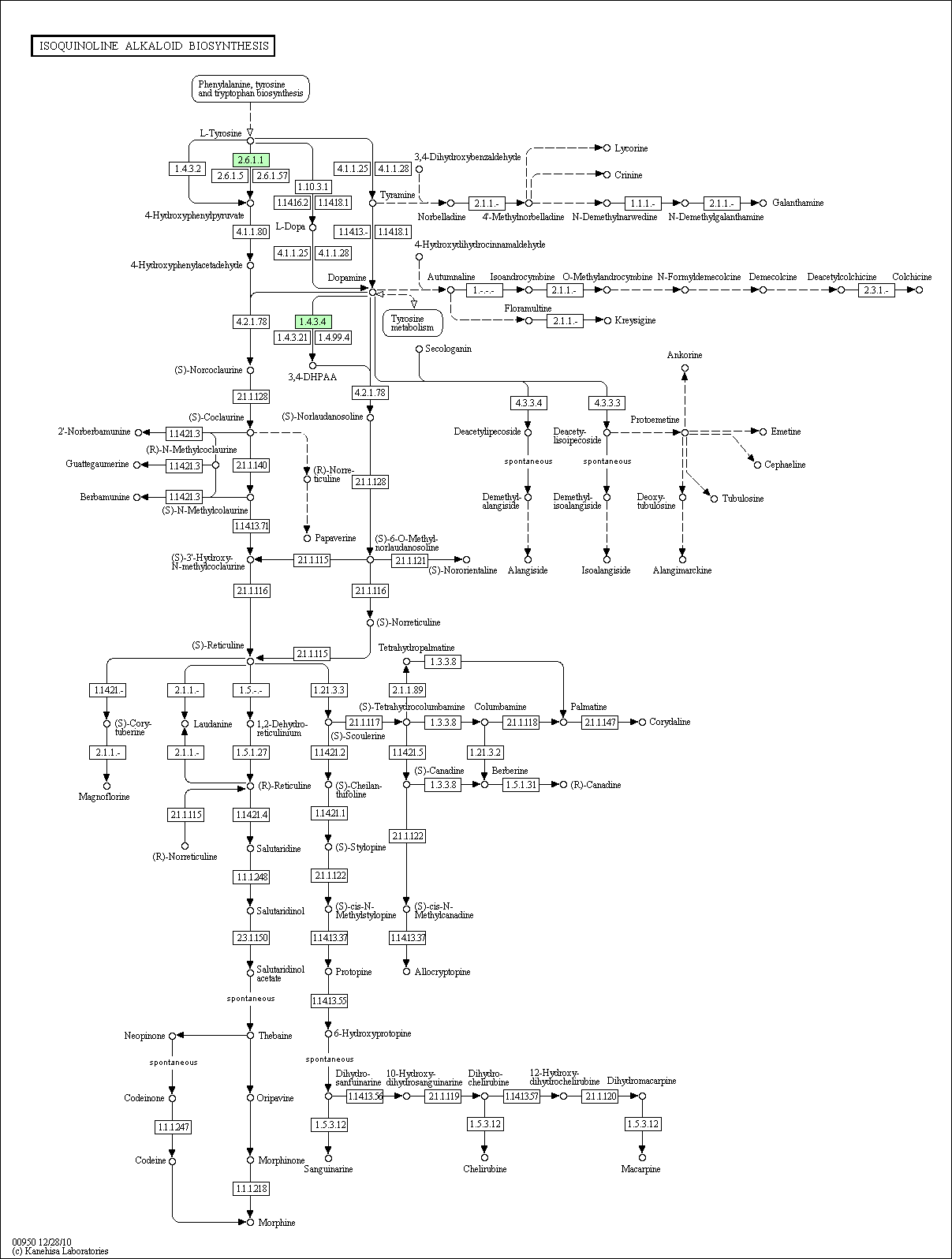

Supplement: Additional file 6 — KEGG annotation results. File contains the KEGG maps where isotigs were successfully mapped. Pathway element in green boxes indicates the components represented by at least, one isotig. Gilthead sea bream isotigs were automatically annotated to KEGG maps using KAAS website [60]. The SBH method, optimized for ESTs annotation, was used against human, chimpanzee, orang-utan, rhesus, mouse, rat, dog, giant panda, cow, pig, horse, opossum, platypus, chicken, clawed frog, zebrafish, fruit fly and nematode pathway databases. [file 1471-2164-13-181-S6.tar › map/map00950.png]

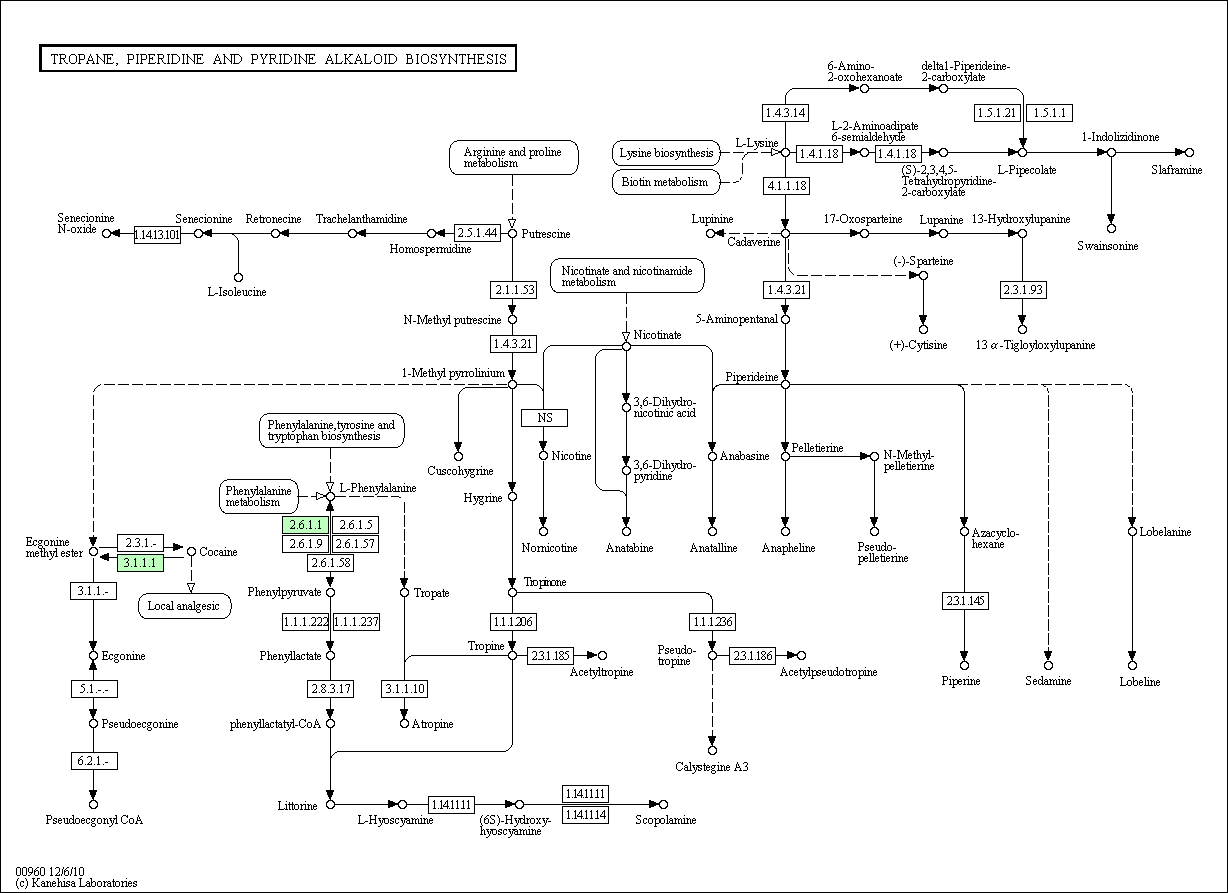

Supplement: Additional file 6 — KEGG annotation results. File contains the KEGG maps where isotigs were successfully mapped. Pathway element in green boxes indicates the components represented by at least, one isotig. Gilthead sea bream isotigs were automatically annotated to KEGG maps using KAAS website [60]. The SBH method, optimized for ESTs annotation, was used against human, chimpanzee, orang-utan, rhesus, mouse, rat, dog, giant panda, cow, pig, horse, opossum, platypus, chicken, clawed frog, zebrafish, fruit fly and nematode pathway databases. [file 1471-2164-13-181-S6.tar › map/map00960.png]

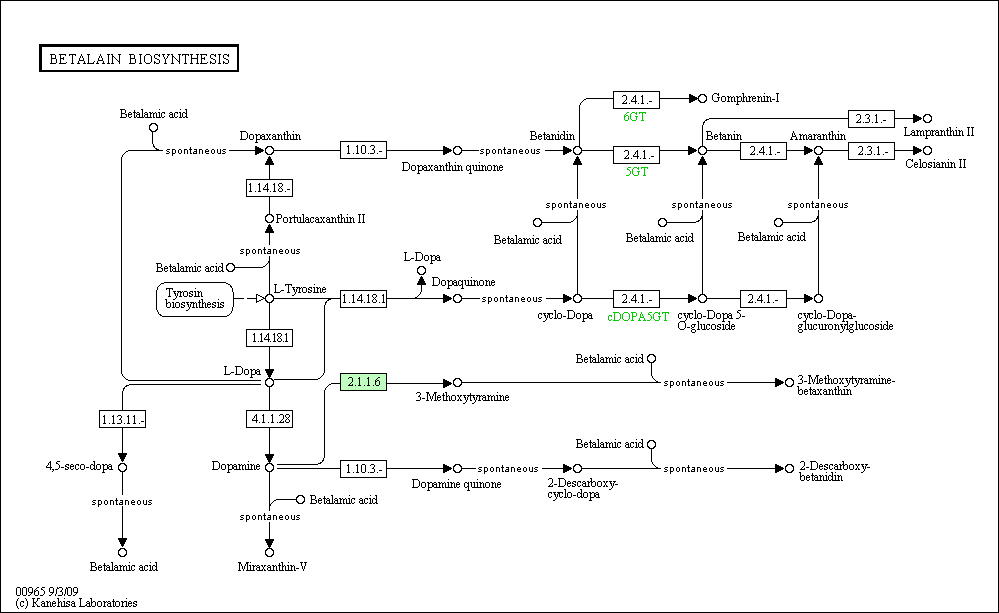

Supplement: Additional file 6 — KEGG annotation results. File contains the KEGG maps where isotigs were successfully mapped. Pathway element in green boxes indicates the components represented by at least, one isotig. Gilthead sea bream isotigs were automatically annotated to KEGG maps using KAAS website [60]. The SBH method, optimized for ESTs annotation, was used against human, chimpanzee, orang-utan, rhesus, mouse, rat, dog, giant panda, cow, pig, horse, opossum, platypus, chicken, clawed frog, zebrafish, fruit fly and nematode pathway databases. [file 1471-2164-13-181-S6.tar › map/map00965.png]

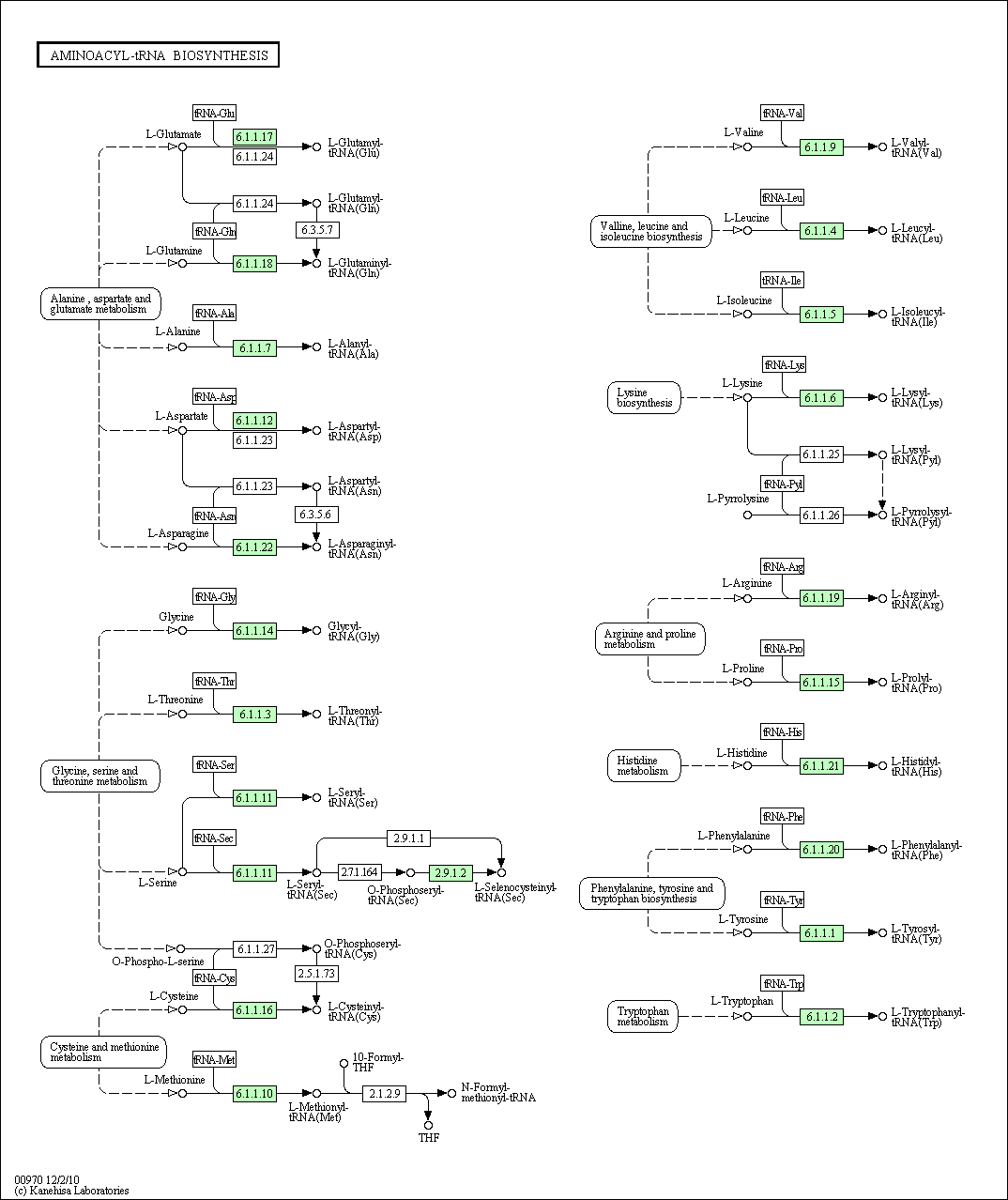

Supplement: Additional file 6 — KEGG annotation results. File contains the KEGG maps where isotigs were successfully mapped. Pathway element in green boxes indicates the components represented by at least, one isotig. Gilthead sea bream isotigs were automatically annotated to KEGG maps using KAAS website [60]. The SBH method, optimized for ESTs annotation, was used against human, chimpanzee, orang-utan, rhesus, mouse, rat, dog, giant panda, cow, pig, horse, opossum, platypus, chicken, clawed frog, zebrafish, fruit fly and nematode pathway databases. [file 1471-2164-13-181-S6.tar › map/map00970.png]

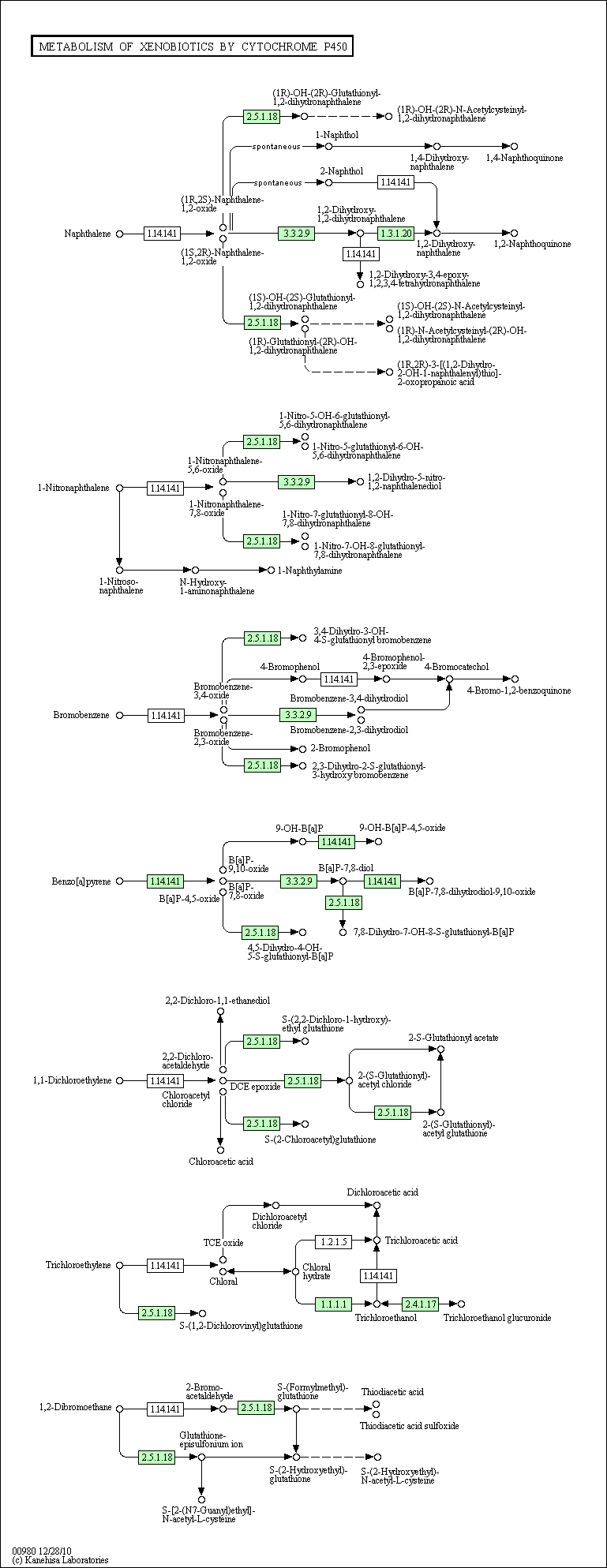

Supplement: Additional file 6 — KEGG annotation results. File contains the KEGG maps where isotigs were successfully mapped. Pathway element in green boxes indicates the components represented by at least, one isotig. Gilthead sea bream isotigs were automatically annotated to KEGG maps using KAAS website [60]. The SBH method, optimized for ESTs annotation, was used against human, chimpanzee, orang-utan, rhesus, mouse, rat, dog, giant panda, cow, pig, horse, opossum, platypus, chicken, clawed frog, zebrafish, fruit fly and nematode pathway databases. [file 1471-2164-13-181-S6.tar › map/map00980.png]

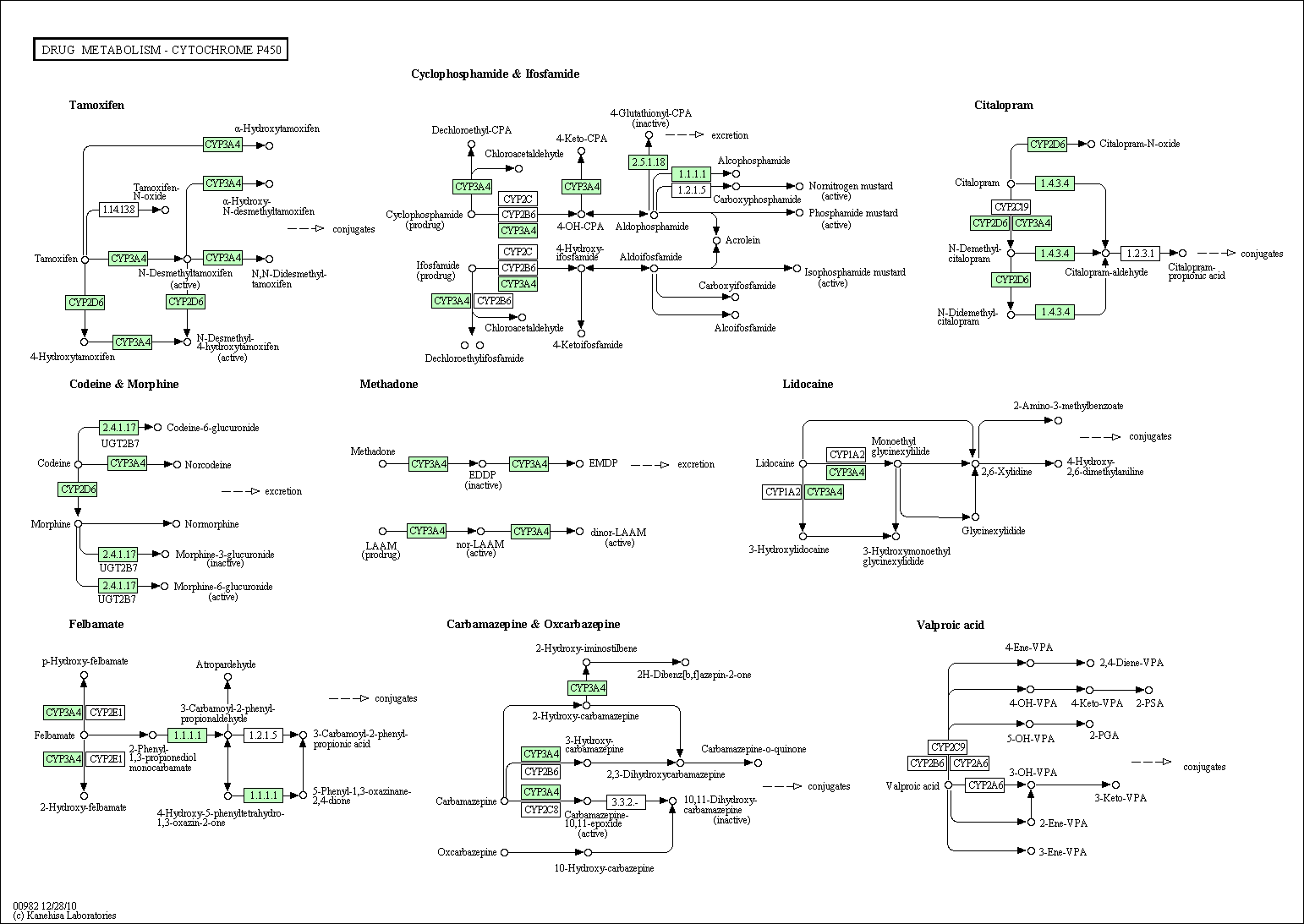

Supplement: Additional file 6 — KEGG annotation results. File contains the KEGG maps where isotigs were successfully mapped. Pathway element in green boxes indicates the components represented by at least, one isotig. Gilthead sea bream isotigs were automatically annotated to KEGG maps using KAAS website [60]. The SBH method, optimized for ESTs annotation, was used against human, chimpanzee, orang-utan, rhesus, mouse, rat, dog, giant panda, cow, pig, horse, opossum, platypus, chicken, clawed frog, zebrafish, fruit fly and nematode pathway databases. [file 1471-2164-13-181-S6.tar › map/map00982.png]

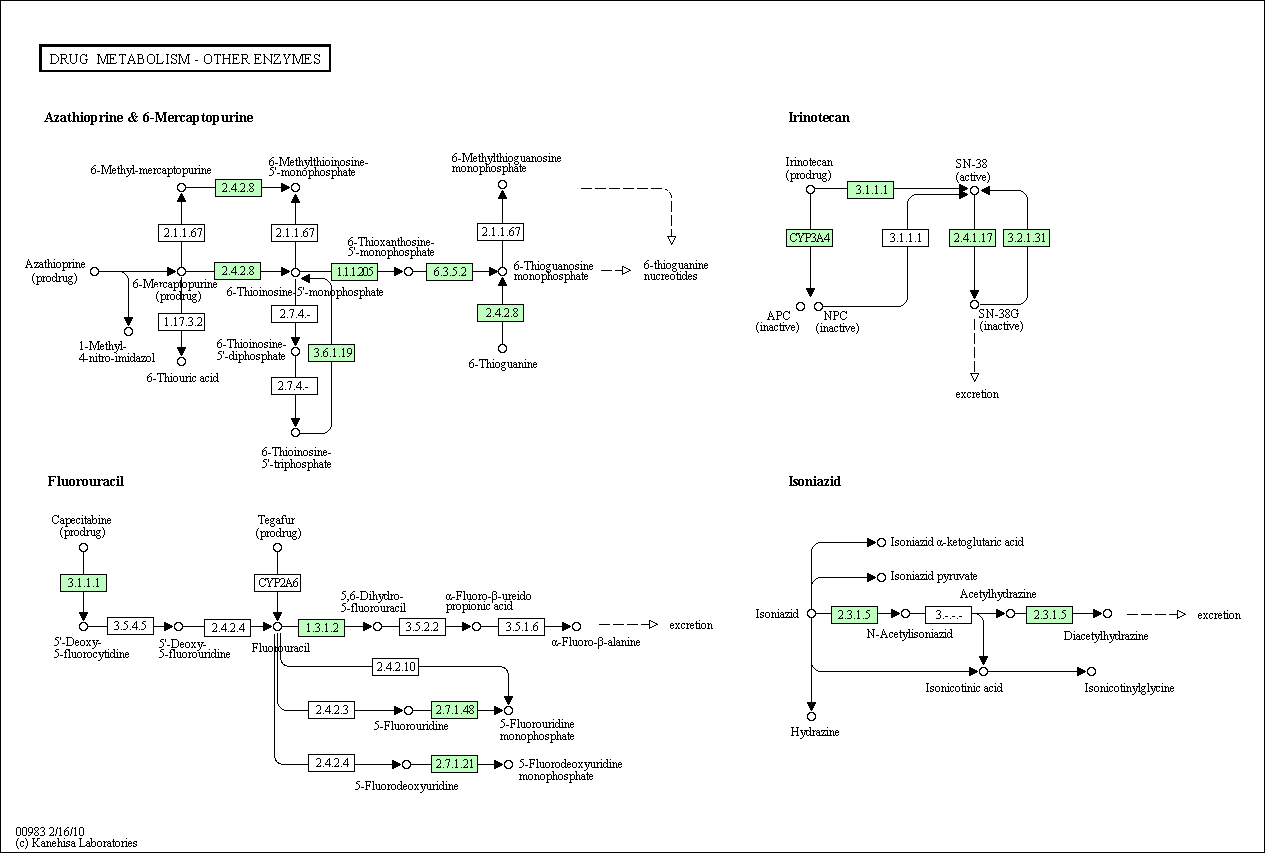

Supplement: Additional file 6 — KEGG annotation results. File contains the KEGG maps where isotigs were successfully mapped. Pathway element in green boxes indicates the components represented by at least, one isotig. Gilthead sea bream isotigs were automatically annotated to KEGG maps using KAAS website [60]. The SBH method, optimized for ESTs annotation, was used against human, chimpanzee, orang-utan, rhesus, mouse, rat, dog, giant panda, cow, pig, horse, opossum, platypus, chicken, clawed frog, zebrafish, fruit fly and nematode pathway databases. [file 1471-2164-13-181-S6.tar › map/map00983.png]

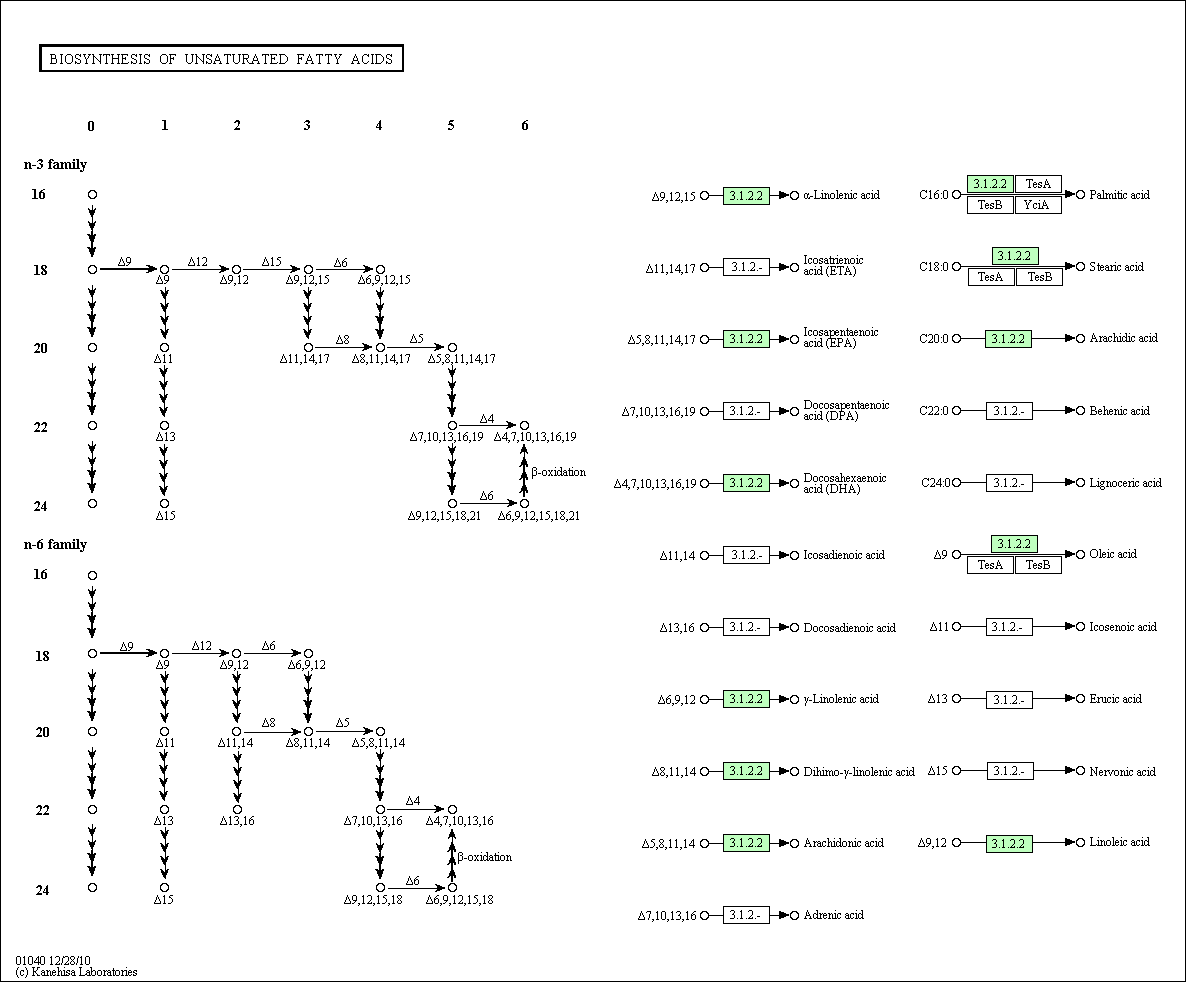

Supplement: Additional file 6 — KEGG annotation results. File contains the KEGG maps where isotigs were successfully mapped. Pathway element in green boxes indicates the components represented by at least, one isotig. Gilthead sea bream isotigs were automatically annotated to KEGG maps using KAAS website [60]. The SBH method, optimized for ESTs annotation, was used against human, chimpanzee, orang-utan, rhesus, mouse, rat, dog, giant panda, cow, pig, horse, opossum, platypus, chicken, clawed frog, zebrafish, fruit fly and nematode pathway databases. [file 1471-2164-13-181-S6.tar › map/map01040.png]

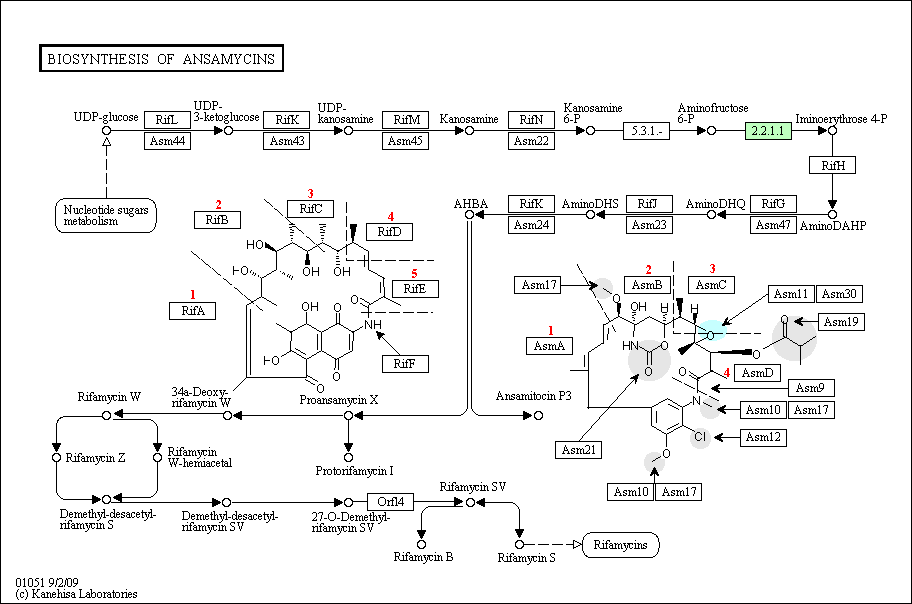

Supplement: Additional file 6 — KEGG annotation results. File contains the KEGG maps where isotigs were successfully mapped. Pathway element in green boxes indicates the components represented by at least, one isotig. Gilthead sea bream isotigs were automatically annotated to KEGG maps using KAAS website [60]. The SBH method, optimized for ESTs annotation, was used against human, chimpanzee, orang-utan, rhesus, mouse, rat, dog, giant panda, cow, pig, horse, opossum, platypus, chicken, clawed frog, zebrafish, fruit fly and nematode pathway databases. [file 1471-2164-13-181-S6.tar › map/map01051.png]

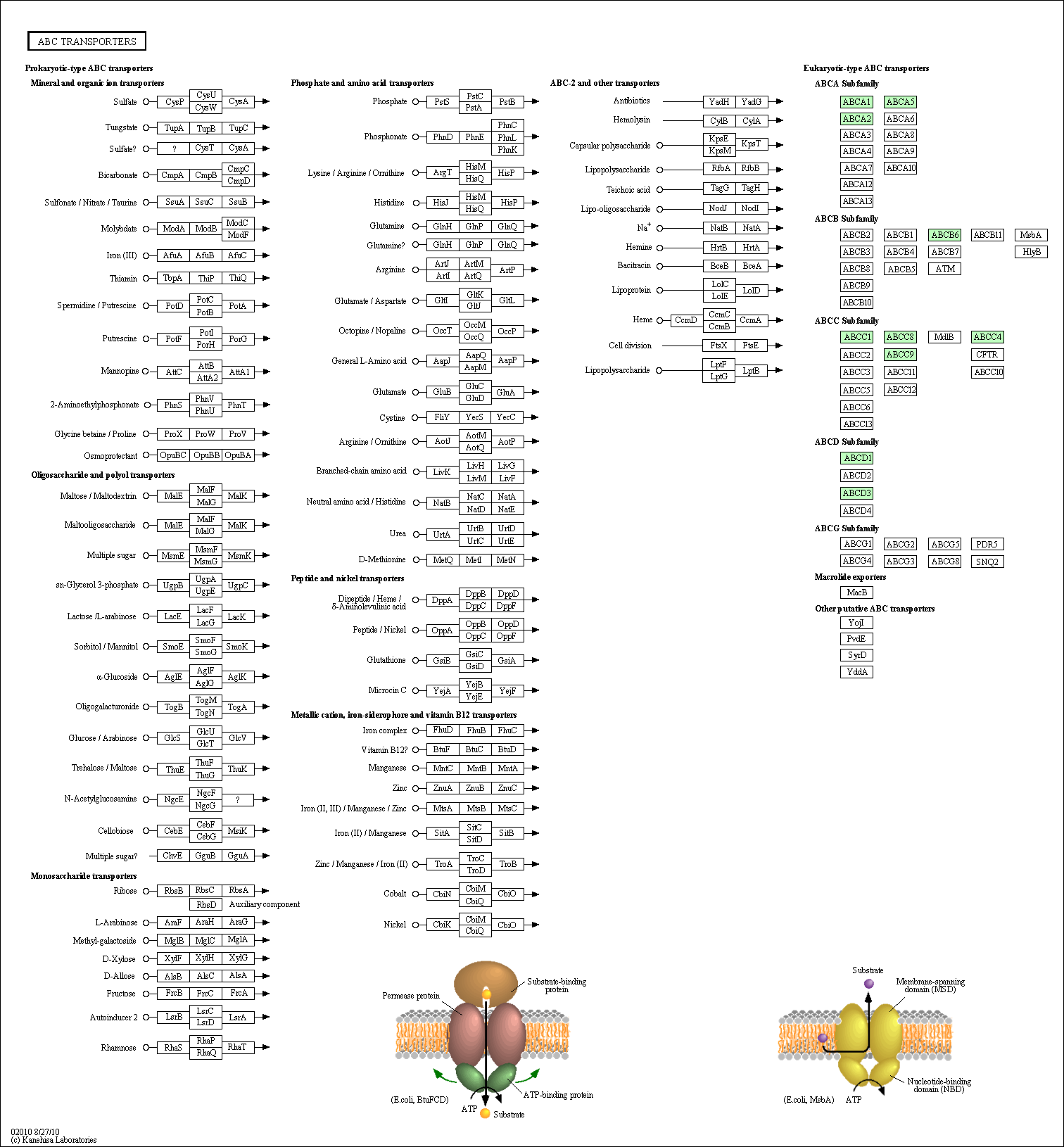

Supplement: Additional file 6 — KEGG annotation results. File contains the KEGG maps where isotigs were successfully mapped. Pathway element in green boxes indicates the components represented by at least, one isotig. Gilthead sea bream isotigs were automatically annotated to KEGG maps using KAAS website [60]. The SBH method, optimized for ESTs annotation, was used against human, chimpanzee, orang-utan, rhesus, mouse, rat, dog, giant panda, cow, pig, horse, opossum, platypus, chicken, clawed frog, zebrafish, fruit fly and nematode pathway databases. [file 1471-2164-13-181-S6.tar › map/map02010.png]

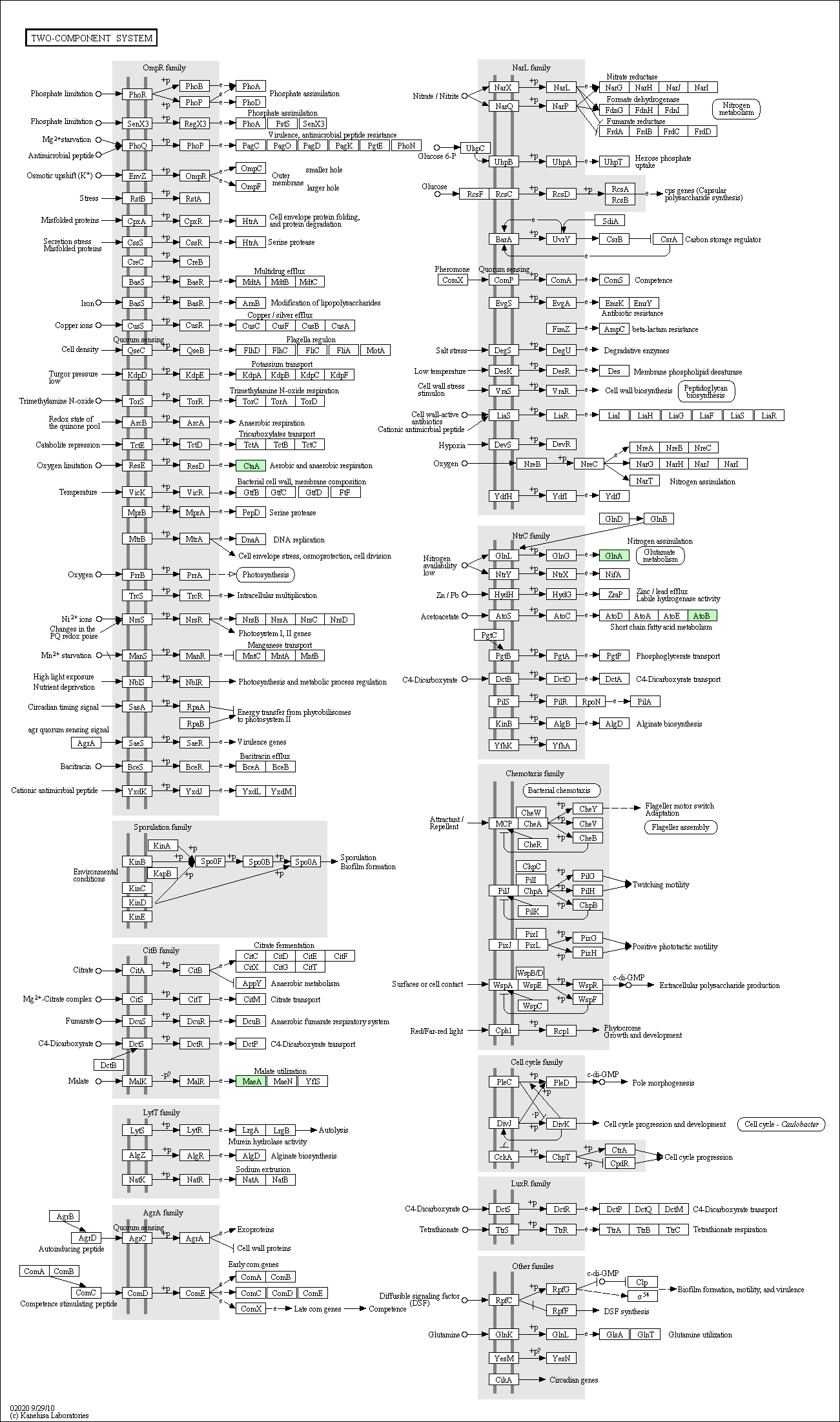

Supplement: Additional file 6 — KEGG annotation results. File contains the KEGG maps where isotigs were successfully mapped. Pathway element in green boxes indicates the components represented by at least, one isotig. Gilthead sea bream isotigs were automatically annotated to KEGG maps using KAAS website [60]. The SBH method, optimized for ESTs annotation, was used against human, chimpanzee, orang-utan, rhesus, mouse, rat, dog, giant panda, cow, pig, horse, opossum, platypus, chicken, clawed frog, zebrafish, fruit fly and nematode pathway databases. [file 1471-2164-13-181-S6.tar › map/map02020.png]

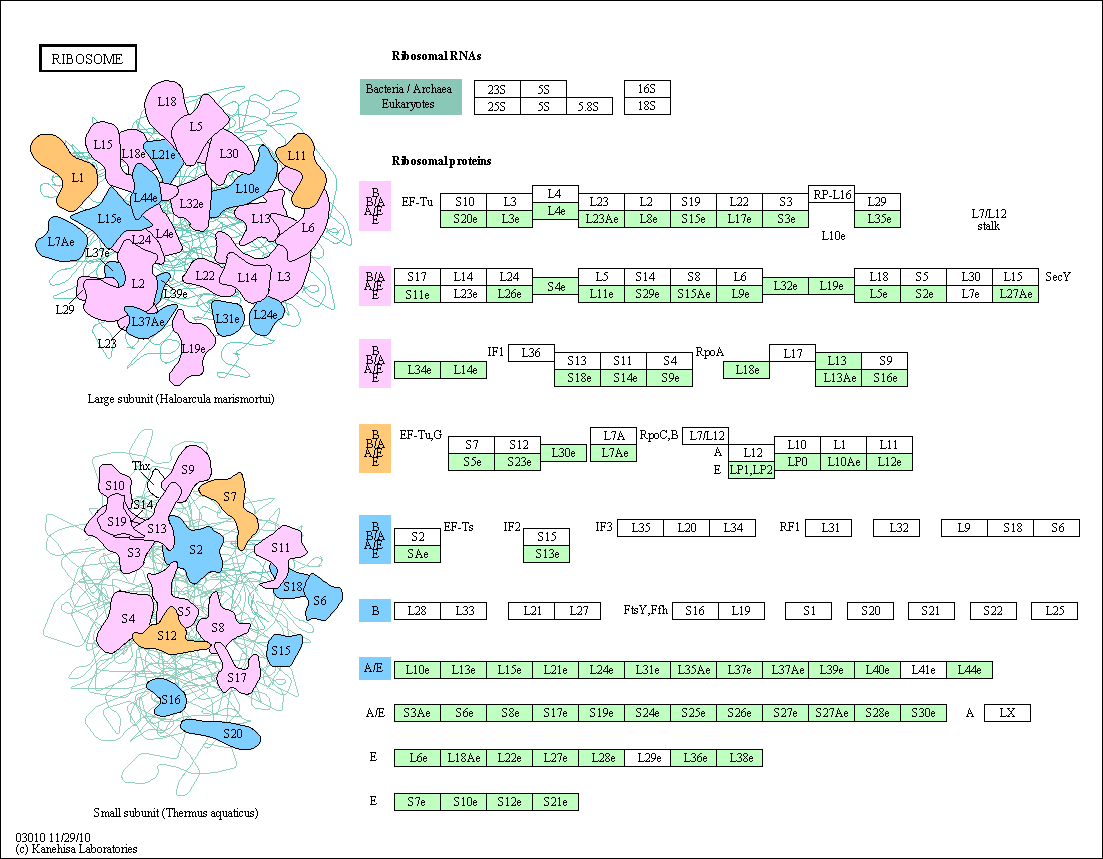

Supplement: Additional file 6 — KEGG annotation results. File contains the KEGG maps where isotigs were successfully mapped. Pathway element in green boxes indicates the components represented by at least, one isotig. Gilthead sea bream isotigs were automatically annotated to KEGG maps using KAAS website [60]. The SBH method, optimized for ESTs annotation, was used against human, chimpanzee, orang-utan, rhesus, mouse, rat, dog, giant panda, cow, pig, horse, opossum, platypus, chicken, clawed frog, zebrafish, fruit fly and nematode pathway databases. [file 1471-2164-13-181-S6.tar › map/map03010.png]

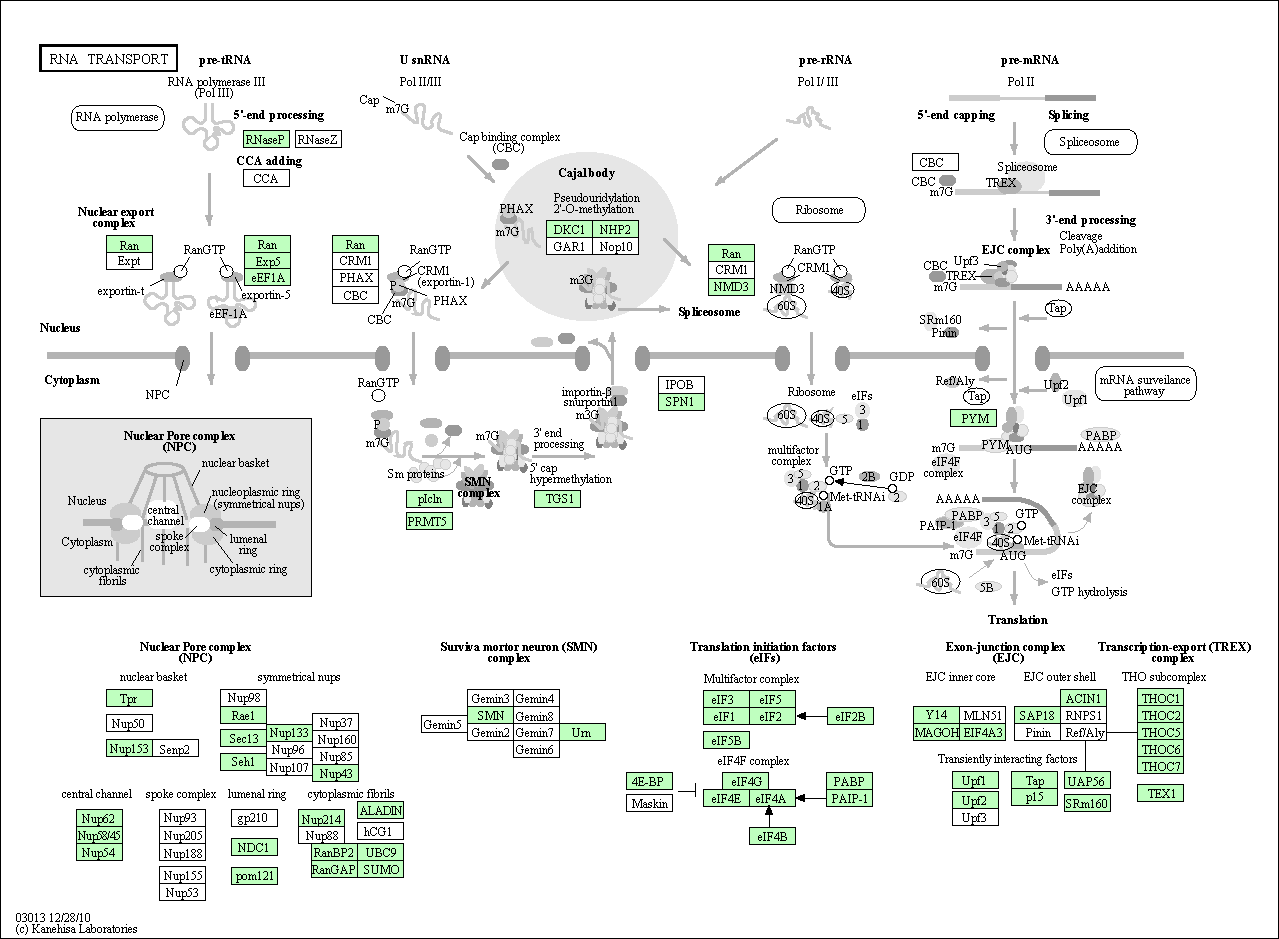

Supplement: Additional file 6 — KEGG annotation results. File contains the KEGG maps where isotigs were successfully mapped. Pathway element in green boxes indicates the components represented by at least, one isotig. Gilthead sea bream isotigs were automatically annotated to KEGG maps using KAAS website [60]. The SBH method, optimized for ESTs annotation, was used against human, chimpanzee, orang-utan, rhesus, mouse, rat, dog, giant panda, cow, pig, horse, opossum, platypus, chicken, clawed frog, zebrafish, fruit fly and nematode pathway databases. [file 1471-2164-13-181-S6.tar › map/map03013.png]

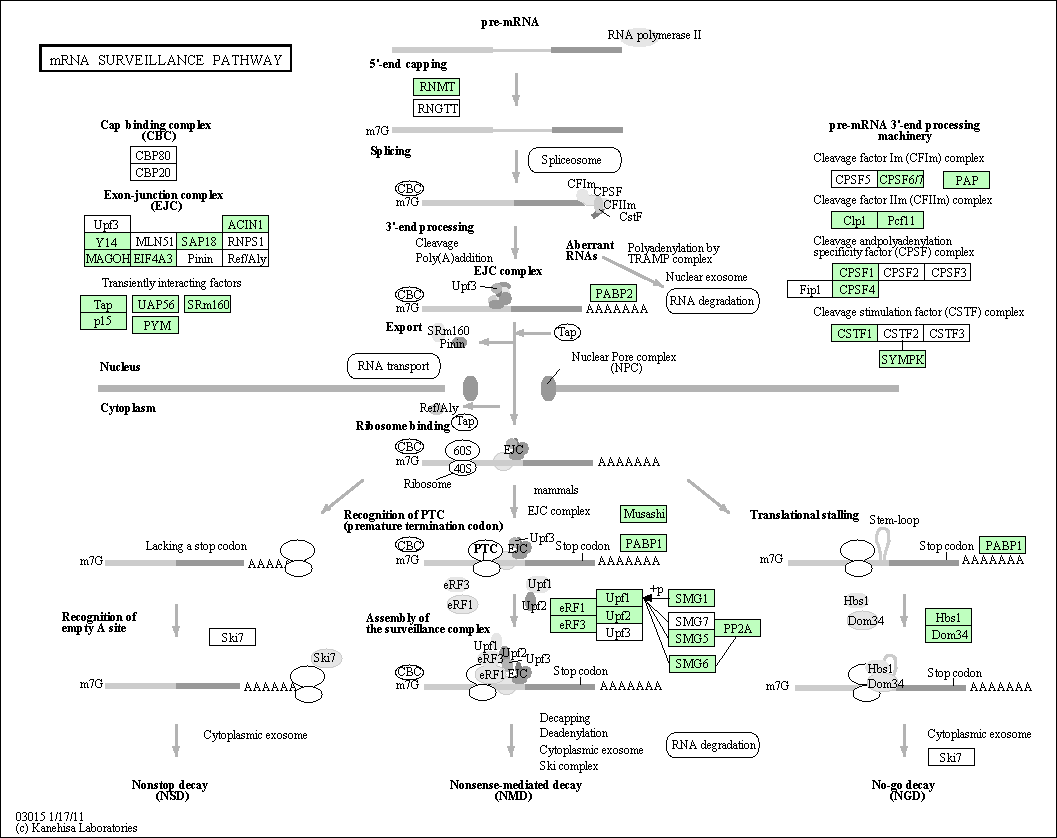

Supplement: Additional file 6 — KEGG annotation results. File contains the KEGG maps where isotigs were successfully mapped. Pathway element in green boxes indicates the components represented by at least, one isotig. Gilthead sea bream isotigs were automatically annotated to KEGG maps using KAAS website [60]. The SBH method, optimized for ESTs annotation, was used against human, chimpanzee, orang-utan, rhesus, mouse, rat, dog, giant panda, cow, pig, horse, opossum, platypus, chicken, clawed frog, zebrafish, fruit fly and nematode pathway databases. [file 1471-2164-13-181-S6.tar › map/map03015.png]

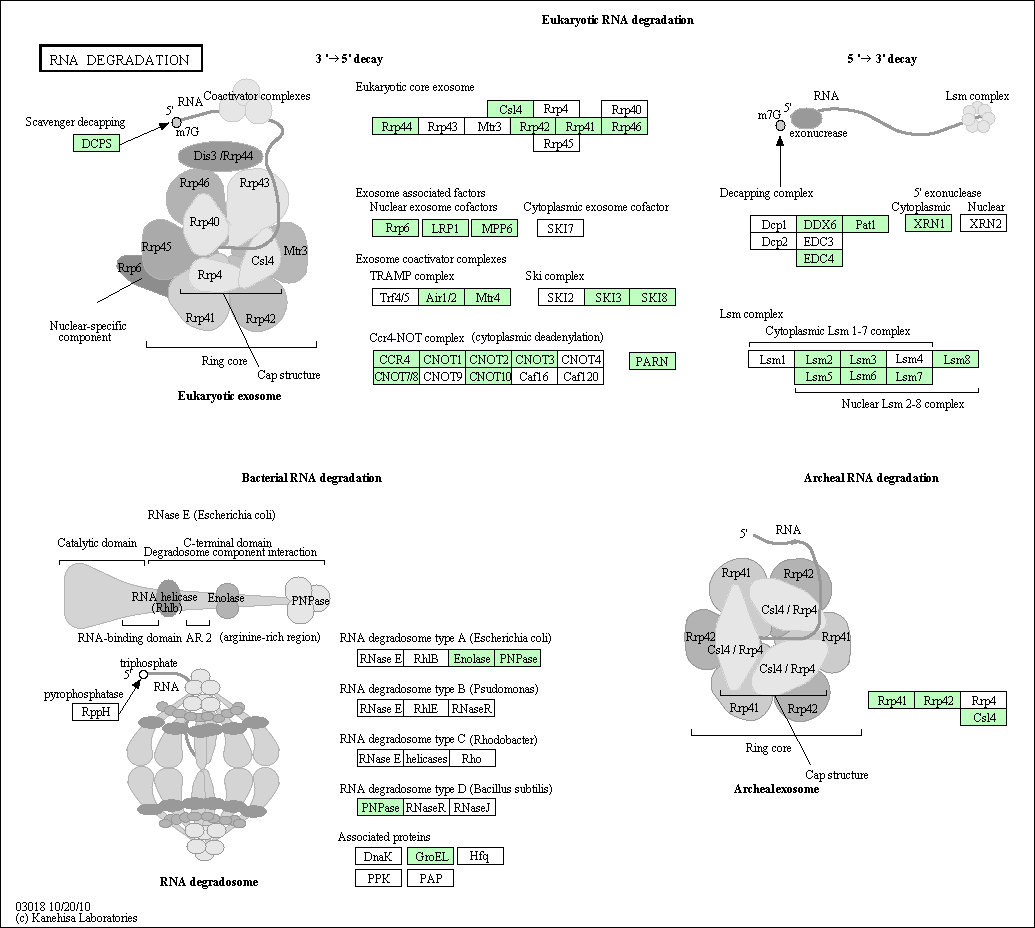

Supplement: Additional file 6 — KEGG annotation results. File contains the KEGG maps where isotigs were successfully mapped. Pathway element in green boxes indicates the components represented by at least, one isotig. Gilthead sea bream isotigs were automatically annotated to KEGG maps using KAAS website [60]. The SBH method, optimized for ESTs annotation, was used against human, chimpanzee, orang-utan, rhesus, mouse, rat, dog, giant panda, cow, pig, horse, opossum, platypus, chicken, clawed frog, zebrafish, fruit fly and nematode pathway databases. [file 1471-2164-13-181-S6.tar › map/map03018.png]

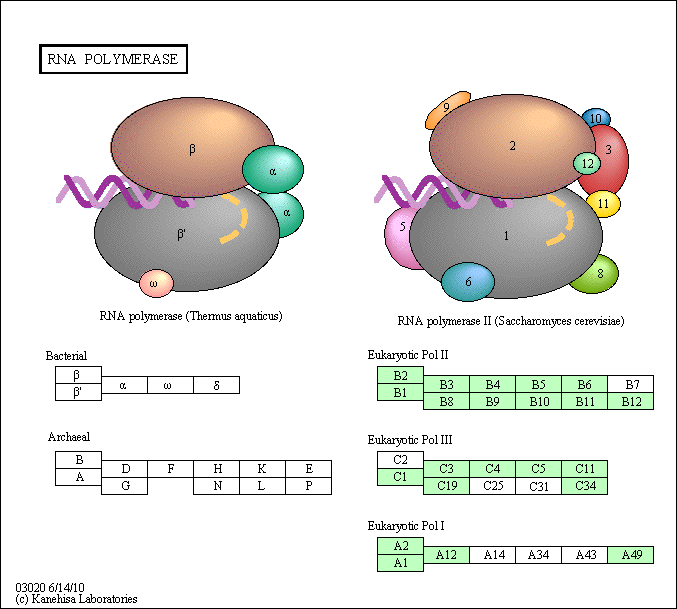

Supplement: Additional file 6 — KEGG annotation results. File contains the KEGG maps where isotigs were successfully mapped. Pathway element in green boxes indicates the components represented by at least, one isotig. Gilthead sea bream isotigs were automatically annotated to KEGG maps using KAAS website [60]. The SBH method, optimized for ESTs annotation, was used against human, chimpanzee, orang-utan, rhesus, mouse, rat, dog, giant panda, cow, pig, horse, opossum, platypus, chicken, clawed frog, zebrafish, fruit fly and nematode pathway databases. [file 1471-2164-13-181-S6.tar › map/map03020.png]

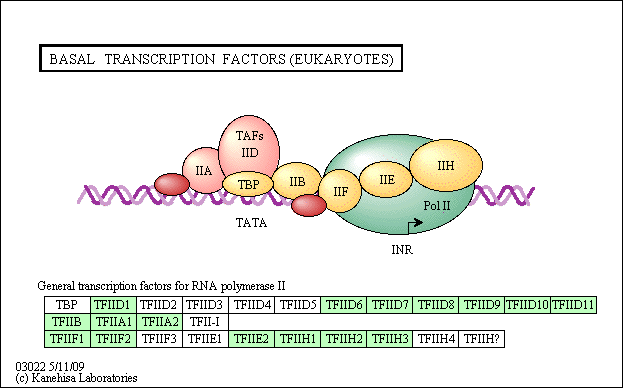

Supplement: Additional file 6 — KEGG annotation results. File contains the KEGG maps where isotigs were successfully mapped. Pathway element in green boxes indicates the components represented by at least, one isotig. Gilthead sea bream isotigs were automatically annotated to KEGG maps using KAAS website [60]. The SBH method, optimized for ESTs annotation, was used against human, chimpanzee, orang-utan, rhesus, mouse, rat, dog, giant panda, cow, pig, horse, opossum, platypus, chicken, clawed frog, zebrafish, fruit fly and nematode pathway databases. [file 1471-2164-13-181-S6.tar › map/map03022.png]

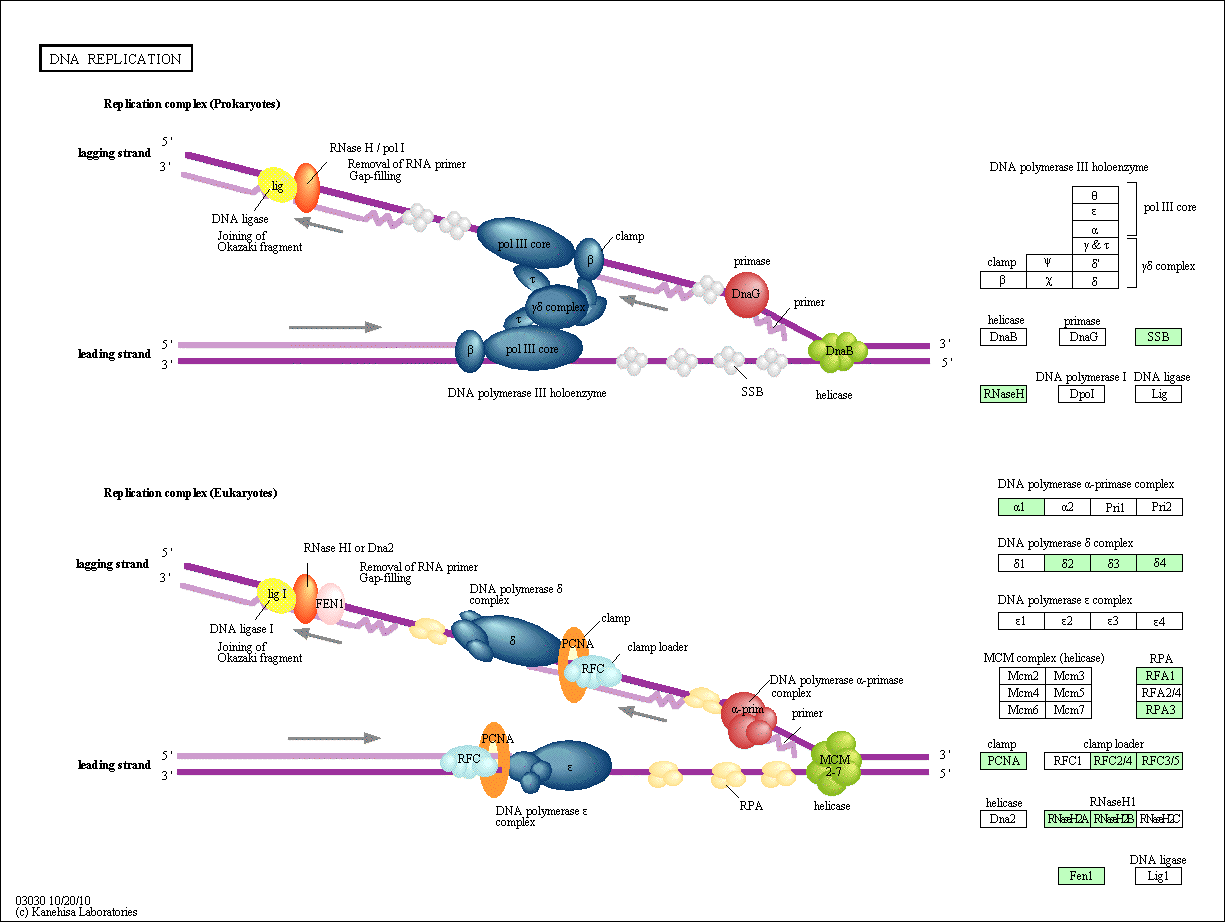

Supplement: Additional file 6 — KEGG annotation results. File contains the KEGG maps where isotigs were successfully mapped. Pathway element in green boxes indicates the components represented by at least, one isotig. Gilthead sea bream isotigs were automatically annotated to KEGG maps using KAAS website [60]. The SBH method, optimized for ESTs annotation, was used against human, chimpanzee, orang-utan, rhesus, mouse, rat, dog, giant panda, cow, pig, horse, opossum, platypus, chicken, clawed frog, zebrafish, fruit fly and nematode pathway databases. [file 1471-2164-13-181-S6.tar › map/map03030.png]

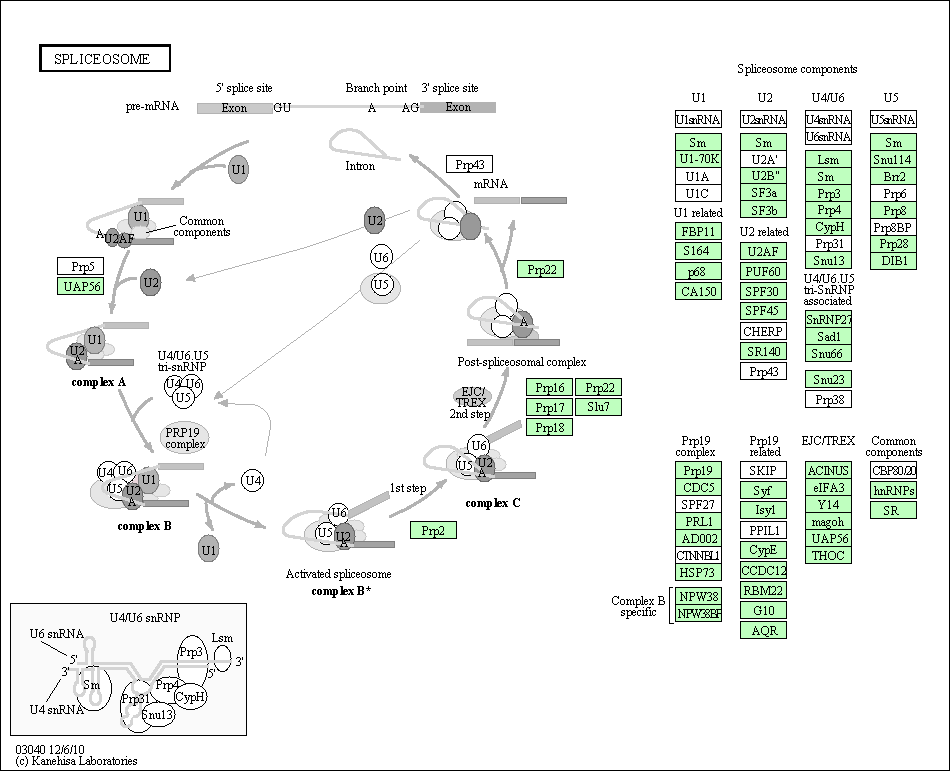

Supplement: Additional file 6 — KEGG annotation results. File contains the KEGG maps where isotigs were successfully mapped. Pathway element in green boxes indicates the components represented by at least, one isotig. Gilthead sea bream isotigs were automatically annotated to KEGG maps using KAAS website [60]. The SBH method, optimized for ESTs annotation, was used against human, chimpanzee, orang-utan, rhesus, mouse, rat, dog, giant panda, cow, pig, horse, opossum, platypus, chicken, clawed frog, zebrafish, fruit fly and nematode pathway databases. [file 1471-2164-13-181-S6.tar › map/map03040.png]

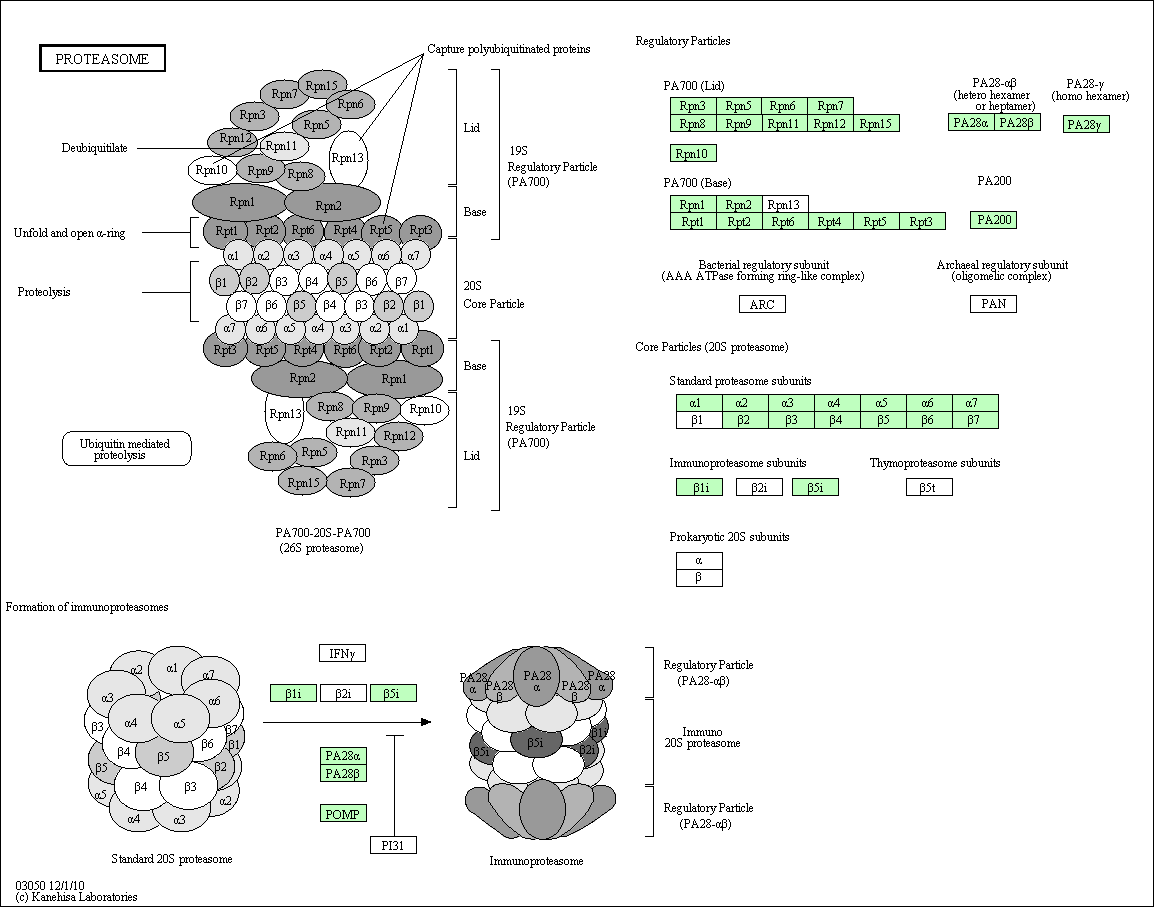

Supplement: Additional file 6 — KEGG annotation results. File contains the KEGG maps where isotigs were successfully mapped. Pathway element in green boxes indicates the components represented by at least, one isotig. Gilthead sea bream isotigs were automatically annotated to KEGG maps using KAAS website [60]. The SBH method, optimized for ESTs annotation, was used against human, chimpanzee, orang-utan, rhesus, mouse, rat, dog, giant panda, cow, pig, horse, opossum, platypus, chicken, clawed frog, zebrafish, fruit fly and nematode pathway databases. [file 1471-2164-13-181-S6.tar › map/map03050.png]

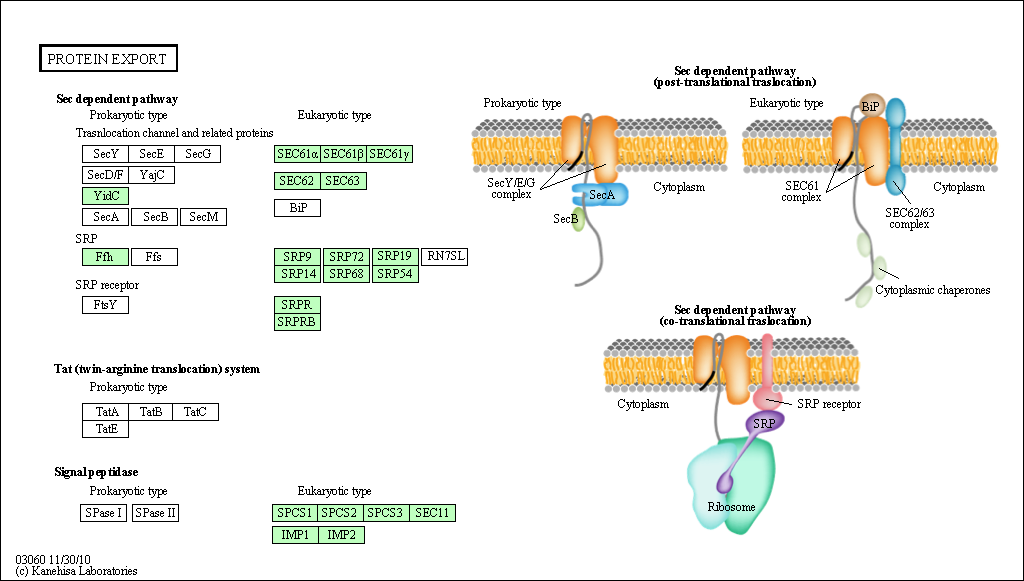

Supplement: Additional file 6 — KEGG annotation results. File contains the KEGG maps where isotigs were successfully mapped. Pathway element in green boxes indicates the components represented by at least, one isotig. Gilthead sea bream isotigs were automatically annotated to KEGG maps using KAAS website [60]. The SBH method, optimized for ESTs annotation, was used against human, chimpanzee, orang-utan, rhesus, mouse, rat, dog, giant panda, cow, pig, horse, opossum, platypus, chicken, clawed frog, zebrafish, fruit fly and nematode pathway databases. [file 1471-2164-13-181-S6.tar › map/map03060.png]

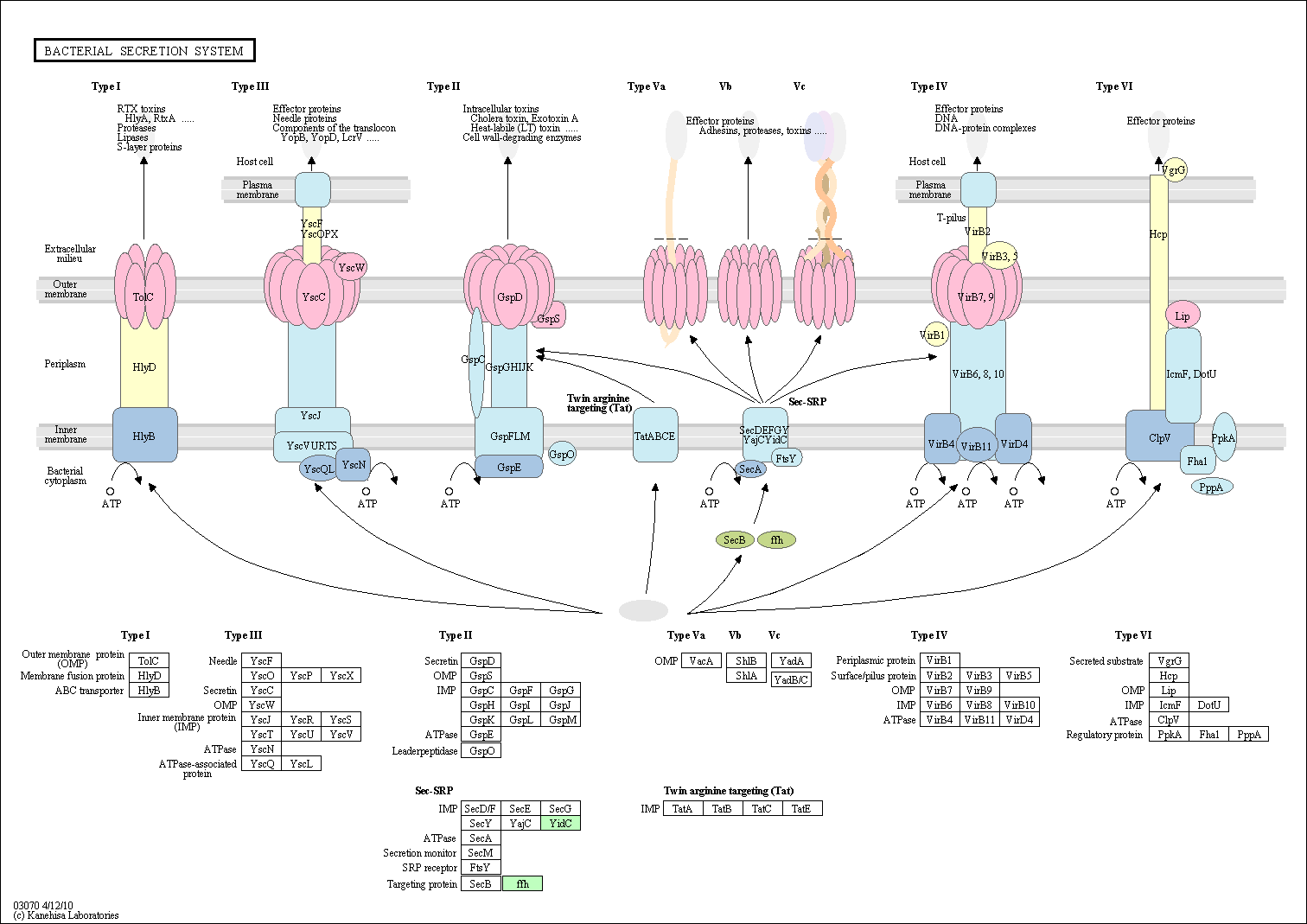

Supplement: Additional file 6 — KEGG annotation results. File contains the KEGG maps where isotigs were successfully mapped. Pathway element in green boxes indicates the components represented by at least, one isotig. Gilthead sea bream isotigs were automatically annotated to KEGG maps using KAAS website [60]. The SBH method, optimized for ESTs annotation, was used against human, chimpanzee, orang-utan, rhesus, mouse, rat, dog, giant panda, cow, pig, horse, opossum, platypus, chicken, clawed frog, zebrafish, fruit fly and nematode pathway databases. [file 1471-2164-13-181-S6.tar › map/map03070.png]

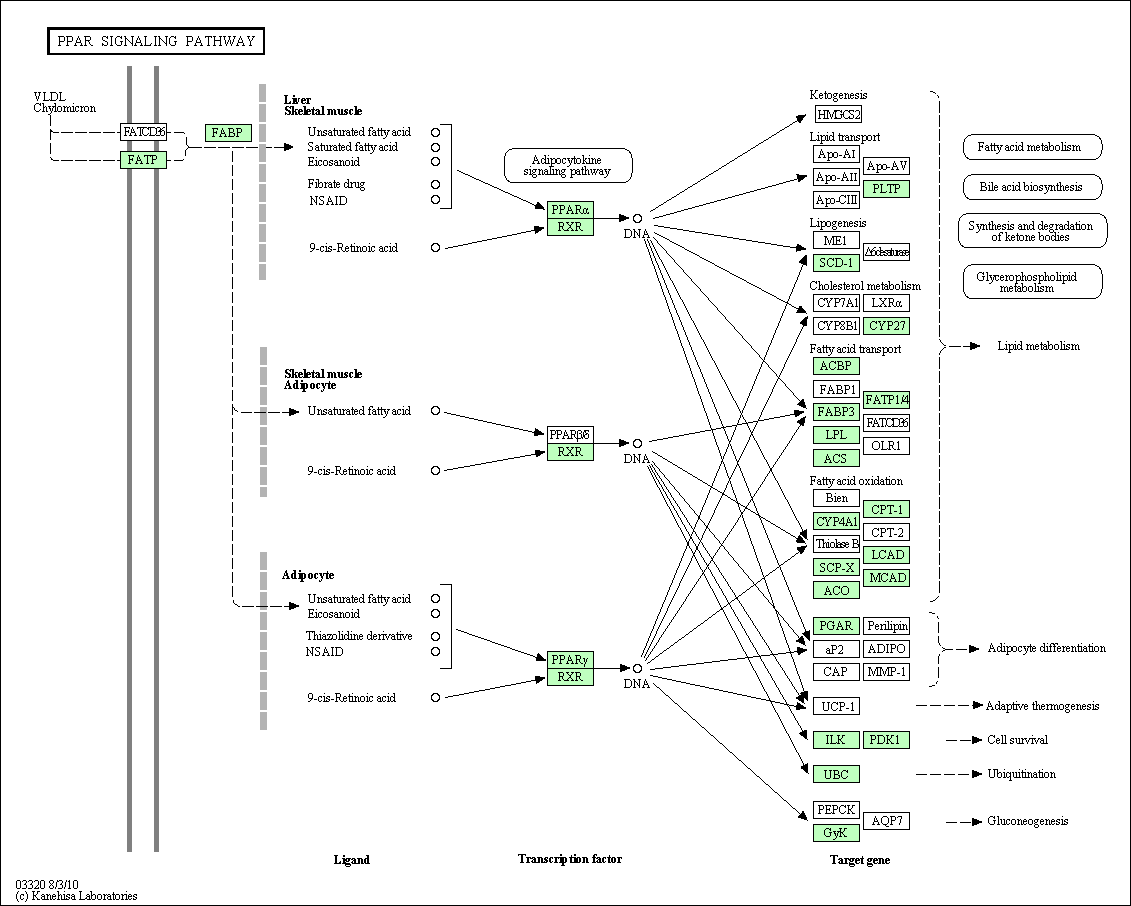

Supplement: Additional file 6 — KEGG annotation results. File contains the KEGG maps where isotigs were successfully mapped. Pathway element in green boxes indicates the components represented by at least, one isotig. Gilthead sea bream isotigs were automatically annotated to KEGG maps using KAAS website [60]. The SBH method, optimized for ESTs annotation, was used against human, chimpanzee, orang-utan, rhesus, mouse, rat, dog, giant panda, cow, pig, horse, opossum, platypus, chicken, clawed frog, zebrafish, fruit fly and nematode pathway databases. [file 1471-2164-13-181-S6.tar › map/map03320.png]

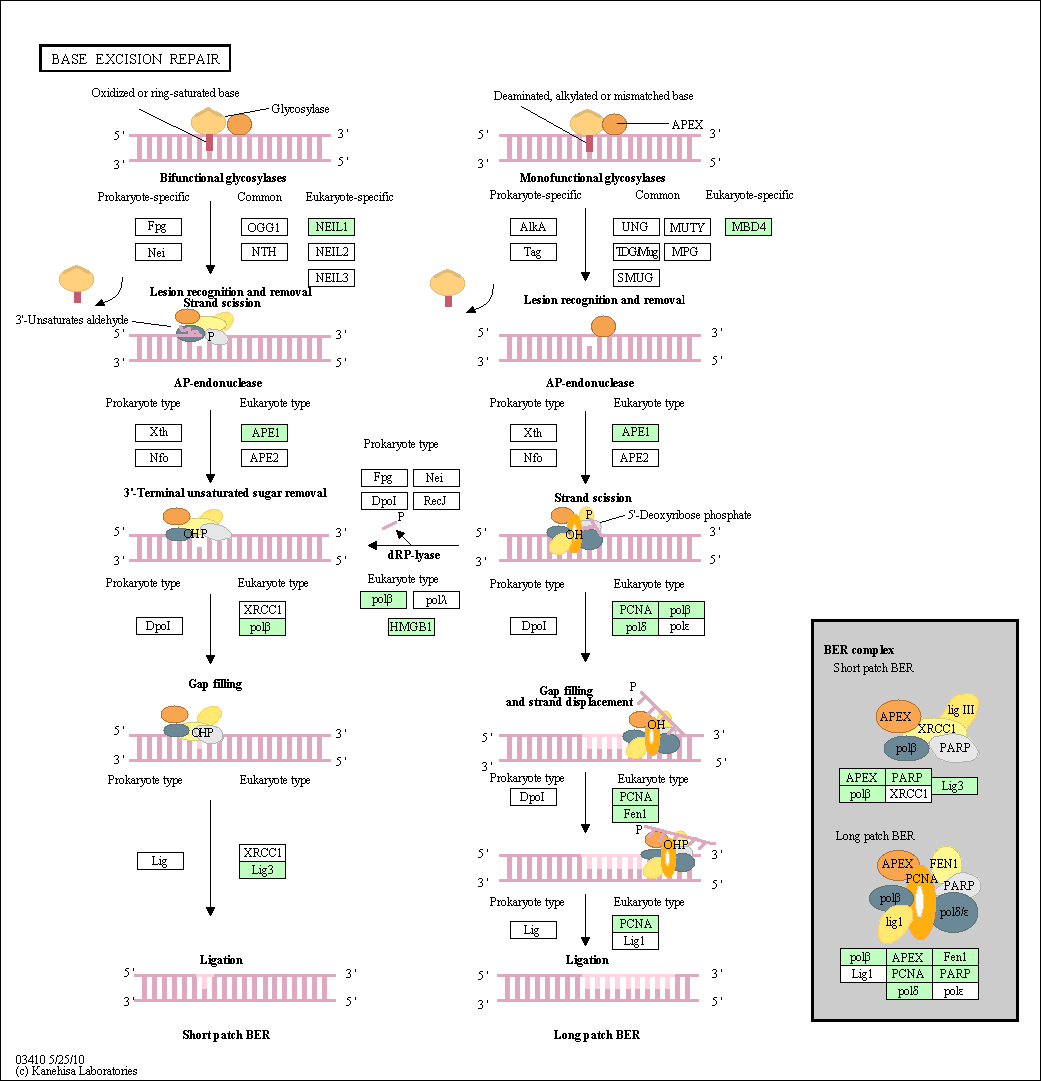

Supplement: Additional file 6 — KEGG annotation results. File contains the KEGG maps where isotigs were successfully mapped. Pathway element in green boxes indicates the components represented by at least, one isotig. Gilthead sea bream isotigs were automatically annotated to KEGG maps using KAAS website [60]. The SBH method, optimized for ESTs annotation, was used against human, chimpanzee, orang-utan, rhesus, mouse, rat, dog, giant panda, cow, pig, horse, opossum, platypus, chicken, clawed frog, zebrafish, fruit fly and nematode pathway databases. [file 1471-2164-13-181-S6.tar › map/map03410.png]

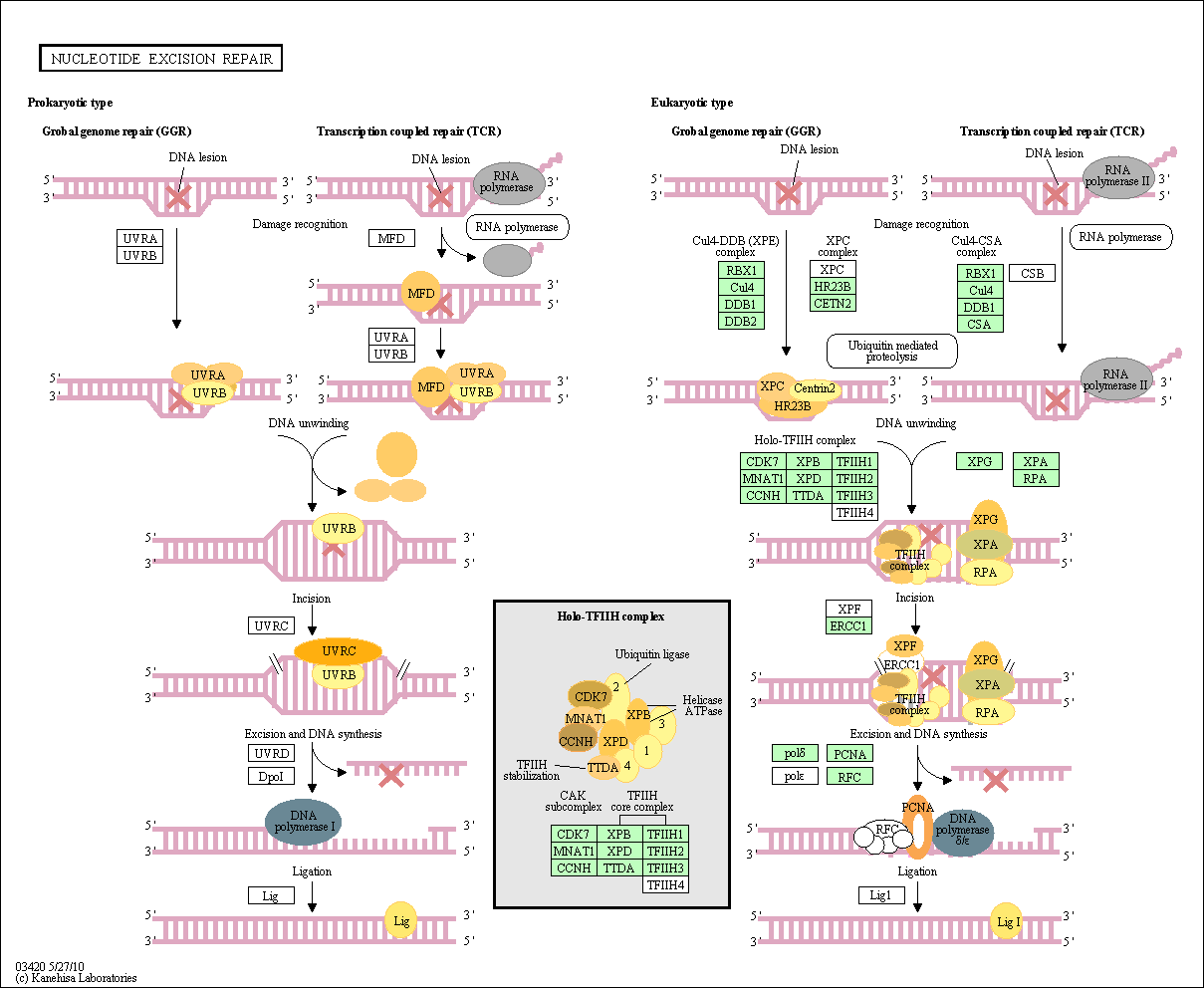

Supplement: Additional file 6 — KEGG annotation results. File contains the KEGG maps where isotigs were successfully mapped. Pathway element in green boxes indicates the components represented by at least, one isotig. Gilthead sea bream isotigs were automatically annotated to KEGG maps using KAAS website [60]. The SBH method, optimized for ESTs annotation, was used against human, chimpanzee, orang-utan, rhesus, mouse, rat, dog, giant panda, cow, pig, horse, opossum, platypus, chicken, clawed frog, zebrafish, fruit fly and nematode pathway databases. [file 1471-2164-13-181-S6.tar › map/map03420.png]

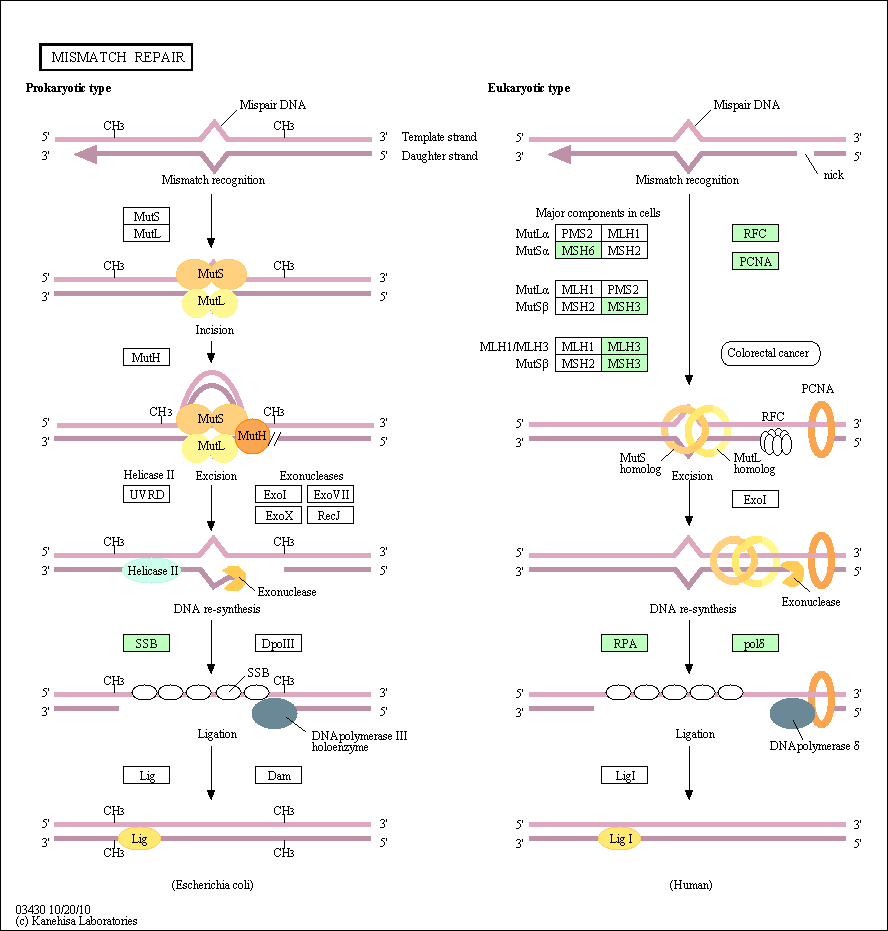

Supplement: Additional file 6 — KEGG annotation results. File contains the KEGG maps where isotigs were successfully mapped. Pathway element in green boxes indicates the components represented by at least, one isotig. Gilthead sea bream isotigs were automatically annotated to KEGG maps using KAAS website [60]. The SBH method, optimized for ESTs annotation, was used against human, chimpanzee, orang-utan, rhesus, mouse, rat, dog, giant panda, cow, pig, horse, opossum, platypus, chicken, clawed frog, zebrafish, fruit fly and nematode pathway databases. [file 1471-2164-13-181-S6.tar › map/map03430.png]

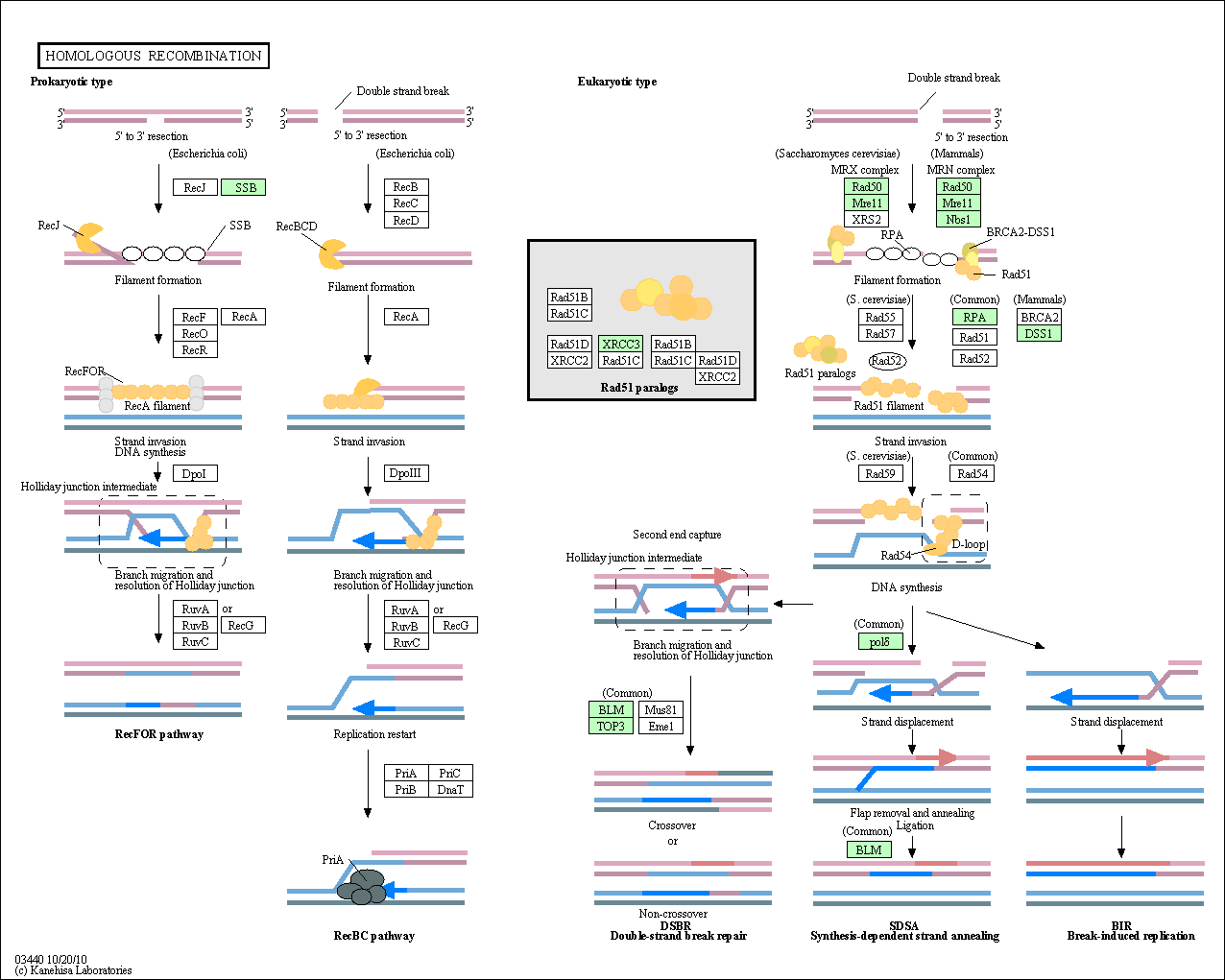

Supplement: Additional file 6 — KEGG annotation results. File contains the KEGG maps where isotigs were successfully mapped. Pathway element in green boxes indicates the components represented by at least, one isotig. Gilthead sea bream isotigs were automatically annotated to KEGG maps using KAAS website [60]. The SBH method, optimized for ESTs annotation, was used against human, chimpanzee, orang-utan, rhesus, mouse, rat, dog, giant panda, cow, pig, horse, opossum, platypus, chicken, clawed frog, zebrafish, fruit fly and nematode pathway databases. [file 1471-2164-13-181-S6.tar › map/map03440.png]

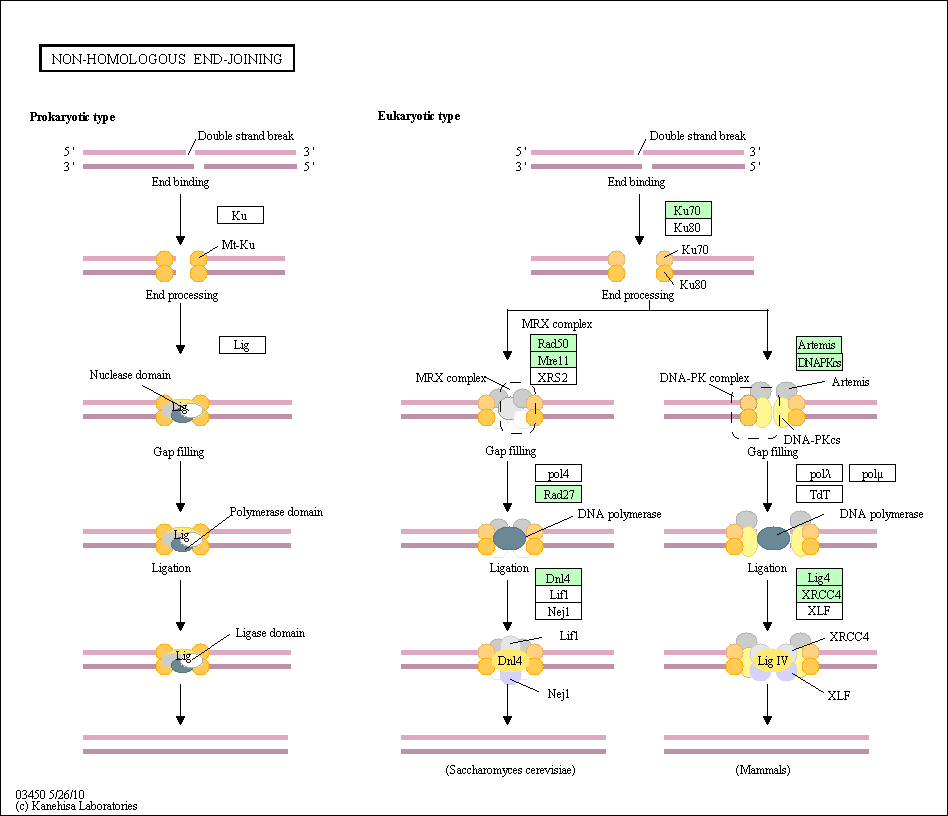

Supplement: Additional file 6 — KEGG annotation results. File contains the KEGG maps where isotigs were successfully mapped. Pathway element in green boxes indicates the components represented by at least, one isotig. Gilthead sea bream isotigs were automatically annotated to KEGG maps using KAAS website [60]. The SBH method, optimized for ESTs annotation, was used against human, chimpanzee, orang-utan, rhesus, mouse, rat, dog, giant panda, cow, pig, horse, opossum, platypus, chicken, clawed frog, zebrafish, fruit fly and nematode pathway databases. [file 1471-2164-13-181-S6.tar › map/map03450.png]

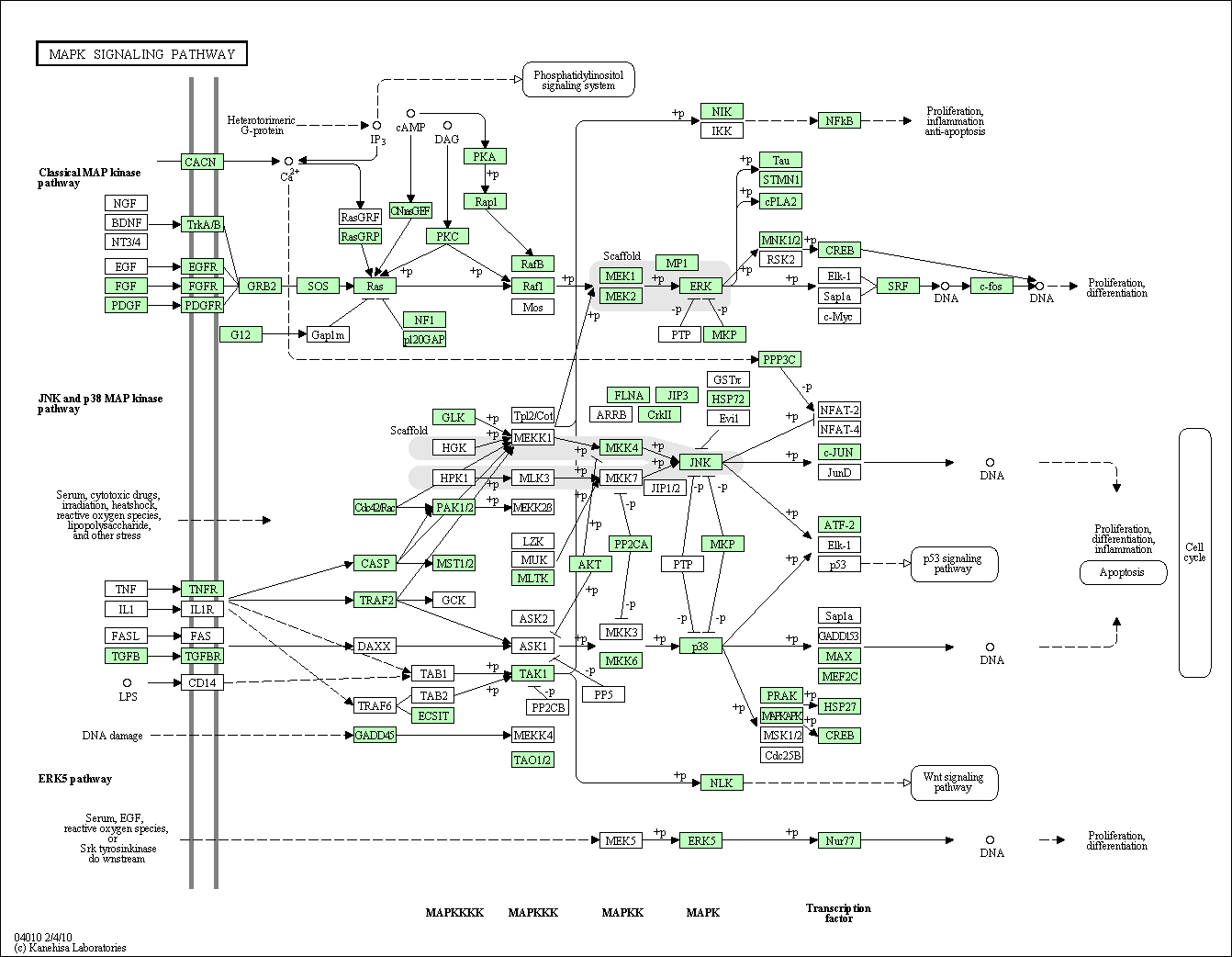

Supplement: Additional file 6 — KEGG annotation results. File contains the KEGG maps where isotigs were successfully mapped. Pathway element in green boxes indicates the components represented by at least, one isotig. Gilthead sea bream isotigs were automatically annotated to KEGG maps using KAAS website [60]. The SBH method, optimized for ESTs annotation, was used against human, chimpanzee, orang-utan, rhesus, mouse, rat, dog, giant panda, cow, pig, horse, opossum, platypus, chicken, clawed frog, zebrafish, fruit fly and nematode pathway databases. [file 1471-2164-13-181-S6.tar › map/map04010.png]

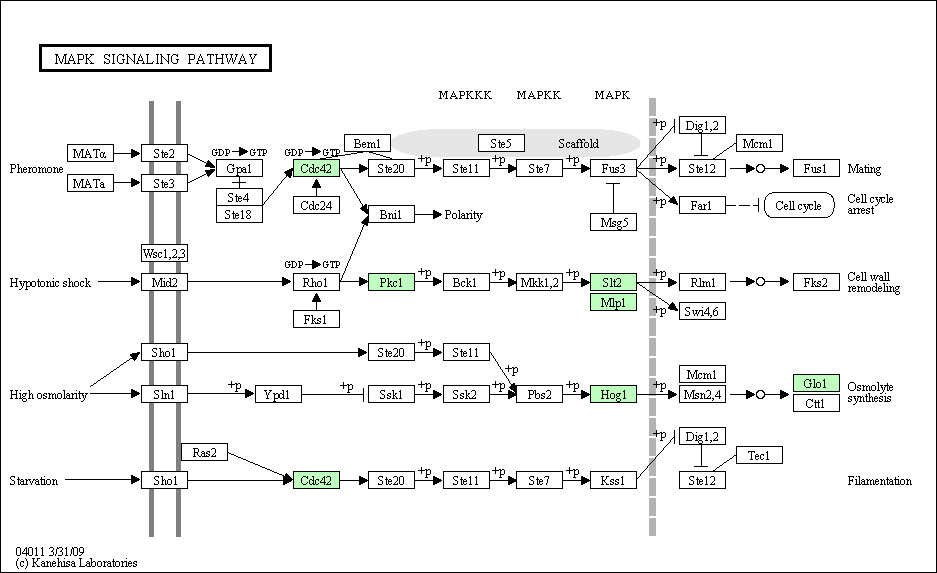

Supplement: Additional file 6 — KEGG annotation results. File contains the KEGG maps where isotigs were successfully mapped. Pathway element in green boxes indicates the components represented by at least, one isotig. Gilthead sea bream isotigs were automatically annotated to KEGG maps using KAAS website [60]. The SBH method, optimized for ESTs annotation, was used against human, chimpanzee, orang-utan, rhesus, mouse, rat, dog, giant panda, cow, pig, horse, opossum, platypus, chicken, clawed frog, zebrafish, fruit fly and nematode pathway databases. [file 1471-2164-13-181-S6.tar › map/map04011.png]

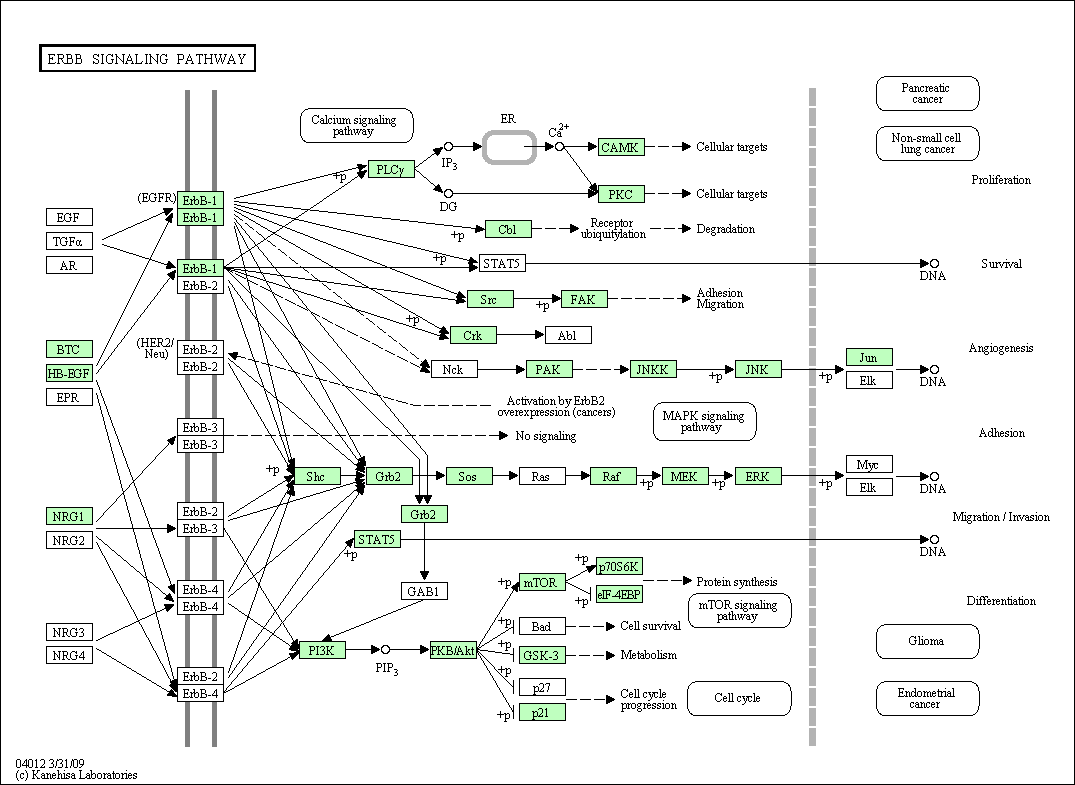

Supplement: Additional file 6 — KEGG annotation results. File contains the KEGG maps where isotigs were successfully mapped. Pathway element in green boxes indicates the components represented by at least, one isotig. Gilthead sea bream isotigs were automatically annotated to KEGG maps using KAAS website [60]. The SBH method, optimized for ESTs annotation, was used against human, chimpanzee, orang-utan, rhesus, mouse, rat, dog, giant panda, cow, pig, horse, opossum, platypus, chicken, clawed frog, zebrafish, fruit fly and nematode pathway databases. [file 1471-2164-13-181-S6.tar › map/map04012.png]

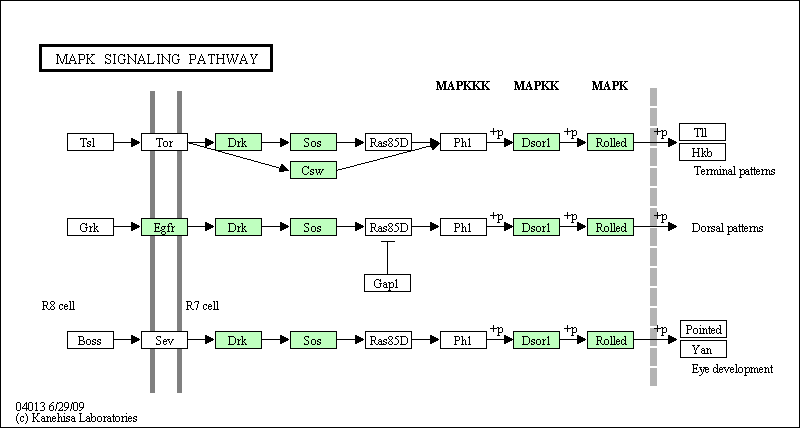

Supplement: Additional file 6 — KEGG annotation results. File contains the KEGG maps where isotigs were successfully mapped. Pathway element in green boxes indicates the components represented by at least, one isotig. Gilthead sea bream isotigs were automatically annotated to KEGG maps using KAAS website [60]. The SBH method, optimized for ESTs annotation, was used against human, chimpanzee, orang-utan, rhesus, mouse, rat, dog, giant panda, cow, pig, horse, opossum, platypus, chicken, clawed frog, zebrafish, fruit fly and nematode pathway databases. [file 1471-2164-13-181-S6.tar › map/map04013.png]

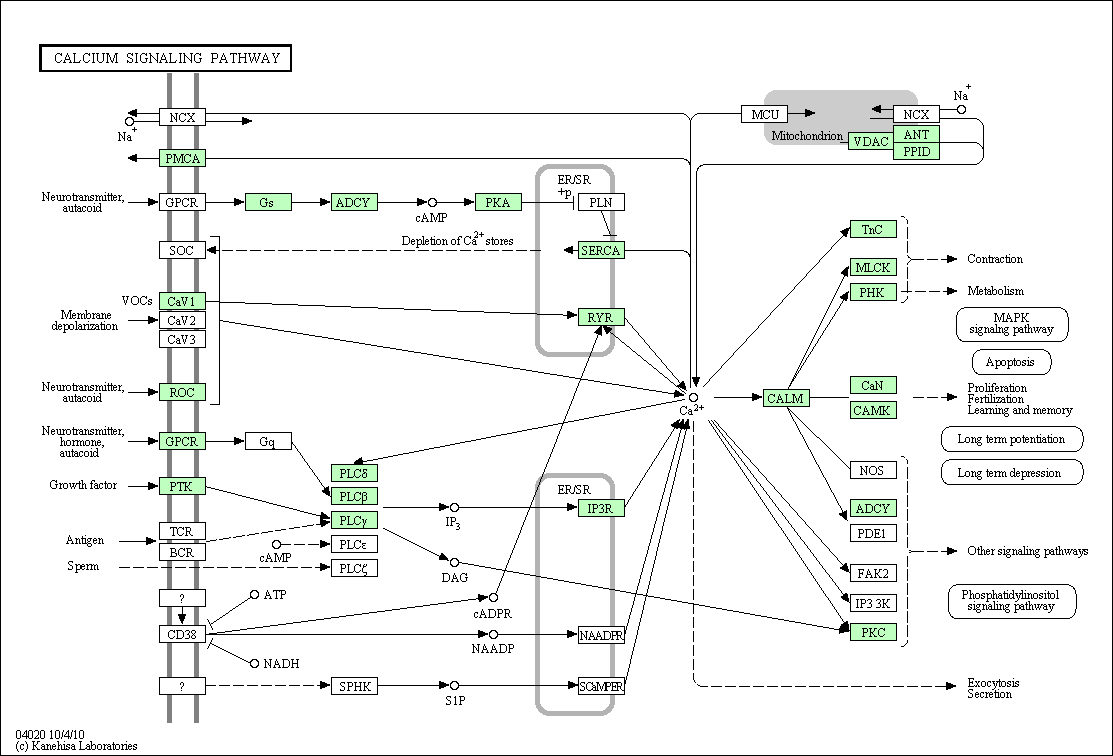

Supplement: Additional file 6 — KEGG annotation results. File contains the KEGG maps where isotigs were successfully mapped. Pathway element in green boxes indicates the components represented by at least, one isotig. Gilthead sea bream isotigs were automatically annotated to KEGG maps using KAAS website [60]. The SBH method, optimized for ESTs annotation, was used against human, chimpanzee, orang-utan, rhesus, mouse, rat, dog, giant panda, cow, pig, horse, opossum, platypus, chicken, clawed frog, zebrafish, fruit fly and nematode pathway databases. [file 1471-2164-13-181-S6.tar › map/map04020.png]

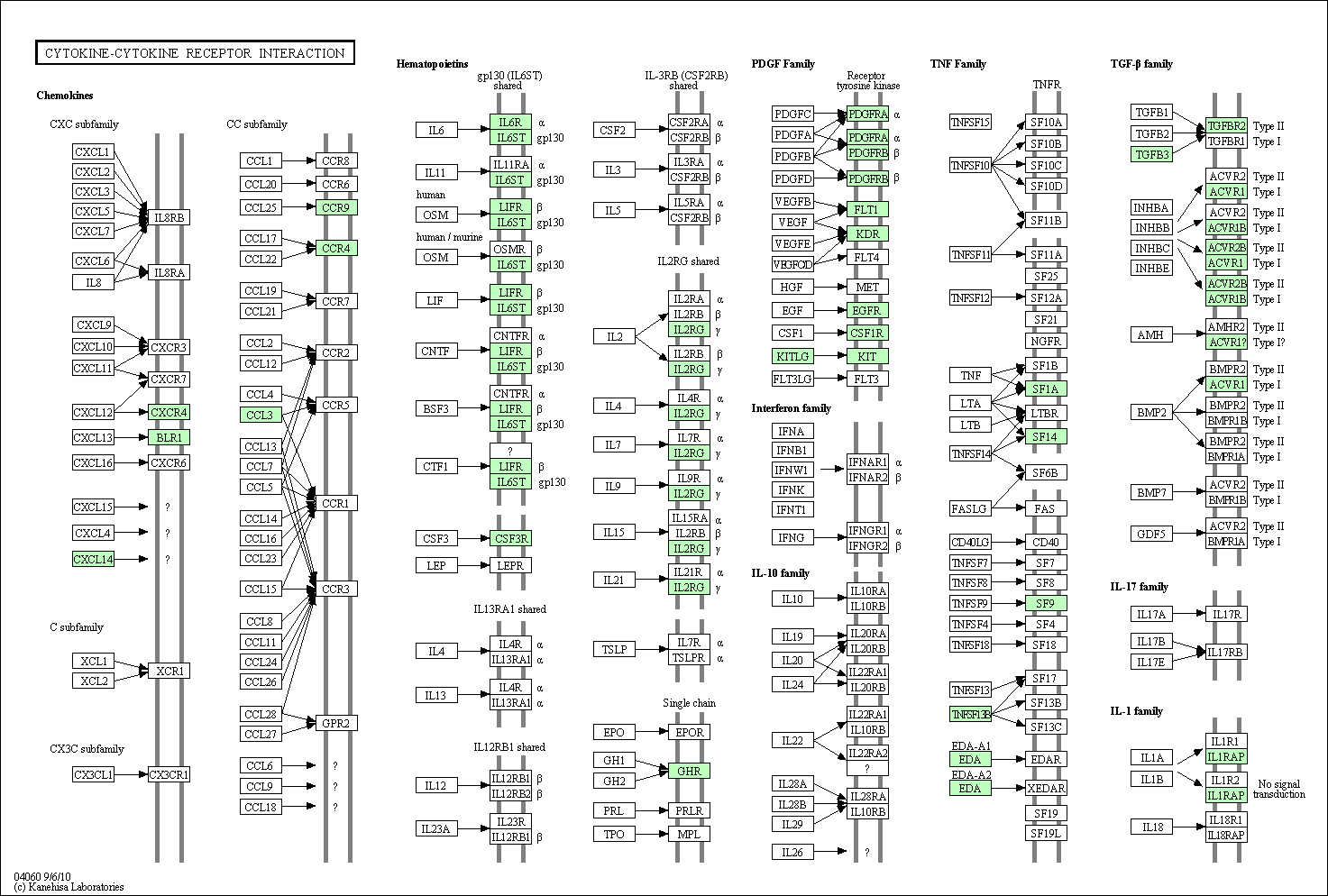

Supplement: Additional file 6 — KEGG annotation results. File contains the KEGG maps where isotigs were successfully mapped. Pathway element in green boxes indicates the components represented by at least, one isotig. Gilthead sea bream isotigs were automatically annotated to KEGG maps using KAAS website [60]. The SBH method, optimized for ESTs annotation, was used against human, chimpanzee, orang-utan, rhesus, mouse, rat, dog, giant panda, cow, pig, horse, opossum, platypus, chicken, clawed frog, zebrafish, fruit fly and nematode pathway databases. [file 1471-2164-13-181-S6.tar › map/map04060.png]

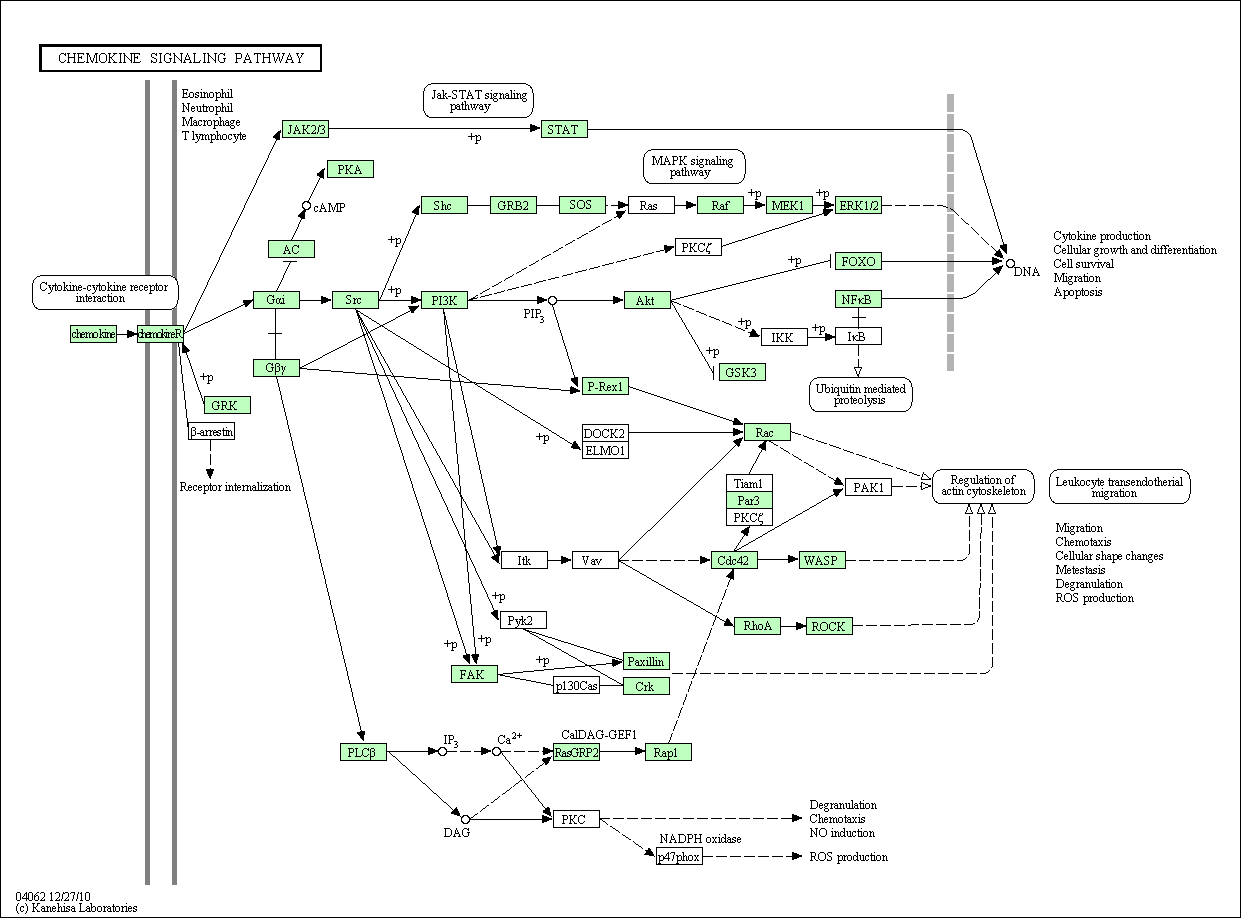

Supplement: Additional file 6 — KEGG annotation results. File contains the KEGG maps where isotigs were successfully mapped. Pathway element in green boxes indicates the components represented by at least, one isotig. Gilthead sea bream isotigs were automatically annotated to KEGG maps using KAAS website [60]. The SBH method, optimized for ESTs annotation, was used against human, chimpanzee, orang-utan, rhesus, mouse, rat, dog, giant panda, cow, pig, horse, opossum, platypus, chicken, clawed frog, zebrafish, fruit fly and nematode pathway databases. [file 1471-2164-13-181-S6.tar › map/map04062.png]

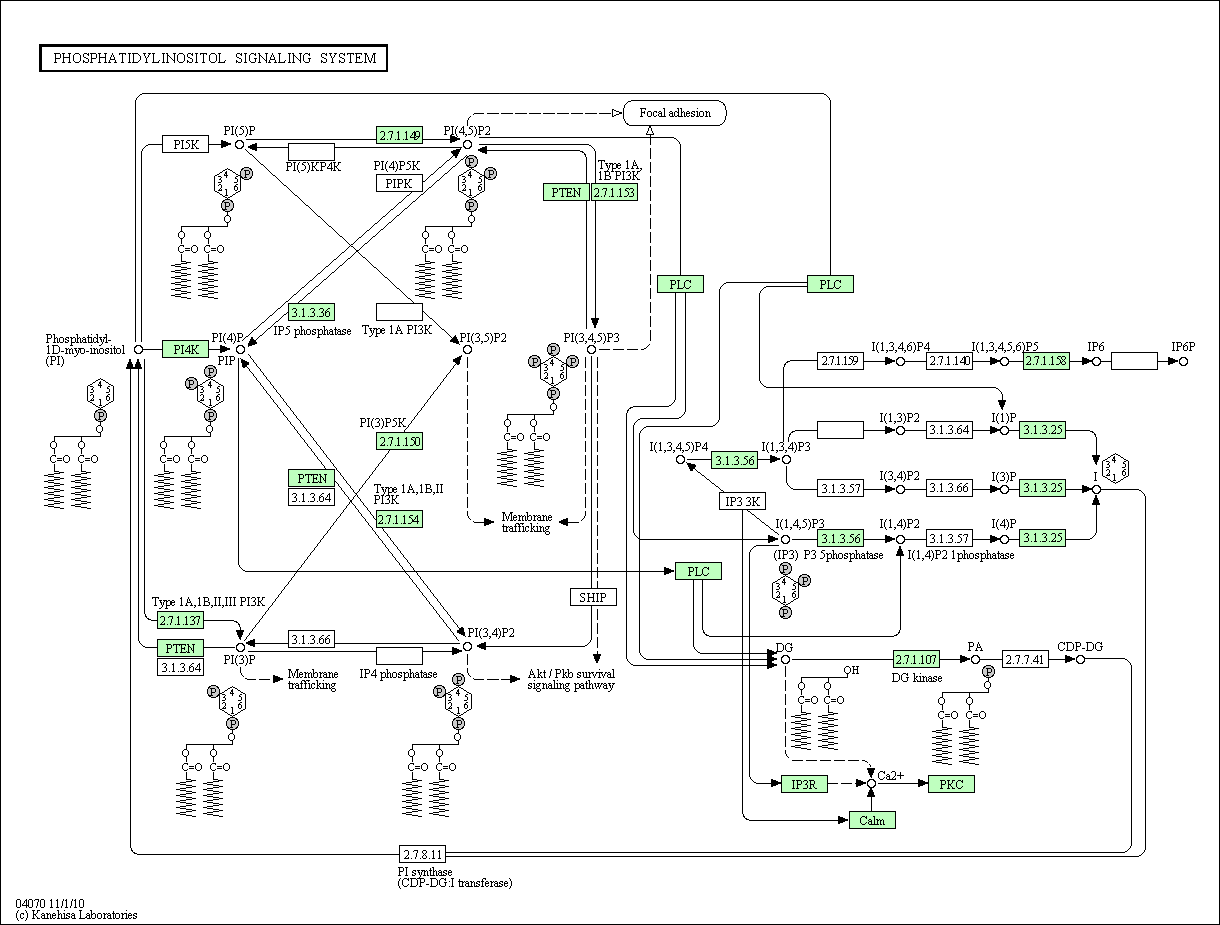

Supplement: Additional file 6 — KEGG annotation results. File contains the KEGG maps where isotigs were successfully mapped. Pathway element in green boxes indicates the components represented by at least, one isotig. Gilthead sea bream isotigs were automatically annotated to KEGG maps using KAAS website [60]. The SBH method, optimized for ESTs annotation, was used against human, chimpanzee, orang-utan, rhesus, mouse, rat, dog, giant panda, cow, pig, horse, opossum, platypus, chicken, clawed frog, zebrafish, fruit fly and nematode pathway databases. [file 1471-2164-13-181-S6.tar › map/map04070.png]

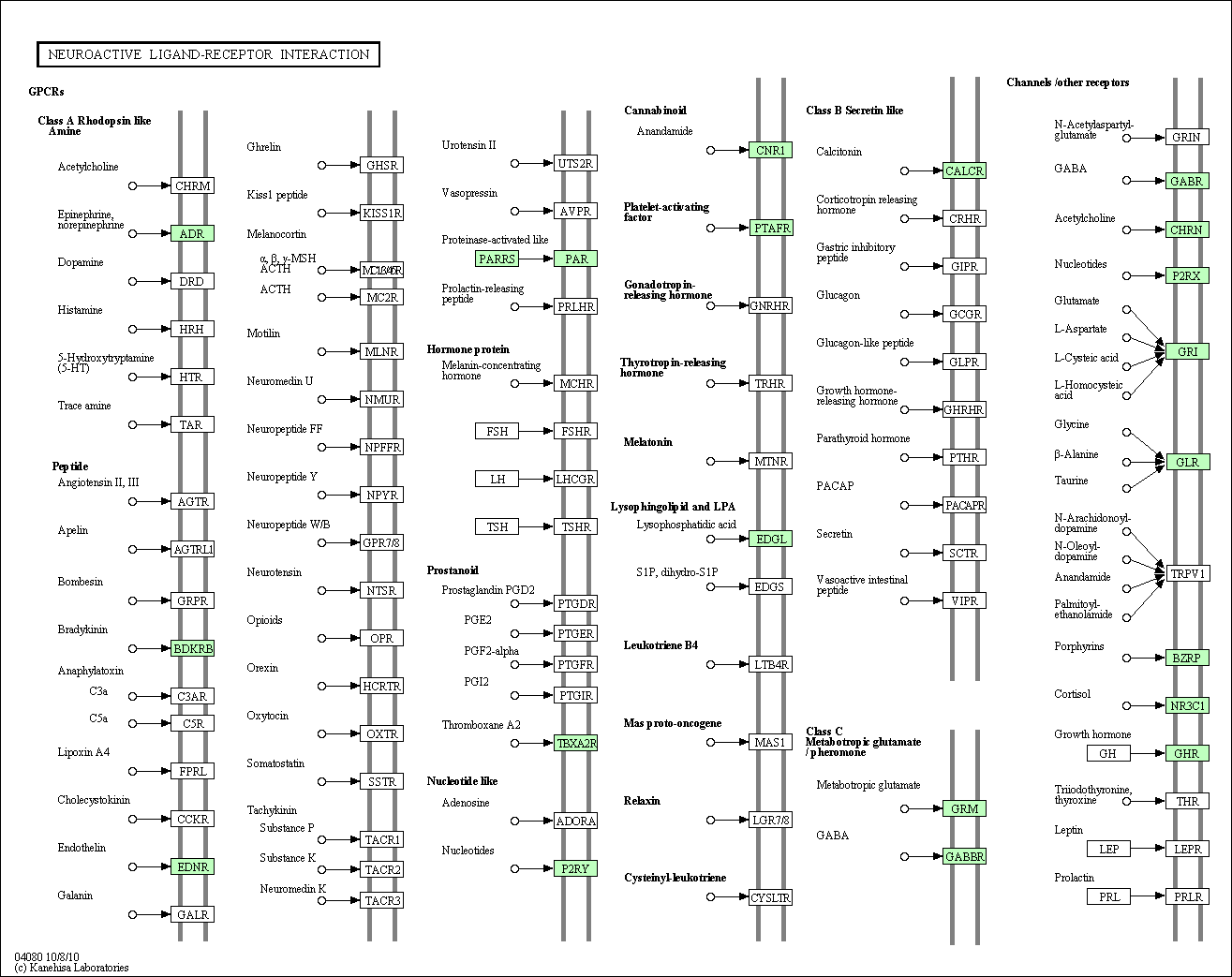

Supplement: Additional file 6 — KEGG annotation results. File contains the KEGG maps where isotigs were successfully mapped. Pathway element in green boxes indicates the components represented by at least, one isotig. Gilthead sea bream isotigs were automatically annotated to KEGG maps using KAAS website [60]. The SBH method, optimized for ESTs annotation, was used against human, chimpanzee, orang-utan, rhesus, mouse, rat, dog, giant panda, cow, pig, horse, opossum, platypus, chicken, clawed frog, zebrafish, fruit fly and nematode pathway databases. [file 1471-2164-13-181-S6.tar › map/map04080.png]

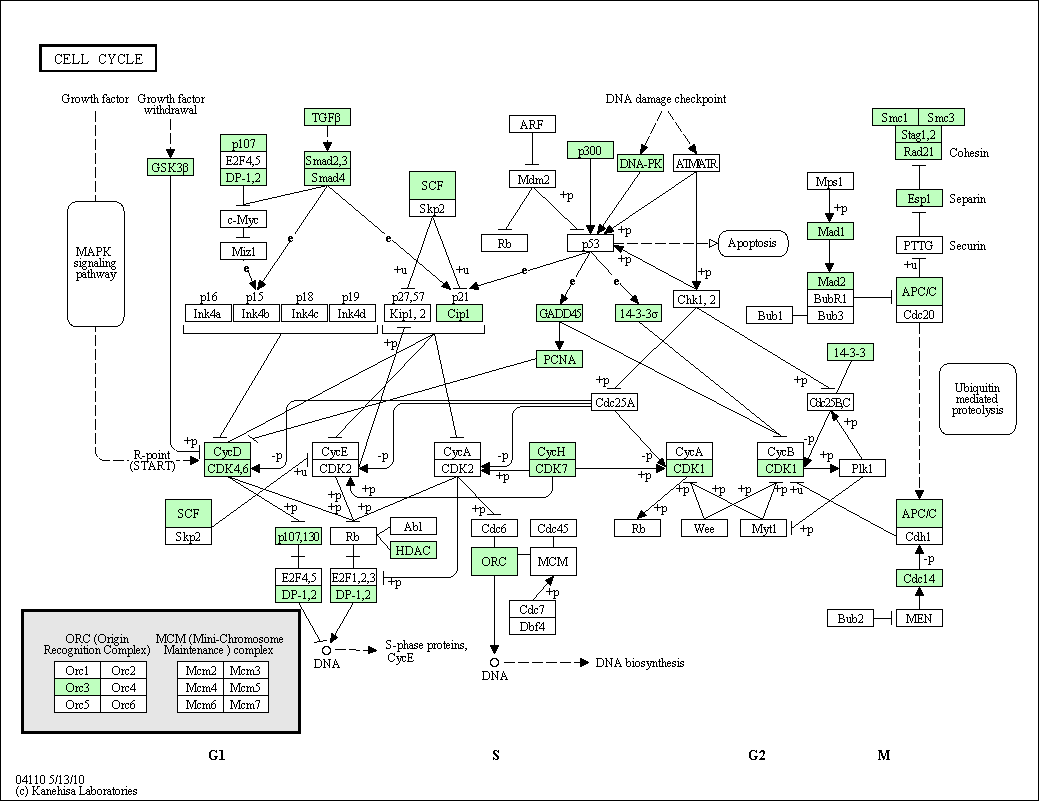

Supplement: Additional file 6 — KEGG annotation results. File contains the KEGG maps where isotigs were successfully mapped. Pathway element in green boxes indicates the components represented by at least, one isotig. Gilthead sea bream isotigs were automatically annotated to KEGG maps using KAAS website [60]. The SBH method, optimized for ESTs annotation, was used against human, chimpanzee, orang-utan, rhesus, mouse, rat, dog, giant panda, cow, pig, horse, opossum, platypus, chicken, clawed frog, zebrafish, fruit fly and nematode pathway databases. [file 1471-2164-13-181-S6.tar › map/map04110.png]

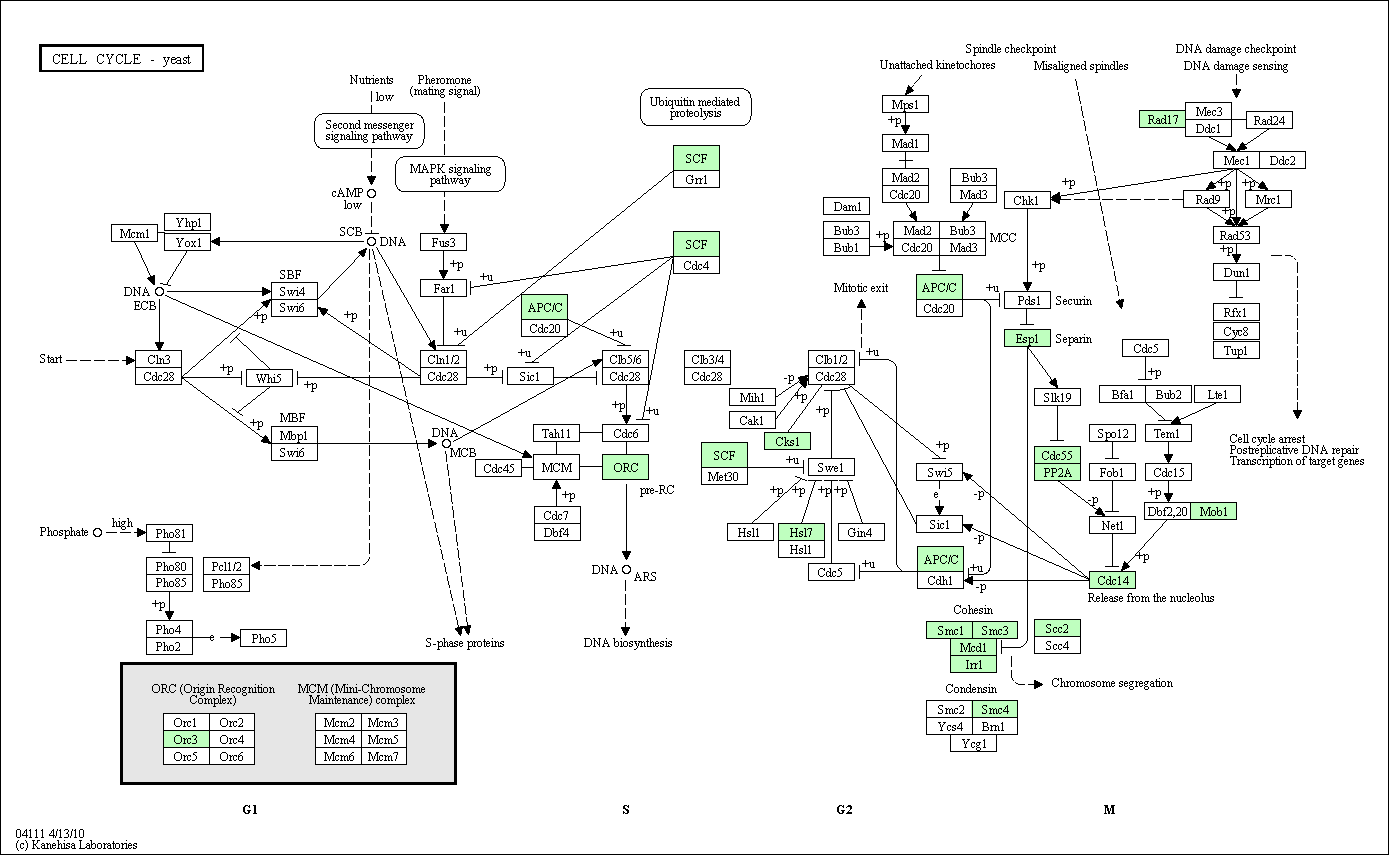

Supplement: Additional file 6 — KEGG annotation results. File contains the KEGG maps where isotigs were successfully mapped. Pathway element in green boxes indicates the components represented by at least, one isotig. Gilthead sea bream isotigs were automatically annotated to KEGG maps using KAAS website [60]. The SBH method, optimized for ESTs annotation, was used against human, chimpanzee, orang-utan, rhesus, mouse, rat, dog, giant panda, cow, pig, horse, opossum, platypus, chicken, clawed frog, zebrafish, fruit fly and nematode pathway databases. [file 1471-2164-13-181-S6.tar › map/map04111.png]

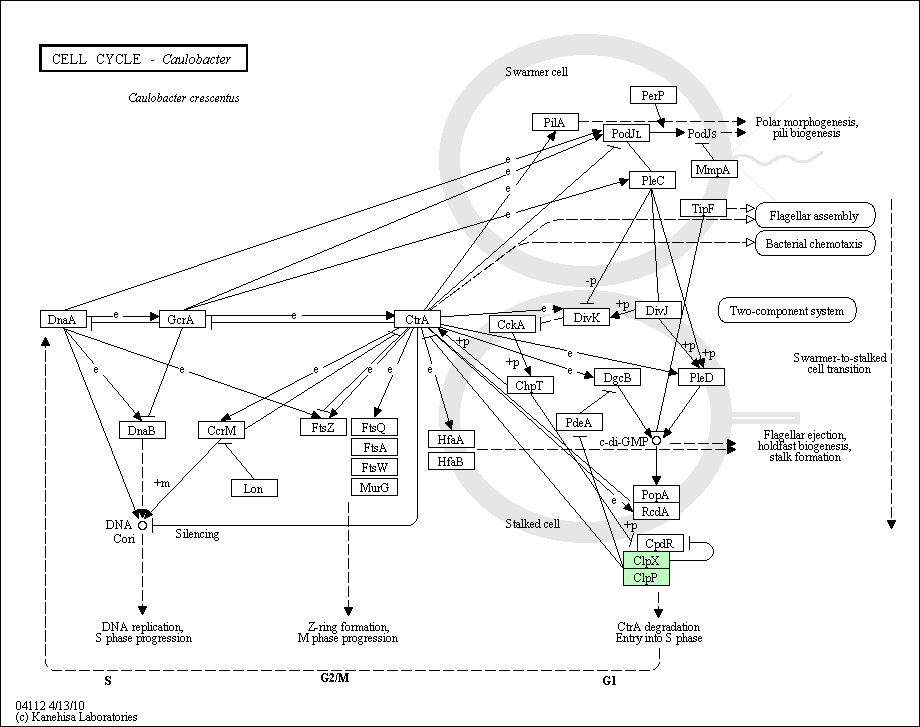

Supplement: Additional file 6 — KEGG annotation results. File contains the KEGG maps where isotigs were successfully mapped. Pathway element in green boxes indicates the components represented by at least, one isotig. Gilthead sea bream isotigs were automatically annotated to KEGG maps using KAAS website [60]. The SBH method, optimized for ESTs annotation, was used against human, chimpanzee, orang-utan, rhesus, mouse, rat, dog, giant panda, cow, pig, horse, opossum, platypus, chicken, clawed frog, zebrafish, fruit fly and nematode pathway databases. [file 1471-2164-13-181-S6.tar › map/map04112.png]

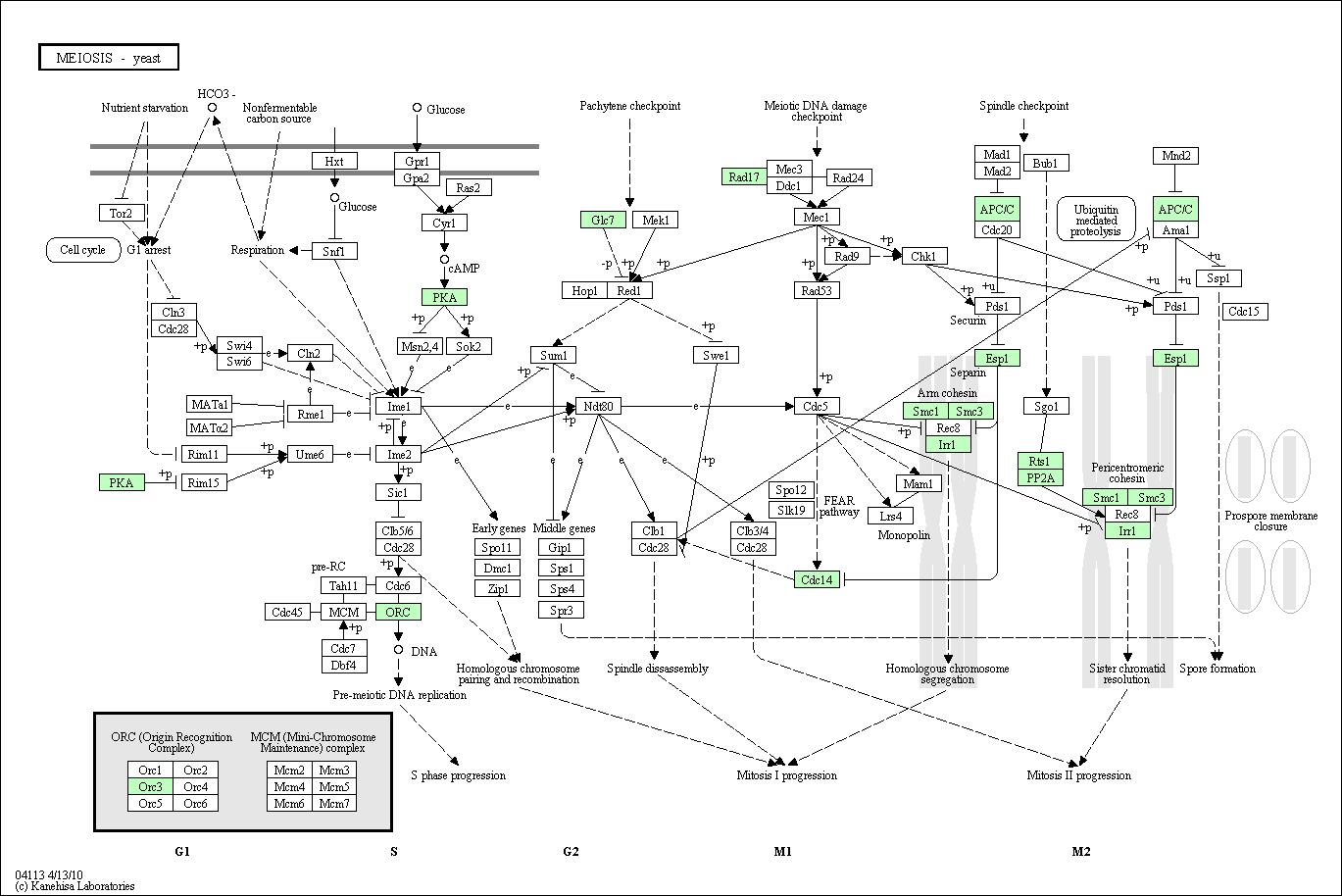

Supplement: Additional file 6 — KEGG annotation results. File contains the KEGG maps where isotigs were successfully mapped. Pathway element in green boxes indicates the components represented by at least, one isotig. Gilthead sea bream isotigs were automatically annotated to KEGG maps using KAAS website [60]. The SBH method, optimized for ESTs annotation, was used against human, chimpanzee, orang-utan, rhesus, mouse, rat, dog, giant panda, cow, pig, horse, opossum, platypus, chicken, clawed frog, zebrafish, fruit fly and nematode pathway databases. [file 1471-2164-13-181-S6.tar › map/map04113.png]

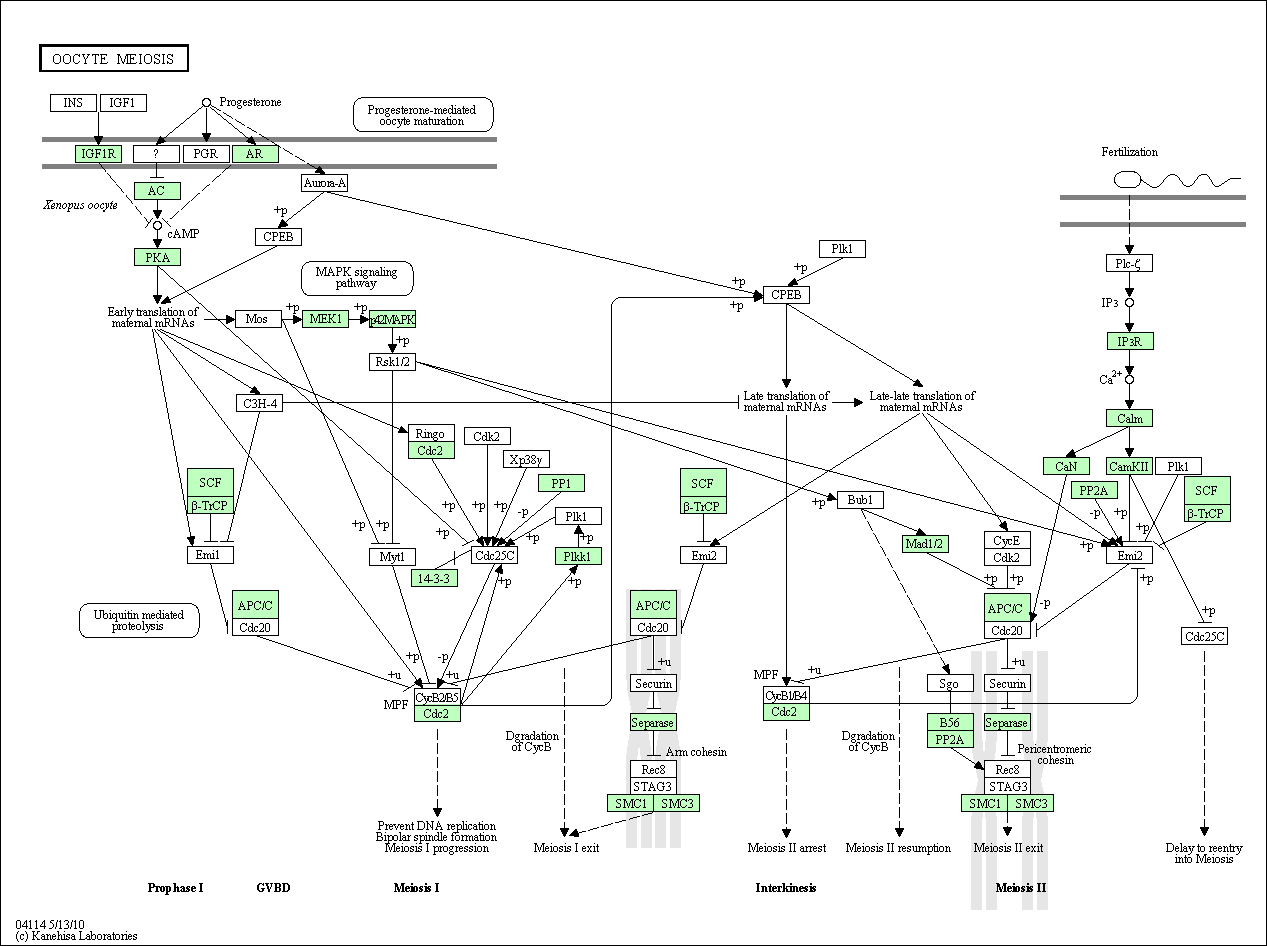

Supplement: Additional file 6 — KEGG annotation results. File contains the KEGG maps where isotigs were successfully mapped. Pathway element in green boxes indicates the components represented by at least, one isotig. Gilthead sea bream isotigs were automatically annotated to KEGG maps using KAAS website [60]. The SBH method, optimized for ESTs annotation, was used against human, chimpanzee, orang-utan, rhesus, mouse, rat, dog, giant panda, cow, pig, horse, opossum, platypus, chicken, clawed frog, zebrafish, fruit fly and nematode pathway databases. [file 1471-2164-13-181-S6.tar › map/map04114.png]

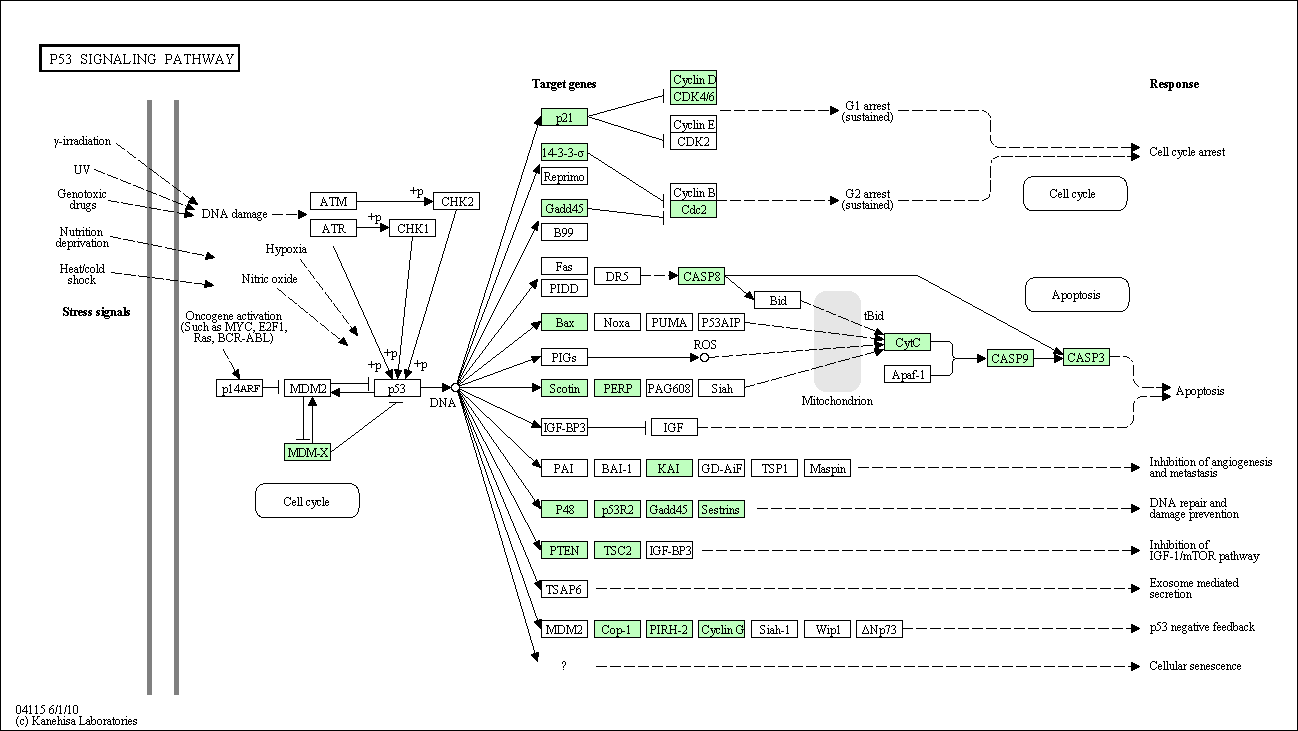

Supplement: Additional file 6 — KEGG annotation results. File contains the KEGG maps where isotigs were successfully mapped. Pathway element in green boxes indicates the components represented by at least, one isotig. Gilthead sea bream isotigs were automatically annotated to KEGG maps using KAAS website [60]. The SBH method, optimized for ESTs annotation, was used against human, chimpanzee, orang-utan, rhesus, mouse, rat, dog, giant panda, cow, pig, horse, opossum, platypus, chicken, clawed frog, zebrafish, fruit fly and nematode pathway databases. [file 1471-2164-13-181-S6.tar › map/map04115.png]

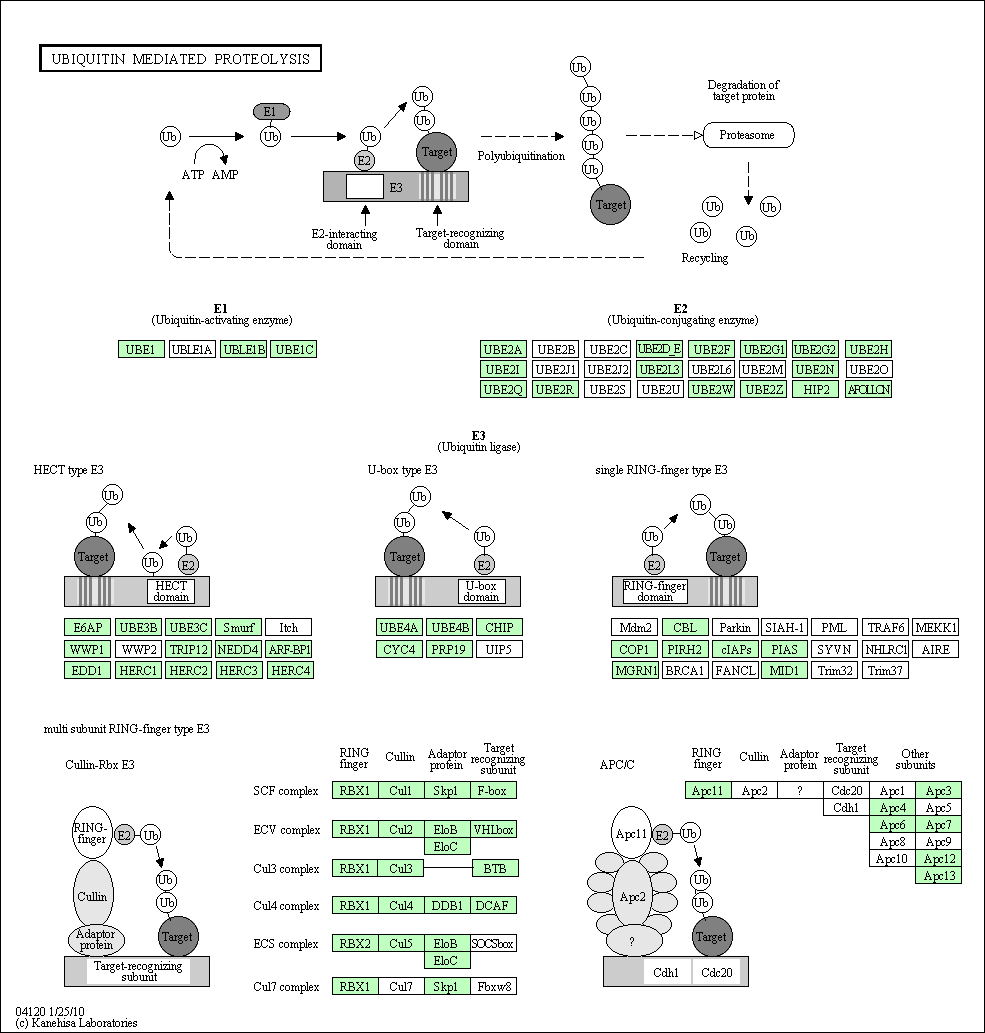

Supplement: Additional file 6 — KEGG annotation results. File contains the KEGG maps where isotigs were successfully mapped. Pathway element in green boxes indicates the components represented by at least, one isotig. Gilthead sea bream isotigs were automatically annotated to KEGG maps using KAAS website [60]. The SBH method, optimized for ESTs annotation, was used against human, chimpanzee, orang-utan, rhesus, mouse, rat, dog, giant panda, cow, pig, horse, opossum, platypus, chicken, clawed frog, zebrafish, fruit fly and nematode pathway databases. [file 1471-2164-13-181-S6.tar › map/map04120.png]

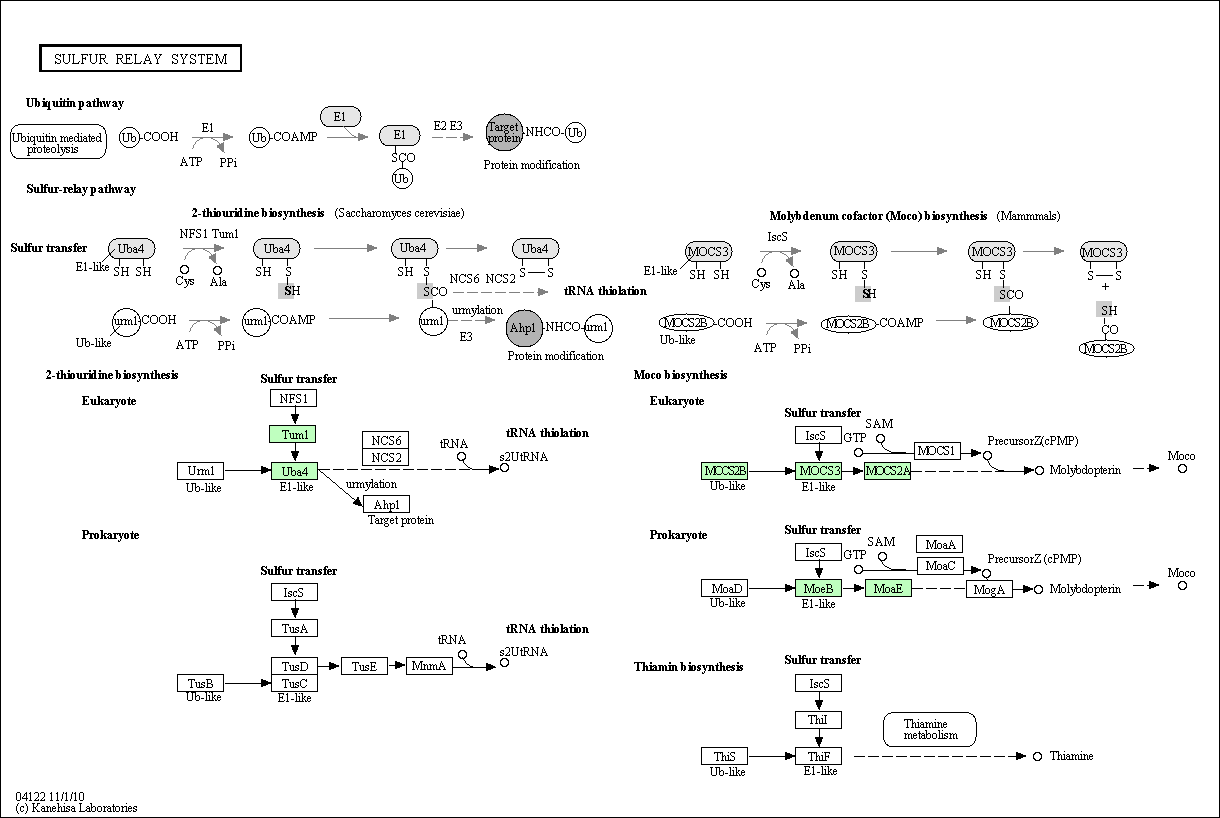

Supplement: Additional file 6 — KEGG annotation results. File contains the KEGG maps where isotigs were successfully mapped. Pathway element in green boxes indicates the components represented by at least, one isotig. Gilthead sea bream isotigs were automatically annotated to KEGG maps using KAAS website [60]. The SBH method, optimized for ESTs annotation, was used against human, chimpanzee, orang-utan, rhesus, mouse, rat, dog, giant panda, cow, pig, horse, opossum, platypus, chicken, clawed frog, zebrafish, fruit fly and nematode pathway databases. [file 1471-2164-13-181-S6.tar › map/map04122.png]

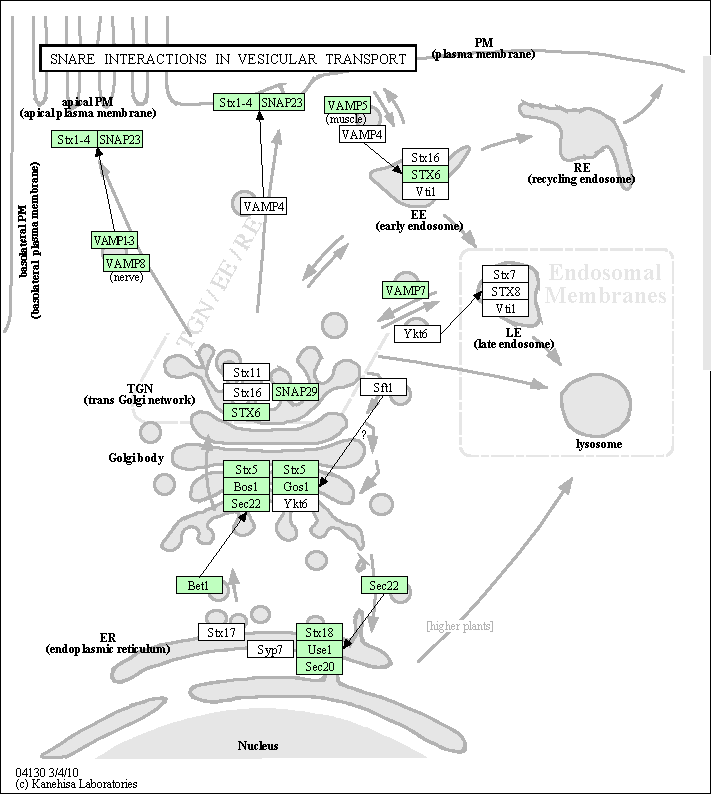

Supplement: Additional file 6 — KEGG annotation results. File contains the KEGG maps where isotigs were successfully mapped. Pathway element in green boxes indicates the components represented by at least, one isotig. Gilthead sea bream isotigs were automatically annotated to KEGG maps using KAAS website [60]. The SBH method, optimized for ESTs annotation, was used against human, chimpanzee, orang-utan, rhesus, mouse, rat, dog, giant panda, cow, pig, horse, opossum, platypus, chicken, clawed frog, zebrafish, fruit fly and nematode pathway databases. [file 1471-2164-13-181-S6.tar › map/map04130.png]

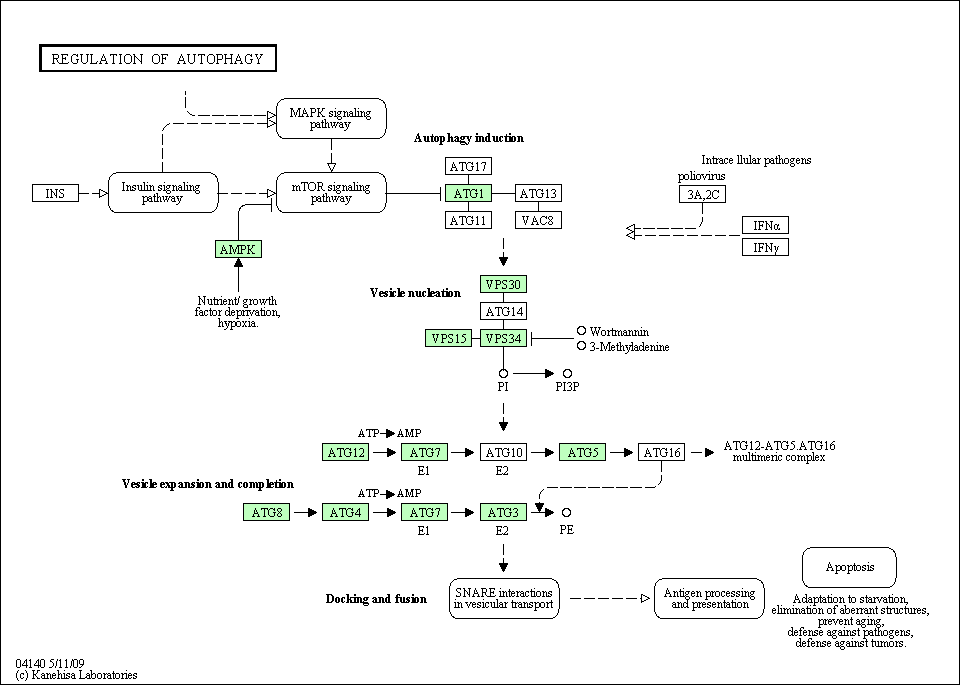

Supplement: Additional file 6 — KEGG annotation results. File contains the KEGG maps where isotigs were successfully mapped. Pathway element in green boxes indicates the components represented by at least, one isotig. Gilthead sea bream isotigs were automatically annotated to KEGG maps using KAAS website [60]. The SBH method, optimized for ESTs annotation, was used against human, chimpanzee, orang-utan, rhesus, mouse, rat, dog, giant panda, cow, pig, horse, opossum, platypus, chicken, clawed frog, zebrafish, fruit fly and nematode pathway databases. [file 1471-2164-13-181-S6.tar › map/map04140.png]

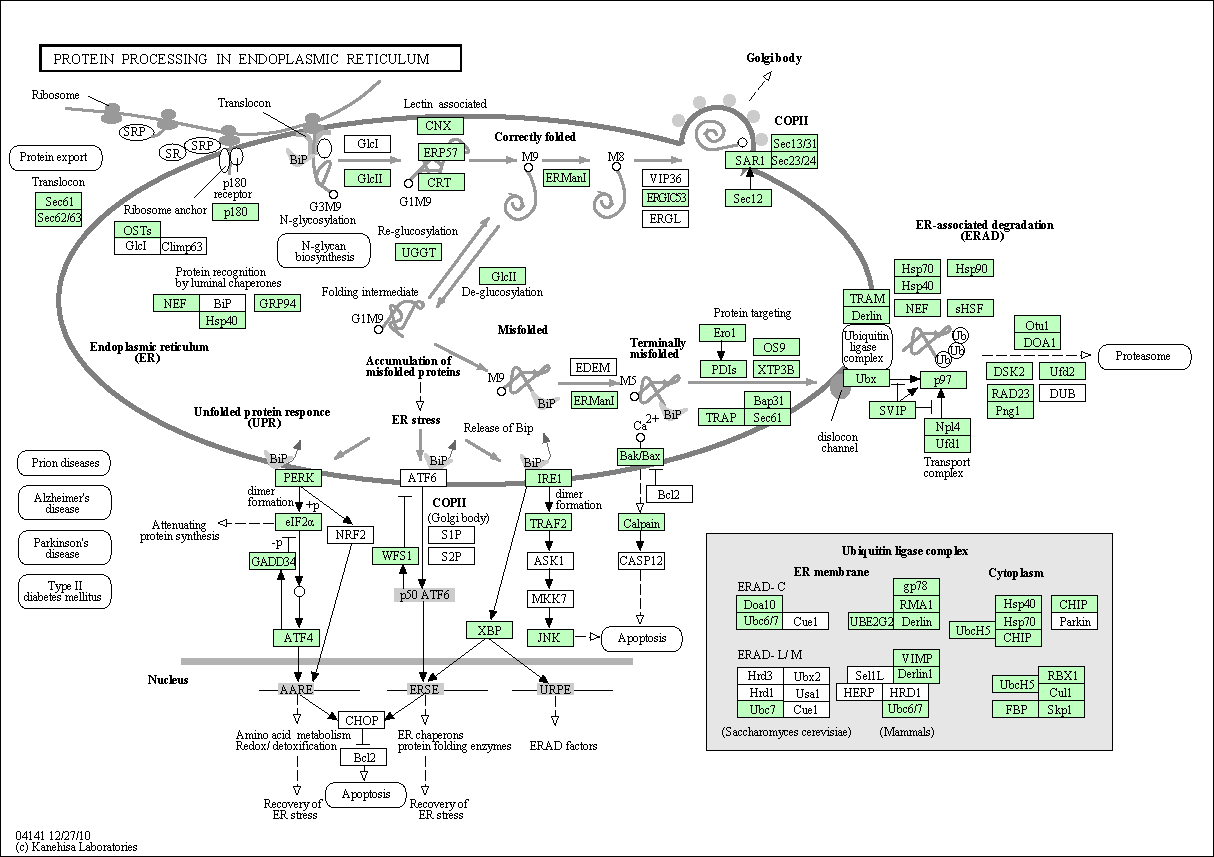

Supplement: Additional file 6 — KEGG annotation results. File contains the KEGG maps where isotigs were successfully mapped. Pathway element in green boxes indicates the components represented by at least, one isotig. Gilthead sea bream isotigs were automatically annotated to KEGG maps using KAAS website [60]. The SBH method, optimized for ESTs annotation, was used against human, chimpanzee, orang-utan, rhesus, mouse, rat, dog, giant panda, cow, pig, horse, opossum, platypus, chicken, clawed frog, zebrafish, fruit fly and nematode pathway databases. [file 1471-2164-13-181-S6.tar › map/map04141.png]

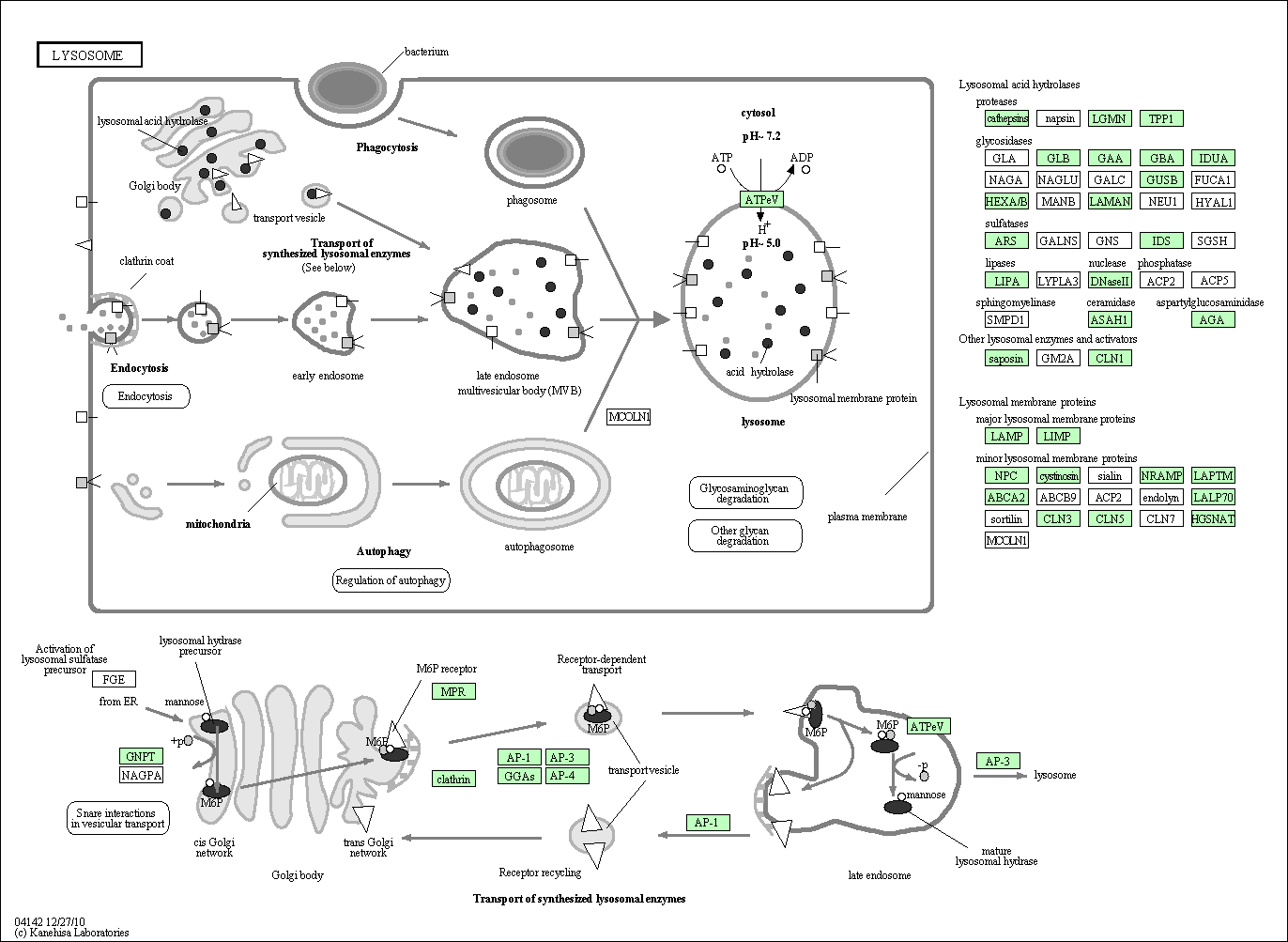

Supplement: Additional file 6 — KEGG annotation results. File contains the KEGG maps where isotigs were successfully mapped. Pathway element in green boxes indicates the components represented by at least, one isotig. Gilthead sea bream isotigs were automatically annotated to KEGG maps using KAAS website [60]. The SBH method, optimized for ESTs annotation, was used against human, chimpanzee, orang-utan, rhesus, mouse, rat, dog, giant panda, cow, pig, horse, opossum, platypus, chicken, clawed frog, zebrafish, fruit fly and nematode pathway databases. [file 1471-2164-13-181-S6.tar › map/map04142.png]

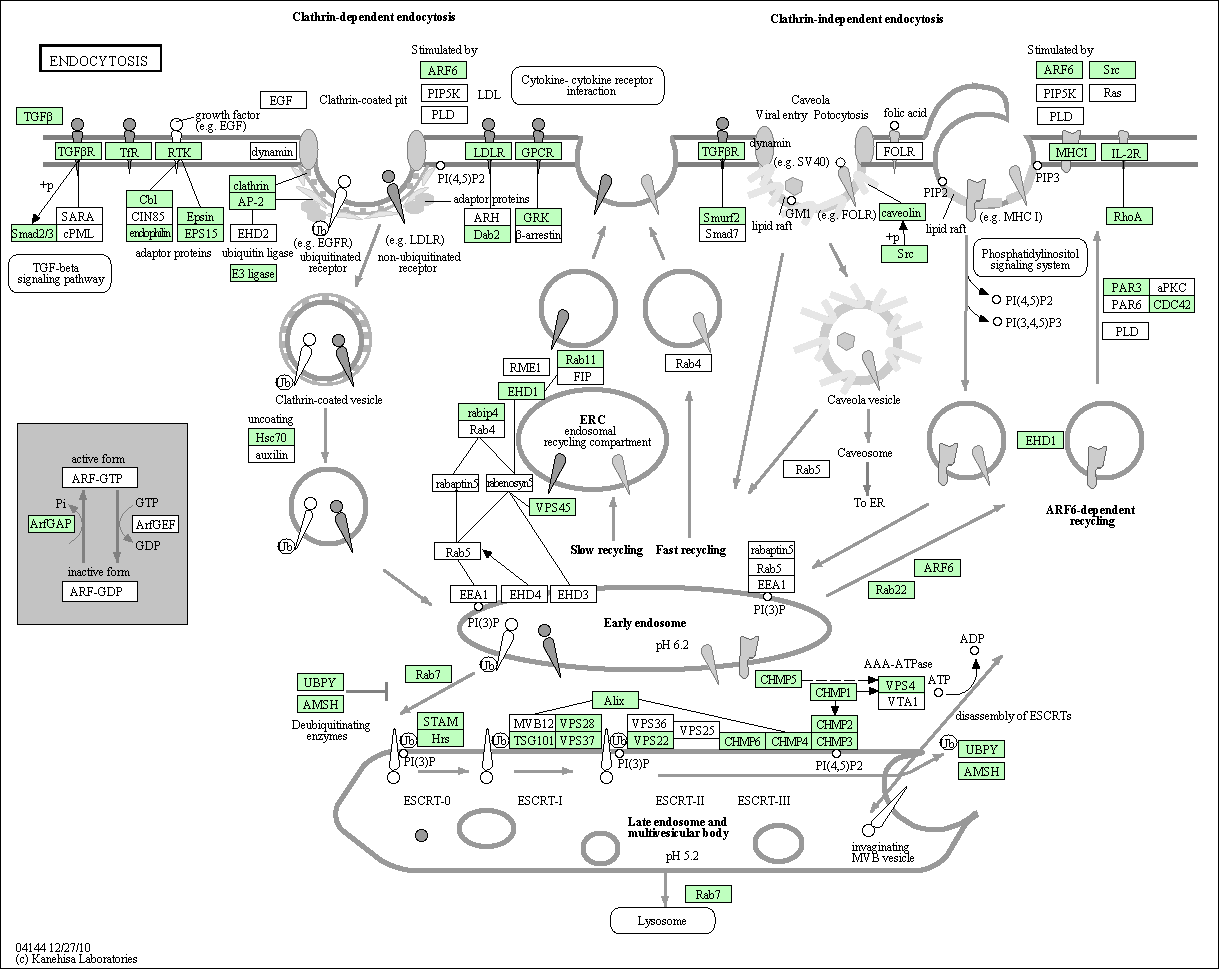

Supplement: Additional file 6 — KEGG annotation results. File contains the KEGG maps where isotigs were successfully mapped. Pathway element in green boxes indicates the components represented by at least, one isotig. Gilthead sea bream isotigs were automatically annotated to KEGG maps using KAAS website [60]. The SBH method, optimized for ESTs annotation, was used against human, chimpanzee, orang-utan, rhesus, mouse, rat, dog, giant panda, cow, pig, horse, opossum, platypus, chicken, clawed frog, zebrafish, fruit fly and nematode pathway databases. [file 1471-2164-13-181-S6.tar › map/map04144.png]

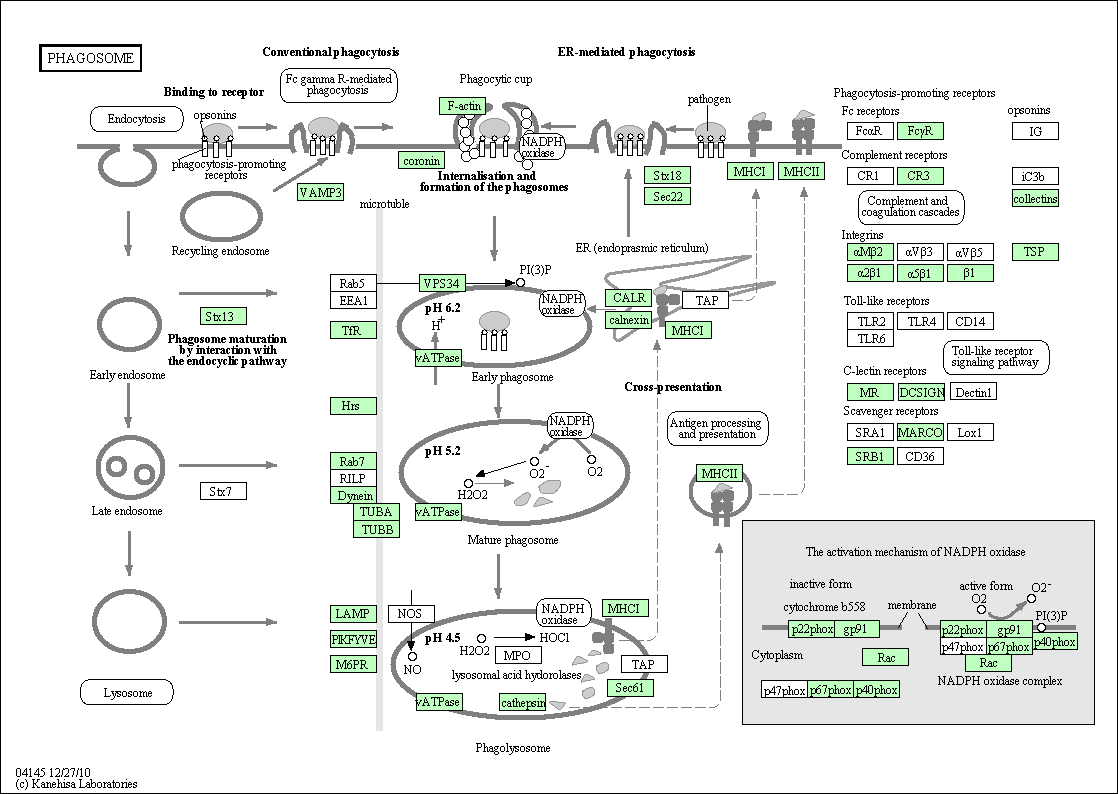

Supplement: Additional file 6 — KEGG annotation results. File contains the KEGG maps where isotigs were successfully mapped. Pathway element in green boxes indicates the components represented by at least, one isotig. Gilthead sea bream isotigs were automatically annotated to KEGG maps using KAAS website [60]. The SBH method, optimized for ESTs annotation, was used against human, chimpanzee, orang-utan, rhesus, mouse, rat, dog, giant panda, cow, pig, horse, opossum, platypus, chicken, clawed frog, zebrafish, fruit fly and nematode pathway databases. [file 1471-2164-13-181-S6.tar › map/map04145.png]

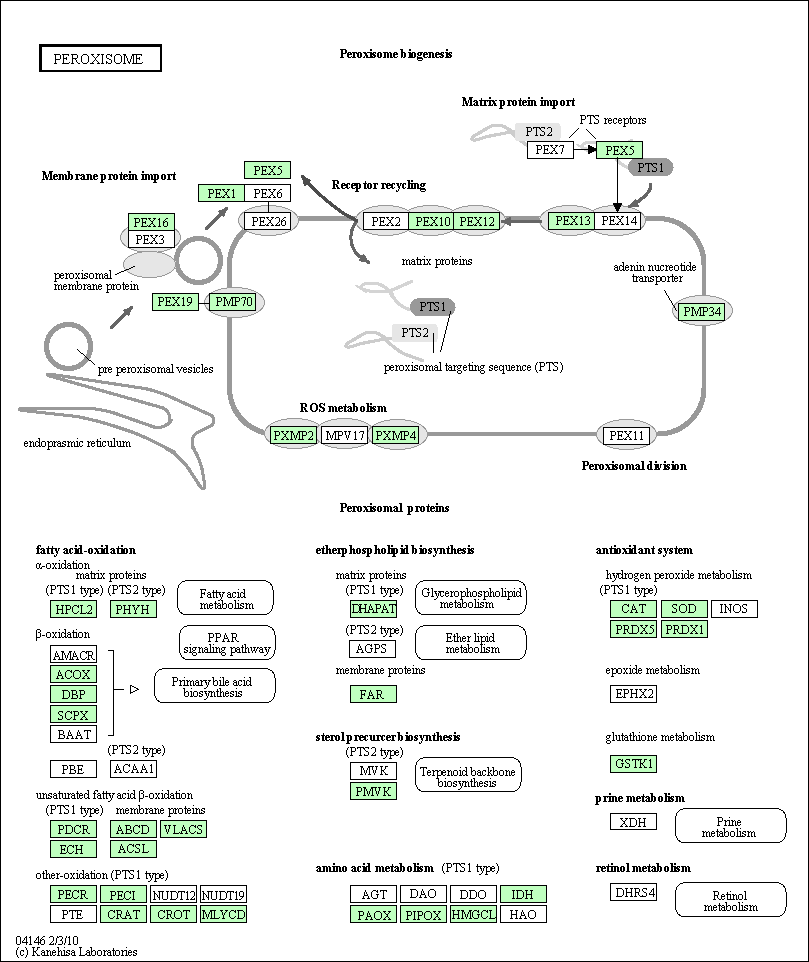

Supplement: Additional file 6 — KEGG annotation results. File contains the KEGG maps where isotigs were successfully mapped. Pathway element in green boxes indicates the components represented by at least, one isotig. Gilthead sea bream isotigs were automatically annotated to KEGG maps using KAAS website [60]. The SBH method, optimized for ESTs annotation, was used against human, chimpanzee, orang-utan, rhesus, mouse, rat, dog, giant panda, cow, pig, horse, opossum, platypus, chicken, clawed frog, zebrafish, fruit fly and nematode pathway databases. [file 1471-2164-13-181-S6.tar › map/map04146.png]

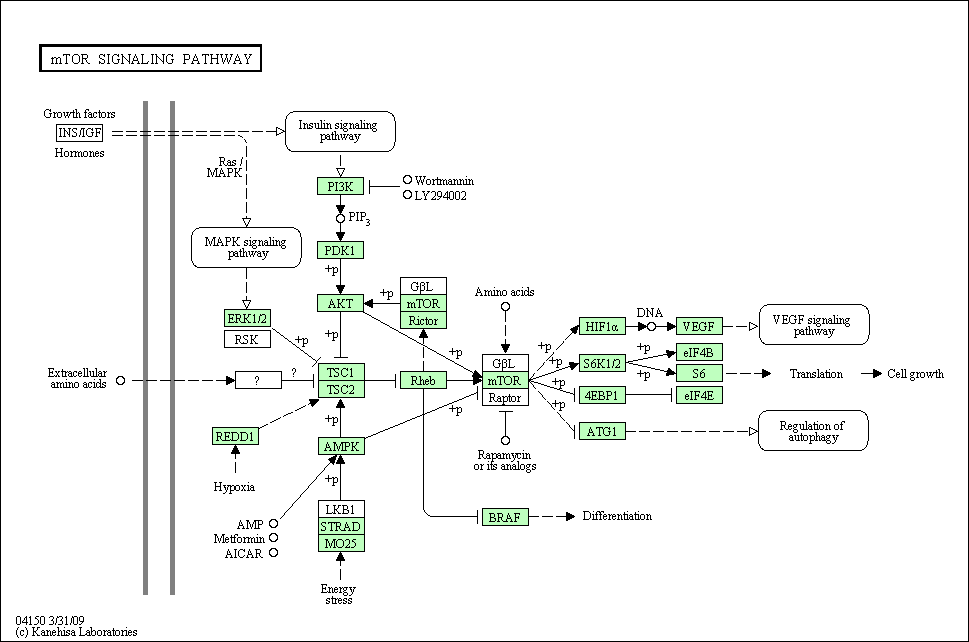

Supplement: Additional file 6 — KEGG annotation results. File contains the KEGG maps where isotigs were successfully mapped. Pathway element in green boxes indicates the components represented by at least, one isotig. Gilthead sea bream isotigs were automatically annotated to KEGG maps using KAAS website [60]. The SBH method, optimized for ESTs annotation, was used against human, chimpanzee, orang-utan, rhesus, mouse, rat, dog, giant panda, cow, pig, horse, opossum, platypus, chicken, clawed frog, zebrafish, fruit fly and nematode pathway databases. [file 1471-2164-13-181-S6.tar › map/map04150.png]

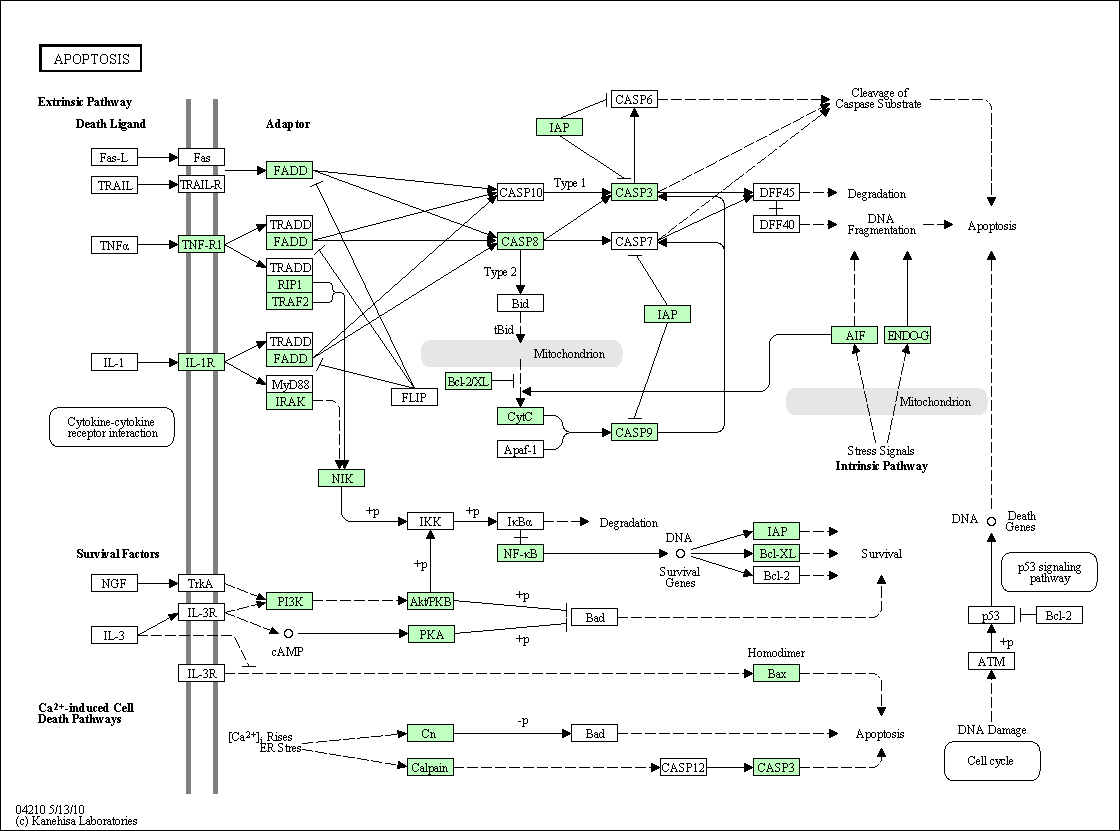

Supplement: Additional file 6 — KEGG annotation results. File contains the KEGG maps where isotigs were successfully mapped. Pathway element in green boxes indicates the components represented by at least, one isotig. Gilthead sea bream isotigs were automatically annotated to KEGG maps using KAAS website [60]. The SBH method, optimized for ESTs annotation, was used against human, chimpanzee, orang-utan, rhesus, mouse, rat, dog, giant panda, cow, pig, horse, opossum, platypus, chicken, clawed frog, zebrafish, fruit fly and nematode pathway databases. [file 1471-2164-13-181-S6.tar › map/map04210.png]

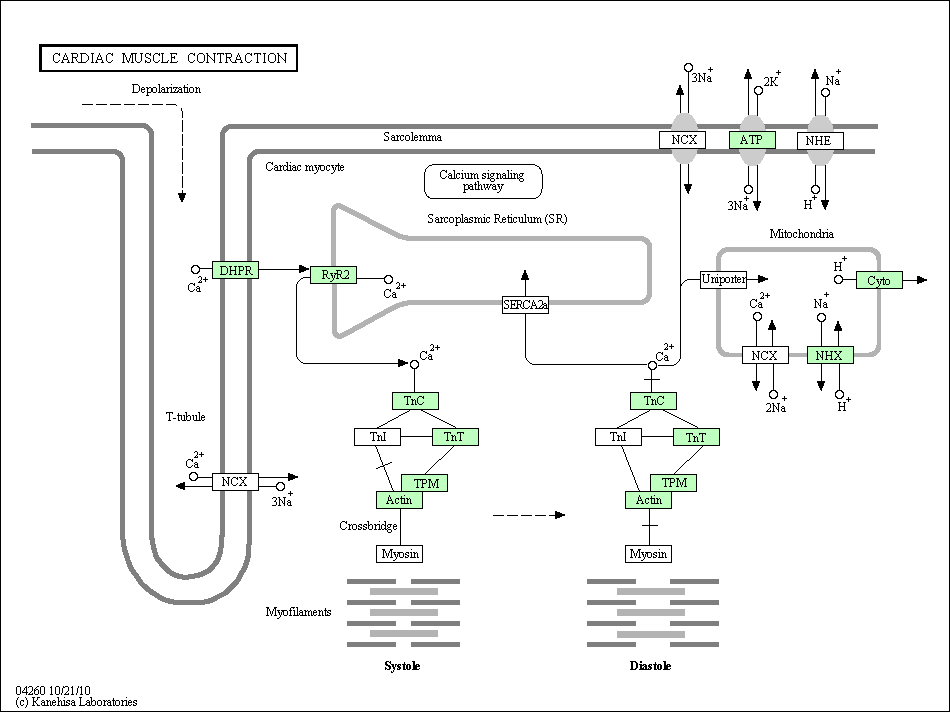

Supplement: Additional file 6 — KEGG annotation results. File contains the KEGG maps where isotigs were successfully mapped. Pathway element in green boxes indicates the components represented by at least, one isotig. Gilthead sea bream isotigs were automatically annotated to KEGG maps using KAAS website [60]. The SBH method, optimized for ESTs annotation, was used against human, chimpanzee, orang-utan, rhesus, mouse, rat, dog, giant panda, cow, pig, horse, opossum, platypus, chicken, clawed frog, zebrafish, fruit fly and nematode pathway databases. [file 1471-2164-13-181-S6.tar › map/map04260.png]

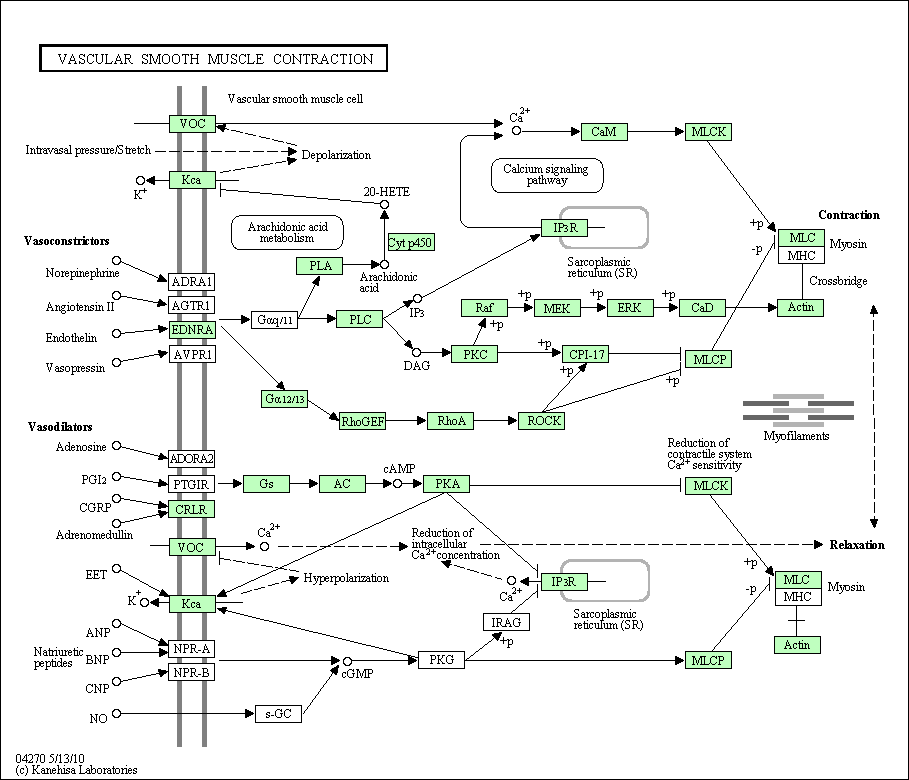

Supplement: Additional file 6 — KEGG annotation results. File contains the KEGG maps where isotigs were successfully mapped. Pathway element in green boxes indicates the components represented by at least, one isotig. Gilthead sea bream isotigs were automatically annotated to KEGG maps using KAAS website [60]. The SBH method, optimized for ESTs annotation, was used against human, chimpanzee, orang-utan, rhesus, mouse, rat, dog, giant panda, cow, pig, horse, opossum, platypus, chicken, clawed frog, zebrafish, fruit fly and nematode pathway databases. [file 1471-2164-13-181-S6.tar › map/map04270.png]

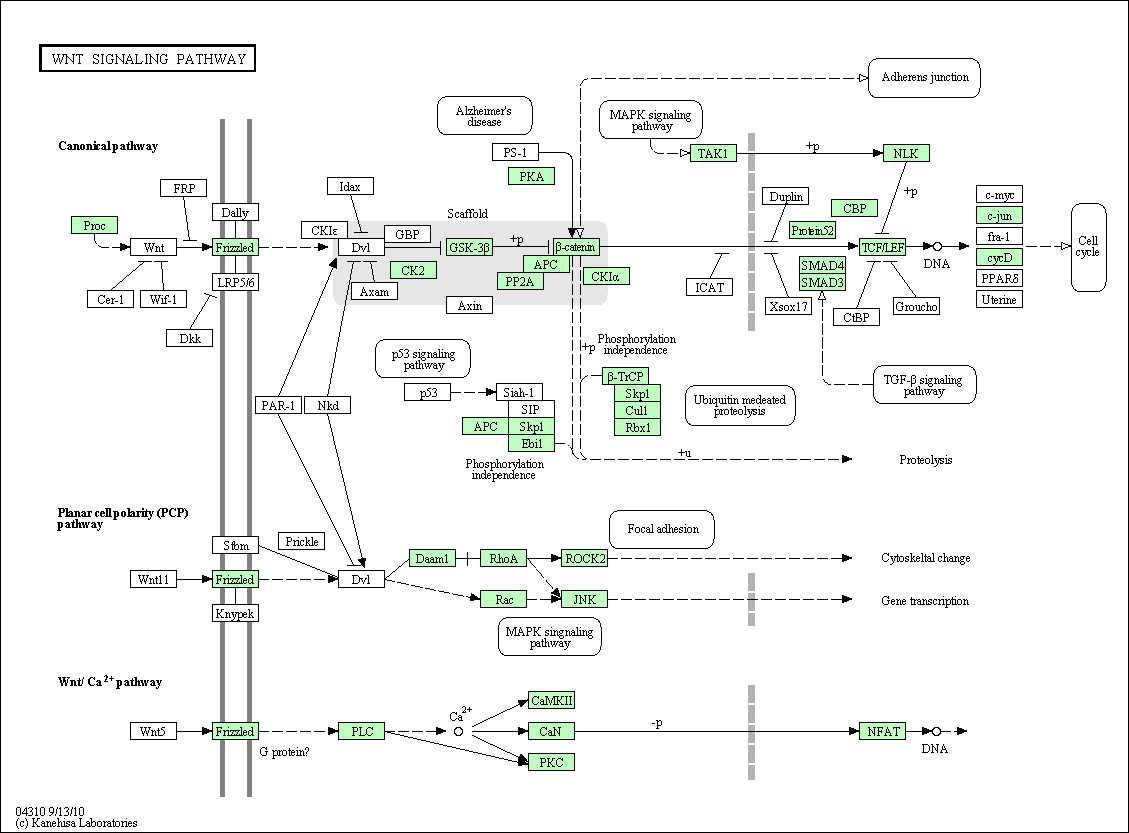

Supplement: Additional file 6 — KEGG annotation results. File contains the KEGG maps where isotigs were successfully mapped. Pathway element in green boxes indicates the components represented by at least, one isotig. Gilthead sea bream isotigs were automatically annotated to KEGG maps using KAAS website [60]. The SBH method, optimized for ESTs annotation, was used against human, chimpanzee, orang-utan, rhesus, mouse, rat, dog, giant panda, cow, pig, horse, opossum, platypus, chicken, clawed frog, zebrafish, fruit fly and nematode pathway databases. [file 1471-2164-13-181-S6.tar › map/map04310.png]

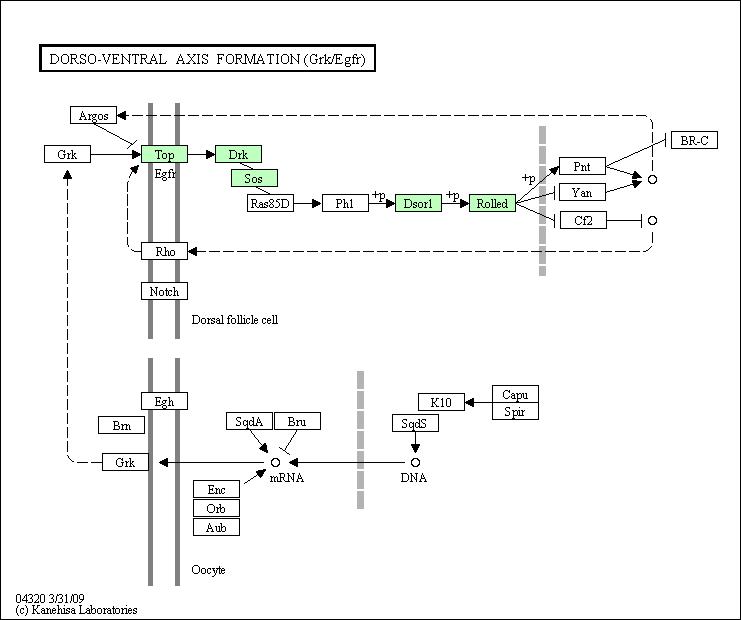

Supplement: Additional file 6 — KEGG annotation results. File contains the KEGG maps where isotigs were successfully mapped. Pathway element in green boxes indicates the components represented by at least, one isotig. Gilthead sea bream isotigs were automatically annotated to KEGG maps using KAAS website [60]. The SBH method, optimized for ESTs annotation, was used against human, chimpanzee, orang-utan, rhesus, mouse, rat, dog, giant panda, cow, pig, horse, opossum, platypus, chicken, clawed frog, zebrafish, fruit fly and nematode pathway databases. [file 1471-2164-13-181-S6.tar › map/map04320.png]

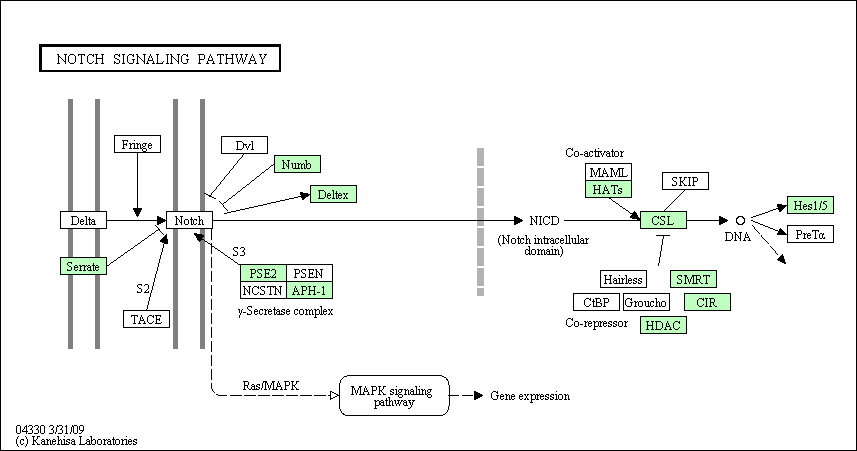

Supplement: Additional file 6 — KEGG annotation results. File contains the KEGG maps where isotigs were successfully mapped. Pathway element in green boxes indicates the components represented by at least, one isotig. Gilthead sea bream isotigs were automatically annotated to KEGG maps using KAAS website [60]. The SBH method, optimized for ESTs annotation, was used against human, chimpanzee, orang-utan, rhesus, mouse, rat, dog, giant panda, cow, pig, horse, opossum, platypus, chicken, clawed frog, zebrafish, fruit fly and nematode pathway databases. [file 1471-2164-13-181-S6.tar › map/map04330.png]

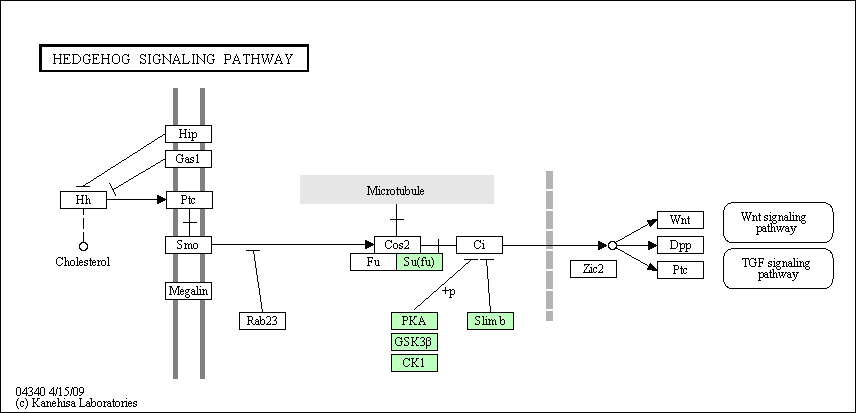

Supplement: Additional file 6 — KEGG annotation results. File contains the KEGG maps where isotigs were successfully mapped. Pathway element in green boxes indicates the components represented by at least, one isotig. Gilthead sea bream isotigs were automatically annotated to KEGG maps using KAAS website [60]. The SBH method, optimized for ESTs annotation, was used against human, chimpanzee, orang-utan, rhesus, mouse, rat, dog, giant panda, cow, pig, horse, opossum, platypus, chicken, clawed frog, zebrafish, fruit fly and nematode pathway databases. [file 1471-2164-13-181-S6.tar › map/map04340.png]

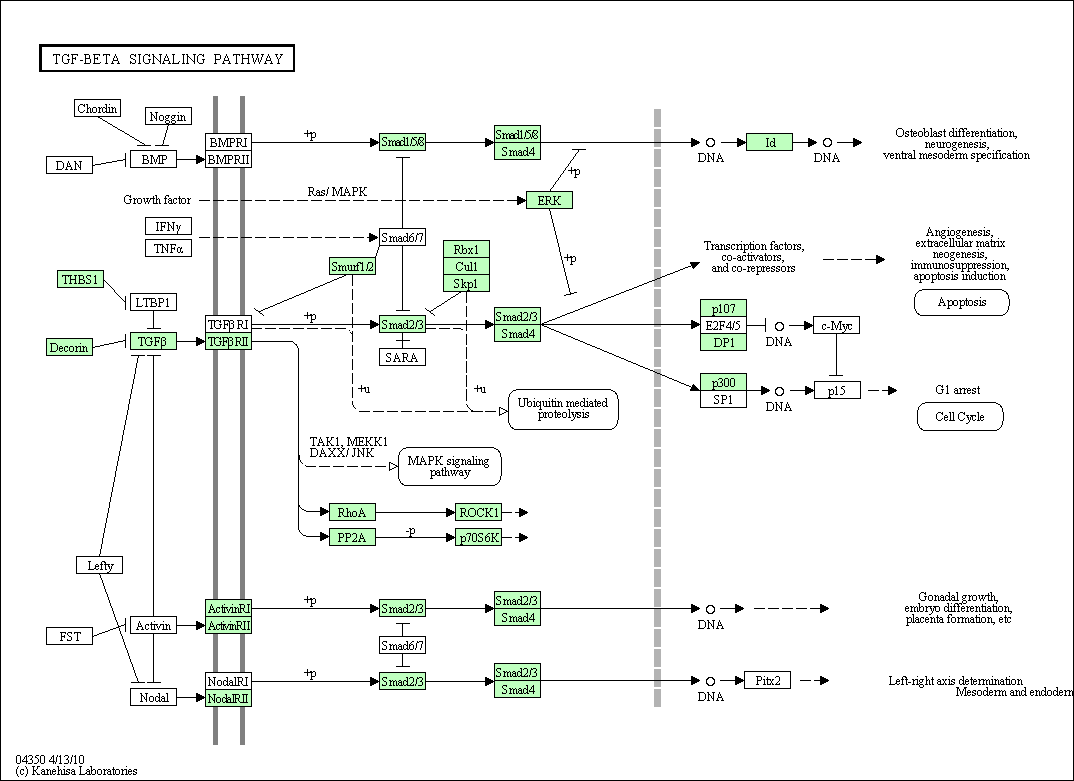

Supplement: Additional file 6 — KEGG annotation results. File contains the KEGG maps where isotigs were successfully mapped. Pathway element in green boxes indicates the components represented by at least, one isotig. Gilthead sea bream isotigs were automatically annotated to KEGG maps using KAAS website [60]. The SBH method, optimized for ESTs annotation, was used against human, chimpanzee, orang-utan, rhesus, mouse, rat, dog, giant panda, cow, pig, horse, opossum, platypus, chicken, clawed frog, zebrafish, fruit fly and nematode pathway databases. [file 1471-2164-13-181-S6.tar › map/map04350.png]

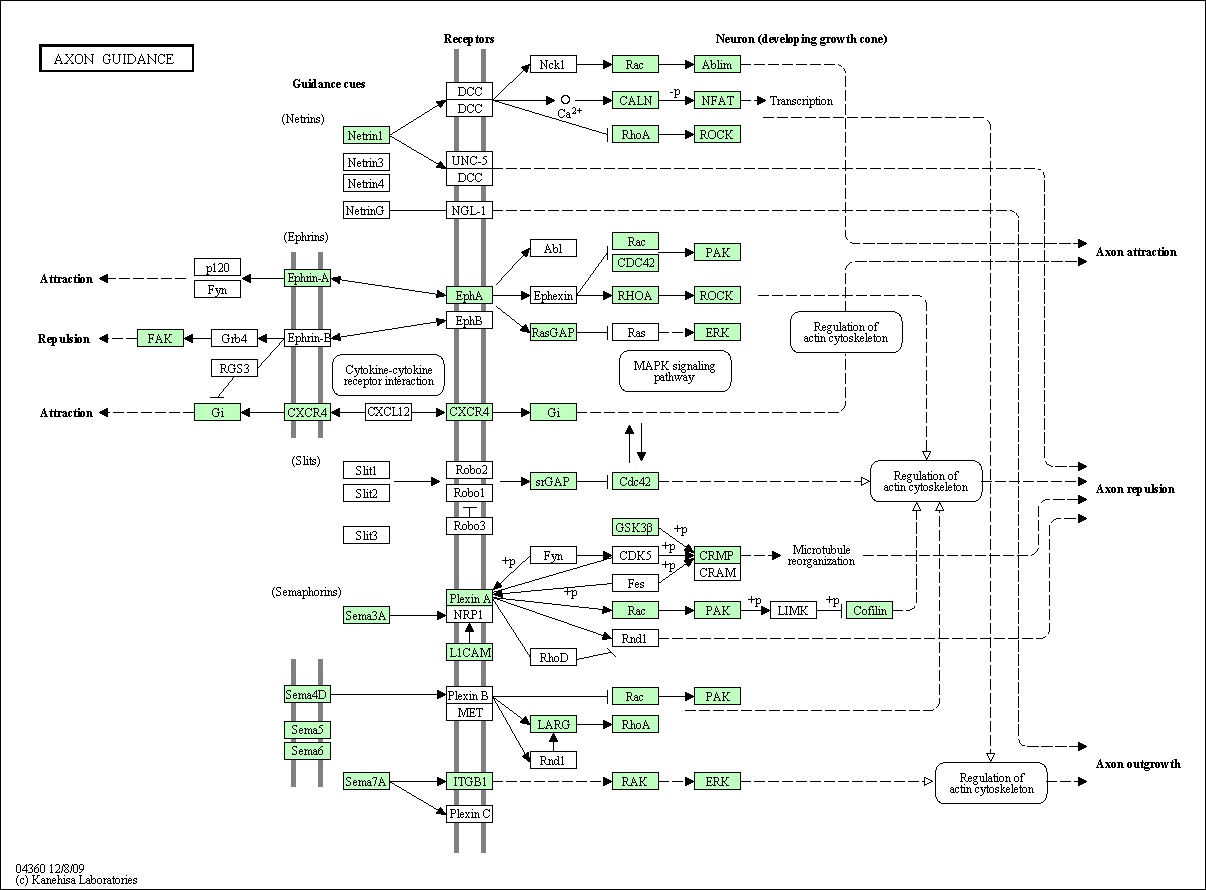

Supplement: Additional file 6 — KEGG annotation results. File contains the KEGG maps where isotigs were successfully mapped. Pathway element in green boxes indicates the components represented by at least, one isotig. Gilthead sea bream isotigs were automatically annotated to KEGG maps using KAAS website [60]. The SBH method, optimized for ESTs annotation, was used against human, chimpanzee, orang-utan, rhesus, mouse, rat, dog, giant panda, cow, pig, horse, opossum, platypus, chicken, clawed frog, zebrafish, fruit fly and nematode pathway databases. [file 1471-2164-13-181-S6.tar › map/map04360.png]

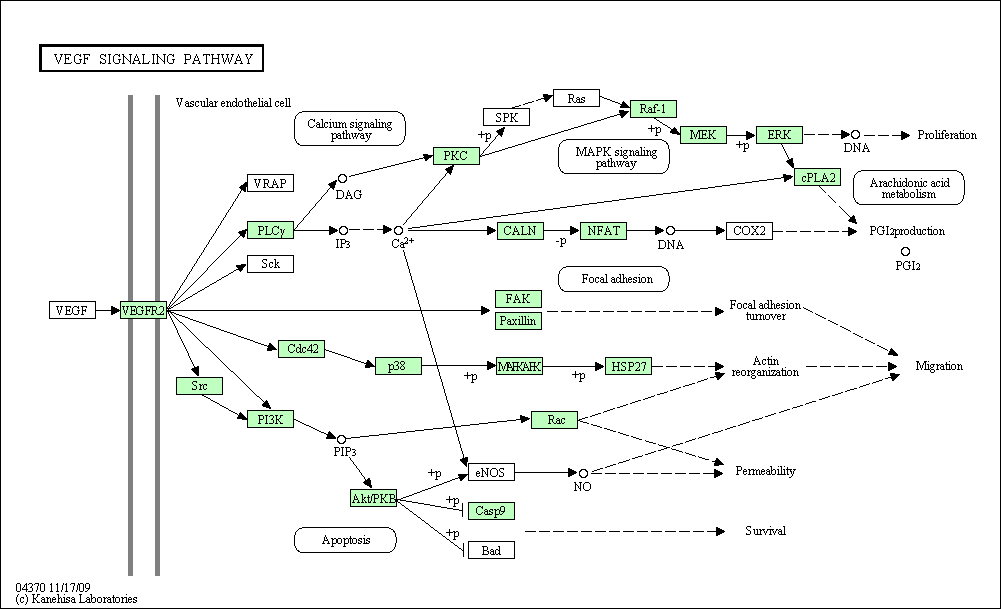

Supplement: Additional file 6 — KEGG annotation results. File contains the KEGG maps where isotigs were successfully mapped. Pathway element in green boxes indicates the components represented by at least, one isotig. Gilthead sea bream isotigs were automatically annotated to KEGG maps using KAAS website [60]. The SBH method, optimized for ESTs annotation, was used against human, chimpanzee, orang-utan, rhesus, mouse, rat, dog, giant panda, cow, pig, horse, opossum, platypus, chicken, clawed frog, zebrafish, fruit fly and nematode pathway databases. [file 1471-2164-13-181-S6.tar › map/map04370.png]

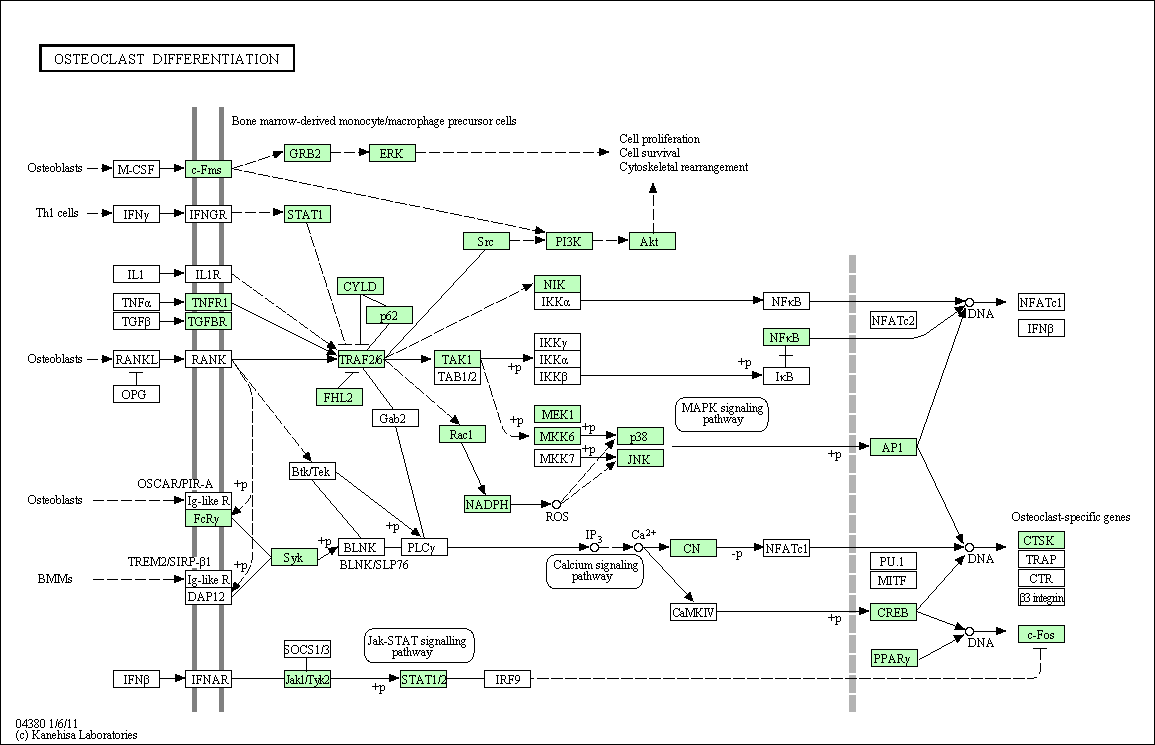

Supplement: Additional file 6 — KEGG annotation results. File contains the KEGG maps where isotigs were successfully mapped. Pathway element in green boxes indicates the components represented by at least, one isotig. Gilthead sea bream isotigs were automatically annotated to KEGG maps using KAAS website [60]. The SBH method, optimized for ESTs annotation, was used against human, chimpanzee, orang-utan, rhesus, mouse, rat, dog, giant panda, cow, pig, horse, opossum, platypus, chicken, clawed frog, zebrafish, fruit fly and nematode pathway databases. [file 1471-2164-13-181-S6.tar › map/map04380.png]

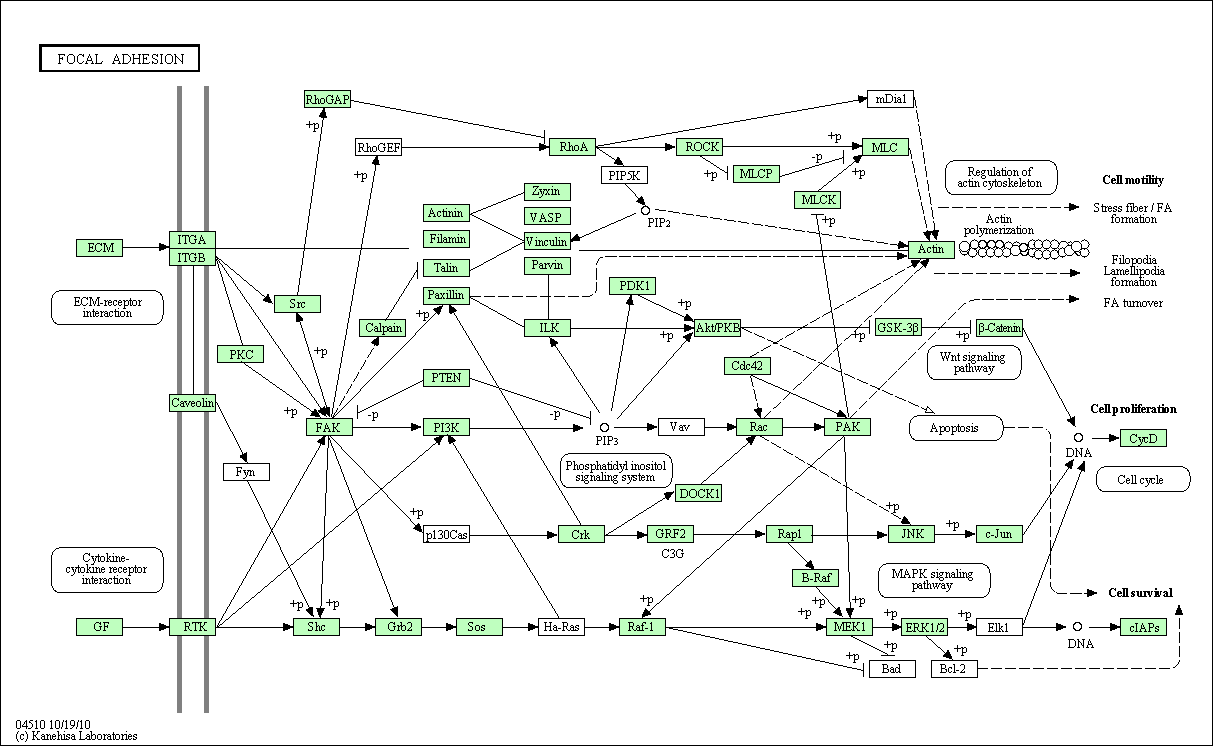

Supplement: Additional file 6 — KEGG annotation results. File contains the KEGG maps where isotigs were successfully mapped. Pathway element in green boxes indicates the components represented by at least, one isotig. Gilthead sea bream isotigs were automatically annotated to KEGG maps using KAAS website [60]. The SBH method, optimized for ESTs annotation, was used against human, chimpanzee, orang-utan, rhesus, mouse, rat, dog, giant panda, cow, pig, horse, opossum, platypus, chicken, clawed frog, zebrafish, fruit fly and nematode pathway databases. [file 1471-2164-13-181-S6.tar › map/map04510.png]

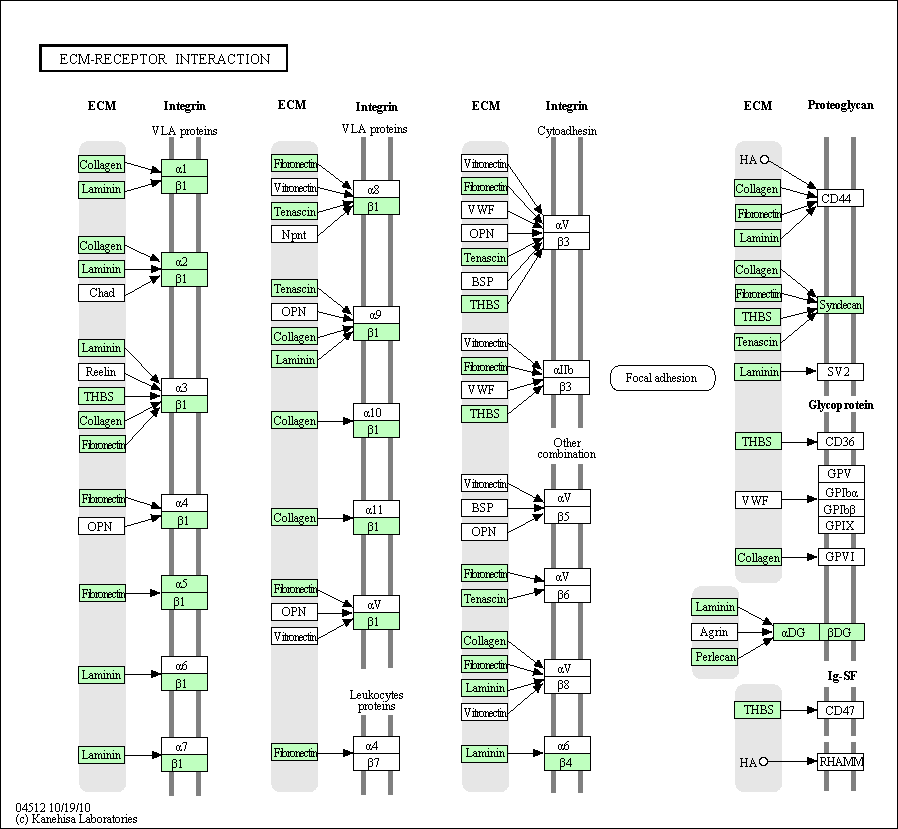

Supplement: Additional file 6 — KEGG annotation results. File contains the KEGG maps where isotigs were successfully mapped. Pathway element in green boxes indicates the components represented by at least, one isotig. Gilthead sea bream isotigs were automatically annotated to KEGG maps using KAAS website [60]. The SBH method, optimized for ESTs annotation, was used against human, chimpanzee, orang-utan, rhesus, mouse, rat, dog, giant panda, cow, pig, horse, opossum, platypus, chicken, clawed frog, zebrafish, fruit fly and nematode pathway databases. [file 1471-2164-13-181-S6.tar › map/map04512.png]

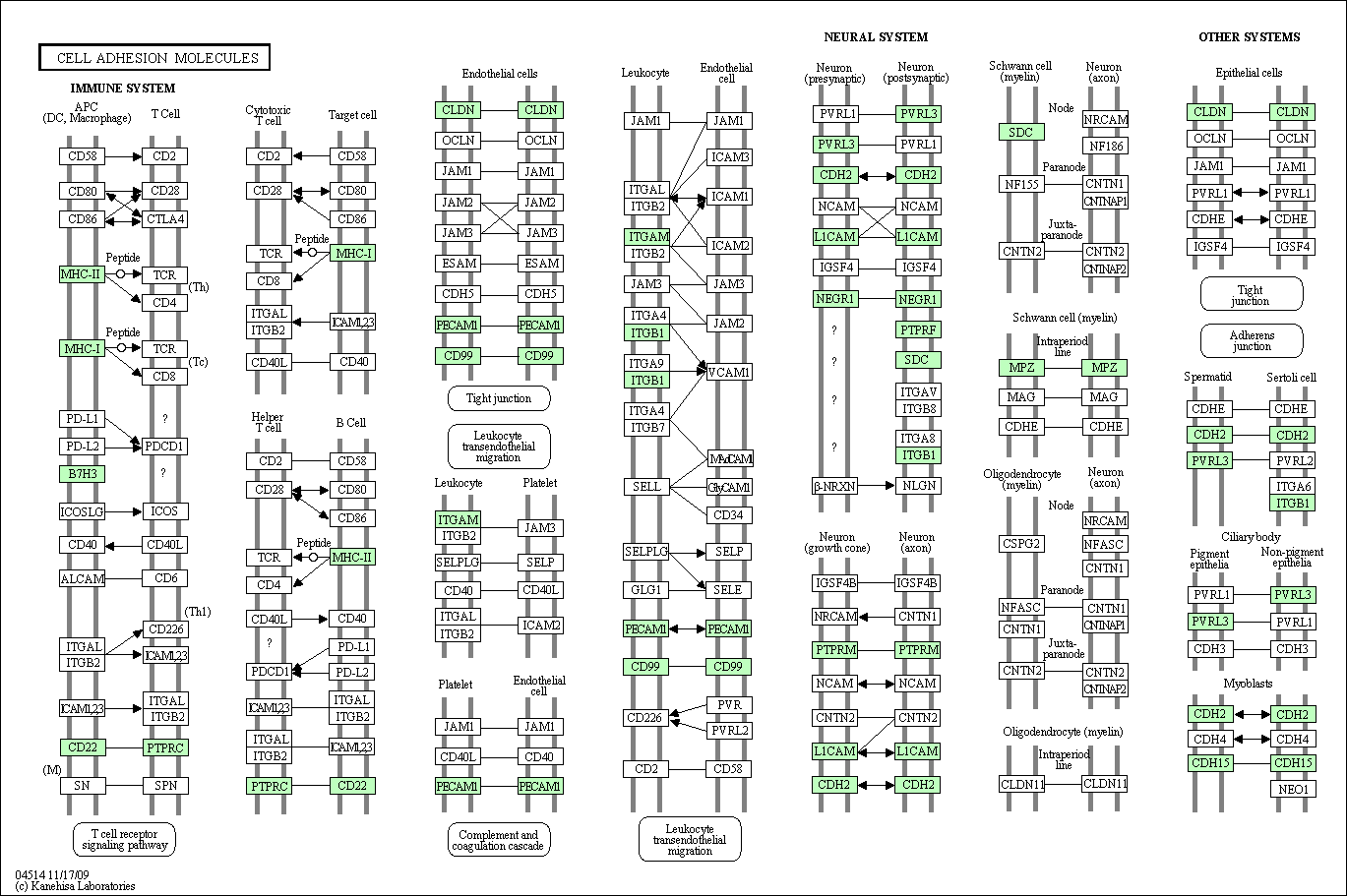

Supplement: Additional file 6 — KEGG annotation results. File contains the KEGG maps where isotigs were successfully mapped. Pathway element in green boxes indicates the components represented by at least, one isotig. Gilthead sea bream isotigs were automatically annotated to KEGG maps using KAAS website [60]. The SBH method, optimized for ESTs annotation, was used against human, chimpanzee, orang-utan, rhesus, mouse, rat, dog, giant panda, cow, pig, horse, opossum, platypus, chicken, clawed frog, zebrafish, fruit fly and nematode pathway databases. [file 1471-2164-13-181-S6.tar › map/map04514.png]

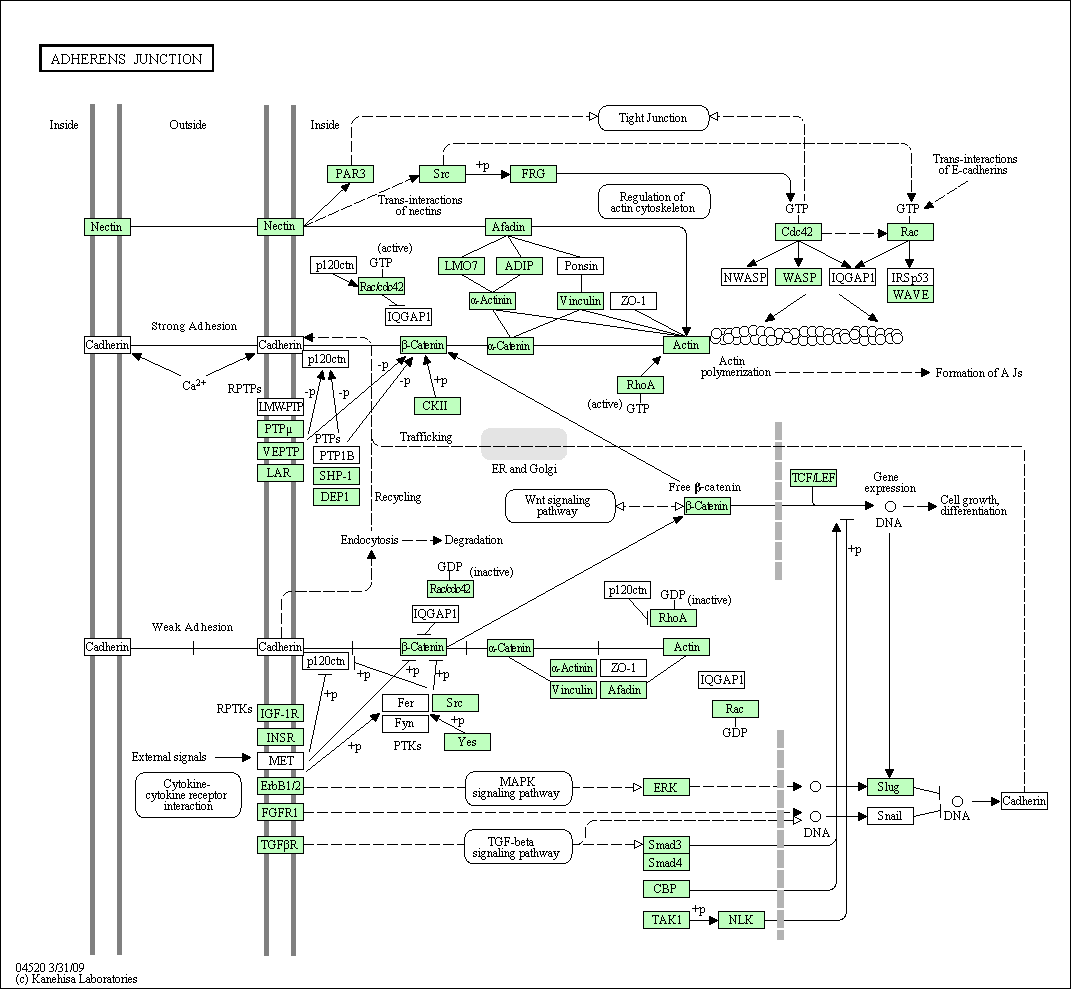

Supplement: Additional file 6 — KEGG annotation results. File contains the KEGG maps where isotigs were successfully mapped. Pathway element in green boxes indicates the components represented by at least, one isotig. Gilthead sea bream isotigs were automatically annotated to KEGG maps using KAAS website [60]. The SBH method, optimized for ESTs annotation, was used against human, chimpanzee, orang-utan, rhesus, mouse, rat, dog, giant panda, cow, pig, horse, opossum, platypus, chicken, clawed frog, zebrafish, fruit fly and nematode pathway databases. [file 1471-2164-13-181-S6.tar › map/map04520.png]

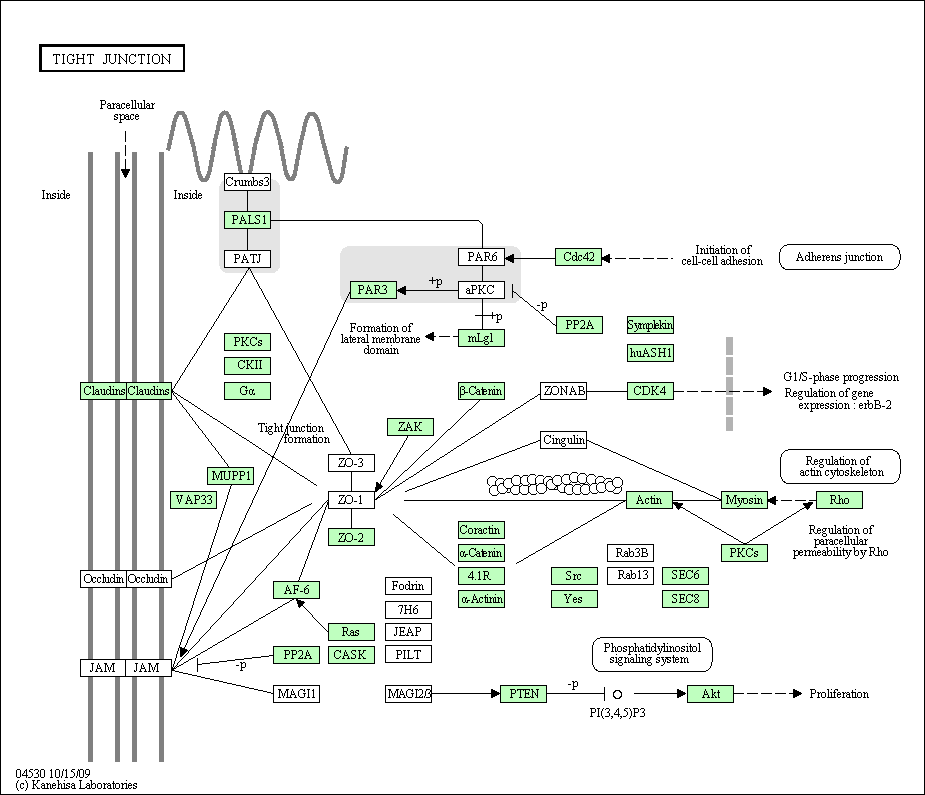

Supplement: Additional file 6 — KEGG annotation results. File contains the KEGG maps where isotigs were successfully mapped. Pathway element in green boxes indicates the components represented by at least, one isotig. Gilthead sea bream isotigs were automatically annotated to KEGG maps using KAAS website [60]. The SBH method, optimized for ESTs annotation, was used against human, chimpanzee, orang-utan, rhesus, mouse, rat, dog, giant panda, cow, pig, horse, opossum, platypus, chicken, clawed frog, zebrafish, fruit fly and nematode pathway databases. [file 1471-2164-13-181-S6.tar › map/map04530.png]

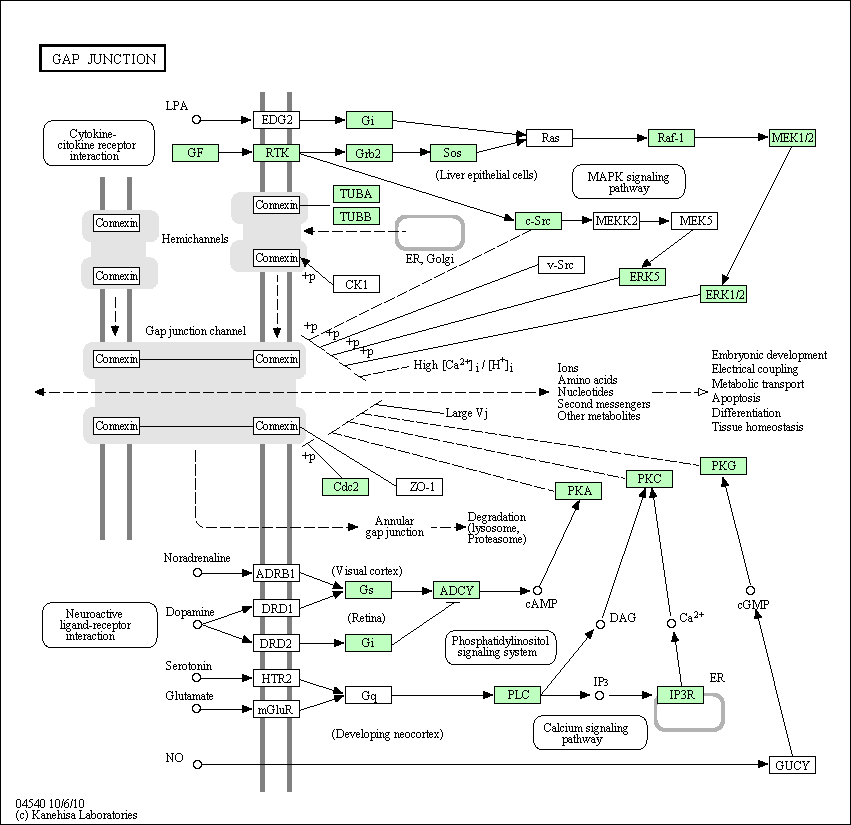

Supplement: Additional file 6 — KEGG annotation results. File contains the KEGG maps where isotigs were successfully mapped. Pathway element in green boxes indicates the components represented by at least, one isotig. Gilthead sea bream isotigs were automatically annotated to KEGG maps using KAAS website [60]. The SBH method, optimized for ESTs annotation, was used against human, chimpanzee, orang-utan, rhesus, mouse, rat, dog, giant panda, cow, pig, horse, opossum, platypus, chicken, clawed frog, zebrafish, fruit fly and nematode pathway databases. [file 1471-2164-13-181-S6.tar › map/map04540.png]

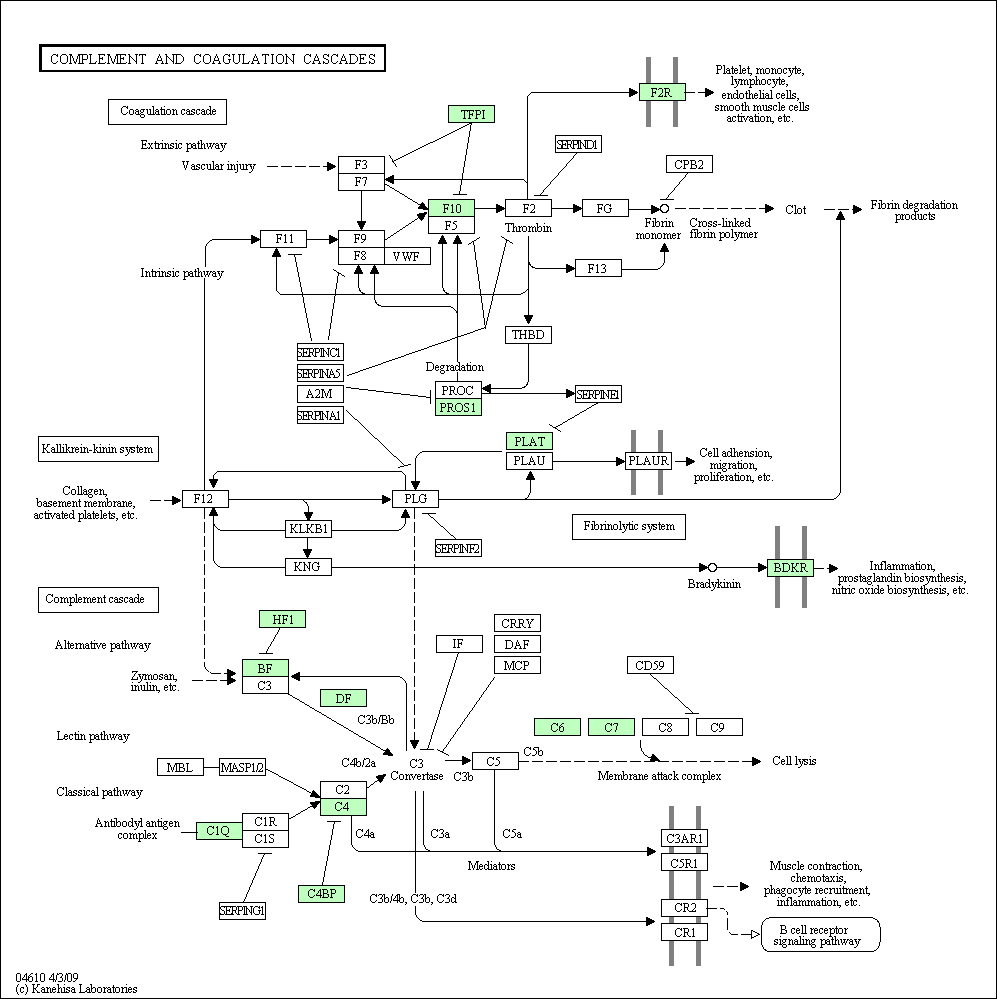

Supplement: Additional file 6 — KEGG annotation results. File contains the KEGG maps where isotigs were successfully mapped. Pathway element in green boxes indicates the components represented by at least, one isotig. Gilthead sea bream isotigs were automatically annotated to KEGG maps using KAAS website [60]. The SBH method, optimized for ESTs annotation, was used against human, chimpanzee, orang-utan, rhesus, mouse, rat, dog, giant panda, cow, pig, horse, opossum, platypus, chicken, clawed frog, zebrafish, fruit fly and nematode pathway databases. [file 1471-2164-13-181-S6.tar › map/map04610.png]

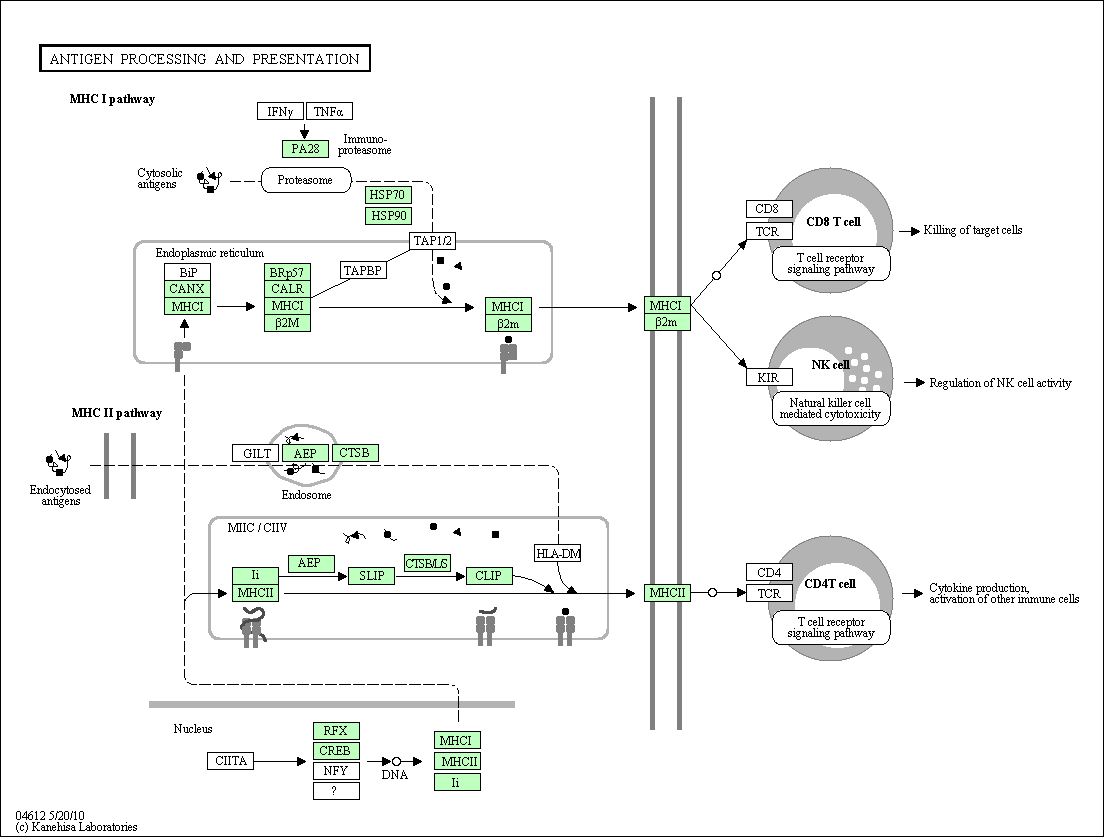

Supplement: Additional file 6 — KEGG annotation results. File contains the KEGG maps where isotigs were successfully mapped. Pathway element in green boxes indicates the components represented by at least, one isotig. Gilthead sea bream isotigs were automatically annotated to KEGG maps using KAAS website [60]. The SBH method, optimized for ESTs annotation, was used against human, chimpanzee, orang-utan, rhesus, mouse, rat, dog, giant panda, cow, pig, horse, opossum, platypus, chicken, clawed frog, zebrafish, fruit fly and nematode pathway databases. [file 1471-2164-13-181-S6.tar › map/map04612.png]

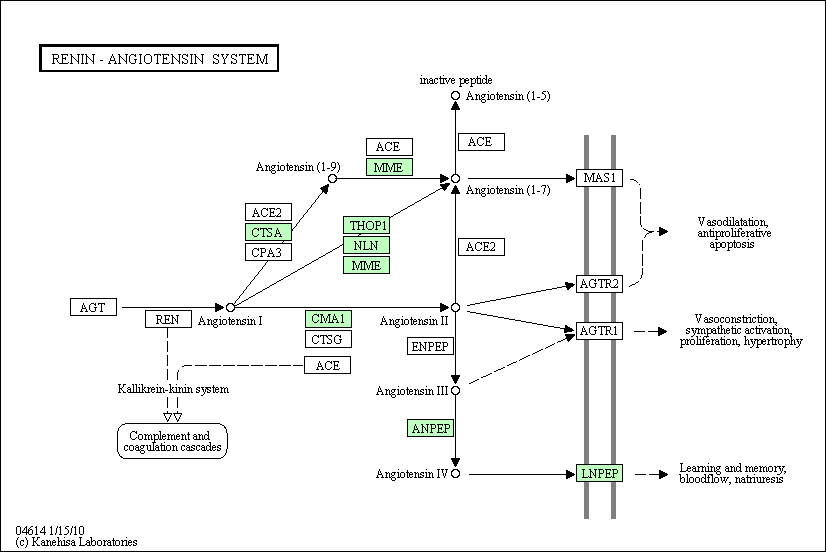

Supplement: Additional file 6 — KEGG annotation results. File contains the KEGG maps where isotigs were successfully mapped. Pathway element in green boxes indicates the components represented by at least, one isotig. Gilthead sea bream isotigs were automatically annotated to KEGG maps using KAAS website [60]. The SBH method, optimized for ESTs annotation, was used against human, chimpanzee, orang-utan, rhesus, mouse, rat, dog, giant panda, cow, pig, horse, opossum, platypus, chicken, clawed frog, zebrafish, fruit fly and nematode pathway databases. [file 1471-2164-13-181-S6.tar › map/map04614.png]

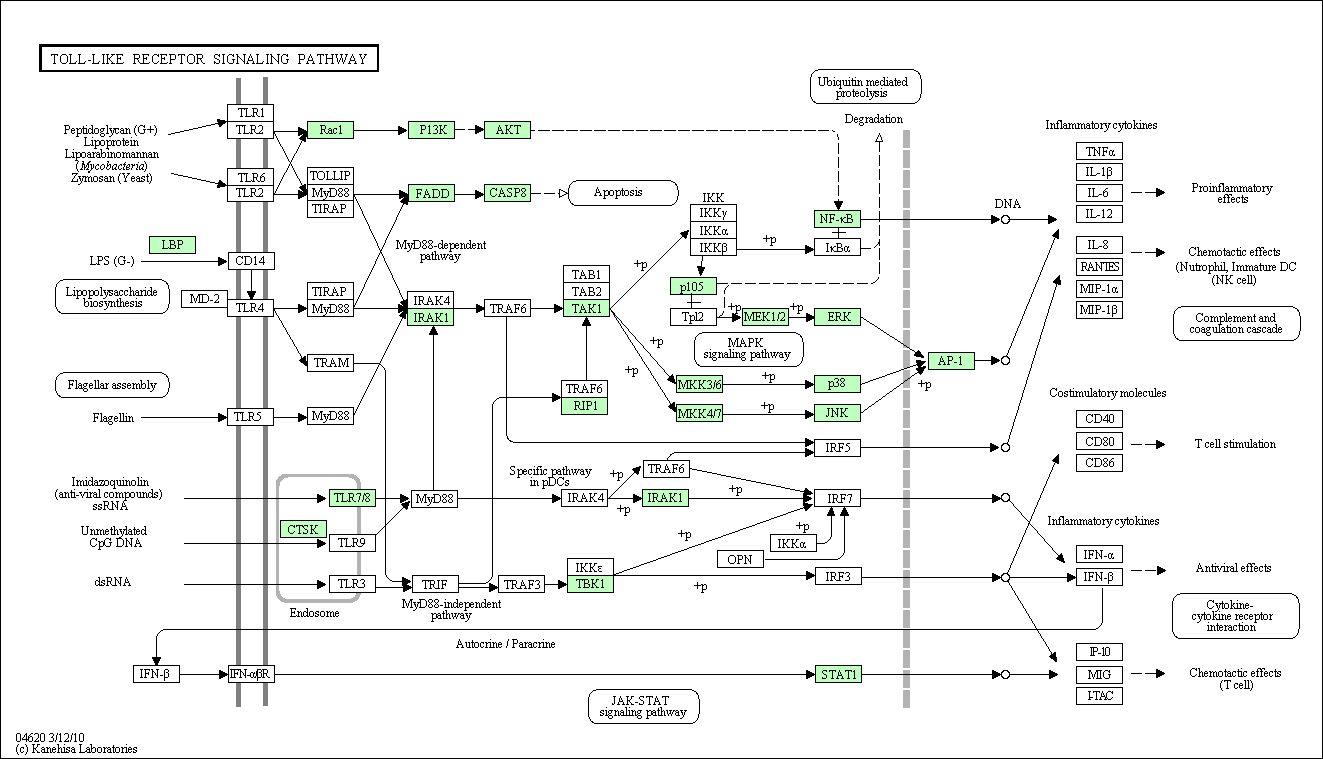

Supplement: Additional file 6 — KEGG annotation results. File contains the KEGG maps where isotigs were successfully mapped. Pathway element in green boxes indicates the components represented by at least, one isotig. Gilthead sea bream isotigs were automatically annotated to KEGG maps using KAAS website [60]. The SBH method, optimized for ESTs annotation, was used against human, chimpanzee, orang-utan, rhesus, mouse, rat, dog, giant panda, cow, pig, horse, opossum, platypus, chicken, clawed frog, zebrafish, fruit fly and nematode pathway databases. [file 1471-2164-13-181-S6.tar › map/map04620.png]

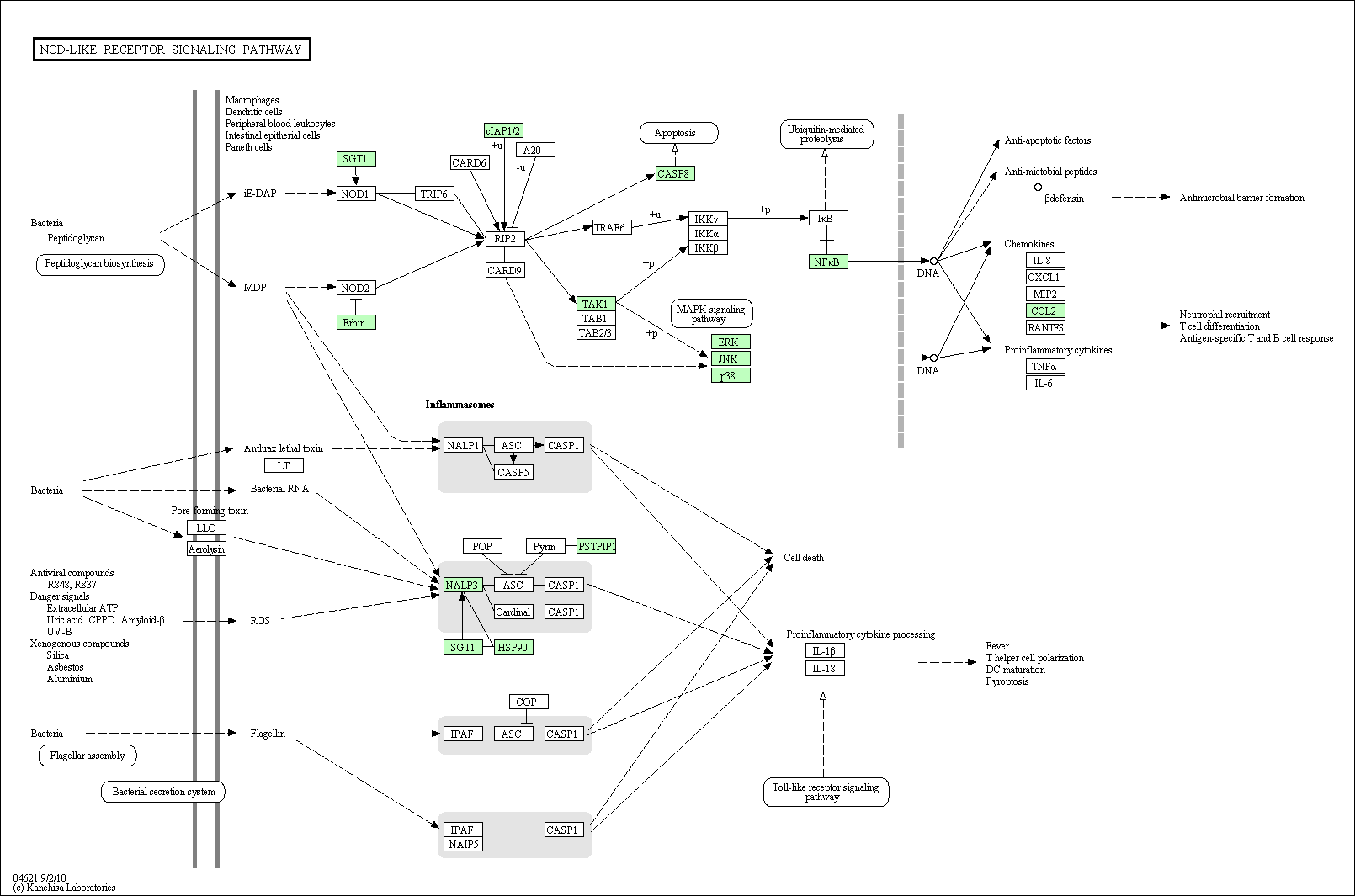

Supplement: Additional file 6 — KEGG annotation results. File contains the KEGG maps where isotigs were successfully mapped. Pathway element in green boxes indicates the components represented by at least, one isotig. Gilthead sea bream isotigs were automatically annotated to KEGG maps using KAAS website [60]. The SBH method, optimized for ESTs annotation, was used against human, chimpanzee, orang-utan, rhesus, mouse, rat, dog, giant panda, cow, pig, horse, opossum, platypus, chicken, clawed frog, zebrafish, fruit fly and nematode pathway databases. [file 1471-2164-13-181-S6.tar › map/map04621.png]

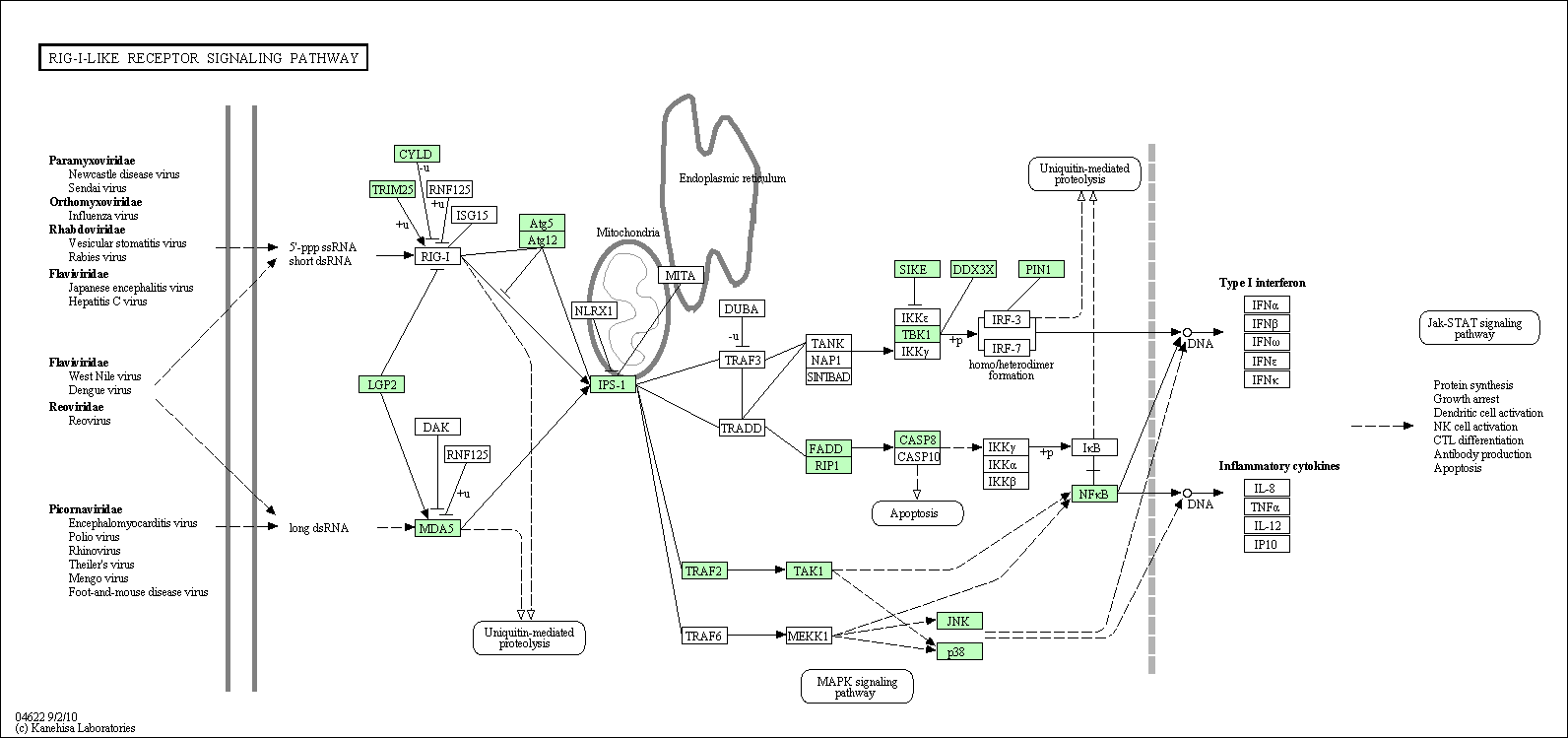

Supplement: Additional file 6 — KEGG annotation results. File contains the KEGG maps where isotigs were successfully mapped. Pathway element in green boxes indicates the components represented by at least, one isotig. Gilthead sea bream isotigs were automatically annotated to KEGG maps using KAAS website [60]. The SBH method, optimized for ESTs annotation, was used against human, chimpanzee, orang-utan, rhesus, mouse, rat, dog, giant panda, cow, pig, horse, opossum, platypus, chicken, clawed frog, zebrafish, fruit fly and nematode pathway databases. [file 1471-2164-13-181-S6.tar › map/map04622.png]

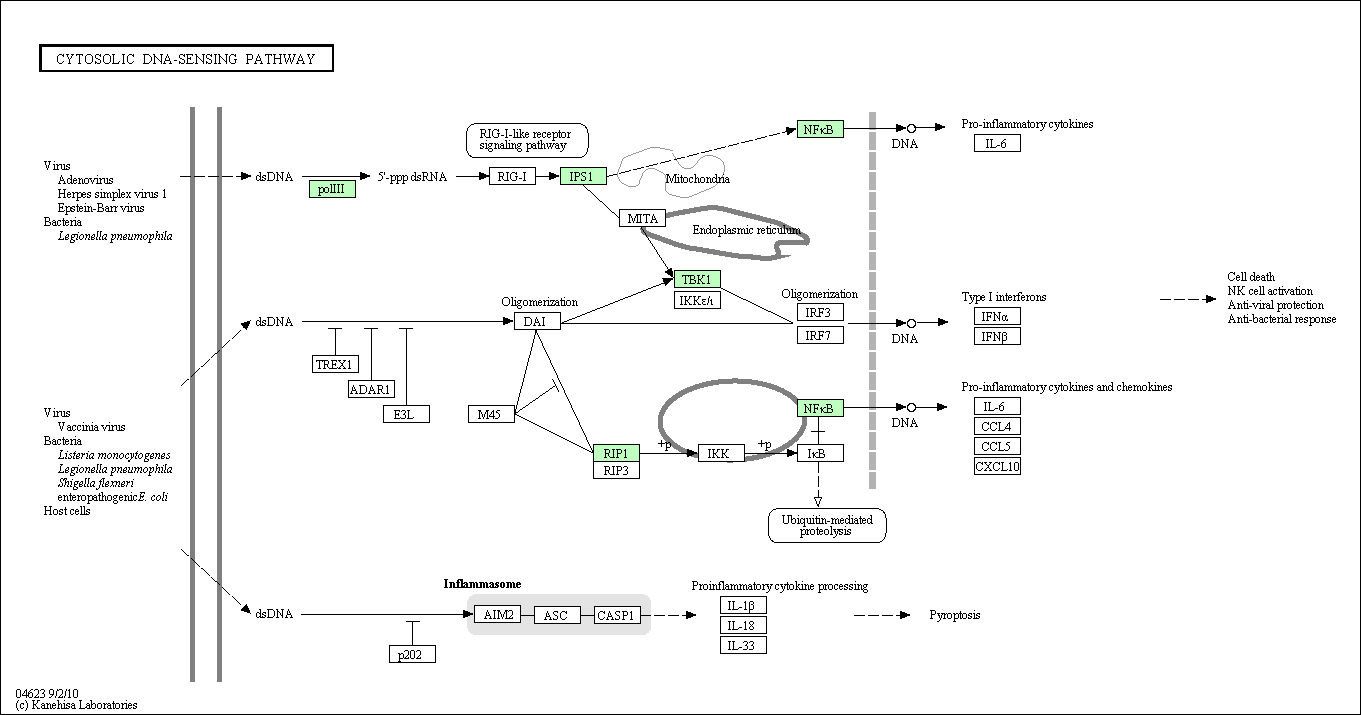

Supplement: Additional file 6 — KEGG annotation results. File contains the KEGG maps where isotigs were successfully mapped. Pathway element in green boxes indicates the components represented by at least, one isotig. Gilthead sea bream isotigs were automatically annotated to KEGG maps using KAAS website [60]. The SBH method, optimized for ESTs annotation, was used against human, chimpanzee, orang-utan, rhesus, mouse, rat, dog, giant panda, cow, pig, horse, opossum, platypus, chicken, clawed frog, zebrafish, fruit fly and nematode pathway databases. [file 1471-2164-13-181-S6.tar › map/map04623.png]

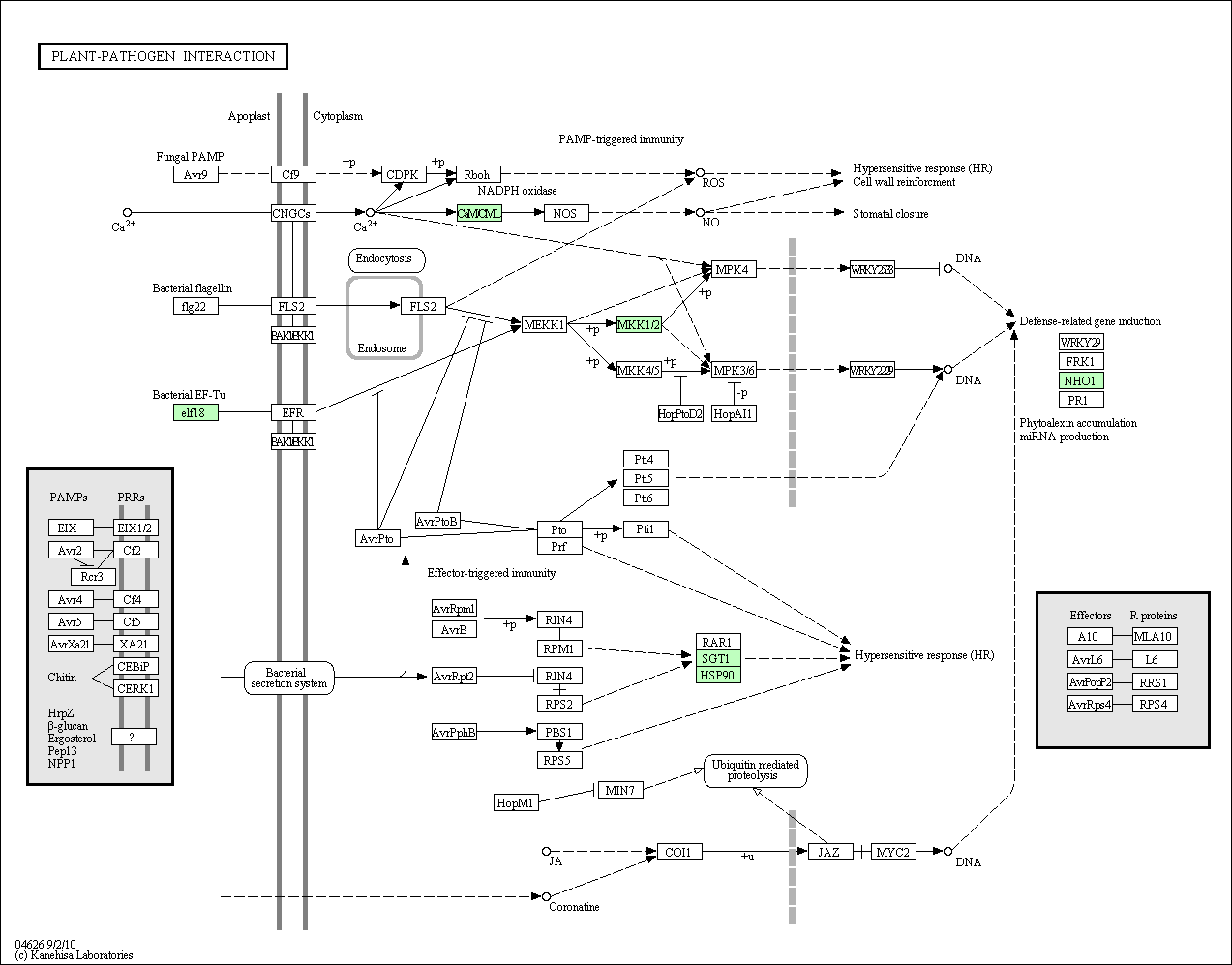

Supplement: Additional file 6 — KEGG annotation results. File contains the KEGG maps where isotigs were successfully mapped. Pathway element in green boxes indicates the components represented by at least, one isotig. Gilthead sea bream isotigs were automatically annotated to KEGG maps using KAAS website [60]. The SBH method, optimized for ESTs annotation, was used against human, chimpanzee, orang-utan, rhesus, mouse, rat, dog, giant panda, cow, pig, horse, opossum, platypus, chicken, clawed frog, zebrafish, fruit fly and nematode pathway databases. [file 1471-2164-13-181-S6.tar › map/map04626.png]

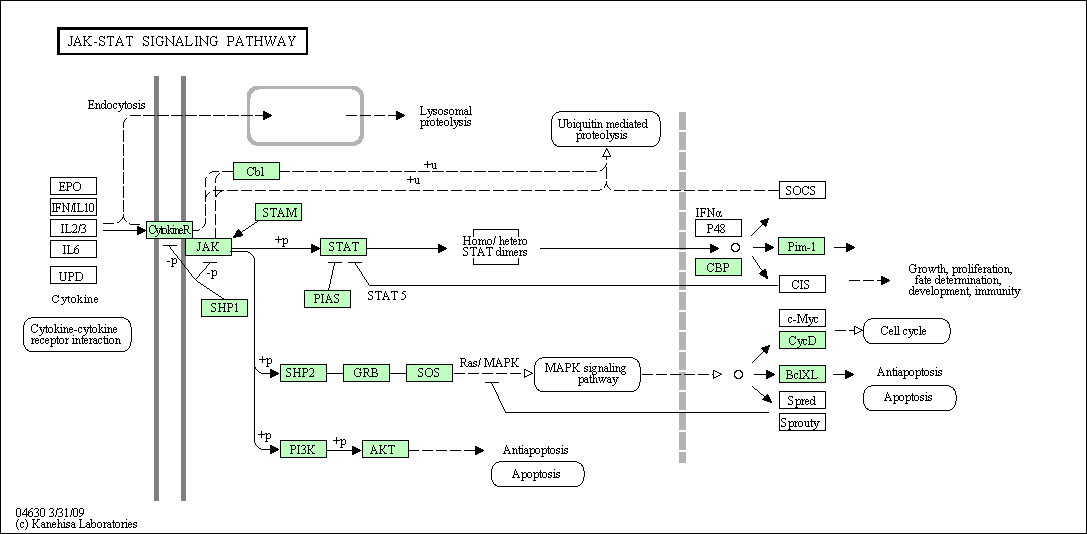

Supplement: Additional file 6 — KEGG annotation results. File contains the KEGG maps where isotigs were successfully mapped. Pathway element in green boxes indicates the components represented by at least, one isotig. Gilthead sea bream isotigs were automatically annotated to KEGG maps using KAAS website [60]. The SBH method, optimized for ESTs annotation, was used against human, chimpanzee, orang-utan, rhesus, mouse, rat, dog, giant panda, cow, pig, horse, opossum, platypus, chicken, clawed frog, zebrafish, fruit fly and nematode pathway databases. [file 1471-2164-13-181-S6.tar › map/map04630.png]

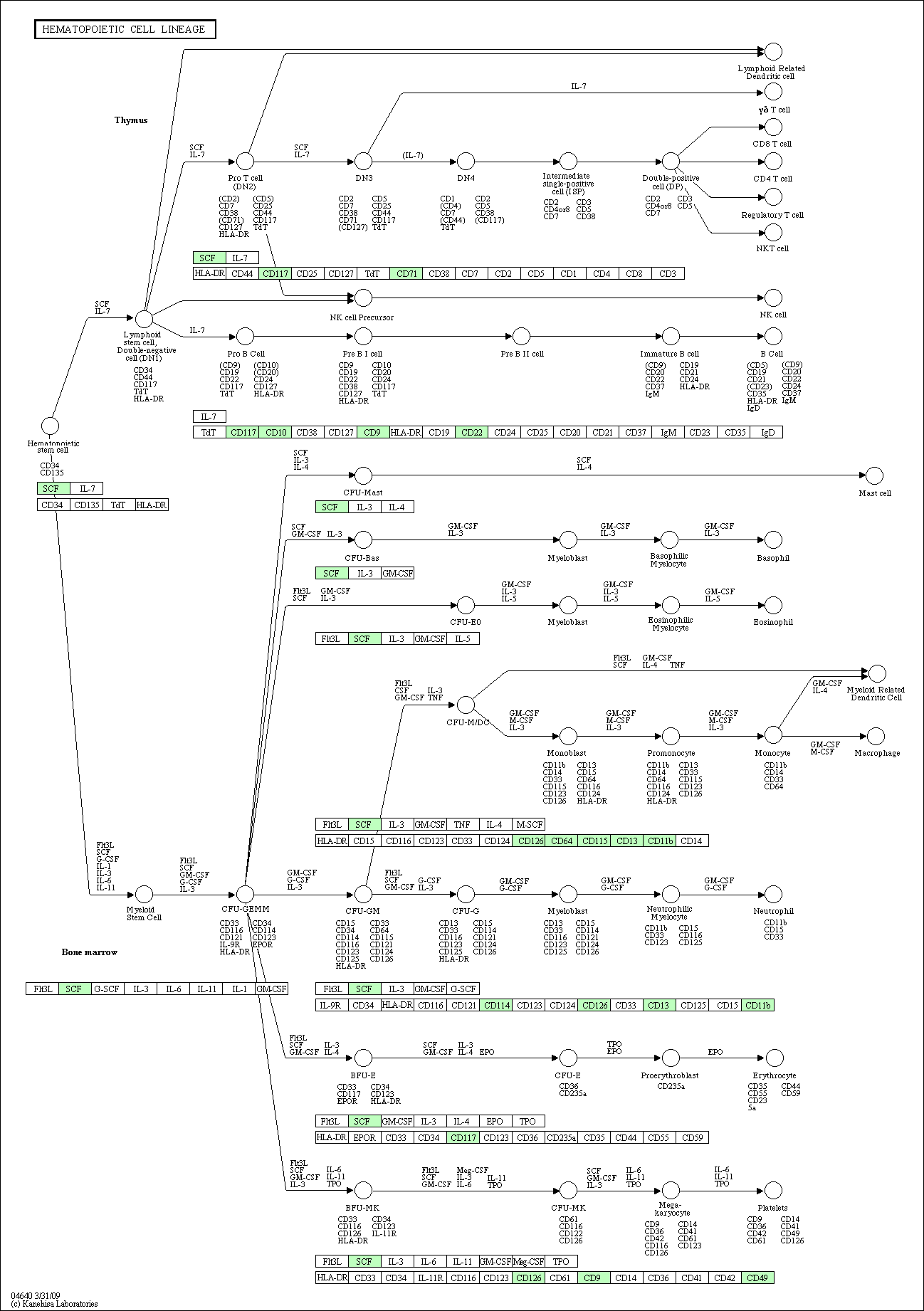

Supplement: Additional file 6 — KEGG annotation results. File contains the KEGG maps where isotigs were successfully mapped. Pathway element in green boxes indicates the components represented by at least, one isotig. Gilthead sea bream isotigs were automatically annotated to KEGG maps using KAAS website [60]. The SBH method, optimized for ESTs annotation, was used against human, chimpanzee, orang-utan, rhesus, mouse, rat, dog, giant panda, cow, pig, horse, opossum, platypus, chicken, clawed frog, zebrafish, fruit fly and nematode pathway databases. [file 1471-2164-13-181-S6.tar › map/map04640.png]

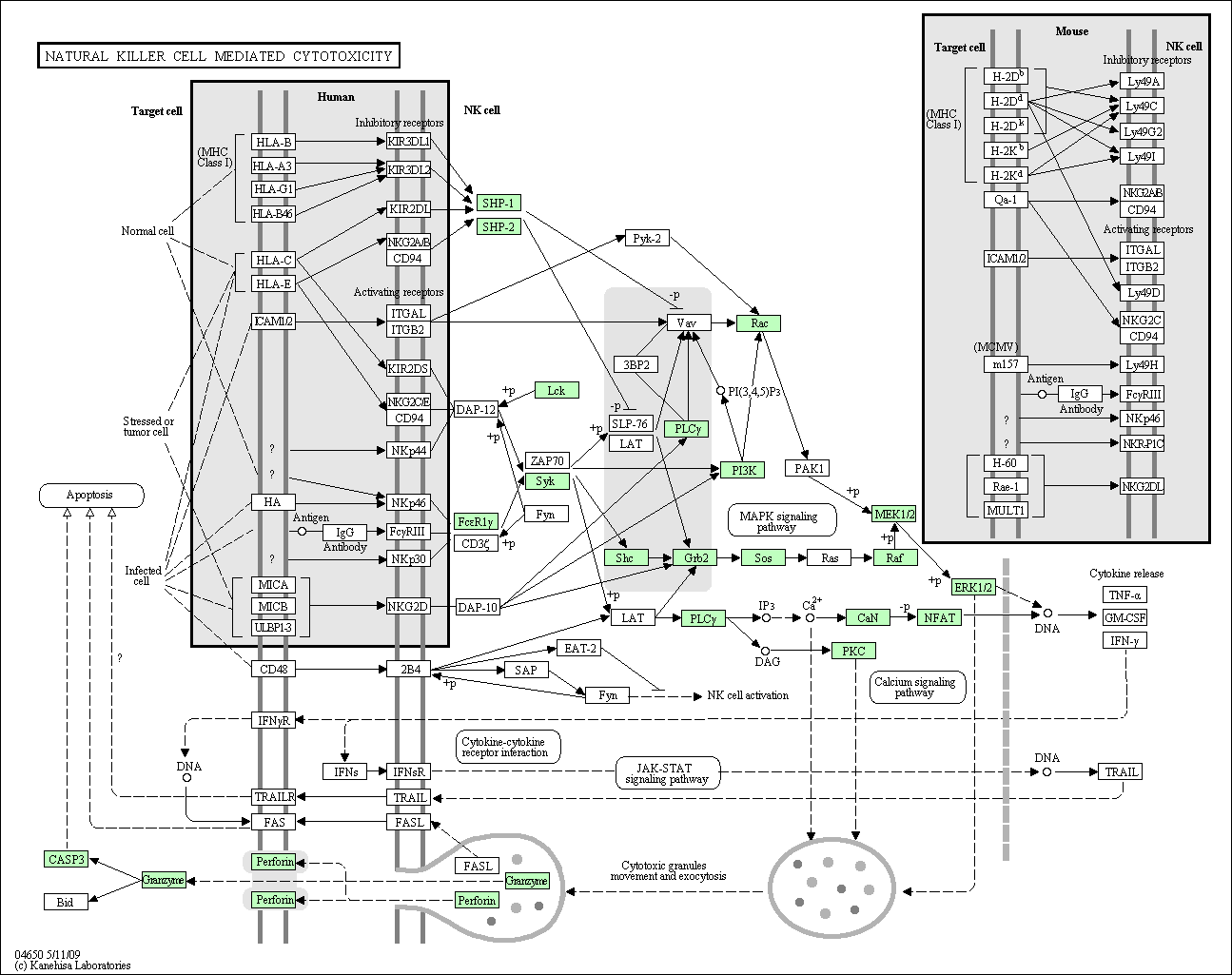

Supplement: Additional file 6 — KEGG annotation results. File contains the KEGG maps where isotigs were successfully mapped. Pathway element in green boxes indicates the components represented by at least, one isotig. Gilthead sea bream isotigs were automatically annotated to KEGG maps using KAAS website [60]. The SBH method, optimized for ESTs annotation, was used against human, chimpanzee, orang-utan, rhesus, mouse, rat, dog, giant panda, cow, pig, horse, opossum, platypus, chicken, clawed frog, zebrafish, fruit fly and nematode pathway databases. [file 1471-2164-13-181-S6.tar › map/map04650.png]

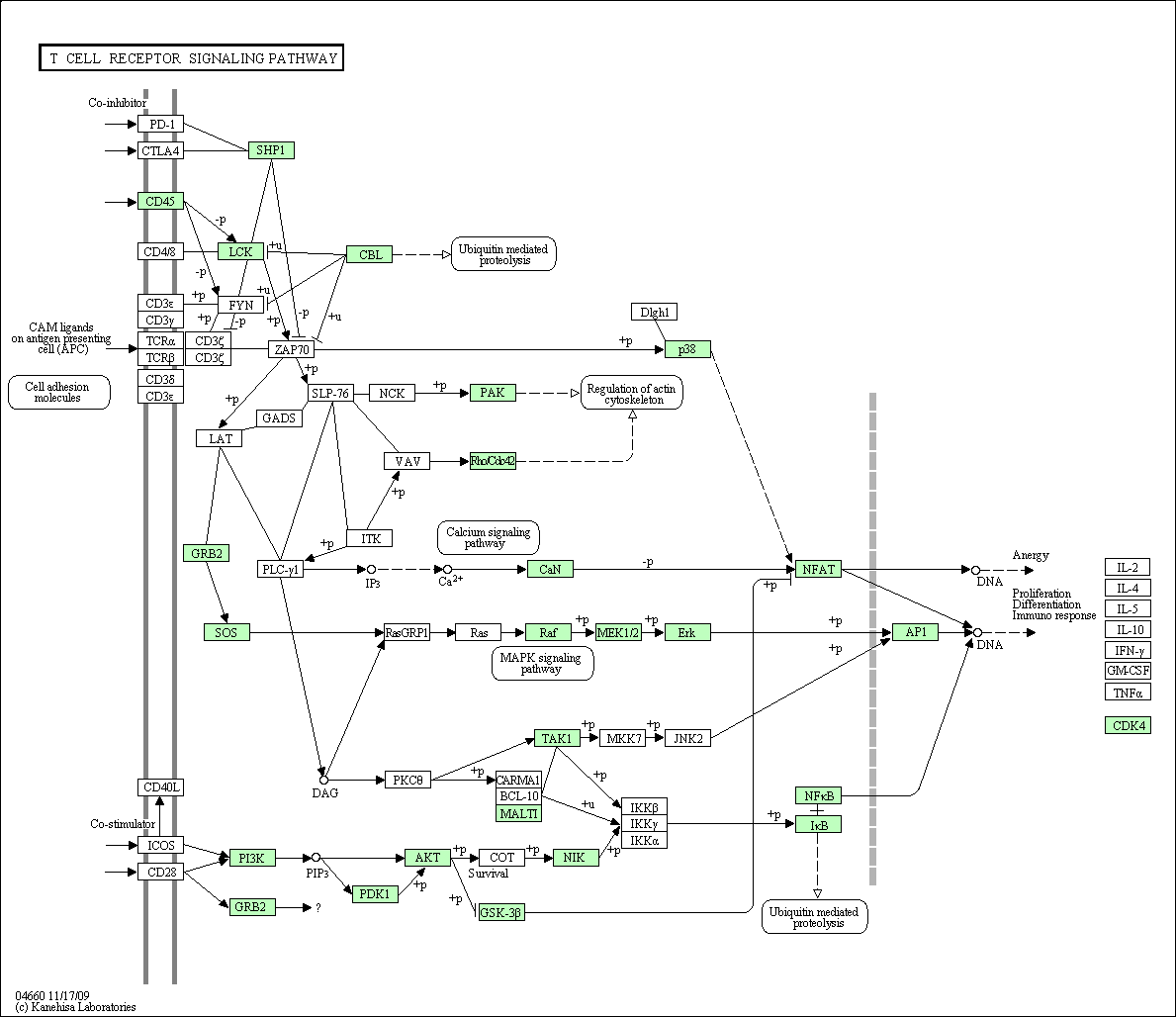

Supplement: Additional file 6 — KEGG annotation results. File contains the KEGG maps where isotigs were successfully mapped. Pathway element in green boxes indicates the components represented by at least, one isotig. Gilthead sea bream isotigs were automatically annotated to KEGG maps using KAAS website [60]. The SBH method, optimized for ESTs annotation, was used against human, chimpanzee, orang-utan, rhesus, mouse, rat, dog, giant panda, cow, pig, horse, opossum, platypus, chicken, clawed frog, zebrafish, fruit fly and nematode pathway databases. [file 1471-2164-13-181-S6.tar › map/map04660.png]

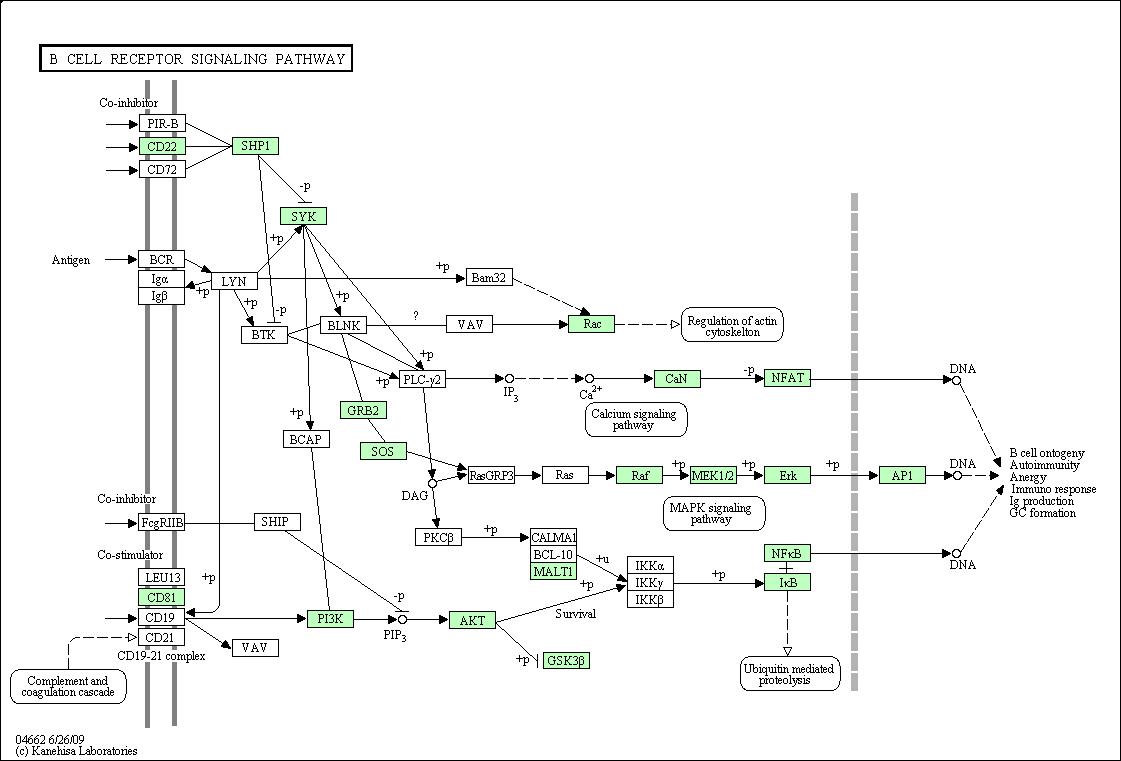

Supplement: Additional file 6 — KEGG annotation results. File contains the KEGG maps where isotigs were successfully mapped. Pathway element in green boxes indicates the components represented by at least, one isotig. Gilthead sea bream isotigs were automatically annotated to KEGG maps using KAAS website [60]. The SBH method, optimized for ESTs annotation, was used against human, chimpanzee, orang-utan, rhesus, mouse, rat, dog, giant panda, cow, pig, horse, opossum, platypus, chicken, clawed frog, zebrafish, fruit fly and nematode pathway databases. [file 1471-2164-13-181-S6.tar › map/map04662.png]

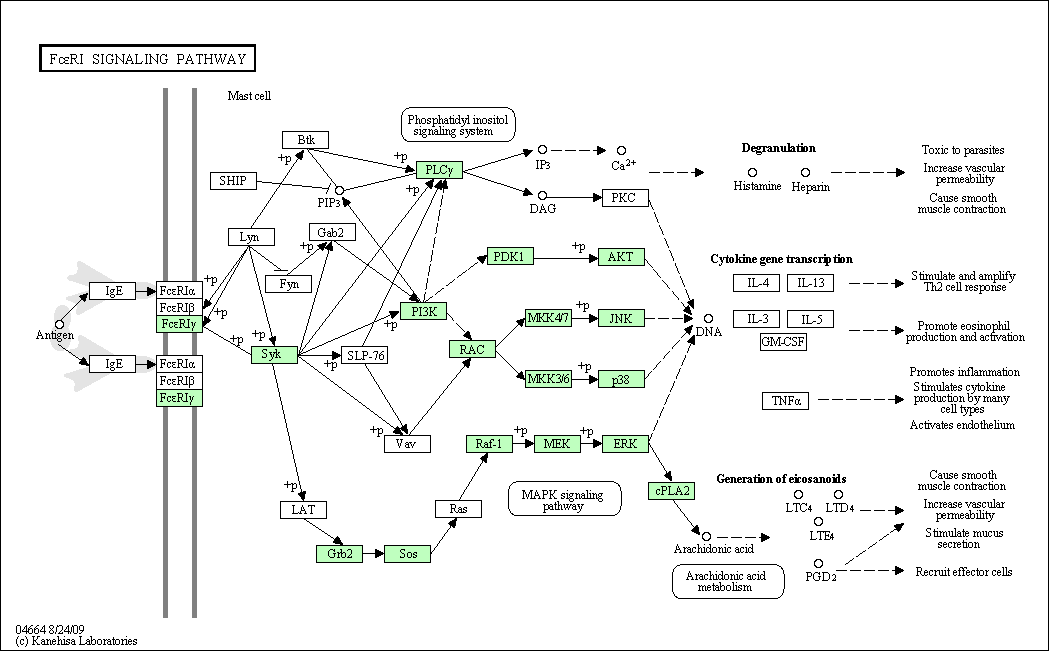

Supplement: Additional file 6 — KEGG annotation results. File contains the KEGG maps where isotigs were successfully mapped. Pathway element in green boxes indicates the components represented by at least, one isotig. Gilthead sea bream isotigs were automatically annotated to KEGG maps using KAAS website [60]. The SBH method, optimized for ESTs annotation, was used against human, chimpanzee, orang-utan, rhesus, mouse, rat, dog, giant panda, cow, pig, horse, opossum, platypus, chicken, clawed frog, zebrafish, fruit fly and nematode pathway databases. [file 1471-2164-13-181-S6.tar › map/map04664.png]

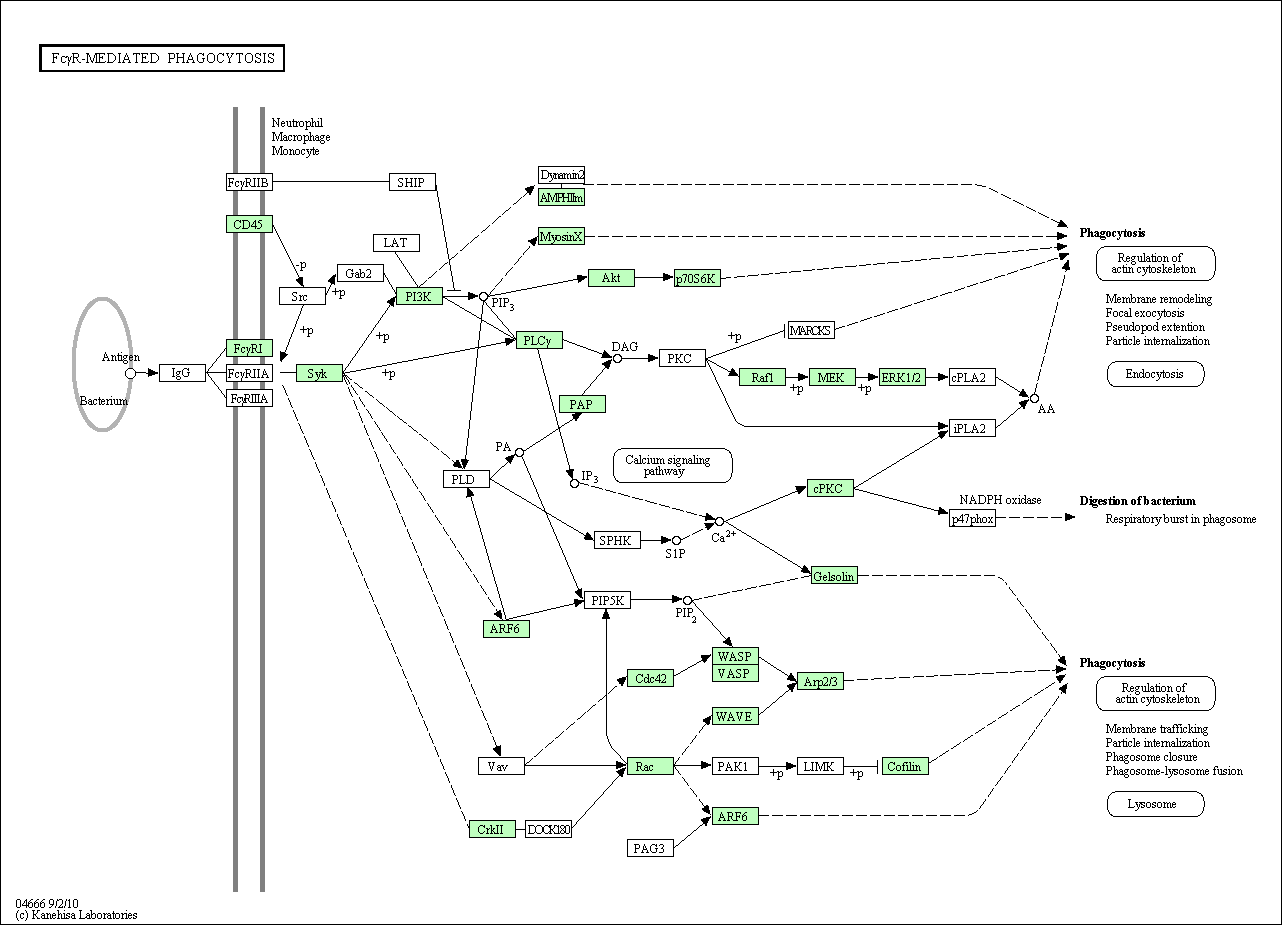

Supplement: Additional file 6 — KEGG annotation results. File contains the KEGG maps where isotigs were successfully mapped. Pathway element in green boxes indicates the components represented by at least, one isotig. Gilthead sea bream isotigs were automatically annotated to KEGG maps using KAAS website [60]. The SBH method, optimized for ESTs annotation, was used against human, chimpanzee, orang-utan, rhesus, mouse, rat, dog, giant panda, cow, pig, horse, opossum, platypus, chicken, clawed frog, zebrafish, fruit fly and nematode pathway databases. [file 1471-2164-13-181-S6.tar › map/map04666.png]

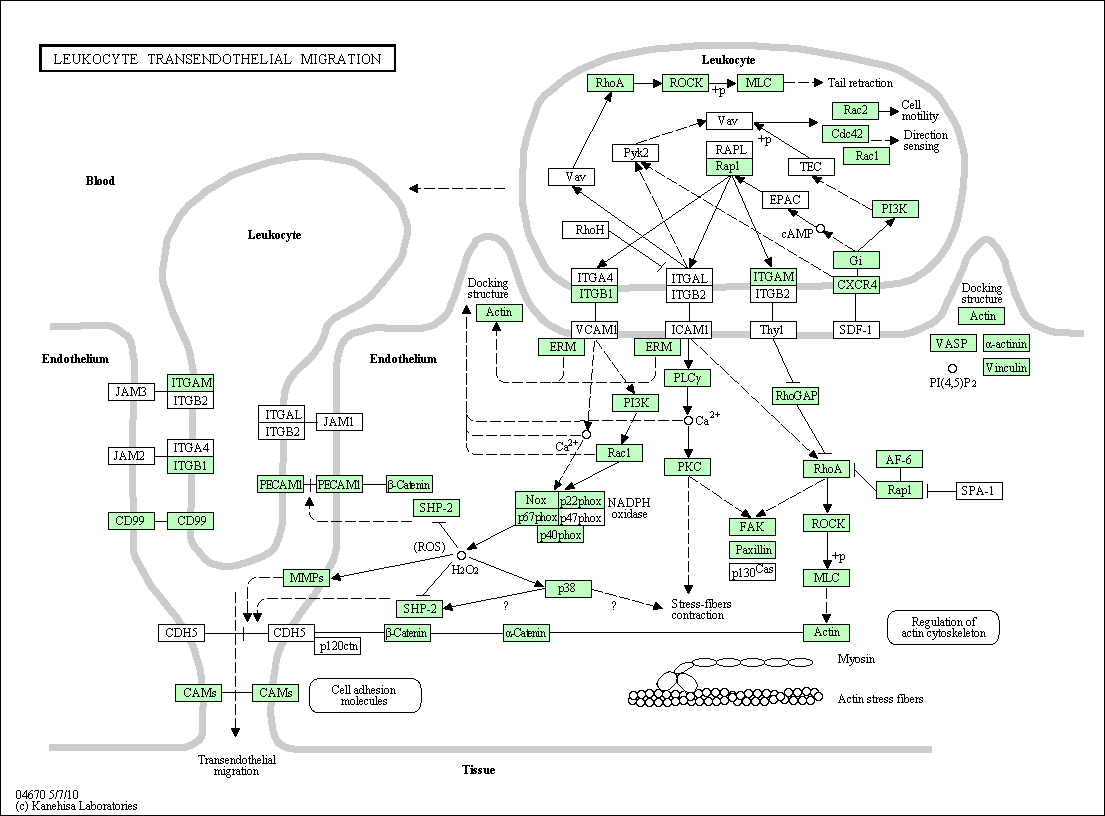

Supplement: Additional file 6 — KEGG annotation results. File contains the KEGG maps where isotigs were successfully mapped. Pathway element in green boxes indicates the components represented by at least, one isotig. Gilthead sea bream isotigs were automatically annotated to KEGG maps using KAAS website [60]. The SBH method, optimized for ESTs annotation, was used against human, chimpanzee, orang-utan, rhesus, mouse, rat, dog, giant panda, cow, pig, horse, opossum, platypus, chicken, clawed frog, zebrafish, fruit fly and nematode pathway databases. [file 1471-2164-13-181-S6.tar › map/map04670.png]

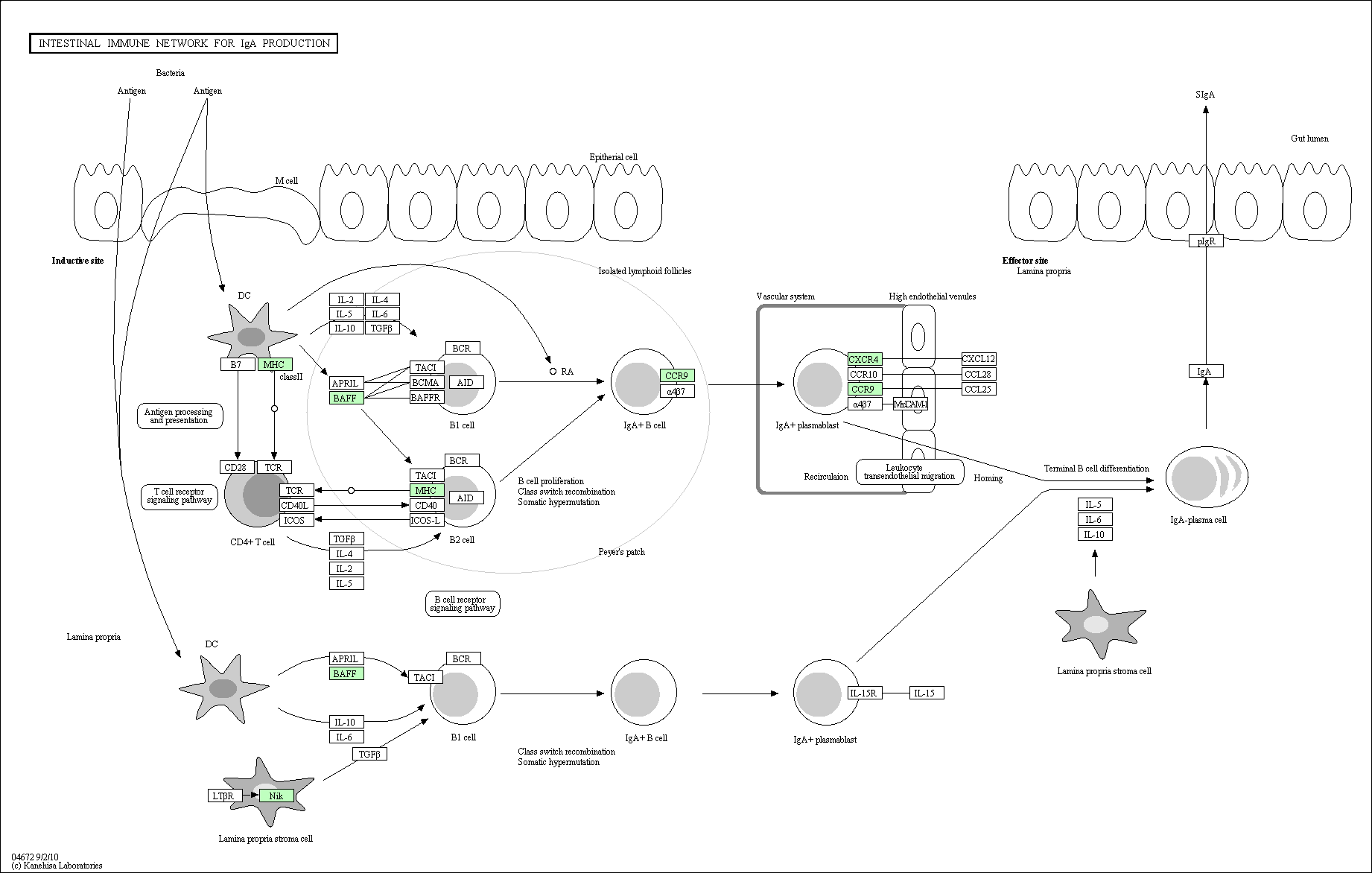

Supplement: Additional file 6 — KEGG annotation results. File contains the KEGG maps where isotigs were successfully mapped. Pathway element in green boxes indicates the components represented by at least, one isotig. Gilthead sea bream isotigs were automatically annotated to KEGG maps using KAAS website [60]. The SBH method, optimized for ESTs annotation, was used against human, chimpanzee, orang-utan, rhesus, mouse, rat, dog, giant panda, cow, pig, horse, opossum, platypus, chicken, clawed frog, zebrafish, fruit fly and nematode pathway databases. [file 1471-2164-13-181-S6.tar › map/map04672.png]

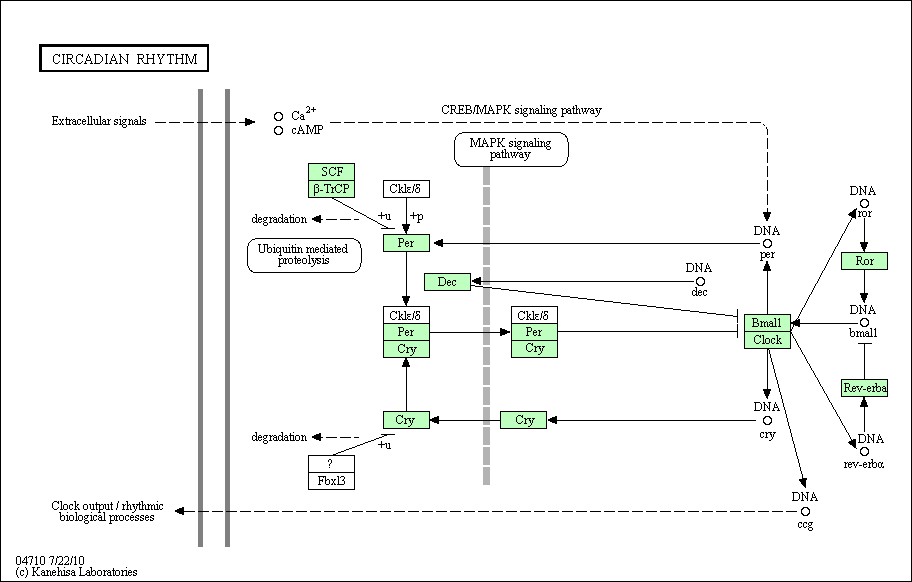

Supplement: Additional file 6 — KEGG annotation results. File contains the KEGG maps where isotigs were successfully mapped. Pathway element in green boxes indicates the components represented by at least, one isotig. Gilthead sea bream isotigs were automatically annotated to KEGG maps using KAAS website [60]. The SBH method, optimized for ESTs annotation, was used against human, chimpanzee, orang-utan, rhesus, mouse, rat, dog, giant panda, cow, pig, horse, opossum, platypus, chicken, clawed frog, zebrafish, fruit fly and nematode pathway databases. [file 1471-2164-13-181-S6.tar › map/map04710.png]

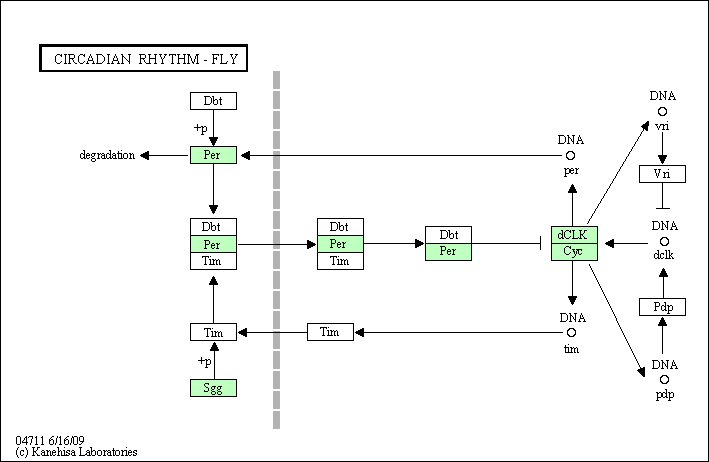

Supplement: Additional file 6 — KEGG annotation results. File contains the KEGG maps where isotigs were successfully mapped. Pathway element in green boxes indicates the components represented by at least, one isotig. Gilthead sea bream isotigs were automatically annotated to KEGG maps using KAAS website [60]. The SBH method, optimized for ESTs annotation, was used against human, chimpanzee, orang-utan, rhesus, mouse, rat, dog, giant panda, cow, pig, horse, opossum, platypus, chicken, clawed frog, zebrafish, fruit fly and nematode pathway databases. [file 1471-2164-13-181-S6.tar › map/map04711.png]

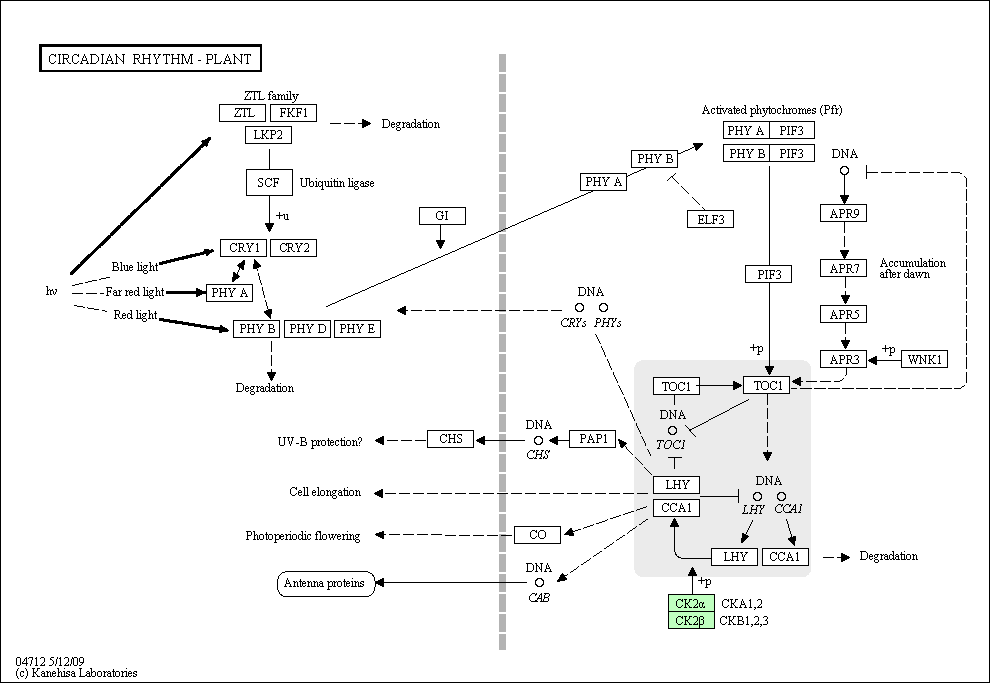

Supplement: Additional file 6 — KEGG annotation results. File contains the KEGG maps where isotigs were successfully mapped. Pathway element in green boxes indicates the components represented by at least, one isotig. Gilthead sea bream isotigs were automatically annotated to KEGG maps using KAAS website [60]. The SBH method, optimized for ESTs annotation, was used against human, chimpanzee, orang-utan, rhesus, mouse, rat, dog, giant panda, cow, pig, horse, opossum, platypus, chicken, clawed frog, zebrafish, fruit fly and nematode pathway databases. [file 1471-2164-13-181-S6.tar › map/map04712.png]

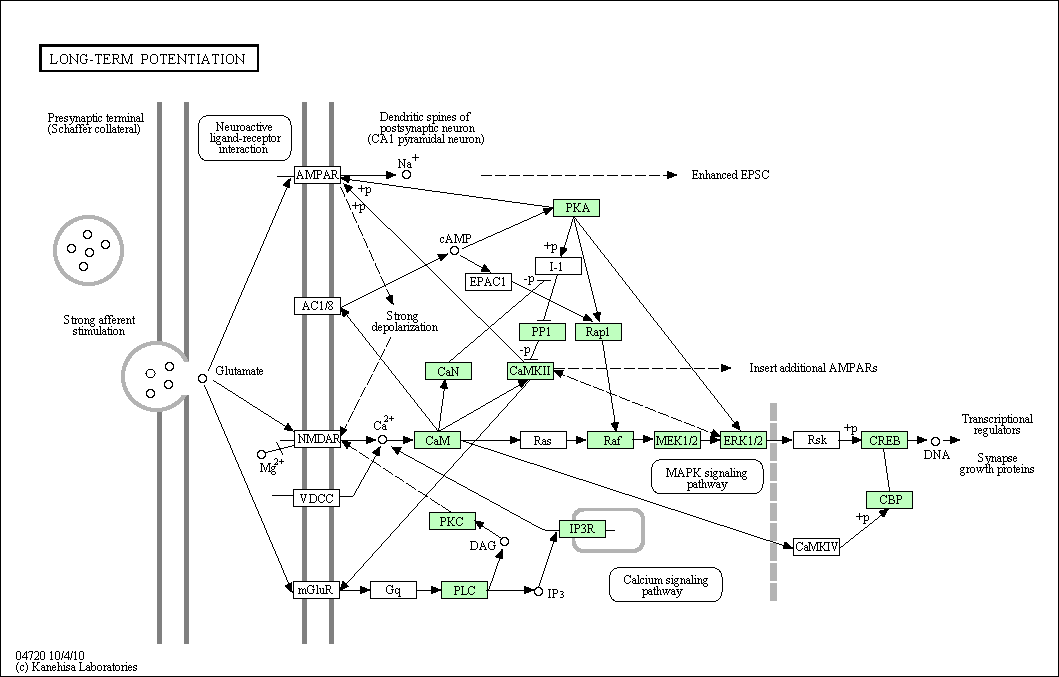

Supplement: Additional file 6 — KEGG annotation results. File contains the KEGG maps where isotigs were successfully mapped. Pathway element in green boxes indicates the components represented by at least, one isotig. Gilthead sea bream isotigs were automatically annotated to KEGG maps using KAAS website [60]. The SBH method, optimized for ESTs annotation, was used against human, chimpanzee, orang-utan, rhesus, mouse, rat, dog, giant panda, cow, pig, horse, opossum, platypus, chicken, clawed frog, zebrafish, fruit fly and nematode pathway databases. [file 1471-2164-13-181-S6.tar › map/map04720.png]

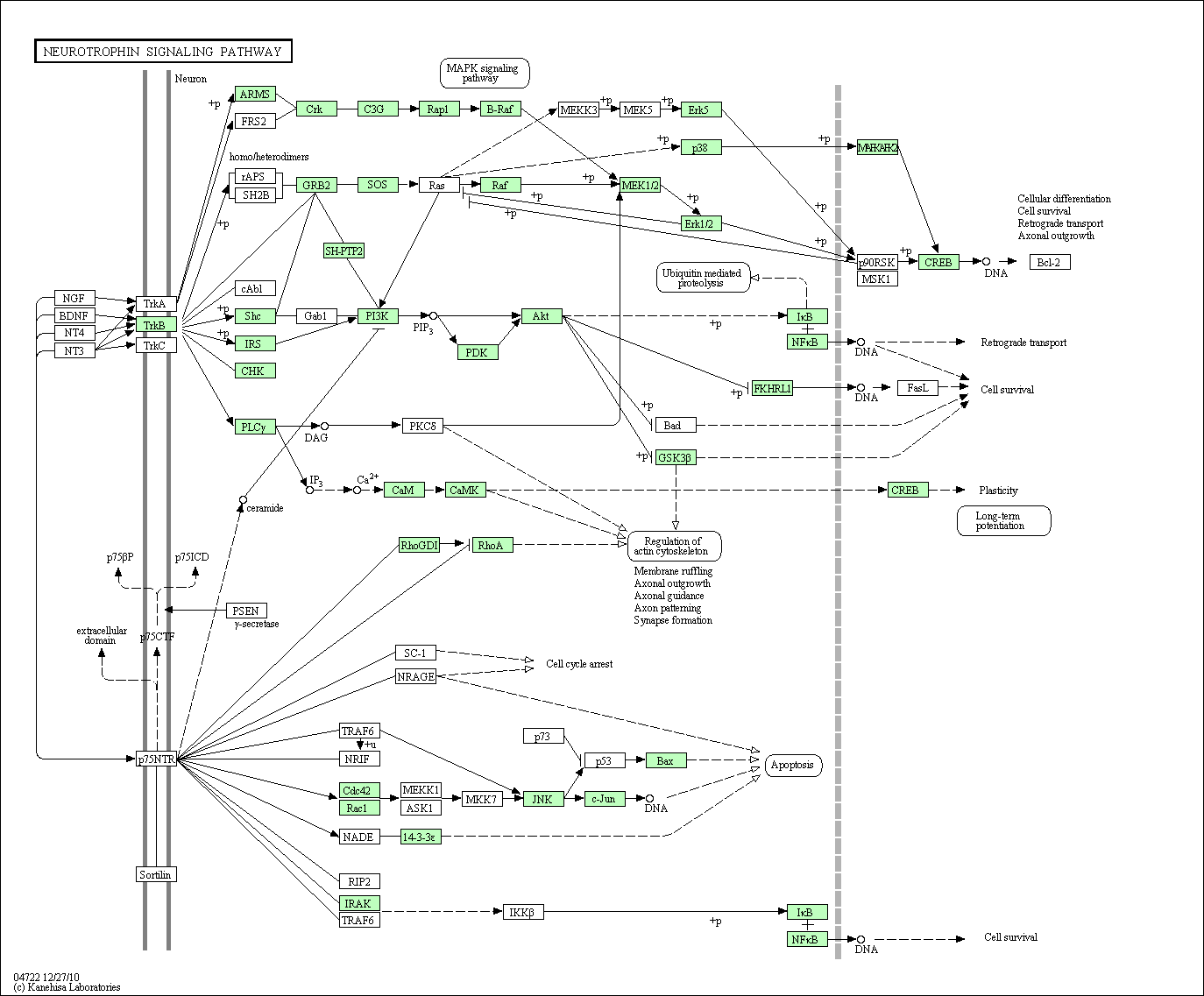

Supplement: Additional file 6 — KEGG annotation results. File contains the KEGG maps where isotigs were successfully mapped. Pathway element in green boxes indicates the components represented by at least, one isotig. Gilthead sea bream isotigs were automatically annotated to KEGG maps using KAAS website [60]. The SBH method, optimized for ESTs annotation, was used against human, chimpanzee, orang-utan, rhesus, mouse, rat, dog, giant panda, cow, pig, horse, opossum, platypus, chicken, clawed frog, zebrafish, fruit fly and nematode pathway databases. [file 1471-2164-13-181-S6.tar › map/map04722.png]

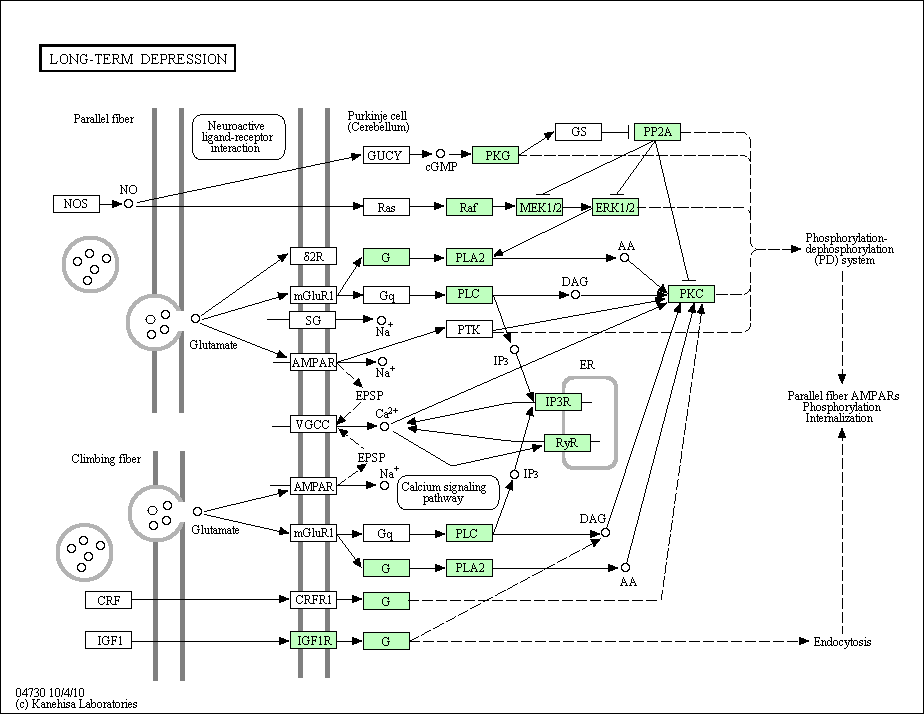

Supplement: Additional file 6 — KEGG annotation results. File contains the KEGG maps where isotigs were successfully mapped. Pathway element in green boxes indicates the components represented by at least, one isotig. Gilthead sea bream isotigs were automatically annotated to KEGG maps using KAAS website [60]. The SBH method, optimized for ESTs annotation, was used against human, chimpanzee, orang-utan, rhesus, mouse, rat, dog, giant panda, cow, pig, horse, opossum, platypus, chicken, clawed frog, zebrafish, fruit fly and nematode pathway databases. [file 1471-2164-13-181-S6.tar › map/map04730.png]

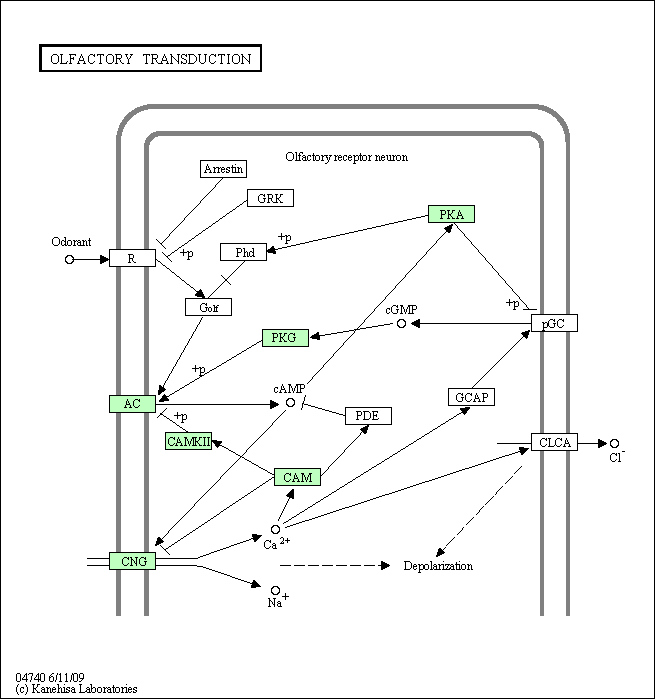

Supplement: Additional file 6 — KEGG annotation results. File contains the KEGG maps where isotigs were successfully mapped. Pathway element in green boxes indicates the components represented by at least, one isotig. Gilthead sea bream isotigs were automatically annotated to KEGG maps using KAAS website [60]. The SBH method, optimized for ESTs annotation, was used against human, chimpanzee, orang-utan, rhesus, mouse, rat, dog, giant panda, cow, pig, horse, opossum, platypus, chicken, clawed frog, zebrafish, fruit fly and nematode pathway databases. [file 1471-2164-13-181-S6.tar › map/map04740.png]

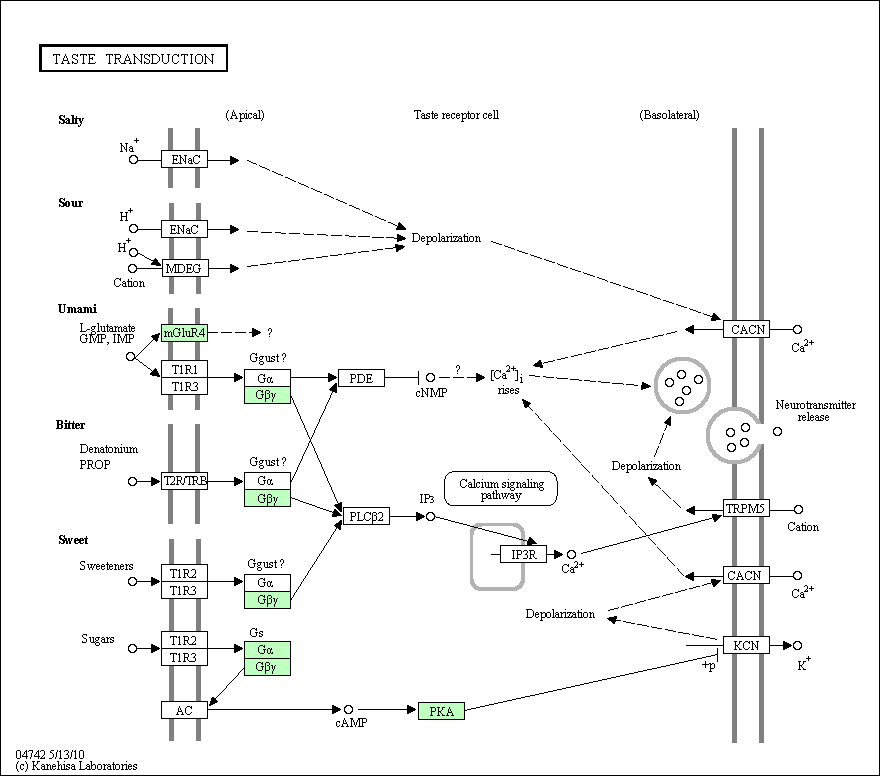

Supplement: Additional file 6 — KEGG annotation results. File contains the KEGG maps where isotigs were successfully mapped. Pathway element in green boxes indicates the components represented by at least, one isotig. Gilthead sea bream isotigs were automatically annotated to KEGG maps using KAAS website [60]. The SBH method, optimized for ESTs annotation, was used against human, chimpanzee, orang-utan, rhesus, mouse, rat, dog, giant panda, cow, pig, horse, opossum, platypus, chicken, clawed frog, zebrafish, fruit fly and nematode pathway databases. [file 1471-2164-13-181-S6.tar › map/map04742.png]

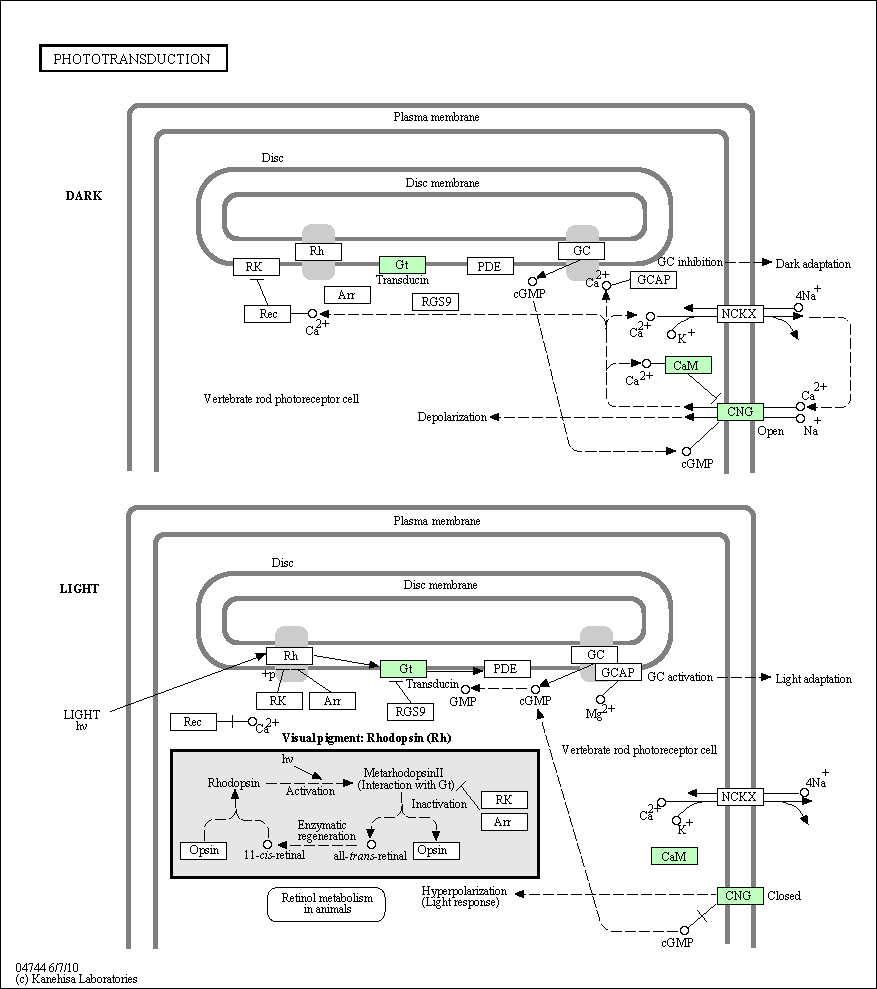

Supplement: Additional file 6 — KEGG annotation results. File contains the KEGG maps where isotigs were successfully mapped. Pathway element in green boxes indicates the components represented by at least, one isotig. Gilthead sea bream isotigs were automatically annotated to KEGG maps using KAAS website [60]. The SBH method, optimized for ESTs annotation, was used against human, chimpanzee, orang-utan, rhesus, mouse, rat, dog, giant panda, cow, pig, horse, opossum, platypus, chicken, clawed frog, zebrafish, fruit fly and nematode pathway databases. [file 1471-2164-13-181-S6.tar › map/map04744.png]

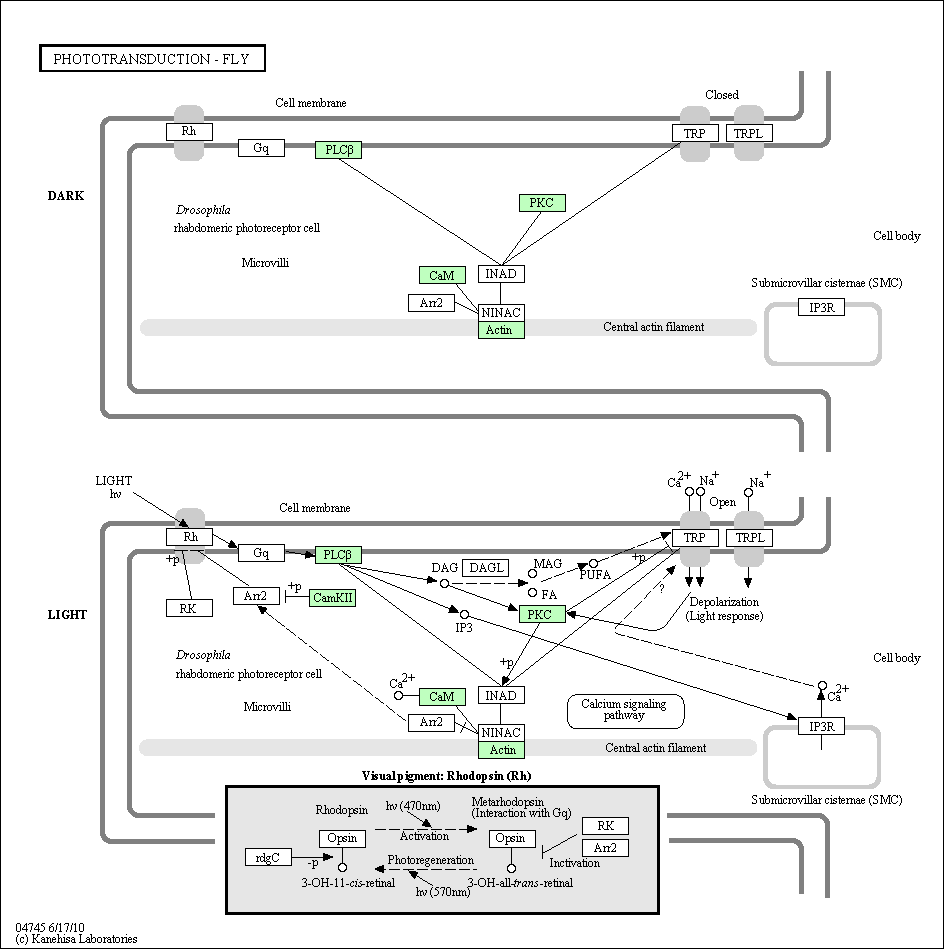

Supplement: Additional file 6 — KEGG annotation results. File contains the KEGG maps where isotigs were successfully mapped. Pathway element in green boxes indicates the components represented by at least, one isotig. Gilthead sea bream isotigs were automatically annotated to KEGG maps using KAAS website [60]. The SBH method, optimized for ESTs annotation, was used against human, chimpanzee, orang-utan, rhesus, mouse, rat, dog, giant panda, cow, pig, horse, opossum, platypus, chicken, clawed frog, zebrafish, fruit fly and nematode pathway databases. [file 1471-2164-13-181-S6.tar › map/map04745.png]
