# Supplementary material for: Fast skeletal muscle transcriptome of the Gilthead sea bream (Sparus aurata) determined by next generation sequencing
Source: BMC Genomics. 2012 May 11;13:181. doi: 10.1186/1471-2164-13-181 (PMC3418159; doi:10.1186/1471-2164-13-181)
Supplement: Additional file 10 — Predicted amino acids sequences of splice variants. [file 1471-2164-13-181-S10.rtf]

>isotig03704_ORF|adenosylmethionine|adenosylmethionine decarboxylase 1MMEDNGAHFFEGTEKLLEVWFSRQDETKGTGDLRTIPRFEWDKLLENVHCLIISVTKSDKQEAYILSESSMFVSKRRFILKTCGTTLLLQALVPLLELAREYCGFDAIENFFYSRKNFMKPTHQEFPHRNFQEEVDFLSQIFPNGAAYCMGRLNSDCW>isotig03705_ORF|s-adenosylmethionine|s-adenosylmethionine decarboxylaseMFVSKRRFILKTCGTTLLLQALVPLLELAREYCGFDAIENFFYSRKNFMKPTHQEFPHRNFQEEVDFLSQIFPNGAAYCMGRLNSDCW>isotig04031_ORF|alpha-enolase|alpha-enolaseMSIVKIHAREIFDSRGNPTVEVDLYTDKGLFRAAVPSGASTGIYEALELRDNDKSRYLGKGVSKAVENINSTIAPALVGQDVSVVEQERIDQMMLDLDGTENKSKLGANAILGVSLAVCKAGAAEKGVPLYRHIADLAENPEVILPVPAFNVINGGSHAGNKLAMQEFMILPVGASTFKEAMRIGAEVYHNLKN>isotig04032_ORF|alpha-enolase|alpha-enolaseMSIVKIHAREIFDSRGNPTVEVDLYTDKGLFRAAVPSGASTGIYEALELRDNDKSRYLGKGVSKAVENINSTIAPALVGQDVSVVEQERIDQMMLDLAVCKAGAAEKGVPLYRHIADLAENPEVILPVPAFNVINGGSHAGNKLAMQEFMILPVGASTFKEAMRIGAEVYHNLKN>isotig03345_ORF|archaemetzincin-2|archaelysin family metallopeptidase 2VKLMPAVTVAETGCSFRVNSHSYNLQILTGDLLRFLGSRKPKDAFCIVGITMIDLYPKESWNFVFGQASLSMGMGVFSFARYDDNFYSRTYAGRLKKRLLPKQGDYSVFDGYYTPPITSTLLLRSCKTMTHEIGHMFGVKHCQWLNCVMQGSNHLEESDRRPLDFCPICLRKLQVAIGFKIAERYQALLHWIEEDQSQTSRPEEASACQSVLHYQKPTEAFHTSALWLRKCLDILEKG>isotig03346_ORF|archaemetzincin-2|archaemetzincin-2 isoform 1MTHEIGHMFGVKHCQWLNCVMQGSNHLEESDRRPLDFCPICLRKLQVAIGFKIAERYQALLHWIEEDQSQTSRPEEASACQSVLHYQKPTEAFHTSALWLRKCLDILEKG>isotig02042_ORF|aspartate|aspartate beta-hydroxylaseMGDPATVVEAATPPVVTAIPAVMAGEMQDGTKTQSANKNGKKTDSGSSSSFFTWFIVLALLGVWTSVAVVYFDLVDYQGVLGKLVAYDTDGDGDFDVEDAKVLLDETPTDAARREAQKETPPTKDVGLKLREALKQQLAIIHERVEAKKLAK>isotig02041_ORF|asph|asph proteinMGDPATVVEAATPPVVTAIPAVMAGEMQDGTKTQSANKNGKKTDSGSSSSFFTWFIVLALLGVWTSVAVVYFDLVDYQGVLDKAKGLQINLSEALQGKLVAYDTDGDGDFDVEDAKVLLDETPTDAARREAQKETPPTKDVGLKLREALKQQLAIIHERVEAKKLAK>isotig03402_ORF|bag|bag family molecular chaperone regulator 2CAKFSRTLSMADGPDDFWKLWSTLSVEISVGTIRNPMQEDALNKATAIIDELVKKLLDDMECVTEAPPCPIDQKFQAIVISCALEDQKKIKRRLETLMRNVENAERNIKIMDHQKLEQPKANGC>isotig03401_ORF|bcl2-associated|bcl2-associated athanogene 2MAQAKIQSKMTEAQCAKFSRTLSMADRSGRLLETLDQLEMRVEALREAASAMEQERECILETLQSIQNGQEMRNICAGEREELDLTANRLMGRTLSVEISVGTIRNPMQEDALNKATAIIDELVKKLLDDMECGRQRLLALHAACVTEAPPCPIDQKFQAIVISCALEDQKKIKRRLETLMRNVENAERNIKIMDHQKLEQPKANGC>isotig03015_ORF|bridging|bridging integrator 1MAEPGKGVTAGKLAINVQKRLTRAQEKVMQKLGKADETRDAAFEEMVSNFNKQMTEGTKLQKDLKAYLTAVKTMHDASRRLQDCLADMYEPDWFGKEEMDTLAEDTDTLWLDYHQNIADKSLSSLDTYLAQFPDIKARIAKRDRKMVDFDSARHHFASLQKGKKKDEAKVAKAEEDLGRAQKIFEELNVELQDELPVLWENRVGVYVNSFQSLAGQQEKFHREMGKLSQNLNDIMTKLEEQRELKKGGTAAAKTGDGAKSEEANHSESASPPPKSAVTNGADGDLPPGFIYKVKVIHDYAATDGDELELKIGDTVLALAFDNPDEQDDGWLLGVQESHWLQNKDISAKGVFPENFTQKV>isotig03016_ORF|bridging|bridging integrator 1MGKLSQNLNDIMTKLEEQRELKKGGTAAAKTGDGAKSEEANHSESASPPPKSAVTNGADGDLPPGFIYKVKVIHDYAATDGDELELKIGDTVLALAFDNPDEQDDGWLLGVQESHWLQNKDISAKGVFPENFTQKV>isotig02977_ORF|c4b-binding|c4b-binding protein alpha chainMGVTDFLLLSCLGFAITAHAQDCAKPTGGPNMHLKGEDILKETFADGDRAVFECAAGYMSAGGSSSITCTAGSWSTVTLKCERKNCGSPGEVPNGQIHYPQENLFGDYIEITCNEGYRLVGNSRITCGAQGWMGRLPRCEVAKCDEAPQVTDATFTPYKDPYDYGDVVQYSCRNGLTLNGSASIECSDDGTFKPAPPICTRVQCEDLDIPNLDWISGSRPPHGYKAILTYRCRTGYRMVGQPTLTCDINSKWSPGLPRCEMVTCNPPHLVVNGTFNPVKDVYKYGDRVQYSCNTNFRLSGSNSASCSDDGTFKPEPPTCTKFIPPPQPDMYYEAPQVENGYLAHDDRPLYRPNDKVTFKCRWFYYMEGDSTLTCQADGQWSPPPPKCAYSHGKVAGVVIGVITATGAVVVVFVYIMKKDQSFRKNNNGNSTNDKRGKSVGDAVGLMETPTGNGQTTEDKPPVGSANEV>isotig02978_ORF|c4b-binding|c4b-binding protein alpha chainMGVTDFLLLSCLGFAITAHAQDCAKPTGGPNMHLKGEDILKETFADGDRAVFECAAGYMSAGGSSSITCTAGSWSTVTLKCERKNCGSPGEVPNGQIHYPQENLFGDYIEITCNEGYRLVGNSRITCGAQGWMGRLPRCEVAKCDEAPQVTDATFTPYKDPYDYGDVVQYSCRNGLTLNGSASIECSDDGTFKPAPPICTRVQCEDLDIPNLDWISGSRPPHGYKAILTYRCRTGYRMVGQPTLTCDINSKWSPGLPRCEKFIPPPQPDMYYEAPQVENGYLAHDDRPLYRPNDKVTFKCRWFYYMEGDSTLTCQADGQWSPPPPKCAYSHGKVAGVVIGVITATGAVVVVFVYIMKKDQSFRKNNNGNSTNDKRGKSVGDAVGLMETPTGNGQTTEDKPPVGSANEV>isotig02938_ORF|calpain-3|calpain-3 isoform cMKIEWKRPSEICENPQFIIDGANRTDICQGELGDCWLLAAIACLTVNEKLLYRVIPPDQSFTENYAGIFHFQFWRYGEWIDVVVDDRIPTCNNKLVFTKSFRNNEFWSALLEKAYAKLHGSYEALKGGNTLEAMEDFTGGVTEFFELSEAPKDLYSIMRKALQRGSLMGCSIDVFSASELESRTDLGLVRGHAYSIIGLEECDEVAKNTKVRLIRLRNPWGFVLWKGPWSVNSKEWSTISTADRENLKKQTIETSEFWMSFDDFKRNFTKLEMCNLTPDTLQCDERHSWTVSVNEGRWVRGSSAGGCRNFPETFWTNPQYRLKLYEEDDDPEDGNMACTLVVALMQKGRRMQRHQGARFLTIGFSIYQVPKEMCGQNQHLQKDFFLYTASKAKCKTYINLREVTERFRLPPGEYVIIPTTFQPHQEGEFILRVFSEKQSTSEEVENTIGSDQTQQDKKKKEKPIVFVSDRARANKEIEHDGILGEKKKKPKRKLLEPEEETEEEKQFRAIYEQIAGEDMQICANELMKVMKNVLAKHSEIKAEGFSLETCRSMIALMDTDGTGKLNLQEFKHLWRKIKAWQLIFKRYDKDKTCSISSFEMRNAVNDAGFHLNNQLYDIIAMRYADEHLNINFDSYICCFVRLEGMFRAFNAFDKDGDGIIKLNVLEWLQLTMYS>isotig02937_ORF|calpain-3|calpain-3-like isoform 3MGDETYKGKVPLVEDTKVKVLYETQASAGPDDKAEYPPAGTNSIYSAILSRNEAVKDAKRLKTFLELRDKYVKKKVVFEDPLFPANDSSLFYSHKSAMKIEWKRPSEICENPQFIIDGANRTDICQGELGDCWLLAAIACLTVNEKLLYRVIPPDQSFTENYAGIFHFQFWRYGEWIDVVVDDRIPTCNNKLVFTKSFRNNEFWSALLEKAYAKLHGSYEALKGGNTLEAMEDFTGGVTEFFELSEAPKDLYSIMRKALQRGSLMGCSIDVFSASELESRTDLGLVRGHAYSIIGLEECDEVAKNTKVRLIRLRNPWGFVLWKGPWSVNSKEWSTISTADRENLKKQTIETSEFWMSFDDFKRNFTKLEMCNLTPDTLQCDERHSWTVSVNEGRWVRGSSAGGCRNFPETFWTNPQYRLKLYEEDDDPEDGNMACTLVVALMQKGRRMQRHQGARFLTIGFSIYQVPKEMCGQNQHLQKDFFLYTASKAKCKTYINLREVTERFRLPPGEYVIIPTTFQPHQEGEFILRVFSEKQSTSEEVENTIGSDQTQQDKKKKEKPIVFVSDRARANKEIEHDGILGEKKKKPKRKLLEPEEETEEEKQFRAIYEQIAGEDMQICANELMKVMKNVLAKHSEIKAEGFSLETCRSMIALMDTDGTGKLNLQEFKHLWRKIKAWQLIFKRYDKDKTCSISSFEMRNAVNDAGFHLNNQLYDIIAMRYADEHLNINFDSYICCFVRLEGMFRAFNAFDKDGDGIIKLNVLEWLQLTMYS>isotig03891_ORF|cathepsin|cathepsin hMGLNQFSDMTFAEFKKSFLWSEPQNCSATKGNYLRSNGPNPDSIDWRKKGNYVSPVKNQGGCGSCWTFSTTGCLESVTAINKGKLLLLSEQQLVDCAQDFNNHGCNGGLPSQAFESSCT>isotig03892_ORF|cathepsin|cathepsin hMKGFLLVALLSTASAFYLREQDEFDFKTWMAQHNKAYSTQEYNERLQIFTENKRRIDKHNAGNHSYTMGLNQFSDMTFAEFKKSFLWSEPQNCSATKGNYLRSNGPNPDSIDWRKKGNYVSPVKNQGGCGSCWTFSTTGCLESVTAINKGKLLLLSEQQLVDCAQDFNNHGCNGGLPSQAFESSCT>isotig03847_ORF|ccctc-binding|ccctc-binding factorMCDYCCRQERHMVMHRRTHTGEKPYACSQCEKTFRQKQLLDMHFKRYHDPNFVPTAFVCPKCSKTFTRRNTMARHAENCTGEVEDTENGAPTPKGRRERE>isotig03848_ORF|ccctc-binding|ccctc-binding factorSRDTYKLKRHMRTHSGEKPYECYICHARFTQSGTMKMHILQKHTENVAKFHCPHCDTVIARKSDLGVHLRKQHSFIETGKKCRYCDAVFHERYALIQHQKSHKNEKRFKCDMCDYCCRQERHMVMHRRTHTGEKPYACSQCEKTFRQKQLLDMHFKRYHDPNFVPTAFVCPKCSKTFTRRNTMARHAENCTGEVEDTENGAPTPKGRRERE>isotig02737_ORF|chromobox|chromobox protein homolog 3MHNMGKKQTTKGKKDAPETQEEPEEFVVEKVLDQRIVNGKVEFYLKWKGFTDADNTWEPEENLDCPELISGFLEAQKNVKEKPAPVKRKASTDEPEPDAKKKDVPEKPRGFARNLEPERIIGATDSSGELMFLMKWKDSDEADLVPAREANTRCPQVVISFYEERLTWHSCPEDEAQ>isotig02738_ORF|chromobox|chromobox protein homolog 3MGKKQTTKGKKDAPETQEEPEEFVVEKVLDQRIVNGKVEFYLKWKGFTDADNTWEPEENLDCPELISGFLEAQKNVKEKPAPVKRKASTDEPEPDAKKKDVPEKPRGFARNLEPERIIGATDSSGELMFLMKWKDSDEADLVPAREANTRCPQVVISFYEERLTWHSCPEDEAQ>isotig02180_ORF|coagulation|coagulation factor xMCSSTTSAALLCVLAGAVAAIQTGTVFLDKQQASTVISRQKRNLDGGNPPTLEQVCMEKVCTYTSYECSCAWGWRLSTLDRNKCEAVGSFPCGKIAVSSRWEERLSSNMRSNFQGLQCNPSECPWQALLTSSESTGYCSGVILKENLVLTSAQCANKYTSFSVAVGKRSTGYESGQQTLPVKNKHVHQRYLEGRPEYDLAVLVLDGRINFKKDVIAACLPEKSFAESVLMSGEYS>isotig02179_ORF|coagulation|proz proteinMCSSTTSAALLCVLAGAVAAIQTGTVFLDKQQASTVISRQKRNLDGGNPPTLEQVCMEKVCTYEQARSVFKDSYRTDIFWAVYVDGDQCAEKPCKNGAMCSDSVGGYDCVCKSGFSGVHCEKDETVCTLEKNKGCSQFCKPGYTSYECSCAWGWRLSTLDRNKCEAVGSFPCGKIAVSSRWEERLSSNMRSNFQGLQCNPSECPWQALLTSSESTGYCSGVILKENLVLTSAQCANKYTSFSVAVGKRSTGYESGQQTLPVKNKHVHQRYLEGRPEYDLAVLVLDGRINFKKDVIAACLPEKSFAESVLMSGEYS>isotig01731_ORF|cytochrome|cytochrome c oxidase subunit 4 isoform mitochondrial precursorMLRLTAERMGSLLARRAAMPLATSSARMASHGHEVATTVDMSKPLYYDRKDTPLPDRAYKDVLDTADKGLKQKEKGPWGQLSKEEKIALYRIMFCQTYAEMKQPSDEWKTVMGGIFILLGLTGLIVWWQSIYVYPERPRTFDEEWKAKQLKRMLDMRINPVEGFSAKWDYEKGQWK>isotig01732_ORF|cytochrome|cytochrome c oxidase subunit 4 isoform mitochondrial precursorMSKPLYYDRKDTPLPDRAYKDVLDTADKGLKQKEKGPWGQLSKEEKIALYRIMFCQTYAEMKQPSDEWKTVMGGIFILLGLTGLIVWWQSIYVYPERPRTFDEEWKAKQLKRMLDMRINPVEGFSAKWDYEKGQWK>isotig03543_ORF|cytoskeletal|cytoskeletal associated proteinMANVQRVQKKTSTMSLTISSSSSSASSSRELSSCSSSTASASTFLAQRHSSLVQPLCVSPQRTQSPIYNQNYSGNGVAPTFVKCLHDVSTVKGQLVVLECRLRGTPPLQVIWYREDEQVLDSDDFRILRKKASSASVPEELCTLVITEAFPEDSGLFKCVAINSFGTVSCSAILEVYEDLEEQLEIEAVHQQEAVSLRQKEEALFQQETSSSLKSSEDFPFELPDSMTLPPPEWPDSPPEDNIPLA>isotig03544_ORF|cytoskeletal|palladin isoform 1MSLTISSSSSSASSSRELSSCSSSTASASTFLAQRHSSLVQPLCVSPQRTQSPIYNQNYSGNGVAPTFVKCLHDVSTVKGQLVVLECRLRGTPPLQVIWYREDEQVLDSDDFRILRKKASSASVPEELCTLVITEAFPEDSGLFKCVAINSFGTVSCSAILEVYEDLEEQLEIEAVHQQEAVSLRQKEEALFQQETSSSLKSSEDFPFELPDSMTLPPPEWPDSPPEDNIPLA>isotig03107_ORF|dead|dead (asp-glu-ala-asp) box polypeptide 1NSGNPKLEGTAVIGGVAAKEQLAALEQGIDIVVGTPGRLDDLISTGKLSLASVRFLVLDECDGLLSAGYTDFINRIHKQIPQVTSDGKRLQVIVCSATLHSFDVKKLSERIMHFPTWVDLKGEDSVPETVHHVVVPVNPKSDRIWERLGKNHIRTDEVHAKDNTRPGANSAEMWSEAIKVLKGEYAVRAIKEHKMDQAIIFCRTKIDCDNMEQYFIQQGGGPDSKGHQFSCVCLHGDRKPNERKTNLERFKRKEVRLLVCTDVAARGIDIHGVPYVINVTLPDEKQNYVHRIGRVGRAERMGLAISLVAMEKEKVWYHVCPNRGRGCYNTRLKEDGGCTIWYNEKELLSEVEEHLKCTITQCEPDIKVPVDDFDGKVTYGQRRALGGGNYKGHVDALAPTVQELANLEREAQSSFLQLGFLQNQLFRAF>isotig03108_ORF|dead|dead (asp-glu-ala-asp) box polypeptide 1MKSMLKKTNLERFKRKEVRLLVCTDVAARGIDIHGVPYVINVTLPDEKQNYVHRIGRVGRAERMGLAISLVAMEKEKVWYHVCPNRGRGCYNTRLKEDGGCTIWYNEKELLSEVEEHLKCTITQCEPDIKVPVDDFDGKVTYGQRRALGGGNYKGHVDALAPTVQELANLEREAQSSFLQLGFLQNQLFRAF>isotig01189_ORF|dihydrolipoamide|dihydrolipoamide dehydrogenaseMQSWTQLYRSLATRSHHLPCKLHGAAALSVRTYADKAAIDADVTVVGSGPGGYVAAIKAAQLGFKTVCVEKNATLGGTCLNVGCIPSKALLNNSYLYHMAHGKDFESRGIEISGLTLNLEKMMAQKSGAVKALTGGIAHLFKQNKVTHVNGFGRVSGKNQVTAVTADGSEQVINTKNILIATGSEVTPFPGIPVDEESIVSSTGALSLNKVPGEMIVIGAGVIGVELGSVWQRLGSKVTAVEFLGHVGGLGIDMEMAKNFQRILQKQGLKFKLGTKVMGAIKRPDGKIDVAVEAAAGGKNETLTCDVLLVCIGRRPFTQNLGLDSVGIELDNRGXIPVNNRFQTKVPSIYAIGDVVAGPMLAXKAEDEGIICVEGMAGGAVHIDYNCVPSVIYTHPEVAWVGKSEEQLKEEGVPYRVGKFPFAANSRAKTNNDTDGMVKILSHKETDRMLGAHIVGTGAGEMINEAALAMEYGASCEDIARVCHAHPTVSEAFREANLAASFGKAINF>isotig01190_ORF|dihydrolipoamide|dihydrolipoamide dehydrogenaseMQSWTQLYRSLATRSHHLPCKLHGAAALSVRTYADKAAIDADVTVVGSGPGGYVAAIKAAQLGFKTVCVEKNATLGGTCLNVGCIPSKALLNNSYLYHMAHGKDFESRGIEISGLTLNLEKMMAQKSGAVKALTGGIAHLFKQNKVTHVNGFGRVSGKNQVTAVTADGSEQVINTKNILIATGSEVTPFPGIPVDEESIVSSTGALSLNKVPGEMIVIGAGVIGVELGSVWQRLGSKVTAVEFLGHVGGLGIDMEMAKNFQRILQKQGLKFKLGTKVMAVEAVAVENETLTCDVLLVCIGRRPFTQNLGLDSVGIELDNRGXIPVNNRFQTKVPSIYAIGDVVAGPMLAXKAEDEGIICVEGMAGGAVHIDYNCVPSVIYTHPEVAWVGKSEEQLKEEGVPYRVGKFPFAANSRAKTNNDTDGMVKILSHKETDRMLGAHIVGTGAGEMINEAALAMEYGASCEDIARVCHAHPTVSEAFREANLAASFGKAINF>isotig04302_ORF|dip13|dip13 betaETMRMAQRELLSVEDTVYMPDKDTEPVNRTLIQKAGYLNIRNKTGLVTTAWDRLYFFTQGGNLMCQPRGAVAGGMVLDLDNSSVMAVECEDRRYCFQITSPSGKTSMILQAESKREYEEWICTVNNISRQIYLTDNPEAVAIRLNQTAIQAVTPITSFEKRPE>isotig04303_ORF|dip13|dip13 betaETMRMAQRELLSVEDTVYMPDKDTEPVNRTLIQKAGYLNIRNKTGLVTTAWDRLYFFTQGGNLMCQPRGAVAGDMAVECEDRRYCFQITSPSGKTSMILQAESKREYEEWICTVNNISRQIYLTDNPEAVAIRLNQTAIQAVTPITSFEKRPE>isotig01278_ORF|elongation|elongation factor 1-deltaMSGLQCLASENIWFDKQRYDEAEKRFYEGVNGPSTQVKTALQQAKGRPQKRQHRNSSSHAGDQELVSRMKSLELENQTLYKVVETMRAALQKLETRVAVLEKTPATAAVPCAKAAPVKQVENGDDDDDDDDIDLFGSDDDDEEAARIKQERVDAYAAKKSKKPTLIAKSSILLDVKPWDDETDMAQLEQCVRSVQMDGLLWGASKLVPVGYGIKKLQINCVVEDDKVGTDILEEEITKFEDFVQSVDVAAFNKI>isotig01279_ORF|elongation|elongation factor 1-deltaMSGLQCLASENIWFDKQRYDEAEKRFYEGVNGPSTQSSSHAGDQELVSRMKSLELENQTLYKVVETMRAALQKLETRVAVLEKTPATAAVPCAKAAPVKQVENGDDDDDDDDIDLFGSDDDDEEAARIKQERVDAYAAKKSKKPTLIAKSSILLDVKPWDDETDMAQLEQCVRSVQMDGLLWGASKLVPVGYGIKKLQINCVVEDDKVGTDILEEEITKFEDFVQSVDVAAFNKI>isotig03423_ORF|elongation|elongation factor 1-deltaFLPVVTHCRRSTRFIRRPGLIPTRQKTQHSTMSSVDFLAQEKIWFDKPRYDEAERRFYERINGSSEPTKDVGANSILQDIARARENIQKSLAGLKTTLCNRGSGEPALSQQSQISSSSVADQGELVSRIKSLELENQSLHKVVDDLRAALSKLECRVAVLEKSPAAVTPTPTPSVPYTNGTTVQQKSSVPVKEEEEEEDDDDDDIDLFGSDEDEEADKLKEQRLKEYAEKKAKKPGLIAKSSILLDVKPWDDETDMAKLEECVRSVQADGLLWGTSKLVPVGYGIKKLQIACVVEDDKVGTDMLEEEITKFEDYVQSVDVAAFNKI>isotig03424_ORF|elongation|elongation factor 1-deltaFLPVVTHCRRSTRFIRRPGLIPTRQKTQHSTMSSVDFLAQEKIWFDKPRYDEAERRFYERINGSSEPTKDVGANSILQDIARARENIQKSLAGSSSSVADQGELVSRIKSLELENQSLHKVVDDLRAALSKLECRVAVLEKSPAAVTPTPTPSVPYTNGTTVQQKSSVPVKEEEEEEDDDDDDIDLFGSDEDEEADKLKEQRLKEYAEKKAKKPGLIAKSSILLDVKPWDDETDMAKLEECVRSVQADGLLWGTSKLVPVGYGIKKLQIACVVEDDKVGTDMLEEEITKFEDYVQSVDVAAFNKI>isotig03439_ORF|hepatoma-derived|hepatoma-derived growth factorMPRSNRQKEYKPGDLVFAKMKGYPHWPARIDELPEGAVKSPSNKYQVFFFGTHETAFLGPKDLFPYDEHKEKFGKPNKRKGFAEGLWEIENNPTVTHEGYESSKKDNASEGAGDTGGSEKADAEGSSDEDEGALVIDEKNERGGTKRKAEESTEASPKRPKDTEAEGDSKVDGDKSNAEAKLNDVAGPKPTAPSSQSESKAEAQENAPAGGQLTADKPVTDSA>isotig03440_ORF|hepatoma-derived|hepatoma-derived growth factorMPRSNRQKEYKPGDLVFAKMKGYPHWPARIDELPEGAVKSPSNKYQVFFFGTHETAFLGPKDLFPYDEHKEKFGKPNKRKGFAEGLWEIENNPTVTHEGYESSKKDNASEGAKLNDVAGPKPTAPSSQSESKAEAQENAPAGGQLTAD>isotig03895_ORF|protein|ik cytokineMPETESYSNPLAPDGHEVEDHRAAAQSKLTNDDFRKLLMTPRATPSSAPPSKSRHHEMPRDYNEDEDPAARRRKKKSYYAKLRQQEMERERELAEKYRDRARERRDGVNKDYEETELISTTANYRAVGPTAEADKSAAEKRRQLIQESKFLGGDMEHTHLVKGLDFALLQKVRAEITSKEKERKRIWWRKSRKRQRK>isotig03896_ORF|protein|protein redMPRDYNEDEDPAARRRKKKSYYAKLRQQEMERERELAEKYRDRARERRDGVNKDYEETELISTTANYRAVGPTAEADKSAAEKRRQLIQESKFLGGDMEHTHLVKGLDFALLQKVRAEITSKEKERKRIWWRKSRKRQRK>isotig01879_ORF|leucine|leucine rich repeat containing 20MAEAVANVARRINATVEDGKDSLDLSNCKLISFPDGVFKVLRSVSDNIRIVTLADNEMKALSSKFFSTFTQLRELDLRGNVLTKLPDTVAEMEHLTCINLANNSFSIFPDKLTEIATLERINLEGNSITDIPVEKLSAMPALKWINVKSNPLDSNTLSALQSPHNFDIVSGADS>isotig01880_ORF|leucine|leucine rich repeat containing 20MTSDTARNSDHRRMAEAVANVARRINATVEDGKDSLDLSNCKLISFPDGVFKVLRSVSDNIRIVTLADNEMKALSSKFFSTFTQLRELDLRGNVLTKLPDTVAEMEHLTCINLANNSFSIFPDKLTEIATLERINLEGNSITDIPVEKLSAMPALKWINVKSNPLDSNTLSALQSPHNFDIVSGADS>isotig03006_ORF|microtubule-associated|microtubule-associated protein rp eb family member 1MAVNVYSTSMTIENLSRHDMLAWVNDSLQLTYTKIEQLGSGAAYCQFMDMLFPGCILLKKVKFSAKLEHEYIHNFKVLQASFKRMNVDKIIPVERLVKGKFQDNFEFLQWFKKFFDANYDGKEYDPLLTRQGQDVTPPPPTPGPMRTSPTVPKTVPTPQRQIAPARRSAPMTRNGGDAELIELNQQLLDLKLTVEGLEKERDFYFTKLRDIELICQENDNDNNPVLTKIMDTLYATEEGFAPPDDDEIDEVAQGDQDEF>isotig03005_ORF|microtubule-associated|microtubule-associated rp eb member 3MAVNVYSTSMTIENLSRHDMLAWVNDSLQLTYTKIEQLGSGAAYCQFMDMLFPGCILLKKVKFSAKLEHEYIHNFKVLQASFKRMNVDKIIPVERLVKGKFQDNFEFLQWFKKFFDANYDGKEYDPLLTRQGQDVTPPPPTPGETILHKPKRPSRSGPMRTSPTVPKTVPTPQRQIAPARRSAPMTRNGGDAELIELNQQLLDLKLTVEGLEKERDFYFTKLRDIELICQENDNDNNPVLTKIMDTLYATEEGFAPPDDDEIDEVAQGDQDEF>isotig03833_ORF|n-myc|n-myc downstream regulated gene 1MDDIQVVSKPLLVDRELPGLREAVQQLVIKEHDVETPHGRIHCTMKGVPKADRPCILTMHDIGLNHKTCWDMLFNHEDMAEIMQHLRCVTSTPPGQHEGANTFSTGYEYPSMDQLSETLPLVLKHFGLKSVIGMGIGAGSYILTRFALDYPNMMEGLMLLNINPCAEGWMDWAAHKISGWTHAMPDMIISHLFGKEEIHHNQDLI>isotig03834_ORF|n-myc|n-myc downstream regulated gene 1MDQLSETLPLVLKHFGLKSVIGMGIGAGSYILTRFALDYPNMMEGLMLLNINPCAEGWMDWAAHKISGWTHAMPDMIISHLFGKEEIHHNQDLI>isotig03559_ORF|nfkb|nfkb activatingEEDPSSVMWVEKKNKKHSEDSDLESDSDEEEIKKKKKKKKSKKSKKSKKKKAKKTRKESSNSSNSEESEEEEEEEDPSSVMWVEKTCMDEHVVGPEAPLTHMSQDDRPLDFGHALLPGEGAAMAEYVKAGKRIPRRGEIGLTSDEIANFEKSGYVMSGSRHRRMEAVRLRKENQIYSADEKRALASFNQEERRKRESKILSSFREMVYRKTKGKEEK>isotig03560_ORF|nfkb|nfkb activatingEEDPSSVMWVEKKNKKHSEDSDLESDSDEEEIKKKKKKKKSKKSKKSKKKKAKKTRKESSNSSNSEESEEEEEEEDPSSVKAGKRIPRRGEIGLTSDEIANFEKSGYVMSGSRHRRMEAVRLRKENQIYSADEKRALASFNQEERRKRESKILSSFREMVYRKTKGKEEK>isotig02789_ORF|nucleotide|nucleotide bindingMAQLTYSRLSHLLRLSVNKPSALRTRTEVNLGPACCLQFVRCQRSMDSKALQERQKQHMAKGLPKQKPITGVKQVIVVASGKGGVGKSTTAVNLADVYGPSIPKLMNLKGNPELSDNNLMIPLTNYGIPCMSMGFLVDDVAPIVWRGLMVMSAIEKLLRQVDWGSLDYLVVDMPPGTGDVQLSISQNIPVAGAVIVSTPQDIALLDARRGAEMFKKVHVPVLGLVQNMSVFQCPNCNHQTHIFGSDGARQLADTLGVQFLGDVPLHLNIREMSDRGKPVVVSSPDSPEAEAYRKVASAVVRRLEEVNT>isotig02790_ORF|nucleotide|nucleotide bindingMAQLTYSRLSHLLRLSVNKPSALRTRTEVNLGPACCLQFVRCQRSMDSKALQERQKQHMAKGLPKQKPITGVKQVIVVASGKGGVGKSTTAVNLADVYGPSIPKLMNLKGNPELSDNNLMIPLTNYGIPCMSMGFLVDDVAPIVWRGLMVMSAIEMFKKGPDSSQTLWESSS>isotig01863_ORF|o-phosphoseryl-trna|o-phosphoseryl-trna selenium transferaseMHLIQQGARVGRIDAFVQSLDKNFMVPVGGAIIAGFDEPFIQEISKMYPGRASASPSLDVLITLLTLGASGYKKLLSERKEIYSFLAQELKSLASAHGERLLHTPHNPISLAMSLDGLQAESDKAVTHLGSMLFTRQVSGARVIPLGKEQTISGHTFRGFMSHSEEYPCPYLNAASAVGITRDDVTLCIKRLDKCLKTLKKDGNAAKSSPPEYPSCQEDTTGE>isotig01864_ORF|o-phosphoseryl-trna|o-phosphoseryl-trna selenium transferaseMFLRAMSCGRTWRQLSARSRSSEPITSCVFIQQRPALPRGSPTGLRSWPLCVPNITLPHIVNNAYGVQSSKCMHLIQQGARVGRIDAFVQSLDKNFMVPVGGAIIAGFDEPFIQEISKMYPGRASASPSLDVLITLLTLGASGYKKLLSERKEIYSFLAQELKSLASAHGERLLHTPHNPISLAMSLDGLQAESDKAVTHLGSMLFTRQVSGARVIPLGKEQTISGHTFRGFMSHSEEYPCPYLNAASAVGITRDDVTLCIKRLDKCLKTLKKDGNAAKSSPPEYPSCQEDTTGE>isotig02685_ORF|osteoblast|osteoblast specific factorGVFINGGLEGGVTNLLKTLQGNNLQVLSVNNSINVNSVDVPDSDLMATNGVIHVVKNVLYPADLPVGRQDLLVLLKKLIRYIQIKFVSGFTYTEIPLTFLKRVITTTHYIETGPELKVTRRIETGEPTITKVTRVIETEPIVTEVVIKGEPTITKVTRVVENDPSFTKVTRVIGGDASVTTRVGTGDPSITKVTRVIDGDDIQTTRVSTGDPLLTKVTRVIKGDASITTRVGTGDSSLTKVTRVNAGQPSITKVTRVIEGGVDAEGKPIAGGAGYTTGNEPYIIEGPDFSKITTIHGNPNLIDEESERITKIIKEGGKFAAARKAPAGMRRRTRLVRRHHKPRE>isotig02686_ORF|osteoblast|osteoblast specific factorGVFINGGLEGGVTNLLKTLQGNNLQVLSVNNSINVNSVDVPDSDLMATNGVIHVVKNVLYPADLPVGRQDLLVLLKKLIRYIQIKFVSGFTYTEIPLTFLKRVITTTHYIETGPELKVTRRIETGEPTITKVTRVIETEPIVTEVVIKGEPTITKVTRVVENDPSFTKVTRVIGGDASVTTRVGTGDPSITKVTRVIDGDDIQTTRVSTGDPLLTKVTRVIKGDASITTRVGTGDSSLTKVTRVNAGQPSITKVTRVIEGGVDAEGKPIAGPDFSKITTIHGNPNLIDEESERITKIIKEGGKFAAARKAPAGMRRRTRLVRRHHKPRE>isotig02999_ORF|poly|poly -binding protein 2MDSGVIEGGLNVTLTIRLLMHGKEVGSIIGKKGESVKKMREESGARINISEGNCPERIITLAGPTTAIFKAFSMIIEKLEEDISSSMTNSTATSKPPVTLRIVVPASQCGSLIGKGGCKIKEIRESTGAQVQVAGDMLPNSTERAITIAGTPQSIIECVKQICVVMLESPPKGVTIPYRPKPSGSPVIFAGGQAYAVQGQHAIPQPDSSSAAISPQLTKLHQLAMQQSPFPIAPSNQGFTGIDASAQTGSHEMTIPNDLIGCIIGRQGAKINEIRQMSGAQIKIANPVDGSTDRQVTITGSPASISLAEYLINARLSSEATGLAAN>isotig03000_ORF|poly|poly -binding protein 2MDSGVIEGGLNVTLTIRLLMHGKEVGSIIGKKGESVKKMREESGARINISEGNCPERIITLAGPTTAIFKAFSMIIEKLEEDISSSMTNSTATSKPPVTLRIVVPASQCGSLIGKGGCKIKEIRESTGAQVQVAGDMLPNSTERAITIAGTPQSIIECVKQICVVMLESPPKGVTIPYRPKPSGSPVIFAGGQAYAVQGQHAIPQPDLTKLHQLAMQQSPFPIAPSNQGFTGIDASAQTGSHEMTIPNDLIGCIIGRQGAKINEIRQMSGAQIKIANPVDGSTDRQVTITGSPASISLAEYLINARLSSEATGLAAN>isotig03889_ORF|probable|probable o-sialoglycoprotein endopeptidaseMTVVIGFEGSANKIGIGIIRDGEVLSNPRRTYITPPGQGFMPSDTARHHRAVVLTVLKEALEQAGLKPADIDCVSYTKGPGMGAPLVTVALVARTVAQLWGKPLLGVNHCIGHIEMGRLITKANNPTVLYVSGGNTQVIAYSERRYRIFGETIDIAVGNCLDRFARVIKISNDPSPGYNIEQMAKTGSQYVELPYTVKGMDVSFSGIL>isotig03890_ORF|probable|probable o-sialoglycoprotein endopeptidaseMTVVIGFEGSANKIGIGIIRDGEVLSNPRRTYITPPGQGHIEMGRLITKANNPTVLYVSGGNTQVIAYSERRYRIFGETIDIAVGNCLDRFARVIKISNDPSPGYNIEQMAKTGSQYVELPYTVKGMDVSFSGIL>isotig03580_ORF|a|a chain crystal structures of human pyruvate dehydrogenase kinase 2 containing physiological and synthetic ligandsTSRISIRMLINQHTLVFNGNINPAHPYTIGCIHSICDVTEVARDAYESAKMLCEQYYLGAPELELREMNANNMKEPIQISYIPSHLYHMLFELFKNAMRATIETHEASRTLPPIKVMIALGGEDLSIKMSDRGGGVPFRKTERLFSYMYSTAPRPSFGDKHRAPLAGFGYGLPISRLYARYFQGDLQLYSMEGTGTDAVIHLKALSTDSVERLPVFNKTALRHYKLSLEADDWCIPSKDPLDLAVYRVAK>isotig03581_ORF|pyruvate|pyruvate dehydrogenase isoenzyme 2MRATIETHEASRTLPPIKVMIALGGEDLSIKMSDRGGGVPFRKTERLFSYMYSTAPRPSFGDKHRAPLAGFGYGLPISRLYARYFQGDLQLYSMEGTGTDAVIHLKALSTDSVERLPVFNKTALRHYKLSLEADDWCIPSKDPLDLAVYRVAK>isotig03389_ORF|ras-related|ras-related gtp binding aMGKSGSGKTSMRSIIFANYIARDTRRLGATIDVEHSHVRFLGNLVLNLWDCGGQDTFMENYFTSQRDNIFRNVEVLIYVFDVESRELEKDMHYYQSCLEAILQNSPDAKVFCLVHKMDLVQEDQRDLIFKEREEDLKRLSRPLACTCFRTSIWDETLYKAWSSIVYQLIPNVQQLETNLRNFAQIIEADEVLLFERATFLVISHYQCKEQRDAHRFEKISNIIKQFKLSCSKLAASFQSMEVRNSNFAAFIDVFTSNTYVMVIMSDPSIPSAATLINIRNARKHFEKLERVMDPSTACTCECASWSRLVPVDTLSQSVHRPQTTSR>isotig03390_ORF|ras-related|ras-related gtp binding aMGKSGSGKTSMRSIIFANYIARDTRRLGATIDVEHSHVRFLGNLVLNLWDCGGQDTFMENYFTSQRDNIFRNVEVQEDQRDLIFKEREEDLKRLSRPLACTCFRTSIWDETLYKAWSSIVYQLIPNVQQLETNLRNFAQIIEADEVLLFERATFLVISHYQCKEQRDAHRFEKISNIIKQFKLSCSKLAASFQSMEVRNSNFAAFIDVFTSNTYVMVIMSDPSIPSAATLINIRNARKHFEKLERVMDPSTACTCECASWSRLVPVDTLSQSVHRPQTTSR>isotig03415_ORF|ribonuclease|ribonuclease h2 subunit bMATKKKRTPNAHTDSWVVIAAESAIDTQKHDIDPAFVRLRNPSTDAASLYMLSSGDVQLFEVKAFEEDFHSWFIGQTVQRDGRLLFVTPMDPLYLILPYLIKSGKEGKFQPVDQVVMDEEFPACSRLLSCTRSLASLHHIAEEKEVGSLKFHRYSQEKTMNWLKKKVERTVVALKKRNISVGEGVKSTTYVRVKSEADCPEEDYLRYAHGLISEYISEDLSKALLKHLQLPELTSPKETEPPSKKRKLSDNPVEAGEDYTKFNSADFVRKPPKKMTAAQKSLAKVDKTGMKTMSSFFSPKAKAEKK>isotig03416_ORF|ribonuclease|ribonuclease h2 subunit bMATKKKRTPNAHTDSWVVIAAESAIDTQKHDIDPAFVRLRNPSTDAASLYMLSSGDVQLFEVKAFEEDFHSWFIGQTVQRDGRLLFVTPMDPLYLILPYLIKSGKEGKFQPVDQVVMDEEFPACSRLLSCTRSLASLHHIAEEKEVGSLKFHRYSQEKTMNWLKKKVERTVVALKKRNISVGEGVKSTTYVRVKSEADCPEEDYLRYAHGLISEYISEDLSKALLKHLQLPELTSPKETEPPSKPPKKMTAAQKSLAKVDKTGMKTMSSFFSPKAKAEKK>isotig02824_ORF|rna|rna terminal phosphate cyclase domain 1QIDYTVKVFKPILEKFGVNFDCDIRMRGYYPKGGGEVVVTVNPVKELQPVTMMERGNITKIYGRAFVAGVLPYKLAKDMSAAAVRTIRKEIKDLYINIQALQEKEKACGSGNGIIIIAESSTGCVFAGSALGKKGVYADKIGIEAAEMLLRNIRHNGCVDEFLQDQLIIFMALAKGRSRIRTGAVTLHTQTAIHIAEQLTQAKFTITKCEDELSSNVTFIIECEGSGASNPHL>isotig02825_ORF|rna|rna terminal phosphate cyclase domain 1QIDYTVKVFKPILEKFGVNFDCDIRMRGYYPKGGGEVVVTVNPVKELQPVTMMERGNITKIYGRAFVAGVLPYKLAKDMSAAAVRTIRKEIKDLYIIFMALAKGRSRIRTGAVTLHTQTAIHIAEQLTQAKFTITKCEDELSSNVTFIIECEGSGASNPHL>isotig02442_ORF|sarcalumenin|sarcalumeninLLAAIMKGIVLVFCFLSLLHLQATAEEEEEAFTSVLRDRSHIDETLRLATEEKAGDYAAALERLRKIYHTSIKPMEQAYKYNELRQHEISAYPGRTLGDSATDGEITSKPMVLFLGPWSVGKSSMINYLLGMQDSPYQLYTGAEPTTSEFTVIMHGEKIRSVEGIVMAADSSRSFSPLEKFGQNFLEKLIGIEMPHKLLERVTFVDTPGIIENRKQQERGYPFNDVCQWFIDRADLIFVVFDPTKLDVGLELEMLFRQLKGRESQIRIILNKADNLATQDLMRVYGALFWSLAPLINVTEPPRVYVSSFWPYDYAPDTSRDLFKREEISLLEDLNQVIENRMENKIAFIRQHGIRVRIHGLLVDRYVQTFKEKMSFFSDPELVFQEIVDDPDKFYIFKSILAKTNVSKFDLPNRDAYRDFFGINPITNFKPLTAQCSYMGGCLLEKIERAITNELPALLSSINSGKQPGLSSCEATGCGEKPKNRYRKN>isotig02444_ORF|sarcalumenin|sarcalumeninLLAAIMKGIVLVFCFLSLLHLQATAEEEEEAFTSVLRDRSHIDETLRLATEEKAGDYAAALERLRKIYHTSIKPMEQAYKYNELRQHEISDGEITSKPMVLFLGPWSVGKSSMINYLLGMQDSPYQLYTGAEPTTSEFTVIMHGEKIRSVEGIVMAADSSRSFSPLEKFGQNFLEKLIGIEMPHKLLERVTFVDTPGIIENRKQQERGYPFNDVCQWFIDRADLIFVVFDPTKLDVGLELEMLFRQLKGRESQIRIILNKADNLATQDLMRVYGALFWSLAPLINVTEPPRVYVSSFWPYDYAPDTSRDLFKREEISLLEDLNQVIENRMENKIAFIRQHGIRVRIHGLLVDRYVQTFKEKMSFFSDPELVFQEIVDDPDKFYIFKSILAKTNVSKFDLPNRDAYRDFFGINPITNFKPLTAQCSYMGGCLLEKIERAITNELPALLSSINSGKQPGLSSCEATGCGEKPKNRYRKN>isotig03331_ORF|paraoxonase|serum paraoxonase arylesterase 2MGKLVCLSIVVAALAALFGERVVNLRKRTLATRELVQNHLPNCVELKNLDYGSEDITILGNGLAFISTGLKYPGLPSQDVTAKLFVVDMKDSRKKPVELRMPRNFDLESFNPHGISLFTDPSDDAVYLFVVNHPQHKSQVELFKFAEEDFSLLHLKTITHELLHSVNDIVAVGVDAFYATNDHYFSHHLLKAFVETFLVQPWCNVVYYSPEGVKVVSEGYYFPNGINLSPDKKHVYVVDLFDHNVHVLERKDDNGLVSVKSMHVGSLCDNVEVDPETGDLWLGCHPNAWKAFMLDPNDPPGSEVIRIQNIHSDKPQ>isotig03332_ORF|paraoxonase|serum paraoxonase arylesterase 2MRELVQNHLPNCVELKNLDYGSEDITILGNGLAFISTGLKYPGLPSQDVTAKLFVVDMKDSRKKPVELRMPRNFDLESFNPHGISLFTDPSDDAVYLFVVNHPQHKSQVELFKFAEEDFSLLHLKTITHELLHSVNDIVAVGVDAFYATNDHYFSHHLLKAFVETFLVQPWCNVVYYSPEGVKVVSEGYYFPNGINLSPDKKHVYVVDLFDHNVHVLERKDDNGLVSVKSMHVGSLCDNVEVDPETGDLWLGCHPNAWKAFMLDPNDPPGSEVIRIQNIHSDKPQ>isotig04130_ORF|suppressor|suppressor of swi4 1 homologMDVRRVMEPFTAESLKIKKKNVLKDFVTIAGPLGVTHFIIFSKTPTSVNMRLARLPKGPTLHFKVLKYSLIKDVVSTLKKHRMHEQQFTHHPLLILNNFSSEGMHVKLMASMFQNMFPSINVHKVNLNNIKRCVLINYNAESGEIEFRHYSLKVVPVGMSRGVKKLMQERFPNMNKLEDISELLMKGANLSESEAEQDGEH>isotig04131_ORF|suppressor|suppressor of swi4 1 homologMDVRRVMEPFTAESLKIKKKNVLKDFVTIAGPLGVTHFIIFSKTPTSVNMRLARLPKGPTLHFKVLKYSLIKDVVSMFQNMFPSINVHKVNLNNIKRCVLINYNAESGEIEFRHYSLKVVPVGMSRGVKKLMQERFPNMNKLEDISELLMKGANLSESEAEQDGEH>isotig03087_ORF|synaptic|synaptic 2MDVLALEAKSVNGAEADKKSAPRPKPKPPKKAKRIVYFEVEIVDLKTKEKLLLLDKVEPTATVLDIKALFQKSYPKWYPARQSLRLDPKAKCLKDEEVLQTLPVGTTASFYFSDLGPQLTWGTVFLAECAGPLIIYLMFYFRLPFIYSPKYDFTTSKHWVVHLACMCHSFHYIKRILETMFVHRISHGTMPLRNIFKNCGYYWCTAAWMAYYINHPLYTTPYYGQQQVNSGLYVFLFCQVGNFSIHVALRNLKLPGSKAKKIPYPTKNPFTWIFWLVSCPNYTYELGSWMGFTVMTQCVPVAFFTVVAFVQMTVWAKGKHRSYLKEFRDYPTLRSSILPFIL>isotig03088_ORF|synaptic|synaptic 2MCHSFHYIKRILETMFVHRISHGTMPLRNIFKNCGYYWCTAAWMAYYINHPLYTTPYYGQQQVNSGLYVFLFCQVGNFSIHVALRNLKLPGSKAKKIPYPTKNPFTWIFWLVSCPNYTYELGSWMGFTVMTQCVPVAFFTVVAFVQMTVWAKGKHRSYLKEFRDYPTLRSSILPFIL>isotig01938_ORF|transitional|transitional endoplasmic reticulum atpaseMPGSGGADPKGEDFSTAILKQKQRPNRLIVDEALNEDSSIVSLSQNKTEELQLFRGDTVVLRGRKRRQTVCIVLTDDTCGEERIRMNRVTRNNLRVRLGDVISIHACPDIKYGKKIHVLPIDDTIEGLTGNLFEVFLKPYFLEAYRPIHTDDIFLVRGSMRAVEFKVMDTDPSPHCIVAPDTVIYCEGEPIKREDEEESLNDIGYDDIGGCRKQLAQIKEMVELPLRHPGLFKAIGVKPPRGILLYGPAGTGKTLVARAVANETGAFFFLINGPEIMSKLAGESESNLRKAFEEAEKNAPAIIFIDELDAIAPKREKTHGEVERRIVSQLLTLMDGLKQRAHVVVMAATNRPNSVDSALRRFGRFDREIDIGIPDTTGRLEILQIHTKNMKLAGDVDLERIAAETHGHVGADLAALCSEAALQAIRKKMTLIDLEDESIDADLLNSLAVTMDDFQWALSQSNPSALRETCAEVPQVSWQDIGGLDEVKRELQELVQYPVEYPDKFLKFGMTPSRGVLFYGPPGCGKTLLAKAIANECQANFVSIKGPEMLTMWFGESEANVRDVFDKARQAAPCILFFDELDSIAKSRGGGAGDAGGAADRVINQILTEMDGMSDKKNVFIIGATNRPDIIDAAILRPGRLDQLIYIPLPDKPSRRAILNANLRKSPVARDVDLEYLSGITEGFSGADLTEICQRACKLAIREAIEAEIKAERQRQNRPGIPMDEDFDPVPEIRKDHFEEAMRFARRSVSDNDIRKYEMFAQTLQQSRGFGSFRFPSDTGTRSGGQGSGSGSGRPGLYRDEGNDDLYQ>isotig01940_ORF|transitional|transitional endoplasmic reticulum atpaseMKLAGDVDLERIAAETHGHVGADLAALCSEAALQAIRKKMTLIDLEDESIDADLLNSLAVTMDDFQWALSQSNPSALRETCAEVPQVSWQDIGGLDEVKRELQELVQYPVEYPDKFLKFGMTPSRGVLFYGPPGCGKTLLAKAIANECQANFVSIKGPEMLTMWFGESEANVRDVFDKARQAAPCILFFDELDSIAKSRGGGAGDAGGAADRVINQILTEMDGMSDKKNVFIIGATNRPDIIDAAILRPGRLDQLIYIPLPDKPSRRAILNANLRKSPVARDVDLEYLSGITEGFSGADLTEICQRACKLAIREAIEAEIKAERQRQNRPGIPMDEDFDPVPEIRKDHFEEAMRFARRSVSDNDIRKYEMFAQTLQQSRGFGSFRFPSDTGTRSGGQGSGSGSGRPGLYRDEGNDDLYQ>isotig01941_ORF|transitional|transitional endoplasmic reticulum atpaseMTPSRGVLFYGPPGCGKTLLAKAIANECQANFVSIKGPEMLTMWFGESEANVRDVFDKARQAAPCILFFDELDSIAKSRGGGAGDAGGAADRVINQILTEMDGMSDKKNVFIIGATNRPDIIDAAILRPGRLDQLIYIPLPDKPSRRAILNANLRKSPVARDVDLEYLSGITEGFSGADLTEICQRACKLAIREAIEAEIKAERQRQNRPGIPMDEDFDPVPEIRKDHFEEAMRFARRSVSDNDIRKYEMFAQTLQQSRGFGSFRFPSDTGTRSGGQGSGSGSGRPGLYRDEGNDDLYQ>isotig01735_ORF|upstream|upstream-binding protein 1 isoform aMSVGIVEPKTHPSQLNAAEFLWDMNKRTSVFVQVHCISTEFTPRKHGGEKGVPFRIQFDTFAQGESGEYTEHLHSASCQIKVFKPKGADRKQKTDREKMEKRTP>isotig01736_ORF|upstream|upstream-binding protein 1 isoform aMLFWQPYTENFRAPVQRQGGGGLTRDVLALPIFKQEDSSIPPENETKNPPFQYVLCTATSPAVKLHDETLTYLNQGQSYEVRMLDNRKPGELPELNNKMVKSIVRVVFHDRRLQYTEHQQLEGWKWNRPGDRLLDIDIPMSVGIVEPKTHPSQLNAAEFLWDMNKRTSVFVQVHCISTEFTPRKHGGEKGVPFRIQFDTFAQGESGEYTEHLHSASCQIKVFKPKGADRKQKTDREKMEKRTP>isotig01737_ORF|upstream|upstream-binding protein 1 isoform aMLDNRKPGELPELNNKMVKSIVRVVFHDRRLQYTEHQQLEGWKWNRPGDRLLDIDIPMSVGIVEPKTHPSQLNAAEFLWDMNKRTSVFVQVHCISTEFTPRKHGGEKGVPFRIQFDTFAQGESGEYTEHLHSASCQIKVFKPKGADRKQKTDREKMEKRTP>isotig02377_ORF|voltage-dependent|voltage-dependent anion channel 3MEEKGVGSVVQQDKTGKVKQAENKDQCVACHHHAPKGHVTMAVPPAYADLGKSAKDIFSKGFGYGTLKLDVKTKSQSGVMEFNTSGSNNTDTGKSGGHLETKYKVKDLGLNFSQKWNTDNTLTTEITMEDQLAKGLKLSLDTSFVPNTGKKSAKLKTGYKRDYANVGYEGWLAGYQLAFDTAKSKLTKNNFALGYKAGDFQLHTSVNDGTEFGGSIYQKVNSNLETAVQLAWTAGSNNTRFGIGAKYQLDKDASLSTKVDNACLVGVGYTQTLRPGVKLTLSGLIDGKNVNGGGHKIGLGFELEA>isotig02376_ORF|voltage-dependent|voltage-dependent anion-selective channel protein 2MEEKGVGSVVQQDKTGKVKQAENKDQCVACHHHAPKGHVTMAVPPAYADLGKSAKDIFSKGFGYGTLKLDVKTKSQSGVMEFNTSGSNNTDTGKSGGHLETKYKVKDLGLNFSQKWNTDNTLTTEITMEDQLAKGLKLSLDTSFVPNTGKKSAKLKTGYKRDYANVGCDLDFDMAGPTVHAAAVLGYEGWLAGYQLAFDTAKSKLTKNNFALGYKAGDFQLHTSVNDGTEFGGSIYQKVNSNLETAVQLAWTAGSNNTRFGIGAKYQLDKDASLSTKVDNACLVGVGYTQTLRPGVKLTLSGLIDGKNVNGGGHKIGLGFELEA>isotig04084_ORF|zinc|zfx proteinHALSSKAPFECEMCGKEFHQQAALFSHRLQHHHREPKNQPPPPPTKMHKCKFCDYETAEQGLLNRHLLAVHSKSFPHICVECGKGFRHPSELIQHGLTHEENKTHHCAHCDHKSSNSSDLKRHIISVHTKDYPHKCAICGKGFHRPSELKKHSVSHRTKKLHQ>isotig04083_ORF|zinc|zinc finger x-chromosomal proteinHALSSKAPFECEMCGKEFHQQAALFSHRLQHHHREPKNQPPPPPTKMHKCKFCDYETAEQGLLNRHLLAVHSKSFPHICVECGKGFRHPSELKKHMRTHTGEKPYSCLYCDYKSADSSNLKTHIKTKHSKEMPYKCERCFQTFAEEEELIQHGLTHEENKTHHCAHCDHKSSNSSDLKRHIISVHTKDYPHKCAICGKGFHRPSELKKHSVSHRTKKLHQ
