# Supplementary material for: Fast skeletal muscle transcriptome of the Gilthead sea bream (Sparus aurata) determined by next generation sequencing
Source: BMC Genomics. 2012 May 11;13:181. doi: 10.1186/1471-2164-13-181 (PMC3418159; doi:10.1186/1471-2164-13-181)
Supplement: Additional file 12 — Gilthead sea bream paralogues sequences and their alignments. Alignments have been done using ClustalW. [file 1471-2164-13-181-S12.rtf]

>adp_atp_translocasa_Sparus_solute_carrier_25_member_6MSETAISFAKDFLAGGIAAAISKTAVAPIERVKLLLQVQHASKQISVDKQYKGIIDCVVRIPKEQGFLSFWRGNLANVIRYFPTQALNFAFKDKYKKIFLDGVDKRKQFWRYFAGNLASGGAAGATSLCFVYPLDFARTRLAADVGKAGAGREFKGLGDCLVKISKSDGIKGLYQGFSVSVQGIIIYRAAYFGVYDTAKGMLPDPKNTHIFVSWMIAQSVTAVAGLISYPFDTVRRRMMMQSGRKGADIMYSGTIDCWRKIVRDEGSKAFFKGAWSNVLRGMGGAFVLVLYDELKKVI>adp_atp_translocasa_Sparus2_solute_carrier_25_member_5AFRRCGLTAVEGSGVYLEYWLFERRTKKIGTMSETAISFAKDFLAGGISAAVSKTAVAPIERVKLLLQVQHASKQITADKHYKGIMDCITRIPKEQGFVSFWRGNLANVIRYFPTQALNFAFKDKYKKIFLDGVDKRTQFWRYFAGNLASGGAAGATSLCFVYPLDFARTRLAADVGKAGAAREFNGLGDCLAKIFKSDGLKGLYQGFNVSVQGIIIYRAAYFGIYDTAKGMLPDPKNTHILVSWMIAQSVTAVAGLTSYPFDTVRRRMMMQSGRKGADIMYTGTIDCWRKIARDEGGKAFFKGAWSNVLRGMGGAFVLVLYDELKKVM>glioblastome_sparus1_Nipsnap2aAFRGKMATRVLQRVGKGLKQTKSGLQTSGRVMVVIRSGSGFREDSWFKSLFVRKVDPRKDAHSHLLAKKEDSNLYKIQFHNVKPECLDAYNELCEDVLPSIHADPEYPCELVGTWNTWYGEQDQAVHLWRYRGGYPALTEVMNKLRQNKKFMDYRNERGKMLLSRRNQLLLEFSFWNEPVPRPGPNIYELRSYQLRPGTMIEWGNYWARAIEIRQQNQEAVGGFFSQIGSLYTVHHLWAYKDLQSRENIRNAAWQRDGWDEVVYYTVPLIQHMESRIMIPMKTSPLK>glioblastome_sparus2_Nipsnap2bVALCALAQSKMATGVLHRITGSLGRAKSTAQSAGQLVVLTRGLATSSSRNREDSWFKSLFVRKVDPRKDAHSNLLTKNEESNLYKIQFHNVKPECLDAYNKLCEDVLPSIHADKYYPCELVGTWNTWYGEQDQAVHLWRYRGGYPALTEVMNKLKQNQDFTAYRKERGKMLMSRRNQLLLEFSFWNEPVPREGPNIYELRSYQLRPGTMIEWGNYWARAIGYRQHNREAVGGFFSQIGDLYMVHHLWAYKDLQSREDTRNGAWQQEGWDEVVYYTVPLIQHMDSRIMIPTKASPLQ>Tyrosine_sparus1_betaMTEPPQKELVQKAKLAEQAERYDDMAAAMMSVTKDSEELTNEERNLLSVAYKNVVGARRSSWRVVSSIEQKAAEGSEKKKEMAKEYREKIEGELEKICREVLGLLDDYLIPKAPAAESRVFYLKMKGDYYRYLAEVATGDKKTEIITDSKEAYQGAFEISKKEMQPTHPIRLGLALNFSVFFYEILNSPEEACQLAKNAFDDAIAELDTLSEDSYKDSTLIMQLLRDNLTLWTSDNQVEGEDTEESRD>Tyrosine_sparus2_gammaEAHAHVFKMVDREQLVQKARLAEQAERYDDMAAAMKSVTELNEALSNEERNLLSVAYKNVVGARRSSWRVISSIEQKTSADGNEKKIEMVRAYREKIEKELEAVCQDVLNLLDNFLIKNCNETQHESKVFYLKMKGDYYRYLAEVATGEKRATVVESSEKSYSEAHEISKEHMQPTHPIRLGLALNYSVFYYEIQNAPEQACHLAKTAFDDAIAELDTLNEDSYKDSTLIMQLLRDNLTLWTSDQQDDEGGEGNN>calpain_small_subunit1a_sparus1MFFAKKFIGGIIDVVSNIDPAQFVPSEPPPPRRPAVYAEQHESDEEKQFRRVFQQLAGDDMEVSPSELMNILNRIIGKHGDLKTDGFSIESCRSMVAVMDSDSTGKLGFHEFKHLWNNIKKWQGVYKAHDRDGSGVIGADELPEAFRAAGFPLNDQLFQMIIRRYSDENGNMDFDNYIGCLVRLDAMCRSFKTLDKDNNGTIKVNVQEWLQLTMYS>calpain_small_subunit1b_sparus2GSTGFLDSGCGSASCLICLSLSLSVSVSLALSPAASSQSSAAEVTTAANMFMAKAFIKGLINVVSDIDPSQFRPSDPPPPRRPLNFAETHESDEEQKFRRVFKQLAGDDMEVSPKELMDILNKIVSKHGGLKTDGFSIESCRSMVAVMDSDSTGKLGFHEFKYLWNNIKRWQGIYMSHDADGSGVICDQELPKAFKAAGFPLNDQLFKLIIRRYSDEHGNMDFDNFVGCLVRLDAMCRAFKTLDKDNSGTIDLDIKEWLQLTMYS>High_mobility_group_Sparus1_HMG1aMGREVGKPRGKMSSYAYFVQTCREEHKKKHPEASVNFAEFSKKCSERWKTMSPKEKGKFEDLAKQDKVRYEREMMSYVPARGGKKKKFKDPNAPKRPPSAFFIFCSEFRPKVKGEAPGLSIGEVAKRLGEMWNGTASEDKQPFEKKAAKLKEKYEKEVAAYRQKTKGGAAPAGKAPAKVEKKADDDDDDDDDDDEEEDDYDDDDDE>High_mobility_group_Sparus2_HMG2SLTSCVAENRSIATMRKDPKKPRGKMTSYAYFVATCREEHKKKHPGAAVAFTEFSKKCSERWKTMSPKEKVKFEDMAKNDKIRYDQEMKSYVPPKGEKSKKKKKDPNAPKRPPSAFFVFCSDHRPRIKEENPGISIGDIAKKLGELWSTQSPKDKAPYSAKAAKLKEKYDKDVAVYRAKCGSGKSDAGKKSGPGRPSAKKAEPVDDDDDDDDDDDEEEDDEDDDDDEDDD>High_mobility_group_Sparus3_HMG1bMGKDPKKPRGKMSSYAYFVQTCREEHKKKHPEASVNFAEFSKKCSERWKTMSPKEKGKFEDMAKLDKVRYEKEMKNYIPPKGHKKKRFKDPNAPKRPPSAFFLFCADFRPKVKGDYPGLTIGDTAKKLGEMWNSSSAEDKQPYEKKAAKLKEKYDKDIVAYRTKGKVDSESAATADDDDEEEDEEEEGEDEEDDDDEDDE>Carnitine_sparus1_a1MLAVFVRSTLRPGMVNPCRLVRPVTQIPERSLVHQEGLPKLPVPPLKQTCERYLAALEPIVSDEELKHTRELVEEFLKGGVGERLQKGLERRARKTDNWLSEWWMQSAYLDCRMPVPVYTSPGVVLPRMHFTDRQGQMRFAAKLIAGVLDFKKMIDTETLPVEYLSGKPLCMDQYYQILSSCRIPGPKRDTVVNHAIGKAPPTHIAVVHNFQFFVLDVYNSDGSRLTVDQLYMQLEKIWNSSLQTNKEPVGILTSQHRNTWGKAYNNLIKDKTNKESVRAIQKSIFTVCLDAPMPRVSDEMYPSRVAAQMLHGGGARWNSGNRWFDKTLQFIVGEDGTCGLVYEHAPAEGPPIVFLIDYVVKYMQRTETVRSPMVPLSMPQKLRFNITPEVKRDIERAKQNMNMMVQDLDVKVLIFSQFGKKVPKQHKLSPDAFMQMVLQLAYFRIYNISCSMYESASLRMFTYGRTDAIRSTTVDSFNFVQAMQDPAKQNSERLALLQKAVQTHKTNTYDAIHGQAIDRHLLGLKLLSIEDLTSMPEIFMDTSFAVAHHYHLSTSQVGSKTDCVMCFGPMVPDGYGVCYNPMDEHINIAITAFNSCEETNAAKFAQAVEDALLDMRALLEDTATAEQ>Carnitine_sparus2_a2DNTNKESVSAIQRSIFTLCLDGAIPRGSEETYRSCAAIQMLHGGGSQWNSGNRWFDKTLQFIVGEDGTCGANYEHAPAEGPPIVALIDHVVEYTRKPEMVRSPMVPLPMPQKLHFTITPEIKKDIEEAKHNMNTLAQDLDMRVIVFGHFGKNVPKAHKMSPDAFIQIALQLAYYRMYQRCCATYESASLRMFRLGRTDTIRSASSASAAFVKAFDNSSKQNTEKVDLMVKAVRAHRSYTNMAVSGQAIDRHLLGLKMVAVEKKLPMPDIFTDTAYAKALCYQLSTSQVPSKTDCVMCFGPVVPNGYGVCYNPMEDHINFAVSSFNSCEETNAAHLAQAVVEALLDMRTVLEQTPRPKL>Serine_sparus1_alphaKFFLLRGNHECAPINRIYGFYDECKRRYNIKLWKTFTDCFNCLPVAAIVDEKIFCCHGGLSPDLQSMEQVRRVMRPTDVPDQGLLCDLLWADPDKDVLGWGENDRGVSFTFGADVVTKFLHKHDMDLICRAHQVVEDGYEFFAKRQLVTLFSAPNYCGEFDNAGAMMSVDETLMCSFQILKPADKKLFYGGGGGMGSGRPVTPPRKS>Serine_sparus2_gammaNHECASINRIYGFYDECKRRYNIKLWKTFTDCFNCLPIAAIVDEKIFCCHGGLSPDLQSMEQIRRIMRPTDVPDQGLLCDLLWSDPDKDVLGWGENDRGVSFTFGSEVVAKFLHKHDLDLICRAHQVVEDGYEFFAKRQLVTLFSAPNYCGEFDNAGAMMSVDETLMCSFQILKPAEKKKPNGSRPVTPPRNMVTKQAKK>myomesin_sparus1_1aMSRSIQVTQKQQHHHQHQHHHHHQQQQHQQLEQHHYESGYHLSTKSSVSKQSFSSVQTSSHGLKTSVRTEKGQEVVKLSPLPKRAKRTYLAMDKDKEVIGYVIPVFRASHEAVRGLMEAQEEEVTEEGIQYVAMRNLFVREAKEAMHVRVEKKTRSTHVRESAERVEMSKTMDTWVEFRRKMNPDNLTHRPEFIVKPRGQTVWEGKTLKLHCTVAGWPKPRVAWYKNNVLIDAKAHPEKYTTESNYNMHSLEIKNCDFLDTAQYCASALNIKGEASSVATIVVKRFQDGEEAGPLDPKPHGFSPEHGVTFRTTILDKFEVAFGSEGETLSLGCTVIIYPTVKNYQPDVVWSRNSVPLKPSKWVHTHWSGERATLTLVHLNKEDEGMYTLRVNTKSGFDTYSAYVFVKDADVEVEGVPVAPLDVHCHDANKDYVVVTWKQPAVEGSSSILGYYIDRCEVGTHHWAQCNDVPVKYARFPVTGLVEGRSYTFRVRAVNKAGVSRPSRVSEAVVAMDPSDRARLRAGPSAPWTGMIKFTEEDPTVGVIPGEPTDVEVTEATKSYVVLAWKPPVQRGHEGVMYYIEKCVSGSDTWQRVNTGMPVKSPRFALFDLAEGKSYNFRVRCCNSAGVGESSVPTGEITVGDKLDLPSTPGNPVVTRNTDTSVVVSWAASKEVAHLVGYYIDCSVVGSNVWMPCNNKPVKKTRFVCHGLTTGENYVFRVKAVNAAGYSQSSHDSEAVAVKAAVSIPGKPNGVTLLEAAKDYMVLGWTAPANDGGADIRGYFVDYRTLKGDVVGKWHEMNHQALTTTSYKAENLKENVLYQFQVRAMNMAGVSKASLASAALECKEWTITVPGAPVGLHVLEVRDTSVVVLWEPPAFDGRTPVNGYYLDLKEASAGEEGLKAVHEKANKSKYMKVTGLKPGTSYVFSVRAQNLAGVGKPSAALGPILAQTRPGTKEIHVDVDDDGVISMVFECSEMSEGSEFIWSKNYKAITDTSRLTVVTENGKSRAIFNTPSLDDLGTYSCMVTGTDGFSSSYTLTEEGLMRLLDISHEHKFPVIPFKNEMAMELLEKGRVRFWTQLEKFTSACEVEYVFNDVIITQGKKYTMNFDKTTGIIEMFMDSLEVTDEGSFTFNLVDGKAKGTTSLVLIGDEFRTLQKKSEFERAEWVRKQGPHFVEYLGFTVTEECNVLLKCKIGNVKAETEITWSKDCIEIAEDDEDAQKIERKEGELTFNIGKISKADAGIYEVFLRDDRGRDKSTFSLTDAGYQAVLNELFRVIANSSSQVQITSTEHGIILFSNVTYHHEELRVGWLHKDTKIAASERVKSGVTGEQLWLKINEPTEKDKGKYAMDIFDGKDGVKRVFDLSGKAWEEAFEEFQRLKAAAIAERNRARVVGGLPDVVAIQEGKSLNLTGNVWGEPTPEVSWTKNEKELVPDEHYKLKFEHGKFASITIAAVTTADSGKYALVVKNKYGTEAGVFTVSVYNPEEEEKEEKKG>myomesin_sparus2_1bMNEDSLMHTPEFVIKPRSHTVWEKQCVRLHCTVSGWPDPRVVWYKNNVAIDPLASPGKYKLESRYSVHSLEINRCDFDDTAQYRVSAMNSKGELSAFASVVVKRFKGEIDDGLPEPRHGPVSEYGITFKTHIVDKFGVSFGREGETMSLGCTVIIYPALHRYQPEVQWYRDDVLLSASKWHHMHWSGDRATLTLTHLNKEDEGLYTLRVTTKSGYETYSAYVFVRDADAEIEGAPGAPLDVRCLDANKDYIIVTWKQPAVDGGNSILGYFVDRCEVGTNHWIQCNDTPVKFARFPVTGLVEGRSYTFRVRAVNKSGMSHPSRVSEPVAAMDPADRARMRGTSAPWTGQIIVTEEEPAEGVVPGRPLELQVTEATKNYVVLSWKPPAGKGLEGVMYYVEKCVSDTDSWQRVNTEIPVKSPRFALFDLAEGKSYSFRVRCCNSAGVGEPSDPTEATTVGDKLDIPSAPSKVVPTNNTDTSVVVSWEASRDAKELVGYYIEASMVDSNVWEPCNNKPVKGTRFFCHGLITGEKYVFRVRAVNAAGLSQFSPDSEPVEVKAAIASPAPPYGISVLECVRDSMVLGWKQPNFIGGADITGYFVDYREVIDGVPGKWHEANIRAVSERAYRVSDLKENKKYQFQVRAANMAGVGIPSLPSDTFLCEEWTIAVPGPPYDLQIREVRSDSLVLLWRPPVYQGRDPVNGFYIDIKEAEAPEEAWRGVNTKATEKTYFKIKNLKEAETYVFRVRAQNKAGVGKTSDVTEPVPALTKPGTKEIVVDVDDDGIISLNFECSELTQDSKFVWSKNYEEMTDTSRLTIETKGNKSKAAFNTPEEEDIGIYSCLVTHTDGASSSYTLSEEELKRLLVISHDHKFPIIPLKSELAVELLEKGRVRFWLQAEKISANGKVEYVFNDNVLSQGEKYKMNFDKNTGVIEMIMECLTPADEGTFTFQMTDGKATNQSSLVLIGDVFKELQKESEFQKKEWHRKQGPHFIEYLGYEVTPECCVVLKCKVGNMKKDTSALWYKDGREIKADNNLGFTEGVLKLEIAQISKKDSGVYEVVLKDDRGKDTSTLNLTDQGFKDLMNEVFSFIANSSTPLKITSTDQGIRLYTFVSYYNDLLQVTWHYKDSAIAFSDRIKSGVVGEQLWLQISEPTEKDMGKYAIEFSDGKGGLRRTVELSGQAFDDAFAEFQRLKAAAIAERNRARVAGGLPDVVTIQEGKALNLTCNISGDPVPEVTWLKNDREITSDDHCILKFESGKFATFTITGVNTSDSGKYSILVKNKYGTESADFTVSVFIPDEAGSKKK>epithelial_sparus1_EMP3aMVCLLISILVLHLTTLAMLLIATLEKSWWVWTDSEIRDLWYNCIHDNETKTWLCAATNESDWLQSVQALMVLSVVFSSISLLVFLGQLLTMSKGGLFYFTGLCQAFAGFTSFAACLIFTFHRKEILNDSRDLSRGRFGYCFILAWLCIPLLLISSVLYVHLRKKQ>epithelial_sparus2_EMP3bMAYLLMFVTLLHLITLAMLFIATMEKSWWEWEGLENSDLWYNCRFDNFTGSWLCASSKETEWLQAVQVLMVLSVVFSSVSFLVFLGQLFTMSKGGLFYFTGLCQIFAGLTAFSAALIYTLHNKEILQDSRELTSGHFGYCFILAWVCVPLLLCSGIIYIHLRKKE>microglobulin2_sparus1_b2ma1MTTSVFAVVFGLLCLQCSMAKESPPKVQVYSRVPGEFGKANTLICHVSGFHPPEITIELLKDEKEMPGAKQTDLAFEENWHYHLSRHVRFTPSEDEKYACRVTHMGTSKTFIWEADM>microglobulin2_sparus2_b2ma2MRIVFFLAALAAVYCSDKSKYSPPKVQVYTTGPGEFGVKNILICHVSGFHPPDITIQLMKEGEELSNANQTDLAFKPDWHFHLTKSVAFTPTRGERFSCKVTHGANMKEYVWVPNM>dehydrogenase_sparus1_7C_AMSLSSAMVLPLLIVVAAGVYYIYNEVMQFMSKSLVQNKVVVITDAMSGVGTECAHLFHKGGARLILCGTSWDKLESLYDSLTNDADPRETFAPKLVIVDFSDMPSMEEVVAEVVECYGCVDVLICNSSMKLKAPVQSVTLEVDRNIMDINYFGPSTLAKGVLPTMISRRSGHIVLVNSIQGRLAIPFRSSYAASKHAAQAFFDCLRAEVEEYGIVVSTISHTFINASEPPPVEEAGPKPNPLSAFIAKQMTHGVRPSVLADEIMQTVNRKRTEVMLAHPIPRVALYLRSLIPSFLFAVLGAGVKDSVLAEQMQ>dehydrogenase_sparus2_7C_BPRQGGRLSCRRSSVNELLRRAAPALLRRGINPESRTPAEAREADILQDMDPTWITTVLLVPCVIVLTAGFFYLYGVVIGLLSKTSVRNKVVVITDALSGLGRECAGVFHKGGARLILCGKSWEKLEEFADDLANASDPTVTFPPKLVLLDFGDMDSMPDVITEILECYGCLDVLILNSSMKVKAPAQSVSLEMDKLLMDNNYFGPATLAKGVLPSMISRRTGHLLLISSIQGKLAVPFRTTYAASKHAVQAFFDCLRAEVEEYGISVSTINHTFISPSSAENTEAASSRSVWSLYSKKPLGVSPDEAAAEIVKTLHNKKKEVVMAPSLPKVAIYARSFFPNVFFAVMAAGVNNAAACENM>solute_carrier_sparus1_38_member5aMELQKLSNGNHQHDSAGSVEGGVPPEEEKFLQHKSNGSKRPQFTDFEGKTSFGMSVFNLSNAIMGSGILGLSYAMSNTGIILFLILLTCIACLSCYSVHLLLCSAGVVGIRAYEQLGFRAFGHPGKILAAVIITLHNIGAMSSYLFIVKSELPLVIQAFLGQTSSSDDWFMNGNYLIIIVTVCIILPLTLMKHLGYLGYTSGFSLSCMVFFLSSVIYKRFKIACPLEVFGNYSVNTVVPEDTCTTKFFTINQETAYTIPILAFAFVCHPEVLPIYTELSNPTKRRMQNIGNVSILGMFIMYFFTAIFGYLTFYENTEAELLHTYSKVDPLDTLILCVRLAVLVAVTLTVPVVLFPIRRALLQLLFPGRPFHWLRHIIIAMCLLFAVNLLVILVPNIRDIFGITGATTAPSLIFILPGLFYIRIIPTNQEPMTSRPKIQAACFTALGFIFMTMSLTFIGIDWVSGEKRNLGGH>solute_carrier_sparus2_38_member5bAILSAYSIHLLLKSAGVVGIRAYEQLGNRAFGPPGKVLAAVIITIHNIGAMSSYLFIVKSELPLVIQAFLSKHENTGEWYLNGNYLIIIVSALIILPLALMRQLGYLGYTSGFSLSCMVFFLISVIYKRFNIACPFGDDHHNQTSFHDHVHINATDDQCEAKMFTVNSQTAYTIPILAFAFVCHPEVLPIYTELRDATKKRMQGVANISIMAMFVMYLLTALFGYLTFYGNVESELLHTYSRVDPLDVLVLCVRLAVLVAVTLTVPVVLFPIRRALLQILFADKPFSWAIHIGIAFCLLFLVNLLVILVPSIRDIFGLIGATSAPSLIFILPGIFYIRIVPEEQEPLLSRTKITAACFAALGFVFMVMSLSFIIIDWSTGESRSGGGH>dysferlin_sparus1_1aMKYNYHVPVSTYTRSSQYTPVYHTPSYYTPSHYTPTHYTPSYTSTTKYTPTQYSSTHYTPSRLTSTYKAASTYVPSYSKGSRYSSTQRAQEQERPAPVIPVTPAKRTVHFPNDIIFQDIVRRGDLEQIGRFMRARKVRVDTLFHSGMAALHEAVLTGNLEVVKLLVKYGADVHQRDEDGWTPLHMACSDGYPEIARYLLSMGASTEAENESGEKPADLIDPDYKDLAKLFEAGCA>dysferlin_sparus2_1bMCPVSQTMGIKAYTQDCGVPVSCKSSASLKPIRSVHFPNDIVFQDYIRQGELERIGRFIRARRVSLQTIYHSGMAAIHEAVLSGNLECVKLLIQYGADIHQRDEEGWTPLHMACSDGFPHIARYLLSLGANPELETDSGDKPADLIEPDNKELLEIFGLAVND>acetylcoline_sparus1_a.1MKTGFFIFHLVILAGAAWASTDETRLVKTLFTGYNKVVRPVTHFKDPVVVTVGLQLIQLISVDEVNQIVSSNVRLKQQWKDVNLLWDPEDYGGIKKIRVPSTDIWRPDLVLYNNADGDFAIVHETKVLLEYTGMITWNPPAIFKSYCEIIVLHFPFDLQNCSMKLGTWTYDGNLVVVNPVIPFRPGKLLHFRKYICLLFNFLLSNSENNSNTNPKLAAG>acetylcoline_sparus2_a.2MNLSMVQIVLAWILCVFAGPVSSSADETSLVKTLFTGYNKVVRPVNHFSEAVVVTVGLQLIQLISVDEVNQIVTSNVRLRQQWLDVNLKWNPDDYGGIRKIRVPSTDIWKPDLVLYNNADGDFAIIHETKVLLEHTGMITWNPPAIFKSYCEIIVLHFPFDLQNCSMKLGTWTYDGLLVVINPDSDRPDLSNFMESGEWVLKDYRSWKHWVYYTCCPSPSSCWLLWS>Sparus1_DUPD1aMPRGFSCLASPEGADGRYETPPASELQRLMWTKKGTSSHLDEVQPRIYIGDMYAAKDKRTLQAHHITHVLNAADGKFNVNTGPSFYRDTKITYYGVEAFDMPSFNLSPFFYPAANFIKNALSSPTGKVFVHCAMGLSRSSTLVLAYLMIHENMSLADAIKGVSANRNISPNNGFLEQLRELDKQLHGQVSSRSLR>Sparus2_DUPD1bEEKQRRAMSSGVVKSRGRNPYTAVRVDPDSDYITPGTLDLEQLFWAGNGAQYAHVNQVWPSLYIGDEKTALECPGLRDLGITHVLNAAEGKFNNVLTGADYYCDMDIQYFGVEADDKPTFNISQYFCSATQFIHDALSHPQNKVLVHCVMGRSRSATLVLAYLMMKHSLSVVDAIEHVRQRRCILPNHGFLKQLRALDITLQEERLRQKREIQDQ>sparus1_TIMP2aLQLRCRYQAKLVGEQEVEVGNDIYGNPIKRIKYEIKQIKMFKGPSQDIDAIYTAPTSAVCGVTLETNGKEYLIAGKLEADGTMHVTLCDFILPWDDTSATQRKSLTQRYEMGCDCKITRCTSIPCMITSPAECLWLDWVMEKSVNGQQAKHYACIKRSDDSCAWYRGSAPPKRDFLDIEDP>sparus2_TIMP2bMPWDTLSNTQKKGLSQRYQMGCECKIVRCPSLPCEISAPEECLWTDLMIEKQVYGRQANHYACIKRADNSCSWYRGVSSPKKEFLDGEEP>sparus1_retinoid_gammaMDSNDPYLHLNSTGPMSTVHTHPPHMGGMMGHPSVISSSRPLPSPMSTLGSPMNGLASPYPVITSSLGSPSVSLPSTPNMNFGPLNSPQMNSMNSVSSSEDIKPPPGLQSLGNINYQCTSPGGMSKHICSICGDRSSGKHYGVYSCEGCKGFFKRTVRKDLTYTCRDSKECLIDKRQRNRCQYCRYQKCLAMGMKREAVQEERQRGKERGESEVESTSSFNEEMPVDKILDAELAVEPKTETYSDGSPSNSTNDPVTNICQAADKQLFTLVEWAKRIPHFSELPLDDQVILLRAGWNELLIASFSHRSVTVKDGILLATGLHVHRSSAHSAGVGSIFDRVLTELVSKMKDMQMDKTELGCLRAIVLFNPDAKGLSNPPEVEGLREKVYASLESYTKQKYPDQPGRFAKLLLRLPALRSIGLKCLEHLFFFKLIGDTPIDTFLMEMLEAPHQIT>sparus2_retinoid_betaMHSISSSEDVKPPFGLRPMQSHSPGLMLSQKRMCVICGDRSSGKHYGVYSCEGCKGFFKRTVRKDLSYTCRDNKECLVDKRQRNRCQYCRYQKCLAMGMKREVVKHVKWNKEDGKDEGWMTVQEERQRNREREGELEFSVGVNEEMPVEKILEAETAVEQKTELHSDGGSAGNSPHDAVTNICQTADKQLFALVEWAKRIPHFSELPLDDQVILLRAGWNELLIASFSHRSIALKDGVLLASELQRDSAHSAGVGAIFDRESVQSAEVGAIFDRVLTELVNKMRDMQMDKTELGCLRAIVLFNPDAKGLSNTSEVELLREKVYASLEAYCKQRYPEQQGRFAKLLLRLPALRSIGLKCLEHLFFFKLIGDTPIDTFLMEMLEAPHQLS>sparus1_Juntophilin1aYRGEWSHGFKGRYGVRQSHNTPARYDGTWSNGLQDGYGIETYGDGGTYQGQWMGGMRHGYGVRQSVPYGMATVIRSPLRTSLASLRSEQSNGTVLQDLSSPTDTPTGSRGGFVLNFHSDSEVVTGKKKGLFRRGSLFGSLRQLRKSDSRTSISSKRSSARSDAAMSRISSSDANSTISIGDGELQDEDLPLEDHVDATTTETYMGEWKNDKRNGFGVSERSNGMKYEGEWLNNKRHGYGCTVFPDATKEEGKYKNNVLVRGIRKQLIPLKNPKTKEKVDRAVEGAQRAAAIARSKVEIAASRTAHARTKSEAAEQAAVSAVHDSEIARAVARELSPNFYQPGPDYIKQQSKEPVEIKEVPVEKKEKSPKDSPHFYRKGTTPPHSPVTSPVATPPPSPLSSKKKGQLANSTSRKTSKEEKPSRKISKEERSSHKASKEERPNRKLSKEEGPSVPDGPKSSHVHVEAPAKPAKTHQPTAASAPPPPQPPAVPVNGELHTEYHSYYVKPPTKGPPPPDPEEDIEEEPSALALARMPPQPPRSFSTPTPKP>sparus2_Juntophilin1bTWSNGLQDGYGVETYGDGGTYQGQWTGGMRHGYGVRQSVPYGMASVIRSPLRTSLASLRSEQSNGTVLRDSLSDSPAGTRGGFVLNFHSDGEGSEKKKGLFRRGSLFGSLQRLRKSDSRSSISSKRSSAHSDTTMSRISSSDANSTISFGDGDPGDDYLPLEDNVDATTTESYMGEWKNDKRSGFGVSERSNGMKYEGEWLNNKRHGYGCTIFPDGTKEEGKYKNNTLARSIRKQLIPLKNTKTKQKVDRAIEGAVRAAAIARTKVEIAISRTSHARAKAEAADQAAHAASQDSDIARAVARELSPAFHQPGPDYIRQKYNEPVEVKEVPVEEKKEKSTSGSPHFYRKGTTPTQSPSQSPGPSPTPSPAAVKKSFFTSKTPATVPPALKDNKTPTAESLTAGITKAASKLELRKQEPPPSVKKEVIATGSSYPSNGQIHSQYHGYYIKADVKITPPEEPIGVDDDFHPSSLARLPPPAKQIQAPAPKPLPAPSPSPVPPKENVKAPEPSKPKRQESTKPKNLAETKKASVEIVPEIAEEESGPNSILVALVMLLNVGLAIIFVHFLT>sparus1_rpl5aMGFVKVVKNKSYFKRYEVKFRRRREGKTDFFARKRLVVQDKNKYNTPKYRMIVRFSNRDLCCQIAYAKIEGDHIVCAAYSHELPKYGITVGLTNYAAAYCTGLLLARRLLHKFGMDQAYEGQVEVTGDEFNVESIDGQPGAFTCYLDAGLARTTTGNKVFGALKGAVDGGLAIPHSLKRFPGYDTESKEFNAEVHRKHILGMNVADYMSYLMEEDEDAYKKQFSRFIKNGVTPDTVEEMYKKAHVGIRANPVHEKKPKKDVKKKRWNRAKLSLAQRKDRVAQKKASFLRAQEQEEGDG>sparus2_rpl5bMGFVKVVKNKAYFKRYQVKFRRRREGKTDFFARKRLVVQDKNKYNTPKYRMIVRFSNRDIVCQIAYAKIEGDMIVCAAYSHELPKYGVTVGLTNYAAAYCTGLLVARRLLNKFGLDKVYEGQVEVTGDEFNVESIDGQPGAFTCYLDAGLARTTTGNKVFGALKGAVDGGLSIPHSTKRFPGYDPESKEFNAEVHRKHIMGVNVSEYMSLLMEEDEDAYKKQFSRFIKNGVTPDSIEEMYKKAHATIRENPVHEKKPPKEVKKKRWNRAKLSLAQRKDRVAQKKASFLRAQEQEASD>sparus1_smyd1aMTVGNMESVELFDAGKKGRGLRATRELNTGEVVFAEPSFAAVVFDSLATQVCHSCFRHQANLHRCAQCKFAHYCDRTCQTACWDEHKQECGAIKKLGKAPSENVRLAARVLWRIHKDTGIASDSQLVSVDQLEDHMADLPEEDLKKLESDVHTFQGYWSYGRKQHPAEYISRIFGIIKCNGFTLSDQRGLQAVGVGLFPNLCLVNHNCWPNCTVILNHGNQSAVSSTLHSQRRIELRAMGMIPEGAELTVSYVDFLSVSADRQKKLKERFHFECTCEHCSQHIKDDLMMATAEGKPSADKVKEVTAFSKERLEKIEKSRIERDYPEVVKLCRECLEKQENVLADTHLYKLRVLSIAGEVLSYLHHFSEAANHARRMVEGYTKLYHPNNAQLGMAIMRAGVTHWHAGQIEAGHGMICQAYRILMVTHGPNHAITRDLETMRRQTEIELKMFKQNEDEYHAMREAALKK>sparus2_smyd1bMENMAIFDSPGKGRGLKATKEFWAGDVIFSEPSLAAVVFDSLAERICHSCFRSQPKLQRCGQCKFAHYCDRTCQRAGWAEHKQECSAIKAFGKAPNESIRLVARLLWRLDKEGSVVSDMQLTTLDELEDHIQDMEEDDLKDFKVDIHNFVDYWPRSSKQHRIEEVSHIFGVVNCNGFTVSDQRGLQAVGVGLFPNLCLVNHDCWPNCTVILNHGKIELRSLCKIEEGEELTVSYVDYLNLSEERQRLLKTQYFFDCTCEHCRDKIKDDLKLAGREVDGVKPSPEQVKEATDYLFEMLEKIEKARIRADYHEVVKICRDSITKTEPVLADTHIYLLRLWSTLSEVQAYLQYFEDAAEYARKMVDGYVKLYHPNNAALGMAAMRAGVNHWQAGLIEIGHAMVCKAYAILMVSHGPTHPITKDLEAMRMQTEMELRMFKQNEYVYHSMRDAALKNKPMTMLHEPKGVEEGIKNLFHRKK>sparus1_terc_aMDVLALEAKSVNGAEADKKSAPRPKPKPPKKAKRIVYFEVEIVDLKTKEKLLLLDKVEPTATVLDIKALFQKSYPKWYPARQSLRLDPKAKCLKDEEVLQTLPVGTTASFYFSDLGPQLTWGTVFLAECAGPLIIYLMFYFRLPFIYSPKYDFTTSKHWVVHLACMCHSFHYIKRILETMFVHRISHGTMPLRNIFKNCGYYWCTAAWMAYYINHPLYTTPYYGQQQVNSGLYVFLFCQVGNFSIHVALRNLKLPGSKAKKIPYPTKNPFTWIFWLVSCPNYTYELGSWMGFTVMTQCVPVAFFTVVAFVQMTVWAKGKHRSYLKEFRDYPTLRSSILPFIL>sparus2_terc_bMDTLALEATGSKKAVNGAVAVPPLVPVQAPVKRKPAKKAKKAVVFFEVEILDAKTKDKLCFLDKVEPNATIGEIKSMFHKSHPQWYPARQSIRLDPKGKSLKDEDVLQHLPVGTTATFYFRDLGAQISWVTVFLTEYTGPLVIYLMFYFRVPFIYAPKYDFTTSKHWVVHLACMCHSFHYVKRLLETLFVHRFSHGTMPLRNIFKNCTYYWGFAAWMAYYINHPLYTPPIYGEQQIRLALIVFLFCQIGNFSIHVALRNLRPPGSKTRKIPYPTKNPFTWIFLLVSCPNYTYELGSWLGFTLMTQCLPVAFFTLVGFIQMTVWAKGKHRSYLKEFRDYPPLRSPILPFVL>sparus1_ald6a1.aRLLLPTVLPAELVKLSRPLRNNISTMATTALRSVLKSKVPLKVGRLCYSSSVPTTKLFIDGKFVESKSSEWLDIHNPATNEVISRVPKATQEEMLAAVDSCSRAYRSWSETSILARQQVFLRYQQLIKDNIKELAKSITVEQGKTLADAEGDVFRGLQVVEHACSVTSLMLGETMPSVTKDMDIYSYRLPLGVCAGIAPFNFPAMIPLWMFPMAMVCGNTYLLKPSERVPACAMLLVKMLQDSGAPDGSLNVIHGQHDAVNFICDHPAIKAISFVGSNQAGEYIYERGSKNGKRVQSNMGAKNHGVVMPDANKENTLNQLVGAAFGAAGQRCMALSTAILVGEARSWLPELVERSKALRVNAGDQPGADVGPLISPQAKSKVCSLIQSGLDEGAKILLDGRNVNVKGYEHGNFVGPTIIGNVKPDMKCYTEEIFGPVLVVLEADTLDDAISLVNRNPYGNGTAIFTTNGATARKYTHEVDVGQVGVNVPIPVPLPMFSFTGSRGSFRGDMNFYGKQGIQFYTQIKTVTSQWKAEDATLKSPAVTMPTMGR>sparus2_ald6a1.bMAAILLRSLLSKKVPLQLGRCYSASSVPTTKLFIDGKFVESNTSEWLDIHNPATNEVVGRVPKATQEEMLAAVDSCSRAFYSWSETSILSRQQIFLRYQQLIKDNIKELAKIITLEQGKTLADAEGDVFRGLQVVEHACSITSLMLGETLPSITKDMDTYTYRLPIGVCAGIAPFNFPAMIPLWMFPIGMVCGNTYLMKPSERVPGCTMLLAKLLQDSGAPDGTINIIHGQHAAVNFICDHPAIKAISFVGSNSAGEYIYERGSKNGKRVQSNMGAKNHGVIMPDANKENTINQLVGAAFGAAGQRCMALSTAIFVGDSREWLPELVERSKSLRVNAGDQPGADVGPLISPEAKARVEFLIQTGVDEGAKLLLDGRRVSVKGYENGNFVGPTILADVTPSMTCYREEIFGPVLVVLEAENLDEAISLINNNQYGNGTAIFTTNGATARKYTHEVDVGQIGVNVPIPVPLPMFSFTGSRGSFRGDTNFYGKQGIQFYTQIKTVTSQWKAEDATVTSPAVTMPTMGR>sparus1_EIF4E3aMAVPVAALQLSTSSSPVLLEHNIHISDRDLGRISSGNEIDTLPLHSPWTFWLDRSLPGTTAAECESNLKKIYTVETVQNFWRVYNNIPGVSSLPLRCSYHLMRGERKPLWEEESNAKGGVWKMKVPKECTSVVWKELLLATIGEQFSDYCASDDEVVGVSVSVRDREDIFQVWNGNASCANESNILGRIHELLPQIPFKAVFYKPHEEHHAFEGGRSRH>sparus2_EIF4E3bTGGGRILKTIRRHSAQMAVPAGHTDVQTDRGGLSGQTVSAENNIDIDEKELENITKKHREDETTTLPLHSPWTFWLDRSLPGTTAAECESNLKKIYTVQSVQMFWSVYNNIPPVTALPLRCSYHLMRGERRPLWEEESNAKGGVWKMKIPKESASVVWKELLLATIGEQFADYCSSDDEVVGVSVSVRDREDVVQIWNKDASLATESNILGKVYELLPSITFKAVFYKSHMEHHAFEGGRSRH>Sparus1_FKBP1A.1PDSCFRSHCRVVDKEGDERANMGVEIETISPGDGQTFPKKGQRVVVHYVGTLPDGKVFDSSRSRGRPFKFKIGHQEVIRGWEEGVAQMSVGQRAKLTCSPDFAYGSKGHPGIIPPNCTLTFDVELIGLEA>Sparus2_FKBP1A.2MGVEIETITPGDGRTFPKKGQTCVVHYVGSLTDGRKFDSSRDRDKPFRFKIGKQEVIRGWEEGVVQMSVGQRAKLTCSPDYAYGNKGHPGIIPPNATLIFDVELLGLE>sparus1_srs11aMNYTTKVVQVTNVSPSTTSEQMRTLFGFLGTIEELKLFPPDDSPMPVTSRVCFVKFQEPESVGVSQHLTNTVFVDRALIVVPFAEGSIPDEAKALSLLAPANAVAGILPGGGLLPTPNPMPNPPMGGNPFGAPNMDAMAAFGFPGPNMNPQAADQLLKFMTDPKLNPLAAGLNLSASLKADASNKEIEEAMKRVREAQSLISAAIEPGNKESKKKRSRTRSRSRRRRSRSRSRHRRSRSRSRRRSNSRNRRRSKSPRRKRTHSRDRGRRSPSRSRDRKKDDSGRRRSKTPPKSYSTARRSRSASRRRRRSRSGSRSPKVSPKRRISRSPSPRRHKKEKKRDKERERNSDKDRSREEPERSASKKKKSKDKERERERKSDGEKGDIKVGKITRDYDEEEQGYDSEKEREDRKDSDDSALSPQSVEGNGTARLVKAKVNGADDRHEEDMDVSD>sparus2_srsf11bMNSNTHVIQVTNVSPSTTSEQMRTLFGFLGNIEELKLFPPDDSPLPVTSRVCFVKFLESESVGVSQHLTNTVFVDRALIVVPFAEGVIPDESKAMSLLAPANAVAGMMPGGGLLPTPNPLASMGGTPFGGLGAPNMEQMAAMGMPGPNMNPQALSADFLKLMQSMDPKLNPLAAGLNLNPGLKTDASNKEIEEAMKRVREAQSLISAAIEPGNKKDDKRKHSRSRSRSRRRRSSSRSRHRRSKSRSRRRSHSRSRRRSKSPRRRRSHSRDRSRRSRSRDRRKEEKSKKRSKTPPKSYSSARRSRSISRRHRRSRSASRSPKRRLSRSPSPRRHKKEKKKDKEREKDRDRERREDRDRSRDERERSTSKKKKSKDKERDRDRKSDSEKGDVKVTRDYDEEEQGYDSEKEGEEDEDERKSDSDSASSPKGQEEMERSEGQIPKKSKLNGDDHHQEDMEMSD>isotig04733_ORF|kelch repeat and btb domain containing 10MDPNAIKEELRLFQSTLLQDGLKELLNENKFVDCTLKVGDRSFPCHRLIMAACSPYFREIFFTEDGKEVENTKEVVLDDVNPSILDMIVQYLYSAEIDLTDDNVQDIIAVANRFQIPSVFTVCVNYLQKKLSLSNCMAIFRMGLVLNCPRLAVAARNYIADRFEFLYKDEEFLKLAPHELFAVIGGDTLNVEKEELVFEAVMAWVRYDKERVKVLKDAFNCIRFRLLPEKYFKDKVETDELIKADPELQTTIQVVKDAYKGKLPEKPKKEEGKEGAGEEGEEEESPFPGFLNDNRRHGMYTRDFILMINDTAAVAYDVSENECFLAAMSEQVPRNHVSLVSQRNQLYIIGGLFVDEENKDVPLQCYVYLLDPLTSDWVAMPPMPSPRCLFNIGESENLLFAVAGKDLQTNESLDTVMCYDVDKMKWSETKKLPLKIHGHAVVSHKGLVYSIGGKTDDNKALNKMFAYNHKQSEWRELAAMKTPRSMFGAVVHNGKILVVGGVNEEGLTASCEAYDFASNKWEPFTEFPQERSSVNLLSNGGCLYAVGGFAMVQMESKEVAPTEVTDVWQFEDDKKQWSGMLREMRYAAGSSCVSMRLNAARMPKL>isotig05371_ORF|kelch repeat and btb domain containing 5MAAPINPMDEPRVYQQTLLQDGLYDLLENDKLVDCVLKIKDKEFPCHRLVLCACSSYFRAIFLSDLDESKKKEIVLEDVEPGVMGLILKYLYTSKINVTEQNVQDIFAVANVYQIPSIFTVCVSFLQKRLSLSNCLAIFRLGLMLDCPRLAVSARNYACERFQLISRDEDFLQLQPSELAAILTNDNLNVETEEAVFEALMSWVSRDAENREKELPDLLDCVRLRLVKEDYLKEKVEKHKLISSNPDLQQKIQLVRDAHAGKMPEVKPTKSKKEEGGAEKDGDGENEDNEEEEALLPGILNDNLRFGMFVRNLILMVNDTGAVAYDPTGNDCFVATLSTQVPKNHISLVTRENQIFVAGGLFFDEQNKEDPLCSYFLQYDPVSADWLGMPPVPSPRFLFGLAEAENSIFVLGGRELKEQEPMLDSVLVYDRQSFKWGESDPIPYPVYGHATVSHNDVVYVIGGKGDSKSCLRKMCAYDAKRFEWKELAPMKTARSLCGATVHKGKIYVAAGVTDTGLTNTMEVYDIASNKWSDHAEFPQERSSLNLVSLAGLLYAVGGFAMMPLEDSDEIVPKEMNDIWRFDEKEQKWYGILREIQYASGATILGVRLNTLRLTKM
