# Supplementary material for: Fast skeletal muscle transcriptome of the Gilthead sea bream (Sparus aurata) determined by next generation sequencing
Source: BMC Genomics. 2012 May 11;13:181. doi: 10.1186/1471-2164-13-181 (PMC3418159; doi:10.1186/1471-2164-13-181)
Supplement: Additional file 14 — Detailed list of transcription related isotigs found in the gilthead sea bream transcriptome. [file 1471-2164-13-181-S14.docx]

| Isotigs ID v2.5 | Transcription factor (TF) name | TF symbol | Human Gene ID | Takifugu Gene ID | UniProt annotation | TF Family (TFconse based classification) |
| --- | --- | --- | --- | --- | --- | --- |
|  |  |  |  |  |  |  |
| isotig06206 | p65 transcription factor | RELA | ENSG00000173039 | SINFRUG00000124092 | REL family | Beta-scaffold |
| isotig08434 | transcription factor cp2 | TFCP2 | ENSG00000135457 | SINFRUG00000131238 | CP2 family | Beta-scaffold |
| isotig03815 | nuclear factor 1a | NFIA | ENSG00000162599 |  | CTF/NF-I family | Beta-scaffold - CCAAT |
| isotig07170 | nuclear factor 1b | NFIB | ENSG00000147862 | SINFRUG00000155441 | CTF/NF-I family | Beta-scaffold - CCAAT |
| isotig10365 | nuclear factor 1c | NFIC | ENSG00000141905 | SINFRUG00000142705 | CTF/NF-I family | Beta-scaffold - CCAAT |
| isotig29184 | nuclear factor 1 x-type | NFIX | ENSG00000008441 | SINFRUG00000141511 | CTF/NF-I family | Beta-scaffold - CCAAT |
| isotig36295 | nuclear transcription factor beta | NFYB | ENSG00000120837 | SINFRUG00000147066 | CTF/NF-I family | Beta-scaffold - CCAAT |
| contig02195 | myocyte enhancer factor 2a | MEF2A | ENSG00000068305 | SINFRUG00000141348 | MEF2 family  MADS domain | Beta-scaffold - MADS |
| isotig06139 | myocyte enhancer factor 2c | MEF2C | ENSG00000081189 | SINFRUG00000160218 | MEF2 family  MADS domain | Beta-scaffold - MADS |
| isotig07505 | myocyte enhancer factor 2d | MEF2D | ENSG00000116604 | SINFRUG00000152421 | MEF2 family  MADS domain | Beta-scaffold - MADS |
| isotig19952 | serum response factor | SRF | ENSG00000112658 | SINFRUG00000133109 | MADS domain | Beta-scaffold - MADS |
| isotig01735 | upstream binding protein 1 (lbp-1a) isoform cra_b | UBP1 | ENSG00000153560 | SINFRUG00000144619 | CP2 Family | Beta-scaffold - Others |
| isotig05979 | signal transducer and activator of transcription 1 | STAT1 | ENSG00000115415 | SINFRUG00000133230 | STAT family  SH2 domain | Beta-scaffold - Others |
| isotig06145 | signal transducer and activator of transcription 3 | STAT3 | ENSG00000168610 | SINFRUG00000156291 | STAT family  SH2 domain | Beta-scaffold - Others |
| isotig08082 | nuclear factor of activated t- calcineurin-dependent 3 | NFATC3 | ENSG00000072736 | SINFRUG00000148851 | RHD domain | Beta-scaffold - Others |
| isotig08589 | signal transducer and activator of transcription 5b | STAT5B | ENSG00000173757 | SINFRUG00000133369 | STAT family  SH2 domain | Beta-scaffold - Others |
| isotig13138 | signal transducer and activator of transcription 6 | STAT6 | ENSG00000166888 | SINFRUG00000152141 | STAT family  SH2 domain | Beta-scaffold - Others |
| isotig16772 | nuclear factor nf-kappa-b p105 subuni | NFKB1 | ENSG00000109320 |  | RHD domain | Beta-scaffold - Others |
| isotig17265 | nuclear factor of activated t-cells 5 | NFAT5 | ENSG00000102908 | SINFRUG00000128794 | RHD domain | Beta-scaffold - Others |
| isotig18423 | grainyhead-like 2 | GRHL2 | ENSG00000083307 | SINFRUG00000165510 | CP2 family | Beta-scaffold - Others |
| isotig19406 | nuclear factor nf-kappa-b p100 subunit | NFKB2 | ENSG00000077150 | SINFRUG00000148742 | RHD domain | Beta-scaffold - Others |
| isotig26238 | v-rel reticuloendotheliosis viral oncogene homolog | REL | ENSG00000162924 | SINFRUG00000127895 | RHD domain | Beta-scaffold - Others |
| isotig35162 | tumor protein p63 | TP73L | ENSG00000073282 | SINFRUG00000122073 | P53/SAM | Beta-scaffold - p53 |
| isotig13326 | transcription factor cbfa1 | RUNX2 | ENSG00000124813 | SINFRUG00000131546 | - | Beta-scaffold - RUNT |
| isotig01397 | hypoxia-inducible factor 3 alpha | HIF3A | ENSG00000124440 | SINFRUG00000141583 | bHLH domain | bHLH |
| isotig03230 | transcription factor 12 | TCF12 | ENSG00000140262 | SINFRUG00000134912 | bHLH domain | bHLH |
| isotig03231 | transcription factor e2-alpha | TCF3 | ENSG00000071564 | SINFRUG00000125838 | bHLH domain | bHLH |
| isotig03497 | class b basic helix-loop-helix protein 2 | BHLHB2 | ENSG00000134107 | SINFRUG00000142673 | bHLH domain | bHLH |
| isotig03862 | mlx interacting protein | MLXIP | ENSG00000175727 | SINFRUG00000121096 | bHLH domain | bHLH |
| isotig06410 | dna-binding protein inhibitor id-2 | ID2 | ENSG00000115738 | SINFRUG00000131034 | bHLH domain but not direct DNA binding domain. Regulates transcription. | bHLH |
| Isotig16828 | dna-binding protein inhibitor id-3 | ID3 | ENSG00000117318 |  | bHLH domain but not direct DNA binding domain. Regulates transcription. | bHLH |
| isotig04984 | dna-binding protein inhibitor id-1 | ID1 | ENSG00000125968 | SINFRUG00000154866 | bHLH domain but not direct DNA binding domain. Regulates transcription. | bHLH |
| isotig03497 | class e basic helix-loop-helix protein 40 | BHLHB40 | ENSG00000134107 | SINFRUG00000142673 | bHLH domain | bHLH |
| isotig06509 | max protein | MAX | ENSG00000125952 | SINFRUG00000125176 | bHLH domain  MAX family | bHLH |
| isotig07514 | transcription factor 15 | TCF15 | ENSG00000125878 | SINFRUG00000123382 | bHLH domain | bHLH |
| isotig09375 | transcription factor 25 (basic helix-loop-helix) | TCF25 | ENSG00000141002 |  | TCF25 familty  bHLH domain | bHLH |
| isotig11048 | hypoxia-inducible factor 1 alpha | HIF1A | ENSG00000100644 | SINFRUG00000154390 | bHLH domain | bHLH |
| isotig06261 | aryl hydrocarbon receptor 2 | AHR | ENSG00000106546 | SINFRUG00000126702 | bHLH and PAS domains | bHLH |
| isotig12396 | aryl hydrocarbon receptor nuclear translocator-like protein 1 | ARNTL 1 | ENSG00000133794 | SINFRUG00000164117 | bHLH and PAS domains | bHLH |
| isotig14512 | neuronal pas domain protein 2 | NPAS2 | ENSG00000170485 | SINFRUG00000141792 | bHLH and PAS domains | bHLH |
| isotig16426 | transcription factor hes1 | HES1 | ENSG00000114315 | SINFRUG00000139781 | bHLH domain | bHLH |
| isotig16746 | circadian locomoter output cycles protein kaput | CLOCK | ENSG00000134852 | SINFRUG00000126258 | bHLH and PAS domains | bHLH |
| isotig17741 | transcription factor 4 | TCF4 | ENSG00000196628 | SINFRUG00000125644 | bHLH domain | bHLH |
| isotig25448 | max gene associated | MGA | ENSG00000174197 | SINFRUG00000143334 | bHLH and T-box domains | bHLH |
| isotig31871 | period circadian protein homolog 1 | PER1 | ENSG00000179094 | SINFRUG00000149376 | PAS domain | PAS |
| isotig32336 | period homolog 3 | PER3 | ENSG00000049246 | SINFRUG00000121784 | PAS domain | PAS |
| isotig34166 | aryl hydrocarbon receptor 2b | ARNTL2 | ENSG00000029153 | SINFRUG00000134985 | bHLH and PAS domains | bHLH |
| isotig38183 | musculin | MSC | ENSG00000178860 | SINFRUG00000164037 | bHLH domain | bHLH |
| isotig02242 | ccaat enhancer-binding protein delta | CEBPD | ENSG00000180733 | SINFRUG00000164900 | bZIP family | bZIP |
| isotig02589 | x-box binding protein 1 | XBP1 | ENSG00000100219 | SINFRUG00000147763 | bZIP family | bZIP |
| isotig04495 | ccaat enhancer-binding protein zeta | CEBPZ | ENSG00000115816 | SINFRUG00000124288 | bZIP family | bZIP |
| isotig02854 | camp responsive element-binding protein | CREB1 | ENSG00000118260 | SINFRUG00000127266 | bZIP family | bZIP |
| isotig02855 | camp-responsive element modulator | CREM | ENSG00000095794 |  | bZIP family | bZIP |
| isotig02896 | cyclic amp-dependent transcription factor atf-4 | ATF4 | ENSG00000128272 | SINFRUG00000136875 | bZIP family | bZIP |
| isotig03291 | creb atf bzip transcription factor | CREB/ATF bZIP | ENSG00000118260 | SINFRUG00000127266 | bZIP family | bZIP |
| isotig03591 | Thyrotroph embryonic factor. | TEF | ENSG00000167074 | SINFRUG00000160902 | bZIP family | bZIP |
| isotig05508 | tsc22 domain member 3 | TSC22D3 | ENSG00000157514 | SINFRUG00000134123 | bZIP family | bZIP |
| isotig05571 | proto-oncogene protein c-fos | FOS | ENSG00000170345 | SINFRUG00000132419 | bZIP family | bZIP |
| isotig06533 | btb and cnc homology basic leucine zipper transcription factor 1 | BACH1 | ENSG00000156273 | SINFRUG00000151757 | bZIP family | bZIP |
| isotig08267 | camp responsive element binding protein 3-like 3 | CREB3L3 | ENSG00000060566 | SINFRUG00000151577 | bZIP family | bZIP |
| isotig08553 | activating transcription factor 1 | ATF1 | ENSG00000123268 | SINFRUG00000152820 | bZIP family | bZIP |
| isotig09048 | dna-damage-inducible transcript 3 | DDIT3 | ENSG00000175197 | SINFRUG00000133607 | bZIP family | bZIP |
| isotig09668 | nuclear factor erythroid 2-related factor 1 | NFE2L1 | ENSG00000038615 | SINFRUG00000131651 | bZIP family | bZIP |
| isotig11339 | nuclear respiratory factor 1 | NRF1 | ENSG00000106459 | SINFRUG00000133458 | NRF1/Ewg family | bZIP |
| isotig12462 | jun dimerization protein 2 | JDP2 | ENSG00000140044 | SINFRUG00000143786 | bZIP family | bZIP |
| isotig13077 | jun b proto-oncogene | JUNB | ENSG00000171223 | SINFRUG00000142362 | bZIP family | bZIP |
| isotig13786 | tsc22 domain family protein 1 isoform 1 | TSC22D1 | ENSG00000102804 |  | bZIP family | bZIP |
| isotig15489 | transcription factor maf | MAF | ENSG00000178573 | SINFRUG00000162641 | bZIP family | bZIP |
| isotig16568 | nuclear factor erythroid-derived 2 | NFE2 | ENSG00000123364 | SINFRUG00000149366 | bZIP family | bZIP |
| isotig23238 | activating transcription factor 7 | ATF7 | ENSG00000170653 | SINFRUG00000145238 | bZIP family | bZIP |
| isotig23898 | activating transcription factor 2 | ATF2 | ENSG00000115966 | SINFRUG00000143403 | bZIP family | bZIP |
| isotig28059 | transcription factor jun-d | JUND | ENSG00000130522 | SINFRUG00000138228 | bZIP family | bZIP |
| isotig28588 | nuclear factor erythroid derived 2-like 1 | NFE2L1 | ENSG00000108511 | SINFRUG00000156875 | bZIP family | bZIP |
| isotig02737 | chromobox protein homolog 3 | CBX3 | ENSG00000106038 | SINFRUG00000165271 | Chromo domain Chromatin remodeling | Chromatin-associated |
| isotig03293 | yy1 transcription factor | YY1 | ENSG00000100811 | SINFRUG00000136422 | Chromatin remodeling | Chromatin-associated |
| isotig03496 | male-specific lethal 3-like 1 | MSL3L1 | ENSG00000005302 | SINFRUG00000136243 | Chromo domain  Chromatin remodeling | Chromatin-associated |
| isotig03934 | chromodomain helicase dna binding protein 4 | CHD4 | ENSG00000111642 | SINFRUG00000133518 | Chromo domain  Chromatin remodeling | Chromatin-associated |
| isotig04372 | chromobox protein homolog 5 | CBX5 | ENSG00000123364 | SINFRUG00000149366 | Chromo domain  Chromatin remodeling | Chromatin-associated |
| isotig04613 | bromodomain containing 7 | BRD7 | ENSG00000166164 | SINFRUG00000132076 | Bromo domain  Chromatin remodeling | Chromatin-associated |
| isotig05090 | high-mobility group 20b | HMG20B | ENSG00000064961 |  | High mobility group  Chromatin remodeling | Chromatin-associated |
| isotig05524 | chromodomain helicase dna binding protein 2 | CHD2 | ENSG00000173575 | SINFRUG00000151294 | Chromo domain  Chromatin remodeling | Chromatin-associated |
| isotig05897 | methyl- binding domain protein 2 | MBD2 | ENSG00000134046 | SINFRUG00000147916 | Methyl-CpG-binding Chromatin remodeling | Chromatin-associated |
| isotig02509 | high-mobility group box 1 | HMGB1 | ENSG00000189403 | SINFRUG00000143203 | High mobility group, Chromatin remodeling | Chromatin-associated |
| isotig06656 | high-mobility group box 2 | HMGB2 | ENSG00000164104 | SINFRUG00000147395 | High mobility group, Chromatin remodeling | Chromatin-associated |
| isotig08462 | myeloid lymphoid or mixed-lineage leukemia 3 | MLL3 | ENSG00000055609 | SINFRUG00000148483 | Different Zinc finger and SET domains  Chromatin remodeling | Chromatin-associated |
| isotig10868 | jumonji domain containing 1b | JMJD1B | ENSG00000120733 | SINFRUG00000154744 | PHD zing finger  Chromatin remodeling | Chromatin-associated |
| isotig11923 | jumonji domain containing 2c | JMJD2C | ENSG00000107077 | SINFRUG00000142650 | PHD zing finger  Chromatin remodeling | Chromatin-associated |
| isotig12189 | bromodomain containing 4 | BRD4 | ENSG00000141867 | SINFRUG00000141925 | Bromo domain  Chromatin remodeling | Chromatin-associated |
| isotig13499 | set bifurcated 1 | SETDB1 | ENSG00000143379 | SINFRUG00000146805 | SET domain  Chromatin remodeling | Chromatin-associated |
| isotig14003 | mortality factor 4 like 1 | MORF4L1 | ENSG00000185787 |  | MRG family  Chromatin remodeling | Chromatin-associated |
| isotig14982 | yeats domain containing 4 | YEATS4 | ENSG00000127337 | SINFRUG00000149180 | YEAST domain  Chromatin remodeling | Chromatin-associated |
| isotig17241 | set domain containing 1a | SET1 | ENSG00000099381 | SINFRUG00000151955 | SET domain  Chromatin remodeling | Chromatin-associated |
| isotig18634 | chromodomain-helicase-dna-binding protein 9 | CHD9 | ENSG00000177200 |  | Chromo domain  Chromatin remodeling | Chromatin-associated |
| isotig20085 | chromodomain helicase dna binding protein 1 | CHD1 | ENSG00000153922 | SINFRUG00000140418 | Chromo domain  Chromatin remodeling | Chromatin-associated |
| isotig26524 | chromodomain helicase dna binding protein 3 | CHD3 | ENSG00000170004 | SINFRUG00000133175 | Chromo domain  Chromatin remodeling | Chromatin-associated |
| isotig27368 | myst histone acetyltransferase (monocytic leukemia) 4 | MYST4 | ENSG00000156650 | SINFRUG00000154304 | PHD-Zinc finger  Chromatin remodeling | Chromatin-associated |
| isotig29369 | histone-lysine n-methyltransferase mll4-like | MLL4 | ENSG00000105663 | SINFRUG00000154657 | Different Zinc finger and SET domains  Chromatin remodeling | Chromatin-associated |
| isotig29620 | histone-lysine n-methyltransferase ezh1-like | EZH1 | ENSG00000108799 | SINFRUG00000130595 | SET domain  Chromatin remodeling | Chromatin-associated |
| isotig30914 | probable global transcription activator snf2l2 | SMARCA2 | ENSG00000080503 | SINFRUG00000129403 | SWI/SNF family  Chromatin remodeling | Chromatin-associated |
| isotig31334 | jumonji domain containing 1c | JMJD1C | ENSG00000171988 | SINFRUG00000132919 | PHD zing finger  Chromatin remodeling | Chromatin-associated |
| isotig33747 | ash1 ( or homeotic)-like | ASH1L | ENSG00000116539 | SINFRUG00000155504 | PHD and SET domains  Chromatin remodeling | Chromatin-associated |
| isotig35265 | mortality factor 4-like protein 2 | MORF4L2 | ENSG00000123562 |  | MRG family  Chromatin remodeling | Chromatin-associated |
| isotig35748 | swi snf matrix actin dependent regulator of subfamily member 5 | SMARCA5 | ENSG00000153147 | SINFRUG00000142894 | SWI/SNF family  Chromatin remodeling | Chromatin-associated |
| isotig37826 | at rich interactive domain 1a | ARID1A | ENSG00000117713 | SINFRUG00000137226 | SWI/SNF family  Chromatin remodeling | Chromatin-associated |
| isotig39617 | bromodomain adjacent to zinc finger 1b | BAZ1B | ENSG00000009954 | SINFRUG00000139770 | PHD and Bromo domain  Chromatin remodeling | Chromatin-associated |
| contig09075 | pdz and lim domain 5 | PDLIM5 | ENSG00000163110 | SINFRUG00000137514 | LIM and PDZ domains  Co-activator of TFs | Cofactor |
| isotig01243 | mybbp1a protein | MYBBP1A | ENSG00000132382 | SINFRUG00000124434 | Co-activator of TFs | Cofactor |
| isotig02046 | e1a binding protein p300 | EP300 | ENSG00000100393 | SINFRUG00000140398 | Co-activator of TFs | Cofactor |
| isotig02592 | MSX2-spen transcriptional regulator | SPEN | ENSG00000065526 | SINFRUG00000124615 | Co-activator of TFs | Cofactor |
| isotig02641 | rrna promoter binding protein | TFB2M | ENSG00000162851 | SINFRUG00000150836 | Co-activator of TFs | Cofactor |
| isotig03593 | lim domain binding 1 | LDB1 | ENSG00000198728 | SINFRUG00000142748 | Co-activator of TFs | Cofactor |
| isotig05802 | activating transcription factor 7 interacting protein | ATF7IP | ENSG00000171681 | SINFRUG00000131479 | Co-activator of TFs | Cofactor |
| isotig06429 | nuclear receptor co-repressor 1 | NCOR1 | ENSG00000141027 | SINFRUG00000130052 | Co-activator of TFs | Cofactor |
| isotig07750 | histone deacetylase 4 | HDAC4 | ENSG00000068024 | SINFRUG00000124419 | Co-repressor of TFs | Cofactor |
| isotig09809 | helicase smarcad1 | SMARCAD1 | ENSG00000163104 | SINFRUG00000147781 | SWI/SNF related  Co-activator of TFs | Cofactor |
| isotig10170 | programmed cell death 11 | AHRR | ENSG00000063438 | SINFRUG00000153764 | Co-repressor of TFs | Cofactor |
| isotig10884 | otu domain containing 7b | ZA20D1 | ENSG00000163113 | SINFRUG00000131069 | Co-repressor of TFs | Cofactor |
| isotig10895 | cyclin-dependent kinase 4 | SERTAD1 | ENSG00000197019 |  | Co-activator of TFs | Cofactor |
| isotig10936 | k acetyltransferase 5 | HTATIP | ENSG00000172977 | SINFRUG00000150224 | Co-repressor of TFs | Cofactor |
| isotig11381 | nuclear receptor coactivator 2 | NCOA2 | ENSG00000140396 | SINFRUG00000143790 | Co-activator of TFs | Cofactor |
| isotig11768 | tata element modulatory factor 1 | TMF1 | ENSG00000144747 | SINFRUG00000121179 | - | Cofactor |
| isotig21616 | peroxisome proliferator-activated receptor coactivator 1 alpha-like | PPARGC1A | ENSG00000109819 | SINFRUG00000150714 | Co-activator of TFs | Cofactor |
| isotig24158 | nuclear receptor coactivator 4 | NCOA4 | ENSG00000138293 | SINFRUG00000132240 | Co-activator of TFs | Cofactor |
| isotig08250 | c-myc-binding protein | MYCBP | ENSG00000131233 |  | Co-activator of TFs | Cofactor |
| isotig26336 | thyroid hormone receptor interactor 11 | TRIP11 | ENSG00000100815 | SINFRUG00000132852 | Co-activator of TFs | Cofactor |
| isotig26921 | retinoblastoma-like protein 2 | RBL2 | ENSG00000103479 | SINFRUG00000152713 | Co-activator of TFs | Cofactor |
| isotig26942 | histone acetyltransferase 2 | MYST2 | ENSG00000136504 | SINFRUG00000132742 | Co-repressor of TFs  Chromatin remodeling | Cofactor |
| isotig27650 | dna-binding protein rfxank | RFXANK | ENSG00000064490 | SINFRUG00000150563 | Co-activator of TFs | Cofactor |
| isotig28212 | protein hira | HIRA | ENSG00000100084 | SINFRUG00000154061 | Co-repressor of TFs  Chromatin remodeling | Cofactor |
| isotig28876 | lim domain-binding protein 2 | LDB2 | ENSG00000169744 | SINFRUG00000163364 | Co-activator of TFs | Cofactor |
| isotig32495 | catenin beta-1 | CTNNB1 | ENSG00000168036 | SINFRUG00000128607 | - | Cofactor |
| isotig06257 | cop9 signalosome complex subunit 5 | COPS5 | ENSG00000121022 | SINFRUG00000146075 | Co-activator of TFs | Cofactor |
| isotig34079 | histone deacetylase 1 | HDAC1 | ENSG00000116478 | SINFRUG00000129449 | Chromatin remodeling | Cofactor |
| isotig36690 | thyroid hormone receptor associated protein 3 | THRAP3 | ENSG00000054118 | SINFRUG00000143511 | Co-activator of TFs | Cofactor |
| isotig02407 | cullin-associated nedd8-dissociated protein 1 | CAND1 | ENSG00000111530 | SINFRUG00000129505 | Co-activator of TFs | Cofactor |
| isotig05877 | sap30 binding protein | SAP30BP | ENSG00000164105 |  | Co-repressor of TFs | Cofactor |
| isotig42356 | yy1 associated factor 2 | YAF2 | ENSG00000015153 | SINFRUG00000164945 | Co-activator of TFs | Cofactor |
| isotig04376 | smad family member 2 | SMAD2 | ENSG00000175387 | SINFRUG00000152596 | MH1  Dwarfin/SMAD | Dwarfin |
| isotig16816 | smad family member 1 | SMAD1 | ENSG00000170365 | SINFRUG00000126514 | MH1  Dwarfin/SMAD | Dwarfin |
| isotig19004 | mothers against decapentaplegic homolog 4 | SMAD4 | ENSG00000141646 | SINFRUG00000125932 | MH1  Dwarfin/SMAD | Dwarfin |
| isotig08286 | e2f transcription factor 6 | E2F6 | ENSG00000169016 | SINFRUG00000124257 | E2F family | E2F |
| isotig10706 | transcription factor dp-1 | TFDP1 | ENSG00000198176 | SINFRUG00000130767 | E2F family | E2F |
| isotig05470 | forkhead box o3 | FOXO3A | ENSG00000118689 | SINFRUG00000135609 | Forkhead family | Forkhead |
| isotig17303 | forkhead box k2 | FOXK2 | ENSG00000141568 | SINFRUG00000137794 | Forkhead family | Forkhead |
| isotig24339 | forkhead box j3 | FOXJ3 | ENSG00000198815 | SINFRUG00000138893 | Forkhead family | Forkhead |
| isotig32310 | forkhead box protein p1-b | FOXP1 | ENSG00000114861 | SINFRUG00000142067 | Forkhead family | Forkhead |
| isotig02366 | basic transcription factor 3 | BTF3 | ENSG00000145741 | SINFRUG00000146164 | NAC-beta family | General transcription factor |
| isotig02496 | transcription initiation factor iia subunit 2 | GTF2A1 | ENSG00000165417 | SINFRUG00000137360 | TFIIA family | General transcription factor |
| isotig02611 | transcription initiation factor tfiid subunit 7 | TAF7 | ENSG00000178913 | SINFRUG00000150296 | TAFH family | General transcription factor |
| isotig03290 | tata box-binding protein-associated factor rna polymerase i subunit b | TAF1B | ENSG00000115750 | SINFRUG00000165198 | TAFH family | General transcription factor |
| isotig04901 | transcription initiation factor tfiid subunit 10 | TAF10 | ENSG00000166337 | SINFRUG00000138771 | TAFH family | General transcription factor |
| isotig04951 | transcription initiation factor tfiid subunit 13 | TAF13 | ENSG00000197780 |  | TAFH family | General transcription factor |
| isotig05078 | general transcription factor iif subunit 1 | GTF2F1 | ENSG00000125651 | SINFRUG00000160254 | TFIIA family | General transcription factor |
| isotig06322 | transcription initiation factor tfiid subunit 12 | TAF12 | ENSG00000120656 | SINFRUG00000127610 | TAFH family | General transcription factor |
| isotig07251 | transcription initiation factor tfiid subunit 9 | TAF9 | ENSG00000085231 | SINFRUG00000163833 | TAFH family | General transcription factor |
| isotig07751 | taf1 rna polymerase tata box binding protein -associated factor | TAF1 | ENSG00000147133 | SINFRUG00000163082 | TAFH family | General transcription factor |
| isotig07759 | general transcription factor polypeptide 34kda | GTF2H3 | ENSG00000111358 | SINFRUG00000121107 | TFB4 family | General transcription factor |
| isotig09046 | tata-binding protein-associated factor 172 | BTAF1 | ENSG00000095564 | SINFRUG00000135604 | SNF2/RAD54 family | General transcription factor |
| isotig09313 | general transcription factor iii c isoform cra_b | GTF3C5 | ENSG00000148308 | SINFRUG00000131172 | TFIIIC family | General transcription factor |
| isotig09664 | tfiih basal transcription factor complex helicase xpb subunit | ERCC2 | ENSG00000104884 | SINFRUG00000141597 | RAD3/XPD family | General transcription factor |
| isotig10156 | activated rna polymerase ii transcriptional coactivator p15 | SUB1 | ENSG00000113387 | SINFRUG00000123340 | PC4 co-activator family | Cofactor |
| isotig10840 | general transcription factor 3c polypeptide 6 | GTF3C6 | ENSG00000155115 |  | TFIIIC family | General transcription factor |
| isotig13153 | transcription initiation factor tfiid subunit 11 | TAF11 | ENSG00000064995 | SINFRUG00000141782 | TAFH family | General transcription factor |
| isotig16977 | transcription factor iib | GTF2B | ENSG00000137947 | SINFRUG00000128194 | TFIIB family | General transcription factor |
| isotig28930 | general transcription factor polypeptide 62kda | GTF2H1 | ENSG00000110768 | SINFRUG00000133718 | TFIIB family | General transcription factor |
| isotig30389 | general transcription factor polypeptide 30kda | GTF2F2 | ENSG00000188342 | SINFRUG00000164075 | TFIIB family | General transcription factor |
| isotig05735 | ubtf protein | UBTF | ENSG00000108312 | SINFRUG00000164124 | HMG-box family | High mobility group box |
| isotig02261 | bbx protein | BBX | ENSG00000114439 |  | HMG-box family | High mobility group box |
| isotig05333 | transcription factor mitochondrial precursor | TFAM | ENSG00000108064 |  | HMG-box family | High mobility group box |
| isotig07697 | transcription factor sox-6 isoform 2 | SOX6.2 | ENSG00000110693 | SINFRUG00000134729 | HMG-box family | High mobility group box |
| isotig13429 | transcription factor 7-like 2 (t-cell hmg-box) | TCF7L2 | ENSG00000148737 | SINFRUG00000148340 | HMG-box family | High mobility group box |
| isotig02099 | set and mynd domain-containing protein 1 | SMYD1 |  |  | Mynd-type zinc finger and SET domains | Znf-Others |
| isotig25230 | transcription factor sox-8 | SOX8 | ENSG00000005513 | SINFRUG00000120942 | HMG-box family | High mobility group box |
| isotig27657 | high-mobility group 20a | HMG20A | ENSG00000140382 | SINFRUG00000126076 | HMG-box family | High mobility group box |
| isotig33307 | myeloid lymphoid or mixed-lineage leukemia 2 | MLL2 | ENSG00000167548 | SINFRUG00000149186 | SET, PHD and Ring-zinc finger domains | Znf-Others |
| isotig39477 | transcription factor 7-like 1a | TCF7L1 | ENSG00000152284 | SINFRUG00000140510 | TCF/LEF  HMG-box family | High mobility group box |
| isotig02235 | six homeobox 1 | SIX1 | ENSG00000126778 | SINFRUG00000120390 | SIX Homeobox family | Homeobox |
| isotig02236 | six homeobox 2 | SIX2 | ENSG00000170577 | SINFRUG00000147598 | SIX Homeobox family | Homeobox |
| isotig05929 | pre-b-cell leukemia transcription factor 3-like | PBX3 | ENSG00000167081 | SINFRUG00000128802 | TALE/PBX Homeobox family | Homeobox |
| isotig09143 | pou class transcription factor 3 | POU1F1 | ENSG00000064835 | SINFRUG00000137354 | POU-Homeobox family | Homeobox |
| isotig09756 | pre-b-cell leukemia transcription factor 4 | PBX4 | ENSG00000105717 | SINFRUG00000141922 | TALE/PBX Homeobox family | Homeobox |
| isotig10829 | zinc finger homeobox protein 2 | ZFHX2 | ENSG00000136367 |  | Homeobox family | Homeobox |
| isotig11574 | homeobox a9 | HOXA9 | ENSG00000106038 | SINFRUG00000165271 | Abd-B-Homeobox family | Homeobox |
| isotig12426 | zinc fingers and homeoboxes 3 | ZHX3 | ENSG00000174306 | SINFRUG00000153537 | ZHX family  Zinc-finger and Homeobox domains | Homeobox |
| isotig17443 | homeobox protein 9aa | LHX9 | ENSG00000143355 | SINFRUG00000120406 | LIM-Homeobox domains | Homeobox |
| isotig18124 | pou class transcription factor 3 | POU3F1 | ENSG00000185668 | SINFRUG00000144429 | POU family  POU and Homeobox domains | Homeobox |
| isotig19096 | cut-like homeobox 1 | CUTL1 | ENSG00000160967 | SINFRUG00000132606 | CUT-Homeobox family | Homeobox |
| isotig26404 | homeobox protein 10ab | B3SU79_SALSA |  |  | Homeobox domain | Homeobox |
| isotig29933 | cut-like 2 | CULT2 | ENSG00000160967 | SINFRUG00000132606 | CUT-Homeobox family | Homeobox |
| isotig33951 | homeodomain interacting protein kinase 3 | HIPK3 | ENSG00000110422 |  | Co-activator of TFs | Cofactor |
| isotig04248 | mineralocorticoid receptor | NR3C2 | ENSG00000151623 | SINFRUG00000122938 | Nuclear Receptor Famliy | Nuclear hormone receptor |
| isotig04276 | nuclear receptor subfamily 4 group a member 1 | NR4A1 | ENSG00000123358 | SINFRUG00000123526 | Nuclear Receptor Famliy | Nuclear hormone receptor |
| isotig05196 | retinoid x receptor beta | RXRB | ENSG00000112472 | SINFRUG00000129871 | Nuclear Receptor Famliy | Nuclear hormone receptor |
| isotig05601 | retinoid x gamma | RXRG | ENSG00000162761 | SINFRUG00000123103 | Nuclear Receptor Famliy | Nuclear hormone receptor |
| isotig05816 | nuclear receptor subfamily A group member 2 | NR1D2 | ENSG00000174738 | SINFRUG00000121364 | Nuclear Receptor Famliy | Nuclear hormone receptor |
| isotig08801 | Peroxisome proliferator-activated receptor alpha | PPARA | ENSG00000186951 | SINFRUG00000144605 | Nuclear Receptor Famliy | Nuclear hormone receptor |
| isotig09781 | rar-related orphan receptor b | RORB | ENSG00000198963 | SINFRUG00000163719 | Nuclear Receptor Famliy | Nuclear hormone receptor |
| isotig12645 | peroxisome proliferator-activated receptor gamma | PPARG | ENSG00000132170 | SINFRUG00000160781 | Nuclear Receptor Famliy | Nuclear hormone receptor |
| isotig13123 | androgen receptor alpha | AR | ENSG00000169083 | SINFRUG00000147103 | Nuclear Receptor Famliy | Nuclear hormone receptor |
| isotig14320 | retinoic acid receptor gamma | RARG | ENSG00000172819 | SINFRUG00000149355 | Nuclear Receptor Famliy | Nuclear hormone receptor |
| isotig14668 | rar-related orphan receptor a | RORA | ENSG00000069667 | SINFRUG00000139635 | Nuclear Receptor Famliy | Nuclear hormone receptor |
| isotig17456 | oxysterols receptor lxr-alpha | NR1H3 | ENSG00000025434 | SINFRUG00000129321 | Nuclear Receptor Famliy | Nuclear hormone receptor |
| isotig25523 | nuclear receptor subfamily 4 group a member 2 | NR4A2 | ENSG00000153234 | SINFRUG00000136739 | Nuclear Receptor Famliy | Nuclear hormone receptor |
| isotig25754 | nuclear receptor subfamily 2 group c member 2-like | NR2C2 | ENSG00000177463 | SINFRUG00000125129 | Nuclear Receptor Famliy | Nuclear hormone receptor |
| isotig34054 | retinoic acid receptor alpha | RARA | ENSG00000131759 | SINFRUG00000142757 | Nuclear Receptor Famliy | Nuclear hormone receptor |
| isotig01991 | tar dna-binding protein 43 | TARDBP | ENSG00000120948 | SINFRUG00000137736 | RRM DNA-binding domain | Others |
| isotig04421 | tubby like protein 4 | TULP4 | ENSG00000130338 | SINFRUG00000145580 | TUB-SOCS | Others |
| isotig05312 | prolactin regulatory element-binding protein | PREB | ENSG00000138073 | SINFRUG00000146057 | WD repeats | Others |
| isotig06004 | pr domain containing with znf domain | PRDM1 | ENSG00000057657 | SINFRUG00000134881 | ZNF-C2H2 & SET-domains | Others |
| isotig09148 | purine-rich element binding protein a | PURA | ENSG00000185129 | SINFRUG00000151348 | PUR-DNA binding | Others |
| isotig01734 | upstream binding protein 1 | FUBP1 | ENSG00000162613 | SINFRUG00000160897 | KH domains | Others |
| isotig09476 | nfkb repressing factor | NKRF | ENSG00000186416 | SINFRUG00000122216 | Co-activator of TFs | Others |
| isotig09903 | bromodomain adjacent to zinc finger 2b | BAZ2B | ENSG00000123636 | SINFRUG00000144665 | WAL family  PHD, MBD,DDT domains | Others |
| isotig26602 | recombination signal binding protein for immunoglobulin kappa j region b | RBPSUH | ENSG00000168214 | SINFRUG00000162697 | Su(H) family | Others |
| isotig29807 | snf2-related cbp activator protein | SRCAP | ENSG00000080603 | SINFRUG00000132728 | SNF2/RAD54 | Others |
| isotig32153 | hematopoietic lineage cell-specific protein | HCLS1 | ENSG00000180353 | SINFRUG00000156355 | SH3 domain | Others |
| isotig10901 | p300 cbp-associated factor | CITED1 | ENSG00000125931 | SINFRUG00000160462 | CITED family | Others |
| isotig33673 | ccr4-not transcription subunit 3 | CNOT3 | ENSG00000088038 | SINFRUG00000131108 | CNOT family | Others |
| isotig36591 | bromodomain adjacent to zinc finger 2a | BAZ2A | ENSG00000076108 | SINFRUG00000121094 | WAL family  PHD, MBD,DDT domains | Others |
| isotig39177 | eyes absent homolog 1 | EYA1 | ENSG00000104313 | SINFRUG00000142160 | HAD-like family | Others |
| isotig42179 | ccr4-not transcription subunit 8 | CNOT8 | ENSG00000155508 | SINFRUG00000124692 | CAF1 family | General Transcription Factor |
| isotig06724 | pre-b-cell leukemia homeobox 1 | PBX1 | ENSG00000185630 | SINFRUG00000128790 | TALE/PBX family Homeobox domain | Homeobox |
| isotig04015 | creb binding protein | CREBBP | ENSG00000005339 | SINFRUG00000160816 | Co-activator of TF | Cofactor |
| isotig04154 | midline 1 (opitz bbb syndrome) | MID1 | ENSG00000101871 | SINFRUG00000153402 | TRIM family | Protein-protein interaction domains |
| isotig04662 | zinc finger and btb domain-containing protein 49 | ZBTB49 (ZNF509) | ENSG00000168826 | SINFRUG00000131011 | ZnF-C2H2 family | Znf-C2H2 |
| isotig05371 | kelch repeat and btb domain containing 5 | KLHDC5 | ENSG00000087448 | SINFRUG00000155427 | BTB-POZ domain  Kelch repeat domain | Protein-protein interaction domains |
| isotig06447 | kelch-like 12 | KLHL12 | ENSG00000117153 | SINFRUG00000165201 | BTB-POZ domain | Protein-protein interaction domains |
| isotig07825 | transcription factor 20-like | TCF20 | ENSG00000100207 | SINFRUG00000139283 | AT-box and PHD-Zinc finger domains | Protein-protein interaction domains |
| isotig08345 | kelch repeat and btb domain containing 10 | KBTBD10 | ENSG00000154474 | SINFRUG00000140602 | BTB-POZ domain  Kelch repeat domain | Protein-protein interaction domains |
| isotig03735 | endoplasmic reticulum to nucleus signalling 1 | BTBD14B | ENSG00000160877 | SINFRUG00000153352 | BTB-POZ domain | Protein-protein interaction domains |
| isotig08537 | kelch-like 21 | KLHL21 | ENSG00000162413 | SINFRUG00000127016 | BTB-POZ domain  Kelch repeat domain | Protein-protein interaction domains |
| isotig10390 | bromodomain phd finger transcription factor | BPTF (FALZ) | ENSG00000171634 | SINFRUG00000163418 | PBTF family  Chromatin remodeling | Protein-protein interaction domains |
| isotig10434 | kelch-like 3 | KLHL3 | ENSG00000146021 | SINFRUG00000145412 | BTB-POZ domain  Kelch repeat domain | Protein-protein interaction domains |
| isotig11155 | zinc finger and btb domain containing 41 | ZBTB41 | ENSG00000177888 |  | BTB-POZ and ZnF-C2H2 type domiains | Protein-protein interaction domains |
| isotig12015 | zinc finger and btb domain containing 2 | ZBTB2 | ENSG00000181472 | SINFRUG00000135353 | BTB-POZ and ZnF-C2H2 type domiains | Protein-protein interaction domains |
| isotig13205 | zinc finger and btb domain containing 26 | ZBTB26 | ENSG00000171448 | SINFRUG00000134883 | BTB-POZ and ZnF-C2H2 type domiains | Protein-protein interaction domains |
| isotig14345 | hypermethylated in cancer 1 | HIC1 | ENSG00000177374 | SINFRUG00000153139 | ZnF-C2H2 family | Znf-C2H2 |
| isotig15004 | zinc finger and btb domain containing 33 | ZBTB33 | ENSG00000177485 | SINFRUG00000156158 | BTB-POZ and ZnF-C2H2 type domiains | Protein-protein interaction domains |
| isotig15308 | zinc finger protein 161 homolog | ZFP161 | ENSG00000198081 | SINFRUG00000142713 | ZnF-C2H2 family | ZnF-C2H2 family |
| isotig21762 | midline 1 (opitz bbb syndrome) isoform 2 | MID1_2 | ENSG00000101871 | SINFRUG00000153402 | TRIM family | Protein-protein interaction domains |
| isotig27678 | zinc finger protein 521 | ZNF521 | ENSG00000198795 | SINFRUG00000155245 | ZnF-C2H2 family | ZnF-C2H2 family |
| isotig32114 | zinc finger and btb domain containing 3 | ZBTB3 | ENSG00000185670 |  | BTB-POZ and ZnF-C2H2 type domains | Protein-protein interaction domains |
| isotig32132 | zinc and double phd fingers family 2 | DPF2 | ENSG00000133884 | SINFRUG00000149887 | Requiem/DPF family | Protein-protein interaction domains |
| isotig06019 | nfx1-type zinc finger-containing protein 1 | NFX1 | ENSG00000086102 | SINFRUG00000134538 | NFX zinc finger family | RFX/others |
| isotig05628 | tea domain family member 4 | TEAD4 | ENSG00000197905 |  | TEA family | TEA |
| isotig07180 | transcriptional enhancer factor tef-5-like | TEAD5 | ENSG00000007866 | SINFRUG00000153549 | TEA family | TEA |
| isotig18924 | tea domain family member 3 | TEAD3 | ENSG00000007866 | SINFRUG00000153549 | TEA family | TEA |
| isotig17721 | ets2 repressor factor | ERF | ENSG00000105722 | SINFRUG00000144886 | ETS famitly | Trp cluster – Ets |
| isotig28110 | ga binding protein transcription alpha subunit 60kda | GABPA | ENSG00000154727 | SINFRUG00000150275 | ETS famitly | Trp cluster – Ets |
| isotig29147 | friend leukemia integration 1 | FLI1 | ENSG00000134954 | SINFRUG00000153673 | ETS famitly | Trp cluster – Ets |
| isotig07160 | interferon regulatory factor 2 | IRF2 | ENSG00000168310 | SINFRUG00000159812 | IRF family | Trp cluster – IRF |
| isotig17546 | interferon regulatory factor 3 | IRF3 | ENSG00000126456 | SINFRUG00000129862 | IRF family | Trp cluster – IRF |
| isotig20053 | ring finger protein 31 | RNF3/ISGF3G | ENSG00000092098 | SINFRUG00000121570 | Znf-finger | Trp cluster – IRF |
| isotig25256 | interferon regulatory factor 5 | IRF5 | ENSG00000128604 | SINFRUG00000142972 | IRF family | Trp cluster – IRF |
| isotig09701 | mesoderm induction early response family member 3 | MIER3 | ENSG00000155545 | SINFRUG00000145798 | ELM2 domain | Trp cluster - Myb |
| isotig32099 | e1a binding protein p400 | EP400 | ENSG00000183495 | SINFRUG00000122028 | SNF2/RAD54 family | Trp cluster - Myb |
| isotig21261 | histone h4 transcription factor | HINFP | ENSG00000172273 |  | ZnF-C2H2 domains | ZnF-C2H2 |
| isotig02899 | zinc finger protein 106 homolog | ZFP106 | ENSG00000103994 | SINFRUG00000148889 | ZnF-C2H2 domains | ZnF-C2H2 |
| isotig03797 | zinc finger protein 143 | ZNF143 | ENSG00000166478 | SINFRUG00000128865 | GLI-ZnF-C2H2 family | ZnF-C2H2 |
| isotig04083 | zinc finger protein y | ZFY | ENSG00000067646 |  | ZnF-C2H2 family | ZnF-C2H2 |
| isotig04084 | zinc finger protein zfx | ZFX | ENSG00000005889 | SINFRUG00000149959 | ZnF-C2H2 family | ZnF-C2H2 |
| isotig04117 | vascular endothelial zinc finger 1 | ZNF161/VEZF1 | ENSG00000136451 | SINFRUG00000142336 | ZnF-C2H2 family | ZnF-C2H2 |
| isotig04120 | interleukin enhancer-binding factor 3 | ILF3 | ENSG00000129351 | SINFRUG00000144346 | DRBM-DZF domains | Others |
| isotig04663 | zinc finger protein 146 | ZNF146 | ENSG00000167635 |  | ZnF-C2H2 family | ZnF-C2H2 |
| isotig05846 | early growth response 1 | EGR1 | ENSG00000120738 | SINFRUG00000154741 | EGR-ZnF-C2H2 family | ZnF-C2H2 |
| isotig06367 | ccctc-binding factor (zinc finger protein) | CTCF | ENSG00000102974 | SINFRUG00000147322 | CTCF-zing finger family | ZnF-C2H2 |
| isotig06421 | rearranged l-myc fusion | RLF | ENSG00000117000 | SINFRUG00000158918 | ZnF-C2H2 | ZnF-C2H2 |
| isotig06924 | zinc finger protein 706 | ZNF706 | ENSG00000120963 | SINFRUG00000154283 | ZnF-C2H2 type domain | ZnF-C2H2 |
| isotig07124 | kruppel-like factor 10 | KLF10 | ENSG00000155090 | SINFRUG00000139409 | ZnF-C2H2 family | ZnF-C2H2 |
| isotig07510 | zinc finger protein 2 | ZNF2 | ENSG00000163067 |  | ZnF-C2H2 family | ZnF-C2H2 |
| isotig07531 | zinc finger protein 576 | ZNF576 | ENSG00000124444 |  | ZnF-C2H2 family | ZnF-C2H2 |
| isotig07644 | zinc finger homeobox protein 3-like | ATBF1 | ENSG00000140836 | SINFRUG00000135959 | ZnF-C2H2 family | ZnF-C2H2 |
| isotig08594 | zinc finger protein 593 | ZNF593 | ENSG00000142684 | SINFRUG00000147877 | ZnF-C2H2 family | ZnF-C2H2 |
| isotig08928 | krueppel-like factor 6 | KLF6 | ENSG00000067082 | SINFRUG00000139622 | ZnF-C2H2 family | ZnF-C2H2 |
| isotig08957 | castor zinc finger 1 | CASZ1 | ENSG00000130940 | SINFRUG00000162856 | ZnF-C2H2 family | ZnF-C2H2 |
| isotig09210 | zinc finger homeobox 4 | ZFHX4 | ENSG00000091656 | SINFRUG00000141224 | ZnF-C2H2 family | ZnF-C2H2 |
| isotig09325 | zinc finger protein 236 | ZNF236 | ENSG00000130856 | SINFRUG00000141202 | ZnF-C2H2 family | ZnF-C2H2 |
| isotig09336 | zinc finger protein 53 | ZNF277 | ENSG00000198839 | SINFRUG00000155524 | ZNF277 family  ZnF-C2H2 type domain | ZnF-C2H2 |
| isotig10408 | gdnf-inducible zinc finger protein 1 | ZNF336/GZF1 | ENSG00000125812 | SINFRUG00000143155 | ZnF-C2H2 family | ZnF-C2H2 |
| isotig10551 | trafficking protein particle complex subunit 2 | ZNF547 | ENSG00000152433 |  | ZnF-C2H2 family | ZnF-C2H2 |
| isotig10710 | re1-silencing transcription factor | REST | ENSG00000084093 | SINFRUG00000140216 | ZnF-C2H2 type domain | ZnF-C2H2 |
| isotig11588 | zinc finger protein 131 | ZNF131 | ENSG00000172262 |  | ZnF-C2H2 family | ZnF-C2H2 |
| isotig12031 | zinc finger protein 287 | ZNF287 | ENSG00000141040 |  | ZnF-C2H2 family | ZnF-C2H2 |
| isotig14391 | zinc finger protein 33a | ZNF33A | ENSG00000189180 |  | ZnF-C2H2 family | ZnF-C2H2 |
| isotig15285 | cdkn1a interacting zinc finger protein 1 | CIZ1 | ENSG00000148337 | SINFRUG00000130135 | Zinc finger domain | ZnF-C2H2 |
| isotig15584 | zinc finger protein 574 | ZNF574 | ENSG00000105732 | SINFRUG00000123771 | ZnF-C2H2 family | ZnF-C2H2 |
| isotig16346 | zinc finger protein 45 | ZNF45 | ENSG00000124459 |  | ZnF-C2H2 family | ZnF-C2H2 |
| isotig16777 | zinc finger e-box-binding homeobox 2-like isoform 1 | NIL-2-A | ENSG00000148516 | SINFRUG00000137753 | ZnF-C2H2 type family | ZnF-C2H2 |
| isotig16797 | e4f transcription factor 1 | E4F1 | ENSG00000167967 | SINFRUG00000133288 | ZnF-C2H2 type domain | ZnF-C2H2 |
| isotig17124 | kruppel-like factor 4 | KLF4 | ENSG00000136826 | SINFRUG00000124975 | ZnF-C2H2 family | ZnF-C2H2 |
| isotig18664 | krueppel-like factor 2 | KLF2 | ENSG00000127528 | SINFRUG00000136204 | ZnF-C2H2 family | ZnF-C2H2 |
| isotig20411 | zinc finger protein 395 | ZNF395 | ENSG00000186918 | SINFRUG00000151266 | ZnF-C2H2 type domain | ZnF-C2H2 |
| isotig20601 | zinc finger protein 214 | ZNF214 | ENSG00000149050 |  | ZnF-C2H2 family | ZnF-C2H2 |
| isotig21017 | zinc finger protein 207 | ZNF207 | ENSG00000010244 | SINFRUG00000147681 | ZnF-C2H2 type domain | ZnF-C2H2 |
| isotig22693 | human immunodeficiency virus type i enhancer binding protein 2 | HIVEP2 | ENSG00000010818 | SINFRUG00000124252 | ZnF-C2H2 type domain | ZnF-C2H2 |
| isotig25043 | zinc finger protein 335 | ZNF335 | ENSG00000198026 | SINFRUG00000145290 | ZnF-C2H2 family | ZnF-C2H2 |
| isotig26679 | glis family zinc finger 2 | GLIS2 | ENSG00000126603 | SINFRUG00000138980 | ZnF-C2H2 family | ZnF-C2H2 |
| isotig26847 | kruppel-like factor 12 | KLF12 | ENSG00000118922 | SINFRUG00000145593 | ZnF-C2H2 family | ZnF-C2H2 |
| isotig26995 | b-cell lymphoma 6 protein | BCL6 | ENSG00000113916 | SINFRUG00000122062 | BTB-POZ domain  ZnF-C2H2 domain | ZnF-C2H2 |
| isotig28729 | zinc finger protein 551 | ZNF551 | SINFRUG00000132728 | | ZnF-C2H2 family | ZnF-C2H2 |
| isotig30737 | b-cell lymphoma 6 protein isoform 2 | BCL6_2 | ENSG00000113916 | SINFRUG00000122062 | BTB-POZ domain  ZnF-C2H2 domain | ZnF-C2H2 |
| isotig32333 | sp3 transcription factor | SP3 | ENSG00000172845 | SINFRUG00000140872 | ZnF-C2H2 family | ZnF-C2H2 |
| isotig33163 | zinc finger protein 22 | ZNF22 | ENSG00000165512 |  | ZnF-C2H2 family | ZnF-C2H2 |
| isotig35744 | snail homolog 2 | SNAI2 | ENSG00000019549 | SINFRUG00000122750 | ZnF-C2H2 family | ZnF-C2H2 |
| isotig37548 | zinc finger protein 347-like | ZNF347 | ENSG00000197937 |  | ZnF-C2H2 family | ZnF-C2H2 |
| isotig40508 | gli-kruppel family member gli3 | GLI3 | ENSG00000106571 | SINFRUG00000153715 | ZnF-C2H2 family | ZnF-C2H2 |
| isotig42157 | zinc finger protein 711 | ZNF6 | ENSG00000147180 | SINFRUG00000160137 | ZnF-C2H2 family | ZnF-C2H2 |
| isotig09696 | zinc finger protein c3h type-like 2 | ZFP36L2 | ENSG00000152518 | SINFRUG00000132508 | C3H1-Zinc finger | ZnF-C3H |
| isotig18315 | gata zinc finger domain containing 1 | GATAD1 | ENSG00000157259 | SINFRUG00000154644 | GATA-type zinc finger domain | ZnF-GATA |
| isotig21584 | metastasis-associated protein mta3 | MTA3 | ENSG00000057935 |  | GATA-type zinc finger domain | ZnF-GATA |
| isotig29975 | arginine glutamic acid dipeptide repeats | RERE | ENSG00000142599 | SINFRUG00000127307 | GATA-type zinc finger domain | ZnF-GATA |
| isotig01753 | zinc an1-type domain 5 | ZA20D2 | ENSG00000107372 | SINFRUG00000124587 | A20-AN1 zinc fingers | ZnF-Others |
| isotig03816 | nuclear factor 1 x-type-like | NFXL1 | ENSG00000170448 |  | NFX zinc finger family | ZnF-Others |
| isotig04260 | four and a half lim domains protein 1 | FHL1 | ENSG00000022267 | SINFRUG00000150293 | LIM-zinc finger domain | ZnF-Others |
| isotig06419 | glucocorticoid receptor dna-binding factor 1 | GRLF1 | ENSG00000160007 | SINFRUG00000150833 | Rho-GAP domain  Bind glucocorticoid receptor | ZnF-Others |
| isotig08651 | lim domain only 4 | LMO4 | ENSG00000143013 | SINFRUG00000157609 | LIM-zinc finger domain | ZnF-Others |
| isotig11053 | zinc finger protein 596 | ZNF596 | ENSG00000172748 |  | ZnF-C2H2 family | ZnF-Others |
| isotig11113 | lim domain 7 | LMO7 | ENSG00000136153 | SINFRUG00000135018 | LIM-zinc finger domain | ZnF-Others |
| isotig17183 | four and a half lim domains 2 | FHL2 | ENSG00000115641 | SINFRUG00000159858 | LIM-zinc finger domain | ZnF-Others |
| isotig26287 | immunoglobulin mu binding protein 2 | IGHMBP2 | ENSG00000132740 | SINFRUG00000123531 | AN1-zinc finger domain | ZnF-Others |
| isotig04812 | cellular nucleic acid-binding protein | ZNF9 | ENSG00000169714 | SINFRUG00000126211 | CCHC-zinc finger domain | ZnF-Others |
