# Supplementary material for: Fast skeletal muscle transcriptome of the Gilthead sea bream (Sparus aurata) determined by next generation sequencing
Source: BMC Genomics. 2012 May 11;13:181. doi: 10.1186/1471-2164-13-181 (PMC3418159; doi:10.1186/1471-2164-13-181)
Supplement: Additional file 15 — The 20 most abundant isotigs from gilthead sea bream partial assemblies. Abundant isotigs were defined as the ones with the highest number of reads. Name in red are genes found in all conditions top 20. Name in blue are genes found in only one condition. [file 1471-2164-13-181-S15.docx]

*Supplementary Table 4*. The 20 most abundant isotigs from gilthead sea bream partial assemblies. Abundant isotigs are defined as the ones with higher number of reads.

| *Experimental Condition* | Fed | Fasted | High Temperature | Low Temperature | Adult |
| --- | --- | --- | --- | --- | --- |
|  |  |  |  |  |  |
| *Gene ID* | Phosphoglucomutase-1 | Phosphoglucomutase-1 | Phosphoglucomutase-1 | Phosphoglucomutase-1 | Sarcoplasmatic reticulum calcium atpase 1 |
|  | Phosphoglucose isomerase 2 | Adenylate kinase 1 | Cyclin g1 | Parvalbumin | Cyclin g1 |
|  | Calsequestrin 1 | Sarcoplasmatic reticulum calcium atpase 1 | Elongation factor 1 alpha | Myosin light chain 1 | Nucleoside diphosphate kinase b |
|  | Elongation factor 1 alpha | Parvalbumin | Solute carrier family 25 member 4 | Beta-enolase | Glycogen muscle form |
|  | Alpha 3 | Heat shock protein 90 alpha | Glycogen muscle form | Glycogen phosphorilase | Elongation factor 1 alpha |
|  | Phosphoglycerate kinase 1 | Elongation factor 1 alpha | Sarcoplasmatic reticulum calcium atpase 1 | Triosephosphate isomerase b | Alpha 3 |
|  | Glycogen muscle form | Phosphoglucose isomerase 2 | Triosephosphate isomerase b | Solute carrier family 25 member 4 | Myosin binding protein fast type |
|  | Myosin binding protein fast type | Triosephosphate isomerase b | Tropomyosin 3 | Lactate dehydrogenase a | Calsequestrin 1 |
|  | Sarcoplasmatic reticulum calcium atpase 1 | Glycogen muscle form | Parvalbumin | Tropomyosin 3 | Parvalbumin |
|  | Cyclin g1 | Lactate dehydrogenase a | Myosin binding protein fast type | Glyceraldehyde 3-phosphate dehydrogenase | Glyceraldehyde 3-phosphate dehydrogenase |
|  | Adenosine monophosphate deaminase 1 | Beta-enolase | Phosphoglucose isomerase 2 | Calsequestrin 1 | Phosphoglycerate kinase 1 |
|  | Adp atp translocase 2 | Glycerol 3 phosphate dehydrogenase | Tropomysin 1 | Cyclin g1 | Phosphoglucose isomerase 2 |
|  | Pyruvate kinase | Adenosine monophosphate deaminase 1 | Alpha actin | Phosphoglucose isomerase 2 | Myosin light chain 1 |
|  | Lactate dehydrogenase a | Cyclin g1 | Myosin light chain 1 | Alpha actin | Pyruvate kinase |
|  | Phosphoglucomutase-2 | Solute carrier family 25 member 4 | Adenosine monophosphate deaminase 1 | Troponin skeletal muscle | Adenosine monophosphate deaminase 1 |
|  | Parvalbumin | Adp atp translocase 1 | Calsequestrin 1 | Nuceloside diphosphatase kinase b | Tropomyosin 1 |
|  | Glycerol 3-phosphate dehydrogenase | Ubiquitin specific protease 25 | Heat shock protein 70 | Elongation factor alpha 1 | Phosphoglycerate mutase 2 |
|  | Myosin light chain 2 | Calsequestrin 1 | Heat shock protein 90 alpha | Adenosine monophosphate deaminase 1 | Alpha actin |
|  | Heat shock protein 8 | Alpha actin | Troponin skeletal muscle | Pyruvate kinase | Lactate dehydrogenase a |
|  | Nucleoside diphosphate kinase b | Phosphoglycerate kinase 1 | Phosphofructokinase muscle type | Nadh dehydrogenase subunit 1 | Tropomyosin 3 |

Name in red are genes found in all conditions top 20

Name in blue are genes found in only one condition
