# Supplementary material for: Fast skeletal muscle transcriptome of the Gilthead sea bream (Sparus aurata) determined by next generation sequencing
Source: BMC Genomics. 2012 May 11;13:181. doi: 10.1186/1471-2164-13-181 (PMC3418159; doi:10.1186/1471-2164-13-181)
Supplement: Additional file 16 — Adapters and abundant genes sequences used for assembly trimming and normalisation respectively. [file 1471-2164-13-181-S16.rtf]

>AlternativePrimerA, with 'V' replced by 'A': AAGCAGTGGTATCAACGCAGAGTCGCAGTCGGTACTTTTTTCTTTTTTVAAGCAGTGGTATCAACGCAGAGTCGCAGTCGGTACTTTTTTCTTTTTTA>AlternativePrimerC - with 'V' replaced by 'C'AAGCAGTGGTATCAACGCAGAGTCGCAGTCGGTACTTTTTTCTTTTTTC>AlternativePrimerG with 'V' replaced by 'G'AAGCAGTGGTATCAACGCAGAGTCGCAGTCGGTACTTTTTTCTTTTTTG>MINTadapter5prime | At the 5'end is added: 5'-AAGCAGTGGTATCAACGCAGAGTACGGGGG-3' : NOTE: Only upper- and lower-case A, C, G, T, and N characters should be used in Newbler trimming. Do not use X or N as masking characters.AAGCAGTGGTATCAACGCAGAGTACGGGGGSequences_for_trimming_abundant_sequences>gi|48476450|gb|AY550963.1| Sparus aurata myosin heavy chain-like mRNA sequenceAAAGAATTCGGCAGAGGNNGAAAATGTCCGTCAAATCAATGACACGAGTGCACAGAAAGCACGTCTTCTGACAGAAAATGGTGAGTTCGGCCGTCAAATTGAGGAGAAGGAAGCTCTGGTCTCCCAGCTGACCAGAGGCAAACAGGCCTTCACACAGCAGATTGAGGAGCTGAAGAGACAGACTGAAGAGGAGGTTAAGGCCAAGAATGCTCTTGCCCATGGACTGCAATCAGCCCGCCATGACTGTGACCTGCTGAGGGAGCAGTTTGAGGAGGAGCAGGAGGCCAAGGCTGAGCTGCAGCGTGGAATGTCCAAGGCCAACAGTGAGGTAGCATCAGTGGAGAACAAAGTATGAAACTGATGCTATCCAGCGCACTGAGGAGCTTGAGGAAGCCAAGAAGAAGCTGGCCCAGCGTCTCCAGGAGGCTGAGGAGCAGATTGAGGCAGTGAATTCCAAGTGTGCTTCTCTTGAGAAAACCAAACAGAGGCTTCAGAGTGAGGTGGAGGACCTCATGATTGATGTAGAGAGGGCCAATGGACTGGCTGCTAACCTGGACAAGAAGCAGAGGAACTTTGACAAGGTGTTGGCAGAGTGGAAACAGAAGTATGAGGAGGGTCAGGCAGAGCTTGAGGGATCTCAGAAAGAGGCTCGCTCTCTTGGCACTGAGCTGTTCAAGATGAAGAACTCTTATGAAGAAGCTCTGGATCAGCTGGAGACCATGAAGCGTGAAAACAAGAACCTGCAGCAGGAGATCTCAGATCTAACTAAACAGATCGGTGAGACTAATAAGAGCATNCATGAGCTGGNGANGGCCAAGNNGCAGGTGGGGACAGAGANGTCTGAGATCCAGACAGCTCTTGAAGAGGCTGAGGAAACTCTGGAACATGAAGAGTCCAAGATCCTGCGTGTCCAGCTGGAGCTCAACCAGATTAAAGGTGAGGTGGACAGGAAGCTGGCAGAGAAAGATGAAGAGATGGAGCAGATCAAGAGGAACAGCCAGAGGGTGACTGACTCCATGCAGGGCACCCTGGATTCTGAAGTCAGGAGCAGGAATGATGCCCTGAGAATCAAGAAGAAGATGGAGGGAGACCTGAATGAGATGGAGATTCAGTTGAGCCACGCCAATCGCCAGGCTTCTGAGTCCCAGAAGCAGCTGAGGCTTGTGCAGGCACAGCTGAAGGATGCCCAACTGCACCTTGATGATGCTGTCAGAGCTCAGGAAGACGTCAAGGAACAAGCTGCTATGGTGGAGCGCAGAAACGGTCTTATGGTAGCTGAAATTGAGGAACTTAGAGCTGCTCTGGAACAGACGGAGAGAAGCCGCAAAATTGCAGAGCAGGAGTTGGTGGATGCCAGTGAGCGTGTTGGACTTCTGCACTCTCAGAACACAAGCCTTATGAACACTAAGAAGAAGCTTGAGACTGATCTGGTCCAGATCCAGAGTGAAGTGGATGACACTGTTCAGGAAGCAAGGAATGCAGAGGAGAAGGCCAAGAAGGCCATCACTGATGCTGCCATGATGGCTGAGGAGCTGAAGAAGGAGCAGGACACTAGCGCTCACCTGGAGAGGATGAAGAAGAACCTTGAGGTTGCTGTTAAGGATCTGGTGCATCGCCTGGATGAGGCAGAGAACCTGGCAATGAAGGGTGGCAAGAAGCAGCTCCAGAAACTTGAGTCCAGGGTGCGTGAGCTGGAGACAGAGGTTGAGGCTGAGCAGAGACGTGGAGCAGATGCTGTTAAGGGTGTCCGCAAATATGAGAGGAGGGTGAAGGAGCTCACCTATCAGACTGAAGAGGACAAGAAAAATGTTACCAGGCTGCAGGATCTGGTTGACAAGCTGCAACTGAAGGTGAAGGCCTACAAGAGGCAGGCTGAGGAAACGGAGGAGCAGGCCAACGTTCATCTGTCCAAGTGCAGAAAGGTCCAGAACGAGCTGGAGGAGGCTGAGGAGCGCGCTGACATTGCAGAGTCCCAGGTCAACAAAATGAGAGTAAAGTCCCGTGATTCCGGCAAGGGAAAAGAGGCAGCAGAGTAACGCGCTGGCTGATCAGGAGTTTTATAATCATATAATATGATGTGAAATACACTGTATAAATAAATGTTTACTTCTCACTCTTAAAAAAAAAAAAAAAAAAAAAAAAAAAAAAAAAAAAAAAAAA>gi|33340044|gb|AF304559.1| Sparus aurata troponin mRNA, partial cdsTTCGTGGAGAGGGACCAGGCCGGCAAGAGCGAGGAAGAGTTGGCAGAGTGCTTCCGCGTGTTCGACAAGAACGGCGACGGCTACATCGACAGAGAGGAGTTCGCCATCATCATCCTTATAAACATCCTCAACAGACAGCTAGGGCACCTGGCAGGGGTCGCTGGCTGGGGCTACACTCTTCCCCAGCCTCGCTTGTGATTTGCATTTTATTAACAGGCACGTGTCCATCAGCGACCCCCTCCCCCCATGCTGGTGAAGCTAATCTGATCTAGAATCTAGTCTAGAGAGAACAGCAGCCTGTTGTCTGCTGCTTAGTCCAATTAAGTCAACCAATTCACTGTACAAAGTAATTTGCAAGAACAGAATAAATGAACTAACTCATGCTCACTTGTAGTTGGTCTGTCGTATTTATGGTCTGTGTAAAAAAATTTTGTTTTGAGGAATATCAATAAAATCTGTTTGTTTCTTC>gi|6653227|gb|AF190473.1| Sparus aurata skeletal alpha-actin mRNA, complete cdsCAACAGCACTTGTTGGGTCTTTCTCTCTCCGAGCCGCAGACACACTCCTAAGAAGCCATCATGTGTGACGACGATGAGACTACCGCCCTTGTGTGCGACAACGGCTCAGGCCTGGTGAAGGCTGGCTTCGCCGGAGACGATGCCCCCAGGGCAGTGTTCCCCTCCATCGTCGGCCGCCCCCGTCATCAGGGTGTCATGGTCGGTATGGGTCAGAAGGACTCCTACGTCGGCGACGAGGCCCAGAGCAAGAGGGGTATCCTGACTCTGAAGTACCCCATCGAGCACGGCATCATCACCAACTGGGACGACATGGAGAAGATCTGGCACCACACCTTCTACAACGAGCTGAGAGTTGCCCCCGAGGAGCACCCCACCCTGCTCACTGAGGCTCCCCTCAACCCCAAGGCCAACAGAGAGAAGATGACCCAGATCATGTTTGAGACCTTCAACGTCCCCGCCATGTATGTTGCCATCCAGGCTGTGCTGTCCCTGTACGCTTCCGGTCGTACCACTGGTATTGTGCTGGATGCTGGTGATGGTGTGACCCACAACGTCCCAGTCTATGAGGGTTACGCCCTGCCCCACGCCATCATGCGTCTGGACCTGGCTGGTCGCGATCTTACCGACTACCTGATGAAGATCCTGACTGAGCGTGGCTACTCCTTCGTCACCACCGCCGAGCGTGAGAATCGTGCGCGACATCAAGGAGAAGCTTGCTATGTGGCTCTGGACTTCGAGAACGAGATGGCCACCGCTGCCTCTTCTTCCTCTCTGGAGAAGAGCTACGAGCTTCCCGACGGTCAGGTCATCACCATCGGTAACGAGAGGTTCCGTTGCCCCGAGACCCTCTTCCAGCCTTCCTTCATTGGTATGGAGTCTGCTGGTATCCATGAGACCGCCTACAACAGCATCATGAAGTGCGACATTGACATCCGTAAGGATCTGTACGCCAACAATGTGCTCTCCGGTGGTACCACCATGTACCCTGGTATTGCTGACCGTATGCAGAAGGAGATCACTGCTCTGGCCCCCAGCACCATGAAGATCAAGATCATTGCCCCTCCCGAGAGGAAGTACTCCGTCTGGATCGGTGGCTCTATCCTGGCTTCCCTGTCCACCTTCCAGCAGATGTGGATCTCCAAGCAGGAGTACGACGAGGCAGGCCCCAGCATTGTCCACAGGAAGTGCTTCTAAATCTTCCATCTTCTTCTCCAGCTTCTCCATCATCTCCACAAACACCACCAGCTATCCGGGGATCATGCGAGAAGGAGGATCAACCTCTGCTGCACAGCAACACTCTGCTGTCAATGGACTTTCTGTCTGCGCTGCTGACATTCATATGCATTTTGTTATCCTTTTAAAAATGTGCATATTTTAGAGCTGTCTGTTGTCTGTGTTGCACGGAGAGAGGCTGTGAAAACATCACTGAACCATGCATCCACCCAAAAAAAAACTTTAATAAAAAAATAACAACAAAACATGTTGCCCAACACATGTGAATGTAAGTGTATATACATATTGAT>gi|34014737|gb|AY362763.1| Sparus aurata beta-actin mRNA, partial cdsGACCAACTGGGATGACATGGAGAAGATCTGGCATCACACCTTCTACAACGAGCTGAGAGTTGCCCCTGAGGAGCACCCAGTCCTGCTCACAGAGGCCCCCCTGAACCCCAAAGCCAACAGGGAGAAGATGACCCAGATCATGTTCGGGACCTTCAACACCCCCGCCATGTACGTTGCCATCCAGGCTGTGCTGTCCCTGTATGC>gi|5852837|gb|AF150904.1| Sparus aurata myosin light chain 2 mRNA, complete cdsTGGCTTTGGCTTAGGCTTCTCTTCTTGACCACCAACAACCCCAGAAACTTGAGGATGGCACCCAAGAAGGCCAAGAGGAGGCAGCAGCAGGGCGAGGGTGGATCCTCCAATGTGTTCTCCATGTTTGAGCAGAGCCAGATCCAGGAGTACAAGGAGGCTTTCACAATCATTGACCAGAACAGAGATGGCATCATCAGCAAGGACGATCTTAGGGACGTGCTGGCCACCATGGGCCAACTGAATGTGAAGAATGAGGAGCTGGAGGCCATGGTGAAGGAGGCCAGCGGCCCCATCAACTTCACCGTCTTTCTGACCATGTTCGGCGAGAAGCTGAAGGGTGCTGATCCCGAGGACGTCATCGTGAGCGCTTTCAAGGTCCTGGACCCCGAGGCCACTGGCGCCATCAAGAAGGAATTCCTTGAGGAGCTCCTGACCACCCAGTGCGACAGGTTCACCGCTGAGGAGATGACCAACCTGTGGGCTGCTTTCCCCCCTGATGTGGCTGGCAATGTGGACTACAAGAACATCTGCTACGTCATCACACACGGAGAAGAGAAGGAGGAATAAATCCCCCTCTCTTTCAAGATCCTTACCTCCGCTCAAATCCCATATACTCGACGCAACATCTACTCTACTCACTCTTCTCCGATGCCGTGGCTCCCTCGGACACTCTCGCGCCCTCGGCCCGCTCTGTCGCTTTGCAGCTCACTACAAAAAGAACTTGTCTCCTGTTCTTGAGATACTCAGTGAGAGGACTGGGGGCTGTGGGGTTGTTTGTGTGTGATTACCAACAGGTGAACATGGGATTATTTTCAATAAAAATAATCCTTGTGGCACTGAAACTCTCTCTCCATCTCTGTCCCTGCCTCTTGTTCCCCCTGCTTTTCCTCCCATCACTCATTCTGTCCTTCTGCGTTGACGCCAACAGTGCATGCATCATGCCTATGTACAGCGCGTATGCATATGCAGTCCAGTGTATACAGTGGCCAGTCAGACATATCTCTTGGGTGCTGTGGTGCAAGCACAGCCGCTCACTTCAAACAAGTAAGCGGCCTGACCCGAGTGGTCTGTTAGTCTCAACCTGACACAGAGTGTTTTATGGACTCGTCCCTTTGTTTGTATCAGGGAGGATAGCACAGTGAAGAGTGGGAGTACCGTACTATAATAGATTGCCTACTCCTTCTCTTTAATCTGTCTCTCCTTCTCTTAAACACAGGCATGACAGGAAAAGTTGCAGTGAAAATGGGAAAGCATGATTTGGTTCAAATCTTGTAATTGGAGAAAGAGATGGTGAAAGATGGTGAGTGGGAGGGAGAGATGAAATAAACGAAAGTGAAATGTCTTGTTTTGGTCTCTTTTTTCTCCGACTCACTGCTGTTTCTCTCCTGTTTTCATGACTGTACCAAATAAAGAAGTACAAATAAAATCCACTATCTTTCGTAAAAAAAAAAA>gi|5852835|gb|AF149756.1| Sparus aurata fast skeletal myosin light chain 3 mRNA, complete cdsCAACTCTCCAACATGACCGAACAGGCCGAGTTCAGTGCCGACCAGATTGAGGACTTCAAGGAGGCTTTCGGTCTCTTTGACAGAGTCGGTGACAGCCAGGTGGCCTTCAACCAGGTTGCTGACATCATGCGCGCTCTGGGCCAGAACCCCACCAACAAGGACGTTACAAAGATTCTGGGCAACCCCTCCGCCGACGACATGGCCAACAAGAGGCTCAACTTCGAGGCTTTCCTGCCCATGCTGAAGGAGGTCGACGCCTTGCAGAAGGGCACCTACGACGACTACGTTGAGGGCCTGCGCGTCTTCGACAAGGAGGGCAACGGCACAGTCATGGGCGCTGAGCTGCGCATCGTGCTGTCCACCCTGGGAGAGAAGATGACCGAGCCTGAGATTGATGCCCTCATGGCCGGCCAGGAGGACGAGAACGGCAGTTTGCACTATGAGGCTTTCGTCAAGCACATCATGTCTGTGTAAGAGGCCGGCAGCAGGAGTGCTGAAGAACAGCCCGACGGTGTTCAGGACATCTACACTGTTGTCAAAGACCAACCAAGGAAAGACAAGGACTATGTACAAGGGATGTTGAAGCAAACCCATCTTGTTGTTTTATTTTGTTTTTCCCTTTCCATTTCGTCTCACMCSCCCTCCTCCCTCTCTCGGGACGCCACCATCACCTCTACACCGTAGACCCACCACCACTCGGCCTCCACTCCCCCCTACCCTCCGGGGCACGTCTACACATGCGCATGGGTGAGGAACGGGGAAAAGGGGTGAGCGCTCATCAAGTGAGGGTAGATCCTCCCTTCCCACCGCCACTCATTCAGTCTCGCCATCACTGGTCCAGCAATGCAAAGGTGCTGAGAATGGTCGTGGATAGACGCCAAAAAAGTCTCCCCGCTTCCCTGAAAAGTTTTATTTATTGTCACTCTGTTCATGTCAGAATAAACTTTTCCAACGTAAAAAAAAAAAAAAAAA>gi|32895273|gb|AY326430.1| Sparus aurata alpha-tubulin mRNA, partial cdsCTGGACAGGATCCGCAAACTGGCTGACCAGTGCACAGGCCTCCAAGGTTTYCTCATCTTCCACTCCTTTGGTGGAGGAACCGGCTCTGGCTTCACCTCTCTGCTGATGGAGCGCCTTTCTGTCGACTACGGCAAAAAGTCCAAGCTGGAGTTTGCCGTGTACCCAGCTCCCCAGGTGTCCACGGCCGTGGTGGAGCCATACAACTCCATCCTGACCACCCACACCACCCTGGAGCACTCCGACTGCGCCTTCATGGTGGACAATGAGGCCATCTATGACATCTGCCGCAGAAACCTGGACATCGAGCGTCCGACCTACACCAACCTCAACAGGCTGATTGGACAGATCGTCTCCTCCATCACCGCCTCCCTGCGCTTCGACGGCGCCTTGAACGTGGACCTGACGGAGTTCCAGACCAACTTGGTGCCCTACCCTCGTATCCACTTCCCTCTGGCCACCTACGCCCCCGTTATCTCAGCAGAGAAGGCCTATCATGAGCAGCTGTCAGTTGCTGACATCACCAATGCCTGCTTCGAGCCCGCCAACCAGATGGTCAAATGCGACCCTCGCCATGGGAAGTACATGGCCTGCTGCCTCCTGTACCGTGGTGACGTTGTACCCAAAGATGTGAACTCCGCCATCGCCACCATCAAAACCAAGCGTACCATCCAGTTTGTGGACTGGTGTCCCACAGGCTTCAAGGTGGGCATCAACTACCAGCCTCCCACAGTGGTTCCTGGAGGAGATCTGGCCAAGGTGCAGAGGGCCGTGTGCATGCTGAGCAACACCACCGCCATYGCCGAGGCCTGGGCTCGTCTCGACCACAAGTTCGACCTGATGTACGCCAAGAGGGCCTTCGTCCACTGGTACGTCGGTGAGGGTATGGAAGAGGGTGA>gi|15281826|gb|AF398343.1|AF398343 Danio rerio 28S ribosomal RNA gene, partial sequence 1500-2400GCCGAAACGATCTCAACCTATTCTCAAACTTTAAATGGGTAAGAAGCCCGGCTCGCTGGCTTGGAGCCGGGCGTGGAATGCGAGACGCCCAGTGGGCCTTTTTTGGTAAGCAGAACTGGCGCTGCGGGATGAACCGAACGCCGGGTTAAGGCGCCCGATGCCGACGCTCATCAGACCCCAGAAAAGGTGTTGGTTGATATAGACAGCAGGACGGTGGCCATGGAAGTCGGAATCCGCTAAGGAGTGTGTAACAACTCACCTGCCGAATCAACTAGCCCTGAAAATGGATGGCGCTGGAGCGTCGGGCCCATACCCGGCCGTCGACGGCAGAAGGAGCCCCCCCCTCCGGGGGGGGAAAGGGTAAGCCTCGACGAGTAGGAGGGCCGCCGCGGTGGCGCGGAAGCCTAGGGCGTGGGCCCGGGTGGAGCCGCCGCGGGTGCAGATCTTGGTGGTAGTAGCAAATATTCAAACGAGAGCTTTGAAGGCCGAAGTGGAGAAGGGTTCCATGTGAACAGCAGTTGAACATGGGTCAGTCGGTCCTAAGGGATGGGCGAACGCCGTTCGGAAGGGAGGAGCGATGGCCTCCGTCGCCCCCGGCCGATCGAAAGGGAGTCGGGTTCAAATCCCCGAACCCGGAGTGGCGGAGACGGGCGCCGCGAGGCGCCCAGTGCGGTGACGCAAACCAACCCGGAGAAGCCGGCGGGAGCCCCGGGAAGAGTTCTCTTTTCTTTGTGAAGGGCAGGGCGCCCTGGAATGGGTTCGCCCCGAGAGAGGGTGTAAATCTCGCGA>gi|148614872|gb|EF417169.1| Danio rerio large subunit ribosomal RNA gene, partial sequenceGCCCAGCGCCGAATCCCCGCCCCGTGCCAGGGGCGAGGGAAATGTGGCGTACGGAAGATCGCTCTCTCTCGGCGCGGGCCGGGGGTCTAAGTCCTTCTGATGGAGGCTTAGCCCGTGGACGGTGTGAGGCCGGTAACGGCCCCCGCCCCGCCGGGGTACGGTCTTCCCGGAGTCGGGTTGTTTGGGAATGCAGCCCAAAGCGGGTGGTAAACTCCATCTAAGGCTAAATACCGGCACGAGACCGATAGTCGACAAGTACCGTGAGGGAAAGTTGAAAAGAACTTTGAAGAGAGAGTTCAACAGGGCGTGAAACCGTTGAGAGGTAAACGGGTGGGGTCCGCACGGTCCGCCCGGAGGATTCAACCCGGCGGGGCTGGTCGGCCCGTCCGGTGCGCTCGGATCCCCCCCCTCTCGGGGGAGGGGGACCGGCGCCCGGACGGAGCTCGGCCGCCGCCGGGCGCACTTCCTCCGTCGGCGGTGCGCCGCGACCGGCTCCGGTTCGGCTTGGAAGGGTTCGGGGGCGAAGGTGGCTCGCGGCTCCGGCCTCGAGCTTTACAGCGCCCCCYCGCCCCGACTTCGCCGCTTGCTACCCGGGGCCGCGGGCAGTGTCCTCCGCGCCTTCTCTCCGGCCCCTCTCTCGGGGGGGGCTCGGAGGGACGGGGCCCCTCGCTCCCGGCGCGTGCGTCGACCGGAACGGACTGTCCTCAGTCCGTTTCCGACCGCGCCGTGCCGCCCAGGGCGGGGACAGGCCCACGTCCAAAAGGGCGCCCGAGGTCCGCGGTGATGCCGGCCACCCACCCGACCCGTCTTGAAACACGGACCAAGGAGTCTAACGCGCGCGCGAGTCAGAGGGTGCCCTCGAGCCCCCACGGCGCAATGAAGGTGAAGGCCGGCGCGCGCCGGCCCAGGTGGGATCCCCCCGCCCCGGCGGGGGGCGCACCACCGGCCCGTCTCGTCCGCTCTGTCGGGGAGGTGGAGCTAGAGCGCGTGCGATGGTACCCGAAAGATGGTGAACTATGCCTGGGCAGGGCGAAGCCAGAGGAAACTCTGGTGGAGGCCCGCAGCGGTCCTGACGTGCAAATCGGT
